# Site‐Selective C−H Oxygenation via Aryl Sulfonium Salts

**DOI:** 10.1002/anie.201908718

**Published:** 2019-09-24

**Authors:** Ruocheng Sang, Stamatis E. Korkis, Wanqi Su, Fei Ye, Pascal S. Engl, Florian Berger, Tobias Ritter

**Affiliations:** ^1^ Max-Planck-Institut für Kohlenforschung Kaiser-Wilhelm-Platz 1 45470 Mülheim an der Ruhr Germany

**Keywords:** C−H functionalization, C−O bond formation, hydroxylation, photo-redox, site-selectivity

## Abstract

Herein, we report a two‐step process forming arene C−O bonds in excellent site‐selectivity at a late‐stage. The C−O bond formation is achieved by selective introduction of a thianthrenium group, which is then converted into C−O bonds using photoredox chemistry. Electron‐rich, ‐poor and ‐neutral arenes as well as complex drug‐like small molecules are successfully transformed into both phenols and various ethers. The sequence differs conceptually from all previous arene oxygenation reactions in that oxygen functionality can be incorporated into complex small molecules at a late stage site‐selectively, which has not been shown via aryl halides.

## Graphical abstract

**Efficient diversification**: The first site‐selective late‐stage aromatic C−O bond formation from arene C−H bonds proceeds in two steps via arylthianthrenium intermediates. Electron‐rich, ‐poor and ‐neutral arenes as well as complex drug‐like small molecules are successfully transformed into both phenols and various ethers.

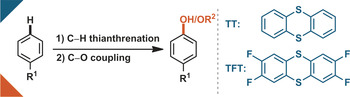

Site‐selective late‐stage C−O bond formation is currently not achievable for most molecules, especially if they are functionally complex.^[1]^ Carbon–oxygen bonds are present in a broad range of chemicals, including but not limited to polymers, intermediates for synthesis, and in several blockbuster pharmaceuticals.^[2]^ Phenols and aryl ethers can be conveniently prepared by a variety of reliable methods,^[3]^ yet, no method has been shown to regioselectively oxygenate in a general sense, unless a functional group to be converted to the oxy substituent is already present in the molecule. For example, C−O bond formation by cross coupling from aryl halides proceeds well,^[3]^ but no halogenation reaction is currently available that proceeds highly regioselectively for most arenes.^[4]^ Here we report a two‐step procedure to selectively access phenols and aryl ethers from complex small molecule arenes that are not, or at least not selectively, readily accessible by other methods. The transformation represents the first example to synthetically access phenol derivatives from aryl thianthrenium salts.

Processes such as the Vilsmeier–Haack formylation followed by Dakin oxidation,^[5]^ and the Hock process,^[6]^ are used to synthesize phenols on an industrial scale. Transition metal‐promoted reactions,^[3]^ such as the Sandmeyer hydroxylation,^[7]^ Ullmann‐type reactions,^[8]^ Chan–Lam couplings,^[9]^ palladium‐catalyzed C−O bond forming reactions,^[10]^ and various others,[^11^, ^12^, ^13^] have been developed to install C−O bonds. Most of the transition metal‐promoted reactions are performed on aryl halides or arylboronic acids, and are robust transformations for phenol and aryl ether synthesis. However, installing the halide or boryl group with high site‐selectivity is challenging or not currently possible on complex molecules, except for arenes with specific substituents or substitution patterns.[^4^, ^11^, ^14^, ^15^] Direct C−H oxygenation reactions are advantageous because they can obviate the pre‐functionalization step, but typically afford mixtures of constitutional isomers.^[1]^ Taken together, there is a vast history of aryl C−O bond‐forming reactions, but neither one‐step nor two‐step procedures are currently available to install oxygen functionality into complex arenes with high site‐selectivity. Here, we fill this void in the field.

Previously, we have disclosed a site‐selective C−H functionalization of arenes by thianthrenation,[^4^, ^16^] but did not identify the utility of aryl thianthrenium salts for the field of C−O bond formation. Herein, we report the development of a highly site‐selective C−O bond forming process via aryl thianthrenium salts, in which oxygen‐nucleophiles such as water, phenols, primary, and secondary alcohols are coupled with arenes including drug‐like small molecules, such as flurbiprofen methyl ester (**1**) (Scheme 1 b).

**Scheme 1:**
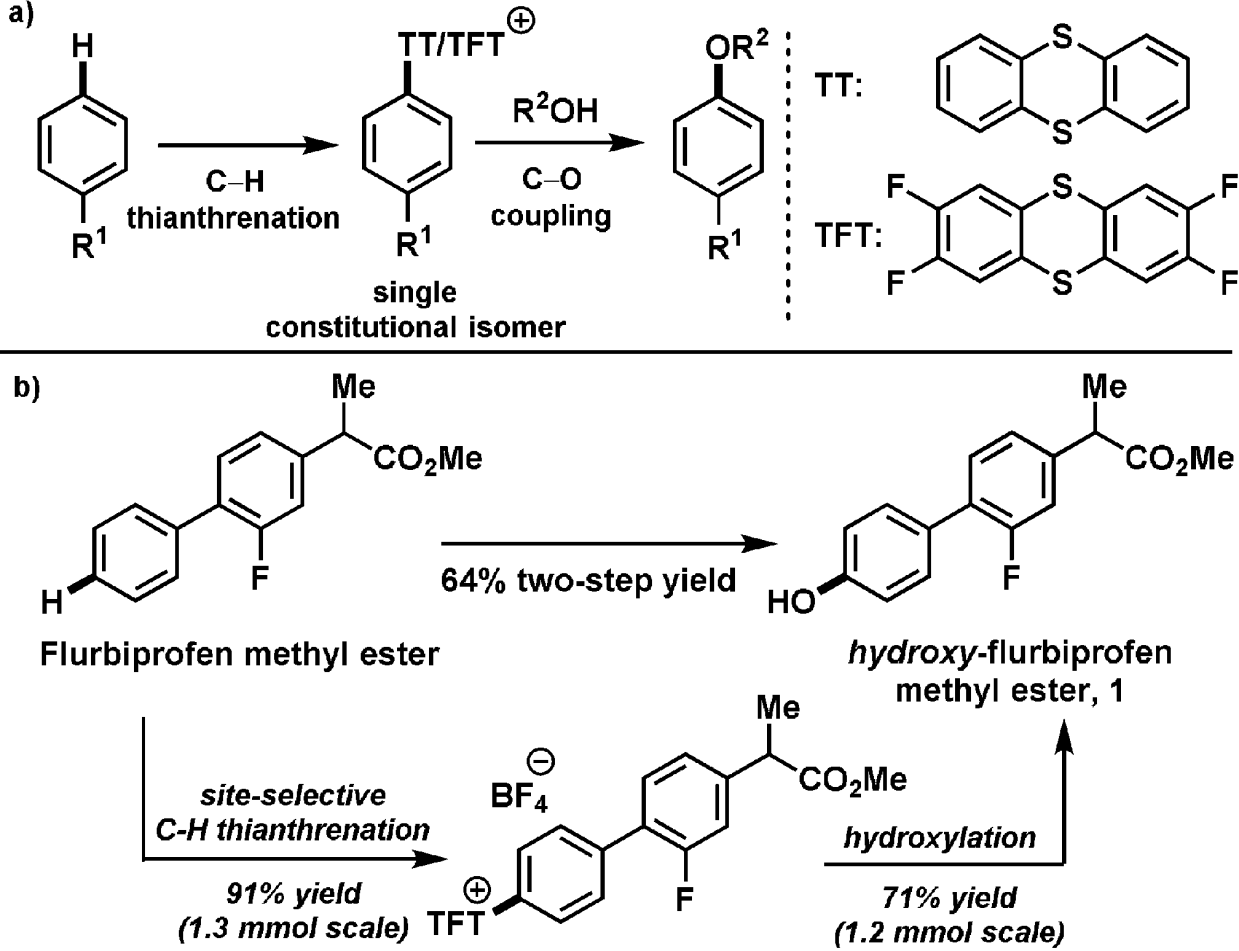
a) Site‐selective C−O bond formation. b) An example of site‐selective C−H thianthrenation followed by photoredox‐mediated hydroxylation.

Hydroxylation of arylthianthrenium salts can be accomplished with water as nucleophile under photoredox conditions (Table 1). The optimized reaction conditions for the hydroxylation of biphenyl‐derived thianthrenium salt are 1 mol % [Ir[dF(CF_3_)ppy]_2_(dtbpy)PF_6_] as photoredox catalyst, 0.8 equiv of Cu_2_O, and 10 mol % of dimethylglyoxime, presumably as ligand for copper, in acetonitrile that afforded 4‐phenylphenol (**22**) in 80 % yield (for optimization, see Supporting Information). We used commercially available copper sources that are less expensive than the valuable small molecules typically employed for late‐stage functionalization; smaller amounts of copper result in lower conversion. The amount of dimethylglyoxime is crucial (see Supporting information, Table S1), with larger amounts of dimethylglyoxime resulting in hydrodefunctionalized side‐product. Both arylthianthrenium (Ar‐TT^+^) and tetrafluorothianthrenium (Ar‐TFT^+^) salts could be successfully hydroxylated, typically, the TFT salts gave higher yield (see Supporting Information, Figure S1). Based on the higher reduction potential of arylthianthrenium salts [*E* ^0^ (ArTT^+^/ArTT^.^) ≅ −1.5 V vs. SCE in CH_3_CN] when compared to aryl halides, single electron reduction of the thianthrenium moiety is possible at a potential that copper(III) is feasible,^[17]^ from which facile C−O reductive elimination could occur.^[18]^ A Stern–Volmer analysis revealed that reductive quenching of the excited iridium(III) photocatalyst by thianthrene is faster than oxidative quenching by the thianthrenium salt. The subsequently formed iridium(II) has a suitable reduction potential for the reduction of the arylthianthrenium salt, which results in aryl radicals that can undergo an oxidative ligation to afford copper(III) aryl complexes (Scheme 2 a). Consistent with the formation of aryl radicals, we could observe the 2,2,6,6‐tetramethylpiperidin‐1‐oxyl (TEMPO) adduct (**52**) upon addition of TEMPO to the reaction mixture (Scheme 2 b).

**Scheme 2:**
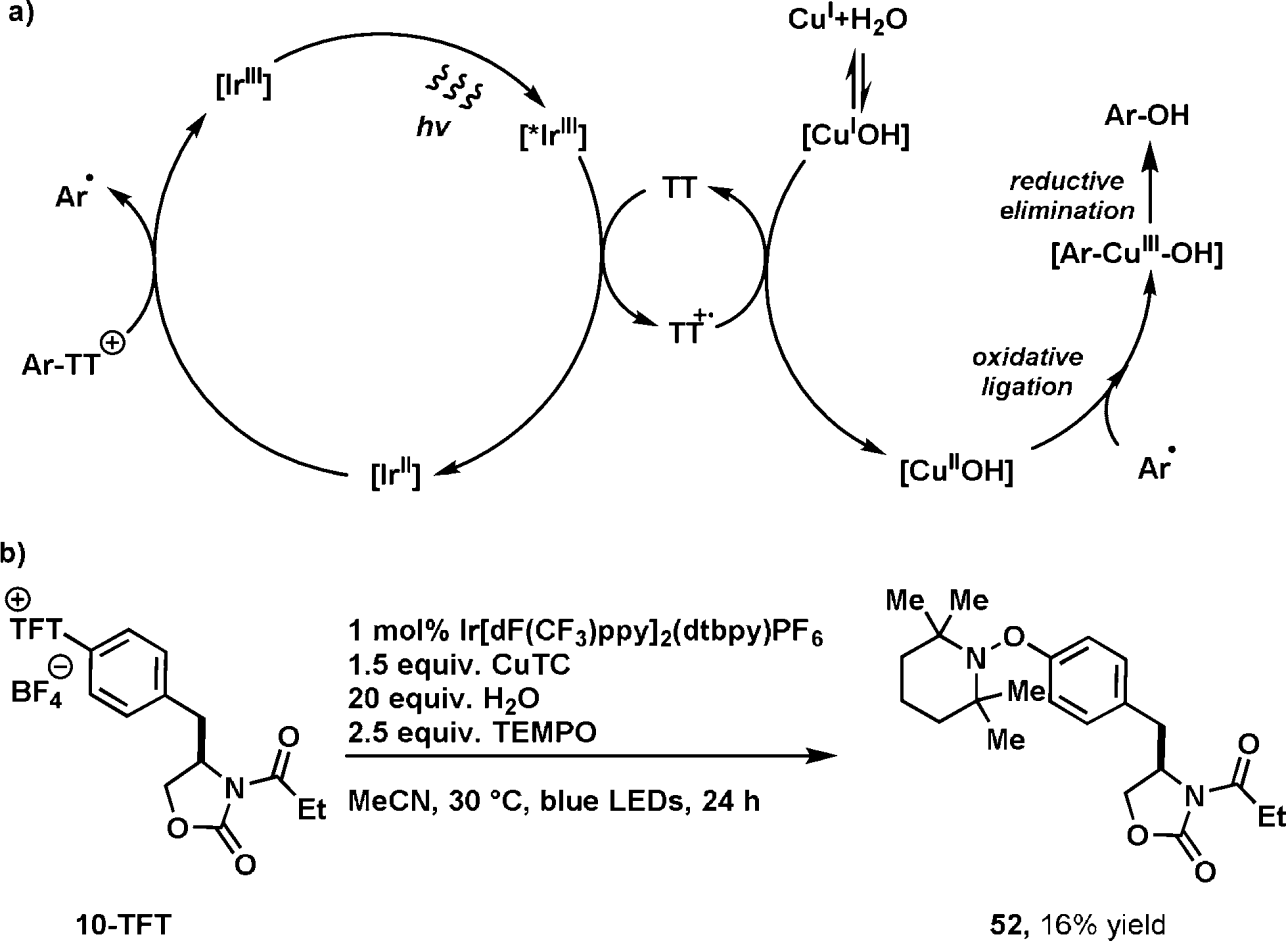
a) Reaction design. b) Radical trapping experiment: reaction conditions: thianthrenium salt (1.0 equiv), TEMPO (2.5 equiv), [Ir[dF(CF_3_)ppy]_2_(dtbpy)PF_6_] (1 mol %), CuTC (1.5 equiv), H_2_O (20 equiv), MeCN, blue LED (34 W), 30 °C, 24 h.

**Table 1:** Substrate scope of site‐selective hydroxylation of arenes. 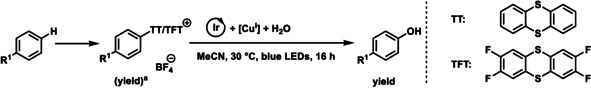

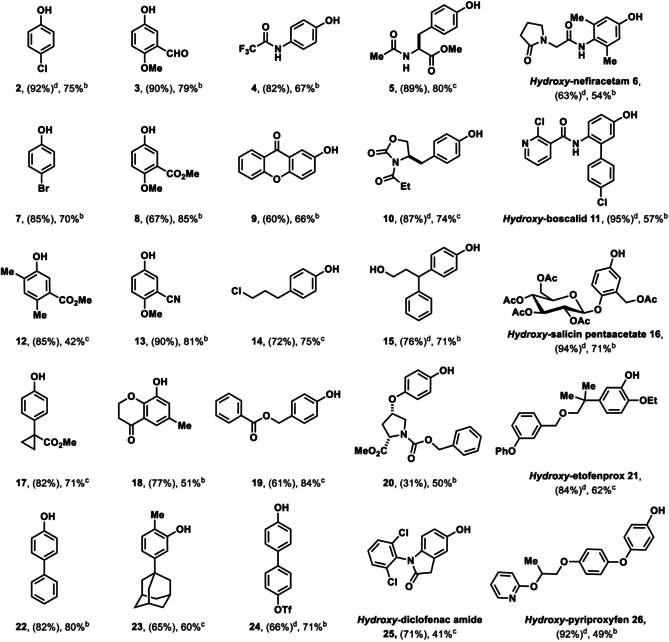

[a] Yield of the thianthrenation. [b] Yield of the hydroxylation. Reaction conditions: thianthrenium salt (1.0 equiv), [Ir[dF(CF_3_)ppy]_2_(dtbpy)PF_6_] (1 mol %), dimethylglyoxime (10 mol %), Cu_2_O (0.8 equiv), MeCN/H_2_O (10/3, v/v), blue LED (34 W), 30 °C, 16 h. [c] Yield of the hydroxylation. Reaction conditions: thianthrenium salt (1.0 equiv), [Ir[dF(CF_3_)ppy]_2_(dtbpy)PF_6_] (1 mol %), CuTC (1.5 equiv), H_2_O (20 equiv), MeCN, blue LED (34 W), 30 °C, 16 h. [d] Yield of the thianthrenation from Ref. ^[4]^.

The scope of the hydroxylation spans from electron‐deficient (**2**, **7**) to electron‐rich arenes (**4**, **6**). A wide range of functional groups are tolerated including cyclopropyls, aldehydes, esters, nitriles, ethers, ketones, protected anilines, protected amino acids (**5**), amides, heterocycles (**11, 26**) and carbamates. Importantly, substrates containing protic groups such as alcohols, halides and pseudohalides (**24**), which can be incompatible with transition metal‐catalyzed hydroxylation reactions,[^8^, ^10^] are tolerated in our process. The basic conditions used for most other cross coupling protocols to introduce hydroxide can lead to side reactions, such as hydrolysis of esters like in compound **20**, or epimerization.^[8j]^ In our protocol, the base‐sensitive methyl ester in **20** was tolerated and no epimerization of the stereocenter was observed. Several pharmaceuticals were hydroxylated site‐selectively (**1**, **6**, **11**, **16**, **21**, **25**, **26**). Occasionally, the hydroxylation of alkyl substituted arenes can be problematic in the sense that hydrodefunctionalized sideproducts are formed with Cu_2_O; in such cases copper(I) thiophene‐2‐carboxylate (CuTC) could be used successfully to circumvent the problem to give the desired phenols (**5**, **10**, **12**, **14**, **19**, **23**) in higher yields.

We could generalize the transformation beyond phenol synthesis to also include the formation of ethers with phenols as well as primary and secondary alcohols (Table 2). The use of CuTC instead of Cu_2_O was crucial to obtain high yields, as was the use of Na_2_CO_3_ as base. Otherwise, substantial amounts of hydrodefunctionalized side‐products were observed. The use of Na_2_CO_3_ additionally reduced the formation of aryl thiophene‐2‐carboxylate (i.e. Ar‐TC) that was observed in the absence of base and was also beneficial to increase the conversion of the transformation. In the absence of Na_2_CO_3_, Ar‐TC byproduct was observed in up to 42 % yield (see Supporting Information, Table S2).

**Table 2:** Substrate scope of site‐selective etherification of arenes. 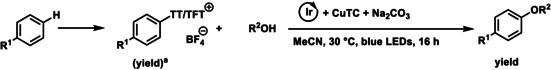

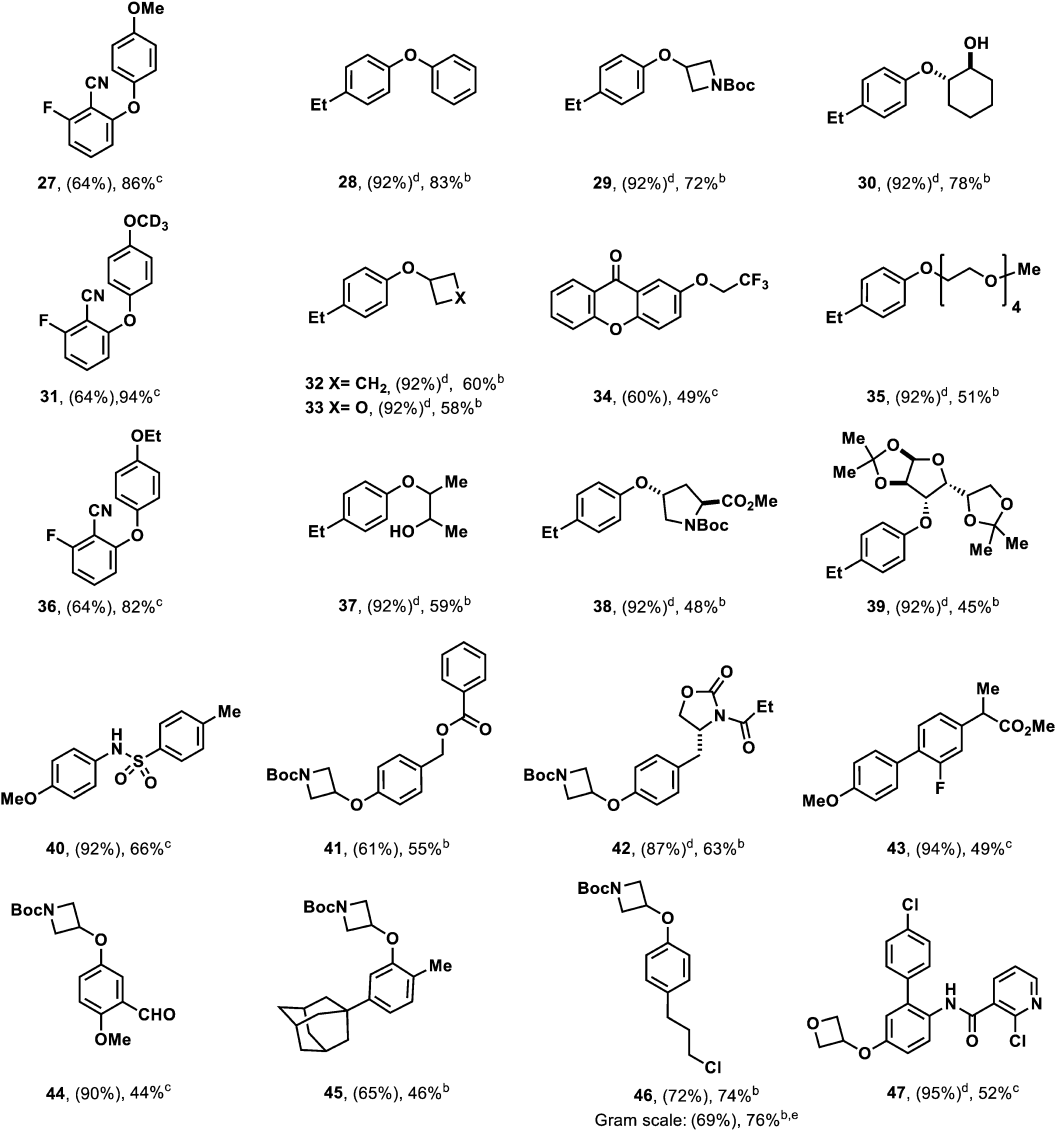

[a] Yield of the thianthrenation. [b] Yield of the etherification. Reaction conditions: ROH (2.0 equiv), CuTC (1.0 equiv), Na_2_CO_3_ (1.0 equiv), 3 Å MS, MeCN, 2 h; followed by addition of thianthrenium salt (1.0 equiv), [Ir[dF(CF_3_)ppy]_2_(dtbpy)PF_6_] (1 mol %), blue LED (34 W), 30 °C, 16 h. [c] Yield of the etherification. Reaction conditions: thianthrenium salt (1.0 equiv), [Ir[dF(CF_3_)ppy]_2_(dtbpy)PF_6_] (1 mol %), CuTC (1.0 equiv), MeCN, blue LED (34 W), 30 °C, 16 h. [d] Yield of the thianthrenation from Ref. ^[4]^ [e] Gram scale synthesis of **46**: 1.36 g, 52 % two‐step yield

Primary alcohols including methanol, d_4_‐methanol, ethanol, and 2,2,2‐trifluoroethanol were coupled in 49–94 % yield. Additionally, 4‐membered cyclic alcohols such as cyclobutanol, 3‐hydroxyoxetane and *N*‐Boc protected 3‐hydroxyazetidine were successfully employed. Coupling with phenol, 1,2‐diols (**30**, **37**), as well as alcohols of higher complexity (**35**, **38**, **39**) also proceeded to give the corresponding ethers. Beta hydride elimination of secondary alcohols, which is a common challenge for transition‐metal‐catalyzed C−O bond formation to access aryl ethers[^10e^, ^10g^] was not observed. The site‐selective etherification exhibits a similar functional group tolerance as the hydroxylation and can be performed on gram scale (**46**). A range of functional groups including nitriles, ketones, aldehydes, esters, carbamates, amides, halides, sulfonamides (**40**) and heterocycles (**47**), as well as substrates bearing an *ortho*‐substituent (**45**) are tolerated. We also demonstrated that complex drug‐like molecules can be alkoxylated in high site‐selectivity (**43**, **47**). Similar to ether bond formation, thioether bond formation could be accomplished under similar reaction conditions, with tetramethylenediamine (TMEDA) as additional reaction component, presumably as ligand for copper (Table 3).

**Table 3:** Substrate scope of site‐selective thioetherification of arenes. 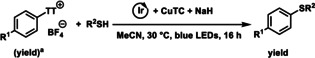

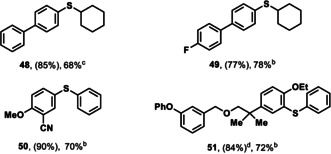

[a] Yield of the thianthrenation. [b] Yield of the thioetherification. Reaction conditions: thianthrenium salt (1.0 equiv), RSH (2.0 equiv), [Ir[dF(CF_3_)ppy]_2_(dtbpy)PF_6_] (1 mol %), TMEDA (20 mol %), CuTC (1.0 equiv), NaH (4.0 equiv), MeCN, blue LED (34 W), 30 °C, 16 h. [c] no TEMDA. [d] Yield of the thianthrenation from Ref. ^[4]^.

In conclusion, we have reported the first site‐selective late‐stage aromatic C−O bond formation synthesizing phenols and arylethers from arene C−H bonds in two steps via arylthianthrenium intermediates. We envisage that the reaction will be enabling for late‐stage diversification, especially in drug discovery.

## Conflict of interest

F.B. and T.R. may benefit from royalty payments of thianthrenium derivatives.

## Supporting information

As a service to our authors and readers, this journal provides supporting information supplied by the authors. Such materials are peer reviewed and may be re‐organized for online delivery, but are not copy‐edited or typeset. Technical support issues arising from supporting information (other than missing files) should be addressed to the authors.

Supplementary
