# Supplementary material for: Site‐Selective C−H Oxygenation via Aryl Sulfonium Salts
Source: Angew Chem Int Ed Engl. 2019 Sep 24;58(45):16161–6. doi: 10.1002/anie.201908718 (PMC7754133; doi:10.1002/anie.201908718)
Supplement: Supplementary file 1 — Supplementary [file ANIE-58-16161-s001.pdf]

Angewandte Chemie
Eine Zeitschrift der Gesellschaft Deutscher Chemiker

Supporting Information

Site-selective C–H Oxygenation via Aryl Sulfonium Salts

*Ruocheng Sang, Stamatis E. Korkis+, Wanqi Su+, Fei Ye, Pascal S. Engl, Florian Berger, and Tobias Ritter**

anie_201908718_sm_miscellaneous_information.pdf

TABLE OF CONTENTS

TABLE OF CONTENTS 1

MATERIALS AND METHODS 11

EXPERIMENTAL DATA 12

General procedure and reaction condition optimization for hydroxylation 12

General procedure of hydroxylation with Cu_2O 12

General procedure of hydroxylation with CuTC 12

Table S1. Reaction condition optimization 13

Figure S1. Comparison of the hydroxylation of TT and TFT salts 14

Figure S2. Comparison of the hydroxylation with Cu_2O and CuTC 14

Thianthrenation and hydroxylation of arenes 15

Flurbiprofen methyl ester tetrafluorothianthrenium salt (**1-TFT**) 15

Flurbiprofen methylester thianthrenium salt (**1-TT**) 16

Hydroxy-flurbiprofen methyl ester (**1**) 16

4-Chlorophenol (**2**) 17

2-Methoxybenzaldehyde tetrafluorothianthrenium salt (**3-TFT**) 18

5-Hydroxy-2-methoxybenzaldehyde (**3**) 19

2,2,2-Trifluoro-N-phenylacetamide tetrafluorothianthrenium salt (**4-TFT**) 19

2,2,2-Trifluoro-N-(4-hydroxyphenyl)acetamide (**4**) 20

Methyl acetyl-L-phenylalate tetrafluorothianthrenium salt (**5-TFT**) 21

Methyl acetyl-L-tyrosinate (**5**) 22

Hydroxy-nefiracetam (**6**) 23

Bromobenzene tetrafluorothianthrenium salt (**7-TFT**) 23

4-Bromophenol (**7**) 24

2-Methoxybenzoate tetrafluorothianthrenium salt (**8-TFT**) 25

5-Hydroxy-2-methoxybenzoate (**8**) 26

Xanthone tetrafluorothianthrenium salt (**9-TFT**) 26

Hydroxyl xanthone (**9**) 27

(*R*)-4-(4-hydroxybenzyl)-3-propionyloxazolidin-2-one (**10**) 28

Hydroxy-boscalid (**11**) 29

Methyl 2,4-dimethylbenzoate tetrafluorothianthrenium salt (**12-TFT**) 29

Methyl 5-hydroxy-2,4-dimethylbenzoate (**12**) 30

2-Methoxy-benzonitrile thianthrenium salt (**13-TT**) 31

5-Hydroxy-2-methoxybenzonitrile (**13**) 32

3-Chloropropyl benzene tetrafluorothianthrenium salt (**14-TFT**) 32

4-(3-Chloropropyl)phenol (**14**) 33
4-(3-Hydroxy-1-phenylpropyl)phenol (**15**) 34
Hydroxy-salicin pentaacetate (**16**) 35
Methyl 1-phenylcyclopropane-1-carboxylate tetrafluorothianthrenium salt (**17-TFT**) 35
Methyl 1-(4-hydroxyphenyl)cyclopropane-1-carboxylate (**17**) 36
6-Methyl-4-chromanone thianthrenium salt (**18-TT**) 37
8-Hydroxy-6-methylchroman-4-one (**18**) 38
Benzyl benzoate tetrafluorothianthrenium salt (**19-TFT**) 38
4-Hydroxybenzyl benzoate (**19**) 39
N-benzyloxycarbonyl-4-*cis*-phenoxy-L-proline methyl ester tetrafluorothianthrenium salt (**20-TFT**) 40
Hydroxy-N-benzyloxycarbonyl-4-*cis*-phenoxy-L-proline methyl ester (**20**) 41
Hydroxy-etofenprox (**21**) 42
Biphenyl tetrafluorothianthrenium salt (**22-TFT**) 42
Biphenyl thianthrenium salt (**22-TT**) 43
4-Phenylphenol (**22**) 44
(3r,5r,7r)-1-(p-Tolyl)adamantane tetrafluorothianthrenium salt (**23-TFT**) 45
5-((3r,5r,7r)-Adamantan-1-yl)-2-methylphenol (**23**) 46
4'-Hydroxy-[1,1'-biphenyl]-4-yl trifluoromethanesulfonate (**24**) 46
Diclofenac amide thianthrenium salt (**25-TT**) 47
Hydroxy-diclofenac amide (**25**) 48
Hydroxy-pyriproxyfen (**26**) 49

General procedure and reaction condition optimization for etherification 49

General procedure of etherification 49
Table S2. Reaction condition optimization 50
Side product without Na_2CO_3: 4-ethylphenyl thiophene-2-carboxylate (**29-B**) 51
Side product biarylether (**29-C**) 51
Figure S3. Comparison of the etherifcation of TT and TFT salts 53

Thianthrenation and etherfication of arenes 53

2-Fluoro-6-phenoxybenzonitrile tetrafluorothianthrenium salt (**27-TFT**) 53
2-Fluoro-6-(4-methoxyphenoxy)benzonitrile (**27**) 54
1-Ethyl-4-phenoxybenzene (**28**) 55
tert-Butyl 3-(4-ethylphenoxy)azetidine-1-carboxylate (**29**) 56
trans-2-(4-Rthylphenoxy)cyclohexan-1-ol (**30**) 56
2-Fluoro-6-(4-(methoxy-d_3)phenoxy)benzonitrile (**31**) 57
1-Cyclobutoxy-4-ethylbenzene (**32**) 58
3-(4-Ethylphenoxy)oxetane (**33**) 59
2-(2,2,2-Trifluoroethoxy)-9H-xanthen-9-one (**34**) 60

Methoxy-PEG(4) ethylbenzene (**35**)........60
2-(4-Ethoxyphenoxy)-6-fluorobenzonitrile (**36**)........61
3-(4-Ethylphenoxy)butan-2-ol (**37**)........62
1-(*tert*-Butyl) 2-methyl (2*S*,4*R*)-4-(4-ethylphenoxy)pyrrolidine-1,2-dicarboxylate (**38**)........63
(3a*R*,5*R*,6*S*,6a*R*)-5-((*R*)-2,2-Dimethyl-1,3-dioxolan-4-yl)-6-(4-ethylphenoxy)-2,2-dimethyltetrahydro-furo[2,3-d][1,3]dioxole (**39**)........64
4-Methyl-*N*-phenylbenzenesulfonamide tetrafluorothianthrenium salt (**40-TFT**)........65
N-(4-methoxyphenyl)-4-methylbenzenesulfonamide (**40**)........65
tert-Butyl 3-(4-((benzoyloxy)methyl)phenoxy)azetidine-1-carboxylate (**41**)........66
tert-Butyl (*R*)-3-(4-((2-oxo-3-propionyloxazolidin-4-yl)methyl)phenoxy)azetidine-1-carboxylate (**42**) .67
4-Methoxy-flurbiprofen methyl ester (**43**).........68
tert-Butyl 3-(3-formyl-4-methoxyphenoxy)azetidine-1-carboxylate (**44**)........69
tert-Butyl 3-(5-((3r,5r,7r)-adamantan-1-yl)-2-methylphenoxy)azetidine-1-carboxylate (**45**)........69
tert-Butyl 3-(4-(3-chloropropyl)phenoxy)azetidine-1-carboxylate (**46**)........70
5-Oxetan-3-yloxy boscalid (**47**)........71
4-(Cyclohexylthio)-1,1'-biphenyl (**48**)........72
4-Fluoro-1,1'-biphenyl thianthrenium salt (**49-TT**)........72
Cyclohexyl(4'-fluoro-[1,1'-biphenyl]-4-yl)sulfane (**49**)........73
2-Methoxy-5-(phenylthio)benzonitrile (**50**)........74
Phenylthio-etofenprox (**51**)........75
Gram-scale synthesis of *tert*-butyl 3-(4-(3-chloropropyl)phenoxy)azetidine-1-carboxylate (**46**)........76
Figure S4. Gram-scale synthesis of **46**.........77

Determination of the stereochemistry of **20**........78

Figure S5. NOESY correlations of **20**........78

MECHANISTIC STUDIES........79

UV-Vis absorption spectra of the aryl thianthrenium salts........79

Figure S6. UV-Vis absorption spectra of biphenyl tetrafluorothianthrenium salt (**22-TFT**)........79
Figure S7. UV-Vis absorption spectra of ethylbenzene tetrafluorothianthrenium salt (**28-TFT**)........80

Emission spectrum of the blue LEDs........80

Figure S8. Emission spectrum of Kessil LEDs A160WE Tuna Blue........80

Stern-Volmer luminescence quenching studies........80

Figure S9. Emission spectrum for $Ir(dF(CF_3)ppy)_2(dtbpy)PF_6$ luminescence quenching by biphenyl tetrafluorothianthrenium salt **22-TFT** (left) and Stern-Volmer plot (right).........81
Figure S10. Emission spectrum for $Ir(dF(CF_3)ppy)_2(dtbpy)PF_6$ luminescence quenching by tetrafluorthianthrene TFT (left) and Stern-Volmer plot (right).........82
Figure S11. Emission spectra for $Ir(dF(CF_3)ppy)_2(dtbpy)PF_6$ luminescence quenching by

tetrafluorthianthrene TFT and biphenyl tetrafluoro-thianthrenium salt **22-TFT** (left) and quencher rate coefficient (right). 82

TEMPO trapping experiment 82

ArTEMPO product (**52**) 81

SPECTROSCOPIC DATA 84

^{1}H NMR of flurbiprofen methyl ester tetrafluorothianthrenium salt (**1-TFT**) 84

^{13}C NMR of flurbiprofen methyl ester tetrafluorothianthrenium salt (**1-TFT**) 85

^{19}F NMR of flurbiprofen methyl ester tetrafluorothianthrenium salt (**1-TFT**) 86

^{1}H NMR of flurbiprofen methylester-derived thianthrenium salt (**1-TT**) 87

^{13}C NMR of flurbiprofen methylester thianthrenium salt (**1-TT**) 88

^{19}F NMR of flurbiprofen methylester thianthrenium salt (**1-TT**) 89

^{1}H NMR of hydroxy-flurbiprofen methyl ester (**1**) 90

^{13}C NMR of hydroxy-flurbiprofen methyl ester (**1**) 91

^{19}F NMR of hydroxy-flurbiprofen methyl ester (**1**) 92

^{1}H NMR of 4-chlorophenol (**2**) 93

^{13}C NMR of 4-chlorophenol (**2**) 94

^{1}H NMR of 2-methoxybenzaldehyde tetrafluorothianthrenium salt (**3-TFT**) 95

^{13}C NMR of 2-methoxybenzaldehyde tetrafluorothianthrenium salt (**3-TFT**) 96

^{19}F NMR of 2-methoxybenzaldehyde tetrafluorothianthrenium salt (**3-TFT**) 97

^{1}H NMR of 5-hydroxy-2-methoxybenzaldehyde (**3**) 98

^{13}C NMR of 5-hydroxy-2-methoxybenzaldehyde (**3**) 99

^{1}H NMR of 2,2,2-trifluoro-N-phenylacetamide tetrafluorothianthrenium salt (**4-TFT**) 100

^{13}C NMR of 2,2,2-trifluoro-N-phenylacetamide tetrafluorothianthrenium salt (**4-TFT**) 101

^{19}F NMR of 2,2,2-trifluoro-N-phenylacetamide tetrafluorothianthrenium salt (**4-TFT**) 102

^{1}H NMR of 2,2,2-trifluoro-N-(4-hydroxyphenyl)acetamide (**4**) 103

^{13}C NMR of 2,2,2-trifluoro-N-(4-hydroxyphenyl)acetamide (**4**) 104

^{19}F NMR of 2,2,2-trifluoro-N-(4-hydroxyphenyl)acetamide (**4**) 105

^{1}H NMR of methyl acetyl-L-phenylalate tetrafluorothianthrenium salt (**5-TFT**) 106

^{13}C NMR of methyl acetyl-L-phenylalate tetrafluorothianthrenium salt (**5-TFT**) 107

^{19}F NMR of methyl acetyl-L-phenylalate tetrafluorothianthrenium salt (**5-TFT**) 108

^{1}H NMR of methyl acetyl-L-tyrosinate (**5**) 109

^{13}C NMR of methyl acetyl-L-tyrosinate (**5**) 110

^{1}H NMR of hydroxy-nefiracetam (**6**) 111

^{13}C NMR of hydroxy-nefiracetam (**6**) 112

^{1}H NMR of bromobenzene tetrafluorothianthrenium salt (**7-TFT**) 113

^{13}C NMR of bromobenzene tetrafluorothianthrenium salt (**7-TFT**) 114

^{19}F NMR of bromobenzene tetrafluorothianthrenium salt (**7-TFT**) 115

^{1}H NMR of 4-bromophenol (**7**) 116

^{13}C NMR of 4-bromophenol (**7**) 117

^{1}H NMR of methyl 2-methoxybenzoate tetrafluorothianthrenium salt (**8-TFT**) 118

^{13}C NMR of methyl 2-methoxybenzoate tetrafluorothianthrenium salt (**8-TFT**) 119

^{19}F NMR of methyl 2-methoxybenzoate tetrafluorothianthrenium salt (**8-TFT**) 120

^{1}H NMR of methyl 5-hydroxy-2-methoxybenzoate (**8**) 121

^{13}C NMR of methyl 5-hydroxy-2-methoxybenzoate (**8**) 122

^{1}H NMR of xanthone tetrafluorothianthrenium salt (**9-TFT**) 123

^{13}C NMR of xanthone tetrafluorothianthrenium salt (**9-TFT**) 124

^{19}F NMR of xanthone tetrafluorothianthrenium salt (**9-TFT**) 125

^{1}H NMR of hydroxyl xanthone (**9**) 126

^{13}C NMR of hydroxyl xanthone (**9**) 127

^{1}H NMR of (*R*)-4-(4-hydroxybenzyl)-3-propionyloxazolidin-2-one (**10**) 128

^{13}C NMR of (*R*)-4-(4-hydroxybenzyl)-3-propionyloxazolidin-2-one (**10**) 129

^{1}H NMR of hydroxy-boscalid (**11**) 130

^{13}C NMR of hydroxy-boscalid (**11**) 131

^{1}H NMR of methyl 2,4-dimethylbenzoate tetrafluorothianthrenium salt (**12-TFT**) 132

^{13}C NMR of methyl 2,4-dimethylbenzoate tetrafluorothianthrenium salt (**12-TFT**) 133

^{19}F NMR of methyl 2,4-dimethylbenzoate tetrafluorothianthrenium salt (**12-TFT**) 134

^{1}H NMR of methyl 5-hydroxy-2,4-dimethylbenzoate (**12**) 135

^{13}C NMR of methyl 5-hydroxy-2,4-dimethylbenzoate (**12**) 136

^{1}H NMR of 2-methoxy-5-methylbenzonitrile thianthrenium salt (**13-TT**) 137

^{13}C NMR of 2-methoxy-5-methylbenzonitrile thianthrenium salt (**13-TT**) 138

^{19}F NMR of 2-methoxy-5-methylbenzonitrile thianthrenium salt (**13-TT**) 139

^{1}H NMR of 5-hydroxy-2-methoxybenzonitrile (**13**) 140

^{13}C NMR of 5-hydroxy-2-methoxybenzonitrile (**13**) 141

^{1}H NMR of 3-chloropropyl benzene tetrafluorothianthrenium salt (**14-TFT**) 142

^{13}C NMR of 3-chloropropyl benzene tetrafluorothianthrenium salt (**14-TFT**) 143

^{19}F NMR of 3-chloropropyl benzene tetrafluorothianthrenium salt (**14-TFT**) 144

^{1}H NMR of 4-(3-chloropropyl)phenol (**14**) 145

^{13}C NMR of 4-(3-chloropropyl)phenol (**14**) 146

^{1}H NMR of 3-(3-hydroxy-1-phenylpropyl)phenol (**15**) 147

^{13}C NMR of 3-(3-hydroxy-1-phenylpropyl)phenol (**15**) 148

^{1}H NMR of hydroxy salicin pentaacetate (**16**) 149

^{13}H NMR of hydroxy salicin pentaacetate (**16**) 150

^{1}H NMR of methyl 1-phenylcyclopropane-1-carboxylate tetrafluorothianthrenium salt (**17-TFT**) 151

^{13}C NMR of methyl 1-phenylcyclopropane-1-carboxylate tetrafluorothianthrenium salt (**17-TFT**) 152

^{19}F NMR of methyl 1-phenylcyclopropane-1-carboxylate tetrafluorothianthrenium salt (**17-TFT**) 153

^{1}H NMR of methyl 1-(4-hydroxyphenyl)cyclopropane-1-carboxylate (**17**) 154

^{13}C NMR of methyl 1-(4-hydroxyphenyl)cyclopropane-1-carboxylate (**17**) 155

^{1}H NMR of 6-methyl-4-chromanone thianthrenium salt (**18-TT**) 156

^{13}C NMR of 6-methyl-4-chromanone thianthrenium salt (**18-TT**) 157

^{19}F NMR of 6-methyl-4-chromanone thianthrenium salt (**18-TT**) 158

^{1}H NMR of 8-hydroxy-6-methylchroman-4-one (**18**) 159

^{13}C NMR of 8-hydroxy-6-methylchroman-4-one (**18**) 160

^{1}H NMR of benzyl benzoate tetrafluorothianthrenium salt (**19-TFT**) 161

^{13}C NMR of benzyl benzoate tetrafluorothianthrenium salt (**19-TFT**) 162

^{19}F NMR of benzyl benzoate tetrafluorothianthrenium salt (**19-TFT**) 163

^{1}H NMR of 4-hydroxybenzyl benzoate (**19**) 164

^{13}C NMR of 4-hydroxybenzyl benzoate (**19**) 165

^{1}H NMR of N-benzyloxycarbonyl-4-*cis*-phenoxy-L-proline methyl ester tetrafluorothianthrenium salt (**20-TFT**) 166

^{13}C NMR of N-benzyloxycarbonyl-4-*cis*-phenoxy-L-proline methyl ester tetrafluorothianthrenium salt (**20-TFT**) 167

^{19}F NMR of N-benzyloxycarbonyl-4-*cis*-phenoxy-L-proline methyl ester tetrafluorothianthrenium salt (**20-TFT**) 168

^{1}H NMR of hydroxy-N-benzyloxycarbonyl-4-*cis*-phenoxy-L-proline methyl ester (**20**) 169

^{13}C NMR of hydroxy-N-benzyloxycarbonyl-4-*cis*-phenoxy-L-proline methyl ester (**20**) 170

^{1}H-^{13}C HSQC NMR of hydroxy-N-benzyloxycarbonyl-4-*cis*-phenoxy-L-proline methyl ester (**20**) 170

NOESY NMR of hydroxy-N-benzyloxycarbonyl-4-*cis*-phenoxy-L-proline methyl ester (**20**) 172

^{1}H NMR of hydroxy-etofenprox (**21**) 173

^{13}C NMR of hydroxy-etofenprox (**21**) 174

^{1}H NMR of biphenyl tetrafluorothianthrenium salt (**22-TFT**) 175

^{13}C NMR of biphenyl tetrafluorothianthrenium salt (**22-TFT**) 176

^{19}F NMR of biphenyl tetrafluorothianthrenium salt (**22-TFT**) 177

^{1}H NMR of biphenyl thianthrenium salt (**22-TT**) 178

^{13}C NMR of biphenyl thianthrenium salt (**22-TT**) 179

^{19}F NMR of biphenyl thianthrenium salt (**22-TT**) 180

^{1}H NMR of 4-phenylphenol (**22**) 181

^{13}C NMR of 4-phenylphenol (**22**) 182

^{1}H NMR of (3r,5r,7r)-1-(*p*-tolyl)adamantane tetrafluorothianthrenium salt (**23-TFT**) 183

^{13}C NMR of (3r,5r,7r)-1-(*p*-tolyl)adamantane tetrafluorothianthrenium salt (**23-TFT**) 184

^{19}F NMR of (3r,5r,7r)-1-(*p*-tolyl)adamantane tetrafluorothianthrenium salt (**23-TFT**) 185

^{1}H NMR of 5-((3r,5r,7r)-adamantan-1-yl)-2-methylphenol (**23**) 186

^{13}C NMR of 5-((3r,5r,7r)-adamantan-1-yl)-2-methylphenol (**23**) 187

^{1}H NMR of 4'-hydroxy-[1,1'-biphenyl]-4-yl trifluoromethanesulfonate (**24**) 188

^{13}C NMR of 4'-hydroxy-[1,1'-biphenyl]-4-yl trifluoromethanesulfonate (**24**) 189

^{19}F NMR of 4'-hydroxy-[1,1'-biphenyl]-4-yl trifluoromethanesulfonate (**24**) 190

^{1}H NMR of diclofenac amide thianthrenium salt (**25-TT**) 191

^{13}C NMR of diclofenac amide thianthrenium salt (**25-TT**) 192

^{19}F NMR of diclofenac amide thianthrenium salt (**25-TT**) 193

^{1}H NMR of hydroxy diclofenac amide (**25**) 194

^{13}C NMR of hydroxy diclofenac amide (**25**) 195

^{1}H NMR of hydroxy-pyriproxyfen (**26**) 196

^{13}C NMR of hydroxy-pyriproxyfen (**26**) 197

^{1}H NMR of 2-fluoro-6-phenoxybenzonitrile tetrafluorothianthrenium salt (**27-TFT**) 198

^{13}C NMR of 2-fluoro-6-phenoxybenzonitrile tetrafluorothianthrenium salt (**27-TFT**) 199

^{19}F NMR of 2-fluoro-6-phenoxybenzonitrile tetrafluorothianthrenium salt (**27-TFT**) 200

^{1}H NMR of 2-fluoro-6-(4-methoxyphenoxy)benzonitrile (**27**) 201

^{13}C NMR of 2-fluoro-6-(4-methoxyphenoxy)benzonitrile (**27**) 202

^{19}F NMR of 2-fluoro-6-(4-methoxyphenoxy)benzonitrile (**27**) 203

^{1}H NMR of 1-ethyl-4-phenoxybenzene (**28**) 204

^{13}C NMR of 1-ethyl-4-phenoxybenzene (**28**) 205

^{1}H NMR of *tert*-butyl 3-(4-ethylphenoxy)azetidine-1-carboxylate (**29**) 206

^{13}C NMR of *tert*-butyl 3-(4-ethylphenoxy)azetidine-1-carboxylate (**29**) 207

^{1}H NMR of 4-ethylphenyl thiophene-2-carboxylate (**29-B**) 208

^{13}C NMR of 4-ethylphenyl thiophene-2-carboxylate (**29-B**) 209

^{1}H NMR of 4,4'-oxybis(ethylbenzene) (**29-C**) 209

^{13}C NMR of 4,4'-oxybis(ethylbenzene) (**29-C**) 211

^{1}H NMR of *trans*-2-(4-ethylphenoxy)cyclohexan-1-ol (**30**) 212

^{13}C NMR of *trans*-2-(4-ethylphenoxy)cyclohexan-1-ol (**30**) 213

^{1}H NMR of 2-fluoro-6-(4-(methoxy-d_3)phenoxy)benzonitrile (**31**) 214

^{13}C NMR of 2-fluoro-6-(4-(methoxy-d_3)phenoxy)benzonitrile (**31**) 215

^{19}F NMR of 2-fluoro-6-(4-(methoxy-d_3)phenoxy)benzonitrile (**31**) 216

^{1}H NMR of 1-cyclobutoxy-4-ethylbenzene (**32**) 217

^{13}C NMR of 1-cyclobutoxy-4-ethylbenzene (**32**) 218

^{1}H NMR of 3-(4-ethylphenoxy)oxetane (**33**) 219

^{13}C NMR of 3-(4-ethylphenoxy)oxetane (**33**) 220

^{1}H NMR of 2-(2,2,2-trifluoroethoxy)-9H-xanthen-9-one (**34**) 221

^{13}C NMR of 2-(2,2,2-trifluoroethoxy)-9H-xanthen-9-one (**34**) 222

^{19}F NMR of 2-(2,2,2-trifluoroethoxy)-9H-xanthen-9-one (**34**) 223

^{1}H NMR of methoxy-PEG(4) ethylbenzene (**35**) 224

^{13}C NMR of methoxy-PEG(4) ethylbenzene (**35**) 225

^{1}H NMR of 2-(4-ethoxyphenoxy)-6-fluorobenzonitrile (**36**) 226

^{13}C NMR of 2-(4-ethoxyphenoxy)-6-fluorobenzonitrile (**36**) 227

^{19}F NMR of 2-(4-ethoxyphenoxy)-6-fluorobenzonitrile (**36**) 228

^{1}H NMR of *erythro*-3-(4-ethylphenoxy)butan-2-ol (**37a**) 229

^{13}C NMR of *erythro*-3-(4-ethylphenoxy)butan-2-ol (**37a**) 230

^{1}H NMR of *threo*-3-(4-ethylphenoxy)butan-2-ol (**37b**) 231

^{13}C NMR of *threo*-3-(4-ethylphenoxy)butan-2-ol (**37b**) 232

^{1}H NMR of 1-(*tert*-butyl) 2-methyl (2*S*,4*R*)-4-(4-ethylphenoxy)pyrrolidine-1,2-dicarboxylate (**38**) 233

^{13}C NMR of 1-(*tert*-butyl) 2-methyl (2*S*,4*R*)-4-(4-ethylphenoxy)pyrrolidine-1,2-dicarboxylate (**38**) 234

^{1}H NMR of (3a*R*,5*R*,6*S*,6a*R*)-5-((*R*)-2,2-dimethyl-1,3-dioxolan-4-yl)-6-(4-ethylphenoxy)-2,2-dimethyltetrahydro-furo[2,3-d][1,3]dioxole (**39**) 235

^{13}C NMR of (3a*R*,5*R*,6*S*,6a*R*)-5-((*R*)-2,2-dimethyl-1,3-dioxolan-4-yl)-6-(4-ethylphenoxy)-2,2-dimethyltetrahydro-furo[2,3-d][1,3]dioxole (**39**) 236

^{1}H NMR of 4-methyl-*N*-phenylbenzenesulfonamide tetrafluorothianthrenium salt (**40-TFT**) 237

^{13}C NMR of 4-methyl-*N*-phenylbenzenesulfonamide tetrafluorothianthrenium salt (**40-TFT**) 238

^{19}F NMR of 4-methyl-*N*-phenylbenzenesulfonamide tetrafluorothianthrenium salt (**40-TFT**) 239

^{1}H NMR of N-(4-methoxyphenyl)-4-methylbenzenesulfonamide (**40**) 240

^{13}C NMR of N-(4-methoxyphenyl)-4-methylbenzenesulfonamide (**40**) 241

^{1}H NMR of *tert*-butyl 3-(4-((benzoyloxy)methyl)phenoxy)azetidine-1-carboxylate (**41**) 242

^{13}C NMR of *tert*-butyl 3-(4-((benzoyloxy)methyl)phenoxy)azetidine-1-carboxylate (**41**) 243

^{1}H NMR of *tert*-butyl (*R*)-3-(4-((2-oxo-3-propionyloxazolidin-4-yl)methyl)phenoxy)azetidine-1-carboxylate (**42**) 244

^{13}C NMR of *tert*-butyl (*R*)-3-(4-((2-oxo-3-propionyloxazolidin-4-yl)methyl)phenoxy)azetidine-1-carboxylate (**42**) 245

^{1}H NMR of 4-methoxy-flurbiprofen methyl ester (**43**) 246

^{13}C NMR of 4-methoxy-flurbiprofen methyl ester (**43**)....247

^{19}F NMR of 4-methoxy-flurbiprofen methyl ester (**43**)....248

^{1}H NMR of *tert*-butyl 3-(3-formyl-4-methoxyphenoxy)azetidine-1-carboxylate (**44**)....249

^{13}C NMR of *tert*-butyl 3-(3-formyl-4-methoxyphenoxy)azetidine-1-carboxylate (**44**)....250

^{1}H NMR of *tert*-butyl 3-(5-((3r,5r,7r)-adamantan-1-yl)-2-methylphenoxy)azetidine-1-carboxylate (**45**)....251

^{13}C NMR of *tert*-butyl 3-(5-((3r,5r,7r)-adamantan-1-yl)-2-methylphenoxy)azetidine-1-carboxylate (**45**)...252

^{1}H NMR of *tert*-butyl 3-(4-(3-chloropropyl)phenoxy)azetidine-1-carboxylate (**46**)....253

^{13}C NMR of *tert*-butyl 3-(4-(3-chloropropyl)phenoxy)azetidine-1-carboxylate (**46**)....254

^{1}H NMR of 5-oxetan-3-yloxy boscalid (**47**)....255

^{13}C NMR of 5-oxetan-3-yloxy boscalid (**47**)....256

^{1}H NMR of 4-(cyclohexylthio)-1,1'-biphenyl (**48**)....257

^{13}C NMR of 4-(cyclohexylthio)-1,1'-biphenyl (**48**)....258

^{1}H NMR of 4-fluoro-1,1'-biphenyl (**49-TT**)....259

^{13}C NMR of 4-fluoro-1,1'-biphenyl (**49-TT**)....260

^{19}F NMR of 4-fluoro-1,1'-biphenyl (**49-TT**)....261

^{1}H NMR of cyclohexyl(4'-fluoro-[1,1'-biphenyl]-4-yl)sulfane (**49**)....262

^{13}C NMR of cyclohexyl(4'-fluoro-[1,1'-biphenyl]-4-yl)sulfane (**49**)....263

^{19}F NMR of cyclohexyl(4'-fluoro-[1,1'-biphenyl]-4-yl)sulfane (**49**)....264

^{1}H NMR of 2-methoxy-5-(phenylthio)benzonitrile (**50**)....265

^{13}C NMR of 2-methoxy-5-(phenylthio)benzonitrile (**50**)....266

^{1}H NMR of phenylthio-etofenprox (**51**)....267

^{13}C NMR of phenylthio-etofenprox (**51**)....268

^{1}H NMR of ArTEMPO product (**52**)....269

^{13}C NMR of ArTEMPO product (**52**)....270

REFERENCES....271

MATERIALS AND METHODS

All air- and moisture-insensitive reactions were carried out under an ambient atmosphere and monitored by thin-layer chromatography (TLC). High-resolution mass spectra were obtained using *Q Exactive Plus* from *Thermo*. Concentration under reduced pressure was performed by rotary evaporation at 25–40 °C at an appropriate pressure. Purified compounds were further dried under vacuum (10^{-6} – 10^{-3} bar). Yields refer to purified and spectroscopically pure compounds, unless otherwise stated. A LED Kessil® A160WE was used as the light source.

Solvents

Acetonitrile *was* purchased from *Sigma-Aldrich* and used as received. Anhydrous solvents were obtained from Phoenix Solvent Drying Systems. All deuterated solvents were purchased from Euriso-Top®.

Chromatography

Thin layer chromatography (TLC) was performed using EMD TLC silica gel 60 F_{254} plates pre-coated with 250 µm thickness silica gel 60 F_{254} and visualized by fluorescence quenching under UV light. Flash column chromatography was performed using silica gel (40–63 µm particle size) purchased from Geduran®. Preparatory high-performance liquid chromatographic separation was executed on a Shimadzu Prominence Preparative HPLC system with an YMC-Triart C18 HPLC column.

Spectroscopy and Instruments

NMR spectra were recorded on a Bruker *Ascend*™ 500 spectrometer operating at 500 MHz, 471 MHz, and 126 MHz, for ^{1}H, ^{19}F, and ^{13}C acquisitions, respectively. Chemical shifts are reported in ppm with the solvent residual peak as the internal standard. For ^{1}H NMR: $CDCl_3$, δ 7.26; CD_2Cl_2, δ 5.32; CD_3CN, δ 1.94; $(CD_3)_2SO$, δ 2.50; CD_3OD, δ 3.31; $(CD_3)_2CO$, δ 2.05. For ^{13}C NMR: $CDCl_3$, δ 77.16; CD_2Cl_2, δ 53.84; CD_3CN, δ 1.32, 118.26; $(CD_3)_2SO$, δ 39.52; CD_3OD, δ 49.00; $(CD_3)_2CO$, δ 29.84. ^{19}F NMR spectra were referenced using a unified chemical shift scale based on the ^{1}H resonance of tetramethylsilane (1% (v/v) solution in the respective solvent). Data is reported as follows: s = singlet, d = doublet, t = triplet, q = quartet, m = multiplet, br = broad; coupling constants in Hz; integration.

Starting materials

All substrates and materials were used as received from commercial suppliers, unless otherwise stated. Compounds **2-TFT**, **6-TFT**, **10-TFT**, **11-TFT**; **15-TFT**, **16-TFT**, **21-TT**, **24-TFT**, **26-TFT**, **28-TFT**, thianthrene-*S*-oxide (TTO) and tetrafluorothianthrene-*S*-oxide (TFTO) were prepared according to the litterature.[1]

EXPERIMENTAL DATA

General procedure and reaction condition optimization for hydroxylation

General procedure of hydroxylation with Cu_2O

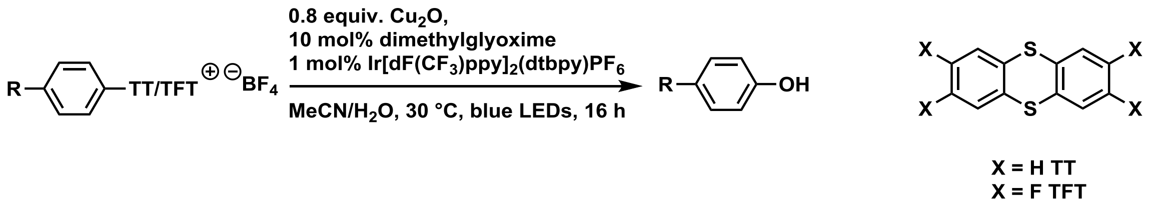

To a 4-mL borosilicate vial, equipped with a magnetic stir bar was added copper(I) oxide (11.5 mg, 80.0 µmol , 0.800 equiv.), dimethylglyoxime (1.2 mg, 10 µmol, 0.10 equiv.), and MeCN/H_2O (0.9 mL, v/v= 2:1). After stirring for 10 mins at ambient temperature, (tetrafluoro)thianthrenium salt (0.100 mmol, 1.00 equiv.), and Ir[dF(CF_3)ppy]$_2$(dtbpy)PF_6 (1.1 mg, 1.0 µmol, 1.0 mol%) in MeCN (0.40 mL, *c*= 0.25 M) were then added. The vial was evacuated and then filled with argon; this procedure was repeated three times. The vial was placed in 2 cm distance to two 34 W blue LEDs. The temperature was kept at approximately 30 °C with the use of a cooling fan. The reaction mixture was stirred for 16 hours under blue LED irradiation, and then diluted with CH_2Cl_2 (2 mL). The reaction mixture was filtered through a short pad of Celite using CH_2Cl_2 (20 mL) as eluent. The filtrate was collected and concentrated *in vacuo,* and the residue was then purified by flash column chromatography on silica gel, eluting with ethyl acetate/hexanes to afford the hydroxylated product.

Note: The amount of dimethylglyoxime is curial. Control experiments showed that yields were lower if different amounts of dimethylglyoxime were used.

General procedure of hydroxylation with CuTC

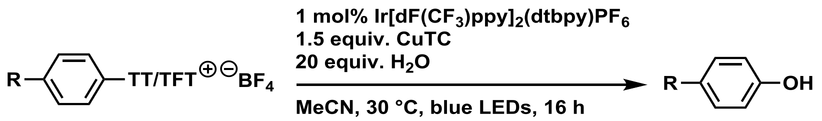

To a 4-mL borosilicate vial, equipped with a magnetic stir bar was added Ir[dF(CF_3)ppy]$_2$(dtbpy)PF_6 (2.2 mg, 2.0 µmol, 1.0 mol%), copper(I) thiophene-2-carboxylate (57.2 mg, 0.300 mmol, 1.50 equiv.), and (tetrafluoro)thianthrenium salt (0.200 mmol, 1.00 equiv.) at 25 °C. The vial was evacuated and then filled with argon; this procedure was repeated three times. MeCN (1 mL, *c* = 0.2 M) was added, followed by H_2O (72.1 mg, 721 µL, 4.00 mmol, 20.0 equiv.). The vial was placed in 2 cm distance to two 34 W blue LEDs. The temperature was kept at approximately 30 °C with the use of a cooling fan. The reaction mixture was stirred for 16 hours under blue LED irradiation, and then diluted with ethyl acetate (1 mL). The reaction mixture was filtered through a short pad of silica using ethyl acetate (20 mL) as eluent. The filtrate was collected and concentrated *in vacuo,* and the residue was then purified by flash column chromatography on silica gel, eluting with ethyl acetate/hexanes to afford the hydroxylated product.

Table S1. Reaction condition optimization

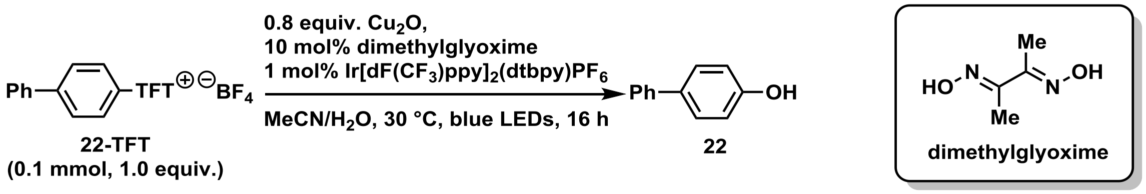

Change of reaction conditions	Yield of **22**[b]
none[a]	83%
no water	< 1%
no Cu_2O	< 1%
no $Ir[dF(CF_3)ppy]_2(dtbpy)PF_6$	< 1%
no dimethylglyoxime	11%
50 mol% dimethylglyoxime	38%
CuI instead of Cu_2O[c]	< 5%
$Cu(MeCN)_4BF_4$ instead of Cu_2O[d]	9%
$Ru(bipy)_3(PF_6)_2$ instead of $Ir[dF(CF_3)ppy]_2(dtbpy)PF_6$[e]	24%
Organic dye 4CzIPN instead of $[Ir[dF(CF_3)ppy]_2(dtbpy)PF_6$[f]	20%
Reaction is carried out without purification of the aryl thianthrenium salts[g]	24%

[a]Thianthrenium salt (1.0 equiv.), $[Ir[dF(CF_3)ppy]_2(dtbpy)PF_6]$ (1 mol%), dimethylglyoxime (0.1 equiv.), Cu_2O (0.8 equiv.), H_2O (0.30 mL.), acetonitrile (1.0 mL), blue LED (34W), 30 °C, 16 hours. [b]Yield based on NMR with 0.1 mmol styrol as internal standard. [c]CuI (1.0 equiv). [d]$Cu(MeCN)_4BF_4$ (1.0 equiv). [e]$Ru(bipy)_3(PF_6)_2$ (2.5 mmol%). [f]Organic dye 4CzIPN (10 mmol%). [g]isolated yield over two steps.

Figure S1. Comparison of the hydroxylation of TT and TFT salts

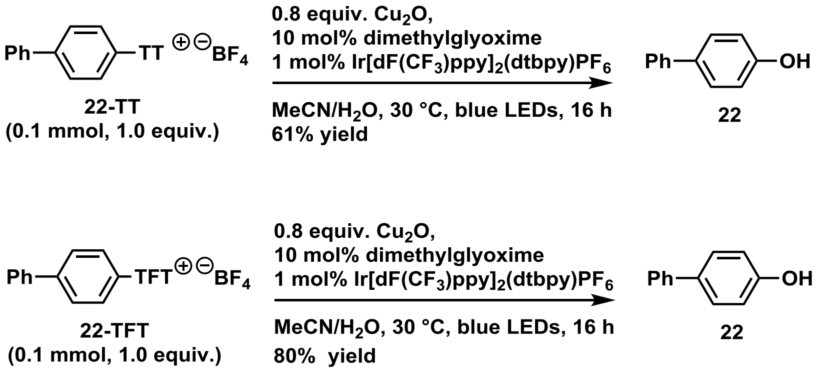

Figure S2. Comparison of the hydroxylation with Cu_2O and CuTC

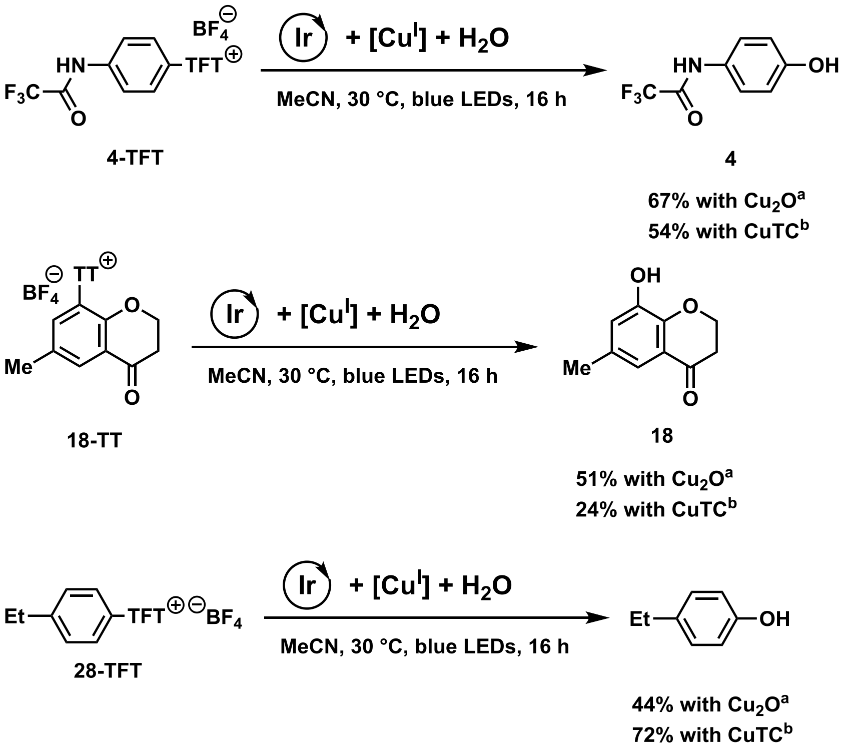

[a]Reaction conditions: thianthrenium salt (1.0 equiv.), $[Ir[dF(CF_3)ppy]_2(dtbpy)PF_6]$ (1 mol%), dimethylglyoxime (0.1 equiv.), Cu_2O (0.8 equiv.), H_2O (160 equiv.), acetonitrile, blue LED (34W), 30 °C, 16 hours. [b]Reaction conditions: thianthrenium salt (1.0 equiv.), $[Ir[dF(CF_3)ppy]_2(dtbpy)PF_6]$ (1 mol%), CuTC (1.5 equiv.), H_2O (20 equiv.), acetonitrile, blue LED (34W), 30 °C, 16 hours.

Thianthrenation and hydroxylation of arenes

Flurbiprofen methyl ester tetrafluorothianthrenium salt (1-TFT)

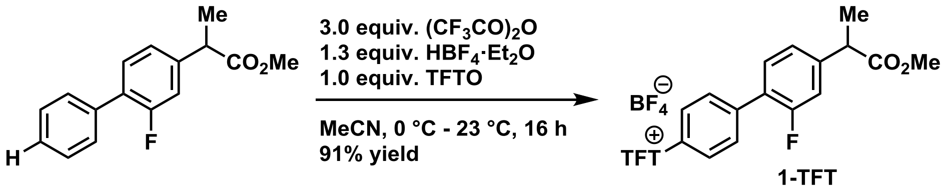

Under an ambient atmosphere, a 20-mL glass vial was charged with flurbiprofen methyl ester (395 mg, 1.30 mmol, 1.00 equiv) and MeCN (10 mL, *c* = 0.13 M). Trifluoroacetic anhydride (0.54 mL, 0.82 g, 3.9 mmol, 3.0 equiv.) was added while stirring the reaction mixture at 23°C. After cooling to 0°C, tetrafluorothianthrene-*S*-oxide (395 mg, 1.30 mmol, 1.00 equiv.) was added in one portion, followed by the dropwise addition of $HBF_4{\cdot}OEt_2$ (230 μL, 274 mg, 1.69 mmol, 1.30 equiv.). The mixture was stirred at 0°C for 2 hours, then at 23°C for 14 hours. The solution was diluted with CH_2Cl_2 (5 mL) and poured onto a mixture of CH_2Cl_2 (20 mL) and saturated aqueous $NaHCO_3$ solution (30 mL). After stirring for 5 min, the mixture was poured into a separating funnel, and the aqueous phase was extracted with DCM (2 × 20 mL). The DCM layer was washed with aqueous $NaBF_4$ solution (10% w/w, 2 × ca. 20 mL). The DCM layer was dried over Na_2SO_4, filtered, and the solvent was removed under reduced pressure. The residue was purified by chromatography on silica gel eluting with DCM/MeOH (30:1 (v/v)), then the solvent was removed *in vacuo* to afford **1-TFT** (744 mg, 91%) as a colorless powder.

R*f* = 0.35 (MeOH/DCM, 1/15, v/v).

NMR Spectroscopy:

^{1}H NMR (300 MHz, CD_2Cl_2, 25 °C, δ): 8.54 (dd, *J* = 8.6, 7.0 Hz, 2H), 7.82 (dd, *J* = 9.3, 6.8 Hz, 2H), 7.71 – 7.62 (m, 2H), 7.45 – 7.26 (m, 3H), 7.22 – 7.02 (m, 2H), 3.77 (q, *J* = 7.2 Hz, 1H), 3.65 (s, 3H), 1.49 (d, *J* = 7.2 Hz, 3H).

^{13}C {^{1}H}NMR (75 MHz, CD_2Cl_2, 25 °C, δ): 174.6, 156.0 (d, *J* = 249.4 Hz), 154.7 (dd, *J* = 241.1, 13.2 Hz), 151.2 (dd, *J* = 235.0, 13.2 Hz), 144.8 (d, *J* = 7.9 Hz), 141.6 (d, *J* = 1.4 Hz), 134.8 (dd, *J* = 8.1, 4.1 Hz), 131.6 (d, *J* = 3.5 Hz), 131.1 (d, *J* = 3.2 Hz), 128.8, 125.3 (dd, *J* = 21.8, 2.5 Hz), 124.8 (d, *J* = 3.3 Hz), 124.1 (d, *J* = 12.8 Hz), 121.7, 120.5 (d, *J* = 21.4 Hz), 116.0 (d, *J* = 23.3 Hz), 114.9 (dd, *J* = 6.8, 3.6 Hz), 52.6, 45.4 (d, *J* = 1.5 Hz), 18.6.

^{19}F NMR (282 MHz, CD_2Cl_2, 25 °C, δ): –117.8, –122.6 (d, *J* = 20.7Hz), –130.7 (dt, *J* = 20.6, 8.1 Hz), –149.2 (brs), –149.3 (brs).

HRMS-FIA(m/z) calc'd for $C_{28}H_{18}O_2S_2F_5^+$ $[M\text{-}BF_4]^+$, 545.0663; found, 545.0660 deviation: –0.6 ppm.

Flurbiprofen methylester thianthrenium salt (1-TT)

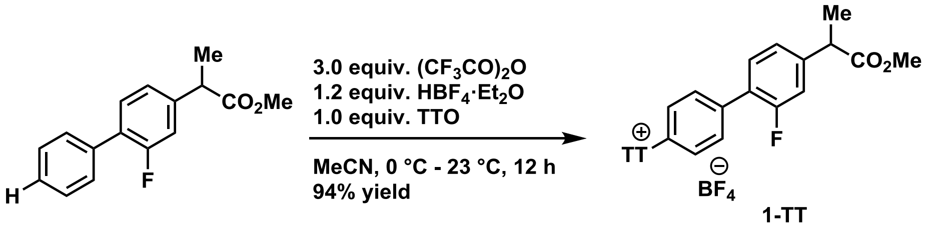

Under an ambient atmosphere, a 20-mL glass vial was charged with flurbiprofen methyl ester (1.29 g, 5.00 mmol, 1.00 equiv) and MeCN (5.0 mL, *c* = 1.0 M). After cooling to 0°C, $HBF_4{\cdot}OEt_2$ (0.82 mL, 0.97 g, 6.0 mmol, 1.2 equiv) and thianthrene-*S*-oxide (1.16 g, 5.00 mmol, 1.00 equiv) was added to the vial while stirring the mixture, leading to a suspension. Subsequently, trifluoroacetic anhydride (2.1 mL, 3.2 g, 15 mmol, 3.0 equiv) was added in one portion at 0°C, resulting in a color change to deep purple. Subsequently, the reaction mixture was allowed to reach 23°C and stirred for 12 hours. The solution was diluted with DCM (5 mL) and poured onto a mixture of DCM (30 mL) and saturated aqueous $NaHCO_3$ solution (20 mL). After stirring for 5 min at 23°C, the mixture was poured into a separating funnel, and the layers were separated. The DCM layer was washed with aqueous $NaBF_4$ solution (10% w/w, 4 × ca. 20 mL). The DCM layer was dried over Na_2SO_4, filtered, and the solvent was removed under reduced pressure. The residue was purified by chromatography on silica gel eluting with DCM/MeOH (30:1 (v/v)), then the solvent was removed in vacuo to afford **1-TT** (2.63 g, 94%) as a colorless powder.

R*f* = 0.35 (MeOH/DCM, 1/15, v/v).

NMR Spectroscopy:

^{1}H NMR (500 MHz, DMSO-d$_6$, 25 C, δ): 8.62 (dd, *J* = 7.9, 1.4 Hz, 2H), 8.09 (d, *J* = 7.1 Hz, 2H), 7.94 (td, *J* = 7.7, 1.5 Hz, 2H), 7.88 (td, *J* = 7.7, 1.4 Hz, 2H), 7.72 (dd, *J* = 8.7, 1.6 Hz, 2H), 7.47 (t, *J* = 8.2 Hz, 1H), 7.35 – 7.30 (m, 2H), 7.30 – 7.19 (m, 2H), 3.90 (q, *J* = 7.1 Hz, 1H), 3.60 (s, 3H),

^{13}C {^{1}H} NMR (126 MHz, DMSO-d$_6$, 25 °C, δ): 173.6, 158.8 (d, *J* = 247.4 Hz), 144.0 (d, *J* = 7.9 Hz), 139.0, 135.7, 135.4, 134.8, 130.9 (d, *J* = 3.1 Hz), 130.6 (d, *J* = 3.0 Hz), 130.3, 129.6, 128.4, 124.6 (d, *J* = 12.8 Hz), 124.3, 124.2 (d, *J* = 16.0 Hz), 119.1, 115.4 (d, *J* = 23.0 Hz), 52.0, 43.8, 18.3.

^{19}F NMR (471 MHz, DMSO-d$_6$, 25 C, δ): –117.8 (t, *J* = 10.0 Hz), –148.2 (brs), –148.3 (brs).

HRMS-ESI(m/z) calc'd for $C_{28}H_{22}FO_2S_2^+$ [M-BF$_4$]$^+$, 473.1040; found, 473.1044; deviation: –0.9 ppm.

Hydroxy-flurbiprofen methyl ester (1)

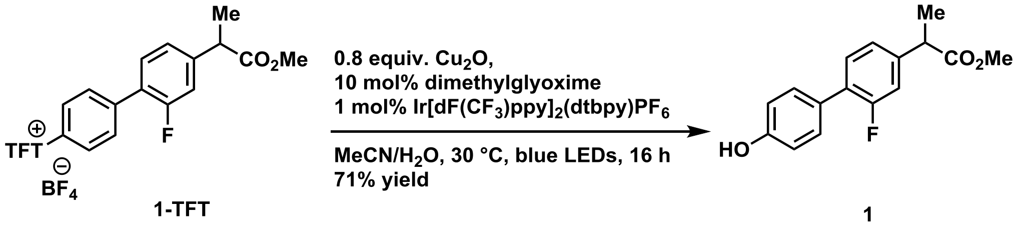

To a 20-mL glass vial, equipped with a magnetic stir bar was added copper(I) oxide (134 mg, 0.936 mmol, 0.800 equiv.), dimethylglyoxime (13.5 mg, 117 µmmol, 0.100 equiv.), and MeCN/H_2O (9.1 mL, v/v = 10:3). After stirring for 10 mins at ambient temperature, flurbiprofen methyl ester tetrafluorothianthrenium salt **1-TFT** (656 mg, 1.17 mmol, 1.00 equiv.), and Ir[dF(CF_3)ppy]$_2$(dtbpy)PF_6 (13.1mg, 11.7 µmol, 1.00 mol%) was then added. The vial was evacuated and then filled with argon; this procedure was repeated three times. The vial was placed in 2 cm distance to two 34 W blue LEDs. The temperature was kept at approximately 30 °C with the use of a cooling fan. The reaction mixture was stirred for 16 hours under blue LED irradiation, and then diluted with CH_2Cl_2 (5 mL). The reaction mixture was filtered through a short pad of Celite using CH_2Cl_2 (20 mL) as eluent. The filtrate was collected and concentrated *in vacuo,* and the residue was then purified by flash column chromatography on silica gel, eluting with ethyl acetate/pentane 1:10 (v/v) to afford **1** (227 mg, 71% yield) as a colorless oil.

R*f* = 0.35 (EtOAc/pentane, 1:5, v/v (UV, cerium molybdate))

NMR Spectroscopy:

^{1}H NMR (500 MHz, CD_3CN, 25 °C, δ): 7.42 – 7.35 (m, 3H), 7.24 – 7.07 (m, 3H), 6.89 (d, *J* = 8.7 Hz, 2H), 3.80 (q, *J* = 7.1 Hz, 1H), 3.63 (s, 3H), 1.46 (d, *J* = 7.2 Hz, 3H).

^{13}C {^{1}H} NMR (126 MHz, CD_3CN, 25 °C, δ): 175.2, 160.4 (d, *J* = 245.5 Hz), 157.7, 142.9 (d, *J* = 8.2 Hz), 131.5 (d, *J* = 3.7 Hz), 131.1 (d, *J* = 3.5 Hz), 128.2 (d, *J* = 14.1 Hz), 127.7, 124.7 (d, *J* = 3.5 Hz), 116.2, 115.9 (d, *J* = 23.9 Hz), 52.6, 45.4, 18.7.

^{19}F NMR (471 MHz, CD_3CN, 25 °C, δ): –119.6 (dd, *J* = 11.7, 8.8 Hz).

HRMS-ESI(m/z) calc'd for $C_{16}H_{16}O_3F^+$ $[M+H]^+$, 275.1078; found, 275.1081; deviation: –1.2 ppm.

4-Chlorophenol (2)

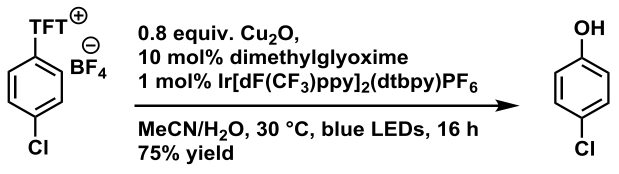

2-TFT **2**

To a 4-mL borosilicate vial, equipped with a magnetic stir bar was added copper(I) oxide (22.9 mg, 0.160 mmol, 0.800 equiv.), dimethylglyoxime (2.3 mg, 20 µmol, 0.10 equiv.), and MeCN/H_2O (1.6 mL, v/v= 5:3). After stirring for 10 mins at ambient temperature, chlorobenzene tetrafluorothianthrenium salt **2-TFT** (97.3 mg, 0.200 mmol, 1.00 equiv.), and Ir[dF(CF_3)ppy]$_2$(dtbpy)PF_6 (2.2 mg, 2.0 µmol, 1.0 mol%) in MeCN (0.40 mL, *c* = 0.50 M) were then added. The vial was evacuated and then filled with argon; this procedure was repeated three times. The vial was placed in 2 cm distance to two 34 W blue LEDs. The temperature was kept at approximately 30 °C with the use of a cooling fan. The reaction mixture was stirred for 16 hours under blue LED irradiation, and then diluted with CH_2Cl_2 (2 mL). The reaction mixture was filtered through a short pad of Celite using CH_2Cl_2 (20 mL) as eluent. The filtrate was collected and concentrated *in vacuo,* and the residue

was then purified by flash column chromatography on silica gel, eluting with ethyl acetate/pentane (1:10, v/v) to afford **2** (19.2 mg, 75% yield) as a colorless soild.

R*f* = 0.29 (EtOAc/pentane ,1:20, v/v, (UV, cerium molybdate)).

NMR Spectroscopy:

^{1}**H NMR** (300 MHz, $CDCl_3$, 25 ºC, δ): 7.24 – 7.13 (m, 2H), 6.87 – 6.70 (m, 2H), 4.99 (brs, 1H, OH).

^{13}**C {^{1}H} NMR** (75 MHz, $CDCl_3$, 25 ºC, δ): 154.1, 129.7, 125.9, 116.8.

HRMS-ESI(m/z) calc'd for $C_6H_4ClO^+$ $[M\text{-}H]^+$, 126.9956; found, 126.9957; deviation: –0.4 ppm.

2-Methoxybenzaldehyde tetrafluorothianthrenium salt (3-TFT)

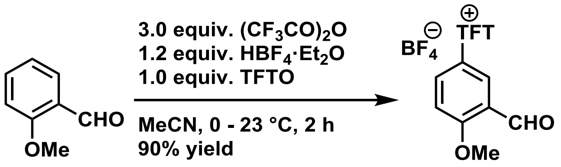

3-TFT

Under ambient atmosphere, a 20 mL round-bottom flask was charged with 2-methoxybenzaldehyde (434 mg, 3.19 mmol, 1.00 equiv.), and dry MeCN (32 mL, *c* = 0.10 M). After cooling to 0°C, $HBF_4{\cdot}OEt_2$ (0.52 mL, 3.8 mmol, 1.2 equiv.) was added to the reaction mixture. Tetrafluorothianthrene-*S*-oxide (970 mg, 3.19 mmol, 1.00 equiv.) was added at 0°C in one portion, followed by trifluoroacetic anhydride (1.33 mL, 2.01 g, 9.56 mmol, 3.00 equiv.) addition in one portion at 0 °C. The vial was sealed with a screw-cap, and the mixture was allowed to stand at 0°C for 1 hour and then warmed to 25°C. After stirring the deep purple reaction mixture at 25 °C for 1 hour, the reaction mixture was concentrated under reduced pressure, and diluted with 30 mL CH_2Cl_2. The CH_2Cl_2 phase was poured onto a saturated aqueous $NaHCO_3$ solution (ca. 20 mL). The mixture was poured into a separatory funnel, and the layers were separated. The CH_2Cl_2 layer was washed with aqueous $NaBF_4$ solution (2 × ca. 20 mL, 5 % w/w), and with water (2 × ca. 20 mL). The CH_2Cl_2 layer was dried over Na_2SO_4, filtered, and the solvent was removed under reduced pressure. The residue was purified by chromatography on silica gel eluting with CH_2Cl_2/*i*-PrOH (30:1, v/v). The product was precipitated by addition of 2 mL CH_2Cl_2, and 20 mL Et_2O. The suspension was decanted, and the solid was dried in vacuo to afford **3-TFT** (1.45 g, 90 % yield) as a colorless solid.

R*f* = 0.35 (MeOH/DCM, 1/15, v/v).

NMR Spectroscopy:

^{1}**H NMR** (500 MHz, CD_3CN, 25 °C, δ): 10.26 (s, 1H), 8.39 (dd, *J* = 9.2 Hz, 7.2 Hz, 2H), 7.97 (dd, *J* = 9.9 Hz, 7.2 Hz, 2H), 7.60 (d, *J* = 2.9 Hz, 1H), 7.47 (dd, *J* = 9.2 Hz, 2.9 Hz, 1H), 7.27 (d, *J* = 9.2 Hz, 1H), 3.98 (s, 3H).

^{13}**C {^{1}H} NMR** (126 MHz, CD_3CN, 25 °C, δ): 188.3, 166.01, 154.8 (dd, *J* = 262.0 Hz, 13.2 Hz), 151.6 (dd, *J* = 255.6 Hz, 13.7 Hz), 136.6, 135.0 (dd, *J* = 8.6, 3.9 Hz), 130.0, 127.0, 125.3 (dd, *J* = 21.8, 2.2 Hz),

121.2 (d, J = 21.8 Hz), 116.3, 115.84 (d, J = 3.6 Hz), 115.78 (d, J = 3.6 Hz), 114.2, 57.9.

^{19}F NMR (471 MHz, CD_3CN, 23ºC, δ): –125.3 (m), –133.5 (m), –151.5 (brs), –151.6 (brs).

HRMS-ESI(m/z) calc'd for $C_{20}H_{11}O_2S_2F_4^+$ $[M-BF_4]^+$, 423.0131; found, 423.0131; deviation: 0 ppm.

5-Hydroxy-2-methoxybenzaldehyde (3)

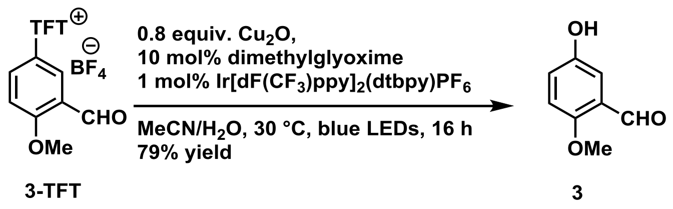

To a 4-mL borosilicate vial, equipped with a magnetic stir bar was added copper(I) oxide (11.5 mg, 80.0 µmol, 0.800 equiv.), dimethylglyoxime (1.2 mg, 10 µmol, 0.10 equiv.), and $MeCN/H_2O$ (0.9 mL, v/v= 2:1). After stirring for 10 mins at ambient temperature, 2-methoxybenzaldehyde tetrafluorothianthrenium salt **3-TFT** (51.0 mg, 0.100 mmol, 1.00 equiv.), and $Ir[dF(CF_3)ppy]_2(dtbpy)PF_6$ (1.1 mg, 1.0 µmol, 1.0 mol%) in MeCN (0.40 mL, c = 0.25 M) were then added. The vial was evacuated and then filled with argon; this procedure was repeated three times. The vial was placed in 2 cm distance to two 34 W blue LEDs. The temperature was kept at approximately 30 °C with the use of a cooling fan. The reaction mixture was stirred for 16 hours under blue LED irradiation, and then diluted with CH_2Cl_2 (2 mL). The reaction mixture was filtered through a short pad of Celite using CH_2Cl_2 (20 mL) as eluent. The filtrate was collected and concentrated *in vacuo,* and the residue was then purified by flash column chromatography on silica gel, eluting with ethyl acetate/pentane (1:5, v/v) to afford **3** (12.1 mg, 79% yield) as a colorless solid.

R*f* = 0.40 (EtOAc/pentane, 1:2, v/v (UV)).

NMR Spectroscopy:

^{1}H NMR (300 MHz, $CDCl_3$, 25 °C, δ): 10.40 (s, 1H), 7.37 (d, J = 3.2 Hz, 1H), 7.12 (dd, J = 9.0, 3.2 Hz, 1H), 6.90 (d, J = 9.0 Hz, 1H), 3.88 (s, 3H).

^{13}C {^{1}H} NMR (75 MHz, $CDCl_3$, 25 °C, δ): 190.4, 156.8, 150.0, 125.1, 123.9, 113.9, 113.5, 56.3.

HRMS-ESI(m/z) calc'd for $C_8H_8O_3^+$ $[M]^+$, 152.0468; found, 152.0470; deviation: –1.2 ppm.

2,2,2-Trifluoro-N-phenylacetamide tetrafluorothianthrenium salt (4-TFT)

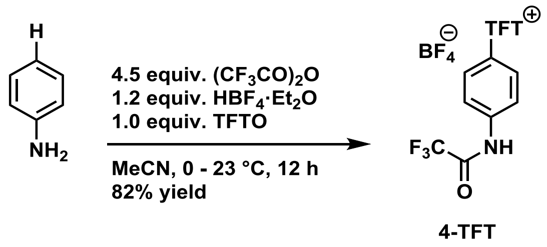

Under an ambient atmosphere, a 20-mL glass vial was charged with aniline (279 mg, 3.00 mmol, 1.00 equiv)

and MeCN (3.0 mL, *c* = 1.0 M). After cooling to 0 °C, $HBF_4{\cdot}OEt_2$ (0.51 mL, 0.59 g, 3.6 mmol, 1.2 equiv) and tetrafluorothianthrene-*S*-oxide (912 mg, 3.00 mmol, 1.00 equiv) was added to the vial while stirring the mixture, leading to a suspension. Subsequently, trifluoroacetic anhydride (1.89 mL, 2.83 g, 13.5 mmol, 4.50 equiv) was added in one portion at 0 °C, resulting in a color change to deep purple. Subsequently, the reaction mixture was allowed to reach 23 ^{o}C and stirred for 12 hours. The solution was diluted with DCM (5 mL) and poured onto a mixture of DCM (30 mL) and saturated aqueous $NaHCO_3$ solution (20 mL). After stirring for 5 min at 23 ^{o}C, the mixture was poured into a separating funnel, and the layers were separated. The DCM layer was washed with aqueous $NaBF_4$ solution (10% w/w, 4 × ca. 20 mL). The DCM layer was dried over Na_2SO_4, filtered, and the solvent was removed under reduced pressure. The residue was purified by chromatography on silica gel eluting with DCM/MeOH (30:1 (v/v)), then the solvent was removed *in vacuo* to afford **4-TFT** (1.1 g, 82%) as a colorless solid.

R*f* = 0.35 (DCM/MeOH, 15:1, v/v).

NMR Spectroscopy:

^{1}H NMR (500 MHz, $CO(CD_3)_2$, 25 °C, δ): 8.82 (dd, *J* = 9.3, 7.3 Hz, 2H), 8.25 (dd, *J* = 10.0, 7.1 Hz, 2H), 8.07 – 7.85 (m, 2H), 7.53 (d, *J* = 9.3 Hz, 2H).

^{13}C {^{1}H} NMR (126 MHz, $CO(CD_3)_2$, 25 °C, δ): 156.2 (q, *J* = 38.1 Hz), 154.6 (dd, *J* = 261.4, 13.1 Hz), 151.3 (dd, *J* = 255.1, 13.7 Hz), 141.9, 135.3 (dd, *J* = 8.6, 3.8 Hz), 130.6, 125.9 (dd, *J* = 22.4, 2.3 Hz), 122.9, 121.0 (d, *J* = 21.9 Hz), 119.5, 116.3 (q, *J* = 287.3 Hz, CF_3), 116.1 (dd, *J* = 7.3, 3.4 Hz).

^{19}F NMR (126 MHz, $CO(CD_3)_2$, 25 °C, δ): –76.4 (s), –126.1 (ddd, *J* = 20.8, 10.3, 7.3 Hz), –134.4 (ddd, *J* = 20.8, 9.3, 7.3 Hz), –149.9 (brs), –150.0 (brs).

HRMS-ESI(m/z) calc'd for $C_{20}H_9NOS_2F_7^+$ $[M-BF_4]^+$, 476.0008; found, 476.0006; deviation: 0.5 ppm.

2,2,2-Trifluoro-N-(4-hydroxyphenyl)acetamide (4)

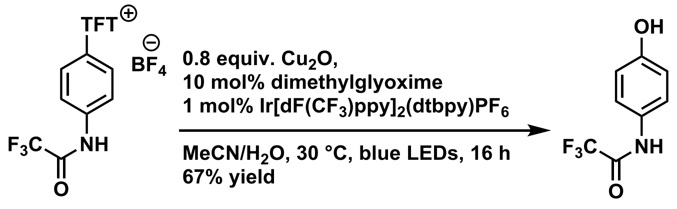

4-TFT 4

To a 4-mL borosilicate vial, equipped with a magnetic stir bar was added copper(I) oxide (57.2 mg, 0.400 mmol, 0.800 equiv.), dimethylglyoxime (5.8 mg, 50 µmol, 0.10 equiv.), and MeCN/H_2O (3 mL, v/v= 3:2). After stirring for 10 mins at ambient temperature, 2,2,2-Trifluoro-N-phenylacetamide thianthrenium salt **4-TT** (282 mg, 0.500 mmol, 1.00 equiv.), and $Ir[dF(CF_3)ppy]_2(dtbpy)PF_6$ (5.6 mg, 5.0 µmol, 1.0 mol%) in MeCN (1.0 mL, *c* = 0.50 M) were then added. The vial was evacuated and then filled with argon; this procedure was repeated three times. The vial was placed in 2 cm distance to two 34 W blue LEDs. The temperature was kept at approximately 30 °C with the use of a cooling fan. The reaction mixture was stirred for 16 hours under blue

LED irradiation, and then diluted with CH_2Cl_2 (2 mL). The reaction mixture was filtered through a short pad of Celite using CH_2Cl_2 (20 mL) as eluent. The filtrate was collected and concentrated *in vacuo,* and the residue was then purified by flash column chromatography on silica gel, eluting with ethyl acetate/pentane (1:10 to 1:4, v/v) to afford **4** (68.9 mg, 67% yield) as a colorless solid.

R*f* = 0.40 (EtOAc/pentane, 1:2, v/v (UV, cerium molybdate)).

NMR Spectroscopy:

^{1}H NMR (500 MHz, CD_3CN, 25 °C, δ): 9.14 (brs, 1H, OH), 7.43 (d, *J* = 9.0 Hz, 2H), 7.19 (s, 1H), 6.85 (d, *J* = 9.0 Hz, 2H).

^{13}C {^{1}H} NMR (126 MHz, CD_3CN, 25 °C, δ) 155.6, 155.5 (q, *J* = 32.2 Hz), 128.6, 123.8, 116.1, 115.9 (q, *J* = 280.4 Hz).

^{19}F NMR (471 MHz, CD_3CN, 25 °C, δ): –76.3 (s).

HRMS-ESI(m/z) calc'd for $C_8H_6NO_2F_3^+$ $[M]^+$, 205.0345; found, 205.0345; deviation: 0 ppm.

Methyl acetyl-L-phenylalate tetrafluorothianthrenium salt (5-TFT)

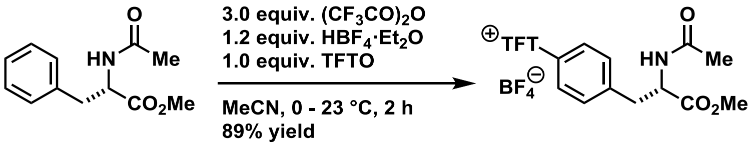

5-TFT

Under ambient atmosphere, a 20 mL round-bottom flask was charged with acetyl-L-phenylalanine methyl ester (664 mg, 3.00 mmol, 1.00 equiv.), and dry MeCN (30 mL, *c* = 0.10 M). After cooling to 0 °C, $HBF_4{\cdot}OEt_2$ (0.49 mL, 3.6 mmol, 1.2 equiv.) was added to the reaction mixture. Tetrafluorothianthrene-*S*-oxide (912 mg, 3.00 mmol, 1.00 equiv.) was added at 0 °C in one portion, followed by trifluoroacetic anhydride (1.25 mL, 1.89 g, 9.00 mmol, 3.00 equiv.) addition in one portion at 0 °C. The vial was sealed with a screw-cap, and the mixture was allowed to stand at 0 °C for 1 hour and then warmed to 25 °C. After stirring the deep purple reaction mixture at 25 °C for 1 hour, the reaction mixture was concentrated under reduced pressure, and diluted with 30 mL CH_2Cl_2. The CH_2Cl_2 phase was poured onto a saturated aqueous $NaHCO_3$ solution (ca. 20 mL). The mixture was poured into a separatory funnel, and the layers were separated. The CH_2Cl_2 layer was washed with aqueous $NaBF_4$ solution (2 × ca. 20 mL, 5 % w/w), and with water (2 × ca. 20 mL). The CH_2Cl_2 layer was dried over Na_2SO_4, filtered, and the solvent was removed under reduced pressure. The residue was purified by chromatography on silica gel eluting with CH_2Cl_2/*i*-PrOH (30:1, v/v). The product was precipitated by addition of 2 mL CH_2Cl_2, and 20 mL Et_2O. The suspension was decanted, and the solid was dried in vacuo to afford **5-TFT** (1.34 g, 89 % yield) as a colorless solid.

R*f* = 0.35 (MeOH/DCM, 1/15, v/v).

NMR Spectroscopy:

^{1}H NMR (500 MHz, CD_3CN, 25 °C, δ): 8.43–8.39 (m, 2H), 7.96 (dd, *J* = 10.0 Hz, 7.1 Hz, 2H), 7.37–7.35

(m, 2H), 7.15–7.12 (m, 2H), 6.76 (d, *J* = 8.1 Hz, 1H), 4.61 – 4.57 (m, 1H), 3.60 (s, 3H), 3.17 – 2.93 (m, 2H), 1.78 (s, 3H).

^{13}C {^{1}H} NMR (126 MHz, CD_3CN, 25 °C, δ): 172.5, 170.7, 154.8 (d, *J* = 261.8 Hz, 13.1 Hz), 151.6 (dd, *J* = 255.4 Hz, 13.6 Hz), 144.9, 135.24 (dd, *J* = 8.5, 3.9 Hz), 132.54, 129.2, 125.6 (dd, *J* = 22.1, 2.3 Hz), 121.6, 121.2 (d, *J* = 21.8 Hz), 115.5–115.4 (m), 54.0, 52.8, 37.7, 22.6.

^{19}F NMR (471 MHz, CD_3CN, 25 °C, δ): –125.3 (m), –133.5 (m), –151.5 (brs), –151.6 (brs).

HRMS-ESI(m/z) calc'd for $C_{24}H_{18}F_4NO_3S_2^+$ $[M\text{-}BF_4]^+$, 508.0659, found, 508.0659, deviation: 0 ppm.

Methyl acetyl-L-tyrosinate (5)

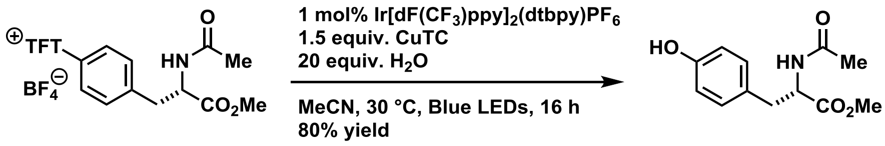

5-TFT 5

To a 4-mL borosilicate vial, equipped with a magnetic stir bar was added Ir[dF(CF_3)ppy]$_2$(dtbpy)PF_6 (2.2 mg, 2.0 µmol, 1.0 mol%), copper(I) thiophene-2-carboxylate (57.2 mg, 0.300 mmol, 1.50 equiv.), and methyl acetyl-L-phenylalate tetrafluorothianthrenium salt **5-TFT** (119 mg, 0.200 mmol, 1.00 equiv.) at 25 °C. The vial was evacuated and then filled with argon; this procedure was repeated three times. MeCN (1 mL, *c* = 0.2 M) was added, followed by H_2O (72.1 mg, 721 µL, 4.00 mmol, 20.0 equiv.). The vial was placed in 2 cm distance to two 34 W blue LEDs. The temperature was kept at approximately 30 °C with the use of a cooling fan. The reaction mixture was stirred for 16 hours under blue LED irradiation, and then diluted with ethyl acetate (1 mL). The reaction mixture was filtered through a short pad of silica using ethyl acetate (20 mL) as eluent. The filtrate was collected and concentrated *in vacuo,* and the residue was then purified by flash column chromatography on silica gel, eluting with ethyl acetate/pentane 1:1 (v/v) to afford **5** (38 mg, 80% yield) as a colorless solid.

R*f* = 0.30 (ethyl acetate/pentane, 1:2, v/v (UV, cerium molybdate))

NMR Spectroscopy:

^{1}H NMR (500 MHz, CD_3CN, 25 °C, δ): 7.01 (d, *J* = 8.5 Hz, 2H), 6.72 (d, *J* = 8.6 Hz, 2H), 6.70 – 6.64 (m, 1H), 4.54 (td, *J* = 7.9 Hz, 5.8 Hz, 1H), 3.63 (s, 3H), 2.98 (dd, *J* = 14.0 Hz, 5.9 Hz, 1H), 2.85 (dd, *J* = 13.9 Hz, 7.9 Hz, 1H), 1.85 (s, 3H).

^{13}C {^{1}H} NMR (126 MHz, CD_3CN, 25 °C, δ): 172.7, 170.6, 156.4, 130.9, 128.4, 115.7, 54.6, 52.2, 36.9, 22.3.

HRMS-ESI(m/z) calc'd for $C_{12}H_{15}NO_4Na^+$ $[M+Na]^+$, 260.0893; found, 260.0896. Deviation: – 0.9 ppm.

Hydroxy-nefiracetam (6)

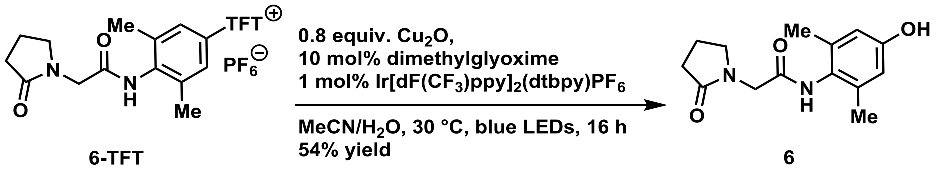

To a 4-mL borosilicate vial, equipped with a magnetic stir bar was added copper(I) oxide (11.5 mg, 80.0 µmol, 0.800 equiv.), dimethylglyoxime (1.2 mg, 10 µmol, 0.10 equiv.), and MeCN/H_2O (0.9 mL, v/v= 2:1). After stirring for 10 mins at ambient temperature, nefiracetam tetrafluorothianthrenium salt **6-TFT** (67.9 mg, 0.100 mmol, 1.00 equiv.), and Ir[dF(CF_3)ppy]$_2$(dtbpy)PF_6 (1.1 mg, 1.0 µmol, 1.0 mol%) in MeCN (0.40 mL, *c* = 0.25 M) were then added. The vial was evacuated and then filled with argon; this procedure was repeated three times. The vial was placed in 2 cm distance to two 34 W blue LEDs. The temperature was kept at approximately 30 °C with the use of a cooling fan. The reaction mixture was stirred for 16 hours under blue LED irradiation, and then diluted with CH_2Cl_2 (2 mL). The reaction mixture was filtered through a short pad of Celite using CH_2Cl_2 (20 mL) as eluent. The filtrate was collected and concentrated *in vacuo,* and the residue was then purified by flash column chromatography on silica gel, eluting with DCM/MeOH (20:1, v/v) to afford **6** with impurities. Further purification of **6** by HPLC (YMC-Actus Triart C18 (30×150 mm: 5 µM), MeOH/TFA in water (1/1000, v/v) = 50:50, flow rate = 42.5 mL/min, 25 °C, retention time; 2.1 min) provided **6** (14.1 mg, 54%) as a colorless solid.

R*f* = 0.58 (MeOH/CH_2Cl_2, 1:10, v/v (UV, cerium molybdate)).

NMR Spectroscopy:

^{1}H NMR (500 MHz, CD_3OD, 25 °C, δ): 6.51 (s, 2H), 4.15 (s, 2H), 3.62 – 3.55 (m, 2H), 2.44 (t, *J* = 8.1 Hz, 2H), 2.13 (m, 8H).

^{13}C {^{1}H} NMR (101 MHz, CD_3OD, 25 °C, δ): 177.2, 168.2, 156.0, 136.7, 125.4, 114.2, 48.3, 45.2, 30.0, 17.4, 17.1.

HRMS-ESI(m/z) calc'd for $C_{14}H_{17}N_2O_3^+$ $[M]^+$, 261.1245; found, 261.1247; deviation: –0.8 ppm.

Bromobenzene tetrafluorothianthrenium salt (7-TFT)

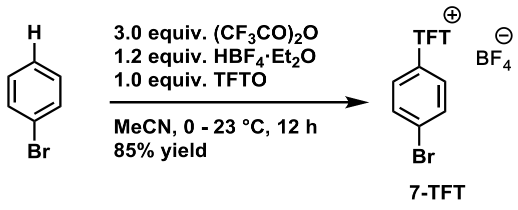

Under an ambient atmosphere, a 20-mL glass vial was charged with bromobenzene (780 mg, 5.00 mmol, 1.00 equiv) and MeCN (5.0 mL, *c* = 1.0 M). After cooling to 0 °C, $HBF_4{\cdot}OEt_2$ (0.82 mL, 0.97 g, 6.0 mmol, 1.2 equiv) and tetrafluorothianthrene-*S*-oxide (1.51 g, 5.00 mmol, 1.00 equiv) was added to the vial while

stirring the mixture, leading to a suspension. Subsequently, trifluoroacetic anhydride (2.1 mL, 3.1 g, 15 mmol, 3.0 equiv) was added in one portion at 0 °C, resulting in a color change to deep purple. Subsequently, the reaction mixture was allowed to reach 23 °C and stirred for 12 hours. The solution was diluted with DCM (5 mL) and poured onto a mixture of DCM (30 mL) and saturated aqueous $NaHCO_3$ solution (20 mL). After stirring for 5 min at 23 °C, the mixture was poured into a separating funnel, and the layers were separated. The DCM layer was washed with aqueous $NaBF_4$ solution (10% w/w, 4 × ca. 20 mL). The DCM layer was dried over Na_2SO_4, filtered, and the solvent was removed under reduced pressure. The residue was purified by chromatography on silica gel eluting with DCM/MeOH (30:1 (v/v)), then the solvent was removed *in vacuo* to afford **7-TFT** (2.15 g, 85%) as a grey powder.

R*f* = 0.35 (MeOH/DCM, 1/15, v/v).

NMR Spectroscopy:

^{1}H NMR (600 MHz, CD_2Cl_2, 25 °C, δ): 8.58 (dd, *J* = 8.4, 7.1 Hz, 2H), 7.77 (dd, *J* = 9.1, 6.7 Hz, 2H), 7.67 (d, *J* = 9.1 Hz, 2H), 7.08 (d, *J* = 9.1 Hz, 2H).

^{13}C {^{1}H} NMR (151 MHz, CD_2Cl_2, 25 °C, δ): 154.5 (dd, *J* = 266.1, 13.1 Hz), 151.4 (dd, *J* = 260.5, 13.4 Hz), 134.5, 134.4 (dd, *J* = 8.0, 4.2 Hz), 129.7, 129.4, 125.5 (dd, *J* = 21.8, 2.6 Hz), 122.0, 120.3 (d, *J* = 21.3 Hz), 114.7 (dd, *J* = 6.8, 3.6 Hz).

^{19}F NMR (376 MHz, CD_2Cl_2, 25 °C, δ): –121.7 (d, *J* = 20.7 Hz), –129.6 (d, *J* = 20.8 Hz), –149.3 (brs), –149.4 (brs).

HRMS-ESI (m/z) calculated for $C_{18}H_8BrF_4S_2^+$ $[M\text{-}BF_4]^+$, 442.9182; found, 442.9185; deviation: –0.7 ppm.

4-Bromophenol (7)

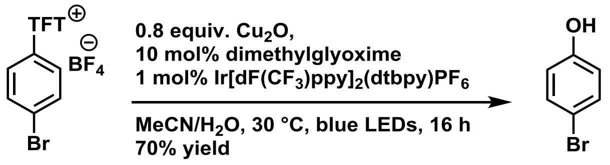

7-TFT 7

To a 4-mL borosilicate vial, equipped with a magnetic stir bar was added copper(I) oxide (11.5 mg, 80.0 µmol, 0.800 equiv.), dimethylglyoxime (1.2 mg, 10 µmol, 0.10 equiv.), and $MeCN/H_2O$ (0.9 mL, v/v= 2:1). After stirring for 10 mins at ambient temperature, bromobenzene tetrafluorothianthrenium salt **7-TFT** (48.7 mg, 0.100 mmol, 1.00 equiv.), and $Ir[dF(CF_3)ppy]_2(dtbpy)PF_6$ (1.1 mg, 1.0 µmol, 1.0 mol%) in MeCN (0.40 mL, *c* = 0.25 M) were then added. The vial was evacuated and then filled with argon; this procedure was repeated three times. The vial was placed in 2 cm distance to two 34 W blue LEDs. The temperature was kept at approximately 30 °C with the use of a cooling fan. The reaction mixture was stirred for 16 hours under blue LED irradiation, and then diluted with CH_2Cl_2 (2 mL). The reaction mixture was filtered through a short pad of Celite using CH_2Cl_2 (20 mL) as eluent. The filtrate was collected and concentrated *in vacuo,* and the residue was then purified by flash column chromatography on silica gel, eluting with ethyl acetate/pentane (1:20 to

1:10, v/v) to afford **7** (12.1 mg, 70% yield) as a brown oil.

R*f* = 0.29 (EtOAc/pentane ,1:20, v/v, (UV, cerium molybdate)).

NMR Spectroscopy:

^{1}H NMR (300 MHz, $CDCl_3$, 25 °C, δ): 7.33 (d, J = 8.9 Hz, 2H), 6.72 (d, J = 8.9 Hz, 2H).

^{13}C {^{1}H} NMR (75 MHz, $CDCl_3$, 25 °C, δ): 154.7, 132.6, 117.3, 113.1.

HRMS-ESI(m/z) calc'd for $C_6H_5OBr^+$ $[M]^+$, 171.9520; found, 171.9518; deviation: –1.1 ppm.

2-Methoxybenzoate tetrafluorothianthrenium salt (8-TFT)

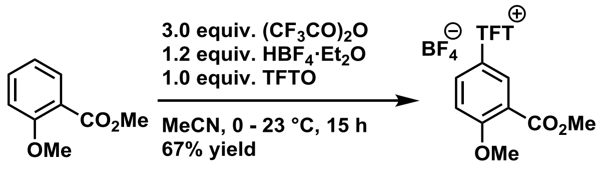

8-TFT

Under ambient atmosphere, a 20 mL round-bottom flask was charged with 2-methoxybenzoate (49.8 mg, 0.300 mmol, 1.00 equiv.), and dry MeCN (3.0 mL, *c* = 0.10 M). After cooling to 0 °C, tetrafluorothianthrene-*S*-oxide (91 mg, 0.30 mmol, 1.0 equiv.) was added to the reaction mixture in one portion. Trifluoroacetic anhydride (125 µL, 189 mg, 0.900 mmol, 3.00 equiv.) was added at 0 °C in one portion, followed by $HBF_4{\cdot}OEt_2$ (49 µL, 58 mg, 0.36 mmol, 1.2 equiv.) dropwise addition at 0 °C. The vial was sealed with a screw-cap, and the mixture was allowed to stand at 0 °C for 1 hour and then warmed to 25 °C. After stirring the deep purple reaction mixture at 25 °C for 14 hours, the reaction mixture was concentrated under reduced pressure, and diluted with 30 mL CH_2Cl_2. The CH_2Cl_2 phase was poured onto a saturated aqueous $NaHCO_3$ solution (ca. 20 mL). The mixture was poured into a separatory funnel, and the layers were separated. The CH_2Cl_2 layer was washed with aqueous $NaBF_4$ solution (2 × ca. 20 mL, 5 % w/w), and with water (2 × ca. 20 mL). The CH_2Cl_2 layer was dried over Na_2SO_4, filtered, and the solvent was removed under reduced pressure. The residue was purified by chromatography on silica gel eluting with CH_2Cl_2/MeOH (30:1, v/v). The product was precipitated by addition of 2 mL CH_2Cl_2, and 20 mL Et_2O. The suspension was decanted, and the solid was dried in vacuo to afford **8-TFT** (109 mg, 67 % yield) as a colorless solid.

R*f* = 0.35 (MeOH/DCM, 1/15, v/v).

NMR Spectroscopy:

^{1}H NMR (500 MHz, CD_3CN, 25 °C, δ): 8.38 (dd, *J* = 9.2, 7.2 Hz, 2H), 7.97 (dd, *J* = 10.0, 7.1 Hz, 2H), 7.56 (d, *J* = 2.8 Hz, 1H), 7.39 (dd, *J* = 9.2, 2.9 Hz, 1H), 7.20 (d, *J* = 9.2 Hz, 1H), 3.89 (s, 3H), 3.78 (s, 3H).

^{13}C {^{1}H} NMR (126 MHz, CD_3CN, 25 °C, δ): 165.3, 163.1, 154.6 (dd, *J* = 261.7, 13.1 Hz), 151.5 (dd, *J* = 255.6, 13.6 Hz), 134.8 (dd, *J* = 8.6, 3.9 Hz), 134.5, 132.2, 125.1 (dd, *J* = 22.2, 2.6 Hz), 123.9, 121.1 (d, *J* = 21.9 Hz), 115.7 (dd, *J* = 7.3, 3.5 Hz), 115.5, 112.7, 57.5, 53.1.

^{19}F NMR (471 MHz, CD_3CN, 25 °C, δ): –125.5 (m), –133.6 (m), –151.0 (brs), –151.1 (brs).

HRMS-ESI(m/z) calc'd for $C_{21}H_{13}O_3S_2F_4^+$ [M-BF$_4$]$^+$, 453.0237; found, 453.0231; deviation: 1.2 ppm.

5-Hydroxy-2-methoxybenzoate (8)

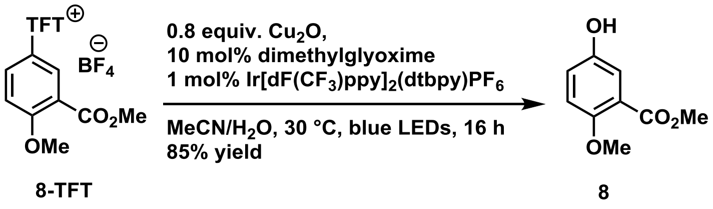

To a 4-mL borosilicate vial, equipped with a magnetic stir bar was added copper(I) oxide (11.5 mg, 80.0 µmol, 0.800 equiv.), dimethylglyoxime (1.2 mg, 10 µmol, 0.10 equiv.), and MeCN/H_2O (0.9 mL, v/v= 2:1). After stirring for 10 mins at ambient temperature, 2-methoxybenzoate tetrafluorothianthrenium salt **8-TFT** (54.0 mg, 0.100 mmol, 1.00 equiv.), and Ir[dF(CF_3)ppy]$_2$(dtbpy)PF_6 (1.1 mg, 1.0 µmol, 1.0 mol%) in MeCN (0.40 mL, *c* = 0.25 M) were then added. The vial was evacuated and then filled with argon; this procedure was repeated three times. The vial was placed in 2 cm distance to two 34 W blue LEDs. The temperature was kept at approximately 30 °C with the use of a cooling fan. The reaction mixture was stirred for 16 hours under blue LED irradiation, and then diluted with CH_2Cl_2 (2 mL). The reaction mixture was filtered through a short pad of Celite using CH_2Cl_2 (20 mL) as eluent. The filtrate was collected and concentrated *in vacuo,* and the residue was then purified by flash column chromatography on silica gel, eluting with ethyl acetate/pentane (1:5, v/v) to afford **8** (15.5 mg, 85% yield) as a colorless solid.

R*f* = 0.35 (EtOAc/pentane, 1:2, v/v (UV, cerium molybdate)).

NMR Spectroscopy:

^{1}H NMR (500 MHz, $CDCl_3$, 25 °C, δ): 7.32 (d, *J* = 3.2 Hz, 1H), 6.99 (dd, *J* = 8.9, 3.2 Hz, 1H), 6.87 (d, *J* = 8.9 Hz, 1H), 5.21 (brs, 1H, OH), 3.89 (s, 3H), 3.84 (s, 3H).

^{13}C {^{1}H} NMR (126 MHz, $CDCl_3$, 25 °C, δ): 166.7, 153.6, 149.2, 120.7, 120.5, 118.3, 114.1, 56.9, 52.4.

HRMS-ESI(m/z) calc'd for $C_9H_{10}O_4$ [M]$^+$, 182.0574; found, 182.0576; deviation: –1.1 ppm.

Xanthone tetrafluorothianthrenium salt (9-TFT)

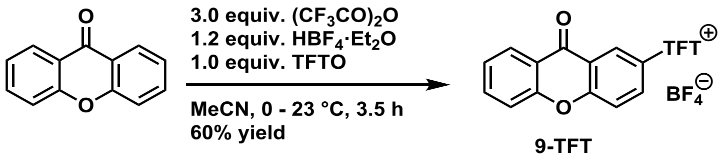

Under an ambient atmosphere, a 20-mL glass vial was charged with xanthone (392 mg, 2.00 mmol, 1.00 equiv) and MeCN (10 mL, *c* = 0.20 M). Trifluoroacetic anhydride (0.834 mL, 1.26 g, 6.00 mmol, 3.00 equiv.) was added while stirring the reaction mixture at 25 °C. After cooling to 0 °C, tetrafluorothianthrene-*S*-oxide (608 mg, 2.00 mmol, 1.00 equiv.) was added in one portion, followed by the dropwise addition of $HBF_4·OEt_2$ (327 µL, 389 mg, 2.40 mmol, 1.20 equiv.). The mixture was stirred at 0°C for 1 hour, then warmed

to 23°C for 2.5 hours. The solution was diluted with CH_2Cl_2 (5 mL) and poured onto a mixture of CH_2Cl_2 (25 mL) and saturated aqueous $NaHCO_3$ solution (30 mL). After stirring for 5 min, the mixture was poured into a separating funnel, and the aqueous phase was extracted with DCM (2 × 20 mL). The DCM layer was washed with aqueous $NaBF_4$ solution (10% w/w, 2 × ca. 25 mL). The DCM layer was dried over Na_2SO_4, filtered, and the solvent was removed under reduced pressure. The residue was purified by chromatography on silica gel eluting with DCM/*i*-PrOH (50:1 (v/v)), then the solvent was removed *in vacuo* to afford **9-TFT** (689 mg, 60% yield) as a colorless solid.

R*f* = 0.35 (MeOH/DCM, 1/15, v/v).

NMR Spectroscopy:

^{1}H NMR (500 MHz, CD_3CN, 25 °C, δ): 8.49 (dd, *J* = 9.1, 7.1 Hz, 2H), 8.15 (dd, *J* = 8.0, 1.8 Hz, 1H), 8.07 (d, *J* = 2.8 Hz, 1H), 7.98 (dd, *J* = 9.9, 7.0 Hz, 2H), 7.84 (ddd, *J* = 8.7, 7.1, 1.8 Hz, 1H), 7.67 (d, *J* = 9.1 Hz, 1H), 7.61 – 7.53 (m, 2H), 7.45 (ddd, *J* = 8.1, 7.1, 1.1 Hz, 1H).

^{13}C {^{1}H} NMR (126 MHz, CD_3CN, 25 °C, δ) 175.4, 159.0, 156.4, 154.5 (dd, *J* = 262.4, 13.8 Hz), 150.2 (dd, *J* = 255.9, 13.6 Hz), 137.0, 135.0 (d, *J* = 12.3 Hz), 134.1, 128.5, 126.8, 125.9, 125.3 (dd, *J* = 22.7, 2.2 Hz), 123.3, 121.8, 121.0 (d, *J* = 22.0 Hz), 118.9, 118.3, 115.1 (d, *J* = 7.0 Hz).

^{19}F NMR (471 MHz, CD_3CN, 25 °C, δ): –124.8 (m), –133.26 (dt, *J* = 20.2, 8.0 Hz), –151.03 (brs), –151.08(brs).

HRMS-ESI(m/z) calc'd for $C_{25}H_{11}F_4O_2S_2^+$ [M-BF$_4$]$^+$, 483.0131; found, 483.0137; deviation: –1.2 ppm.

Hydroxyl xanthone (9)

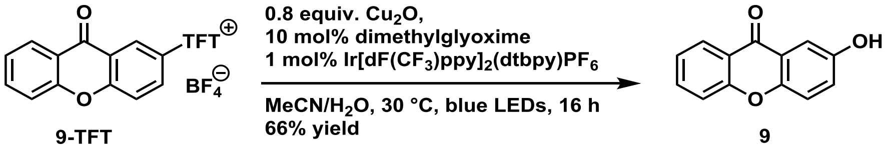

9-TFT 9

To a 4-mL borosilicate vial, equipped with a magnetic stir bar was added copper(I) oxide (22.9 mg, 0.160 mmol, 0.800 equiv.), dimethylglyoxime (2.3 mg, 20 µmol, 0.10 equiv.), and MeCN/H_2O (1.6 mL, v/v= 5:3). After stirring for 10 mins at ambient temperature, 6-methyl-4-chromanone tetrafluorothianthrenium salt **9-TFT** (114 mg, 0.200 mmol, 1.00 equiv.), and Ir[dF(CF_3)ppy]$_2$(dtbpy)PF_6 (2.2 mg, 2.0 µmol, 1.0 mol%) in MeCN (0.40 mL, *c* = 0.50 M) were then added. The vial was evacuated and then filled with argon; this procedure was repeated three times. The vial was placed in 2 cm distance to two 34 W blue LEDs. The temperature was kept at approximately 30 °C with the use of a cooling fan. The reaction mixture was stirred for 16 hours under blue LED irradiation, and then diluted with CH_2Cl_2 (2 mL). The reaction mixture was filtered through a short pad of Celite using CH_2Cl_2 (20 mL) as eluent. The filtrate was collected and concentrated *in vacuo,* and the residue was then purified by flash column chromatography on silica gel, eluting with ethyl acetate/pentane (1:10, v/v) to afford **9** (27.8 mg, 66% yield) as a colorless solid.

R*f* = 0.43 (EtOAc/pentane, 1:2, v/v (UV, cerium molybdate))

NMR Spectroscopy:

^{1}H NMR (500 MHz, CD_3CN, 25 °C, δ): 9.03 (brs, 1H, OH), 8.24 (dd, *J* = 8.0, 1.7 Hz, 1H), 7.82 (td, *J* = 7.9, 7.1, 1.7 Hz, 1H), 7.63 (d, *J* = 3.1 Hz, 1H), 7.57 (d, *J* = 8.5 Hz, 1H), 7.51 (d, *J* = 9.0 Hz, 1H), 7.43 (t, *J* = 7.5 Hz, 1H), 7.37 (dd, *J* = 9.0, 3.1 Hz, 1H).

^{13}C {^{1}H} NMR (126 MHz, $CO(CD_3)_2$, 25 °C, δ): 176.9, 157.0, 154.9, 150.9, 135.7, 127.0, 125.1, 124.6, 123.2, 121.9, 120.3, 118.9, 109.9.

HRMS-ESI(m/z) calc'd for $C_{13}H_8O_3^+$ $[M]^+$, 212.0468; found, 212.0467; deviation: 0.4 ppm.

(*R*)-4-(4-hydroxybenzyl)-3-propionyloxazolidin-2-one (10)

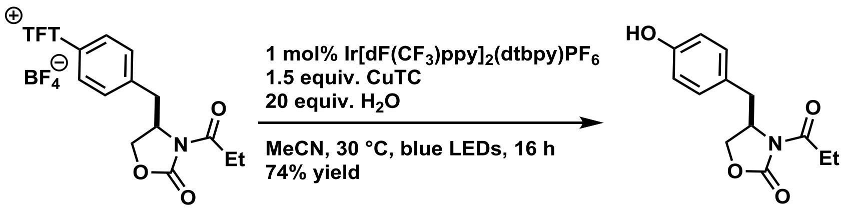

10-TFT **10**

To a 4-mL borosilicate vial, equipped with a magnetic stir bar was added copper(I) thiophene-2-carboxylate (57.2 mg, 0.300 mmol, 1.50 equiv.), (*R*)-4-(4-hydroxybenzyl)-3-propionyloxazolidin-2-one tetrafluoro-thianthrenium salt **10-TFT** (121 mg, 0.200 mmol, 1.00 equiv.), $Ir[dF(CF_3)ppy]_2(dtbpy)PF_6$ (1.1 mg, 1.0 μmol, 1.0 mol%), water (72 μL, 36 mg, 4.0 mmol, 20 equiv.) and MeCN (1.0 mL, *c* = 0.20 M). The vial was evacuated and then filled with argon; this procedure was repeated three times. The vial was placed in 2 cm distance to two 34 W blue LEDs. The temperature was kept at approximately 30 °C with the use of a cooling fan. The reaction mixture was stirred for 16 hours under blue LED irradiation, and then diluted with CH_2Cl_2 (2 mL). The reaction mixture was filtered through a short pad of Celite using CH_2Cl_2 (20 mL) as eluent. The filtrate was collected and concentrated *in vacuo,* and the residue was then purified by flash column chromatography on silica gel, eluting with ethyl acetate/pentane (1:5 to 1:2, v/v) to afford **10** (37.1 mg, 74% yield) as a colorless solid.

R*f* = 0.31 (EtOAc/pentane, 1:2, v/v (UV, cerium molybdate)).

NMR Spectroscopy:

^{1}H NMR (500 MHz, CD_3CN, 25 °C, δ): 7.11 – 6.98 (m, 2H), 6.88 (brs, 1H, OH), 6.75 (d, *J* = 8.5 Hz, 2H), 4.78 – 4.56 (m, 1H), 4.24 (dd, *J* = 9.0, 8.2 Hz, 1H), 4.14 (dd, *J* = 9.0, 2.8 Hz, 1H), 3.04 – 2.72 (m, 4H), 1.12 (t, *J* = 7.3 Hz, 3H).

^{13}C {^{1}H} NMR (126 MHz, CD_3CN, 25 °C, δ): 174.7, 156.9, 154.8, 131.7, 127.7, 116.2, 67.2, 55.6, 37.0, 29.7, 8.7.

HRMS-ESI(m/z) calc'd for $C_{13}H_{14}N_1O_4$ $[M+H]^+$, 250.1074; found, 250.1071; deviation: 1.2 ppm.

Hydroxy-boscalid (11)

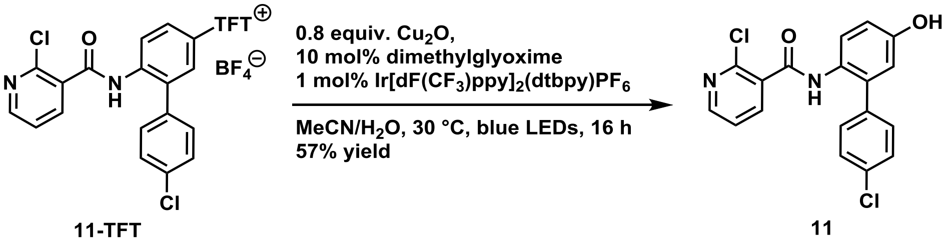

To a 4-mL borosilicate vial, equipped with a magnetic stir bar was added copper(I) oxide (11.5 mg, 80.0 µmol, 0.800 equiv.), dimethylglyoxime (1.2 mg, 10 µmol, 0.10 equiv.), and MeCN/H_2O (0.9 mL, v/v= 2:1). After stirring for 10 mins at ambient temperature, boscalid tetrafluorothianthrenium salt **11-TFT** (67.9 mg, 0.100 mmol, 1.00 equiv.), and Ir[dF(CF_3)ppy]$_2$(dtbpy)PF_6 (1.1 mg, 1.0 µmol, 1.0 mol%) in MeCN (0.40 mL, *c* = 0.25 M) were then added. The vial was evacuated and then filled with argon; this procedure was repeated three times. The vial was placed in 2 cm distance to two 34 W blue LEDs. The temperature was kept at approximately 30 °C with the use of a cooling fan. The reaction mixture was stirred for 16 hours under blue LED irradiation, and then diluted with CH_2Cl_2 (2 mL). The reaction mixture was filtered through a short pad of Celite using CH_2Cl_2 (20 mL) as eluent. The filtrate was collected and concentrated *in vacuo,* and the residue was then purified by flash column chromatography on silica gel, eluting with ethyl acetate/ pentane (1:5 to 1:2, v/v) to afford **11** (20.3 mg, 57%) as a colorless solid.

R*f* = 0.25 (EtOAc/pentane, 1:1, v/v (UV)).

NMR Spectroscopy:

^{1}H NMR (500 MHz, CD_3CN, 25 °C, δ): 8.39 (dd, *J* = 4.8, 2.0 Hz, 1H), 8.16 (brs, 1H, OH), 7.74 (dd, *J* = 7.6, 2.0 Hz, 1H), 7.53 (d, *J* = 8.6 Hz, 1H), 7.48 – 7.21 (m, 5H), 6.89 (dd, *J* = 8.6, 2.8 Hz, 1H), 6.79 (d, *J* = 2.8 Hz, 1H).

^{13}C {^{1}H} NMR (126 MHz, CD_3CN, 25 °C, δ): 164.8, 155.9, 150.9, 147.3, 138.2, 138.1, 137.9, 133.5, 133.1, 131.2, 128.8, 128.5, 128.4, 126.3, 123.2, 117.7, 117.0, 115.5.

HRMS-ESI(m/z) calc'd for $C_{18}H_{11}N_2O_2Cl_2^+$ [M-H]$^+$, 357.0203; found, 357.0206; deviation: –0.9 ppm.

Methyl 2,4-dimethylbenzoate tetrafluorothianthrenium salt (12-TFT)

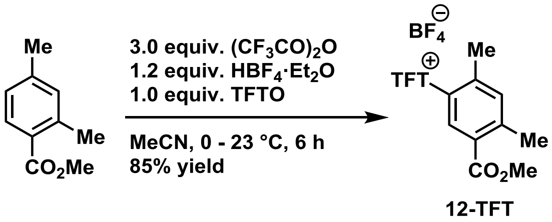

Under ambient atmosphere, a 20 mL round-bottom flask was charged with 2,4-dimethyl-methylbenzoate (328 mg, 2.00 mmol, 1.00 equiv.), and dry MeCN (10 mL, *c* = 0.20 M). After cooling to 0 °C, trifluoroacetic

anhydride (834 μL, 1.26 g, 6.00 mmol, 3.00 equiv.) was added to the reaction mixture. $HBF_4 \cdot OEt_2$ (327 μL, 2.40 mmol, 1.20 equiv.) was added at 0 °C in one portion, followed by tetrafluorothianthrene-*S*-oxide (752 mg, 2.00 mmol, 1.00 equiv.) addition in one portion at 0 °C. The vial was sealed with a screw-cap, and the mixture was allowed to stand at 0 °C for 1 hour and then warmed to 25 °C. After stirring the deep purple reaction mixture at 25 °C for 5 hours, the reaction mixture was concentrated under reduced pressure, and diluted with 30 mL CH_2Cl_2. The CH_2Cl_2 phase was poured onto a saturated aqueous $NaHCO_3$ solution (ca. 20 mL). The mixture was poured into a separatory funnel, and the layers were separated. The CH_2Cl_2 layer was washed with aqueous $NaBF_4$ solution (2 × ca. 20 mL, 5 % w/w), and with water (2 × ca. 20 mL). The CH_2Cl_2 layer was dried over Na_2SO_4, filtered, and the solvent was removed under reduced pressure. The residue was purified by chromatography on silica gel eluting with CH_2Cl_2/*i*-PrOH (30:1, v/v). The product was precipitated by addition of 5 mL CH_2Cl_2, and 10 mL Et_2O. The suspension was decanted, and the solid was dried in vacuo to afford **12-TFT** (998 mg, 93 %) as a colorless solid.

R*f* = 0.35 (MeOH/DCM, 1/15, v/v).

NMR Spectroscopy:

^{1}H NMR (500 MHz, CD_3CN, 25 °C, δ): 8.24 (ddt, *J* = 9.0, 5.5, 1.7 Hz, 2H), 8.00 (dd, *J* = 9.9, 7.0 Hz, 2H), 7.47 (s, 1H), 7.45 (s, 1H), 3.76 (s, 3H), 2.63 (s, 3H), 2.54 (s, 3H).

^{13}C {^{1}H} NMR (126 MHz, $CDCl_3$, 25 °C, δ): 165.4, 153.7 (dd, *J* = 266.1, 13.1 Hz), 150.8 (dd, *J* = 260.0, 13.2 Hz), 147.7, 143.7, 137.8, 135.3 (dd, *J* = 7.9, 3.9 Hz), 131.5, 129.0, 124.6 (d, *J* = 21.6 Hz), 120.1 (d, *J* = 21.0 Hz), 117.0, 113.6 (dd, *J* = 6.5, 3.4 Hz), 52.8, 21.7, 20.2.

^{19}F NMR (471 MHz, CD_3CN, 25 °C, δ): –125.55 (ddd, *J* = 20.3, 9.8, 7.0 Hz), –133.24 (ddd, *J* = 20.3, 9.1, 7.0 Hz), –151.57 (brs), –151.62 (brs).

HRMS-ESI (m/z) calculated for $C_{22}H_{15}F_4O_2S_2^+$ $[M-BF_4]^+$, 451.0444; found, 451.0448; deviation: –0.9 ppm.

Methyl 5-hydroxy-2,4-dimethylbenzoate (12)

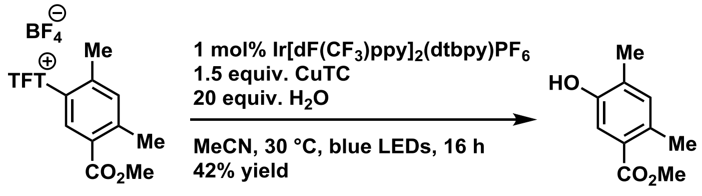

12-TFT **12**

To a 4-mL borosilicate vial, equipped with a magnetic stir bar was added copper(I) thiophene-2-carboxylate (57.2 mg, 0.300 mmol, 1.50 equiv.), methyl 2,4-dimethylbenzoate tetrafluorothianthrenium salt **12-TFT** (108 mg, 0.200 mmol, 1.00 equiv.), $Ir[dF(CF_3)ppy]_2(dtbpy)PF_6$ (1.1 mg, 1.0 μmol, 1.0 mol%), water (72 μL, 36 mg, 4.0 mmol, 20 equiv.), and MeCN (1.0 mL, *c* = 0.2 M). The vial was evacuated and then filled with argon; this procedure was repeated three times. The vial was placed in 2 cm distance to two 34 W blue LEDs. The temperature was kept at approximately 30 °C with the use of a cooling fan. The reaction mixture was stirred for 16 hours under blue LED irradiation, and then diluted with CH_2Cl_2 (2 mL). The reaction mixture was

filtered through a short pad of Celite using CH_2Cl_2 (20 mL) as eluent. The filtrate was collected and concentrated *in vacuo,* and the residue was then purified by flash column chromatography on silica gel, eluting with ethyl acetate/pentane (1:10 to 1:5, v/v) to afford **12** (15.1 mg, 42% yield) as a colorless solid.

R*f* = 0.40 (EtOAc/pentane, 1:10, v/v, (UV, cerium molybdate)).

NMR Spectroscopy:

^{1}H NMR (500 MHz, $CDCl_3$, 25 °C, δ): 7.38 (s, 1H), 6.99 (s, 1H), 3.87 (s, 3H), 2.49 (s, 3H), 2.25 (s, 3H).

^{13}C {^{1}H} NMR (126 MHz, CD_3CN, 25 °C, δ): 168.4, 153.5, 135.0, 131.9, 130.3, 128.6, 117.0, 52.2, 20.7, 16.1.

HRMS-ESI(m/z) calc'd for $C_{10}H_{12}O_3Na_1^+$ $[M+Na]^+$, 203.0679; found, 203.0678; deviation: –0.2 ppm.

2-Methoxy-benzonitrile thianthrenium salt (13-TT)

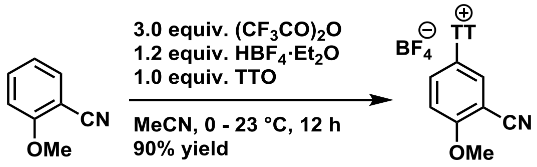

13-TT

Under an ambient atmosphere, a 20-mL glass vial was charged with 2-methoxy-benzonitrile (266 mg, 2.00 mmol, 1.00 equiv) and MeCN (3.0 mL, *c* = 0.67 M). After cooling to 0 °C, $HBF_4{\cdot}OEt_2$ (0.34 mL, 0.40 g, 2.4 mmol, 1.2 equiv) and thianthrene-*S*-oxide (464 mg, 2.00 mmol, 1.00 equiv) was added to the vial while stirring the mixture, leading to a suspension. Subsequently, trifluoroacetic anhydride (0.84 mL, 1.3 g, 6.0 mmol, 3.0 equiv) was added in one portion at 0 °C, resulting in a color change to deep purple. Subsequently, the reaction mixture was allowed to reach 23 ^{o}C and stirred for 12 hours. The solution was diluted with DCM (5 mL) and poured onto a mixture of DCM (30 mL) and saturated aqueous $NaHCO_3$ solution (20 mL). After stirring for 5 min at 23 ^{o}C, the mixture was poured into a separating funnel, and the layers were separated. The DCM layer was washed with aqueous $NaBF_4$ solution (10% w/w, 4 × ca. 20 mL). The DCM layer was dried over Na_2SO_4, filtered, and the solvent was removed under reduced pressure. The residue was purified by chromatography on silica gel eluting with DCM/MeOH (30:1 (v/v)), then the solvent was removed *in vacuo* to afford **13-TT** (770 mg, 90% yield) as a colorless solid.

R*f* = 0.35 (DCM/MeOH, 15:1, v/v).

NMR Spectroscopy:

^{1}H NMR (500 MHz, CD_3CN, 25 °C, δ): 8.32 (dd, *J* = 8.0, 1.4 Hz, 2H), 7.95 (dd, *J* = 7.9, 1.4 Hz, 2H), 7.87 (td, *J* = 7.7, 1.4 Hz, 2H), 7.79 (td, *J* = 7.7, 1.4 Hz, 2H), 7.40 (d, *J* = 2.7 Hz, 1H), 7.34 (dd, *J* = 9.3, 2.7 Hz, 1H), 7.16 (d, *J* = 9.3 Hz, 1H), 3.92 (s, 3H).

^{13}C {^{1}H} NMR (126 MHz, CD_3CN, 25 °C, δ): 165.4, 137.4, 136.2, 135.9, 135.7, 134.7, 131.8, 131.0, 119.4, 115.5, 115.0, 114.9, 104.5, 58.2.

^{19}F NMR (471 MHz, CD_3CN, 25 °C, δ):–151.5 (brs), –151.6 (brs).

HRMS-ESI(m/z) calc'd for $C_{20}H_{14}NOS_2^+$ $[M]^+$, 348.0511; found, 348.0508; deviation: 0.9 ppm.

5-Hydroxy-2-methoxybenzonitrile (13)

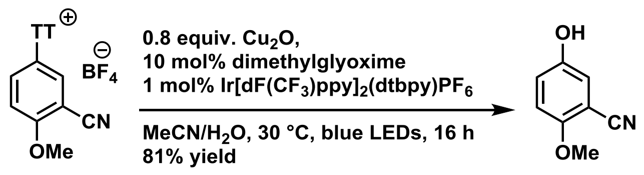

13-TT 13

To a 4-mL borosilicate vial, equipped with a magnetic stir bar was added copper(I) oxide (11.5 mg, 80.0 µmol, 0.800 equiv.), dimethylglyoxime (1.2 mg, 10 µmol, 0.10 equiv.), and $MeCN/H_2O$ (0.9 mL, v/v= 2:1). After stirring for 10 mins at ambient temperature, 2-methoxy-5-methylbenzonitrile thianthrenium salt **13-TT** (43.8 mg, 0.100 mmol, 1.00 equiv.), and $Ir[dF(CF_3)ppy]_2(dtbpy)PF_6$ (1.1 mg, 1.0 µmol, 1.0 mol%) in MeCN (0.40 mL, c = 0.25 M) were then added. The vial was evacuated and then filled with argon; this procedure was repeated three times. The vial was placed in 2 cm distance to two 34 W blue LEDs. The temperature was kept at approximately 30 °C with the use of a cooling fan. The reaction mixture was stirred for 16 hours under blue LED irradiation, and then diluted with CH_2Cl_2 (2 mL). The reaction mixture was filtered through a short pad of Celite using CH_2Cl_2 (20 mL) as eluent. The filtrate was collected and concentrated *in vacuo,* and the residue was then purified by flash column chromatography on silica gel, eluting with ethyl acetate/pentane (1:5, v/v) to afford **13** (12.1 mg, 81% yield) as a colorless solid.

R*f* = 0.35 (EtOAc/pentane, 1:2, v/v, (UV, cerium molybdate)).

NMR Spectroscopy:

^{1}H NMR (500 MHz, $CDCl_3$, 25 °C, δ): 7.13 – 7.03 (m, 2H), 6.85 (d, J = 9.9 Hz, 1H), 3.87 (s, 3H).

^{13}C {^{1}H} NMR (126 MHz, $CDCl_3$, 25 °C, δ): 155.8, 149.6, 122.1, 119.8, 116.4, 112.8, 101.8, 56.6.

HRMS-ESI(m/z) calc'd for $C_8H_7NO_2^+$ $[M]^+$, 149.0471; found, 149.0473; deviation: –1.1 ppm.

3-Chloropropyl benzene tetrafluorothianthrenium salt (14-TFT)

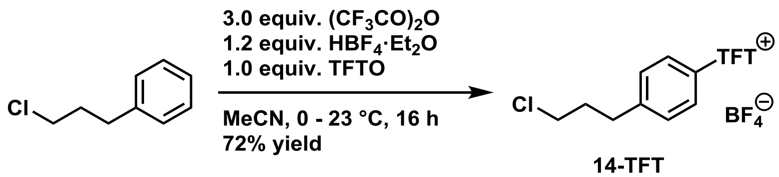

14-TFT

Under an ambient atmosphere, a 20-mL glass vial was charged with 3-chloropropyl benzene (154 mg, 1.00 mmol, 1.00 equiv) and MeCN (4.0 mL, c = 0.25 M). Trifluoroacetic anhydride (0.42 mL, 0.63 g, 3.0 mmol, 3.0 equiv.) was added while stirring the reaction mixture at 25 °C. After cooling to 0 °C, tetrafluorothianthren-*S*-oxide (304 mg, 1.00 mmol, 1.00 equiv.) was added in one portion, followed by the dropwise addition of

$HBF_4·OEt_2$ (163 µL, 194 mg, 1.20 mmol, 1.20 equiv.). The mixture was stirred at 0 °C for 2 hours, then warmed to 25 °C for 14 hours. The solution was diluted with CH_2Cl_2 (5 mL) and poured onto a mixture of CH_2Cl_2 (20 mL) and saturated aqueous $NaHCO_3$ solution (30 mL). After stirring for 5 min, the mixture was poured into a separating funnel, and the aqueous phase was extracted with DCM (2 × 20 mL). The DCM layer was washed with aqueous $NaBF_4$ solution (10% w/w, 2 × ca. 20 mL). The DCM layer was dried over Na_2SO_4, filtered, and the solvent was removed under reduced pressure. The residue was purified by chromatography on silica gel eluting with DCM/MeOH (30:1 (v/v)), then the solvent was removed *in vacuo* to afford **14-TFT** (378 mg, 72% yield) as a colorless powder.

R*f* = 0.35 (MeOH/DCM, 1/15, v/v).

NMR Spectroscopy:

^{1}H NMR (500 MHz, CD_3CN, 25 °C, δ): 8.41 (dd, *J* = 9.1, 7.1 Hz, 2H), 7.95 (dd, *J* = 10.0, 7.0 Hz, 2H), 7.38 (d, *J* = 8.5 Hz, 2H), 7.16 (d, *J* = 8.7 Hz, 2H), 3.53 (t, *J* = 6.5 Hz, 2H), 2.92 – 2.70 (m, 2H), 2.07 – 1.98 (m, 2H).

^{13}C {^{1}H} NMR (126 MHz, CD_3CN, 25 °C, δ) 154.4 (dd, *J* = 255.6, 13.7 Hz), 151.2 (dd, *J* = 255.8, 13.7 Hz), 148.5, 134.8 (dd, *J* = 8.8, 3.8 Hz), 131.3, 129.0, 125.1 (d, *J* = 24.2 Hz), 120.7 (d, *J* = 21.8 Hz), 120.3, 115.1 (dd, *J* = 7.7, 3.8 Hz), 44.8, 33.8, 32.6.

^{19}F NMR (471 MHz, CD_3CN, 25 °C, δ): –125.2 (m), –133.8 (m), –151.2 (brs), –151.3 (brs).

HRMS-ESI(m/z) calc'd for $C_{21}H_{14}ClF_4S_2^+$ $[M\text{-}BF_4]^+$, 441.0156; found, 441.0156; deviation: 0 ppm.

4-(3-Chloropropyl)phenol (14)

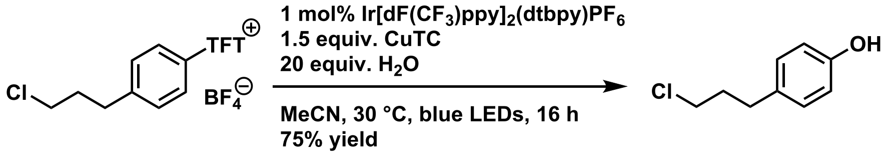

14-TFT **14**

To a 4-mL borosilicate vial, equipped with a magnetic stir bar was added $Ir[dF(CF_3)ppy]_2(dtbpy)PF_6$ (2.2 mg, 2.0 µmol, 1.0 mol%), copper(I) thiophene-2-carboxylate (57.2 mg, 0.300 mmol, 1.50 equiv.), and 3-chloropropyl benzene tetrafluorothianthrenium salt **14-TFT** (105 mg, 0.200 mmol, 1.00 equiv.) at 25 °C. The vial was evacuated and then filled with argon; this procedure was repeated three times. MeCN (1 mL, *c* = 0.2 M) was added, followed by H_2O (72.1 mg, 721 µL, 4.00 mmol, 20.0 equiv.). The vial was placed in 2 cm distance to two 34 W blue LEDs. The temperature was kept at approximately 30 °C with the use of a cooling fan. The reaction mixture was stirred for 16 hours under blue LED irradiation, and then diluted with ethyl acetate (1 mL). The reaction mixture was filtered through a short pad of silica using ethyl acetate (20 mL) as eluent. The filtrate was collected and concentrated *in vacuo,* and the residue was then purified by flash column chromatography on silica gel, eluting with ethyl acetate/pentane 1:10 (v/v) to afford **14** (25 mg, 75% yield) as a colorless oil.

R*f* = 0.40 (ethyl acetate/pentane, 1:5, v/v (UV, cerium molybdate))

NMR Spectroscopy:

^{1}H NMR (500 MHz, CD_3CN, 25 °C, δ): 7.04 (d, *J* = 8.5 Hz, 2H), 6.82 (s, 1H), 6.73 (d, *J* = 8.5 Hz, 2H), 3.55 (t, *J* = 6.6 Hz, 2H), 2.65 (t, *J* = 6.6 Hz, 2H), 2.03 – 1.96 (m, 2H).

^{13}C {^{1}H} NMR (126 MHz, CD_3CN, 25 °C, δ): 155.5, 132.5, 129.9, 115.5, 44.9, 34.7, 31.9.

HRMS-ESI(m/z) calc'd for $C_9H_{11}OCl^+$ [M]$^+$, 170.0493; found, 170.0497. Deviation: –2.2 ppm.

4-(3-Hydroxy-1-phenylpropyl)phenol (15)

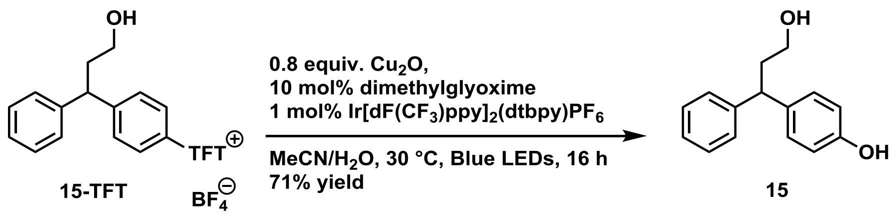

To a 4-mL borosilicate vial, equipped with a magnetic stir bar was added copper(I) oxide (11.5 mg, 80.0 µmol , 0.800 equiv.), dimethylglyoxime (1.2 mg, 10 µmol , 0.10 equiv.), and MeCN/H_2O (0.9 mL, v/v= 2:1). After stirring for 10 mins at ambient temperature, 3,3-diphenyl-propanol tetrafluorothianthrenium salt **15-TFT** (56.7 mg, 0.100 mmol, 1.00 equiv.), Ir[dF(CF_3)ppy]$_2$(dtbpy)PF_6 (1.1 mg, 1.0 µmol, 1.0 mol%), and MeCN (0.40 mL, *c* = 0.25 M) were then added. The vial was evacuated and then filled with argon; this procedure was repeated three times. The vial was placed in 2 cm distance to two 34 W blue LEDs. The temperature was kept at approximately 30 °C with the use of a cooling fan. The reaction mixture was stirred for 16 hours under blue LED irradiation, and then diluted with CH_2Cl_2 (2 mL). The reaction mixture was filtered through a short pad of Celite using CH_2Cl_2 (20 mL) as eluent. The filtrate was collected and concentrated *in vacuo,* and the residue was then purified by flash column chromatography on silica gel, eluting with ethyl acetate/pentane (1:5 to 1:2, v/v) to afford **15** (16.1 mg, 71% yield) as a colorless solid.

R*f* = 0.20 (EtOAc/pentane, 1:2, v/v (UV, cerium molybdate)).

NMR Spectroscopy:

^{1}H NMR (500 MHz, $CDCl_3$, 25 °C, δ) 7.32 – 7.21 (m, 4H), 7.20 – 7.15 (m, 2H), 7.11 (d, *J* = 8.5 Hz, 2H), 6.74 (d, *J* = 8.6 Hz, 2H), 4.07 (t, *J* = 7.9 Hz, 1H), 3.62 (t, *J* = 6.4 Hz, 2H), 2.28 (dt, *J* = 8.0, 6.4 Hz, 2H).

^{13}C {^{1}H} NMR (126 MHz, $CDCl_3$, 25 °C, δ): 154.1, 145.0, 136.8, 129.1, 128.7, 127.9, 126.4, 115.5, 61.4, 46.7, 38.5.

HRMS-ESI(m/z) calc'd for $C_{15}H_{16}O_2^+$ [M]$^+$, 228.1145; found, 228.1145; deviation: 0 ppm.

Hydroxy-salicin pentaacetate (16)

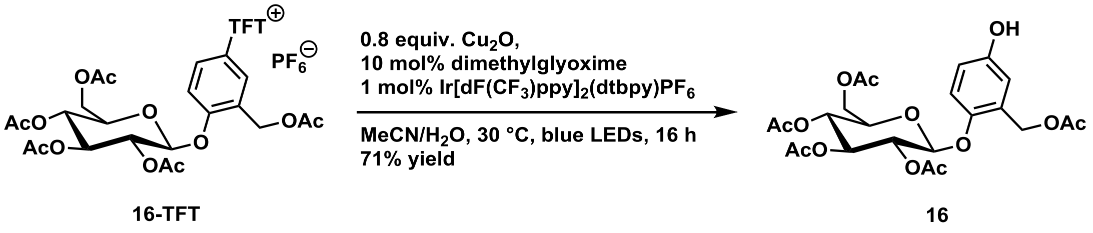

To a 4-mL borosilicate vial, equipped with a magnetic stir bar was added copper(I) oxide (11.5 mg, 80.0 µmol, 0.800 equiv.), dimethylglyoxime (1.2 mg, 10 µmol, 0.10 equiv.), and MeCN/H_2O (0.9 mL, v/v= 2:1). After stirring for 10 mins at ambient temperature, salicin pentaacetate tetrafluorothianthrenium salt **16-TFT** (92.8 mg, 0.100 mmol, 1.00 equiv.), and Ir[dF(CF_3)ppy]$_2$(dtbpy)PF_6 (1.1 mg, 1.0 µmol, 1.0 mol%) in MeCN (0.40 mL, *c* = 0.25 M) were then added. The vial was evacuated and then filled with argon; this procedure was repeated three times. The vial was placed in 2 cm distance to two 34 W blue LEDs. The temperature was kept at approximately 30 °C with the use of a cooling fan. The reaction mixture was stirred for 16 hours under blue LED irradiation, and then diluted with CH_2Cl_2 (2 mL). The reaction mixture was filtered through a short pad of Celite using CH_2Cl_2 (20 mL) as eluent. The filtrate was collected and concentrated *in vacuo,* and the residue was then purified by flash column chromatography on silica gel, eluting with ethyl acetate/pentane (1:3 to 1:1, v/v) to afford **16** (36.4 mg, 71% yield) as a pale yellow solid.

R*f* = 0.46 (EtOAc/pentane, 1:1, v/v (UV, cerium molybdate)).

NMR Spectroscopy:

^{1}H NMR (500 MHz, $CDCl_3$, 25 °C, δ): 6.98 (d, *J* = 8.8 Hz, 1H), 6.83 (d, *J* = 3.1 Hz, 1H), 6.72 (dd, *J* = 8.8, 3.1 Hz, 1H), 5.32 – 5.23 (m, 2H), 5.23 – 5.13 (m, 1H), 5.08 (d, *J* = 13.0 Hz, 1H), 5.00 (d, *J* = 13.0 Hz, 1H), 4.94 – 4.88 (m, 1H), 4.26 (dd, *J* = 12.3, 5.1 Hz, 1H), 4.18 (dd, *J* = 12.3, 2.6 Hz, 1H), 3.78 (ddd, *J* = 10.0, 5.1, 2.5 Hz, 1H), 2.12 – 2.00 (m, 15H).

^{13}C {^{1}H} NMR (126 MHz, $CDCl_3$, 25 °C, δ): 171.1, 170.9, 170.5, 169.6, 169.6, 152.2, 148.4, 128.1, 119.0, 116.1, 115.8, 100.7, 72.9, 72.1, 71.3, 68.5, 62.0, 61.1, 21.1, 20.9, 20.8, 20.7.

HRMS-ESI(m/z) calc'd for $C_{23}H_{28}O_{13}Na^+$ [M+Na]$^+$, 535.1422; found, 535.1423; deviation: –0.1 ppm.

Methyl 1-phenylcyclopropane-1-carboxylate tetrafluorothianthrenium salt (17-TFT)

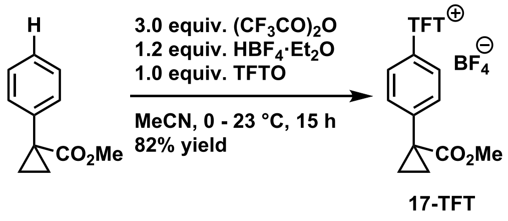

Under ambient atmosphere, a 20 mL round-bottom flask was charged with methyl 1-phenylcyclopropane-1-carboxylate (88.1 mg, 0.500 mmol, 1.00 equiv.), and dry MeCN (5.0 mL, *c* = 0.10 M). After cooling to 0 °C,

trifluoroacetic anhydride (209 µL, 315 mg, 1.50 mmol, 3.00 equiv.) was added to the reaction mixture. $HBF_4 \cdot OEt_2$ (82 µL, 0.60 mmol, 1.2 equiv.) was added at 0 °C in one portion, followed by tetrafluorothianthrene-*S*-oxide (152 mg, 0.500 mmol, 1.00 equiv.) addition in one portion at 0 °C. The vial was sealed with a screw-cap, and the mixture was allowed to stand at 0 °C for 1 hour and then warmed to 25 °C. After stirring the deep purple reaction mixture at 25 °C for 14 hours, the reaction mixture was concentrated under reduced pressure, and diluted with 30 mL CH_2Cl_2. The CH_2Cl_2 phase was poured onto a saturated aqueous $NaHCO_3$ solution (ca. 20 mL). The mixture was poured into a separatory funnel, and the layers were separated. The CH_2Cl_2 layer was washed with aqueous $NaBF_4$ solution (2 × ca. 20 mL, 5 % w/w), and with water (2 × ca. 20 mL). The CH_2Cl_2 layer was dried over Na_2SO_4, filtered, and the solvent was removed under reduced pressure. The residue was purified by chromatography on silica gel eluting with DCM/MeOH (30:1 (v/v)), then the solvent was removed *in vacuo* to afford **17-TFT** (203 mg, 82% yield) as a colorless powder.

R*f* = 0.35 (MeOH/DCM, 1/15, v/v).

NMR Spectroscopy:

^{1}H NMR (500 MHz, CD_3CN, 25 °C, δ): 8.48 (dd, *J* = 9.2, 7.2 Hz, 2H), 7.98 (dd, *J* = 10.0, 7.1 Hz, 2H), 7.52 (d, *J* = 8.8 Hz, 2H), 7.22 (d, *J* = 8.8 Hz, 2H), 3.53 (s, 3H), 1.54 (q, *J* = 4.2 Hz, 2H), 1.17 (q, *J* = 4.2 Hz, 2H).

^{13}C {^{1}H} NMR (126 MHz, CD_3CN, 25 °C, δ): 174.1, 154.6 (dd, *J* = 263.3, 13.6 Hz), 151.4 (dd, *J* = 255.6, 13.6 Hz), 146.6, 135.1 (dd, *J* = 8.5, 3.9 Hz), 133.5, 128.9, 125.5 (dd, *J* = 22.2, 2.3 Hz), 121.8, 120.9 (d, *J* = 21.9 Hz), 115.1 (dd, *J* = 7.3, 3.4 Hz), 52.8, 29.1, 17.1.

^{19}F NMR (471 MHz, CD_3CN, 25 °C, δ): –125.2 (ddd, *J* = 20.4, 9.9, 7.1 Hz), –133.6 (ddd, *J* = 20.8, 9.3, 7.1 Hz), –150.4 (brs), –150.5 (brs).

HRMS-ESI(m/z) calc'd for $C_{23}H_{15}F_4O_2S_2^+$ $[M-BF_4]^+$, 463.0444; found, 463.0435; deviation: 2.0 ppm.

Methyl 1-(4-hydroxyphenyl)cyclopropane-1-carboxylate (17)

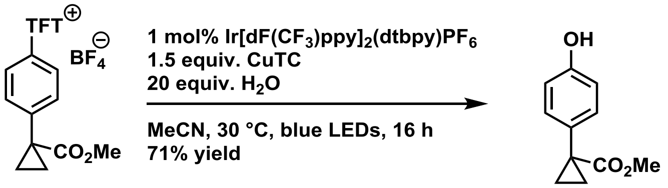

17-TFT **17**

To a 4-mL borosilicate vial, equipped with a magnetic stir bar was added $Ir[dF(CF_3)ppy]_2(dtbpy)PF_6$ (2.2 mg, 2.0 µmol, 1.0 mol%), copper(I) thiophene-2-carboxylate (57.2 mg, 0.300 mmol, 1.50 equiv.), and methyl 1-phenylcyclopropane-1-carboxylate tetrafluorothianthrenium salt **17-TFT** (110 mg, 0.200 mmol, 1.00 equiv.) at 25 °C. The vial was evacuated and then filled with argon; this procedure was repeated three times. MeCN (1 mL, *c* = 0.2 M) was added, followed by H_2O (72.1 mg, 721 µL, 4.00 mmol, 20.0 equiv.). The vial was placed in 2 cm distance to two 34 W blue LEDs. The temperature was kept at approximately 30 °C with the use of a cooling fan. The reaction mixture was stirred for 16 hours under blue LED irradiation, and then diluted with

ethyl acetate (1 mL). The reaction mixture was filtered through a short pad of silica using ethyl acetate (20 mL) as eluent. The filtrate was collected and concentrated *in vacuo,* and the residue was then purified by flash column chromatography on silica gel, eluting with ethyl acetate/pentane 1:8 (v/v) to afford **17** (27mg, 71% yield) as a colorless solid.

R*f* = 0.40 (ethyl acetate/pentane, 1:4, v/v)

NMR Spectroscopy:

^{1}H NMR (300 MHz, CD_3CN, 25 °C, δ): 7.17 (d, *J* = 8.7 Hz, 2H), 6.91 (s, 1H), 6.73 (d, *J* = 8.7 Hz, 2H), 3.55 (s, 3H), 1.48 (q, *J* = 3.9 Hz, 2H), 1.12 (q, J = 3.9 Hz, 2H).

^{13}C {^{1}H} NMR (126 MHz, CD_3CN, 25 °C , δ): 175.5, 156.5, 132.2, 131.7, 115.3, 52.2, 28.4, 16.7.

HRMS-ESI(m/z) calc'd for $C_{11}H_{12}O_3^+$ $[M]^+$, 192.0781; found, 192.0783. Deviation: –1.2 ppm.

6-Methyl-4-chromanone thianthrenium salt (18-TT)

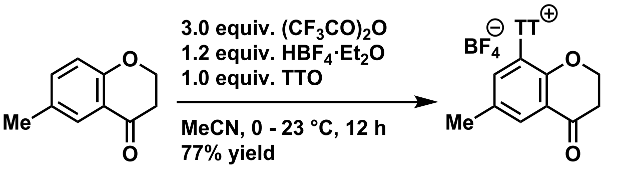

18-TT

Under an ambient atmosphere, a 20-mL glass vial was charged with 6-methyl-4-chromanone (294 mg, 1.50 mmol, 1.00 equiv) and MeCN (3.0 mL, *c* = 0.50 M). After cooling to 0 °C, $HBF_4·OEt_2$ (0.26 mL, 0.31 g, 1.8 mmol, 1.2 equiv) and thianthrene-*S*-oxide (348 mg, 1.50 mmol, 1.00 equiv) was added to the vial while stirring the mixture, leading to a suspension. Subsequently, trifluoroacetic anhydride (0.63 mL, 0.95 g, 4.5 mmol, 3.0 equiv) was added in one portion at 0 °C, resulting in a color change to deep purple. Subsequently, the reaction mixture was allowed to reach 23 °C and stirred for 12 hours. The solution was diluted with DCM (5 mL) and poured onto a mixture of DCM (30 mL) and saturated aqueous $NaHCO_3$ solution (20 mL). After stirring for 5 min at 23 °C, the mixture was poured into a separating funnel, and the layers were separated. The DCM layer was washed with aqueous $NaBF_4$ solution (10% w/w, 4 × ca. 20 mL). The DCM layer was dried over Na_2SO_4, filtered, and the solvent was removed under reduced pressure. The residue was purified by chromatography on silica gel eluting with DCM/MeOH (30:1 (v/v)), then the solvent was removed *in vacuo* to afford **18-TT** (530 mg, 77% yield) as a light yellow solid.

R*f* = 0.35 (DCM/MeOH, 15:1, v/v).

NMR Spectroscopy:

^{1}H NMR (500 MHz, $CDCl_3$, 25 °C, δ): 8.35 (dd, *J* = 7.9, 1.4 Hz, 2H), 7.84 (dd, *J* = 7.9, 1.5 Hz, 2H), 7.80 (s, 2H), 7.73 (dd, *J* = 7.9, 1.5 Hz, 3H), 6.53 (d, *J* = 2.1 Hz, 1H), 4.71 (t, *J* = 6.5 Hz, 2H), 2.75 (t, *J* = 6.5 Hz, 2H), 2.13 (s, 3H).

^{13}C {^{1}H} NMR (126 MHz, $CDCl_3$, 25 °C, δ): 189.0, 157.9, 137.1, 135.1, 135.1, 134.3, 133.0, 132.1, 130.5,

130.2, 128.5, 127.7, 123.1, 116.3, 109.4, 69.0, 37.2, 20.6.

^{19}F NMR (471 MHz, $CDCl_3$, 25 °C, δ):–151.8 (brs), –151.9 (brs).

HRMS-ESI(m/z) calc'd for $C_{22}H_{17}S_2O_2^+$ $[M-BF_4]^+$, 377.0665; found, 377.0664; deviation: 0.2 ppm.

8-Hydroxy-6-methylchroman-4-one (18)

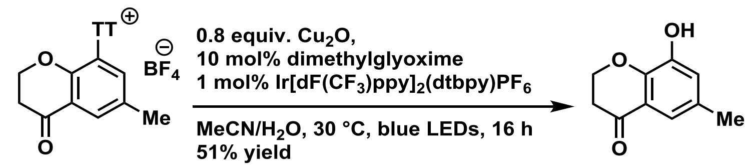

18-TT 18

To a 4-mL borosilicate vial, equipped with a magnetic stir bar was added copper(I) oxide (22.9 mg, 0.160 mmol, 0.800 equiv.), dimethylglyoxime (2.3 mg, 20 µmol, 0.10 equiv.), and $MeCN/H_2O$ (1.6 mL, v/v= 5:3). After stirring for 10 mins at ambient temperature, 6-methyl-4-chromanone thianthrenium salt **18-TT** (92.9 mg, 0.200 mmol, 1.00 equiv.), and $Ir[dF(CF_3)ppy]_2(dtbpy)PF_6$ (2.2 mg, 2.0 µmol, 1.0 mol%) in MeCN (0.40 mL, *c* = 0.50 M) were then added. The vial was evacuated and then filled with argon; this procedure was repeated three times. The vial was placed in 2 cm distance to two 34 W blue LEDs. The temperature was kept at approximately 30 °C with the use of a cooling fan. The reaction mixture was stirred for 16 hours under blue LED irradiation, and then diluted with CH_2Cl_2 (2 mL). The reaction mixture was filtered through a short pad of Celite using CH_2Cl_2 (20 mL) as eluent. The filtrate was collected and concentrated *in vacuo,* and the residue was then purified by flash column chromatography on silica gel, eluting with ethyl acetate/pentane (1:10, v/v) to afford **18** (26.9 mg, 51% yield) as a colorless solid.

R*f* = 0.28 (EtOAc/pentane, 1:2, v/v (UV, cerium molybdate)).

NMR Spectroscopy:

^{1}H NMR (500 MHz, CD_3CN, 25 °C, δ): 7.12 (dd, *J* = 2.1, 1.1 Hz, 1H), 6.90 (d, *J* = 2.1 Hz, 1H), 6.70 (brs, 1H, OH), 4.61 – 4.35 (m, 3H), 2.83 – 2.68 (m, 2H), 2.16 (s, 3H).

^{13}C {^{1}H} NMR (126 MHz, CD_3CN, 25 °C, δ): 192.6, 149.1, 146.6, 131.7, 122.5, 118.3, 117.5, 68.5, 38.5, 20.7.

HRMS-ESI(m/z) calc'd for $C_{10}H_{11}O_3^+$ $[M+H]^+$, 179.0703; found, 179.0705; deviation: –1.2 ppm.

Benzyl benzoate tetrafluorothianthrenium salt (19-TFT)

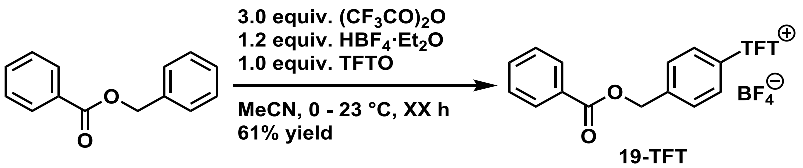

19-TFT

Under an ambient atmosphere, a 20-mL glass vial was charged with benzyl benzoate (636 mg, 3.00 mmol,

1.00 equiv) and MeCN (5.0 mL, *c* = 0.60 M). After cooling to 0 °C, $HBF_4 \cdot OEt_2$ (0.50 mL, 0.59 g, 3.6 mmol, 1.2 equiv) and tetrafluorothianthrene-*S*-oxide (912 mg, 3.00 mmol, 1.00 equiv) was added to the vial while stirring the mixture, leading to a suspension. Subsequently, trifluoroacetic anhydride (1.3 mL, 1.8 g, 9.0 mmol, 3.0 equiv) was added in one portion at 0 °C, resulting in a color change to deep purple. Subsequently, the reaction mixture was allowed to reach 23 ^{o}C and stirred for 12 hours. The solution was diluted with DCM (5 mL) and poured onto a mixture of DCM (30 mL) and saturated aqueous $NaHCO_3$ solution (20 mL). After stirring for 5 min at 23 ^{o}C, the mixture was poured into a separating funnel, and the layers were separated. The DCM layer was washed with aqueous $NaBF_4$ solution (10% w/w, 4 × ca. 20 mL). The DCM layer was dried over Na_2SO_4, filtered, and the solvent was removed under reduced pressure. The residue was purified by chromatography on silica gel eluting with DCM/MeOH (30:1 (v/v)), then the solvent was removed *in vacuo* to afford **19-TFT** (1.05 g, 61% yield) as a colorless solid.

R*f* = 0.35 (DCM/MeOH, 15:1, v/v).

NMR Spectroscopy:

^{1}H NMR (500 MHz, CD_3CN, 25 °C, δ): 8.48-8.45 (m, 2H), 7.98-7.93 (m, 4H), 7.62-7.58 (m, 3H), 7.63 (t, *J* = 8.0 Hz, 3H), 7.26 (d, *J* = 8.5 Hz, 2H), 5.36 (s, 2H).

^{13}C {^{1}H} NMR (126 MHz, CD_3CN, 25 °C, δ): 166.8, 154.9 (dd, *J* = 262.1, 13.9 Hz), 151.6 (dd, *J* = 254.5, 12.6 Hz), 143.6, 135.3 (dd, *J* = 8.8, 5.0 Hz), 134.5, 130.6, 130.4, 130.3, 129.7, 129.6, 125.7 (dd, *J* = 22.7, 2.5 Hz), 123.1, 121.2 (d, *J* = 21.4 Hz), 115.2 (dd, *J* = 7.4, 3.8 Hz), 65.9.

^{19}F NMR (471 MHz, CD_3CN, 25 °C, δ): –125.2 (ddd, *J* = 20.8, 10.2, 7.3 Hz), –133.6 (ddd, *J* = 20.8, 9.2, 7.2 Hz), –150.7 (brs), –150.8 (brs).

HRMS-ESI(m/z) calc'd for $C_{26}H_{15}F_4O_2S_2^{+}$ [M-BF_4]$^{+}$, 499.0444; found, 499.0442; deviation: 0.4 ppm.

4-Hydroxybenzyl benzoate (19)

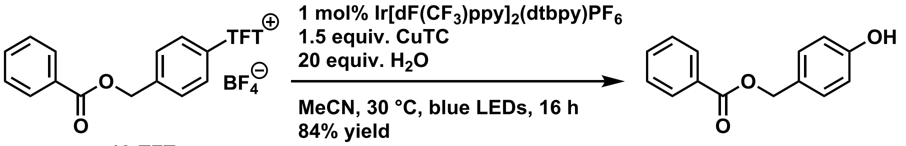

19-TFT 19

To a 4-mL borosilicate vial, equipped with a magnetic stir bar was added Ir[dF(CF_3)ppy]$_2$(dtbpy)PF_6 (2.2 mg, 2.0 µmol, 1.0 mol%), copper(I) thiophene-2-carboxylate (57.2 mg, 0.300 mmol, 1.50 equiv.), and benzyl benzoate tetrafluorothianthrenium salt **19-TFT** (117 mg, 0.200 mmol, 1.00 equiv.) at 25 °C. The vial was evacuated and then filled with argon; this procedure was repeated three times. MeCN (1 mL, *c* = 0.2 M) was added, followed by H_2O (72.1 mg, 721 µL, 4.00 mmol, 20.0 equiv.). The vial was placed in 2 cm distance to two 34 W blue LEDs. The temperature was kept at approximately 30 °C with the use of a cooling fan. The reaction mixture was stirred for 16 hours under blue LED irradiation, and then diluted with ethyl acetate (1 mL). The reaction mixture was filtered through a short pad of silica using ethyl acetate (20 mL) as eluent. The filtrate was collected and concentrated *in vacuo,* and the residue was then purified by flash column

chromatography on silica gel, eluting with ethyl acetate/pentane 1:8 (v/v) to afford **19** (38 mg, 84% yield) as a colorless solid.

R*f* = 0.40 (ethyl acetate/pentane, 1:4, v/v (UV))

NMR Spectroscopy:

^{1}H NMR (500 MHz, CD_3CN, 25 °C, δ): 8.00 (dd, *J* = 8.4 Hz, 1.4 Hz, 2H), 7.61 (t, *J* = 7.4 Hz, 1H), 7.48 (dd, *J* = 8.6 Hz, 7.1 Hz, 2H), 7.32 (d, *J* = 8.5 Hz, 2H), 7.05 (s, 1H), 6.83 (d, *J* = 8.6 Hz, 2H), 5.24 (s, 2H).

^{13}C {^{1}H} NMR (126 MHz, CD_3CN, 25 °C, δ): 166.9, 157.8, 133.8, 131.1, 130.9, 130.0, 129.3, 128.4, 116.0, 67.1.

HRMS-ESI(m/z) calc'd for $C_{14}H_{12}O_3^+$ $[M]^+$, 228.0781; found, 228.0778. Deviation: 1.4 ppm.

N-benzyloxycarbonyl-4-*cis*-phenoxy-L-proline methyl ester tetrafluorothianthrenium salt (20-TFT)

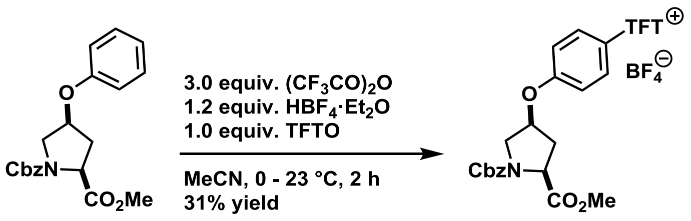

20-TFT

Under an ambient atmosphere, a 20-mL glass vial was charged with N-benzyloxycarbonyl-4-*trans*-phenoxy-L-proline methyl ester (178 mg, 0.500 mmol, 1.00 equiv) and MeCN (5 ml, *c* = 0.1 M). Trifluoroacetic anhydride (208 µL, 315 mg, 1.50 mmol, 3.00 equiv.) was added while stirring the reaction mixture at 25 °C. After cooling to 0 °C, tetrafluorothianthrene-*S*-oxide (152 mg, 0.500 mmol, 1.00 equiv.) was added in one portion, followed by the dropwise addition of $HBF_4·OEt_2$ (82 µL, 0.60 mmol, 1.2 equiv.). The mixture was stirred at 0 °C for 1 hour, then warmed to 25 °C for 1 hour. The solution was diluted with CH_2Cl_2 (5 mL) and poured onto a mixture of CH_2Cl_2 (20 mL) and saturated aqueous $NaHCO_3$ solution (30 mL). After stirring for 5 min, the mixture was poured into a separating funnel, and the aqueous phase was extracted with DCM (2 × 20 mL). The DCM layer was washed with aqueous $NaBF_4$ solution (10% w/w, 2 × ca. 20 mL). The DCM layer was dried over Na_2SO_4, filtered, and the solvent was removed under reduced pressure. The residue was purified by chromatography on silica gel eluting with DCM/MeOH (30:1 (v/v)), then the solvent was removed *in vacuo* to afford **20-TFT** (112 mg, 31% yield) as a light yellow powder.

R*f* = 0.35 (MeOH/DCM, 1/15, v/v).

NMR Spectroscopy:

^{1}H NMR (500 MHz, CD_2Cl_2, 25 °C, δ): 8.40 – 8.23 (m, 2H), 7.70 – 7.62 (m, 2H), 7.30 – 7.17 (m, 7H), 6.90 – 6.82 (m, 2H), 5.10 – 4.84 (m, 3H), 4.48 (ddd, *J* = 15.7, 8.7, 2.4 Hz, 1H), 3.73 (ddd, *J* = 12.5, 10.4, 4.8 Hz, 1H), 3.64 – 3.50 (m, 4H), 2.49 – 2.34 (m, 2H).

^{13}C {^{1}H} NMR (126 MHz, CD_2Cl_2, 25 °C, δ): 172.4, 172.2, 161.7, 154.9, 154.7, 154.3 (dd, *J* = 261.6, 13.2 Hz), 151.3 (dd, *J* = 259.3, 13.5 Hz), 137.2, 137.1, 134.1 (dd, *J* = 8.6, 3.8 Hz),131.4, 131.3, 129.0, 128.9, 128.6, 128.5, 128.3, 128.1, 124.5 (d, *J* = 21.9 Hz), 120.3 (d, *J* = 21.2 Hz), 118.5, 118.4, 115.6 (dd, *J* = 7.4, 3.6 Hz), 113.0, 77.2, 76.2, 67.6, 67.5, 58.5, 58.2, 52.7, 52.7, 52.5, 52.2, 36.6, 35.6.

^{19}F NMR (471 MHz, CD_2Cl_2, 25 °C, δ): –120.8 (m), –129.2 (m), –148.9 (brs), –149.0 (brs).

HRMS-ESI(m/z) calc'd for $C_{24}H_{18}F_4NO_5S_2^+$ $[M-BF_4]^+$, 642.1027, found, 642.1021, deviation: 0.9 ppm.

Hydroxy-N-benzyloxycarbonyl-4-*cis*-phenoxy-L-proline methyl ester (20)

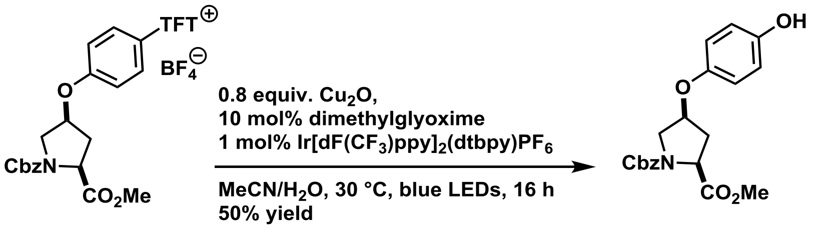

20-TFT **20**

To a 4-mL borosilicate vial, equipped with a magnetic stir bar was added copper(I) oxide (11.5 mg, 80.0 µmol , 0.800 equiv.), dimethylglyoxime (1.2 mg, 10 µmol , 0.10 equiv.), and MeCN/H_2O (0.9 mL, v/v= 2:1). After stirring for 10 mins at ambient temperature, N-benzyloxycarbonyl-4-*trans*-phenoxy-L-proline methyl ester tetrafluorothianthrenium salt **20-TFT** (72.9 mg, 0.100 mmol, 1.00 equiv.), and Ir[dF(CF_3)ppy]$_2$(dtbpy)PF_6 (1.1 mg, 1.0 µmol, 1.0 mol%) in MeCN (0.40 mL, *c* = 0.25 M) were then added The vial was evacuated and then filled with argon; this procedure was repeated three times. The vial was placed in 2 cm distance to two 34 W blue LEDs. The temperature was kept at approximately 30 °C with the use of a cooling fan. The reaction mixture was stirred for 16 hours under blue LED irradiation,, and then diluted with CH_2Cl_2 (2 mL). The reaction mixture was filtered through a short pad of Celite using CH_2Cl_2 (20 mL) as eluent. The filtrate was collected and concentrated *in vacuo,* and the residue was then purified by flash column chromatography on silica gel, eluting with ethyl acetate/ pentane (1:10 to 1:4, v/v) to afford **20** (18.4 mg, 50% yield) as a colorless solid.

R*f* = 0.29 (EtOAc/pentane, 1:2, v/v (UV, cerium molybdate)).

NMR Spectroscopy:

^{1}H NMR (500 MHz, CD_3CN, 25 °C, δ): 7.42 – 7.29 (m, 5H), 6.70 (qd, *J* = 9.2, 2.4 Hz, 4H), 6.61 (bs, 1H, OH), 5.18 – 4.97 (m, 2H), 4.92 – 4.80 (m, 1H), 4.49 (ddd, *J* = 9.7, 8.1, 2.0 Hz, 1H), 3.77 – 3.52 (m, 5H), 2.45 (m, 1H), 2.37 – 2.28 (m, 1H).

^{13}C {^{1}H} NMR (126 MHz, CD_3CN, 25 °C, δ): 173.2, 172.9, 155.5, 155.2, 152.4, 150.8, 138.1, 138.0, 134.3, 133.7, 129.4, 129.3, 128.9, 128.8, 128.6, 128.5, 118.1, 116.8, 77.3, 76.2, 67.5, 58.9, 58.6, 53.0, 52.7, 52.7, 36.7, 35.8.

HRMS-ESI(m/z) calc'd for $C_{20}H_{21}NO_6Na_1^+$ $[M+Na]^+$, 394.1261, found, 394.1263, deviation: –0.4 ppm.

Hydroxy-etofenprox (21)

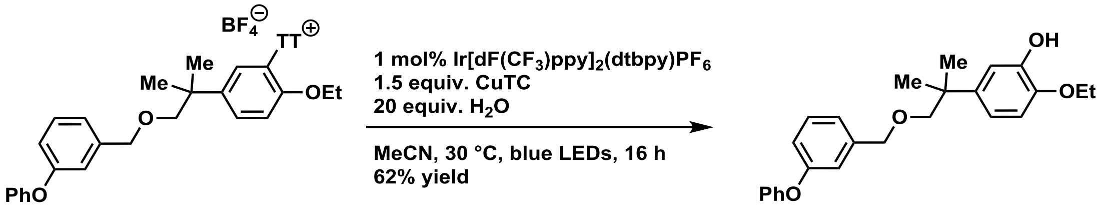

To a 4-mL borosilicate vial, equipped with a magnetic stir bar was added copper(I) thiophene-2-carboxylate (28.6 mg, 0.150 mmol, 1.50 equiv.), etofenprox thianthrenium salt **21-TT** (67.8 mg, 0.100 mmol, 1.00 equiv.), Ir[dF(CF_3)ppy]$_2$(dtbpy)PF_6 (1.1 mg, 1.0 µmol, 1.0 mol%), water (36 µL, 18 mg, 2.0 mmol, 20 equiv.), and MeCN (0.5 mL, *c* = 0.2 M). The vial was evacuated and then filled with argon; this procedure was repeated three times. The vial was placed in 2 cm distance to two 34 W blue LEDs. The temperature was kept at approximately 30 °C with the use of a cooling fan. The reaction mixture was stirred for 16 hours under blue LED irradiation, and then diluted with CH_2Cl_2 (2 mL). The reaction mixture was filtered through a short pad of Celite using CH_2Cl_2 (20 mL) as eluent. The filtrate was collected and concentrated *in vacuo,* and the residue was then purified by flash column chromatography on silica gel, eluting with ethyl acetate/pentane (1:20, v/v) to afford **21** (24.2 mg, 62% yield) as a colorless solid.

R*f* = 0.36 (EtOAc/pentane, 1:20, v/v (UV, cerium molybdate)).

NMR Spectroscopy:

^{1}H NMR (500 MHz, $CDCl_3$, 25 °C, δ): 7.40 – 7.23 (m, 3H), 7.11 (ddt, *J* = 7.7, 6.9, 1.1 Hz, 1H), 7.07 –6.87 (m, 6H), 6.84 – 6.69 (m, 2H), 5.61 (brs, 1H, OH), 4.45 (s, 2H), 4.08 (q, *J* = 7.0 Hz, 2H), 3.40 (s, 2H), 1.42 (t, *J* = 7.0 Hz, 3H), 1.29 (s, 6H).

^{13}C {^{1}H} NMR (126 MHz, $CDCl_3$, 25 °C, δ): 157.3, 157.2, 145.2, 143.9, 141.0, 140.9, 129.7, 129.5, 123.2, 122.0, 120.0, 117.7, 117.6, 117.5, 112.7, 111.1, 80.2, 72.8, 64.5, 38.7, 26.1, 15.0.

HRMS-ESI(m/z) calc'd for $C_{25}H_{28}O_4Na^+$ [M+Na]$^+$, 415.1880; found, 415.1877; deviation: 0.8 ppm.

Biphenyl tetrafluorothianthrenium salt (22-TFT)

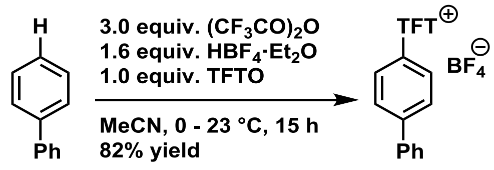

Under an ambient atmosphere, a 50-ml glass vial was charged with biphenyl (0.77 g, 5.0 mmol, 1.0 equiv) and MeCN (20 ml, *c* = 0.25 M). Trifluoroacetic anhydride (2.08 mL, 3.15 g, 15.0 mmol, 3.00 equiv.) was added at 25 °C while stirring the reaction mixture. After cooling to 0 °C, tetrafluorothianthrene-*S*-oxide (1.52 g, 5.00 mmol, 1.00 equiv.) was added in one portion, followed by the dropwise addition of HBF_4·OEt_2 (1.08 mL,

1.29 g, 8.00 mmol, 1.60 equiv.). The mixture was stirred at 0 °C for 1 hour, then warmed to 25 °C for 14 hours. The solution was diluted with CH_2Cl_2 (5 mL) and poured onto a mixture of CH_2Cl_2 (20 mL) and saturated aqueous $NaHCO_3$ solution (30 mL). After stirring for 5 min, the mixture was poured into a separating funnel, and the aqueous phase was extracted with DCM (2 × 20 mL). The DCM layer was washed with aqueous $NaBF_4$ solution (10% w/w, 2 × ca. 20 mL). The DCM layer was dried over Na_2SO_4, filtered, and the solvent was removed under reduced pressure. The residue was purified by chromatography on silica gel eluting with DCM/MeOH (30:1 (v/v)), then the solvent was removed *in vacuo* to afford **22-TFT** (1.88g, 82% yield) as a colorless powder.

R*f* = 0.35 (MeOH/DCM, 1/15, v/v).

NMR Spectroscopy:

^{1}H NMR (300 MHz, CD_3CN, 25 °C, δ): 8.47 (dd, *J* = 9.1, 7.2 Hz, 2H), 7.97 (dd, *J* = 9.9, 7.0 Hz, 2H), 7.80 – 7.71 (m, 2H), 7.64 – 7.58 (m, 2H), 7.52 – 7.40 (m, 3H), 7.34 – 7.24 (m, 2H).

^{13}C {^{1}H} NMR (75 MHz, CD_3CN, 25 °C, δ): 154.7 (dd, *J* = 260.2, 13.5 Hz), 151.5 (dd, *J* = 254.3, 13.6 Hz), 146.8, 138.8, 135.2 (dd, *J* = 8.6, 4.0 Hz), 130.1, 130.0, 129.8, 129.7, 128.2, 125.5 (dd, *J* = 22.2, 2.6 Hz), 121.9, 121.1 (d, *J* = 21.8 Hz), 115.3 (dd, *J* = 7.3, 3.6 Hz).

^{19}F NMR (471 MHz, CD_3CN, 25 °C, δ): –125.3 (m), –133.7 (m), –150.6 (brs), –150.7 (brs).

HRMS-ESI (m/z) calculated for $C_{22}H_{15}F_4O_2S_2^+$ $[M-BF_4]^+$, 441.0389; found, 441.0382; deviation: 1.7 ppm.

Biphenyl thianthrenium salt (22-TT)

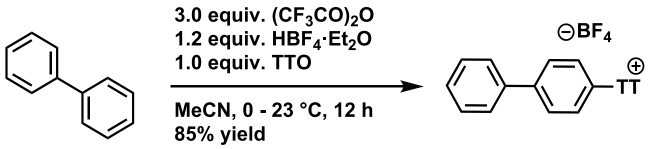

22-TT

Under an ambient atmosphere, a 20-mL glass vial was charged with 1,1'-biphenyl (720mg, 5.00 mmol, 1.00 equiv) and MeCN (5.0 mL, *c* = 1.0 M). After cooling to 0 °C, $HBF_4 \cdot OEt_2$ (0.82 mL, 0.97 g, 6.0 mmol, 1.2 equiv) and thianthrene-*S*-oxide (1.16 g, 5.00 mmol, 1.00 equiv) was added to the vial while stirring the mixture, leading to a suspension. Subsequently, trifluoroacetic anhydride (2.1 mL, 3.1 g, 15 mmol, 3.0 equiv) was added in one portion at 0 °C, resulting in a color change to deep purple. Subsequently, the reaction mixture was allowed to reach 23 ^{o}C and stirred for 12 hours. The solution was diluted with DCM (5 mL) and poured onto a mixture of DCM (30 mL) and saturated aqueous $NaHCO_3$ solution (20 mL). After stirring for 5 min at 23 ^{o}C, the mixture was poured into a separating funnel, and the layers were separated. The DCM layer was washed with aqueous $NaBF_4$ solution (10% w/w, 4 × ca. 20 mL). The DCM layer was dried over Na_2SO_4, filtered, and the solvent was removed under reduced pressure. The residue was purified by chromatography on silica gel eluting with DCM/MeOH (30:1 (v/v)), then the solvent was removed *in vacuo* to afford **22-TT** (1.94 g, 85% yield) as a colorless powder.

R$_f$ = 0.35 (DCM/MeOH, 15:1, v/v).

NMR Spectroscopy:

^{1}H NMR (500 MHz, DMSO-d_6, 25 °C, δ): 8.65 (dd, J = 7.9, 1.3 Hz, 2H), 8.13 (dd, J = 7.9, 1.3 Hz, 2H), 7.99 (td, J = 7.7, 1.4 Hz, 2H), 7.94 – 7.88 (m, 4H), 7.70 – 7.68 (m, 2H), 7.53 – 7.50 (m, 2H), 7.48 – 7.47 (m, 1H), 7.34 – 7.33 (m, 2H).

^{13}C NMR (126 MHz, CD_3CN, 25 °C, δ): 144.7, 137.1, 135.8, 134.3, 134.2, 129.9, 129.1, 128.3, 128.1, 128.0, 127.7, 126.4, 121.5, 117.7.

^{19}F NMR (471 MHz, CD_3CN, 25 °C, δ): –152.4 (brs), –152.5 (brs).

HRMS-ESI (m/z) calculated for $C_{24}H_{17}S_2^+$ $[M\text{-}BF_4]^+$, 369.0766; found, 369.0767; deviation: –0.3 ppm.

4-Phenylphenol (22)

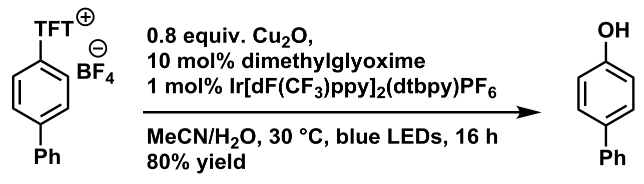

22-TFT 22

To a 4-mL borosilicate vial, equipped with a magnetic stir bar was added copper(I) oxide (22.9 mg, 0.160 mmol, 0.800 equiv.), dimethylglyoxime (2.3 mg, 20 µmol, 0.10 equiv.), and MeCN/H_2O (1.6 mL, v/v= 3:2). After stirring for 10 mins at ambient temperature, biphenyl tetrafluorothianthrenium salt **22-TFT** (107 mg, 0.200 mmol, 1.00 equiv.), and Ir[dF(CF_3)ppy]$_2$(dtbpy)PF_6 (2.2 mg, 2.0 µmol, 1.0 mol%) in MeCN (0.40 mL, c= 0.50 M) were then added. The vial was evacuated and then filled with argon; this procedure was repeated three times. The vial was placed in 2 cm distance to two 34 W blue LEDs. The temperature was kept at approximately 30 °C with the use of a cooling fan. The reaction mixture was stirred for 16 hours under blue LED irradiation, and then diluted with CH_2Cl_2 (2 mL). The reaction mixture was filtered through a short pad of Celite using CH_2Cl_2 (20 mL) as eluent. The filtrate was collected and concentrated *in vacuo,* and the residue was then purified by flash column chromatography on silica gel, eluting with ethyl acetate/pentane (1:20, v/v) to afford **22** (27.1 mg, 80% yield) as a colorless solid.

R$_f$ = 0.25 (EtOAc/pentane, 1:20, v/v).

NMR Spectroscopy:

^{1}H NMR (500 MHz, CD_3CN, 25 °C, δ): 7.66 – 7.55 (m, 2H), 7.52 – 7.48 (m, 2H), 7.41 (dd, J = 8.6, 7.0 Hz, 2H), 7.34 – 7.26 (m, 1H), 7.09 (brs, 1H, OH), 7.01 – 6.87 (m, 2H).

^{13}C {^{1}H} NMR (126 MHz, CD_3CN, 25 °C, δ): 157.6, 141.6, 133.5, 129.7, 129.0, 127.5, 127.3, 116.6.

HRMS-ESI(m/z) calc'd for $C_{12}H_9O^+$ $[M\text{-}H]^+$, 169.0659; found, 169.0660; deviation: –0.5 ppm.

(3r,5r,7r)-1-(p-Tolyl)adamantane tetrafluorothianthrenium salt (23-TFT)

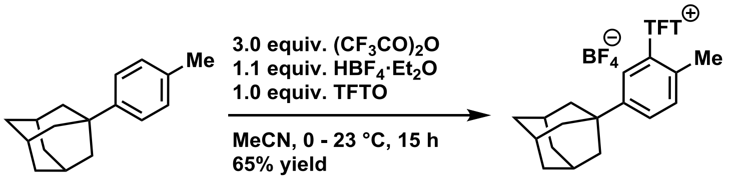

23-TFT

Under an ambient atmosphere, a 20-mL glass vial was charged with p-(1-Adamantyl)toluene (452 mg, 2.00 mmol, 1.00 equiv) and MeCN (8.0 mL, *c* = 0.25 M). Trifluoroacetic anhydride (834 µL, 1.26 g, 6.00 mmol, 3.00 equiv.) was added while stirring the reaction mixture at 25 °C. After cooling to 0 °C, tetrafluorothianthrene-*S*-oxide (608 mg, 2.00 mmol, 1.00 equiv.) was added in one portion, followed by the dropwise addition of $HBF_4 \cdot OEt_2$ (299 µL, 356 mg, 2.20 mmol, 1.10 equiv.). The mixture was stirred at 0 °C for 2 hours, then warmed to 25 °C for 14 hours. The solution was diluted with CH_2Cl_2 (5 mL) and poured onto a mixture of CH_2Cl_2 (20 mL) and saturated aqueous $NaHCO_3$ solution (30 mL). After stirring for 5 min, the mixture was poured into a separating funnel, and the aqueous phase was extracted with DCM (2 × 20 mL). The DCM layer was washed with aqueous $NaBF_4$ solution (10% w/w, 2 × ca. 20 mL). The DCM layer was dried over Na_2SO_4, filtered, and the solvent was removed under reduced pressure. The residue was purified by chromatography on silica gel eluting with DCM/MeOH (30:1 (v/v)), then the solvent was removed *in vacuo* to afford **23-TFT** (780 mg, 65% yield) as a colorless powder.

R*f* = 0.35 (MeOH/DCM, 1/15, v/v).

NMR Spectroscopy:

^{1}H NMR (500 MHz, CD_3CN, 25 °C, δ): 8.25 (dd, *J* = 9.2, 7.1 Hz, 2H), 8.02 (dd, *J* = 9.9, 7.0 Hz, 2H), 7.67 (dd, *J* = 8.0, 1.9 Hz, 1H), 7.50 (d, *J* = 8.1 Hz, 1H), 6.96 (d, *J* = 1.9 Hz, 1H), 2.66 (s, 3H), 2.00 (q, *J* = 3.2 Hz, 3H), 1.76 – 1.63 (m, 12H).

^{13}C {^{1}H} NMR (126 MHz, CD_3CN, 25 °C, δ): 154.4 (dd, *J* = 261.7, 13.0 Hz), 152.5, 151.8 (dd, *J* = 14.5, 1.0 Hz), 138.8, 135.2 (dd, *J* = 8.3, 3.9 Hz), 134.8, 132.7, 127.0, 124.4 (dd, *J* = 22.1, 2.3 Hz), 121.0 (d, *J* = 21.7 Hz), 120.6, 115.4 (dd, *J* = 7.0, 3.6 Hz), 43.0, 37.0, 36.8, 3.54, 20.1.

^{19}F NMR (471 MHz, CD_3CN, 25 °C, δ): –125.7 (dd, *J* = 10.0, 4.7 Hz), –133.2 (m), –150.7 (brs), –150.8 (brs).

HRMS-ESI(m/z) calc'd for $C_{29}H_{25}F_4S_2^+$ $[M-BF_4]^+$, 513.1328; found, 513.1327. Deviation: –1.1 ppm.

5-((3r,5r,7r)-Adamantan-1-yl)-2-methylphenol (23)

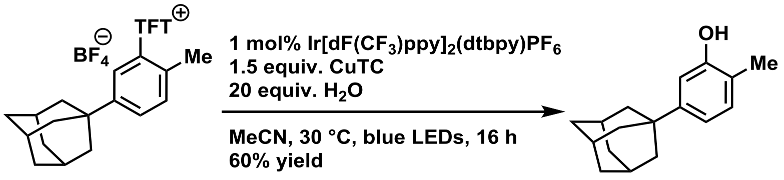

23-TFT **23**

To a 4-mL borosilicate vial, equipped with a magnetic stir bar was added Ir[dF(CF_3)ppy]$_2$(dtbpy)PF_6 (2.2 mg, 2.0 µmol, 1.0 mol%), copper(I) thiophene-2-carboxylate (57.2 mg, 0.300 mmol, 1.50 equiv.), and (3r,5r,7r)-1-(p-tolyl)adamantane tetrafluorothianthrenium salt **23-TFT** (120 mg, 0.200 mmol, 1.00 equiv.) at 25 °C. The vial was evacuated and then filled with argon; this procedure was repeated three times. MeCN (1 mL, *c* = 0.2 M) was added, followed by H_2O (72.1 mg, 721 µL, 4.00 mmol, 20.0 equiv.). The vial was placed in 2 cm distance to two 34 W blue LEDs. The temperature was kept at approximately 30 °C with the use of a cooling fan. The reaction mixture was stirred for 16 hours under blue LEDs, and then diluted with ethyl acetate (1 mL). The reaction mixture was filtered through a short pad of silica using ethyl acetate (20 mL) as eluent. The filtrate was collected and concentrated *in vacuo,* and the residue was then purified by flash column chromatography on silica gel, eluting with ethyl acetate/pentane 1:20 (v/v) to afford **23** (29.1mg, 60% yield) as a colorless solid.

R*f* = 0.40 (ethyl acetate/pentane, 1:10, v/v (UV, cerium molybdate))

NMR Spectroscopy:

^{1}H NMR (300 MHz, CD_3CN, 25 °C, δ): 7.08 – 6.91 (m, 1H), 6.79 – 6.76 (m, 2H), 6.52 (s, 1H), 2.12 (d, *J* = 0.7 Hz, 3H), 2.07 – 2.03 (m, 3H), 1.86 (d, *J* = 2.9 Hz, 6H), 1.84 – 1.71 (m, 6H).

^{13}C {^{1}H} NMR (75 MHz, CD_3CN, 25 °C, δ): 155.1, 151.2, 130.9, 121.6, 116.8, 112.0, 43.5, 37.0, 36.2, 29.6, 15.2.

HRMS-ESI(m/z) calc'd for $C_{17}H_{22}O^+$ $[M]^+$, 242.1665; found, 242.1668. Deviation: –1.1 ppm.

4'-Hydroxy-[1,1'-biphenyl]-4-yl trifluoromethanesulfonate (24)

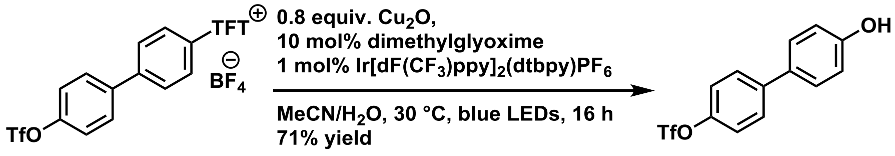

24-TFT **24**

To a 4-mL borosilicate vial, equipped with a magnetic stir bar was added copper(I) oxide (22.9 mg, 0.160 mmol, 0.800 equiv.), dimethylglyoxime (2.3 mg, 20 µmol, 0.10 equiv.), and MeCN/H_2O (1.6 mL, v/v= 5:3). After stirring for 10 mins at ambient temperature, 4'-hydroxy-[1,1'-biphenyl]-4-yl trifluoromethanesulfonate tetrafluorothianthrenium salt **24-TFT** (135 mg, 0.200 mmol, 1.00 equiv.), and Ir[dF(CF_3)ppy]$_2$(dtbpy)PF_6 (2.2 mg, 2.0 µmol, 1.0 mol%) in MeCN (0.40 mL, *c* = 0.50 M) were then added. The vial was evacuated and then

filled with argon; this procedure was repeated three times. The vial was placed in 2 cm distance to two 34 W blue LEDs. The temperature was kept at approximately 30 °C with the use of a cooling fan. The reaction mixture was stirred for 16 hours under blue LED irradiation, and then diluted with CH_2Cl_2 (2 mL). The reaction mixture was filtered through a short pad of Celite using CH_2Cl_2 (20 mL) as eluent. The filtrate was collected and concentrated *in vacuo,* and the residue was then purified by flash column chromatography on silica gel, eluting with ethyl acetate/pentane (1:10, v/v) to afford **24** (44.9 mg, 71% yield) as a grey solid.

R*f* = 0.33 (EtOAc/pentane, 1:10, v/v (UV)).

NMR Spectroscopy:

^{1}**H NMR** (500 MHz, $CDCl_3$, 25 °C, δ): 7.71 – 7.53 (m, 2H), 7.44 (d, *J* = 8.6 Hz, 2H), 7.35 – 7.28 (m, 2H), 6.92 (d, *J* = 8.6 Hz, 2H).

^{13}**C {^{1}H} NMR** (126 MHz, $CDCl_3$, 25 °C, δ): 155.9, 148.7, 141.4, 132.2, 128.7, 128.5, 121.7, 118.9 (q, *J* = 280.2 Hz, CF_3), 116.0.

^{19}**F NMR** (471 MHz, $CDCl_3$, 25 °C, δ): –72.8 (s).

HRMS-ESI(m/z) calc'd for $C_{12}H_{15}NO_4Na^+$ $[M+Na]^+$, 260.0893; found, 260.0896; deviation: –0.9 ppm.

Diclofenac amide thianthrenium salt (25-TT)

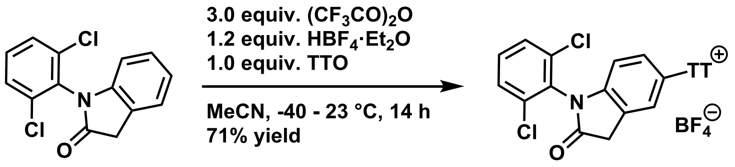

25-TT

Under an ambient atmosphere, a 20-mL glass vial was charged with diclofenac amide (695 mg, 2.50 mmol, 1.00 equiv) and MeCN (10 mL, *c* = 0.25 M). Trifluoroacetic anhydride (1.04 mL, 1.58 g, 7.50 mmol, 3.00 equiv.) was added while stirring the reaction mixture at 25 °C. After cooling to –40 °C, thianthrene-*S*-oxide (580 mg, 2.50 mmol, 1.00 equiv.) was added in one portion, followed by the dropwise addition of $HBF_4 \cdot OEt_2$ (408 µL, 485 mg, 3.00 mmol, 1.2 equiv.). The mixture was stirred at –40 °C for 1 hour, then warmed to 25 °C for 14 hours. The solution was diluted with CH_2Cl_2 (5 mL) and poured onto a mixture of CH_2Cl_2 (20 mL) and saturated aqueous $NaHCO_3$ solution (30 mL). After stirring for 5 min, the mixture was poured into a separating funnel, and the aqueous phase was extracted with DCM (2 × 20 mL). The DCM layer was washed with aqueous $NaBF_4$ solution (10% w/w, 2 × ca. 20 mL). The DCM layer was dried over $MgSO_4$, filtered, and the solvent was removed under reduced pressure. The residue was purified by chromatography on silica gel eluting with DCM/MeOH (40:1 (v/v)), then the solvent was removed *in vacuo* to afford **25-TT** (1.03 g, 71% yield) as a grey powder.

R*f* = 0.35 (MeOH/DCM, 1/15, v/v).

NMR Spectroscopy:

^{1}H NMR (500 MHz, CD_3CN, 25 °C, δ) 7.93 (d, *J* = 1.4 Hz, 2H), 7.85 (td, *J* = 7.7, 1.4 Hz, 2H), 7.78 (td, *J* = 7.7, 1.4 Hz, 2H), 7.57 (dd, *J* = 8.1, 0.9 Hz, 2H), 7.49 (dd, *J* = 9.1, 7.2 Hz, 2H), 7.27 (dt, *J* = 2.3, 1.2 Hz, 1H), 7.10 (ddd, *J* = 8.5, 2.1, 1.0 Hz, 1H), 6.51 (d, *J* = 8.6 Hz, 1H), 3.75 (d, *J* = 1.0 Hz, 2H).

^{13}C {^{1}H} NMR (126 MHz, CD_3CN, 25 °C, δ): 173.6, 148.4, 137.1, 135.9, 135.5, 135.5, 133.0, 131.5, 130.9, 130.2, 129.9, 128.5, 125.8, 119.6, 117.6, 111.2, 35.8.

^{19}F NMR (471 MHz, CD_3CN, 25 °C, δ): –150.5 (brs), –150.6 (brs).

HRMS-ESI(m/z) calc'd for $C_{26}H_{16}Cl_2NOS_2^+$ $[M-BF_4]^+$, 492.0045; found, 492.0041; deviation: 0.8 ppm.

Hydroxy-diclofenac amide (25)

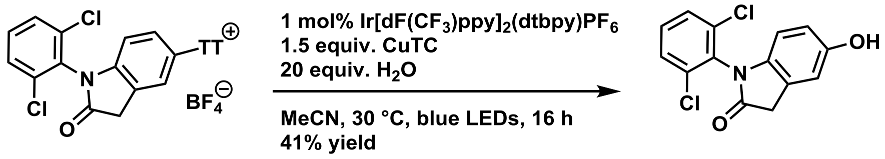

25-TT **25**

To a 4-mL borosilicate vial, equipped with a magnetic stir bar was added copper(I) thiophene-2-carboxylate (28.6 mg, 0.150 mmol, 1.50 equiv.), diclofenac amide thianthrenium salt **25-TT** (58.1 mg, 0.100 mmol, 1.00 equiv.), $Ir[dF(CF_3)ppy]_2(dtbpy)PF_6$ (1.1 mg, 1.0 µmol, 1.0 mol%), water (36 µL, 18 mg, 2.0 mmol, 20 equiv.), and MeCN (0.5 mL, *c* = 0.2 M). The vial was evacuated and then filled with argon; this procedure was repeated three times. The vial was placed in 2 cm distance to two 34 W blue LEDs. The temperature was kept at approximately 30 °C with the use of a cooling fan. The reaction mixture was stirred for 16 hours under blue LED irradiation, and then diluted with CH_2Cl_2 (2 mL). The reaction mixture was filtered through a short pad of Celite using CH_2Cl_2 (20 mL) as eluent. The filtrate was collected and concentrated *in vacuo,* and the residue was then purified by flash column chromatography on silica gel, eluting with ethyl acetate/pentane (1:10 to 1:5, v/v) to afford **25** (12.1 mg, 41% yield) as a black solid.

R_f = 0.30 (EtOAc/pentane,1:2, v/v (UV, cerium molybdate)).

NMR Spectroscopy:

^{1}H NMR (500 MHz, CD_3CN, 25 °C, δ) 7.61 (d, *J* = 8.1 Hz, 2H), 7.49 (dd, *J* = 8.7, 7.6 Hz, 1H), 6.89 (d, *J* = 2.4 Hz, 1H), 6.77 (brs, 1H, OH), 6.63 (dd, *J* = 8.4, 2.5 Hz, 1H), 6.24 (d, *J* = 8.4 Hz, 1H), 3.69 (s, 2H).

^{13}C {^{1}H} NMR (126 MHz, CD_3CN, 25 °C, δ): 174.2, 154.2, 137.1, 136.1, 132.3, 131.7, 130.2, 127.0, 114.7, 113.9, 110.2, 36.5.

HRMS-ESI(m/z) calc'd for $C_{15}H_{16}NO_2Cl_2^+$ $[M]^+$, 294.0083; found, 294.0083; deviation: 0 ppm.

Hydroxy-pyriproxyfen (26)

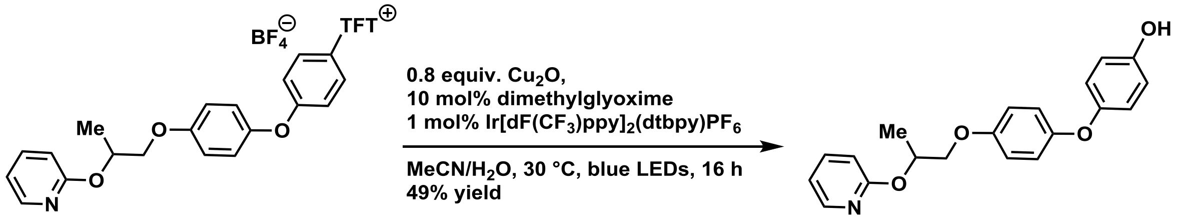

26-TFT 26

To a 4-mL borosilicate vial, equipped with a magnetic stir bar was added copper(I) oxide (11.5 mg, 80.0 µmol, 0.800 equiv.), dimethylglyoxime (1.2 mg, 10 µmol, 0.10 equiv.), and MeCN/H_2O (0.9 mL, v/v= 2:1). After stirring for 10 mins at ambient temperature, pyriproxyfen tetrafluorothianthrenium salt **26-TFT** (92.8 mg, 0.100 mmol, 1.00 equiv.), and Ir[dF(CF_3)ppy]$_2$(dtbpy)PF_6 (1.1 mg, 1.0 µmol, 1.0 mol%) in MeCN (0.40 mL, *c* = 0.25 M) were then added. The vial was evacuated and then filled with argon; this procedure was repeated three times. The vial was placed in 2 cm distance to two 34 W blue LEDs. The temperature was kept at approximately 30 °C with the use of a cooling fan. The reaction mixture was stirred for 16 hours under blue LED irradiation, and then diluted with CH_2Cl_2 (2 mL). The reaction mixture was filtered through a short pad of Celite using CH_2Cl_2 (20 mL) as eluent. The filtrate was collected and concentrated *in vacuo,* and the residue was then purified by flash column chromatography on silica gel, eluting with ethyl acetate/pentane (1:20 to 1:5, v/v) to afford **26** (16.6 mg, 49% yield) as a pale yellow solid.

R*f* = 0.29 (EtOAc/pentane, 1:2, v/v (UV, cerium molybdate)).

NMR Spectroscopy:

^{1}H NMR (500 MHz, CD_3CN, 25 °C, δ): 8.13 (ddd, *J* = 5.0, 2.0, 0.9 Hz, 1H), 7.64 (ddd, *J* = 8.4, 7.1, 2.0 Hz, 1H), 7.04 – 6.59 (m, 10H), 5.66 – 5.50 (m, 1H), 4.23 – 4.02 (m, 2H), 1.39 (d, *J* = 6.4 Hz, 3H).

^{13}C {^{1}H} NMR (126 MHz, $CDCl_3$, 25 °C, δ): 163.1, 154.6, 152.0, 151.6, 151.5, 146.3, 139.7, 119.9, 119.5, 117.1, 116.4, 115.9, 112.0, 71.3, 70.4, 17.1.

HRMS-ESI(m/z) calc'd for $C_{20}H_{20}NO_2^+$ [M+H]$^+$, 338.1387; found, 338.1388; deviation: –0.4 ppm.

General procedure and reaction condition optimization for etherification

General procedure of etherification

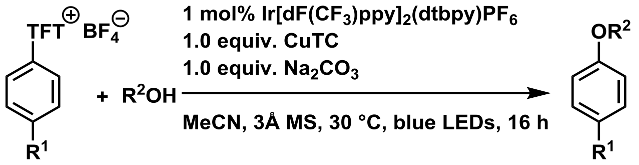

In an anhydrous, N_2-filled glovebox, a 4-mL borosilicate vial equipped with a magnetic stir bar was charged with copper(I) thiophene-2-carboxylate (38.1 mg, 0.200 mmol, 1.00 equiv.), alcohol (0.400 mmol, 2.00 equiv.), Na_2CO_3 (10.6 mg, 0.100 mmol, 1.00 equiv.), and 3 Å molecular sieves (120 mg). Dry MeCN (1 mL, *c* = 0.2 M)

was then added into the vial. The vial was sealed with a Teflon cap. The reaction mixture was stirred at 23 °C: After 2 hours, the vial was opened and (tetrafluoro)thianthrenium salt (0.200 mmol, 1.00 equiv.) and Ir[dF(CF_3)ppy]$_2$(dtbpy)PF_6 (2.2 mg, 2.0 µmol, 1.0 mol%) were added into the reaction mixture. The vial was sealed with the same Teflon cap and transferred out of glovebox. The vial was placed in 2 cm distance to two 34 W blue LEDs. The temperature was kept at approximately 30 °C with the use of a cooling fan. The reaction mixture was stirred for 16 hours under blue LED irradiation, and then diluted with CH_2Cl_2 (2 mL). The reaction mixture was filtered through a short pad of silica using CH_2Cl_2 (25 mL) as eluent. The filtrate was collected and concentrated *in vacuo,* and the residue was then purified by flash column chromatography on silica gel, eluting with ethyl acetate/hexanes to afford the etherificated product.

Note: The reaction is air sensitive. Schlenk techniques can be used to avoid air if a glovebox is not available. For simplicity, in our own research, we have opted to execute the transformation for most compounds by using a glovebox. If moisture is present, phenols (resulting from hydroxylation as opposed to alkoxylation) are observed as byproducts. In addition, biarylethers (**29-C**) are observed as byproducts, possibly resulting from cross coupling of the in situ formed phenols and arylthianthrenium salts.

Table S2. Reaction condition optimization

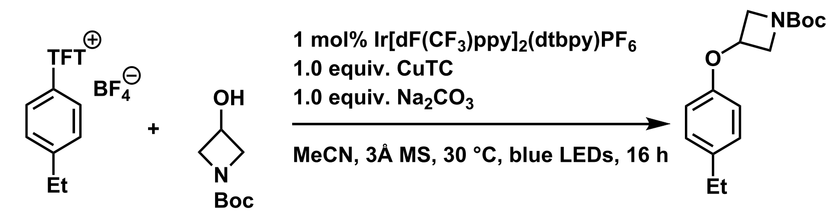

28-TFT **29**

0.1 mmol (1.0 equiv.) **0.2 mmol, (2.0 equiv.)**

Change of reaction conditions	Yield of **29**[b]
none[a]	72%
no Na_2CO_3	30%
Open air instead of in an anhydrous, N_2-filled glovebox	42%
1 equiv. N-Boc-3-hydroxyazetidine	61%
No prestrring of N-Boc-3-hydroxyazetidine, Na_2CO_3 and CuTC	64%

Organic dye 4CzIPN instead of [Ir[dF(CF_3)ppy]$_2$(dtbpy)PF_6 [c]	30%
Reaction is carried out without purification of the aryl thianthrenium salts	17%

[a]N-Boc-3-hydroxyazetidine (2.0 equiv.), CuTC (1.0 equiv.), Na_2CO_3 (1.0 equiv.), 3 Å MS, acetonitrile, 2 hours; followed by addition of thianthrenium salt (1.0 equiv.), [Ir[dF(CF_3)ppy]$_2$(dtbpy)PF_6] (1 mol%), blue LED (34 W), 30 ^{o}C, 16 hours. [b]Yield based on NMR with 0.1 mmol styrol as internal standard. [c]Organic dye 4CzIPN (5 mmol%).

Side product without Na_2CO_3: 4-ethylphenyl thiophene-2-carboxylate (29-B)

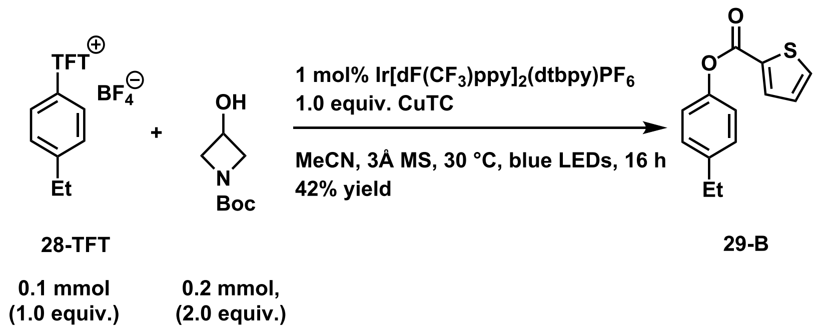

In an anhydrous, N_2-filled glovebox, a 4-mL borosilicate vial equipped with a magnetic stir bar was charged with copper(I) thiophene-2-carboxylate (19.0 mg, 0.100 mmol, 1.00 equiv.), N-Boc-3-hydroxyazetidin (34.6 mg, 0.200 mmol, 2.00 equiv.), and 3 Å molecular sieves (120 mg). Dry MeCN (1.0 mL, *c* = 0.10 M) was then added into the vial. The vial was sealed with a Teflon cap. The reaction mixture was stirred at 23 ^{o}C: After 2 hours, the vial was opened and ethyl benzene-derived tetrafluorothianthrenium salt **28-TFT** (48.0 mg, 0.100 mmol, 1.00 equiv.) and Ir[dF(CF_3)ppy]$_2$(dtbpy)PF_6 (1.1 mg, 1.0 µmol, 1.0 mol%) were added into the reaction mixture. The vial was sealed with the same Teflon cap and transferred out of glovebox. The vial was placed in 2 cm distance to two 34 W blue LEDs. The temperature was kept at approximately 30 °C with the use of a cooling fan. The reaction mixture was stirred for 16 hours under blue LED irradiation, and then diluted with CH_2Cl_2 (2 mL). The reaction mixture was filtered through a short pad of silica using CH_2Cl_2 (25 mL) as eluent. The filtrate was collected and concentrated *in vacuo,* and the residue was then purified by flash column chromatography on silica gel, eluting with EtOAc/pentane (1: 100 v/v) to afford **29-B** (8.9 mg, 42% yield) as a colorless solid.

R_f = 0.3 (EtOAc/pentane, 1:50, v/v (UV)

NMR Spectroscopy:

^{1}**H NMR** (500 MHz, $CDCl_3$, 25 °C, δ): 7.98 (dd, J = 3.7, 1.4 Hz, 1H), 7.65 (dd, J = 4.9, 1.3 Hz, 1H), 7.24 (d, J = 8.5 Hz, 2H), 7.17 (dd, J = 5.1, 3.7 Hz, 1H), 7.12 (d, J = 8.5 Hz, 2H), 2.67 (q, J = 7.6 Hz, 2H), 1.25 (t, J = 7.6 Hz, 3H).

^{13}**C {^{1}H} NMR** (126 MHz, $CDCl_3$, 25 °C, δ): 160.9, 148.6, 142.1, 134.7, 133.5, 133.2, 128.9, 128.1, 121.5,

28.5, 15.7.

HRMS-ESI(m/z) calc'd for $C_{13}H_{12}O_2S_1^+$ [M]$^+$, 232.0553; found, 232.0553. Deviation: 0 ppm.

Side product: biarylether (29-C)

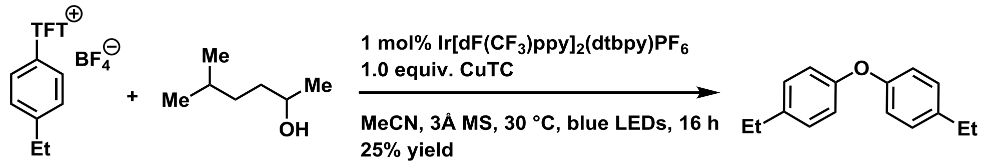

32-TFT 29-C

In an anhydrous, N_2-filled glovebox, a 4-mL borosilicate vial equipped with a magnetic stir bar was charged with copper(I) thiophene-2-carboxylate (19.0 mg, 0.100 mmol, 1.00 equiv.), 5-methylhexan-2-ol (23.2 mg, 0.200 mmol, 2.00 equiv.), and 3 Å molecular sieves (120 mg). Dry MeCN (1.0 mL, *c* = 0.10 M) was then added into the vial. The vial was sealed with a Teflon cap. The reaction mixture was stirred at 23 ^{o}C: After 2 hours, the vial was opened and ethyl benzene-derived tetrafluorothianthrenium salt **28-TFT** (48.0 mg, 0.100 mmol, 1.00 equiv.) and Ir[dF(CF_3)ppy]$_2$(dtbpy)PF_6 (1.1 mg, 1.0 µmol, 1.0 mol%) were added into the reaction mixture. The vial was sealed with the same Teflon cap and transferred out of glovebox. The vial was placed in 2 cm distance to two 34 W blue LEDs. The temperature was kept at approximately 30 °C with the use of a cooling fan. The reaction mixture was stirred for 16 hours under blue LED irradiation, and then diluted with CH_2Cl_2 (2 mL). The reaction mixture was filtered through a short pad of silica using CH_2Cl_2 (25 mL) as eluent. The filtrate was collected and concentrated *in vacuo,* and the residue was then purified by flash column chromatography on silica gel, eluting with pentane to afford **29-C** (5.4 mg, 25% yield) as a colorless solid.

R*f* = 0.30 (pentane (UV))

NMR Spectroscopy:

^{1}H NMR (500 MHz, $CDCl_3$, 25 °C, δ): 7.21 – 7.11 (m, 4H), 7.03 – 6.87 (m, 4H), 2.74 – 2.61 (m, 4H), 1.23 (td, *J* = 7.6, 0.9 Hz, 6H).

^{13}C {^{1}H} NMR (126 MHz, $CDCl_3$, 25 °C, δ): 155.6, 139.0, 129.1, 118.8, 28.3, 15.9.

HRMS-ESI(m/z) calc'd for $C_{16}H_{18}O_1^+$ [M]$^+$, 226.1352; found, 226.1354. Deviation: –0.82 ppm.

Figure S3. Comparison of the etherifcation of TT and TFT salts

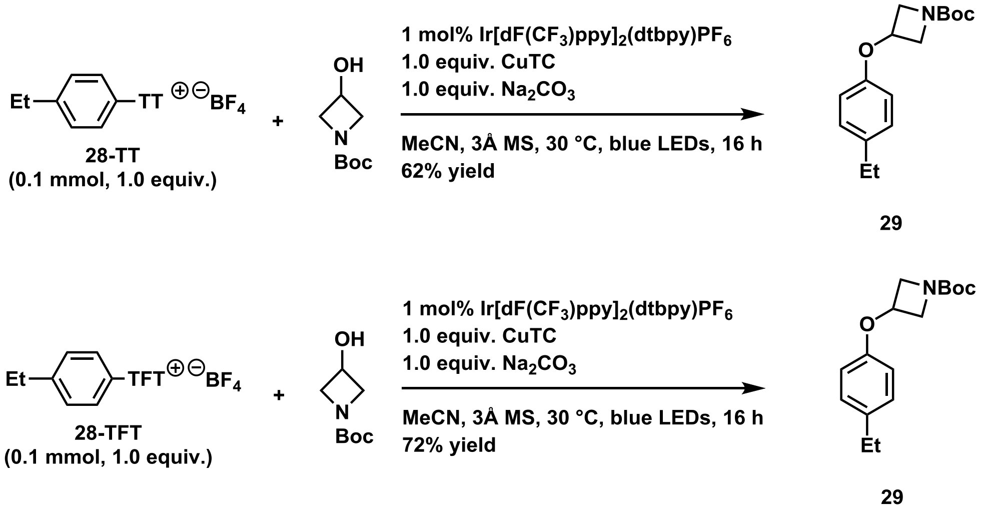

Thianthrenation and etherfication of arenes

2-Fluoro-6-phenoxybenzonitrile tetrafluorothianthrenium salt (27-TFT)

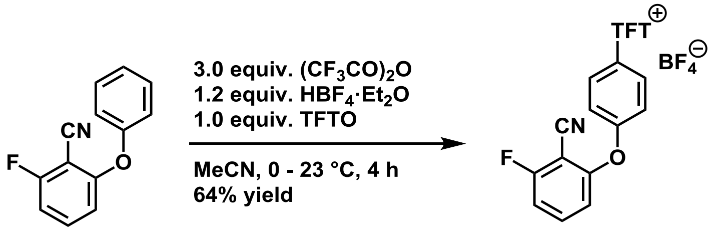

27-TFT

Under an ambient atmosphere, a 20-mL glass vial was charged with 2-fluoro-6-phenoxybenzonitrile (640 mg, 3.00 mmol, 1.00 equiv) and MeCN (5 mL, *c* = 0.6 M). After cooling to 0 °C, $HBF_4{\cdot}OEt_2$ (0.50mL, 0.59 g, 3.6 mmol, 1.2 equiv) and tetrafluorothianthrene-*S*-oxide (912 mg, 3.00 mmol, 1.00 equiv) was added to the vial while stirring the mixture, leading to a suspension. Subsequently, trifluoroacetic anhydride (1.25 mL, 1.89 g, 9.00 mmol, 3.00 equiv) was added in one portion at 0 °C, resulting in a color change to deep purple. Subsequently, the reaction mixture was allowed to reach 23 ^{o}C and stirred for 4 hours. The solution was diluted with DCM (5 mL) and poured onto a mixture of DCM (30 mL) and saturated aqueous $NaHCO_3$ solution (20 mL). After stirring for 5 min at 23 ^{o}C, the mixture was poured into a separating funnel, and the layers were separated. The DCM layer was washed with aqueous $NaBF_4$ solution (10% w/w, 4 × ca. 20 mL). The DCM layer was dried over Na_2SO_4, filtered, and the solvent was removed under reduced pressure. The residue was purified by chromatography on silica gel eluting with DCM/MeOH (30:1 (v/v)), then the solvent was removed *in vacuo* to afford **27-TFT** (963 mg, 64% yield) as a colorless solid.

R*f* = 0.35 (DCM/MeOH, 15:1, v/v).

NMR Spectroscopy:

^{1}H NMR (500 MHz, CD_3CN, 25 °C, δ): 8.44 (dd, *J* = 9.1, 7.2 Hz, 2H), 7.97 (dd, *J* = 9.9, 7.0 Hz, 2H), 7.67 (td, *J* = 8.6, 6.6 Hz, 1H), 7.40 – 7.28 (m, 2H), 7.26 – 7.12 (m, 3H), 6.94 – 6.82 (m, 1H) ppm.

^{13}C {^{1}H} NMR (126 MHz, CD_3CN, 25 °C, δ): 164.8 (d, *J* = 257.7 Hz), 160.4, 158.7 (d, *J* = 3.6 Hz), 154.7 (dd, *J* = 262.0, 13.1 Hz), 151.5 (dd, *J* = 255.5, 13.5 Hz), 137.4 (d, *J* = 10.7 Hz), 135.2 (dd, *J* = 8.7, 3.9 Hz), 132.0, 125.4 (dd, *J* = 22.1, 2.4 Hz), 121.3, 121.1 (d, *J* = 21.7 Hz), 118.4, 116.1 (d, *J* = 3.1 Hz), 115.5 (dd, *J* = 7.2, 3.5 Hz), 113.1 (d, *J* = 19.7 Hz), 111.6, 95.9 (d, *J* = 18.3 Hz) ppm.

^{19}F NMR (471 MHz, CD_3CN, 25 °C, δ): δ –106.6 (d, *J* = 6.8 Hz), –125.3 (m), –133.7 (ddd, *J* = 20.4, 9.3, 7.0 Hz), –150.9 (brs), –151.0 (brs) ppm.

HRMS-ESI(m/z) calc'd for $C_{25}H_{11}F_5NOS_2^+$ $[M-BF_4]^+$, 500.0197; found, 500.0195; deviation: 0.4 ppm.

2-Fluoro-6-(4-methoxyphenoxy)benzonitrile (27)

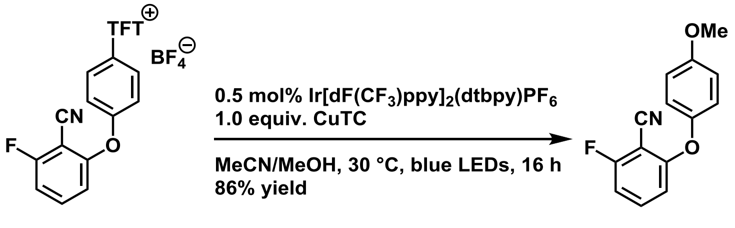

27-TFT 27

To a 4-mL borosilicate vial, equipped with a magnetic stir bar was added copper(I) thiophene-2-carboxylate (38.1 mg, 0.200 mmol, 1.00 equiv.), 2-fluoro-6-phenoxybenzonitrile tetrafluorothianthrenium salt **27-TFT** (118 mg, 0.200 mmol, 1.00 equiv.), $Ir[dF(CF_3)ppy]_2(dtbpy)PF_6$ (1.1 mg, 1.0 µmol, 0.50 mol%), and MeCN/MeOH (1.2 mL, v/v= 5:1, *c* = 0.17 M). The vial was evacuated and then filled with argon; this procedure was repeated three times. The vial was placed in 2 cm distance to two 34 W blue LEDs. The temperature was kept at approximately 30 °C with the use of a cooling fan. The reaction mixture was stirred for 16 hours under blue LED irradiation, and then diluted with TBME (1 mL). The reaction mixture was filtered through a short pad of silica using TBME (20 mL) as eluent. The filtrate was collected and concentrated *in vacuo,* and the residue was then purified by flash column chromatography on silica gel, eluting with Et_2O/pentane (1:5, v/v) to afford **27** (42.0 mg, 86% yield) as a colorless oil.

R*f* = 0.40 (EtOAc/pentane, 1:10, v/v (UV)).

NMR Spectroscopy:

^{1}H NMR (400 MHz, CD_2Cl_2, 25 °C, δ): 7.42 (td, *J* = 8.6, 6.6 Hz, 1H), 7.12 – 7.04 (m, 2H), 7.02 – 6.93 (m, 2H), 6.87 (td, *J* = 8.5, 0.8 Hz, 1H), 6.55 (dt, *J* = 8.7, 0.8 Hz, 1H), 3.82 (s, 3H).

^{13}C {^{1}H} NMR (101 MHz, CD_2Cl_2, 25 °C, δ): 164.6 (d, *J* = 258.0 Hz), 162.5 (d, *J* = 4.3 Hz), 158.0, 148.0, 135.5 (d, *J* = 10.5 Hz), 122.3, 115.7, 111.8, 111.5 (d, *J* = 3.3 Hz), 109.6 (d, *J* = 19.6 Hz), 93.3 (d, *J* = 18.1 Hz), 56.2.

^{19}F NMR (376 MHz, CD_2Cl_2, 25 °C, δ): –106.5.

HRMS-ESI(m/z) calc'd for $C_{14}H_{10}NO_2F^+$ [M]$^+$, 243.0690; found, 243.0690. Deviation: 0 ppm.

1-Ethyl-4-phenoxybenzene (28)

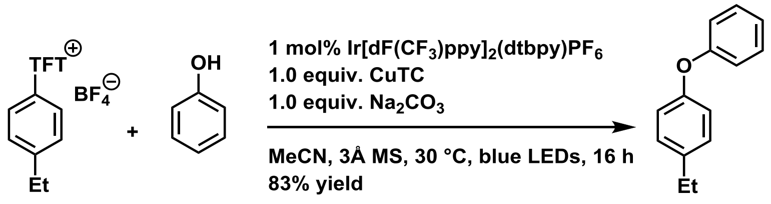

28-TFT **28**

In an anhydrous, N_2-filled glovebox, a 4-mL borosilicate vial equipped with a magnetic stir bar was charged with copper(I) thiophene-2-carboxylate (38.1 mg, 0.200 mmol, 1.00 equiv.), phenol (37.6 mg, 0.400 mmol, 2.00 equiv.), Na_2CO_3 (10.6 mg, 0.100 mmol, 1.00 equiv.), and 3 Å molecular sieves (120 mg). Dry MeCN (1 mL, *c* = 0.2 M) was then added into the vial. The vial was sealed with a Teflon cap. The reaction mixture was stirred at 23 °C: After 2 hours, the vial was opened and ethyl benzene-derived tetrafluorothianthrenium salt **28-TFT** (48.0 mg, 0.100 mmol, 1.00 equiv.) and Ir[dF(CF_3)ppy]$_2$(dtbpy)PF_6 (1.1 mg, 1.0 µmol, 1.0 mol%) were added into the reaction mixture. The vial was sealed with the same Teflon cap and transferred out of glovebox. The vial was placed in 2 cm distance to two 34 W blue LEDs. The temperature was kept at approximately 30 °C with the use of a cooling fan. The reaction mixture was stirred for 16 hours under blue LED irradiation, and then diluted with CH_2Cl_2 (2 mL). The reaction mixture was filtered through a short pad of silica using CH_2Cl_2 (25 mL) as eluent. The filtrate was collected and concentrated *in vacuo,* and the residue was then purified by flash column chromatography on silica gel, eluting with pentane to afford **28** (32.8 mg, 83% yield) as a colorless solid.

$\mathbf{R}_f$ = 0.30 (pentane (UV))

NMR Spectroscopy:

> ^{1}**H NMR** (500 MHz, $CDCl_3$, 25 °C, δ): 7.37–7.30 (m, 2H), 7.18 (d, *J* = 8.6 Hz, 2H), 7.09 (tt, *J* = 7.4 Hz, 1.2 Hz, 1H), 7.01 (dd, *J* = 8.7 Hz, 1.1 Hz, 2H), 6.98 – 6.94 (m, 2H), 2.66 (q, *J* = 7.6 Hz, 2H), 1.26 (t, *J* = 7.6 Hz, 3H).

> ^{13}**C {^{1}H} NMR** (126 MHz, $CDCl_3$, 25 °C, δ): 157.8, 154.9, 139.3, 129.7, 129.0, 122.8, 119.1, 118.5, 28.2, 15.8.

HRMS-ESI(m/z) calc'd for calc'd for $C_{14}H_{14}O^+$ [M]$^+$,198.1039; found,198.1040. Deviation: –0.2 ppm.

tert-Butyl 3-(4-ethylphenoxy)azetidine-1-carboxylate (29)

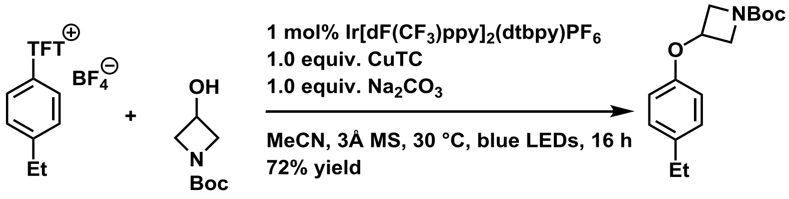

28-TFT **29**

In an anhydrous, N_2-filled glovebox, a 4-mL borosilicate vial equipped with a magnetic stir bar was charged with copper(I) thiophene-2-carboxylate (38.1 mg, 0.200 mmol, 1.00 equiv.), N-Boc-3-hydroxyazetidin (69.2 mg, 0.400 mmol, 2.00 equiv.), Na_2CO_3 (10.6 mg, 0.100 mmol, 1.00 equiv.), and 3 Å molecular sieves (120 mg). Dry MeCN (1 mL, *c* = 0.2 M) was then added into the vial. The vial was sealed with a Teflon cap. The reaction mixture was stirred at 23 ^{o}C: After 2 hours, the vial was opened and ethyl benzene-derived tetrafluorothianthrenium salt **28-TFT** (48.0 mg, 0.100 mmol, 1.00 equiv.) and Ir[dF(CF_3)ppy]$_2$(dtbpy)PF_6 (1.1 mg, 1.0 µmol, 1.0 mol%) were added into the reaction mixture. The vial was placed in 2 cm distance to two 34 W blue LEDs. The temperature was kept at approximately 30 °C with the use of a cooling fan. The reaction mixture was stirred for 16 hours under blue LED irradiation, and then diluted with CH_2Cl_2 (2 mL). The reaction mixture was filtered through a short pad of silica using CH_2Cl_2 (25 mL) as eluent. The filtrate was collected and concentrated *in vacuo,* and the residue was then purified by flash column chromatography on silica gel, eluting with EtOAc/pentane (1: 10 v/v) to afford **29** (40.0 mg, 72% yield) as a colorless solid.

$\mathbf{R}_f$ = 0.40 (EtOAc/pentane, 1:10, v/v (UV, cerium molybdate))

NMR Spectroscopy:

^{1}H NMR (300 MHz, CD_3CN, 25 °C, δ): 7.13 (d, *J* = 8.8 Hz, 2H), 6.71 (d, *J* = 8.6 Hz, 2H), 4.89 (tt, *J* = 6.4 Hz, 4.0 Hz, 1H), 4.25 (ddd, *J* = 9.6 Hz, 6.4 Hz, 1.1 Hz, 1H), 3.87 – 3.80 (m, 1H), 2.58 (q, *J* = 7.6 Hz, 2H), 1.42 (s, 9H), 1.17 (t, *J* = 7.6 Hz, 3H).

^{13}C {^{1}H} NMR (126 MHz, CD_3CN, 25 °C, δ): 157.3, 156.0, 138.6, 130.1, 115.8, 80.2, 67.2, 57.5, 28.7, 28.7, 16.3.

HRMS-ESI(m/z) calc'd for $C_{16}H_{23}NO_3Na^+$ [M+Na]$^+$, 300.1570; found, 300.1572. Deviation: –0.5 ppm.

trans-2-(4-Rthylphenoxy)cyclohexan-1-ol (30)

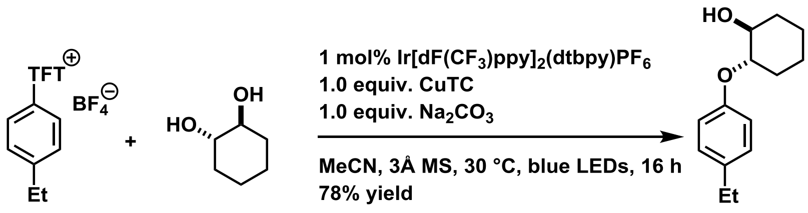

28-TFT **30**

In an anhydrous, N_2-filled glovebox, a 4-mL borosilicate vial equipped with a magnetic stir bar was charged

with copper(I) thiophene-2-carboxylate (38.1 mg, 0.200 mmol, 1.00 equiv.), *trans*-1,2-cyclohexandiol (46.5 mg, 0.400 mmol, 2.00 equiv.), Na_2CO_3 (10.6 mg, 0.100 mmol, 1.00 equiv.), and 3 Å molecular sieves (120 mg). Dry MeCN (1 mL, *c* = 0.2 M) was then added into the vial. The vial was sealed with a Teflon cap. The reaction mixture was stirred at 23 °C: After 2 hours, the vial was opened and ethyl benzene-derived tetrafluorothianthrenium salt **28-TFT** (48.0 mg, 0.100 mmol, 1.00 equiv.) and Ir[dF(CF_3)ppy]$_2$(dtbpy)PF_6 (1.1 mg, 1.0 µmol, 1.0 mol%) were added into the reaction mixture. The vial was sealed with the same Teflon cap and transferred out of glovebox. The vial was placed in 2 cm distance to two 34 W blue LEDs. The temperature was kept at approximately 30 °C with the use of a cooling fan. The reaction mixture was stirred for 16 hours under blue LED irradiation, and then diluted with CH_2Cl_2 (2 mL). The reaction mixture was filtered through a short pad of silica using CH_2Cl_2 (25 mL) as eluent. The filtrate was collected and concentrated *in vacuo,* and the residue was then purified by flash column chromatography on silica gel, eluting with EtOAc/pentane (1:10 v/v) to afford **30** (34.5 mg, 78% yield) as a colorless solid.

R*f* = 0.30 (EtOAc/pentane, 1:5. v/v (UV, cerium molybdate))

NMR Spectroscopy:

^{1}H NMR (500 MHz, CD_3CN, 25 °C, δ): 7.17 (d, *J* = 8.6 Hz, 2H), 6.87 (d, *J* = 8.6 Hz, 2H), 3.94 (ddd, *J* = 9.8 Hz, 8.2 Hz, 4.2 Hz, 1H), 3.57 (ddt, J = 9.7 Hz, 7.9 Hz, 3.8 Hz, 1H), 3.18 (d, *J* = 3.3 Hz, 1H), 2.57 (q, *J* = 7.6 Hz, 2H), 2.08 – 2.04 (m, 1H), 1.98 – 1.95 (m, 0.5H), 1.69 – 1.66 (m, 2H), 1.39 – 1.21 (m, 4.5H), 1.18 (t, *J* = 7.6 Hz, 3H).

^{13}C {^{1}H} NMR (126 MHz, CD_3CN, 25 °C, δ): 157.4, 137.5, 129.7, 117.0, 82.6, 73.4, 33.5, 30.2, 28.6, 24.5, 24.5, 16.4.

HRMS-ESI(m/z) calc'd for $C_{14}H_{20}O_2Na^+$ $[M+Na]^+$, 243.1355; found, 243.1353. Deviation: 1.2 ppm.

2-Fluoro-6-(4-(methoxy-d$_3$)phenoxy)benzonitrile (31)

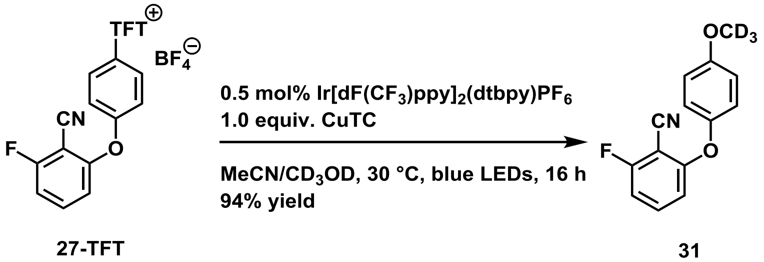

27-TFT **31**

To a 4-mL borosilicate vial, equipped with a magnetic stir bar was added Ir[dF(CF_3)ppy]$_2$(dtbpy)PF_6 (1.1 mg, 1.0 µmol, 0.50 mol%), copper(I) thiophene-2-carboxylate (38.1 mg, 0.200 mol, 1.00 equiv.), and 2-fluoro-6-phenoxybenzonitrile tetrafluorothianthrenium salt **27-TFT** (118 mg, 0.200 mmol, 1.00 equiv.) at 25 °C. The vial was evacuated and then filled with argon; this procedure was repeated three times. MeCN (1 mL, *c* = 0.2 M) was added, followed by CD_3OD (0.16 g, 0.20 mL, 4.4 mmol, 22 equiv.). The vial was placed in 2 cm distance to two 34 W blue LEDs. The temperature was kept at approximately 30 °C with the use of a cooling fan. The reaction mixture was stirred for 16 hours under blue LED irradiation, and then diluted with methyl-

tert-butylether (1 mL). The reaction mixture was filtered through a short pad of silica using methyl-*tert*-butylether (20 mL) as eluent. The filtrate was collected and concentrated *in vacuo* and then purified by flash column chromatography on silica gel, eluting with Et_2O/pentane (1:5 v/v) to afford **31** (46.2 mg, 94% yield) as a colorless oil.

R*f* = 0.40 (EtOAc/pentane, 1:10, v/v (UV)).

NMR Spectroscopy:

^{1}H NMR (500 MHz, $CDCl_3$, 25 °C, δ): 7.06–7.03 (m, 2H), 6.95–6.92 (m, 2H), 6.83 (td, *J* = 8.5 Hz, 0.9 Hz, 1H), 6.53 (dt, *J* = 8.5 Hz, 0.9 Hz, 1H).

^{13}C {^{1}H} NMR (126 MHz, $CDCl_3$, 25 °C, δ): 165.3, 163.2, 162.2 (d, *J* = 4.1 Hz), 157.5, 147.6, 134.9 (d, *J* = 10.2 Hz), 122.0, 115.3, 111.4, 111.0 (d, *J* = 3.4 Hz), 109.2 (d, *J* = 19.6 Hz), 55.5–54.7 (m, OCD_3).

^{19}F NMR (471 MHz, $CDCl_3$, 25 °C, δ): –105.0.

HRMS-ESI(m/z) calc'd for $C_{14}H_7NO_2FD_3Na^+$ $[M+Na]^+$, 269.0776; found, 269.0777. Deviation: –0.4 ppm.

1-Cyclobutoxy-4-ethylbenzene (32)

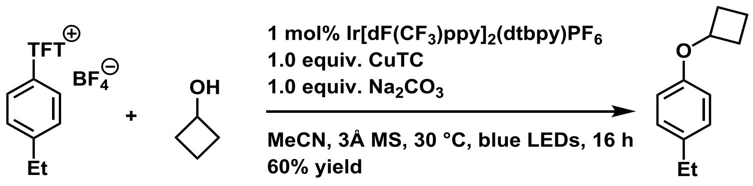

28-TFT **32**

In an anhydrous, N_2-filled glovebox, a 4-mL borosilicate vial equipped with a magnetic stir bar was charged with copper(I) thiophene-2-carboxylate (38.1 mg, 0.200 mmol, 1.00 equiv.), cyclobutanol (72.1 mg, 78.3 µL, 1.00 mmol, 5.00 equiv.), Na_2CO_3 (10.6 mg, 0.100 mmol, 1.00 equiv.), and 3 Å molecular sieves (120 mg). Dry MeCN (1 mL, *c* = 0.2 M) was then added into the vial. The vial was sealed with a Teflon cap. The reaction mixture was stirred at 23 ^{o}C: After 2 hours, the vial was opened and ethyl benzene-derived tetrafluorothianthrenium salt **28-TFT** (48.0 mg, 0.100 mmol, 1.00 equiv.) and Ir[dF(CF_3)ppy]$_2$(dtbpy)PF_6 (1.1 mg, 1.0 µmol, 1.0 mol%) were added into the reaction mixture. The vial was sealed with the same Teflon cap and transferred out of glovebox. The vial was placed in 2 cm distance to two 34 W blue LEDs. The temperature was kept at approximately 30 °C with the use of a cooling fan. The reaction mixture was stirred for 16 hours under blue LED irradiation, and then diluted with CH_2Cl_2 (2 mL). The reaction mixture was filtered through a short pad of silica using CH_2Cl_2 (25 mL) as eluent. The filtrate was collected and concentrated *in vacuo,* and the residue was then purified by flash column chromatography on silica gel, eluting with EtOAc/pentane (1: 200 v/v) to afford **32** (21.4 mg, 60% yield) as a colorless oil.

R*f* = 0.30 (EtOAc/pentane, 1:100, v/v (UV, cerium molybdate))

NMR Spectroscopy:

^{1}H NMR (500 MHz, $CDCl_3$, 25 °C, δ): 7.09 (d, *J* = 8.8 Hz, 2H), 6.75 (d, *J* = 8.6 Hz, 2H), 4.68 – 4.56 (m, 1H), 2.59 (q, *J* = 7.6 Hz, 2H), 2.44 (dddt, *J* = 9.5 Hz, 8.1 Hz, 6.7 Hz, 2.6 Hz, 2H), 2.27 – 2.09 (m, 2H), 1.85 (m, 1H), 1.68 (dtt, *J* = 11.2 Hz, 10.1 Hz, 8.2 Hz, 1H), 1.21 (t, *J* = 7.6 Hz, 3H).

^{13}C {^{1}H} NMR (126 MHz, $CDCl_3$, 25 °C, δ): 155.6, 136.3, 128.7, 114.8, 71.5, 30.8, 28.0, 15.9, 13.3.

HRMS-ESI(m/z) calc'd for $C_{12}H_{17}O^+$ $[M+H]^+$, 177.1274; found, 177.1274. Deviation: –0.3 ppm.

3-(4-Ethylphenoxy)oxetane (33)

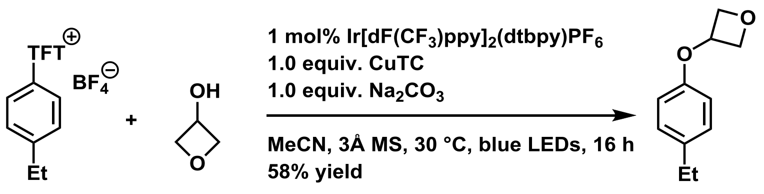

28-TFT **33**

In an anhydrous, N_2-filled glovebox, a 4-mL borosilicate vial equipped with a magnetic stir bar was charged with copper(I) thiophene-2-carboxylate (38.1 mg, 0.200 mmol, 1.00 equiv.), 3-hydroxyoxetan (37.0 mg, 31.7 µL, 0.500 mmol, 2.50 equiv.), Na_2CO_3 (10.6 mg, 0.100 mmol, 1.00 equiv.), and 3 Å molecular sieves (120 mg). Dry MeCN (1 mL, *c* = 0.2 M) was then added into the vial. The vial was sealed with a Teflon cap. The reaction mixture was stirred at 23 ^{o}C: After 2 hours, the vial was opened and ethyl benzene-derived tetrafluorothianthrenium salt **28-TFT** (48.0 mg, 0.100 mmol, 1.00 equiv.) and Ir[dF(CF_3)ppy]$_2$(dtbpy)PF_6 (1.1 mg, 1.0 µmol, 1.0 mol%) were added into the reaction mixture.The vial was sealed with the same Teflon cap and transferred out of glovebox. The vial was placed in 2 cm distance to two 34 W blue LEDs. The temperature was kept at approximately 30 °C with the use of a cooling fan. The reaction mixture was stirred for 16 hours under blue LED irradiation, and then diluted with CH_2Cl_2 (2 mL). The reaction mixture was filtered through a short pad of silica using CH_2Cl_2 (25 mL) as eluent. The filtrate was collected and concentrated *in vacuo,* and the residue was then purified by flash column chromatography on silica gel, eluting with EtOAc/pentane (1: 100 v/v) to afford **33** (20.6 mg, 58% yield) as a colorless oil.

R_f = 0.30 (EtOAc/pentane, 1:50, v/v (UV, cerium molybdate))

NMR Spectroscopy:

^{1}H NMR (300 MHz, $CDCl_3$, 25 °C, δ): 7.03 (d, *J* = 8.8 Hz, 2H), 6.55 (d, *J* = 8.6 Hz, 2H), 5.15 – 5.07(m, 1H), 4.92 – 4.83 (m, 2H), 4.75 – 4.61 (m, 2H), 2.51 (q, *J* = 7.6 Hz, 2H), 1.13 (t, *J* = 7.6 Hz, 3H).

^{13}C {^{1}H} NMR (126 MHz, $CDCl_3$, 25 °C, δ): 154.6, 137.4, 129.0, 114.3, 78.2, 70.1, 28.0, 15.8.

HRMS-ESI(m/z) calc'd for $C_{11}H_{15}O_2$ $[M+H]^+$, 179.1067; found, 179.1067. Deviation: 0 ppm.

2-(2,2,2-Trifluoroethoxy)-9H-xanthen-9-one (34)

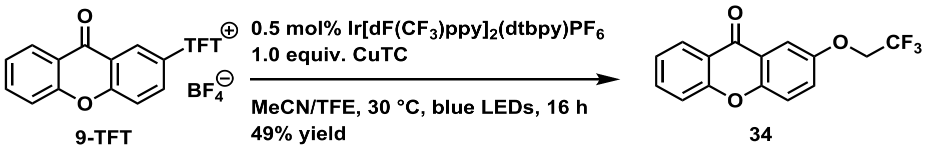

To a 4-mL borosilicate vial, equipped with a magnetic stir bar was added Ir[dF(CF_3)ppy]$_2$(dtbpy)PF_6 (1.1 mg, 1.0 µmol, 0.50 mol%), copper(I) thiophene-2-carboxylate (38.1 mg, 0.200 mol, 1.00 equiv.), and xanthen tetrafluorothianthrenium salt **9-TFT** (114 mg, 0.200 mmol, 1.00 equiv.) at 25 °C. The vial was evacuated and then filled with argon; this procedure was repeated three times. MeCN (1 mL, *c* = 0.2 M) was added, followed by 2,2,2-trifluoroethanol (0.56 g, 0.40 mL, 5.4 mmol, 28 equiv.). The vial was placed in 2 cm distance to two 34 W blue LEDs. The temperature was kept at approximately 30 °C with the use of a cooling fan. The reaction mixture was stirred for 16 hours under blue LED irradiation, and then diluted with methyl-*tert*-butylether (1 mL). The reaction mixture was filtered through a short pad of silica using methyl-*tert*-butylether (20 mL) as eluent. The filtrate was collected and concentrated *in vacuo* and then purified by flash column chromatography on silica gel, eluting with TBME/pentane (1:10, v/v) to afford **34** (28.9 mg, 49% yield) as a colorless solid.

R*f* = 0.45 (EtOAc /pentane, 1:5, v/v (UV))

NMR Spectroscopy:

^{1}H NMR (500 MHz, $CDCl_3$, 25 °C, δ): 8.33 (dd, *J* = 8.0, 1.8 Hz, 1H), 7.84 – 7.66 (m, 2H), 7.57 – 7.46 (m, 2H), 7.44 – 7.34 (m, 2H), 4.47 (q, *J* = 8.0 Hz, 2H).

^{13}C {^{1}H} NMR (126 MHz, $CDCl_3$, 25 °C, δ): 176.7, 156.1, 153.6, 151.8, 134.9, 126.7, 125.2, 124.0, 122.1, 121.2, 120.0, 118.0, 107.4, 77.2, 66.2 (q, *J* = 35.9 Hz).

^{19}F NMR (471 MHz, $CDCl_3$, 25 °C, δ): –73.8.

HRMS-ESI(m/z) calc'd for $C_{15}H_9O_3F_3Na^+$ $[M+Na]^+$, 317.0396; found, 317.0399. Deviation: –0.9 ppm.

Methoxy-PEG(4) ethylbenzene (35)

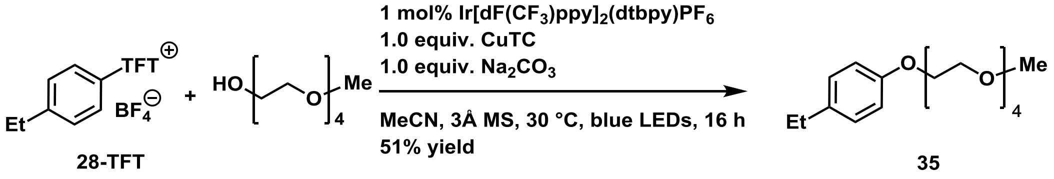

In an anhydrous, N_2-filled glovebox, a 4-mL borosilicate vial equipped with a magnetic stir bar was charged with copper(I) thiophene-2-carboxylate (38.1 mg, 0.200 mmol, 1.00 equiv.), 2,5,8,11-tetraoxatridecan-13-ol (208 mg, 200 µL, 1.00 mmol, 5.00 equiv.), Na_2CO_3 (10.6 mg, 0.100 mmol, 1.00 equiv.), and 3 Å molecular sieves (120 mg). Dry MeCN (1 mL, *c* = 0.2 M) was then added into the vial. The vial was sealed with a Teflon cap. The reaction mixture was stirred at 23 ^{o}C: After 2 hours, the vial was opened and ethyl benzene-derived

tetrafluorothianthrenium salt **28-TFT** (48.0 mg, 0.100 mmol, 1.00 equiv.) and Ir[dF(CF_3)ppy]$_2$(dtbpy)PF_6 (1.1 mg, 1.0 µmol, 1.0 mol%) were added into the reaction mixture. The vial was sealed with the same Teflon cap and transferred out of glovebox. The vial was placed in 2 cm distance to two 34 W blue LEDs. The temperature was kept at approximately 30 °C with the use of a cooling fan. The reaction mixture was stirred for 16 hours under blue LED irradiation, and then diluted with CH_2Cl_2 (2 mL). The reaction mixture was filtered through a short pad of silica using CH_2Cl_2 (25 mL) as eluent. The filtrate was collected and concentrated *in vacuo,* and the residue was then purified by flash column chromatography on silica gel, eluting with EtOAc/pentane (1:1 v/v) to afford **35** (32.4 mg, 51% yield) as a pale yellow oil.

R*f* = 0.30 (EtOAc/pentane, 2:1, v/v (UV, cerium molybdate))

NMR Spectroscopy:

^{1}H NMR (500 MHz, $CDCl_3$, 25 °C, δ): 7.09 (d, *J* = 8.6 Hz, 2H), 6.83 (d, *J* = 8.6 Hz, 2H), 4.10 (dd, *J* = 5.8 Hz, 4.1 Hz, 2H), 3.84 (dd, *J* = 5.8 Hz, 4.1 Hz, 2H), 3.72 (dd, *J* = 5.9 Hz, 3.5 Hz, 2H), 3.68 – 3.62 (m, 8H), 3.54 (dd, *J* = 5.8 Hz, 3.5 Hz, 1H), 3.37 (s, 3H), 2.58 (q, *J* = 7.6 Hz, 2H), 1.20 (t, *J* = 7.6 Hz, 3H).

^{13}C {^{1}H} NMR (126 MHz, $CDCl_3$, 25 °C, δ): 156.8, 136.6, 128.7, 114.5, 71.9, 70.8, 70.6, 70.6, 70.5, 69.8, 67.5, 59.0, 28.0, 15.9.

HRMS-ESI(m/z) calc'd for $C_{17}H_{28}O_5Na$ $[M+Na]^+$, 335.1829; found, 335.1827. Deviation: 0.6 ppm.

2-(4-Ethoxyphenoxy)-6-fluorobenzonitrile (36)

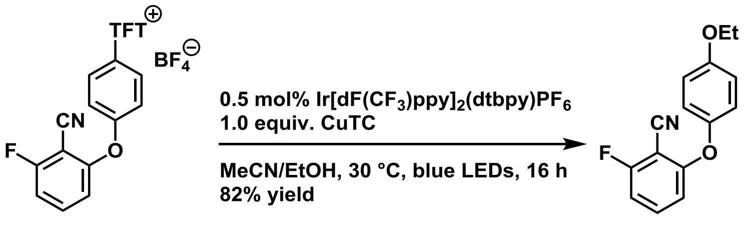

27-TFT **36**

To a 4-mL borosilicate vial, equipped with a magnetic stir bar was added Ir[dF(CF_3)ppy]$_2$(dtbpy)PF_6 (1.1 mg, 1.0 µmol, 0.50 mol%), copper(I) thiophene-2-carboxylate (38.1 mg, 0.200 mol, 1.00 equiv.), and 2-fluoro-6-phenoxybenzonitrile tetrafluorothianthrenium salt **27-TFT** (118 mg, 0.200 mmol, 1.00 equiv.) at 25 °C. The vial was evacuated and then filled with argon; this procedure was repeated three times. MeCN (1 mL, *c* = 0.2 M) was added, followed by EtOH (0.32 g, 0.40 mL, 6.8 mmol, 34 equiv.). The vial was placed in 2 cm distance to two 34 W blue LEDs. The temperature was kept at approximately 30 °C with the use of a cooling fan. The reaction mixture was stirred for 16 hours under blue LED irradiation, and then diluted with methyl-*tert*-butylether (1 mL). The reaction mixture was filtered through a short pad of silica using methyl-*tert*-butylether (20 mL) as eluent. The filtrate was collected and concentrated *in vacuo* and then purified by flash column chromatography on silica gel, eluting with Et_2O/pentane (1:5 v/v) to afford **36** (42.2 mg, 82% yield) as a colorless oil.

R*f* = 0.40 (EtOAc/pentane, 1:10, v/v (UV)).

NMR Spectroscopy:

^{1}H NMR (500 MHz, $CDCl_3$, 25 °C, δ): 7.38 (td, *J* = 8.5 Hz, 6.5 Hz, 1H), 7.05–7.01 (m, 2H), 6.94–6.91 (m, 2H), 6.83 (td, *J* = 8.5 Hz, 0.9 Hz, 1H), 6.53 (dt, *J* = 8.5 Hz, 0.9 Hz, 1H), 4.04 (q, *J* = 7.0 Hz, 2H), 1.43 (t, *J* = 7.0 Hz, 3H).

^{13}C {^{1}H} NMR (126 MHz, $CDCl_3$, 25 °C, δ): 165.3, 163.2, 162.2 (d, *J* = 4.1 Hz), 156.9, 147.5, 134.9 (d, *J* = 10.2 Hz), 121.9, 115.9, 111.4, 111.0 (d, *J* = 3.5 Hz), 109.2 (d, *J* = 19.6 Hz), 64.1, 15.0.

^{19}F NMR (471 MHz, $CDCl_3$, 25 °C, δ): –105.0.

HRMS-ESI(m/z) calc'd for $C_{15}H_{12}FNO_2Na$ $[M+Na]^+$, 280.0744; found, 280.0745. Deviation: –0.4 ppm.

3-(4-Ethylphenoxy)butan-2-ol (37)

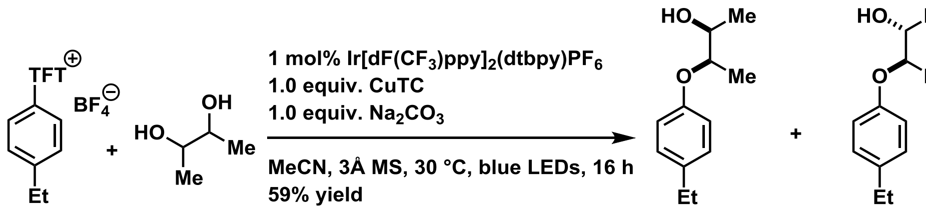

28-TFT **37a** **37b**

In an anhydrous, N_2-filled glovebox, a 4-mL borosilicate vial equipped with a magnetic stir bar was charged with copper(I) thiophene-2-carboxylate (38.1 mg, 0.200 mmol, 1.00 equiv.), 2,3-butandiol (50.1 mg, 50.0 µL, 0.560 mmol, 2.77 equiv.), Na_2CO_3 (10.6 mg, 0.100 mmol, 1.00 equiv.), and 3 Å molecular sieves (120 mg). Dry MeCN (1 mL, *c* = 0.2 M) was then added into the vial. The vial was sealed with a Teflon cap. The reaction mixture was stirred at 23 ^{o}C: After 2 hours, the vial was opened and ethyl benzene-derived tetrafluorothianthrenium salt **28-TFT** (48.0 mg, 0.100 mmol, 1.00 equiv.) and Ir[dF(CF_3)ppy]$_2$(dtbpy)PF_6 (1.1 mg, 1.0 µmol, 1.0 mol%) were added into the reaction mixture. The vial was placed in 2 cm distance to two 34 W blue LEDs. The temperature was kept at approximately 30 °C with the use of a cooling fan. The reaction mixture was stirred for 16 hours under blue LED irradiation, and then diluted with CH_2Cl_2 (2 mL). The reaction mixture was filtered through a short pad of silica using CH_2Cl_2 (25 mL) as eluent. The filtrate was collected and concentrated *in vacuo,* and the residue was then purified by flash column chromatography on silica gel, eluting with EtOAc/pentane (1: 10 v/v) to afford **37a** and **37b** (23.0 mg, **37a**:**37b** = 3:2, 59% yield) as a colorless solid.

R_f = 0.50 (EtOAc/pentane, 1:5, v/v (UV, cerium molybdate))

NMR Spectroscopy (37a):

^{1}H NMR (500 MHz, $CDCl_3$, 25 °C, δ): 7.11 (d, *J* = 8.4 Hz, 2H), 6.84 (d, *J* = 8.5 Hz, 2H), 4.28 (qd, *J* = 6.3 Hz, 3.3 Hz, 1H), 4.02 (qd, *J* = 6.5 Hz, 3.3 Hz, 1H), 2.59 (q, *J* = 7.6 Hz, 2H), 1.26 (d, *J* = 6.3 Hz, 3H), 1.23 (d, *J* = 6.4 Hz, 2H), 1.21 (t, *J* = 7.6 Hz, 3H).

^{13}C {^{1}H} NMR (126 MHz, $CDCl_3$, 25 °C, δ): 155.5, 137.0, 128.8, 116.1, 77.5, 69.4, 28.0, 17.9, 15.8, 13.5.

HRMS-ESI(m/z) calc'd for calc'd for $C_{12}H_{18}O_2$ $[M]^+$, 194.1301; found, 194.1303. Deviation: –0.72 ppm.

NMR Spectroscopy (37b):

^{1}H NMR (300 MHz, $CDCl_3$, 25 °C, δ): 7.11 (d, *J* = 8.5 Hz, 2H), 6.86 (d, *J* = 8.6 Hz, 2H), 4.10 (p, *J* = 6.3 Hz, 1H), 3.82 (p, *J* = 6.4 Hz, 1H), 2.60 (q, *J* = 7.6 Hz, 2H), 1.25 (d, *J* = 3.1 Hz, 3H), 1.23 (d, *J* = 2.9 Hz, 3H), 1.20 (d, *J* = 7.6 Hz, 3H).

^{13}C {^{1}H} NMR (101 MHz, $CDCl_3$, 25 °C, δ): 155.7, 137.1, 128.8, 116.2, 79.2, 71.0, 28.0, 18.5, 15.8, 15.7.

HRMS-ESI(m/z) calc'd for $C_{12}H_{18}O_2Na$ $[M+Na]^+$, 217.1199; found, 217.1199. Deviation: 0.2 ppm.

1-(*tert*-Butyl) 2-methyl (2*S*,4*R*)-4-(4-ethylphenoxy)pyrrolidine-1,2-dicarboxylate (38)

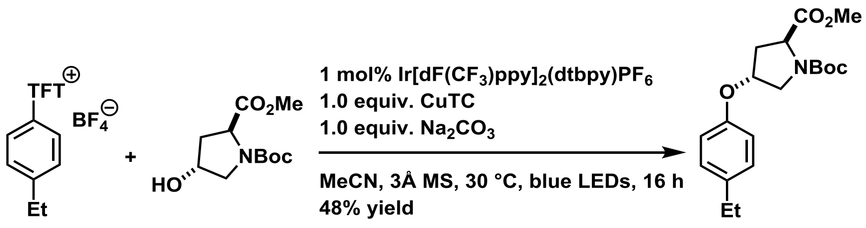

28-TFT **38**

In an anhydrous, N_2-filled glovebox, a 4-mL borosilicate vial equipped with a magnetic stir bar was charged with copper(I) thiophene-2-carboxylate (38.1 mg, 0.200 mmol, 1.00 equiv.), N-Boc-*trans*-4-hydroxy-L-prolin-methylester (105 mg, 0.400 mmol, 2.00 equiv.), Na_2CO_3 (10.6 mg, 0.100 mmol, 1.00 equiv.), and 3 Å molecular sieves (120 mg). Dry MeCN (1 mL, *c* = 0.2 M) was then added into the vial. The vial was sealed with a Teflon cap. The reaction mixture was stirred at 23 ^{o}C: After 2 hours, the vial was opened and ethyl benzene-derived tetrafluorothianthrenium salt **28-TFT** (48.0 mg, 0.100 mmol, 1.00 equiv.) and $Ir[dF(CF_3)ppy]_2(dtbpy)PF_6$ (1.1 mg, 1.0 µmol, 1.0 mol%) were added into the reaction mixture. The vial was sealed with the same Teflon cap and transferred out of glovebox. The vial was placed in 2 cm distance to two 34 W blue LEDs. The temperature was kept at approximately 30 °C with the use of a cooling fan. The reaction mixture was stirred for 16 hours under blue LED irradiation, and then diluted with CH_2Cl_2 (2 mL). The reaction mixture was filtered through a short pad of silica using CH_2Cl_2 (25 mL) as eluent. The filtrate was collected and concentrated *in vacuo,* and the residue was then purified by flash column chromatography on silica gel, eluting with EtOAc/pentane (1:5 v/v) to afford **38** (33.5 mg, 48% yield) as a colorlesssolid.

R_f = 0.30 (EtOAc/pentane, 1:4, v/v (UV, cerium molybdate))

NMR Spectroscopy:

^{1}H NMR (500 MHz, CD_3CN, 25 °C, δ): 7.14 (d, *J* = 8.6 Hz, 2H), 6.81 (d, *J* = 8.6 Hz, 2H), 4.94 – 4.90 (m, 1H), 4.35 (td, *J* = 8.1 Hz, 2.9 Hz, 1H), 3.70, 3.68 (s, 3H), 3.66 – 3.57 (m, 2H), 2.57 (q, *J* = 7.6 Hz, 2H), 2.51 – 2.37 (m, 1H), 2.21 – 2.11 (m, 1H), 1.40, 1.38 (s, 9H), 1.18 (t, *J* = 7.6 Hz, 3H).

^{13}C {^{1}H} NMR (126 MHz, CD_3CN, 25 °C, δ): 173.9, 173.6, 155.5, 154.1, 137.9, 129.5, 116.3, 116.2, 80.3, 76.4, 75.5, 58.5, 58.2, 52.7, 52.3, 52.3, 36.7, 35.9, 28.1, 28.1, 28.0, 15.9.

HRMS-ESI(m/z) calc'd for $C_{19}H_{27}NO_5Na^+$ $[M+Na]^+$, 372.1781; found, 372.1777. Deviation: 1.2 ppm.

(3a*R*,5*R*,6*S*,6a*R*)-5-((*R*)-2,2-Dimethyl-1,3-dioxolan-4-yl)-6-(4-ethylphenoxy)-2,2-dimethyltetrahydro-furo[2,3-d][1,3]dioxole (39)

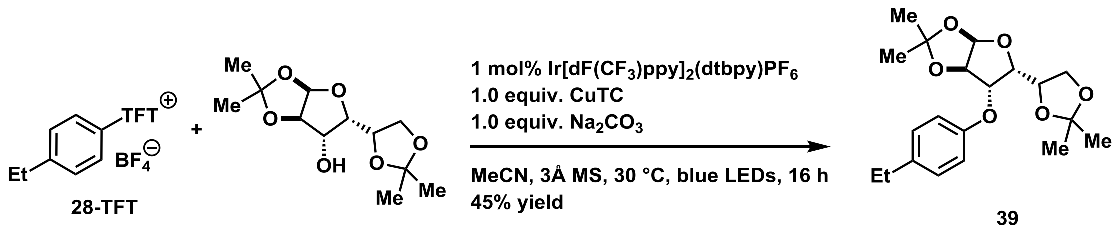

In an anhydrous, N_2-filled glovebox, a 4-mL borosilicate vial equipped with a magnetic stir bar was charged with copper(I) thiophene-2-carboxylate (38.1 mg, 0.200 mmol, 1.00 equiv.), 1,2:5,6-Di-O-isopropyliden-alpha-D-glucofuranose (104 mg, 0.400 mmol, 2.00 equiv.), Na_2CO_3 (10.6 mg, 0.100 mmol, 1.00 equiv.), and 3 Å molecular sieves (120 mg). Dry MeCN (2 mL, *c* = 0.1 M) was then added into the vial. The vial was sealed with a Teflon cap. The reaction mixture was stirred at 23 ^{o}C: After 2 hours, the vial was opened and ethyl benzene-derived tetrafluorothianthrenium salt **28-TFT** (48.0 mg, 0.100 mmol, 1.00 equiv.) and Ir[dF(CF_3)ppy]$_2$(dtbpy)PF_6 (1.1 mg, 1.0 µmol, 1.0 mol%) were added into the reaction mixture. The vial was sealed with the same Teflon cap and transferred out of glovebox. The vial was placed in 2 cm distance to two 34 W blue LEDs. The temperature was kept at approximately 30 °C with the use of a cooling fan. The reaction mixture was stirred for 16 hours under blue LED irradiation, and then diluted with CH_2Cl_2 (2 mL). The reaction mixture was filtered through a short pad of silica using CH_2Cl_2 (25 mL) as eluent. The filtrate was collected and concentrated *in vacuo,* and the residue was then purified by flash column chromatography on silica gel, eluting with EtOAc/pentane (1:20 (v/v)) to afford **39** (32.8 mg, 45% yield) as a colorless solid.

R_f = 0.30 (EtOAc/pentane, 1:10, v/v (UV, cerium molybdate))

NMR Spectroscopy:

^{1}H NMR (500 MHz, $CDCl_3$, 25 °C, δ): 7.14 (d, *J* = 8.6 Hz, 2H), 6.90 (d, *J* = 8.7 Hz, 2H), 5.92 (d, *J* = 3.8 Hz, 1H), 4.69 (d, *J* = 3.1 Hz, 1H), 4.60 (d, *J* = 3.9 Hz, 1H), 4.47 (dt, *J* = 7.3 Hz, 5.8 Hz, 1H), 4.33 (dd, *J* = 7.3 Hz, 3.1 Hz, 1H), 4.20 – 4.08 (m, 2H), 2.60 (q, J = 7.6 Hz, 2H), 1.55 (s, 3H), 1.44 (s, 3H), 1.32 (s, 3H), 1.30 (s, 3H), 1.21 (t, *J* = 7.6 Hz, 3H).

^{13}C {^{1}H} NMR (126 MHz, $CDCl_3$, 25 °C, δ): 154.9, 137.6, 129.0, 115.4, 112.0, 109.1, 105.3, 82.1, 80.5, 79.9, 72.4, 67.0, 28.0, 26.8, 26.7, 26.2, 25.3, 15.9.

HRMS-ESI(m/z) calc'd for $C_{20}H_{28}O_6Na^+$ $[M+Na]^+$, 387.1778; found, 387.1774. Deviation: 1.1 ppm.

4-Methyl-*N*-phenylbenzenesulfonamide tetrafluorothianthrenium salt (40-TFT)

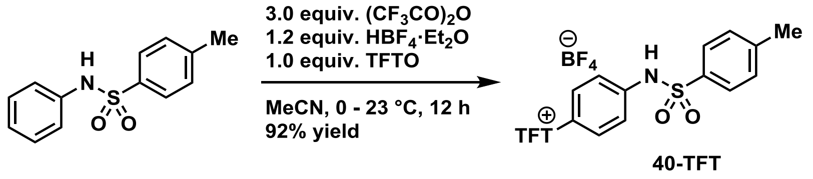

Under an ambient atmosphere, a 20-mL glass vial was charged with 4-methyl-*N*-phenylbenzenesulfonamide (1.24 g, 5.00 mmol, 1.00 equiv), and MeCN (5.0 ml, *c* = 1.0 M). After cooling to 0 C, HBF_4·OEt_2 (0.82 mL, 0.97 g, 6.0 mmol, 1.2 equiv) and tetrafluorothianthrene-*S*-oxide (1.51 g, 5.00 mmol, 1.00 equiv) was added to the vial while stirring the mixture, leading to a suspension. Subsequently, trifluoroacetic anhydride (2.1 mL, 3.1 g, 15 mmol, 3.0 equiv) was added in one portion at 0°C, resulting in a color change to deep purple. Subsequently, the reaction mixture was allowed to reach at 23 ^{o}C and stirred for 12 hours. The solution was diluted with DCM (5 mL) and poured onto a mixture of DCM (30 mL) and saturated aqueous $NaHCO_3$ solution (20 mL). After stirring for 5 min at 23 ^{o}C, the mixture was poured into a separating funnel, and the layers were separated. The DCM layer was washed with aqueous $NaBF_4$ solution (10% w/w, 4 × ca. 20 mL). The DCM layer was dried over Na_2SO_4, filtered, and the solvent was removed under reduced pressure. The residue was purified by chromatography on silica gel eluting with DCM/MeOH (30:1 v/v), then the solvent was removed *in vacuo* to afford **40-TFT** (2.86 g, 92% yield) as a pale yellow powder.

R*f* = 0.35 (MeOH/DCM, 1/15, v/v).

NMR Spectroscopy:

^{1}H NMR (500 MHz, CD_3CN, 25 °C, δ): 8.71 (brs, 1H), 8.33 (dd, *J* = 9.0, 2.0 Hz, 2H), 7.92 (dd, *J* = 10.0, 2.0 Hz, 2H), 7.69–7.69 (m, 2H), 7.31 (d, *J* = 8.0 Hz, 2H), 7.21–7.19 (m, 2H), 7.11–7.08 (m, 2H), 2.36 (s, 3H) ppm.

^{13}C NMR (126 MHz, CD_3CN, 25 °C, δ): 155.3 (dd, *J* = 261.7, 13.2 Hz), 152.1 (dd, *J* = 255.6, 13.4 Hz), 146.6, 144.2, 137.4, 135.6 (dd, *J* = 8.5, 4.0 Hz), 131.6, 131.5, 128.7, 125.8 (dd, *J* = 22.1, 2.4 Hz), 121.7 (d, *J* = 21.9 Hz), 121.3, 117.2, 116.1 (dd, *J* = 7.2, 3.4 Hz), 22.2.

^{19}F NMR (471 MHz, CD_3CN, 25 °C, δ): –125.5 (ddd, *J* = 20.8, 10.2, 7.3 Hz), –133.8 (ddd, *J* = 20.8, 9.2, 7.3 Hz), –151.0 (brs), –151.1 (brs)

HRMS-ESI (m/z) calculated for $C_{25}H_{16}F_4NO_2S_3^+$ $[M-BF_4]^+$, 534.0274; found, 534.0267; Deviation: 1.3 ppm.

N-(4-methoxyphenyl)-4-methylbenzenesulfonamide (40)

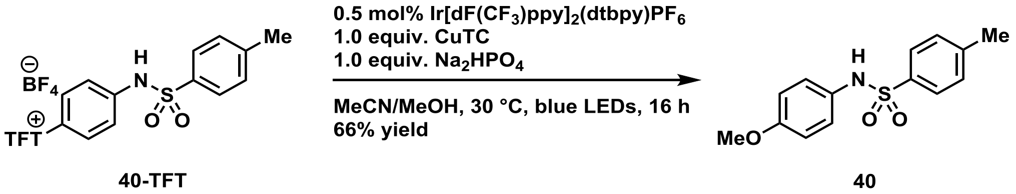

To a 4-mL borosilicate vial, equipped with a magnetic stir bar was added Ir[dF(CF_3)ppy]$_2$(dtbpy)PF_6 (1.1 mg, 1.0 µmol, 0.50 mol%), copper(I) thiophene-2-carboxylate (38.1 mg, 0.200 mol, 1.00 equiv.), 4-methyl-N-phenylbenzenesulfonamide tetrafluorothianthrenium salt **40-TFT** (124 mg, 0.200 mmol, 1.00 equiv.), and Na_2HPO_4 (28.4 mg, 0.200 mmol, 1.00 equiv.) at 25 °C. The vial was evacuated and then filled with argon; this procedure was repeated three times. MeCN (1 mL, *c* = 0.2 M) was added, followed by MeOH (0.16 g, 0.20 mL, 4.9 mmol, 25 equiv.). The vial was placed in 2 cm distance to two 34 W blue LEDs. The temperature was kept at approximately 30 °C with the use of a cooling fan. The reaction mixture was stirred for 16 hours under blue LED irradiation, and then diluted with methyl-*tert*-butylether (1 mL). The reaction mixture was filtered through a short pad of silica using methyl-*tert*-butylether (20 mL) as eluent. The filtrate was collected and concentrated *in vacuo* and then purified by flash column chromatography on silica gel, eluting with TBME/pentane (1:5, v/v) to afford **40** (36.6 mg, 66% yield) as a colorless solid.

R*f* = 0.57 (EtOAc/pentane,1:1, v/v (UV, cerium molybdate)).

NMR Spectroscopy:

^{1}H NMR (500 MHz, CD_2Cl_2, 25 °C, δ): 7.57 (d, *J* = 8.1 Hz, 2H), 7.24 (d, *J* = 8.0 Hz, 2H), 7.04 – 6.88 (m, 2H), 6.83 – 6.73 (m, 2H), 6.60 (s, 1H), 3.74 (d, *J* = 0.9 Hz, 3H), 2.38 (s, 3H).

^{13}C {^{1}H} NMR (126 MHz, CD_2Cl_2, 25 °C, δ): 158.53, 144.52, 136.54, 130.1, 129.6, 127.8, 125.7, 114.9, 55.9, 21.8.

HRMS-ESI(m/z) calc'd for $C_{14}H_{15}NO_3SNa^+$ $[M+Na]^+$, 300.0665; found, 300.0666. Deviation: – 0.3 ppm.

tert-Butyl 3-(4-((benzoyloxy)methyl)phenoxy)azetidine-1-carboxylate (41)

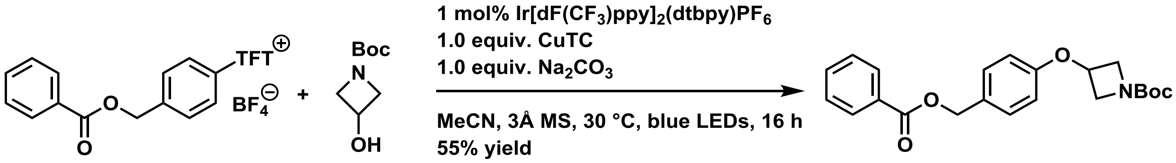

In an anhydrous, N_2-filled glovebox, a 4-mL borosilicate vial equipped with a magnetic stir bar was charged with copper(I) thiophene-2-carboxylate (38.1 mg, 0.200 mmol, 1.00 equiv.), N-Boc-3-hydroxyazetidin (69.2 mg, 0.400 mmol, 2.00 equiv.), Na_2CO_3 (10.6 mg, 0.100 mmol, 1.00 equiv.), and 3 Å molecular sieves (120 mg). Dry MeCN (1 mL, *c* = 0.2 M) was then added into the vial. The vial was sealed with a Teflon cap. The reaction mixture was stirred at 23 ^{o}C: After 2 hours, the vial was opened and ethyl benzene-derived tetrafluorothianthrenium salt **28-TFT** (48.0 mg, 0.100 mmol, 1.00 equiv.) and Ir[dF(CF_3)ppy]$_2$(dtbpy)PF_6 (1.1 mg, 1.0 µmol, 1.0 mol%) were added into the reaction mixture. The vial was sealed with the same Teflon cap and transferred out of glovebox. The vial was placed in 2 cm distance to two 34 W blue LEDs. The temperature was kept at approximately 30 °C with the use of a cooling fan. The reaction mixture was stirred for 16 hours under blue LED irradiation, and then diluted with CH_2Cl_2 (2 mL). The reaction mixture was filtered through a short pad of silica using CH_2Cl_2 (25 mL) as eluent. The filtrate was collected and concentrated *in vacuo,* and the residue was then purified by flash column chromatography on silica gel,

eluting with EtOAc/pentane (1:8 v/v) to afford **41** (42.0 mg, 55% yield) as a colorless solid.

R*f* = 0.40 (EtOAc/pentane, 1:4, v/v (UV))

NMR Spectroscopy:

^{1}H NMR (500 MHz, CD_3CN, 25 °C, δ): 8.00 (dd, *J* = 8.4 Hz, 1.4 Hz, 2H), 7.61 (ddt, *J* = 8.8 Hz, 7.1 Hz, 1.3 Hz, 1H), 7.48 (dd, *J* = 8.2 Hz, 7.4 Hz, 2H), 7.41 (d, *J* = 8.7 Hz, 2H), 6.80 (d, *J* = 8.6 Hz, 2H), 5.27 (s, 2H), 4.92 (tt, *J* = 6.4 Hz, 4.0 Hz, 1H), 4.26 (dd, *J* = 9.6 Hz, 6.4 Hz, 2H), 3.85 (dd, *J* = 9.8 Hz, 3.9 Hz, 2H), 1.41 (s, 9H).

^{13}C {^{1}H} NMR (126 MHz, CD_3CN, 25 °C, δ): 167.0, 157.7, 157.1, 134.1, 131.2, 131.1, 130.3, 130.2, 129.6, 115.6, 80.0, 67.1, 66.9, 57.1, 28.5.

HRMS-ESI(m/z) calc'd for $C_{22}H_{25}NO_5Na^+$ [M+Na]$^+$,406.1625; found, 406.1619. Deviation: 1.4 ppm.

tert-Butyl (*R*)-3-(4-((2-oxo-3-propionyloxazolidin-4-yl)methyl)phenoxy)azetidine-1-carboxylate (42)

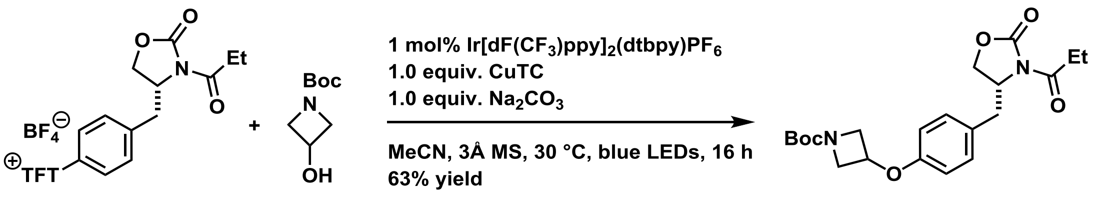

10-TFT 42

In an anhydrous, N_2-filled glovebox, a 4-mL borosilicate vial equipped with a magnetic stir bar was charged with copper(I) thiophene-2-carboxylate (38.1 mg, 0.200 mmol, 1.00 equiv.), N-Boc-3-hydroxyazetidin (69.2 mg, 0.400 mmol, 2.00 equiv.), Na_2CO_3 (10.6 mg, 0.100 mmol, 1.00 equiv.), and 4 Å molecular sieves (120 mg). Dry MeCN (1 mL, *c* = 0.2 M) was then added into the vial. The vial was sealed with a Teflon cap. The reaction mixture was stirred at 23 °C: After 2 hours, the vial was opened and (*R*)-4-(4-hydroxybenzyl)-3-propionyloxazolidin-2-one tetrafluoro-thianthrenium salt **10-TFT** (121 mg, 0.200 mmol, 1.00 equiv.) and Ir[dF(CF_3)ppy]$_2$(dtbpy)PF_6 (2.2 mg, 2.0 µmol, 1.0 mol%) were added into the reaction mixture. The vial was sealed with the same Teflon cap and transferred out of glovebox. The vial was placed in 2 cm distance to two 34 W blue LEDs. The temperature was kept at approximately 30 °C with the use of a cooling fan. The reaction mixture was stirred for 16 hours under blue LED irradiation, and then diluted with CH_2Cl_2 (2 mL). The reaction mixture was filtered through a short pad of silica using CH_2Cl_2 (25 mL) as eluent. The filtrate was collected and concentrated *in vacuo,* and the residue was then purified by flash column chromatography on silica gel, eluting with EtOAc/pentane (1:2 v/v) to afford **42** (51.0 mg, 63% yield) as a colorless solid.

R*f* = 0.40 (EtOAc/pentane, 1:2, v/v (cerium molybdate))

NMR Spectroscopy:

^{1}H NMR (500 MHz, CD_3CN, 25 °C, δ): 7.12 (d, *J* = 8.6 Hz, 2H), 6.73 (d, *J* = 8.6 Hz, 2H), 4.89 (tt, J = 6.5 Hz, 4.0 Hz, 1H), 4.62 (tt, J = 7.9 Hz, 3.1 Hz, 1H), 4.26 – 4.22 (m, 3H), 4.13 (dd, *J* = 9.0 Hz, 2.8 Hz, 1H), 3.82 (dd, *J* = 10.3 Hz, 4.0 Hz, 2H), 2.99 (dd, *J* = 13.8 Hz, 3.4 Hz, 1H), 2.95 – 2.77 (m, 3H), 1.41 (s, 9H),

1.12 (t, J = 7.4 Hz, 3H).

^{13}C {^{1}H} NMR (126 MHz, CD_3CN, 25 °C, δ): 174.3, 156.7, 156.4, 154.3, 131.5, 129.3, 115.3, 79.6, 66.8, 66.5, 56.59, 55.2, 36.7, 29.3, 28.1, 8.3.

HRMS-ESI(m/z) calc'd for $C_{21}H_{28}N_2O_6Na^+$ $[M+Na]^+$, 427.1840; found, 427.1833. Deviation: 1.6 ppm.

4-Methoxy-flurbiprofen methyl ester (43).

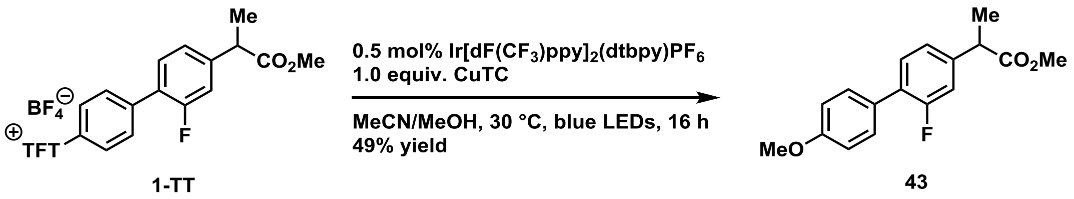

To a 4-mL borosilicate vial, equipped with a magnetic stir bar was added $Ir[dF(CF_3)ppy]_2(dtbpy)PF_6$ (1.1 mg, 1.0 µmol, 0.50 mol%), copper(I) thiophene-2-carboxylate (38 mg, 40 µmol, 1.0 equiv.), and flurbiprofen methyl ester-derived thianthrenium salt **1-TT** (112 mg, 0.200 mmol, 1.00 equiv.) at 25 °C. The vial was evacuated and then filled with argon; this procedure was repeated three times. MeCN (1 mL, c = 0.2 M) was added, followed by MeOH (128 mg, 162 µL, 4.00 mmol, 20.0 equiv.). The vial was placed in 2 cm distance to two 34 W blue LEDs. The temperature was kept at approximately 30 °C with the use of a cooling fan. The reaction mixture was stirred for 16 hours under blue LED irradiation, and then diluted with methyl-*tert*-butylether (1 mL). The reaction mixture was filtered through a short pad of silica using methyl-*tert*-butylether (20 mL) as eluent. The filtrate was collected and concentrated *in vacuo* and then purified by flash column chromatography on silica gel, eluting with Et_2O/pentane(1:20 v/v) to afford **43** (27.9 mg, 49% yield) as a colorless oil.

R*f* = 0.30 (Et_2O/pentane, 1:10,.v/v (UV, cerium molybdate))

NMR Spectroscopy:

^{1}H NMR (500 MHz, $CDCl_3$, 25 °C, δ): 7.48–7.46 (m, 2H), 7.36 (t, J = 8.1 Hz, 1H), 7.13–7.09 (m, 2H), 6.99–6.96 (m, 2H), 3.85 (s, 3H), 3.77–3.73 (m, 1H), 3.70 (s, 3H), 1.53 (d, J = 7.1 Hz, 3H).

^{13}C {^{1}H} NMR (126 MHz, $CDCl_3$, 25 °C , δ): 174.6, 159.8 (d, J = 247.4 Hz), 159.4, 141.3 (d, J = 7.7 Hz), 130.7 (d, J = 4.1 Hz), 129.7, 130.19, 130.17, 128.0, 127.6 (d, J = 13.6 Hz), 123.6 (d, J = 3.2 Hz), 115.3 (d, J = 23.8 Hz), 114.1, 55.4, 52.4, 45.0, 18.6.

^{19}F NMR (471 MHz, $CDCl_3$, 25 °C, δ): –117.8.

HRMS-ESI(m/z) calc'd for $C_{17}H_{17}O_3FNa^+$ $[M+Na]^+$, 311.1054; found, 311.1054. Deviation: –0.2 ppm.

tert-Butyl 3-(3-formyl-4-methoxyphenoxy)azetidine-1-carboxylate (44)

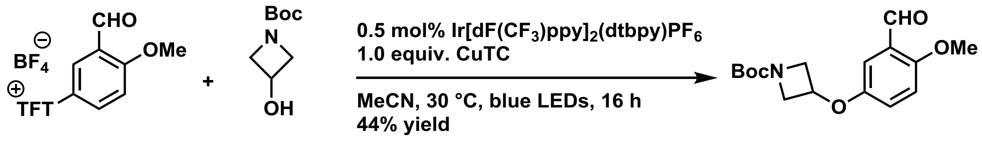

3-TFT **44**

To a 4-mL borosilicate vial, equipped with a magnetic stir bar was added Ir[dF(CF_3)ppy]$_2$(dtbpy)PF_6 (1.1 mg, 1.0 µmol, 0.50 mol%), copper(I) thiophene-2-carboxylate (38.1 mg, 0.200 mol, 1.00 equiv.), N-Boc-3-hydroxyazetidin (173 mg, 1.00 mmol, 5.00 equiv.), and 2-methoxybenzaldehyde tetrafluorothianthrenium salt **3-TFT** (102 mg, 0.200 mmol, 1.00 equiv.) at 25 °C. The vial was evacuated and then filled with argon; this procedure was repeated three times. MeCN (1 mL, *c* = 0.2 M) was added. The vial was placed in 2 cm distance to two 34 W blue LEDs. The temperature was kept at approximately 30 °C with the use of a cooling fan. The reaction mixture was stirred for 16 hours under blue LED irradiation, and then diluted with methyl-*tert*-butylether (1 mL). The reaction mixture was filtered through a short pad of silica using methyl-*tert*-butylether (20 mL) as eluent. The filtrate was collected and concentrated *in vacuo* and then purified by flash column chromatography on silica gel, eluting with TBME/pentane (1:2, v/v) to afford **44** (27.3 mg, 44% yield) as a colorless solid.

R*f* = 0.56 (EtOAc/pentane, 1:2, v/v (UV, cerium molybdate)).

NMR Spectroscopy:

^{1}H NMR (500 MHz, $CDCl_3$, 25 °C, δ): 10.42 (s, 1H), 7.09–7.06 (m, 2H), 6.97–6.95 (m, 1H), 4.86 (tt, *J* = 6.4 Hz, 4.1 Hz, 1H), 4.30 (ddd, *J* = 9.6 Hz, 6.4 Hz, 1.1 Hz, 2H), 3.96 (ddd, *J* = 9.6 Hz, 4.1 Hz, 1.1 Hz, 2H), 3.90 (s, 3H), 1.44 (s, 9H).

^{13}C {^{1}H} NMR (126 MHz, $CDCl_3$, 25 °C, δ): 189.2, 157.2, 156.1, 150.5, 125.0, 124.0, 113.7, 111.3, 79.9, 66.2, 56.3, 56.2, 28.4.

HRMS-ESI(m/z) calc'd for $C_{16}H_{21}O_5NNa^+$ [M+Na]$^+$, 330.1312; found, 330.1312.Deviation: 0 ppm.

tert-Butyl 3-(5-((3r,5r,7r)-adamantan-1-yl)-2-methylphenoxy)azetidine-1-carboxylate (45)

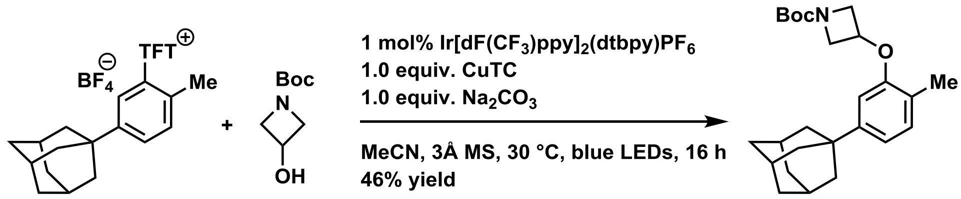

23-TFT **45**

In an anhydrous, N_2-filled glovebox, a 4-mL borosilicate vial equipped with a magnetic stir bar was charged with copper(I) thiophene-2-carboxylate (38.1 mg, 0.200 mmol, 1.00 equiv.), N-Boc-3-hydroxyazetidin (69.2 mg, 0.400 mmol, 2.00 equiv.), Na_2CO_3 (10.6 mg, 0.100 mmol, 1.00 equiv.), and 3 Å molecular sieves (120 mg). Dry MeCN (1 mL, *c* = 0.2 M) was then added into the vial. The vial was sealed with a Teflon cap. The

reaction mixture was stirred at 23 °C: After 2 hours, the vial was opened and (3r,5r,7r)-1-(p-tolyl)adamantane derived-tetrafluorothianthrenium salt **23-TFT** (120 mg, 0.200 mmol, 1.00 equiv.) and Ir[dF(CF_3)ppy]$_2$(dtbpy)PF_6 (2.2 mg, 2.0 µmol, 1.0 mol%) were added into the reaction mixture. The vial was sealed with the same Teflon cap and transferred out of glovebox. The vial was placed in 2 cm distance to two 34 W blue LEDs. The temperature was kept at approximately 30 °C with the use of a cooling fan. The reaction mixture was stirred for 16 hours under blue LED irradiation, and then diluted with CH_2Cl_2 (2 mL). The reaction mixture was filtered through a short pad of silica using CH_2Cl_2 (25 mL) as eluent. The filtrate was collected and concentrated *in vacuo,* and the residue was then purified by flash column chromatography on silica gel, eluting with EtOAc/pentane (1:20 v/v) to afford **45** (36.5 mg, 46% yield) as a colorless solid.

R$_f$ = 0.30 (EtOAc/pentane (1:20 v/v))

NMR Spectroscopy:

^{1}H NMR (500 MHz, $CDCl_3$, 25 °C, δ): 7.11 (d, *J* = 7.8 Hz, 1H), 6.90 (dd, *J* = 7.9 Hz, 1.8 Hz, 1H), 6.46 (d, *J* = 1.8 Hz, 1H), 4.92 (tt, *J* = 6.5 Hz, 4.3 Hz, 1H), 4.33 (dd, *J* = 9.6 Hz, 6.5 Hz, 2H), 4.03 (dd, *J* = 9.8 Hz, 4.3 Hz, 2H), 2.21 (s, 3H), 2.12 – 2.05 (m, 3H), 1.87 (d, *J* = 2.9 Hz, 6H), 1.82 – 1.70 (m, 6H), 1.47 (s, 9H).

^{13}C {^{1}H} NMR (126 MHz, $CDCl_3$, 25 °C, δ): 156.4, 154.7, 150.7, 130.8, 124.1, 117.7, 107.9, 79.9, 65.6, 56.8, 43.5, 36.9, 36.2, 29.1, 28.5, 15.8.

HRMS-ESI(m/z) calc'd for $C_{25}H_{35}NO_3Na^+$ [M+Na]$^+$, 420.2509; found, 420.2502. Deviation: 1.7 ppm.

tert-Butyl 3-(4-(3-chloropropyl)phenoxy)azetidine-1-carboxylate (46)

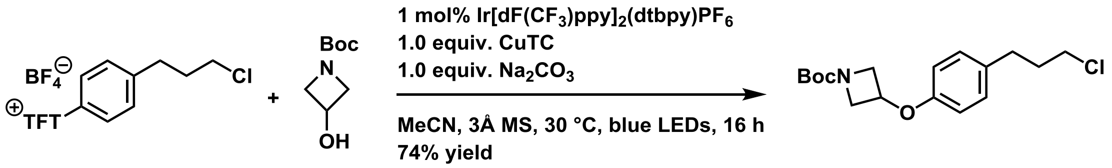

14-TFT **46**

In an anhydrous, N_2-filled glovebox, a 4-mL borosilicate vial equipped with a magnetic stir bar was charged with copper(I) thiophene-2-carboxylate (38.1 mg, 0.200 mmol, 0.800 equiv.), N-Boc-3-hydroxyazetidin (76.2 mg, 0.440 mmol, 1.76 equiv.), Na_2CO_3 (10.6 mg, 0.100 mmol, 0.800 equiv.), and 4 Å molecular sieves (120 mg). Dry MeCN (1 mL, *c* = 0.2 M) was then added into the vial. The vial was sealed with a Teflon cap. The reaction mixture was stirred at 23 °C: After 2 hours, the vial was opened and 3-chloropropyl benzene derived-tetrafluorothianthrenium salt **14-TFT** (132 mg, 0.250 mmol, 1.00 equiv.) and Ir[dF(CF_3)ppy]$_2$(dtbpy)PF_6 (2.2 mg, 2.0 µmol, 0.80 mol%) were added into the reaction mixture. The vial was sealed with the same Teflon cap and transferred out of glovebox. The vial was placed in 2 cm distance to two 34 W blue LEDs. The temperature was kept at approximately 30 °C with the use of a cooling fan. The reaction mixture was stirred for 16 hours under blue LED irradiation, and then diluted with CH_2Cl_2 (2 mL). The reaction mixture was filtered through a short pad of silica using CH_2Cl_2 (25 mL) as eluent. The filtrate was collected and concentrated *in vacuo,* and the residue was then purified by flash column chromatography on silica gel, eluting with EtOAc/pentane (1:8 v/v) to afford **46** (61.0 mg, 74% yield) as a colorless solid.

R*f* = 0.30 (EtOAc/pentane, 1:8, v/v (UV, cerium molybdate))

NMR Spectroscopy:

^{1}H NMR (300 MHz, CD_3CN, 25 °C, δ): 7.14 (d, *J* = 8.8 Hz, 2H), 6.72 (d, *J* = 8.6 Hz, 2H), 4.89 (tt, *J* = 6.4 Hz, 4.0 Hz, 1H), 4.25 (ddd, *J* = 9.6 Hz, 6.4 Hz, 1.1 Hz, 2H), 3.83 (ddd, *J* = 9.5 Hz, 4.0 Hz, 1.1 Hz, 2H), 3.55 (t, *J* = 6.6 Hz, 2H), 2.68 (dd, *J* = 8.5 Hz, 6.6 Hz, 2H), 2.07 – 1.96 (m, 2H), 1.42 (s, 9H).

^{13}C {^{1}H} NMR (75 MHz, CD_3CN, 25 °C, δ): 157.1, 156.1, 135.0, 130.7, 115.6, 80.0, 66.9, 66.9, 57.29, 45.4, 35.1, 32.5, 28.5.

HRMS-ESI(m/z) calc'd for $C_{17}H_{24}NO_3ClNa^+$ $[M+Na]^+$, 348.1337; found, 348.1334. Deviation: 0.9 ppm.

5-Oxetan-3-yloxy boscalid (47)

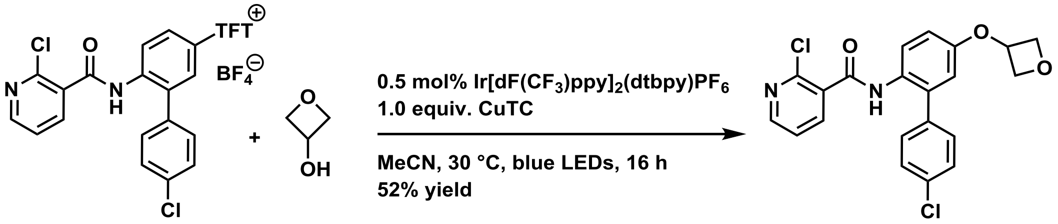

11-TFT **47**

To a 4-mL borosilicate vial, equipped with a magnetic stir bar was added $Ir[dF(CF_3)ppy]_2(dtbpy)PF_6$ (1.1 mg, 1.0 µmol, 0.50 mol%), copper(I) thiophene-2-carboxylate (38 mg, 40 µmol, 1.0 equiv.), 3-hydroxyoxetane (74.0 mg, 63.5 µL, 1.00 mmol, 5.00 equiv.), and boscalid derived-tetrafluorothianthrenium salt **11-TFT** (144 mg, 0.200 mmol, 1.00 equiv.) at 25 °C. The vial was evacuated and then filled with argon; this procedure was repeated three times. MeCN (1 mL, *c* = 0.2 M) was added. The vial was placed in 2 cm distance to two 34 W blue LEDs. The temperature was kept at approximately 30 °C with the use of a cooling fan. The reaction mixture was stirred for 16 hours under blue LED irradiation, and then diluted with methyl-*tert*-butylether (1 mL). The reaction mixture was filtered through a short pad of silica using methyl-*tert*-butylether (20 mL) as eluent. The filtrate was collected and concentrated *in vacuo* and then purified by flash column chromatography on silica gel, eluting with EtOAc/DCM (2:15, v/v) to afford **47** (42.8 mg, 52% yield) as a colorless solid.

R*f* = 0.36 (EtOAc/pentane, 1:2, v/v (UV)).

NMR Spectroscopy:

^{1}H NMR (500 MHz, $CDCl_3$, 25 °C, δ): 8.44 (d, *J* = 2.7 Hz, 1H), 8.19 (d, *J* = 9.0 Hz, 1H), 8.12 (dd, *J* = 7.7 Hz, 2.0 Hz, 1H), 7.99 (bs, 1H), 7.44–7.32 (m, 2H), 7.36–7.30 (m, 3H), 6.75 (dd, *J* = 8.9 Hz, 2.9 Hz, 1H), 6.65 (d, *J* = 2.9 Hz, 1H), 5.26–5.21 (m, 1H), 4.99–4.96 (m, 2H), 4.79–4.76 (m, 2H).

^{13}C {^{1}H} NMR (126 MHz, $CDCl_3$, 25 °C , δ): 162.7, 154.1, 151.4, 146.9, 140.3, 136.1, 134.9, 134.8, 131.1, 130.7, 129.5, 128.3, 124.7, 123.1, 116.6, 114.3, 78.0, 70.6.

HRMS-ESI(m/z) calc'd for $C_{21}H_{16}O_3N_2Cl_2Na^+$ $[M+Na]^+$, 437.0430; found, 437.0434. Deviation: –0.9 ppm.

4-(Cyclohexylthio)-1,1'-biphenyl (48)

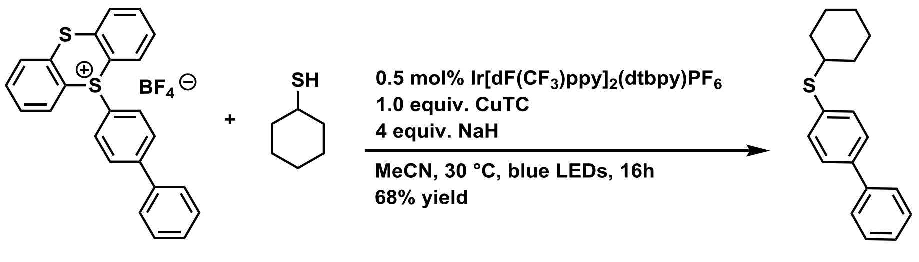

To a 4-mL borosilicate vial, equipped with a magnetic stir bar was added Ir[dF(CF_3)ppy]$_2$(dtbpy)PF_6 (1.1 mg, 1.0 µmol, 0.50 mol%), copper(I) thiophene-2-carboxylate (38 mg, 40 µmol, 1.0 equiv.), NaH (c = 60 % dispersion in mineral oil; 36 mg, 0.80 mmol, 4.0 equiv.), and biphenyl thianthrenium salt **22-TT** (91.3 mg, 0.200 mmol, 1.00 equiv.) at 25 °C. The vial was evacuated and then filled with argon; this procedure was repeated three times. MeCN (1 mL, *c* = 0.2 M) was added, followed by cyclohexanethiol (116 mg, 48.9 µL, 0.400 mmol, 2.00 equiv.). The vial was placed in 2 cm distance to two 34 W blue LEDs. The temperature was kept at approximately 30 °C with the use of a cooling fan. The reaction mixture was stirred for 16 hours under blue LED irradiation, then diluted with ethyl acetate (1 mL), and filtered through a short pad of silica using ethyl acetate (20 mL) as eluent. The filtrate was collected and concentrated *in vacuo* and then purified by flash column chromatography on silica gel, eluting with ethyl acetate/pentane 1:200 (v/v) to afford **48** (41.8 mg, 68% yield) as a colorless solid.

R*f* = 0.30 (ethyl acetate/pentane, 1:200, v/v (UV))

NMR Spectroscopy:

^{1}H NMR (500 MHz, $CDCl_3$, 25 °C, δ): 7.58 (d, *J* = 8.3 Hz, 2H), 7.53 (d, *J* = 8.3 Hz, 2H), 7.49 – 7.40 (m, 4H), 7.35 (dd, *J* = 8.3, 6.4 Hz, 1H), 3.25 – 3.10 (m, 1H), 2.12 – 1.97 (m, 2H), 1.80 (dt, *J* = 13.0, 3.8 Hz, 2H), 1.64 (dt, *J* = 12.4, 3.8 Hz, 1H), 1.52 – 1.26 (m, 5H).

^{13}C {^{1}H} NMR (126 MHz, $CDCl_3$, 25 °C , δ): 140.5, 139.5, 134.3, 132.1, 128.8, 127.4, 127.3, 126.9, 46.6, 33.4, 26.1, 25.8.

HRMS-ESI(m/z) calc'd for $C_{18}H_{20}S^+$ [M]$^+$, 268.1285; found, 268.1280. Deviation: –1.7 ppm.

4-Fluoro-1,1'-biphenyl thianthrenium salt (49-TT)

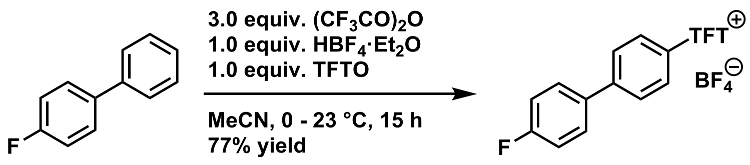

Under an ambient atmosphere, a 20-mL glass vial was charged with 4-fluoro-1,1'-biphenyl (516 mg, 3.00 mmol, 1.00 equiv) and MeCN (11 mL, *c* = 0.24 M). After cooling to 0 °C, HBF_4·OEt_2 (0.40 mL, 0.49 g,

3.0 mmol, 1.0 equiv) and thianthrene-*S*-oxide (912 mg, 3.00 mmol, 1.00 equiv) was added to the vial while stirring the mixture, leading to a suspension. Subsequently, trifluoroacetic anhydride (1.3 mL, 1.8 g, 9.0 mmol, 3.0 equiv) was added in one portion at 0 °C, resulting in a color change to deep purple. Subsequently, the reaction mixture was allowed to reach 23 °C and stirred for 15 hours. The solution was diluted with DCM (5 mL) and poured onto a mixture of DCM (30 mL) and saturated aqueous $NaHCO_3$ solution (20 mL). After stirring for 5 min at 23 °C, the mixture was poured into a separating funnel, and the layers were separated. The DCM layer was washed with aqueous $NaBF_4$ solution (10% w/w, 4 × ca. 20 mL). The DCM layer was dried over Na_2SO_4, filtered, and the solvent was removed under reduced pressure. The residue was purified by chromatography on silica gel eluting with DCM/MeOH (30:1 (v/v)), then the solvent was removed *in vacuo* to afford **49-TT** (1.1 g, 77% yield) as a colorless solid.

R*f* = 0.35 (DCM/MeOH, 15:1, v/v).

NMR Spectroscopy:

^{1}H NMR (500 MHz, CD_3CN, 25 °C, δ): 8.40 (dd, *J* = 8.0, 1.4 Hz, 2H), 7.98 (dd, *J* = 7.9, 1.4 Hz, 2H), 7.91 (td, *J* = 7.7, 1.4 Hz, 2H), 7.84 (td, *J* = 7.7, 1.4 Hz, 2H), 7.74 – 7.67 (m, 2H), 7.67 – 7.59 (m, 2H), 7.24 – 7.14 (m, 4H).

^{13}C {^{1}H} NMR (126 MHz, CD_3CN, 25 °C, δ): 164.2 (d, *J* = 247.0 Hz), 145.3, 137.5, 136.1 (d, *J* = 16.6 Hz), 135.3 (d, *J* = 3.1 Hz), 131.6, 130.9, 130.3 (d, *J* = 8.4 Hz), 129.6 (d, *J* = 16.9 Hz), 123.3, 119.4, 116.9 (d, *J* = 21.6 Hz).

^{19}F NMR (471 MHz, CD_3CN, 25 °C, δ): –114.8 (dd, *J* = 9.3, 5.3 Hz), –151.7 (brs), –151.8 (brs).

HRMS-ESI(m/z) calc'd for $C_{24}H_{16}FS_2^+$ $[M-BF_4]^+$, 387.0672; found, 387.0665; deviation: 1.8 ppm.

Cyclohexyl(4'-fluoro-[1,1'-biphenyl]-4-yl)sulfane (49)

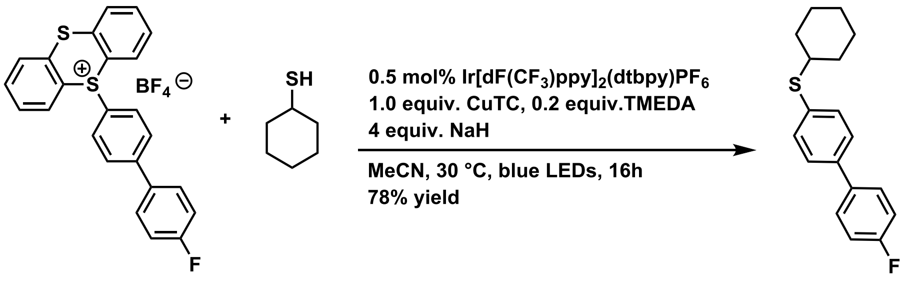

49-TT 49

To a 4-mL borosilicate vial, equipped with a magnetic stir bar was added $Ir[dF(CF_3)ppy]_2(dtbpy)PF_6$ (1.1 mg, 1.0 µmol, 0.50 mol%), copper(I) thiophene-2-carboxylate (38 mg, 0.20 mmol, 1.0 equiv.), NaH (*c* = 60 % dispersion in mineral oil; 36 mg, 0.80 mmol,4.0 equiv.), and 4-fluoro-1,1'-biphenyl thianthrenium salt **49-TT** (94.8 mg, 0.200 mmol, 1.00 equiv.) at 25 °C. The vial was evacuated and then filled with argon; this procedure was repeated three times. MeCN (1 mL, *c* = 0.2 M) was added, followed by 1,2-bis(dimethylamino)ethane (5 mg, 6 µL, 0.04mmol, 0.2 equiv.) and cyclohexanethiol (116 mg, 48.9 µL, 0.400 mmol, 2.00 equiv.). The vial was placed in 2 cm distance to two 34 W blue LEDs. The temperature was kept

at approximately 30 °C with the use of a cooling fan. The reaction mixture was stirred for 16 hours under blue LED irradiation, then diluted with ethyl acetate (1 mL), and filtered through a short pad of silica using ethyl acetate (20 mL) as eluent. The filtrate was collected and concentrated *in vacuo* and then purified by flash column chromatography on silica gel, eluting with pentane to afford **49** (44.6 mg, 78% yield) as a colorless solid.

R*f* = 0.30 (EtOAc/pentane, 1:200, v/v (UV))

NMR Spectroscopy:

^{1}H NMR (500 MHz, $CDCl_3$, 25 °C, δ): 7.53 (dd, *J* = 8.6, 5.4 Hz, 2H), 7.46 (d, *J* = 1.1 Hz, 4H), 7.12 (t, *J* = 8.6 Hz, 2H), 3.15 (tt, J = 10.6 Hz, 3.7 Hz, 1H), 2.09 – 1.95 (m, 2H), 1.80 (dt, *J* = 12.8 Hz, 3.9 Hz, 2H), 1.63 (dd, *J* = 11.6 Hz, 4.8 Hz, 1H), 1.47 – 1.23 (m, 5H).

^{13}C {^{1}H} NMR (126 MHz, $CDCl_3$, 25 °C , δ): 162.5 (d, *J* = 246.2 Hz), 138.5, 136.6 (d, *J* = 3.3 Hz), 134.4, 132.1, 128.5 (d, *J* = 8.0 Hz), 127.3, 115.7 (d, *J* = 21.4 Hz), 46.6, 33.4, 26.1, 25.8.

^{19}F NMR (471 MHz, $CDCl_3$, 25 °C, δ): –115.5.

HRMS-ESI(m/z) calc'd for $C_{18}H_{19}SF^+$ $[M]^+$, 286.1186; found, 286.1184. Deviation: 0.7 ppm.

2-Methoxy-5-(phenylthio)benzonitrile (50)

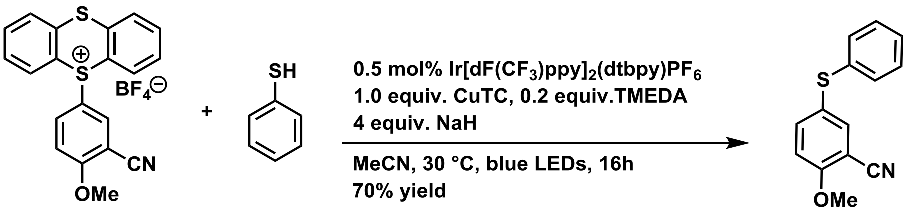

13-TT 50

To a 4-mL borosilicate vial, equipped with a magnetic stir bar was added $Ir[dF(CF_3)ppy]_2(dtbpy)PF_6$ (1.1 mg, 1.0 µmol, 0.50 mol%), copper(I) thiophene-2-carboxylate (38 mg, 0.20 mmol, 1.0 equiv.), NaH (*c* = 60 % dispersion in mineral oil; 36 mg, 0.80 mmol,4.0 equiv.), and (2-methoxy-5-methylbenzonitrile thianthrenium salt **13-TT** (87.0 mg, 0.200 mmol, 1.00 equiv.) at 25 °C. The vial was evacuated and then filled with argon; this procedure was repeated three times. MeCN (1 mL, *c* = 0.2 M) was added, followed by 1,2-bis(dimethylamino)ethane (5 mg, 6 µL, 0.04mmol, 0.2 equiv.) and thiophenol (44.1 mg, 41.0 µL, 0.400 mmol, 2.00 equiv.). The vial was placed in 2 cm distance to two 34 W blue LEDs. The temperature was kept at approximately 30 °C with the use of a cooling fan. The reaction mixture was stirred for 16 hours under blue LED irradiation, then diluted with ethyl acetate (1 mL), and filtered through a short pad of silica using ethyl acetate (20 mL) as eluent. The filtrate was collected and concentrated *in vacuo* and then purified by flash column chromatography on silica gel, eluting with EtOAc/pentane (1:10 v/v) to afford **50** (33.7 mg, 70% yield) as a pale yellow solid.

R*f* = 0.40 (EtOAc/pentane, 1:9, v/v (UV))

NMR Spectroscopy:

^{1}**H NMR** (500 MHz, CD_3CN, 25 °C, δ): 7.68 (d, *J* = 2.3 Hz, 1H), 7.64 (dd, *J* = 8.8 Hz, 2.4 Hz, 1H), 7.36 – 7.30 (m, 2H), 7.29 – 7.24 (m, 3H), 7.13 (d, *J* = 8.8 Hz, 1H), 3.93 (s, 3H).

^{13}**C {^{1}H} NMR** (126 MHz, CD_3CN, 25 °C, δ): 161.8, 139.8, 138.1, 136.7, 130.3, 130.0, 127.7, 126.8, 116.1, 113.7, 102.9, 56.9.

HRMS-ESI(m/z) calc'd for $C_{14}H_{11}NOS^+$ $[M]^+$, 241.0556; found, 241.0557. Deviation: – 0.4 ppm.

Phenylthio-etofenprox (51)

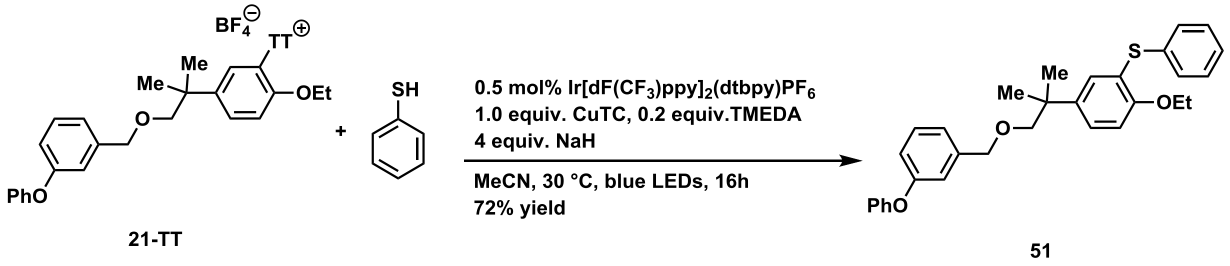

To a 4-mL borosilicate vial, equipped with a magnetic stir bar was added $Ir[dF(CF_3)ppy]_2(dtbpy)PF_6$ (1.1 mg, 1.0 µmol, 0.50 mol%), copper(I) thiophene-2-carboxylate (38 mg, 0.20 mmol, 1.0 equiv.), NaH (*c* = 60 % dispersion in mineral oil; 36 mg, 0.80 mmol,4.0 equiv.), and entofenprox thianthrenium salt **21-TT** (135 mg, 0.200 mmol, 1.00 equiv.) at 25 °C. The vial was evacuated and then filled with argon; this procedure was repeated three times. MeCN (1 mL, *c* = 0.2 M) was added, followed by 1,2-bis(dimethylamino)ethane (5 mg, 6 µL, 0.04mmol, 0.2 equiv.) and thiophenol (44.1 mg, 41.0 µL, 0.400 mmol, 2.00 equiv.). The vial was placed in 2 cm distance to two 34 W blue LEDs. The temperature was kept at approximately 30 °C with the use of a cooling fan. The reaction mixture was stirred for 16 hours under blue LED irradiation, then diluted with ethyl acetate (1 mL), and filtered through a short pad of silica using ethyl acetate (20 mL) as eluent. The filtrate was collected and concentrated *in vacuo* and then purified by flash column chromatography on silica gel, eluting with EtOAc/pentane (1:20 v/v) to afford **51** (69.6 mg, 72% yield) as a colorless solid.

$\mathbf{R}_f$ = 0.70 (EtOAc/pentane, 1:10 v/v (UV))

NMR Spectroscopy:

^{1}**H NMR** (500 MHz, CD_3CN, 25 °C, δ): 7.41 – 7.32 (m, 2H), 7.32 – 7.20 (m, 7H), 7.18 (d, *J* = 2.5 Hz, 1H), 7.13 (tt, *J* = 7.4 Hz, 1.1 Hz, 1H), 6.98 (dd, *J* = 8.7 Hz, 1.1 Hz, 2H), 6.97 – 6.94 (m, 1H), 6.92 – 6.85 (m, 2H), 6.84 (t, *J* = 2.0 Hz, 1H), 4.34 (s, 2H), 4.01 (q, *J* = 7.0 Hz, 2H), 3.36 (s, 2H), 1.23 (t, *J* = 7.0 Hz, 3H), 1.17 (s, 6H).

^{13}**C {^{1}H} NMR** (126 MHz, CD_3CN, 25 °C , δ): 157.9, 157.7, 155.9, 141.8, 141.0, 136.0, 135.2, 131.0, 130.9, 130.5, 130.3, 129.7, 127.3, 127.3, 124.0, 122.8, 122.7, 119.4, 118.1, 112.6, 80.3, 72.7, 64.8, 38.9, 26.0, 14.6.

HRMS-ESI(m/z) calc'd for $C_{31}H_{32}O_3SNa^+$ $[M+Na]^+$, 507.1964; found, 507.1965. Deviation: – 0.1 ppm.

Gram-scale synthesis of *tert*-butyl 3-(4-(3-chloropropyl)phenoxy)azetidine-1-carboxylate (46)

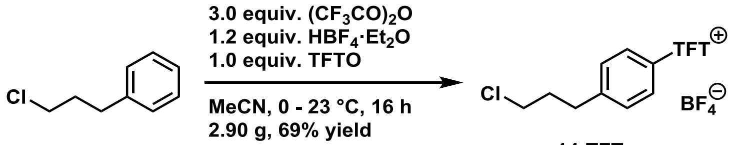

14-TFT

Under an ambient atmosphere, a 50-mL glass flask was charged with 3-chloropropyl benzene (1.23 g, 8.00 mmol, 1.00 equiv) and MeCN (20 mL, *c* = 0.40 M). Trifluoroacetic anhydride (3.33 mL, 5.04 g, 24.0 mmol, 3.00 equiv.) was added while stirring the reaction mixture at 25 °C. After cooling to 0 °C, tetrafluorothianthren-*S*-oxide (2.43 g, 8.00 mmol, 1.00 equiv.) was added in one portion, followed by the dropwise addition of $HBF_4{\cdot}OEt_2$ (1.31 mL, 1.55 g, 9.60 mmol, 1.20 equiv.). The mixture was stirred at 0 °C for 2 hours, then warmed to 25 °C for 14 hours. The solution was diluted with CH_2Cl_2 (10 mL) and poured onto a mixture of CH_2Cl_2 (70 mL) and saturated aqueous $NaHCO_3$ solution (100 mL). After stirring for 5 min, the mixture was poured into a separating funnel, and the aqueous phase was extracted with DCM (2 × 30 mL). The DCM layer was washed with aqueous $NaBF_4$ solution (10% w/w, 2 × ca. 100 mL). The DCM layer was dried over Na_2SO_4, filtered, and the solvent was removed under reduced pressure. The residue was purified by chromatography on silica gel eluting with DCM/MeOH (20:1 (v/v)), then the solvent was removed *in vacuo* to afford **14-TFT** (2.90 g, 69% yield) as a colorless powder.

R*f* = 0.30 (MeOH/DCM, 1/15, v/v).

NMR Spectroscopy:

^{1}H NMR (500 MHz, CD_3CN, 25 °C, δ): 8.41 (dd, *J* = 9.1, 7.1 Hz, 2H), 7.95 (dd, *J* = 10.0, 7.0 Hz, 2H), 7.38 (d, *J* = 8.5 Hz, 2H), 7.16 (d, *J* = 8.7 Hz, 2H), 3.53 (t, *J* = 6.5 Hz, 2H), 2.92 – 2.70 (m, 2H), 2.07 – 1.98 (m, 2H).

^{13}C {^{1}H} NMR (126 MHz, CD_3CN, 25 °C, δ) 154.4 (dd, *J* = 255.6, 13.7 Hz), 151.2 (dd, *J* = 255.8, 13.7 Hz), 148.5, 134.8 (dd, *J* = 8.8, 3.8 Hz), 131.3, 129.0, 125.1 (d, *J* = 24.2 Hz), 120.7 (d, *J* = 21.8 Hz), 120.3, 115.1 (dd, *J* = 7.7, 3.8 Hz), 44.8, 33.8, 32.6.

^{19}F NMR (471 MHz, CD_3CN, 25 °C, δ): –125.2 (m), –133.8 (m), –151.2 (brs), –151.3 (brs).

HRMS-ESI(m/z) calc'd for $C_{17}H_{24}NO_3ClNa^+$ $[M+Na]^+$, 348.1337; found, 348.1334. Deviation: 0.9 ppm.

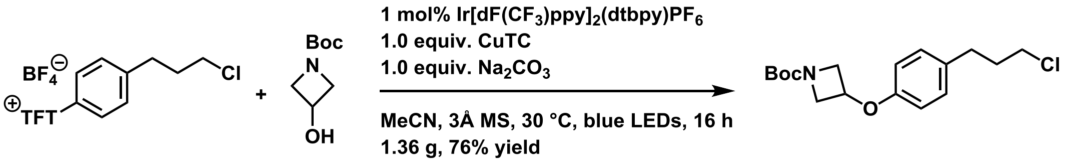

14-TFT **46**

In an anhydrous, N_2-filled glovebox, a 50-mL borosilicate flask equipped with a magnetic stir bar was charged with copper(I) thiophene-2-carboxylate (836 mg, 4.39 mmol, 0.800 equiv.), N-Boc-3-hydroxyazetidin (1.67 g, 9.65 mmol, 1.76 equiv.), Na_2CO_3 (465 mg, 4.39 mmol, 0.800 equiv.), and 4 Å molecular sieves (1 g).

Dry MeCN (22 mL, c = 0.25 M) was then added into the flask. The vial was sealed with a rubber septum. The reaction mixture was stirred at 23 ^{o}C for 2 hours: After 2 hours, 3-chloropropyl benzene derived-tetrafluorothianthrenium salt **14-TFT** (2.90 g, 5.48 mmol, 1.00 equiv.), and Ir[dF(CF_3)ppy]$_2$(dtbpy)PF_6 (49 mg, 44 µmol, 0.80 mol%) were added into the reaction mixture. The vial was sealed with the same rubber septum and transferred out of the glovebox. The flask was placed in 2 cm distance to three 34 W blue LEDs (see Figure S4 left). The temperature was kept at approximately 30 °C with the use of a cooling fan. The reaction mixture was stirred for 16 hours under blue LED irradiation, and then diluted with CH_2Cl_2 (40 mL). The reaction mixture was filtered through a short pad of silica using CH_2Cl_2 (100 mL) as the eluent. The filtrate was collected and concentrated *in vacuo,* and the residue was then purified by flash column chromatography on silica gel, eluting with EtOAc/pentane (1:8 v/v) to afford **46** (1.36 g, 76% yield) as a yellow oil, which solidified upon standing to a pale yellow solid. (see Figure S4 right)

R_f = 0.30 (EtOAc/pentane, 1:8, v/v (UV, cerium molybdate))

NMR Spectroscopy:

^{1}H NMR (300 MHz, CD_3CN, 25 °C, δ): 7.14 (d, J = 8.8 Hz, 2H), 6.72 (d, J = 8.6 Hz, 2H), 4.89 (tt, J = 6.4 Hz, 4.0 Hz, 1H), 4.25 (ddd, J = 9.6 Hz, 6.4 Hz, 1.1 Hz, 2H), 3.83 (ddd, J = 9.5 Hz, 4.0 Hz, 1.1 Hz, 2H), 3.55 (t, J = 6.6 Hz, 2H), 2.68 (dd, J = 8.5 Hz, 6.6 Hz, 2H), 2.07 – 1.96 (m, 2H), 1.42 (s, 9H).

^{13}C {^{1}H} NMR (75 MHz, CD_3CN, 25 °C, δ): 157.1, 156.1, 135.0, 130.7, 115.6, 80.0, 66.9, 66.9, 57.29, 45.4, 35.1, 32.5, 28.5.

HRMS-ESI(m/z) calc'd for $C_{17}H_{24}NO_3ClNa^+$ [M+Na]$^+$, 348.1337; found, 348.1334. Deviation: 0.9 ppm.

Figure S4. Gram-scale synthesis of 46 : reaction set up under blue LEDs (left), reaction after 16 h (middle), and solidified product 46 (right).

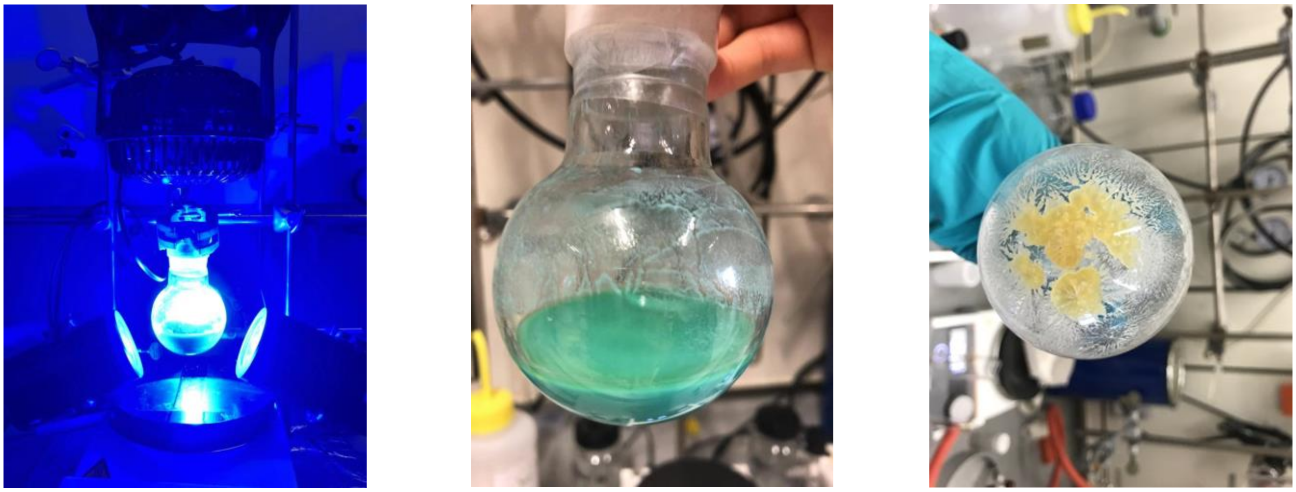

Determination of the stereochemistry of 20

Figure S5. NOESY correlations of 20

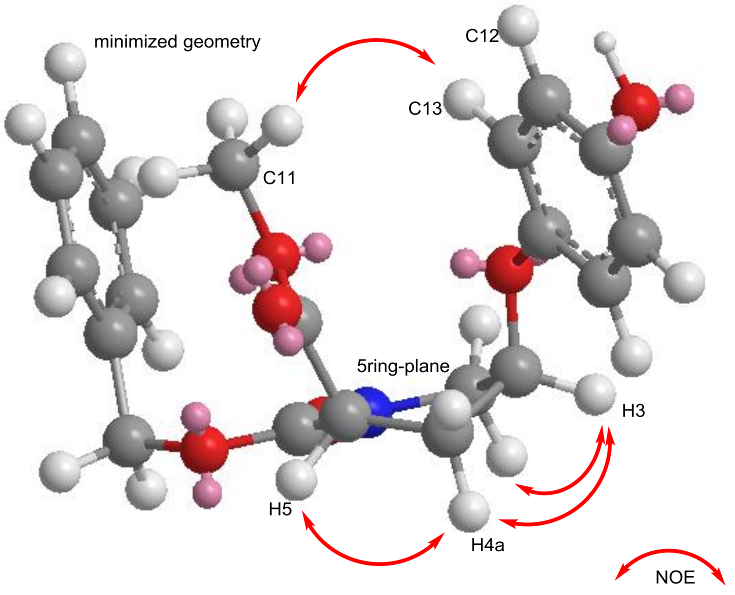

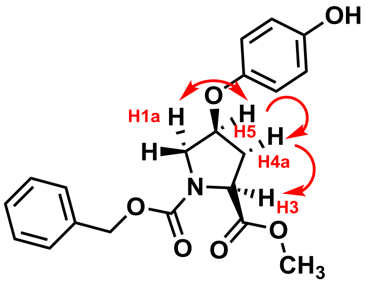

20

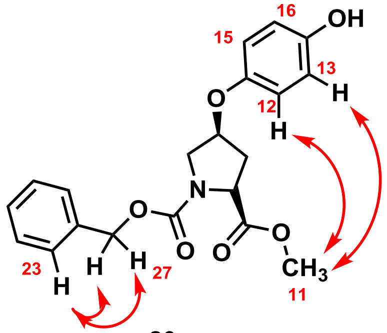

20

Due to the N-CBz group of **20**, the molecule has 2 slowly converting forms. The equilibrium is surprisingly almost exactly 50:50. This is seen from peak doubling in most ^{13}C and ^{1}H signals. In a full equilibration due to epimerization of one stereocenter, 2 sets of NMR siganls (peak doubling 50:50) would also be visible, however, with the important difference that the two forms would **NOT be interconverting**. In the NOESY spectrum, we clearly see "exchange peaks" between the two forms (e.g. between the 2 methyl signals near 3.7 ppm). This rules out the presence of 2 diastereomers.

NOESY can present the evidence to determine the stereochemistry of **20** for which diastereomer is present. The strongest evidence is between the methyl (CH_3-11) and the aromatic CH-12 and CH-13). This NOE can be explained if both substituents are on the same side of the 5-ring plane. H5 and H3 also have the same partner H4a as close neighbors. This is also the evidence that they are on the same side. The fact that H3 and H5 do not have a direct NOE between them, is probably due to the fact that they assume a more "equatorial" position.

MECHANISTIC STUDIES

UV-Vis absorption spectra of the aryl thianthrenium salts

In a typical procedure, biphenyl tetrafluorothianthrenium tetrafluoroborate salt (**22-TFT**) (52.8 mg, 0.100 mmol) was dissolved in MeCN (10 mL, *c* = 10 mM arylthianthrenium salt). Next, 60 μL of the above solution was further diluted with MeCN to a final volume of 25 mL (*c* = 24 μM arylthianthrenium salt). The absorbance of the 24 μM solution was measured using a UV-Vis spectrometer in a cuvette (l = 1.0 cm).

Figure S6. UV-Vis absorption spectra of biphenyl tetrafluorothianthrenium salt (22-TFT) in MeCN (*c* = 24 μM)

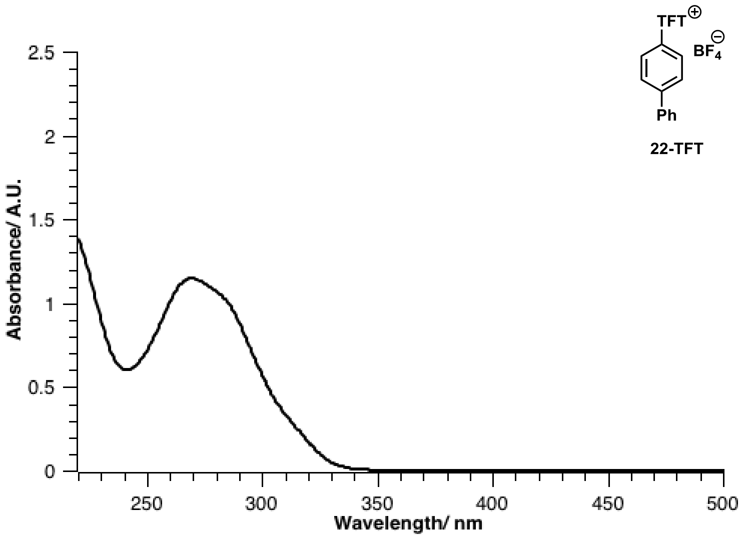

In a typical procedure, ethylbenzene tetrafluorothianthrenium tetrafluoroborate salt (**28-TFT**) (24 mg, 0.050 mmol) was dissolved in MeCN (10 mL, *c* = 5.0 mM arylthianthrenium salt). Next, 100 μL of the above solution was further diluted with MeCN to a final volume of 25 mL (*c* = 50 μM arylthianthrenium salt). The absorbance of the 50 μM solution was measured using a UV-Vis spectrometer in a cuvette (l = 1.0 cm).

Figure S7. UV-Vis absorption spectra of ethylbenzene tetrafluorothianthrenium salt (28-TFT) in MeCN (c = 50 μM)

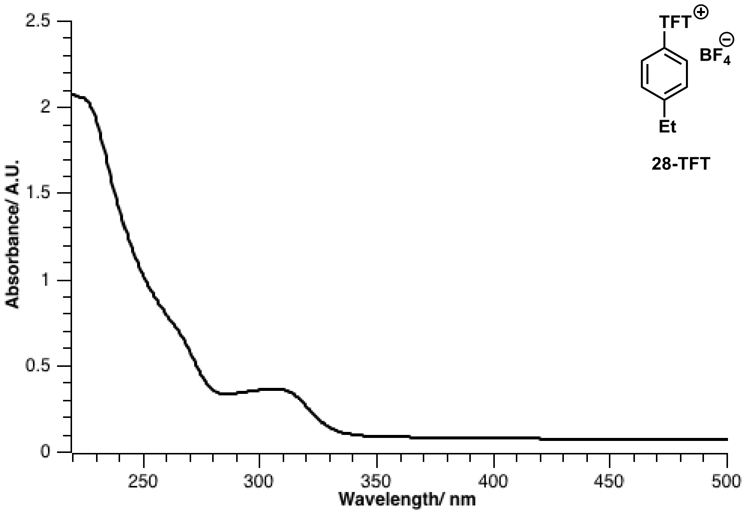

Emission spectrum of the blue LEDs

Figure S8. Emission spectrum of Kessil LEDs A160WE Tuna Blue

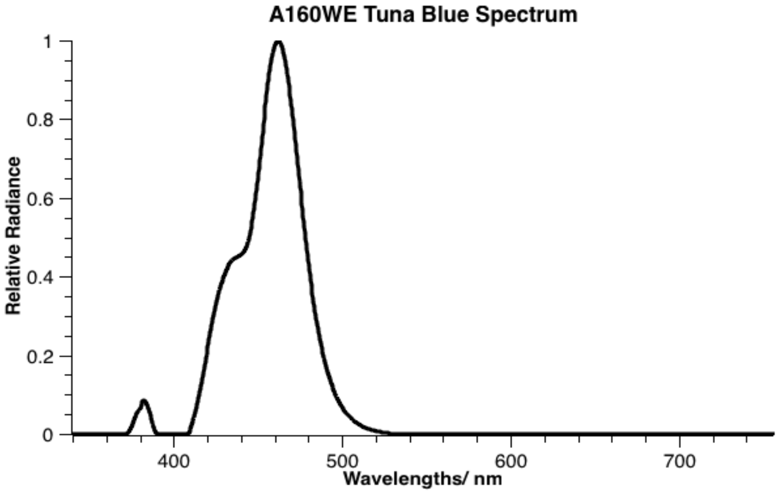

Stern-Volmer luminescence quenching studies

The visible light luminescence intensities were recorded using an Edinburgh Instruments FS5 spectrofluorometer. All luminescence measurements were recorded using a screw-top quartz cuvette (Hellma fluorescence quartz cuvette, 10 × 10 mm, 3.5 mL). All solutions of $[Ir\{dF(CF_3)ppy\}_2(dtbpy)]PF_6$, biphenyl tetrafluorothianthrenium tetrafluoroborate salt (**22-TFT**), and tetrafluorothianthrene were prepared in MeCN, in a nitrogen-filled glovebox. The solutions were transferred to a screw-top cuvette, sealed, and then brought out of the glovebox for visible light luminescence measurements.

In a typical procedure, **22-TFT** (211 mg, 0.400 mmol) was dissolved in 10 mL of stock solution (c = 18 μM $[Ir\{dF(CF_3)ppy\}_2(dtbpy)]PF_6$ in MeCN) to the concentration (c = 40 mM arylthianthrenium salt). Next, the 7 mL

of the arylthianthrenium salt solution (c = 40 mM arylthianthrenium salt) was further diluted with 3 mL of stock solution (c = 18 μM $[Ir\{dF(CF_3)ppy\}_2(dtbpy)]PF_6$ in MeCN) to the concentration (c = 28 mM arylthianthrenium salt). All subsequent solutions were prepared by diluting 7 mL of the preceding solution to the volume of 10 mL. The final solution with a concentration of 5 mM was prepared by diluting 2 mL of the 14 mM arylthianthrenium salt solution with 3 mL of stock solution (c = 18 μM $[Ir\{dF(CF_3)ppy\}_2(dtbpy)]PF_6$ in MeCN) to a final volume of 5 mL. All solutions were excited at 450 nm and the emission was measured from 455 to 600 nm.

Quenching was analyzed by plotting I_0/I according to the Stern-Volmer relationship:

$I_0/I = k_q\tau_0[Q]+1$

where I_0 represents the integral of the luminescence over the range of 455 to 600 nm in the absence of a quencher, I is the integral of luminescence over the range of 455 to 600 nm in the presence of a quencher, k_q represents the quenching rate constant, [Q] is the concentration of a given quencher, and τ_0 is the excited state lifetime of the emissive photocatalyst in the absence of quencher. The excited state lifetime of $Ir[dF(CF_3)ppy]_2(dtbpy)PF_6$ in MeCN is 2300 ns.[2]

Figure S9. Emission spectrum for $Ir(dF(CF_3)ppy)_2(dtbpy)PF_6$ luminescence quenching by biphenyl tetrafluorothianthrenium salt 22-TFT (left) and Stern-Volmer plot (right).

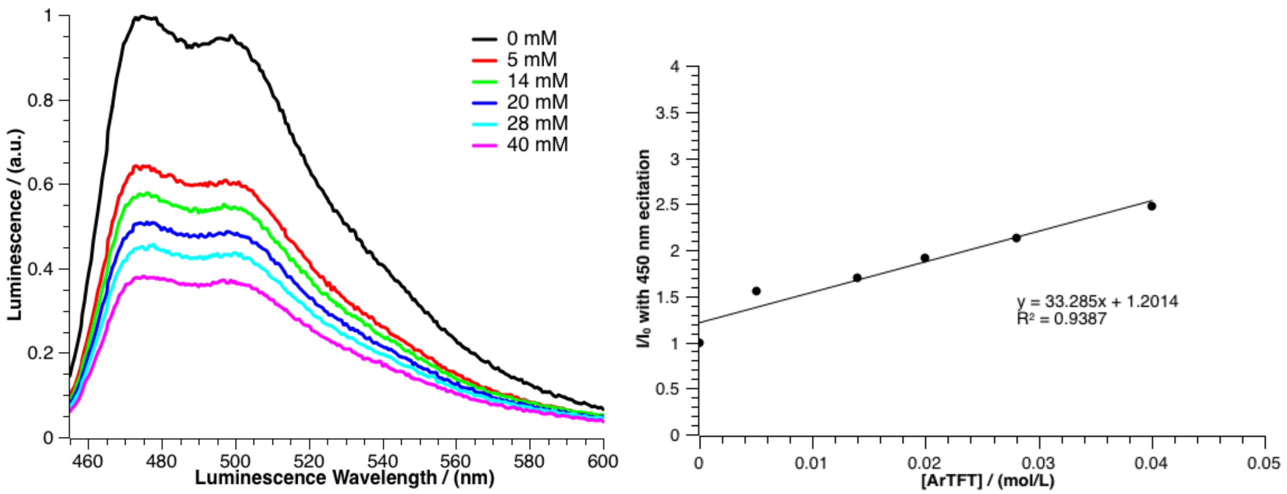

Figure S10. Emission spectrum for Ir(dF(CF$_3$)ppy)$_2$(dtbpy)PF$_6$ luminescence quenching by tetrafluorthianthrene TFT (left) and Stern-Volmer plot (right).

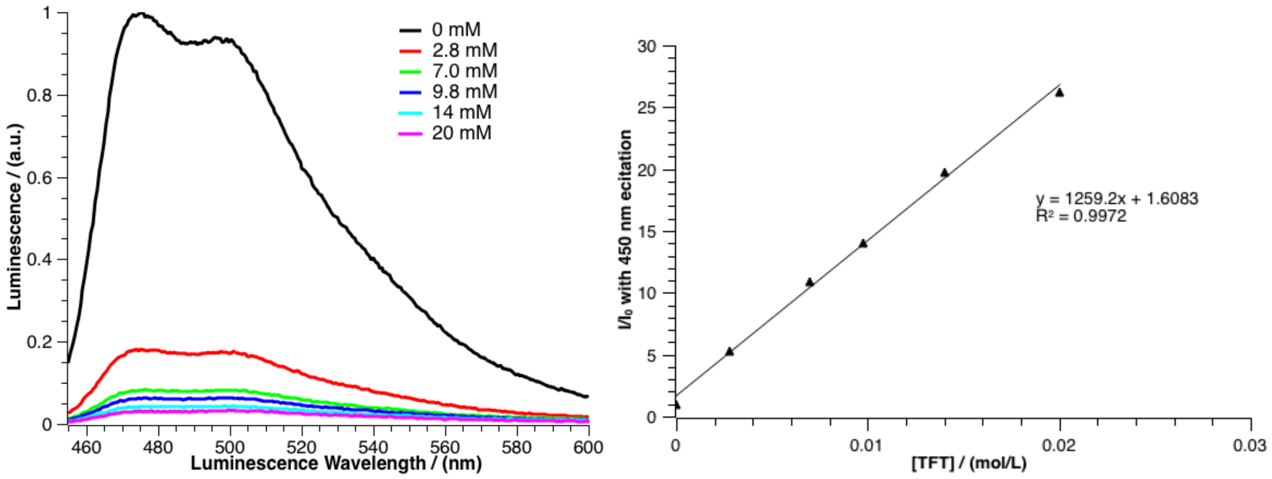

Figure S11. Emission spectra for Ir(dF(CF$_3$)ppy)$_2$(dtbpy)PF$_6$ luminescence quenching by tetrafluorthianthrene TFT and biphenyl tetrafluoro-thianthrenium salt 22-TFT (left) and quencher rate coefficient (right).

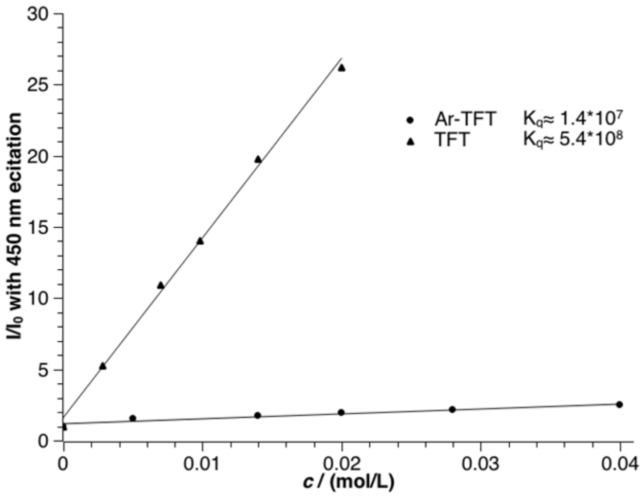

Quencher	$K_q/M^{-1}S^{-1}$
**TFT**	$5.4*10^8$
**22-TFT**	$1.4*10^7$

TEMPO trapping experiment

ArTEMPO product (52)

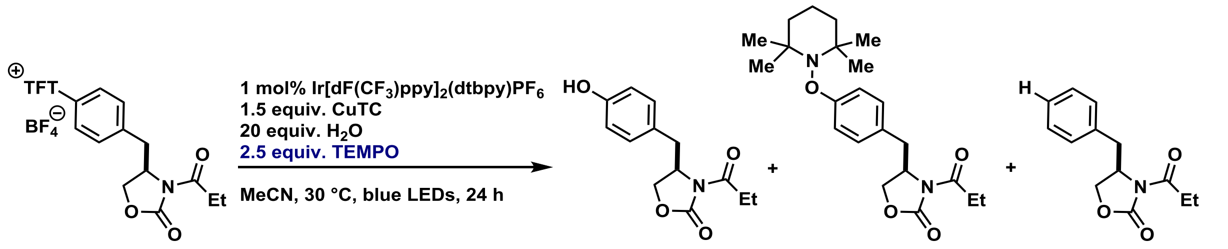

10-TFT 10, 11% yield 52, 16% yield 10-H, < 2%

To a 4-mL borosilicate vial, equipped with a magnetic stirrer bar was added copper(I) thiophene-2-carboxylate (57.2 mg, 0.300 mmol, 1.50 equiv.), (*R*)-4-(4-hydroxybenzyl)-3-propionyloxazolidin-2-one tetrafluoro-thianthrenium salt **10-TFT** (121 mg, 0.200 mmol, 1.00 equiv.), Ir[dF(CF_3)ppy]$_2$(dtbpy)PF_6 (2.2 mg, 2.0 µmol, 1.0 mol%), water (72 µL, 36 mg, 4.0 mmol, 20 equiv.), TEMPO (78.1 mg, 0.500 mmol, 2.50 equiv.), and MeCN (1 mL, *c* = 0.2 M). The vial was evacuated and then filled with argon; this procedure was repeated three times. The vial was placed in 2 cm distance to two 34 W blue LEDs. The temperature was kept at approximately 30 °C with the use of a cooling fan. The reaction mixture was stirred for 24 hours under blue LED irradiation, and then diluted with CH_2Cl_2 (2 mL). The reaction mixture was filtered through a short pad of Celite using CH_2Cl_2 (20 mL) as eluent. The filtrate was collected and concentrated *in vacuo,* and the residue was then purified by flash column chromatography on silica gel, eluting with ethyl acetate/pentane (1:10 to 1:5, v/v) to afford **52** (12.2 mg, 16% yield) as a colorless oil and **10** (5.4 mg, 11% yield) as a light yellow solid.

R*f* = 0.50 (EtOAc/pentane, 1:5, v/v (UV, cerium molybdate)).

NMR Spectroscopy:

^{1}H NMR (500 MHz, CD_3CN, 25 °C, δ): 7.27 – 7.08 (m, 2H), 7.06 – 6.94 (m, 2H), 4.60 (tt, *J* = 7.9, 3.1 Hz, 1H), 4.24 (dd, *J* = 8.9, 8.1 Hz, 1H), 4.14 (dd, *J* = 8.9, 2.8 Hz, 1H), 3.08 – 2.68 (m, 4H), 1.81 – 1.54 (m, 5H), 1.48 – 1.34 (m, 1H), 1.22 (s, 6H), 1.11 (t, *J* = 7.4 Hz, 3H), 0.96 (d, *J* = 3.1 Hz, 6H).

^{13}C {^{1}H} NMR (126 MHz, CD_3CN, 25 °C, δ): 174.7, 163.7, 154.8, 131.0, 128.0, 114.9, 67.3, 61.1, 61.1, 55.7, 40.4, 37.2, 32.9, 29.7, 20.7, 17.7, 8.7.

HRMS-ESI(m/z) calc'd for $C_{22}H_{33}N_2O_4^+$ [M+H]$^+$, 389.2435; found, 389.2432; deviation: 0.82 ppm.

SPECTROSCOPIC DATA

^{1}H NMR of flurbiprofen methyl ester tetrafluorothianthrenium salt (1-TFT)

CD_2Cl_2, 25 °C

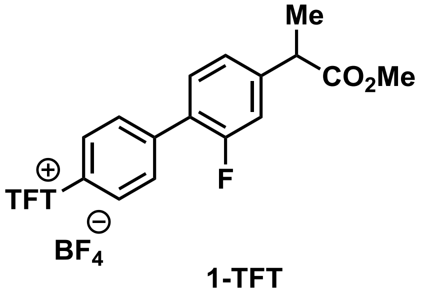

1-TFT

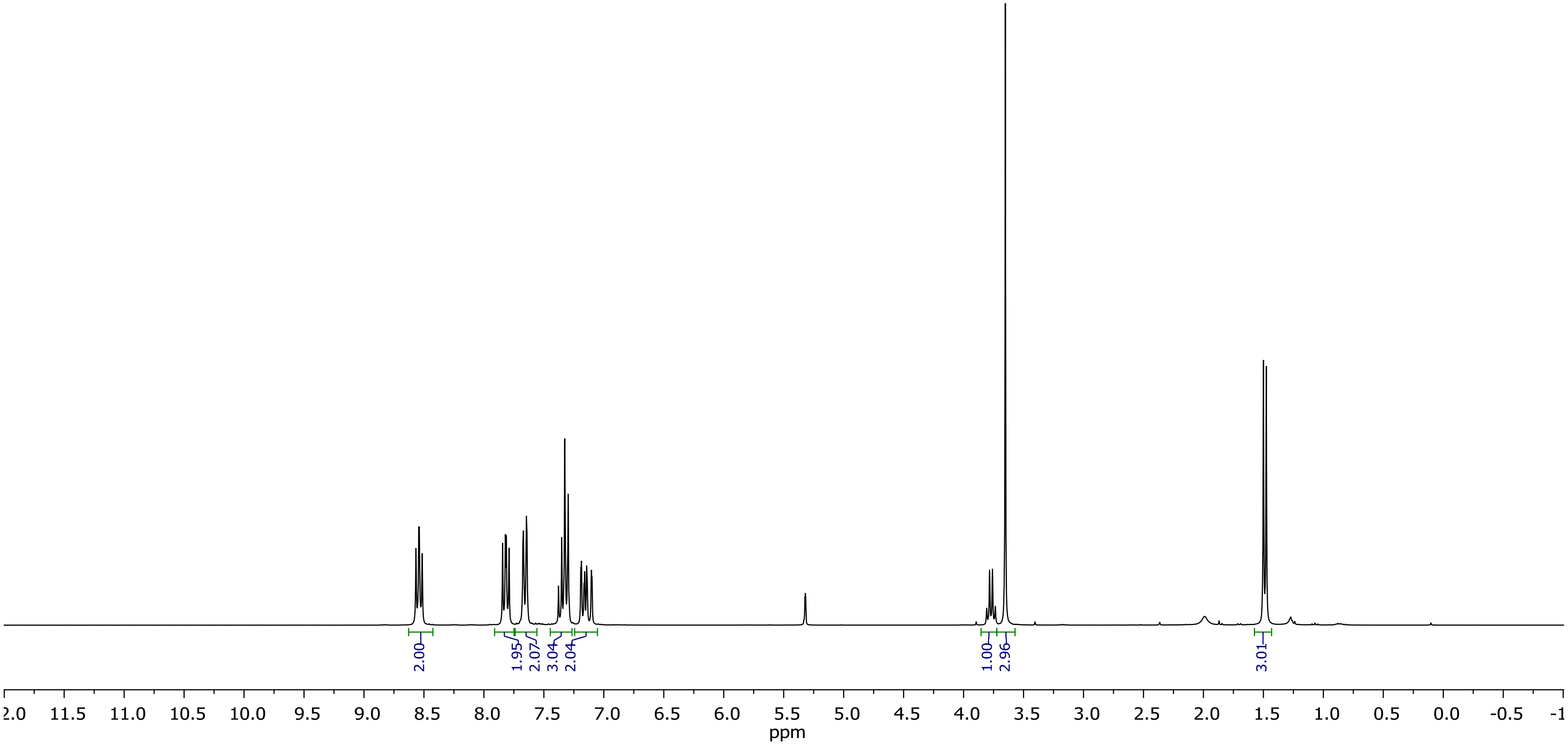

^{13}C NMR of flurbiprofen methyl ester tetrafluorothianthrenium salt (1-TFT)

CD_2Cl_2, 25 °C

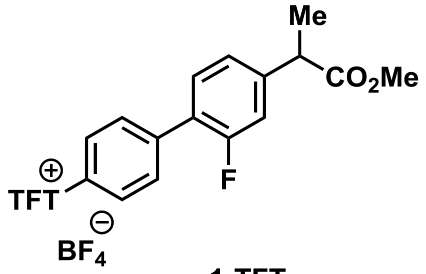

1-TFT

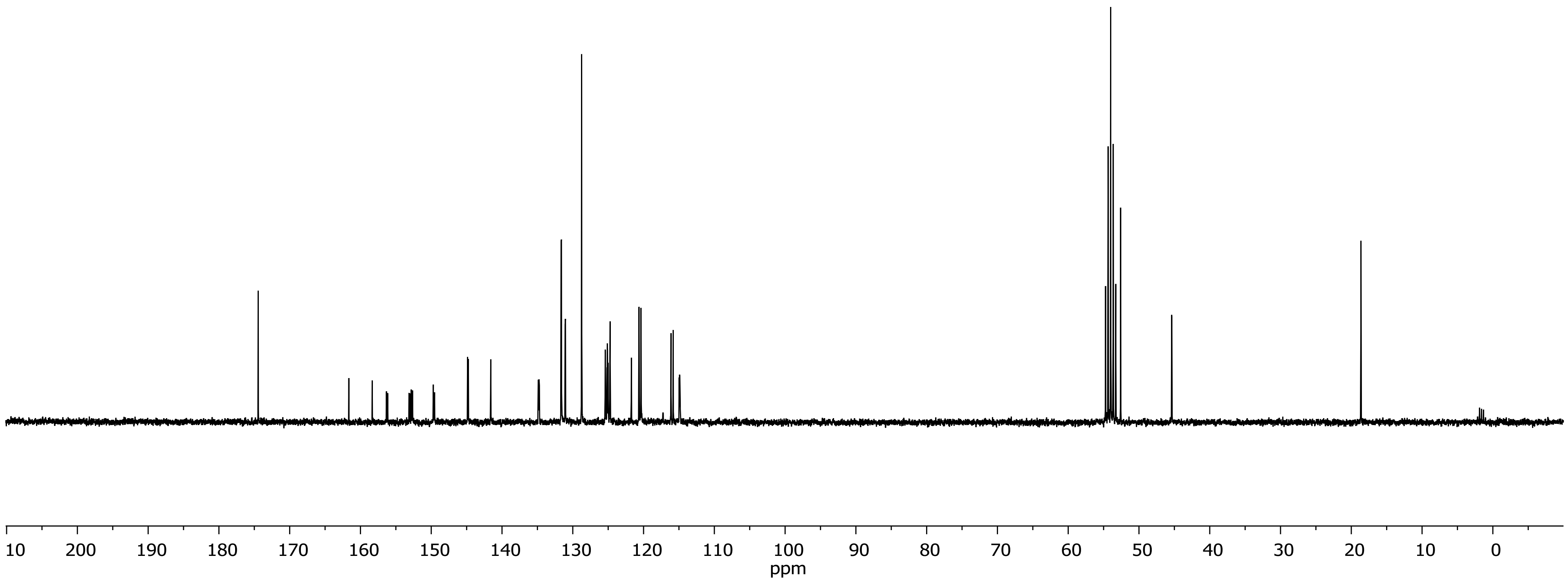

^{19}F NMR of flurbiprofen methyl ester tetrafluorothianthrenium salt (1-TFT)

CD_2Cl_2, 25 °C

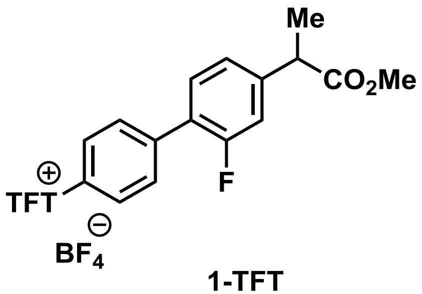

1-TFT

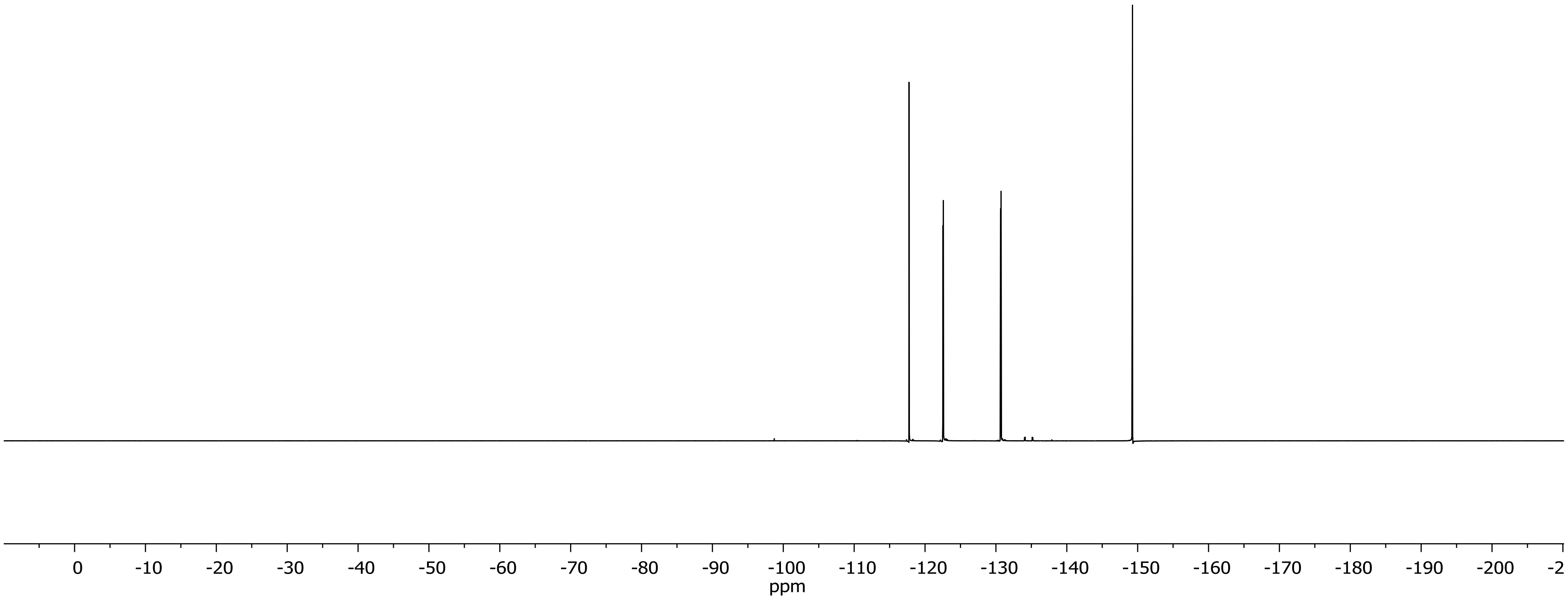

^{1}H NMR of flurbiprofen methylester-derived thianthrenium salt (1-TT)

DMSO-d$_6$, 25 °C

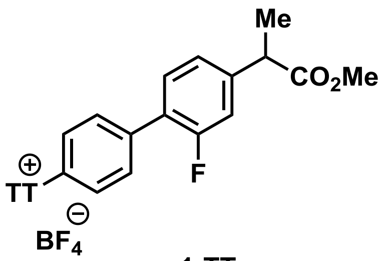

1-TT

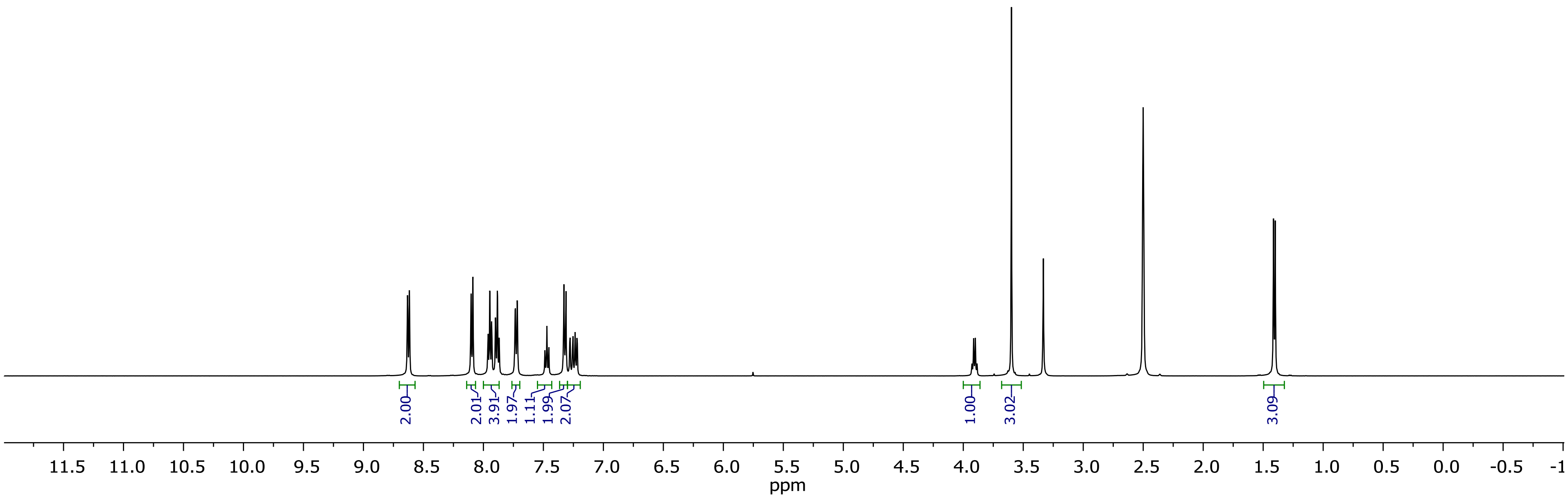

^{13}C NMR of flurbiprofen methylester thianthrenium salt (1-TT)

DMSO-d$_6$, 25 °C

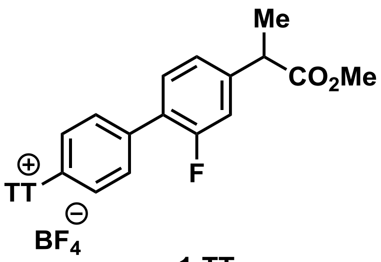

1-TT

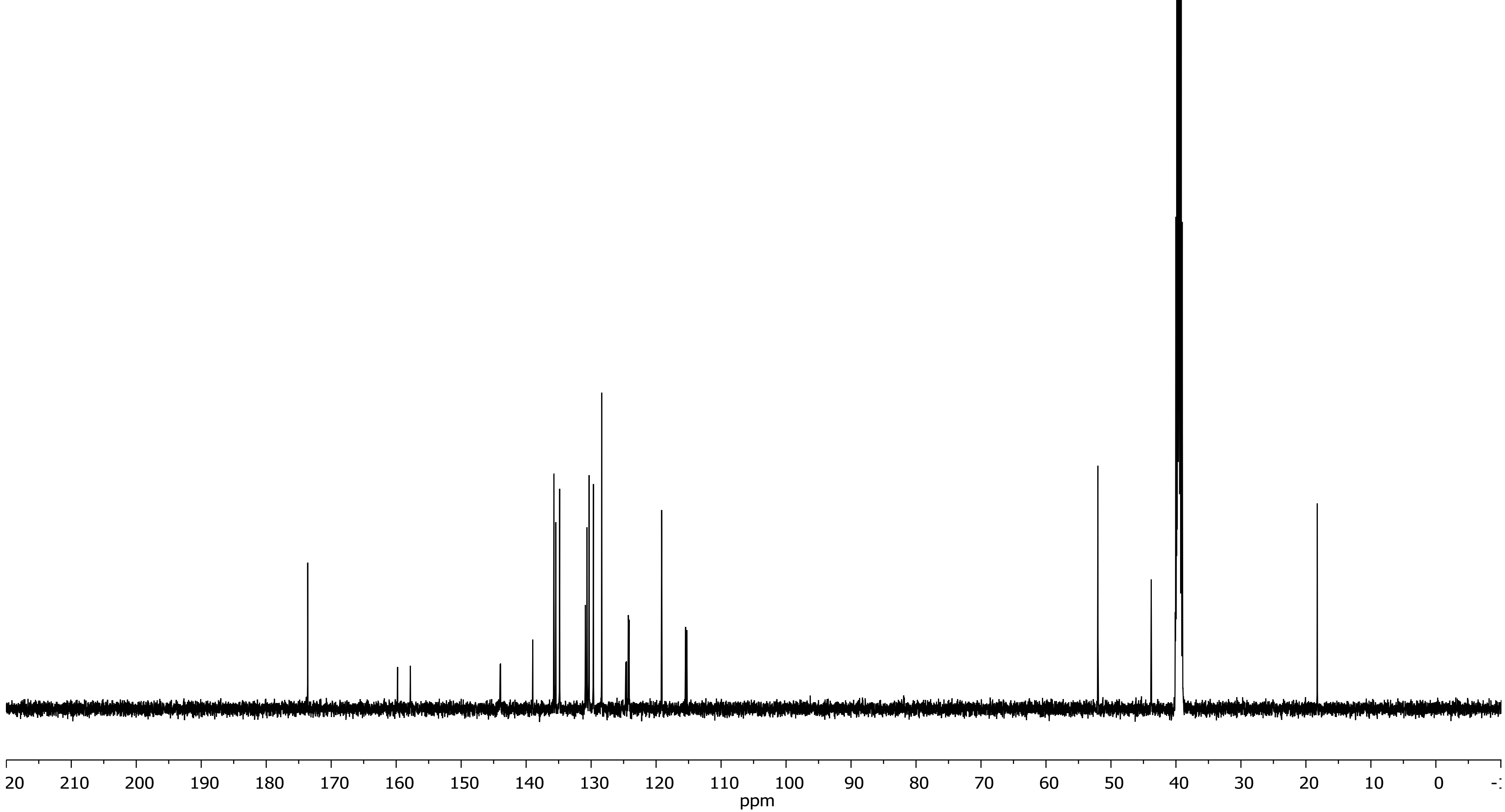

^{19}F NMR of flurbiprofen methylester thianthrenium salt (1-TT)

DMSO-d_6, 25 °C

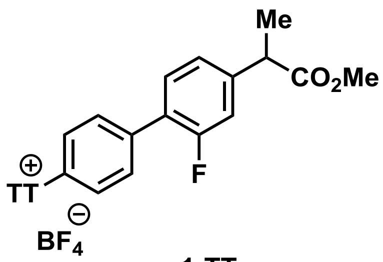

1-TT

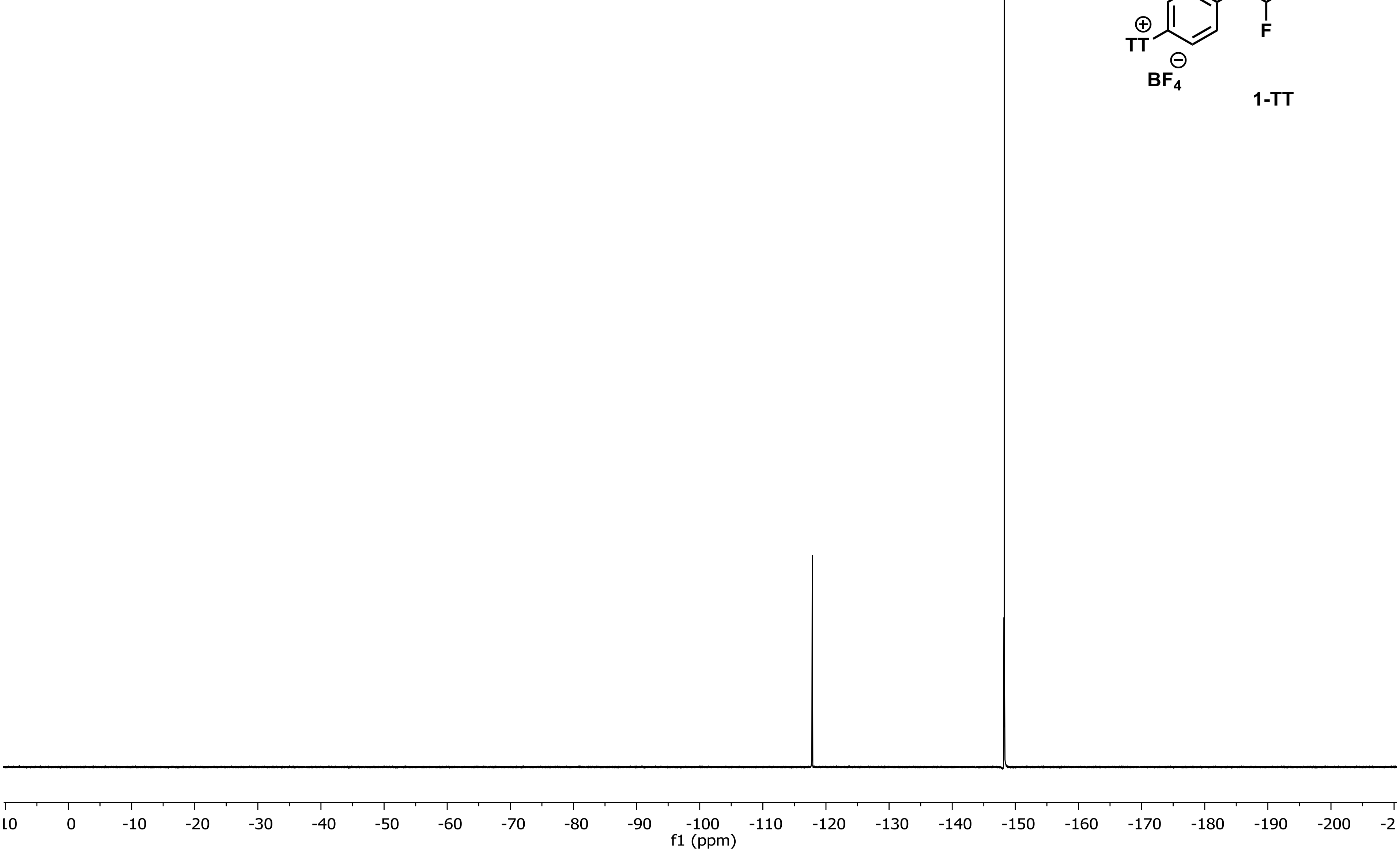

^{1}H NMR of hydroxy-flurbiprofen methyl ester (1)

CD_3CN, 25 °C

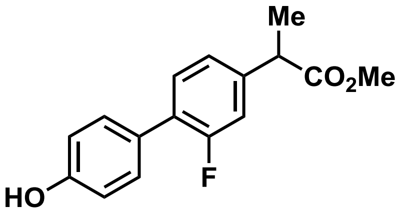

1

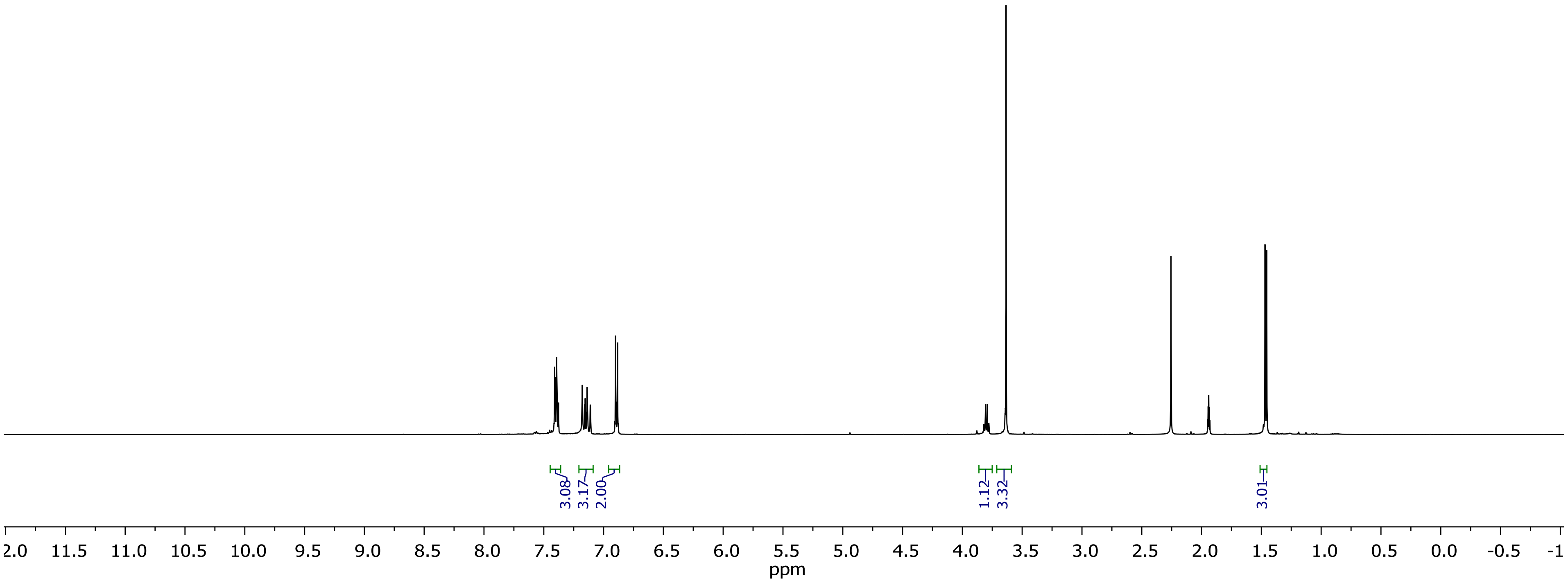

^{13}C NMR of hydroxy-flurbiprofen methyl ester (1)

CD_3CN, 25 °C

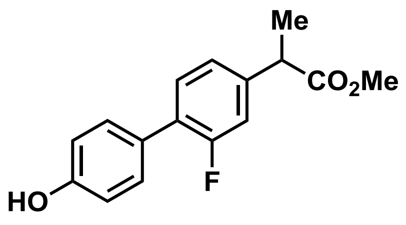

1

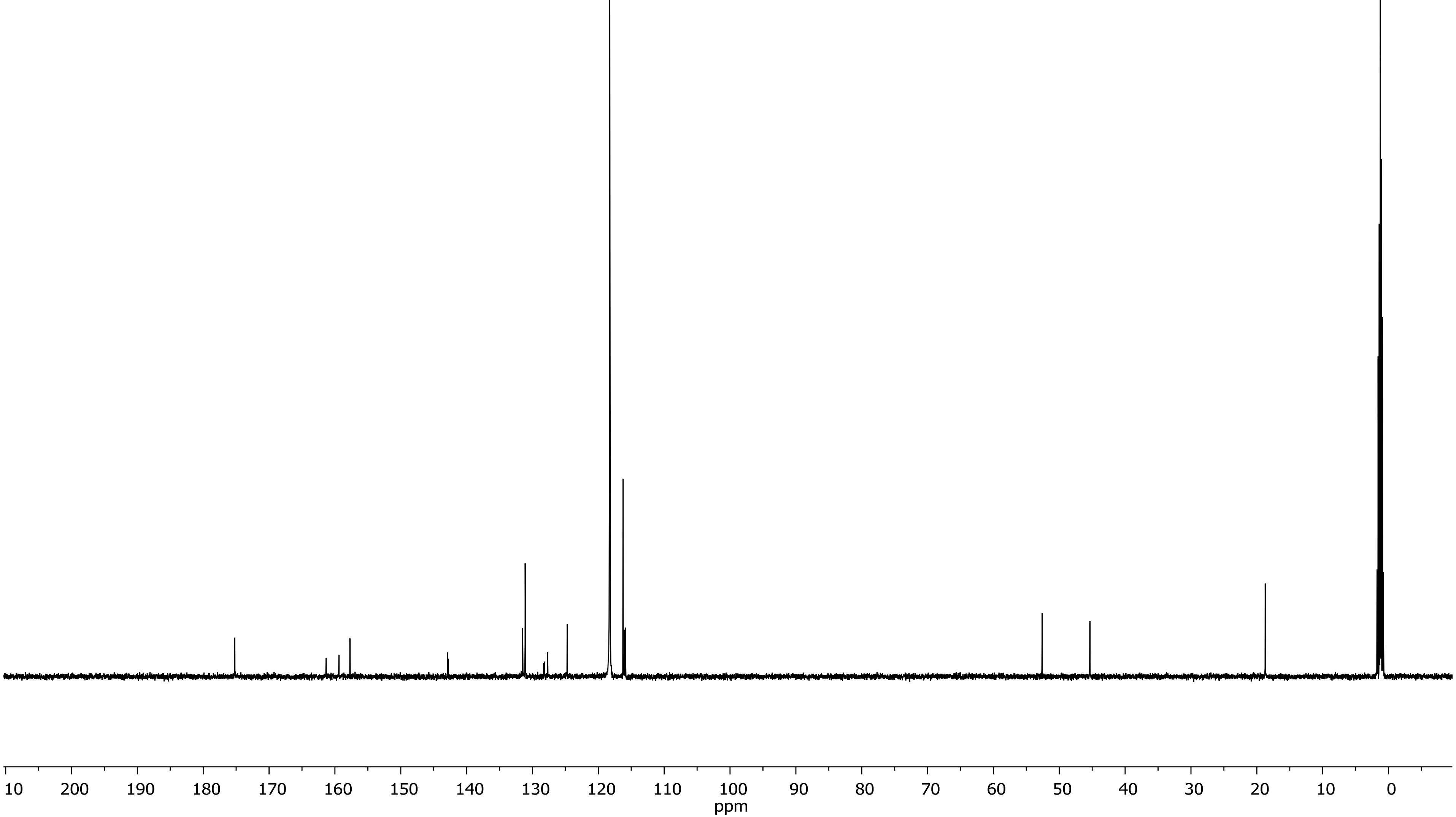

^{19}F NMR of hydroxy-flurbiprofen methyl ester (1)

CD_3CN, 25 °C

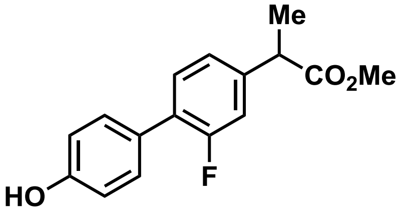

1

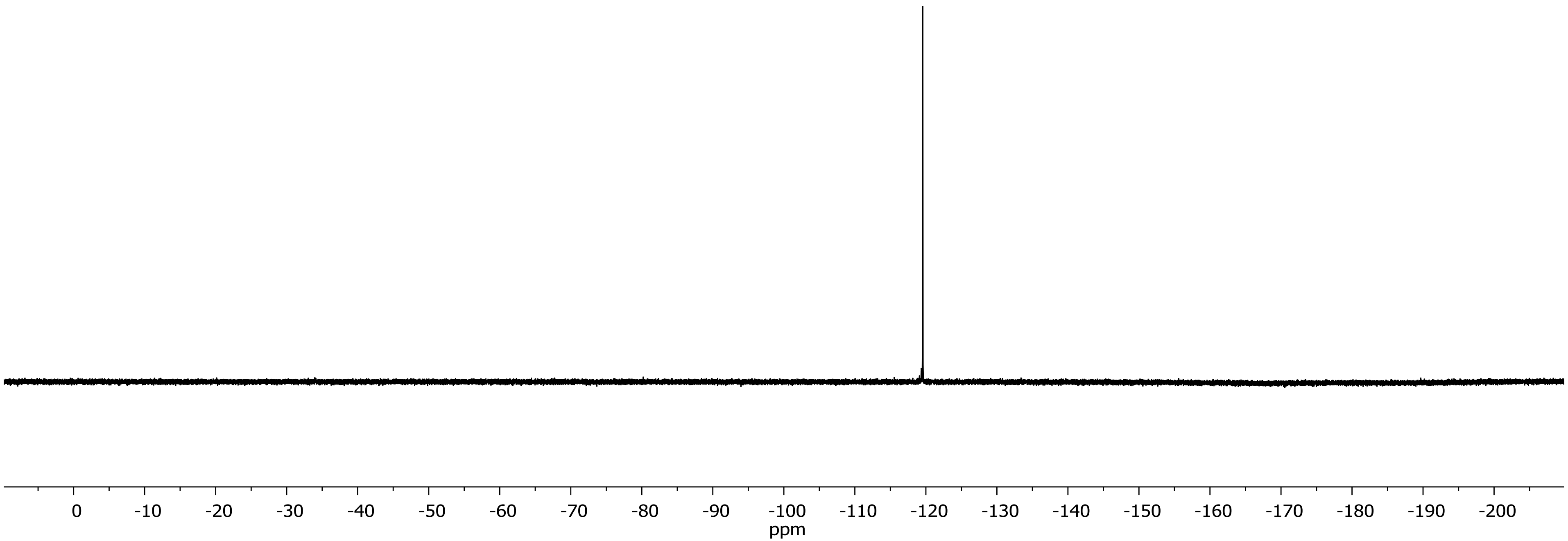

^{1}H NMR of 4-chlorophenol (2)

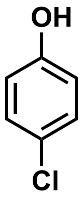

2

$CDCl_3$, 25 °C

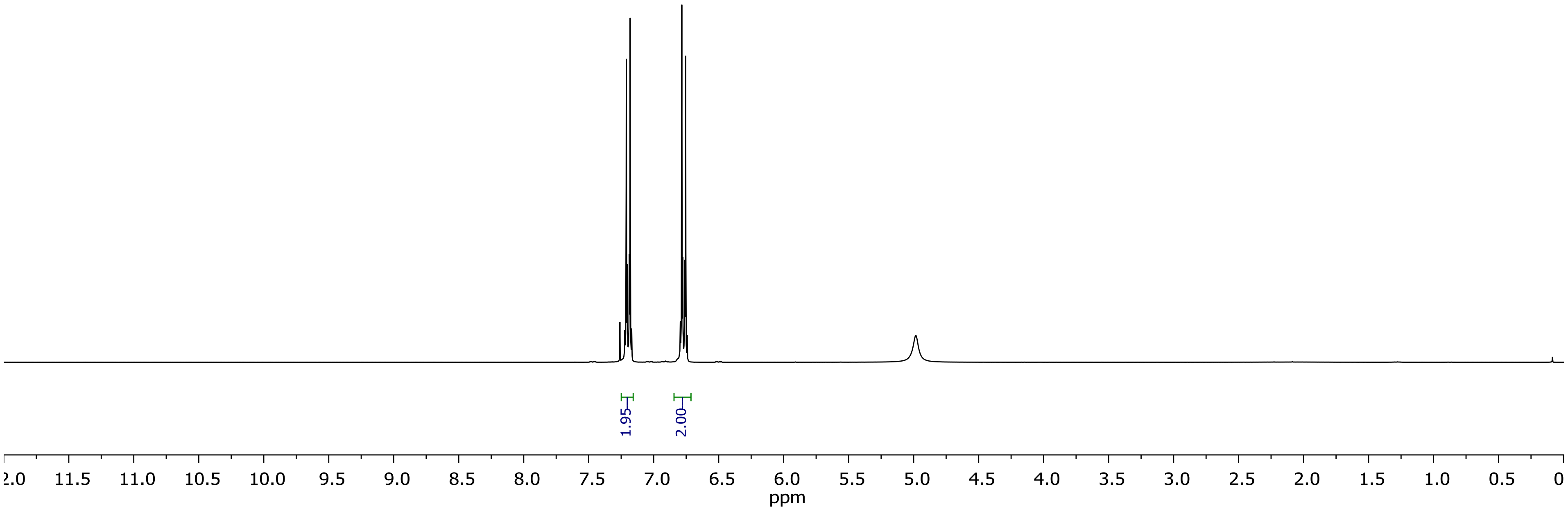

^{13}C NMR of 4-chlorophenol (2)

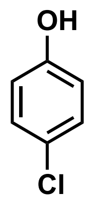

2

$CDCl_3$, 25 °C

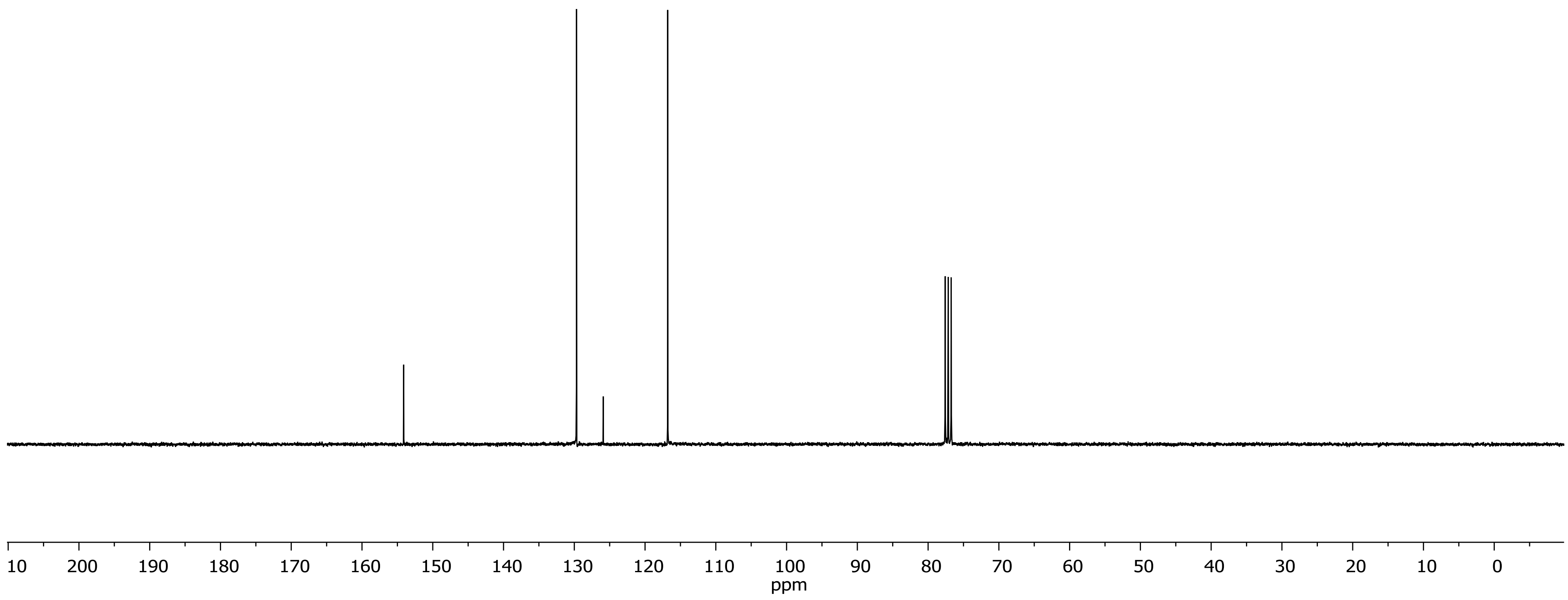

^{1}H NMR of 2-methoxybenzaldehyde tetrafluorothianthrenium salt (3-TFT)

CD_3CN, 25 °C

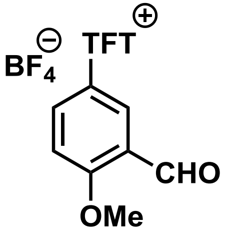

3-TFT

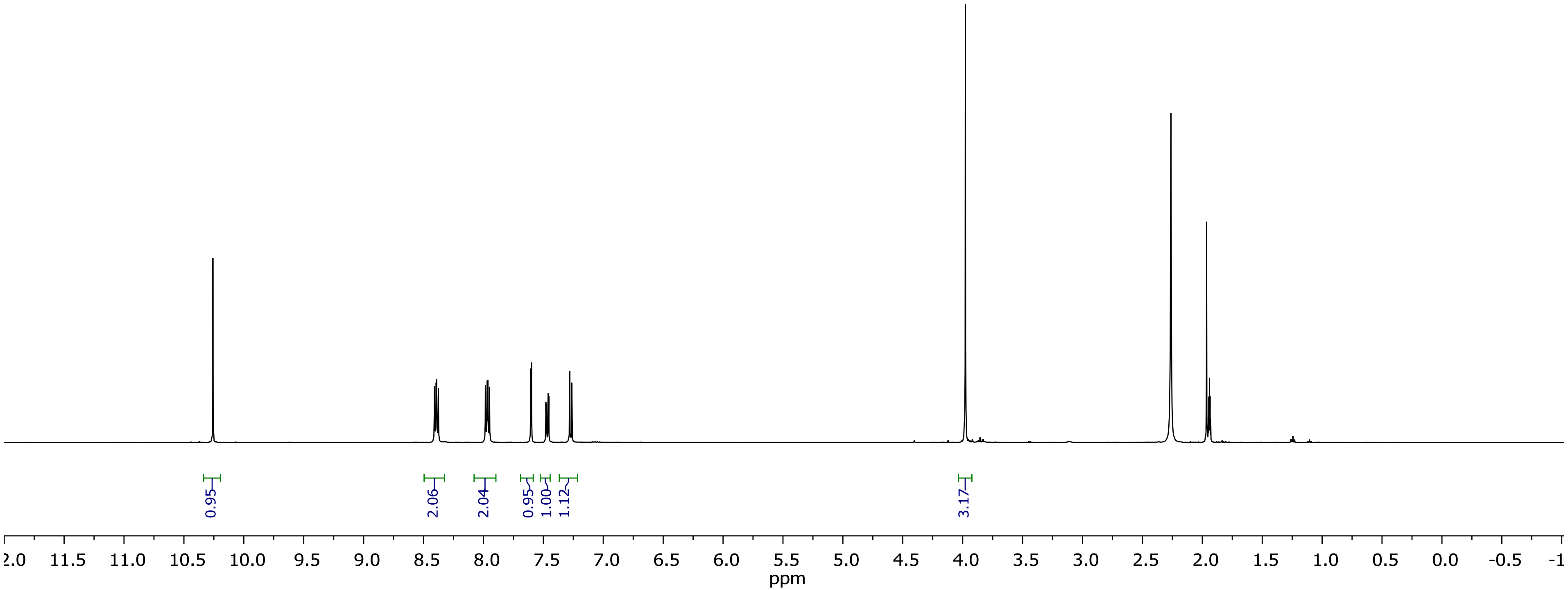

^{13}C NMR of 2-methoxybenzaldehyde tetrafluorothianthrenium salt (3-TFT)

CD_3CN, 25 °C

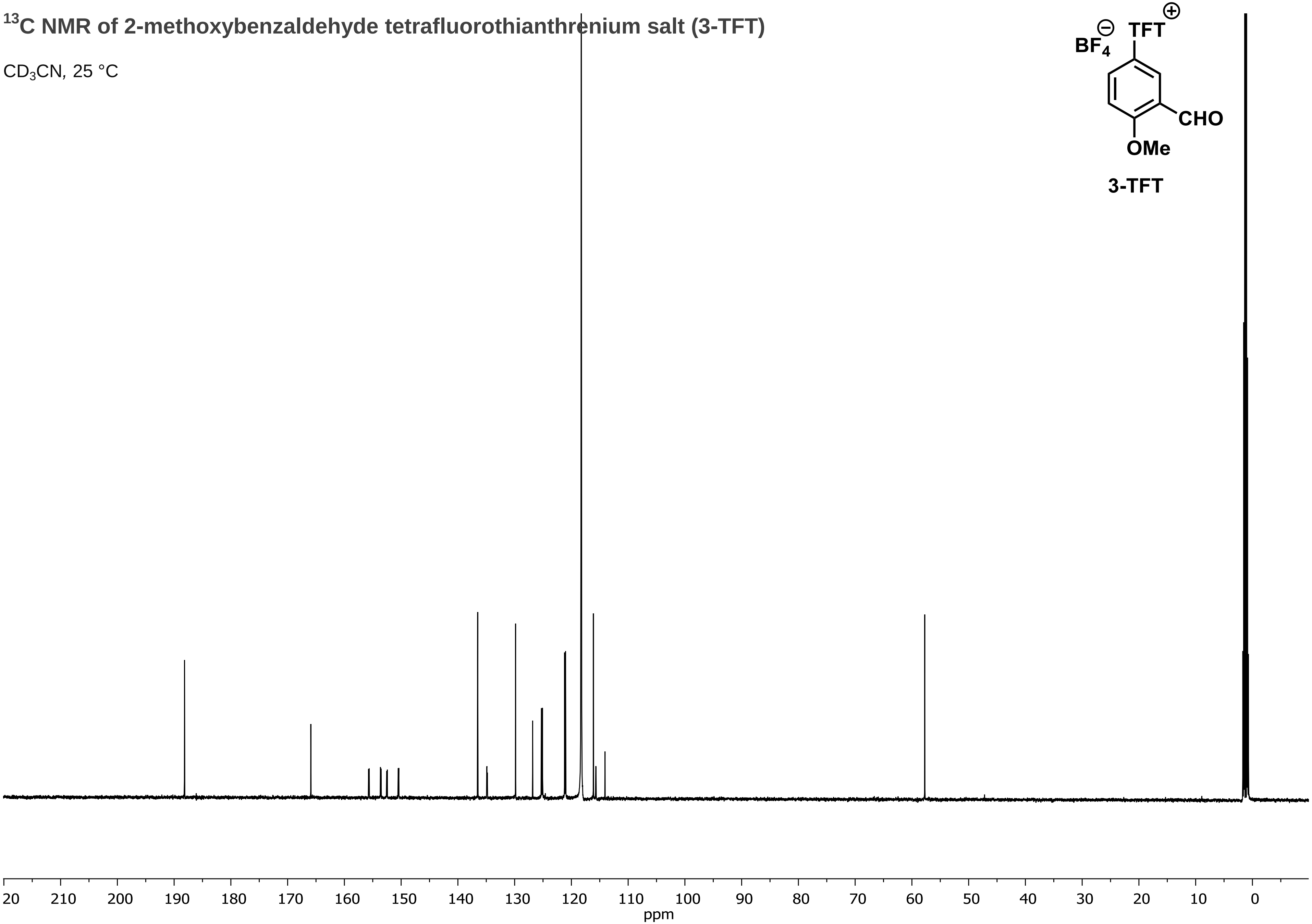

^{19}F NMR of 2-methoxybenzaldehyde tetrafluorothianthrenium salt (3-TFT)

CD_3CN, 25 °C

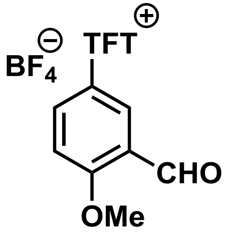

3-TFT

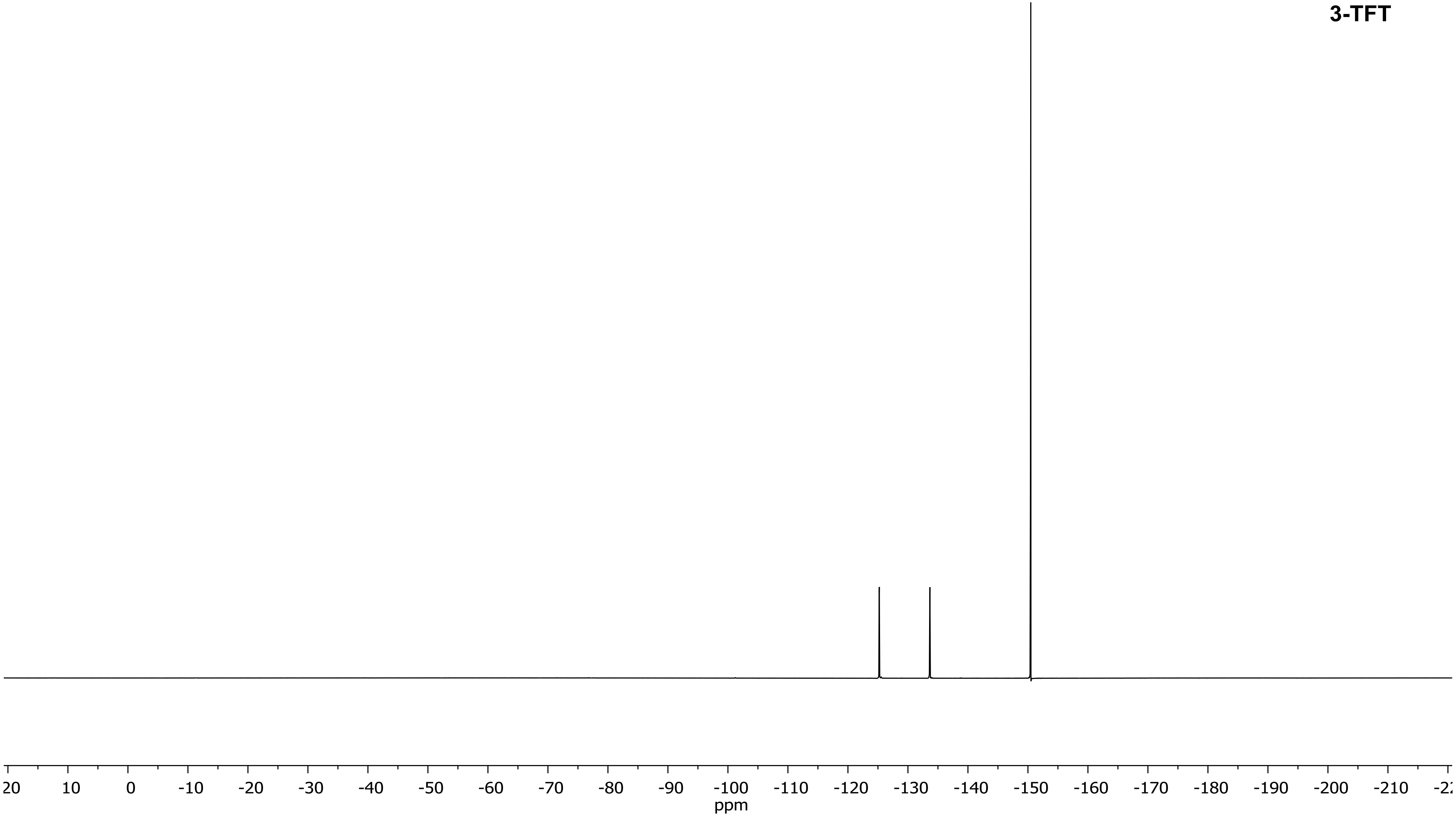

^{1}H NMR of 5-hydroxy-2-methoxybenzaldehyde (3)

$CDCl_3$, 25 °C

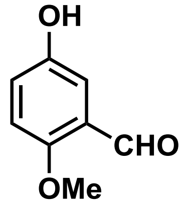

3

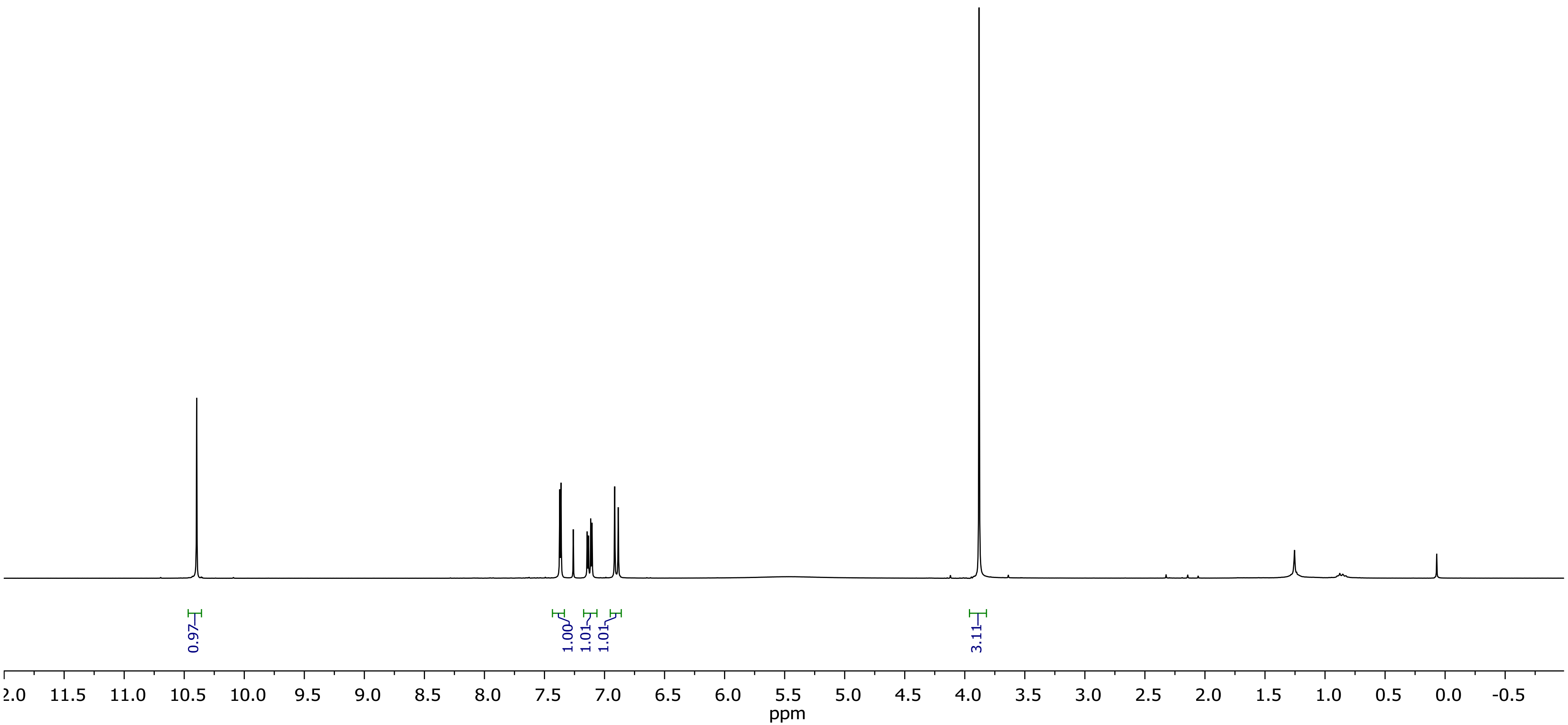

^{13}C NMR of 5-hydroxy-2-methoxybenzaldehyde (3)

$CDCl_3$, 25 °C

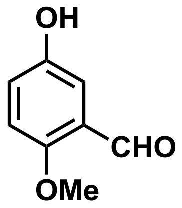

3

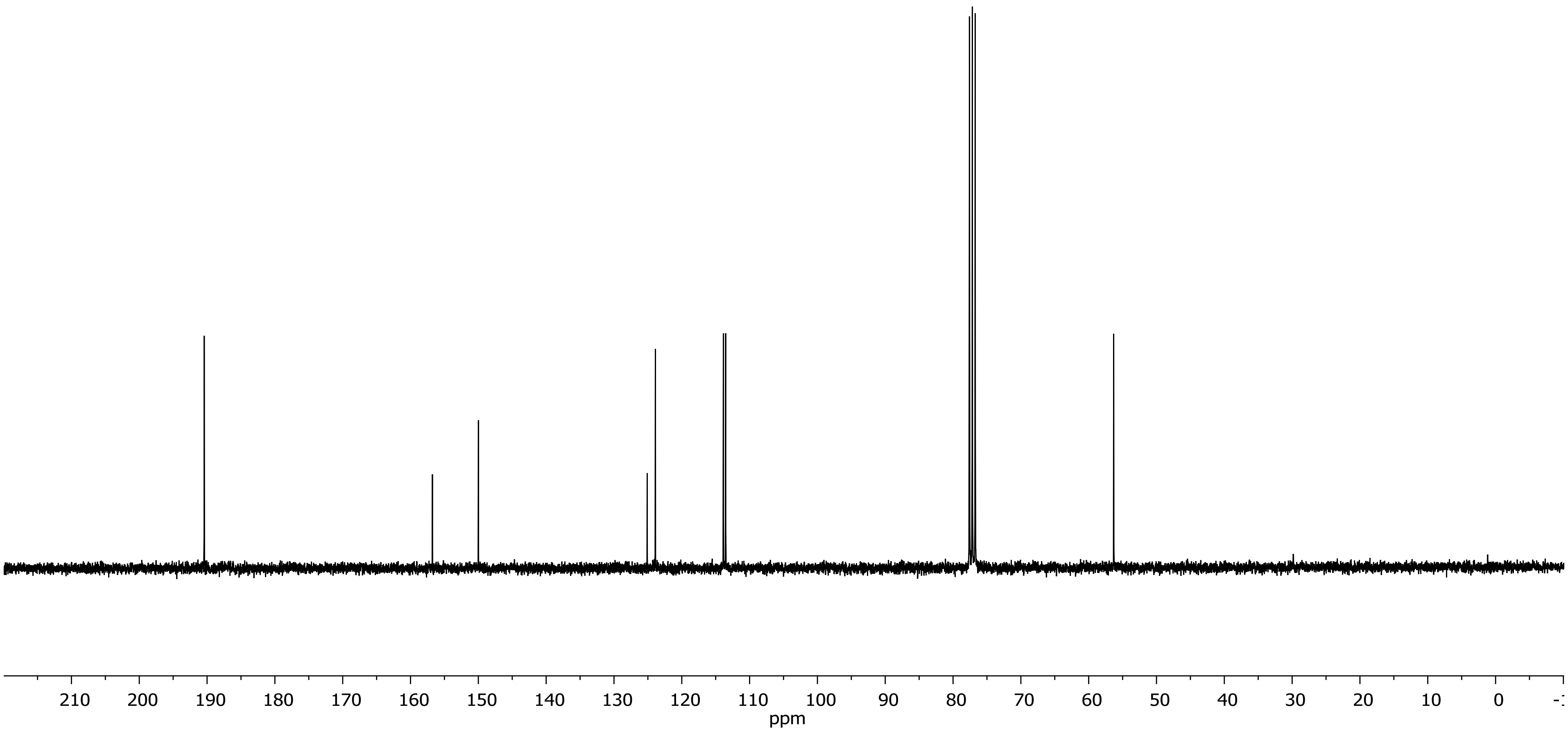

^{1}H NMR of 2,2,2-trifluoro-N-phenylacetamide tetrafluorothianthrenium salt (4-TFT)

CD_3CN, 25 °C

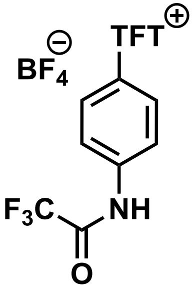

4-TFT

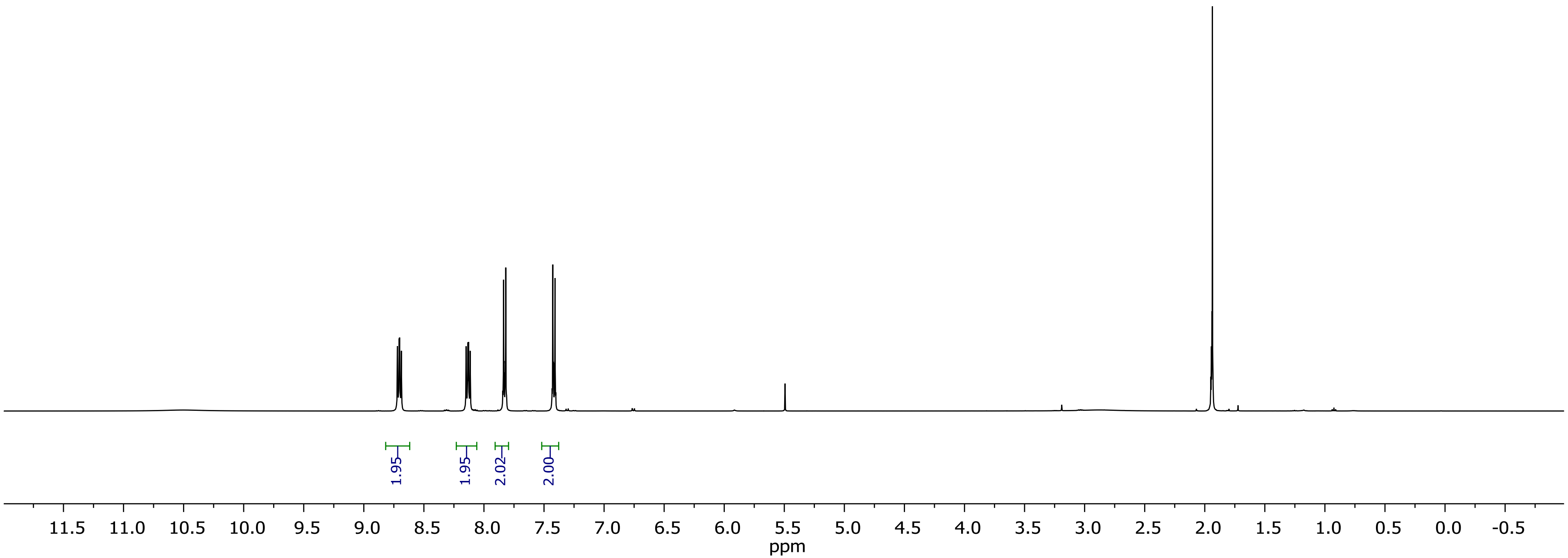

^{13}C NMR of 2,2,2-trifluoro-N-phenylacetamide tetrafluorothianthrenium salt (4-TFT)

CD_3CN, 25 °C

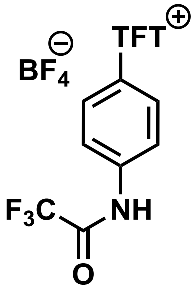

4-TFT

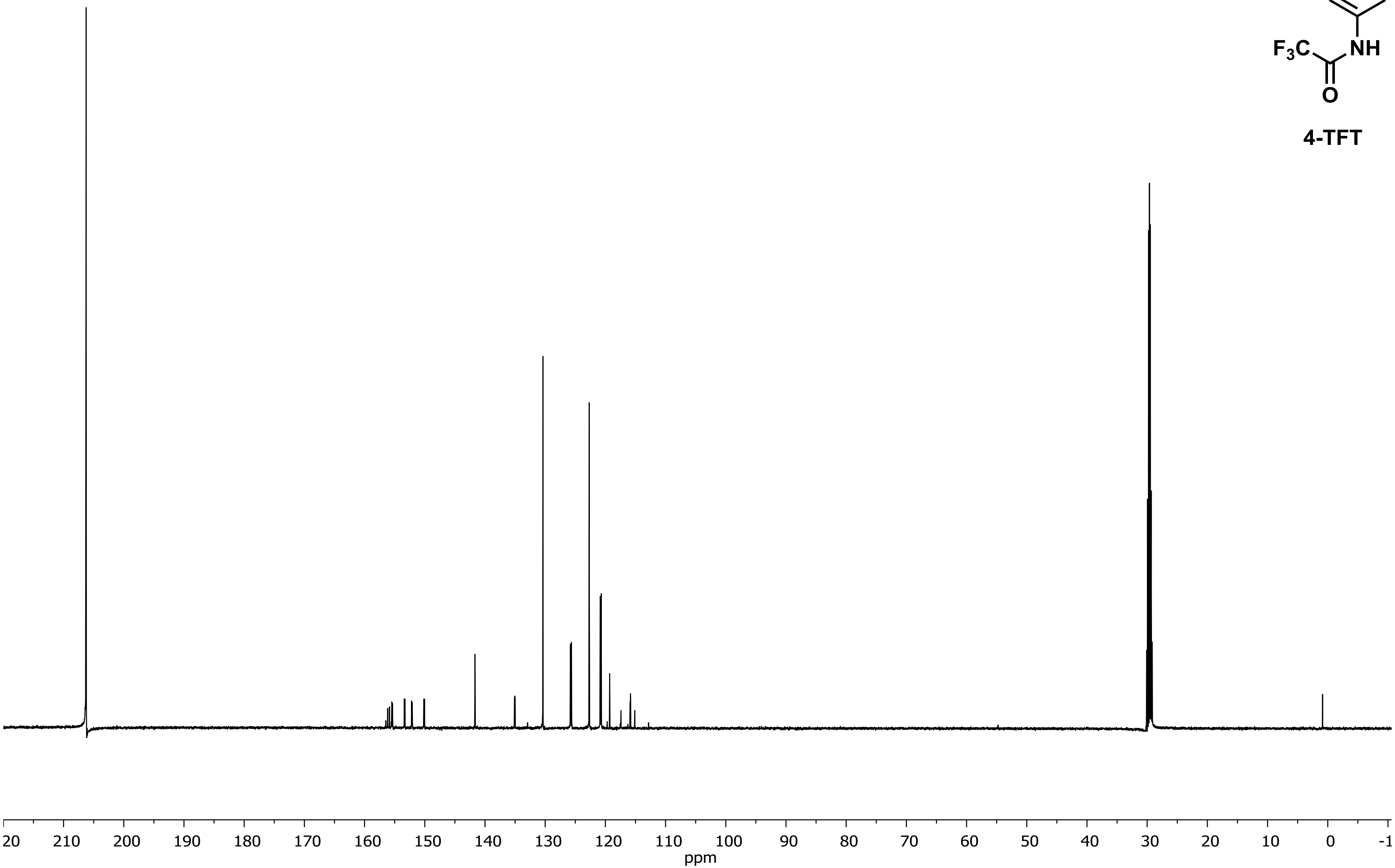

^{19}F NMR of 2,2,2-trifluoro-N-phenylacetamide tetrafluorothianthrenium salt (4-TFT)

CD_3CN, 25 °C

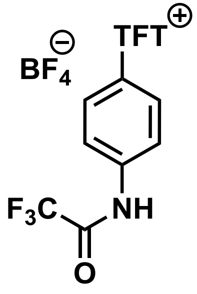

4-TFT

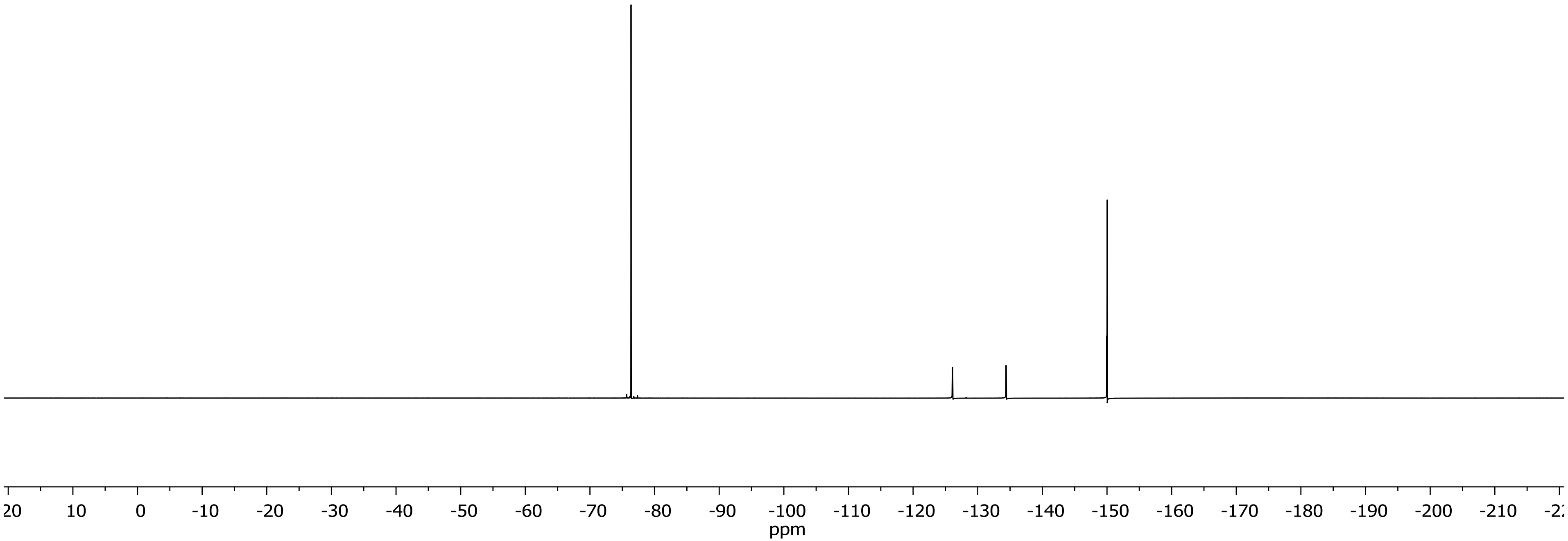

^{1}H NMR of 2,2,2-trifluoro-N-(4-hydroxyphenyl)acetamide (4)

CD_3CN, 25 °C

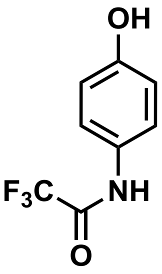

4

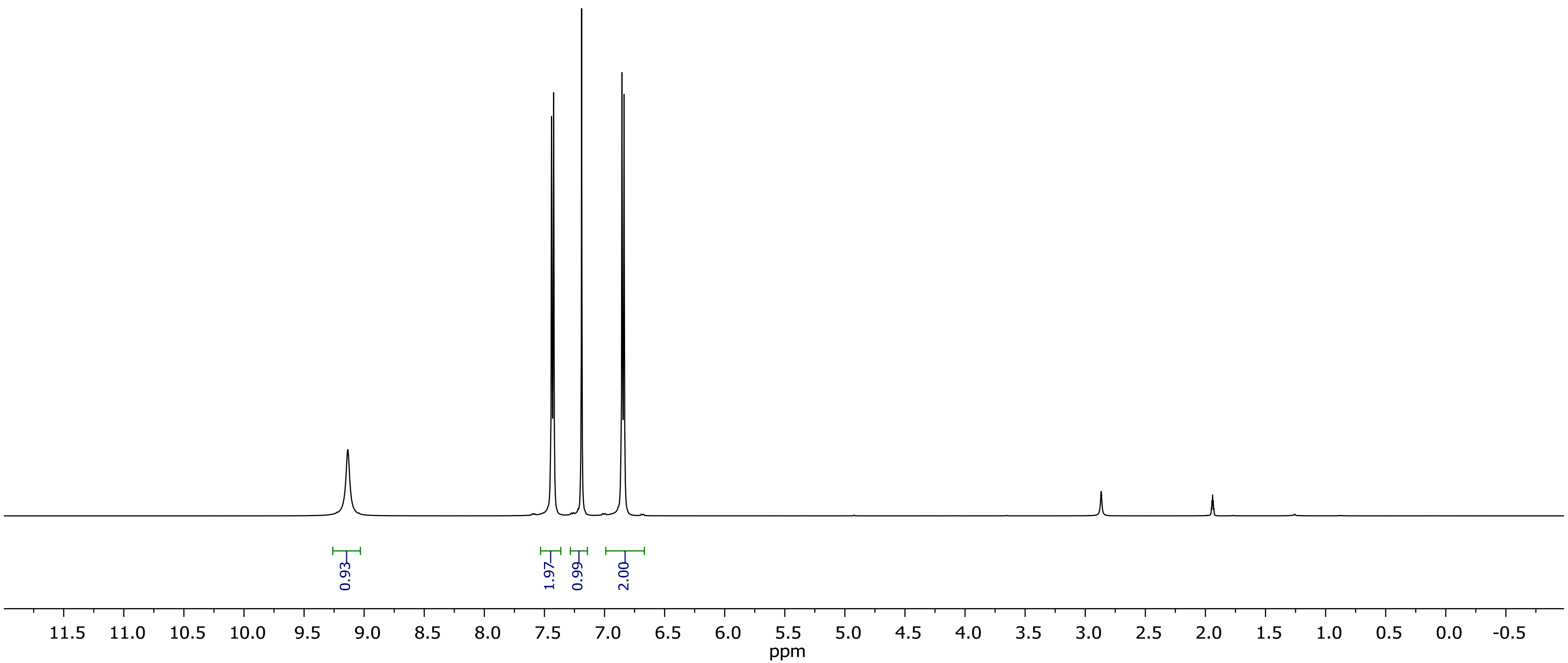

^{13}C NMR of 2,2,2-trifluoro-N-(4-hydroxyphenyl)acetamide (4)

CD_3CN, 25 °C

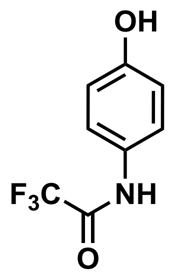

4

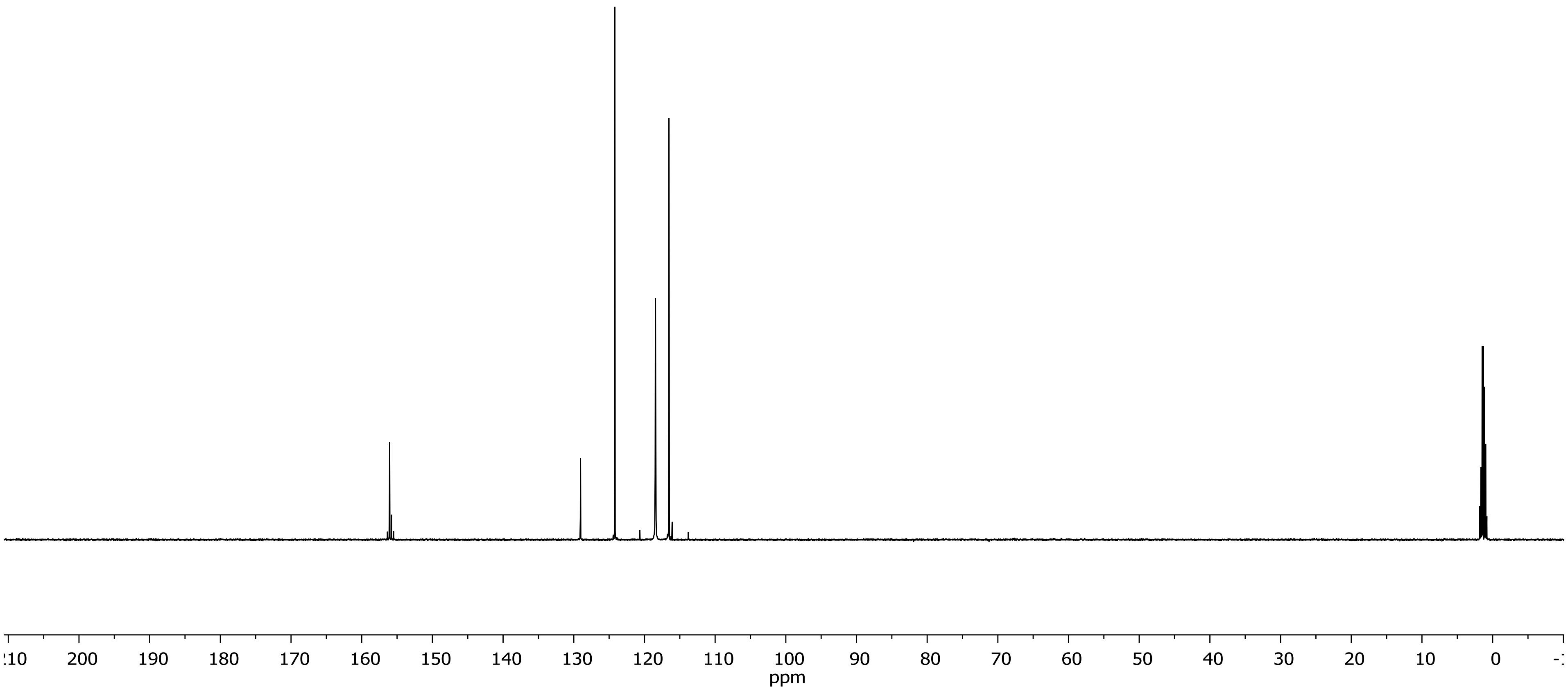

^{19}F NMR of 2,2,2-trifluoro-N-(4-hydroxyphenyl)acetamide (4)

CD_3CN, 25 °C

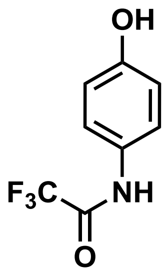

4

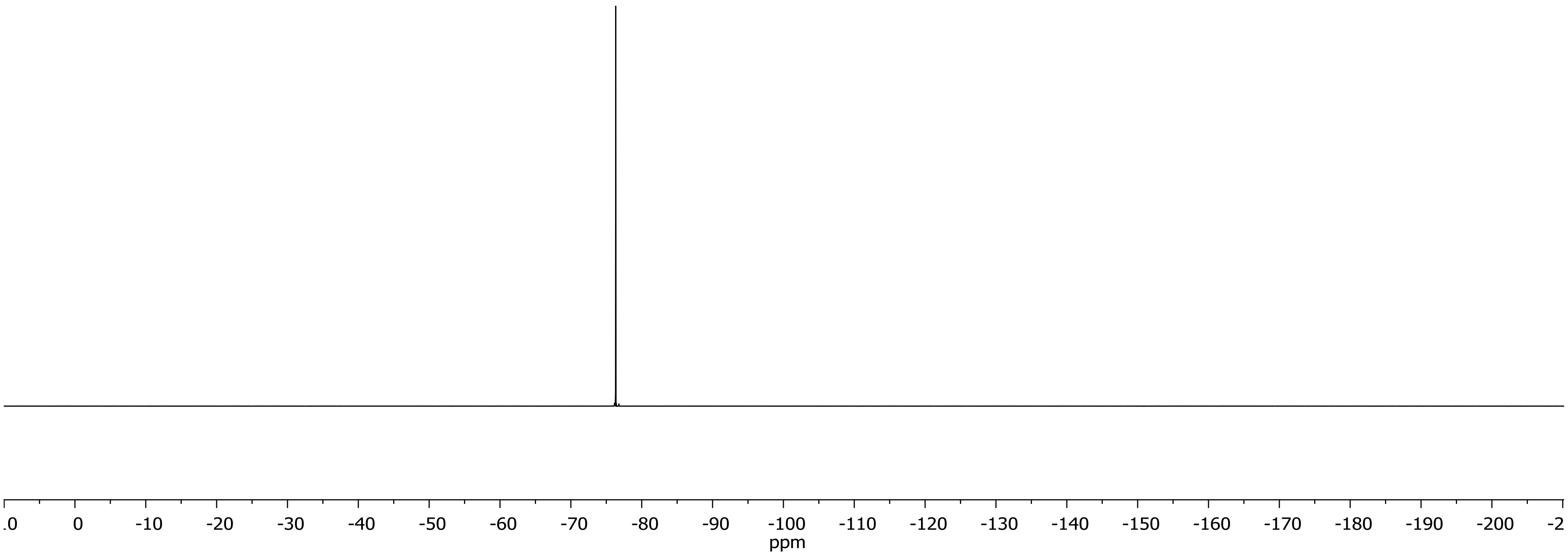

^{1}H NMR of methyl acetyl-L-phenylalate tetrafluorothianthrenium salt (5-TFT)

CD_3CN, 25 °C

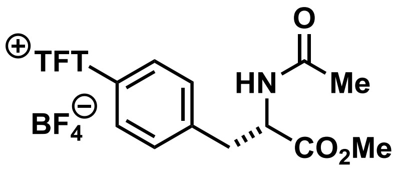

5-TFT

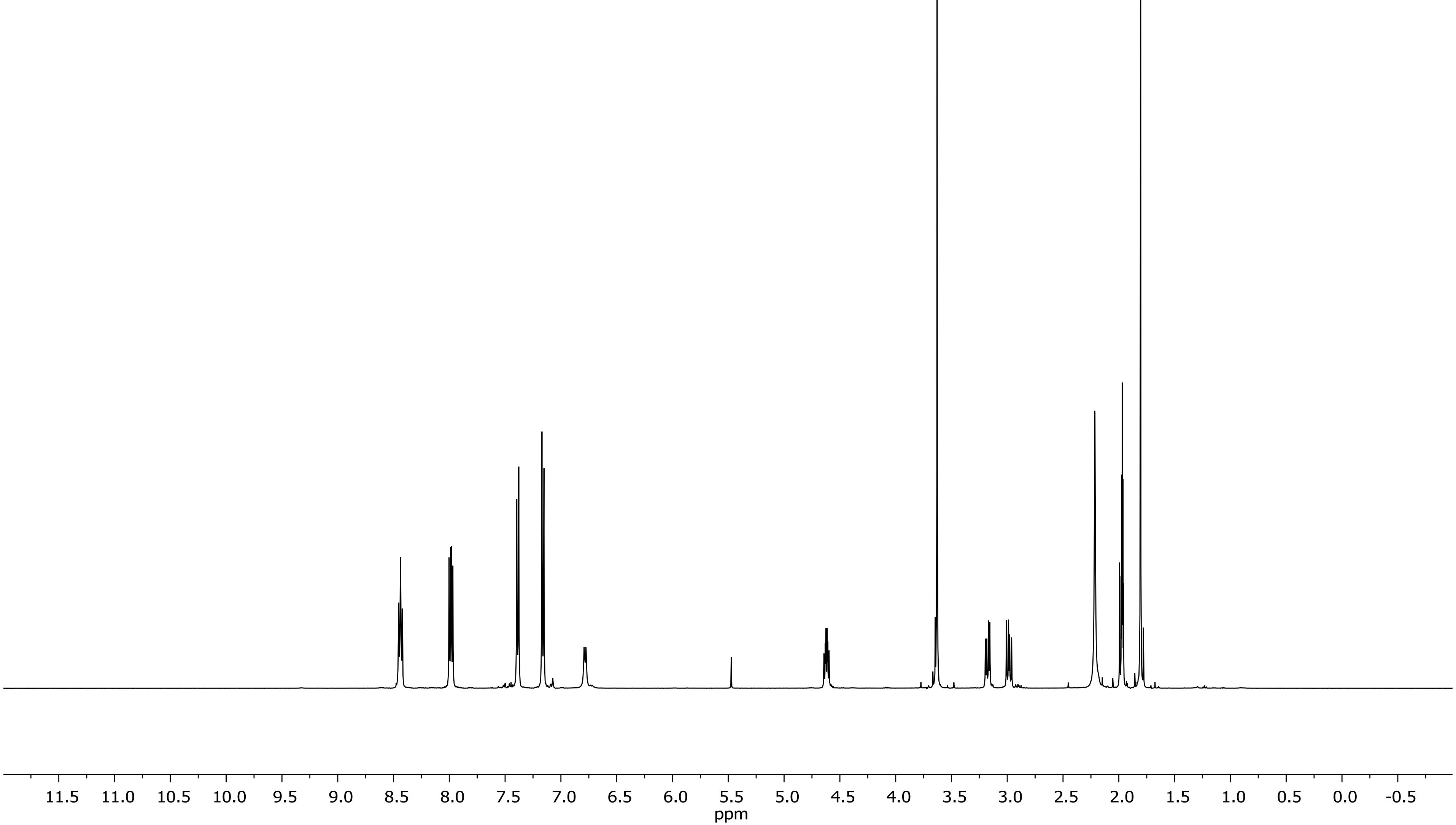

^{13}C NMR of methyl acetyl-L-phenylalate tetrafluorothianthrenium salt (5-TFT)

CD_3CN, 25 °C

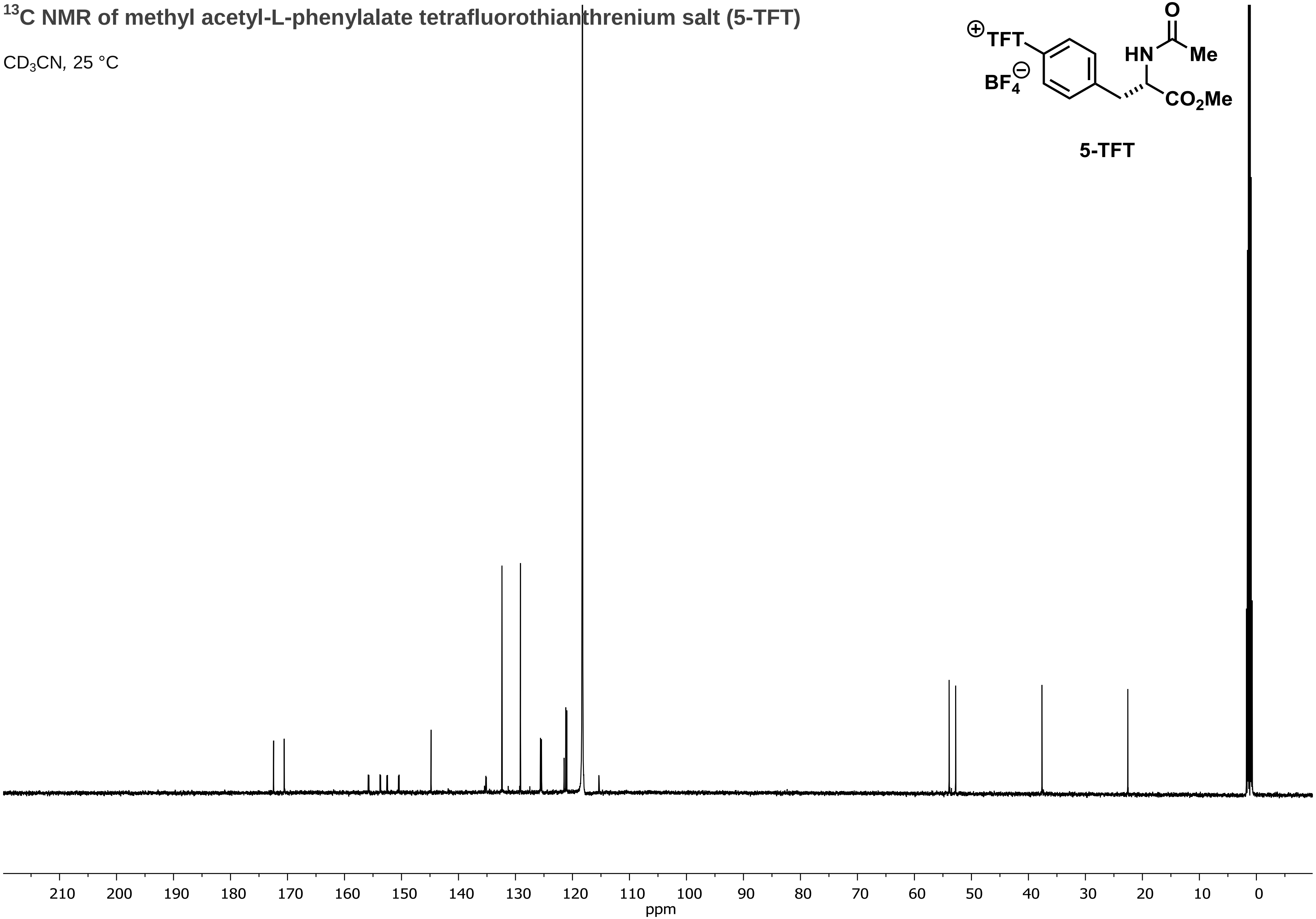

^{19}F NMR of methyl acetyl-L-phenylalate tetrafluorothianthrenium salt (5-TFT)

CD_3CN, 25 °C

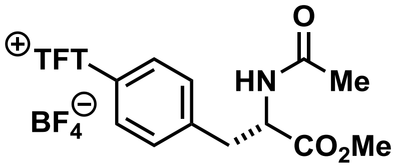

5-TFT

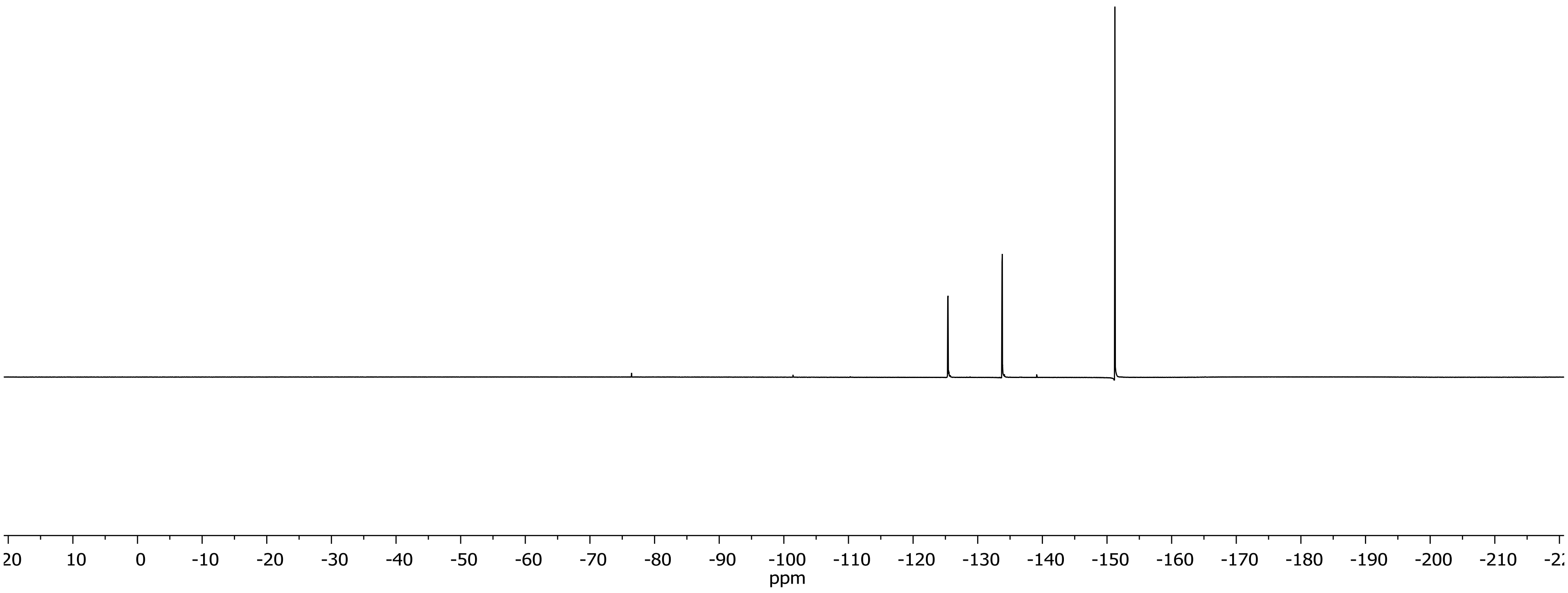

^{1}H NMR of methyl acetyl-L-tyrosinate (5)

CD_3CN, 25 °C

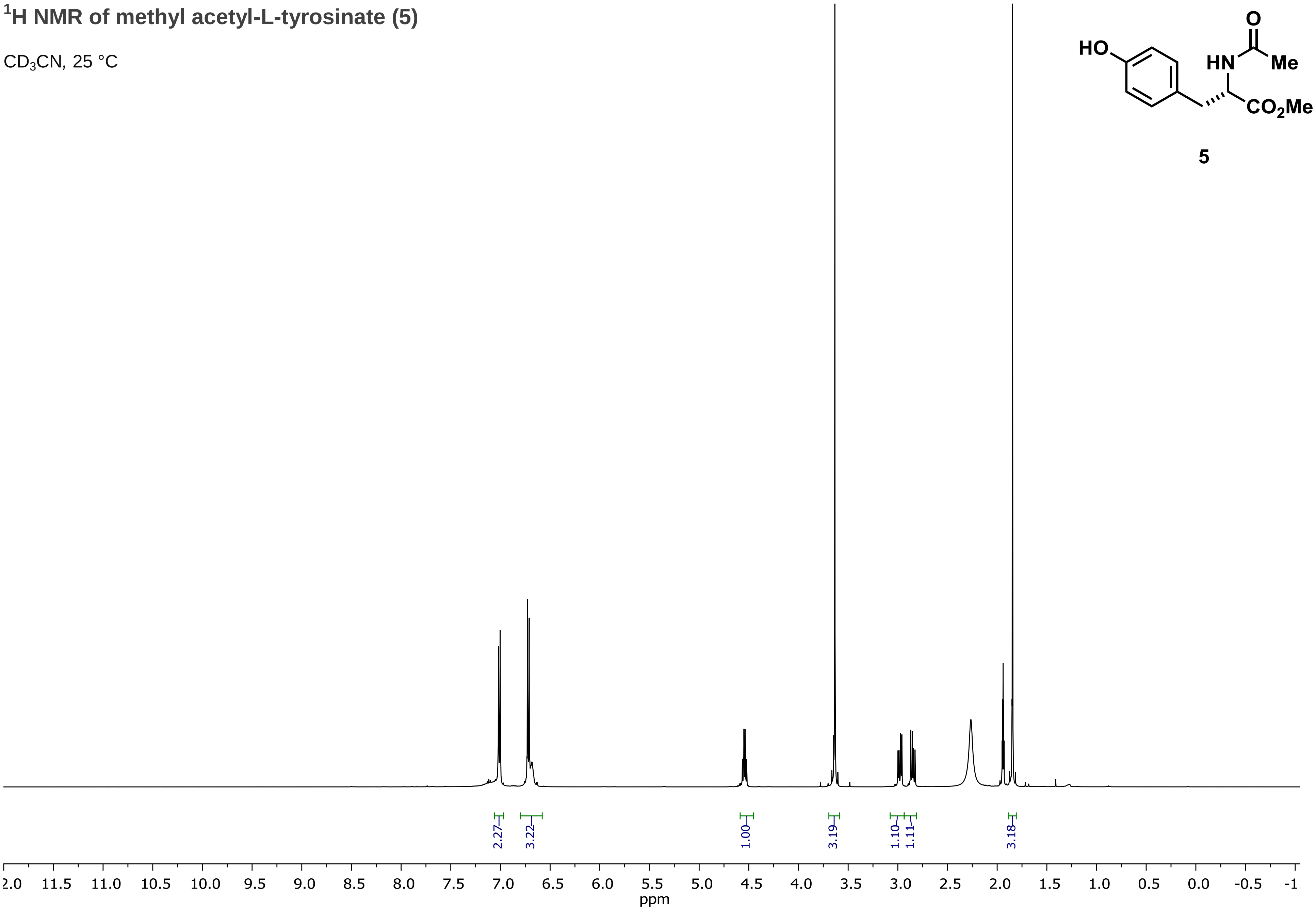

^{13}C NMR of methyl acetyl-L-tyrosinate (5)

CD_3CN, 25 °C

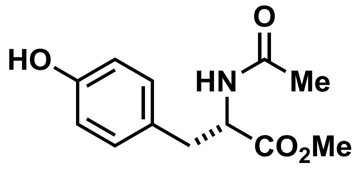

5

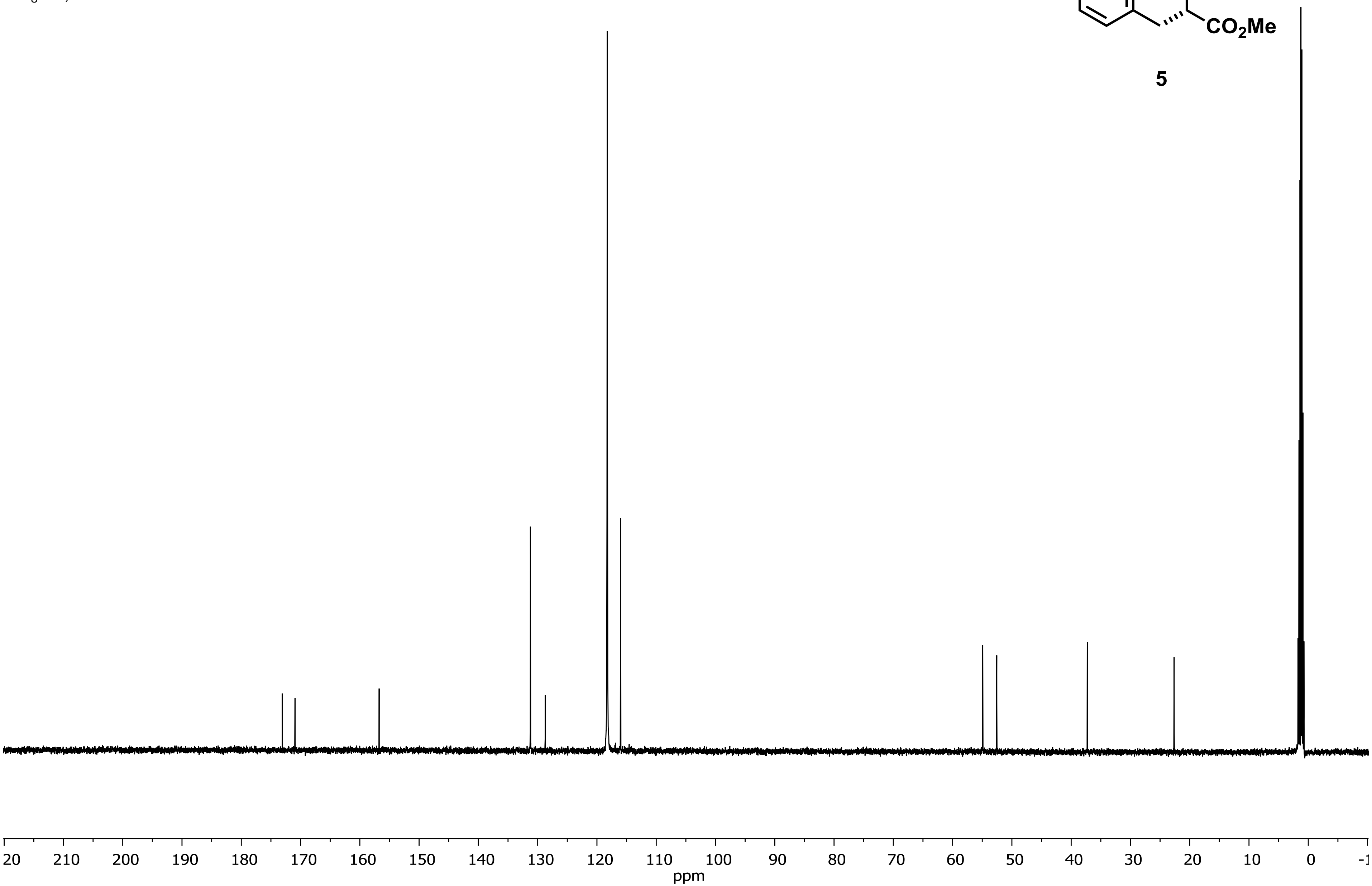

1H NMR of hydroxy-nefiracetam (6)

CD_3OD, 25 °C

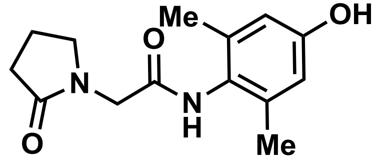

6

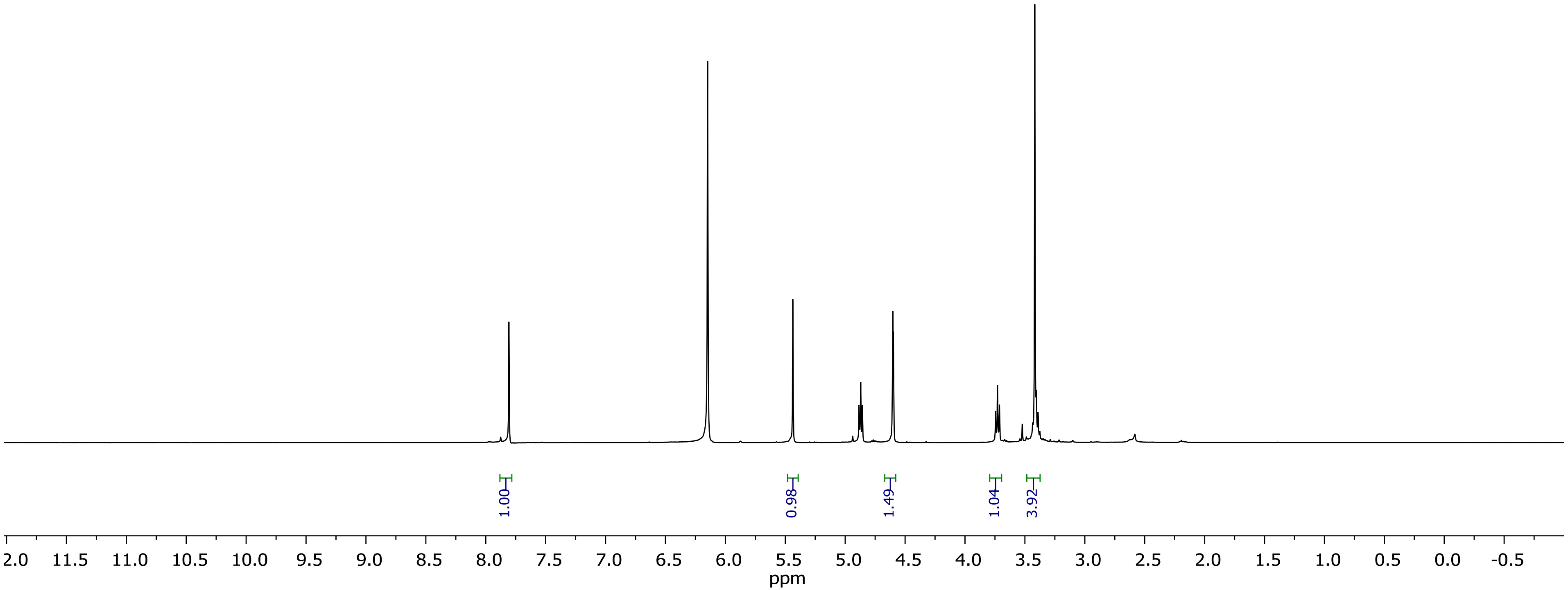

^{13}C NMR of hydroxy-nefiracetam (6)

CD_3OD, 25 °C

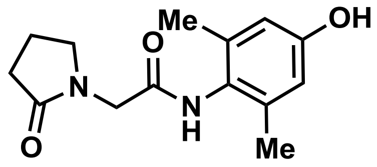

6

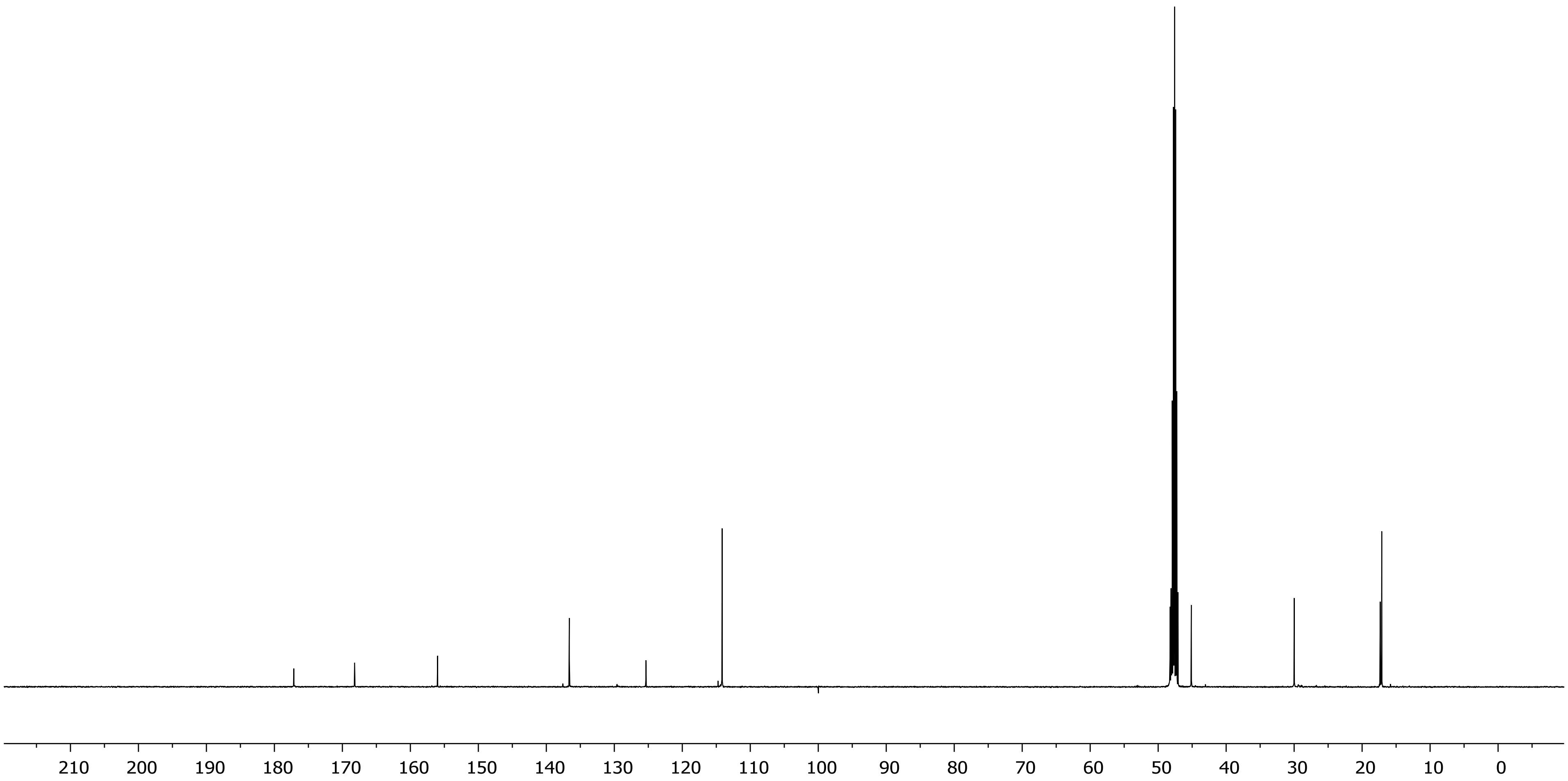

^{1}H NMR of bromobenzene tetrafluorothianthrenium salt (7-TFT)

CD_2Cl_2, 25 °C

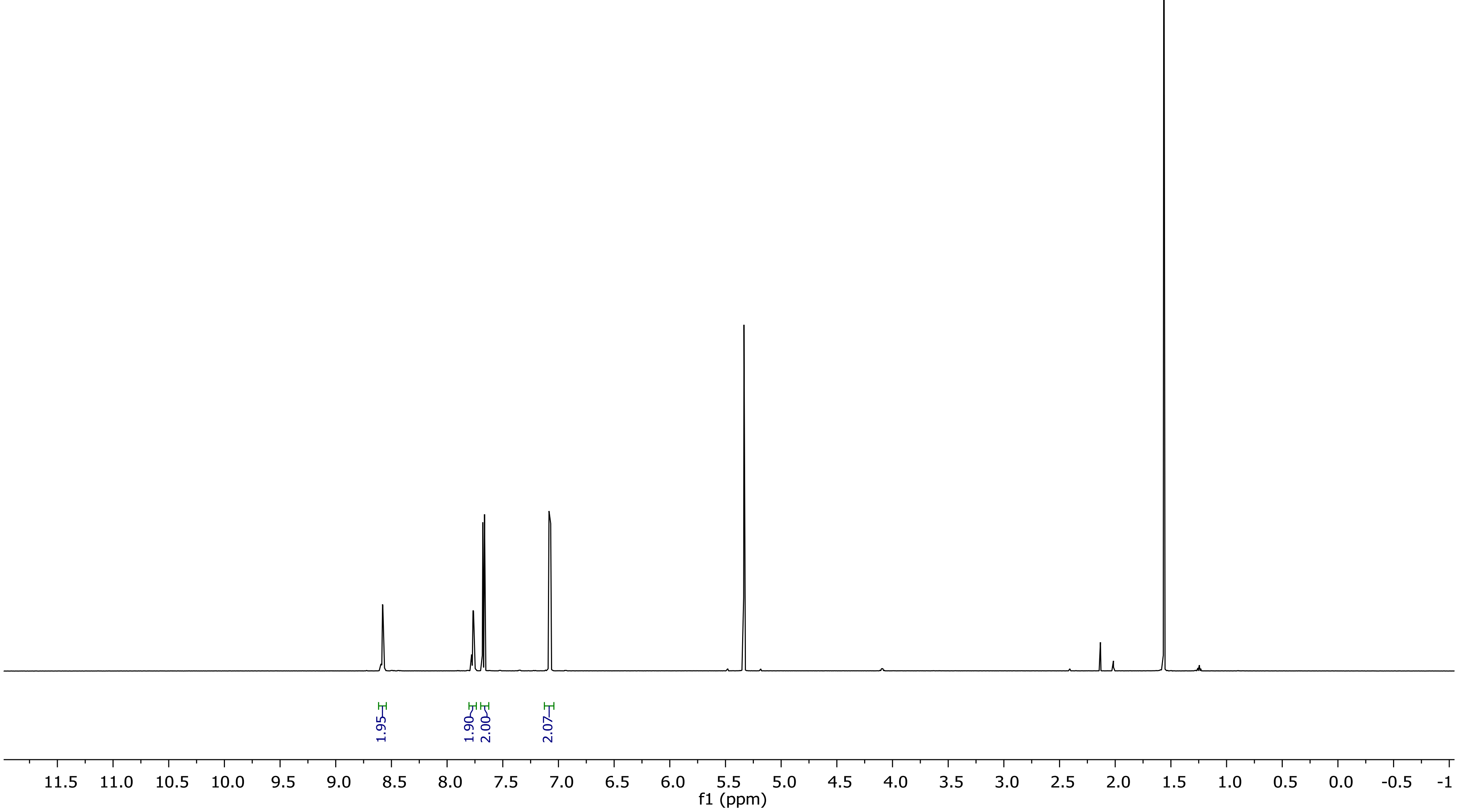

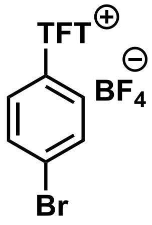

7-TFT

^{13}C NMR of bromobenzene tetrafluorothianthrenium salt (7-TFT)

CD_2Cl_2, 25 °C

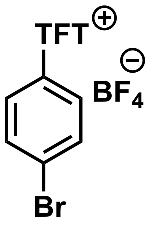

7-TFT

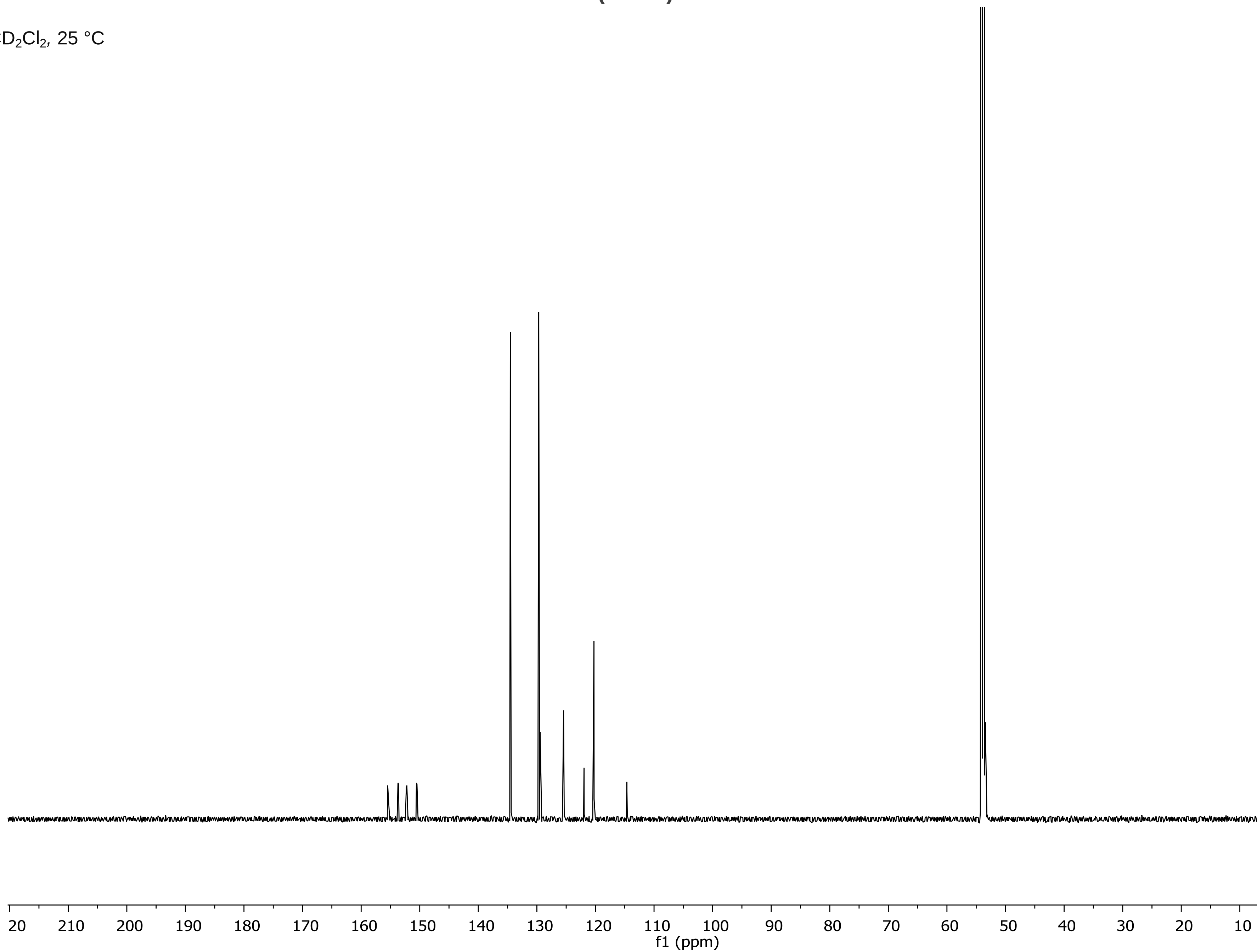

^{19}F NMR of bromobenzene tetrafluorothianthrenium salt (7-TFT)

CD_2Cl_2, 25 °C

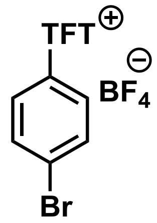

7-TFT

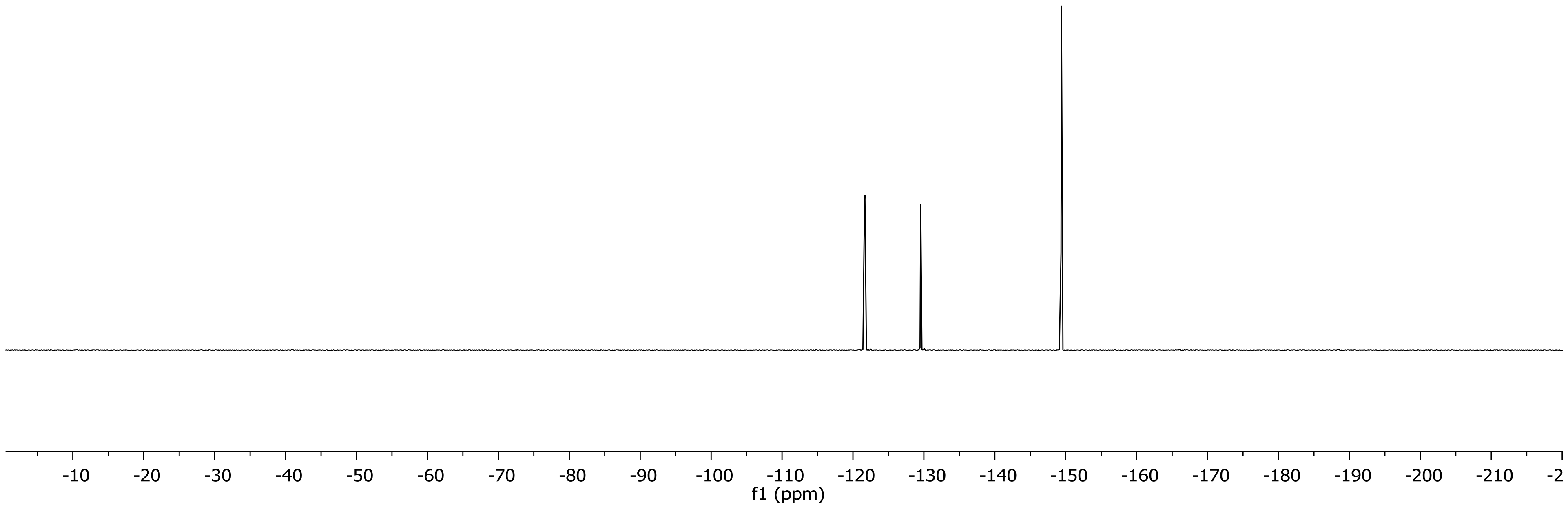

^{1}H NMR of 4-bromophenol (7)

$CDCl_3$, 25 °C

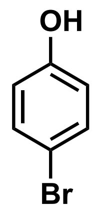

7

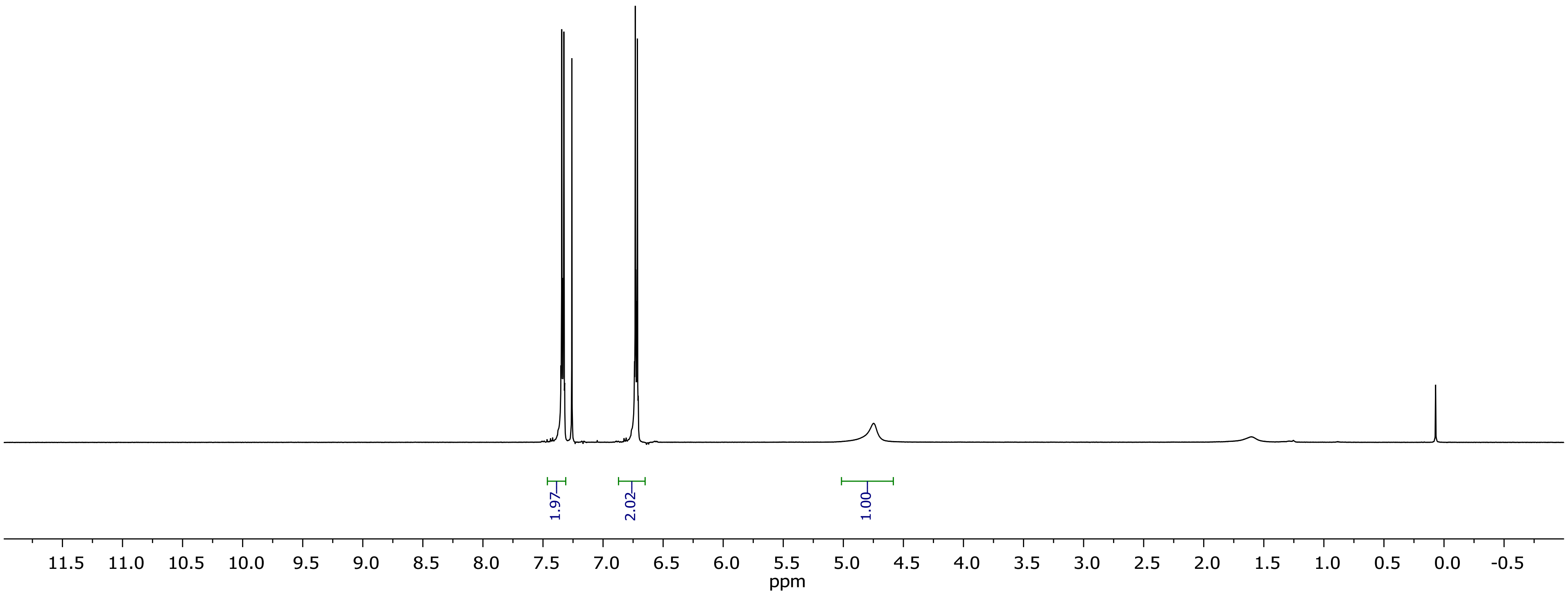

^{13}C NMR of 4-bromophenol (7)

CDCl$_3$, 25 °C

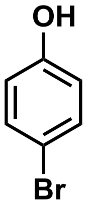

7

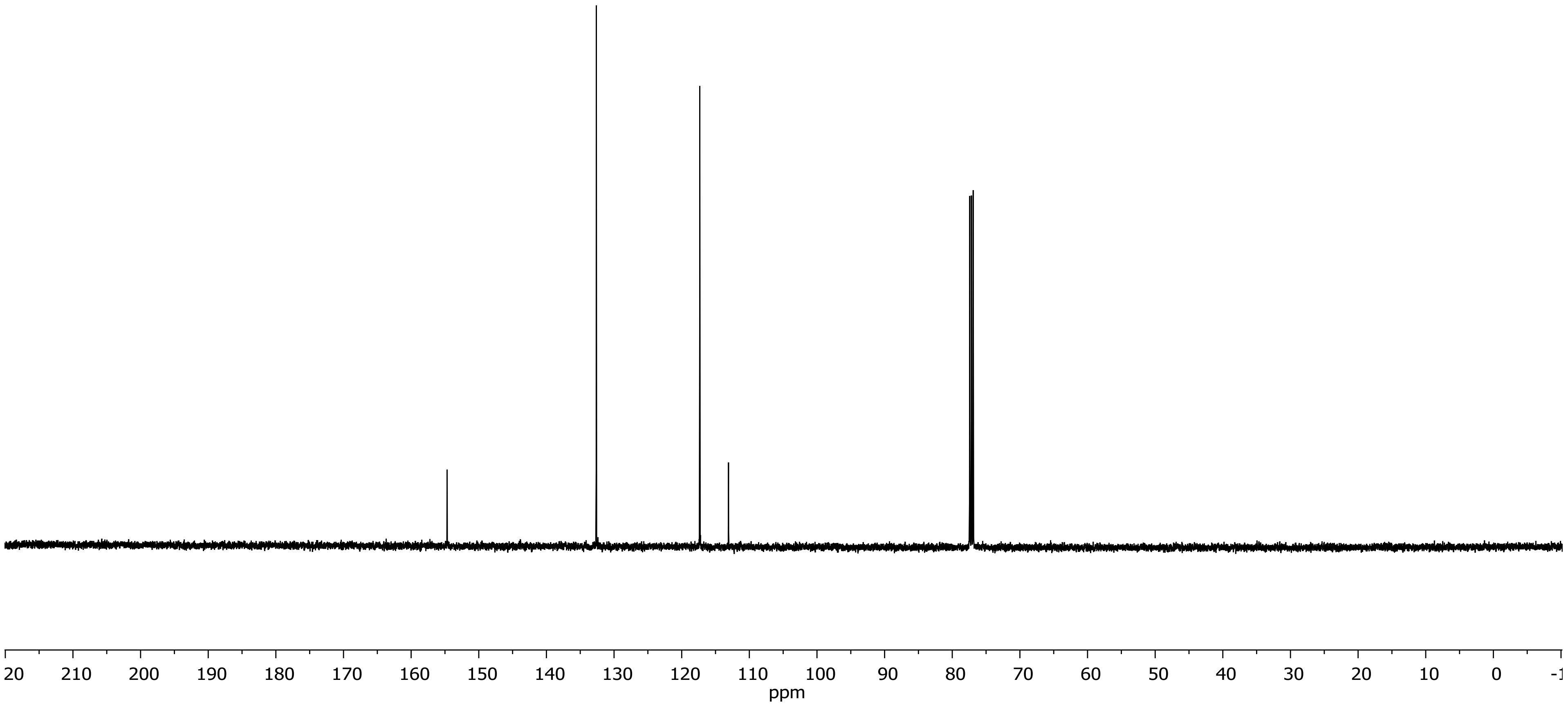

^{1}H NMR of methyl 2-methoxybenzoate tetrafluorothianthrenium salt (8-TFT)

CD$_3$CN, 25 °C

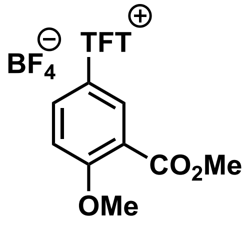

8-TFT

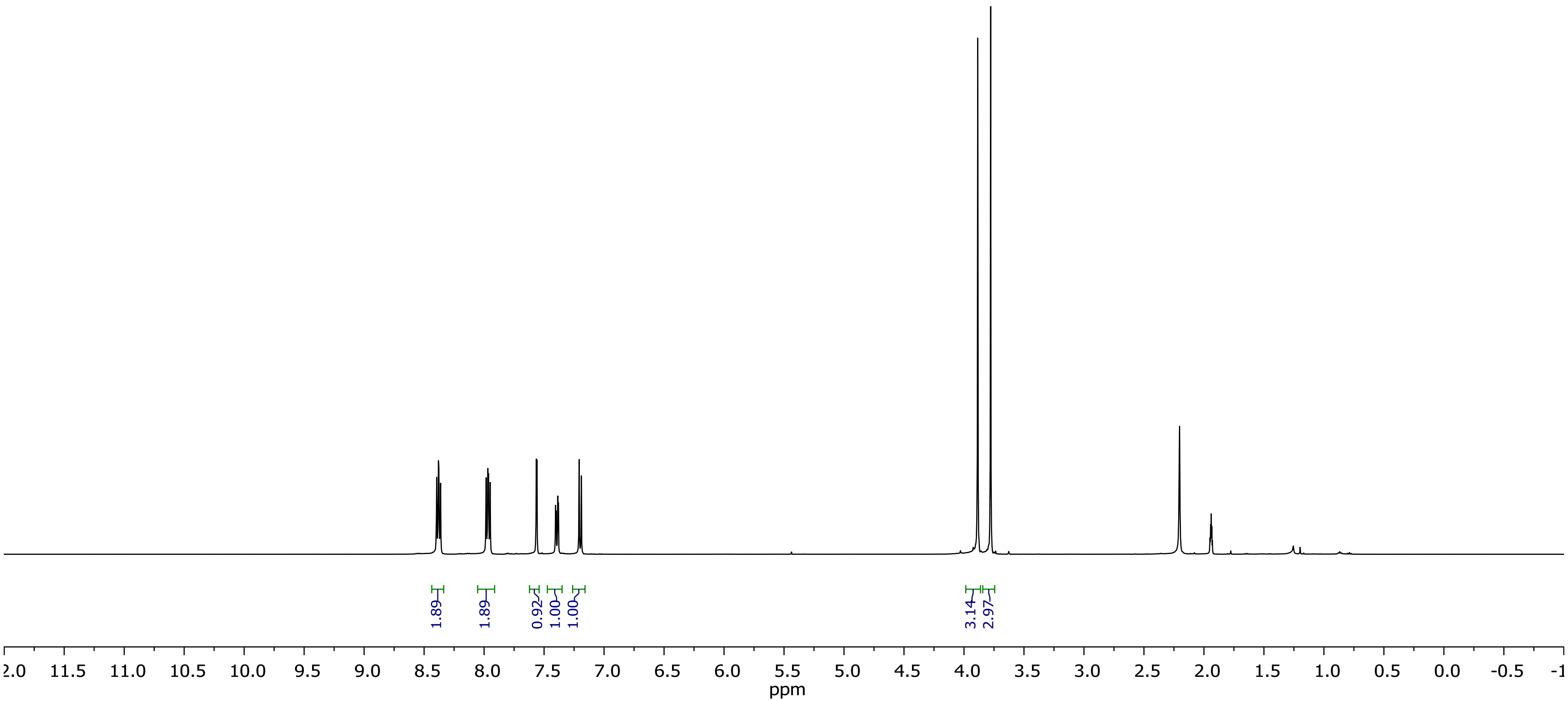

^{13}C NMR of methyl 2-methoxybenzoate tetrafluorothianthrenium salt (8-TFT)

CD_3CN, 25 °C

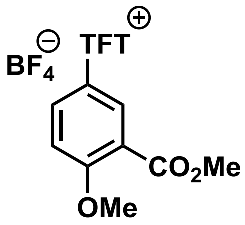

8-TFT

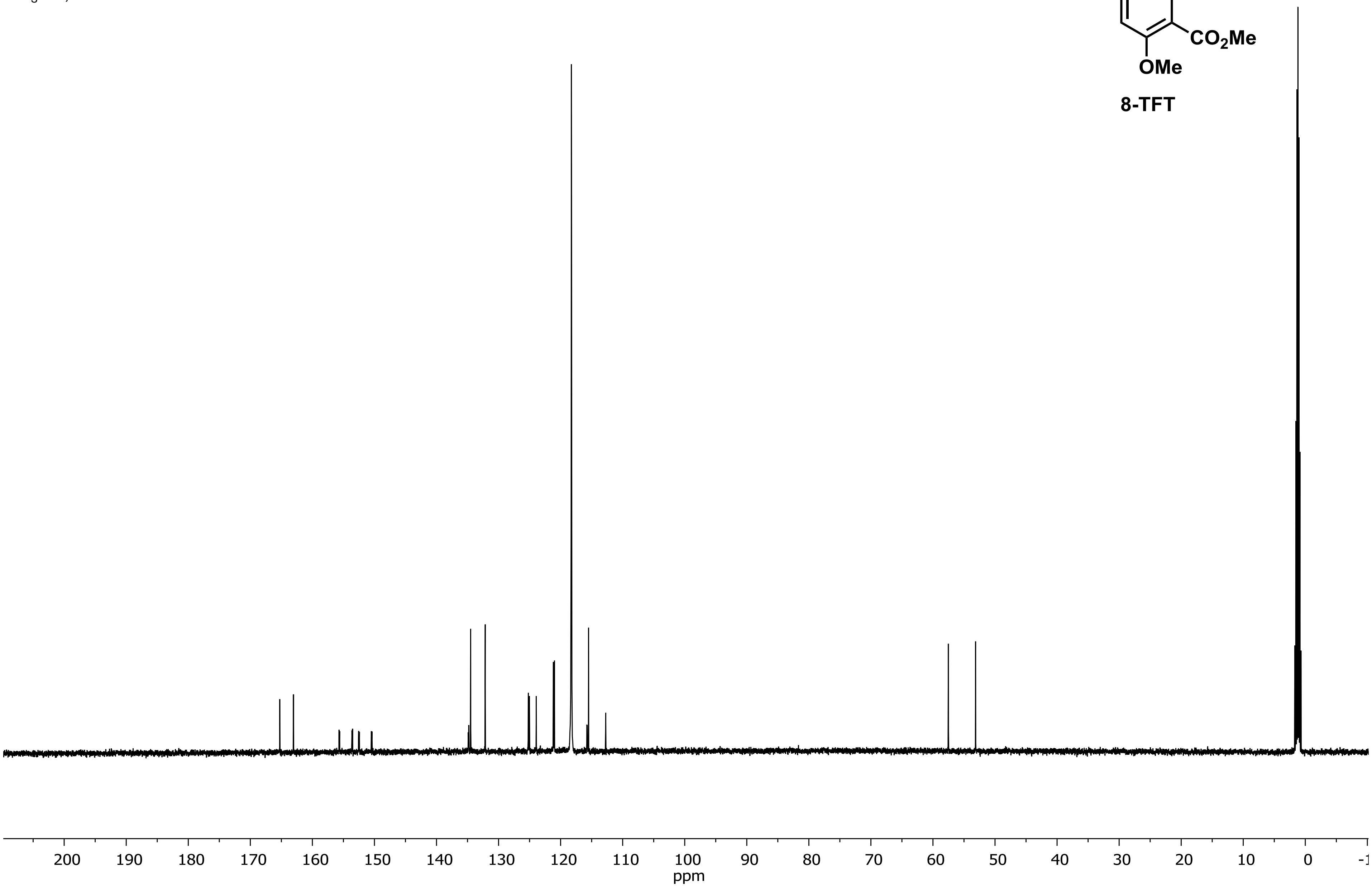

^{19}F NMR of methyl 2-methoxybenzoate tetrafluorothianthrenium salt (8-TFT)

CD_3CN, 25 °C

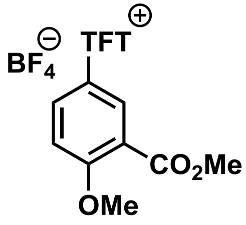

8-TFT

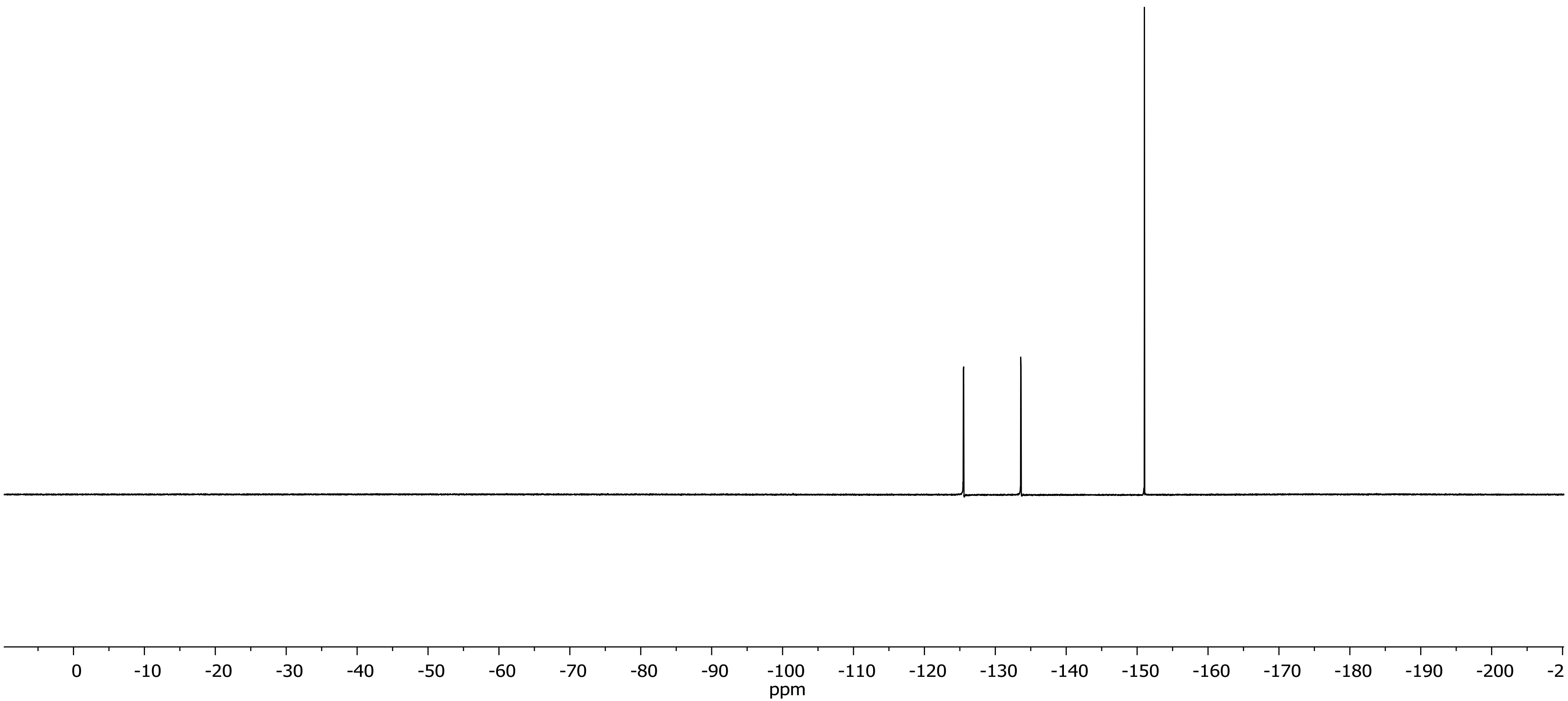

^{1}H NMR of methyl 5-hydroxy-2-methoxybenzoate (8)

CDCl$_3$, 25 °C

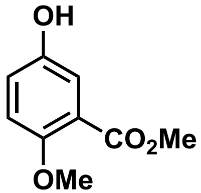

8

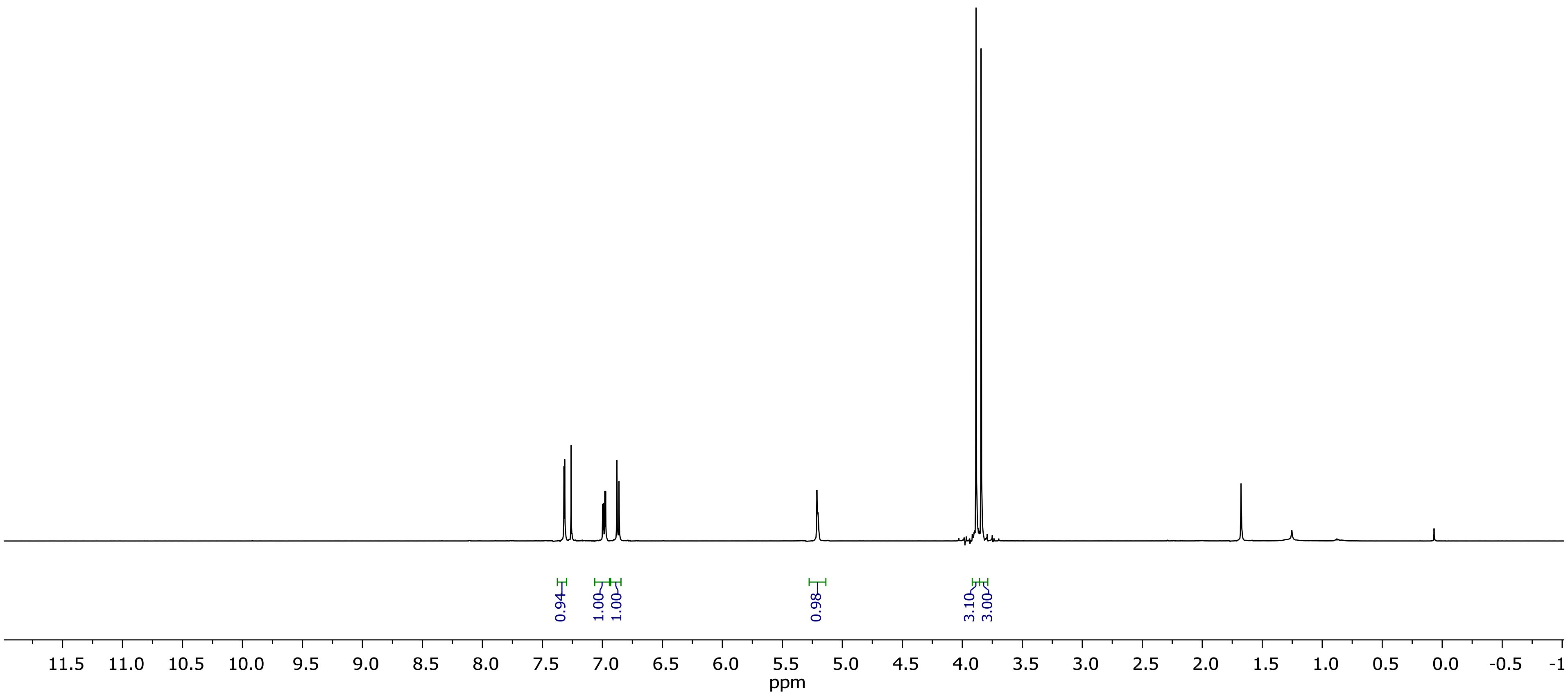

^{13}C NMR of methyl 5-hydroxy-2-methoxybenzoate (8)

$CDCl_3$, 25 °C

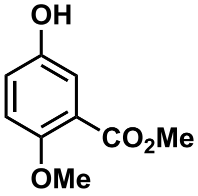

8

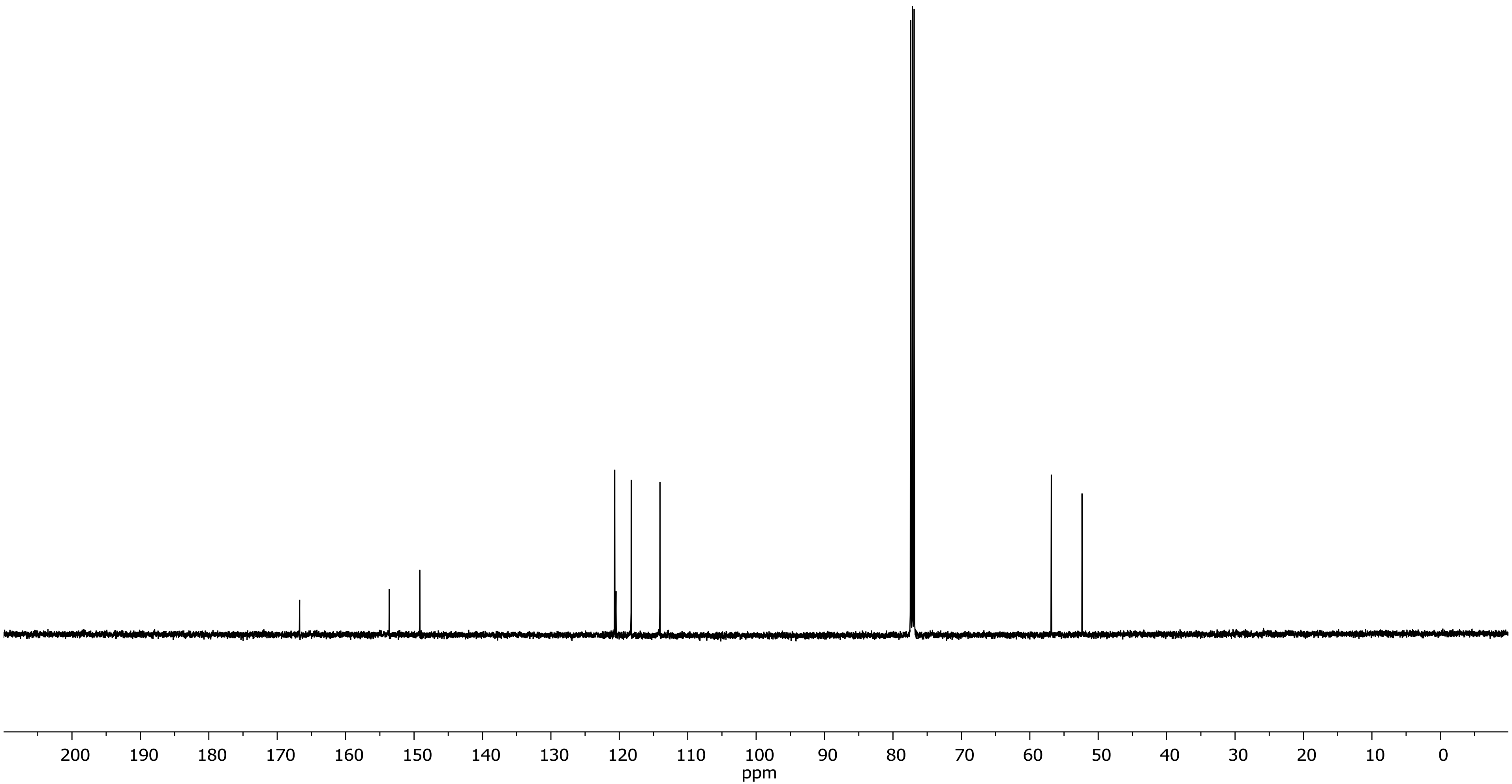

^{1}H NMR of xanthone tetrafluorothianthrenium salt (9-TFT)

CD_3CN, 25 °C

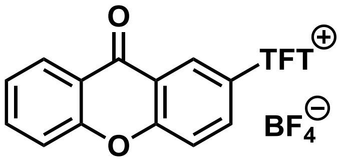

9-TFT

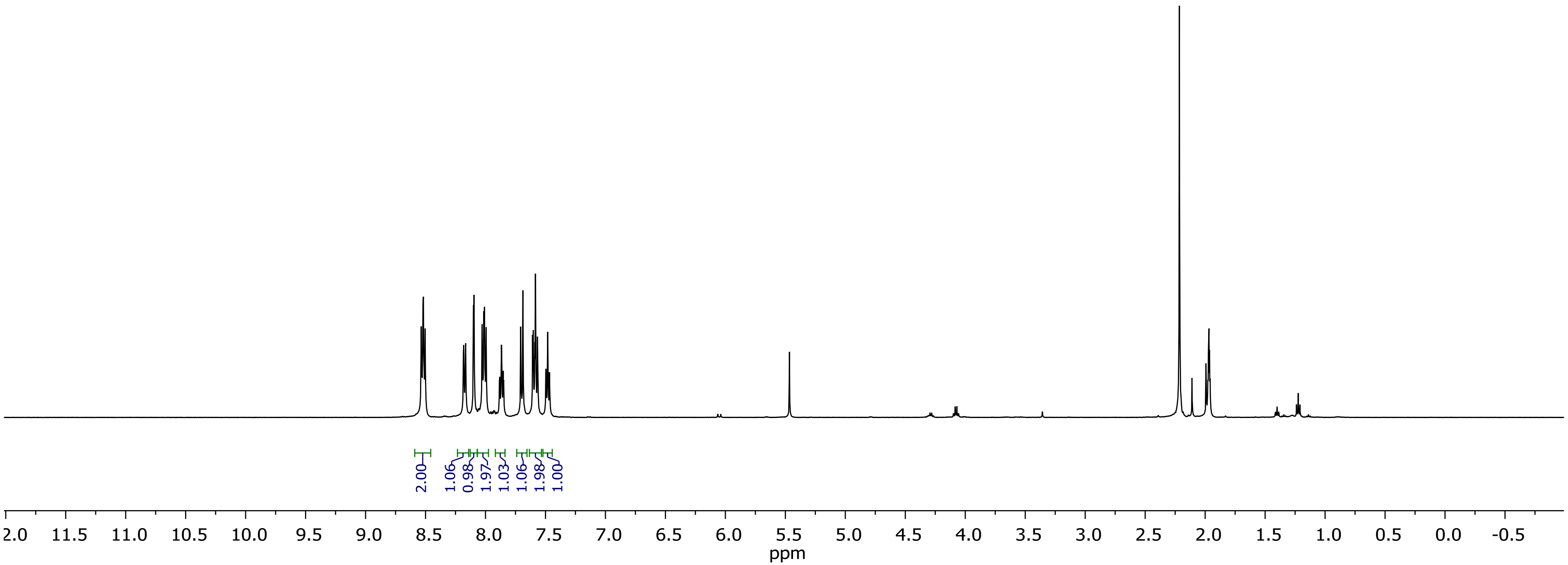

^{13}C NMR of xanthone tetrafluorothianthrenium salt (9-TFT)

CD_3CN, 25 °C

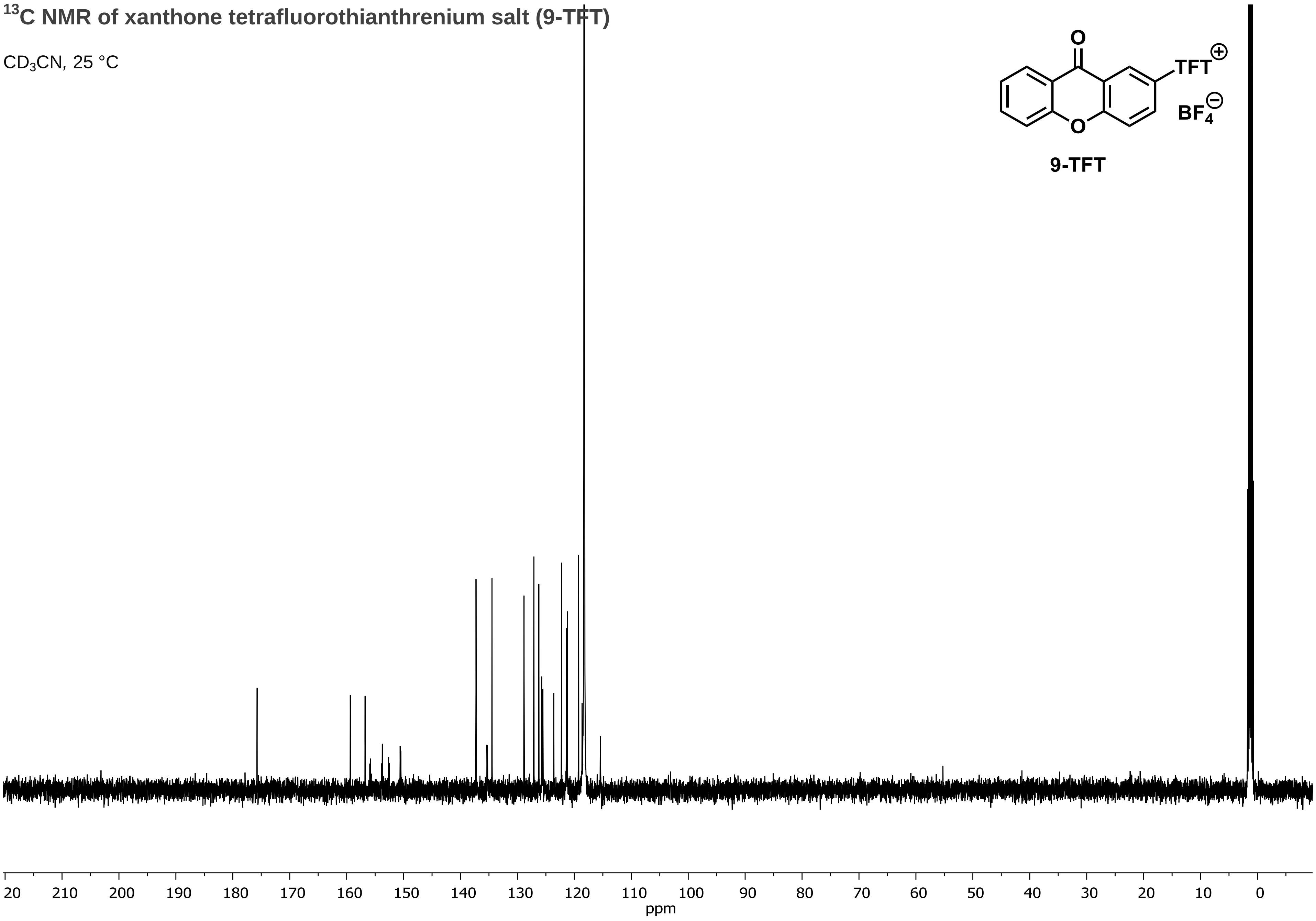

^{19}F NMR of xanthone tetrafluorothianthrenium salt (9-TFT)

CD_3CN, 25 °C

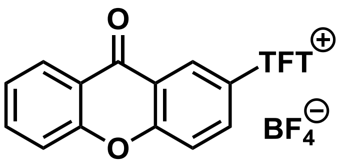

9-TFT

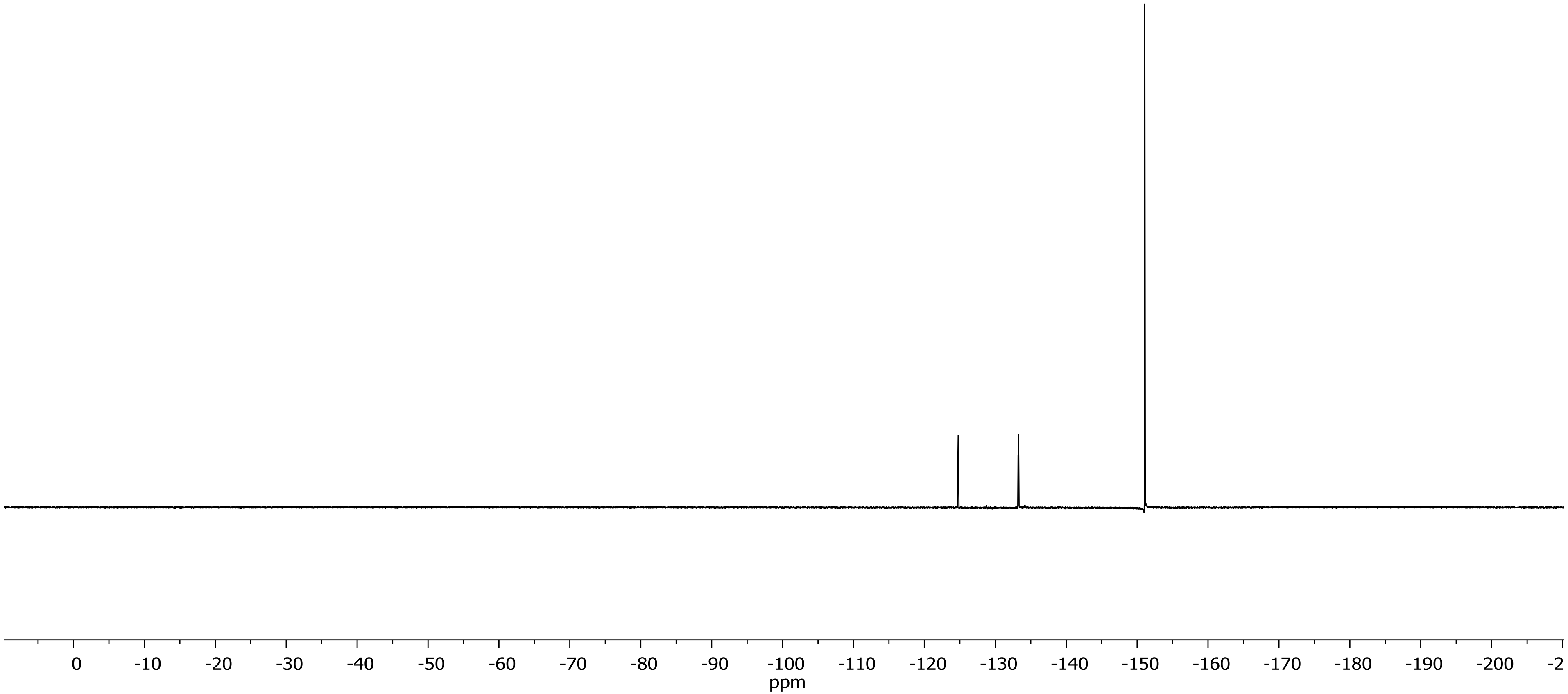

^{1}H NMR of hydroxyl xanthone (9)

CD_3CN, 25 °C

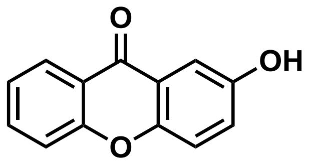

9

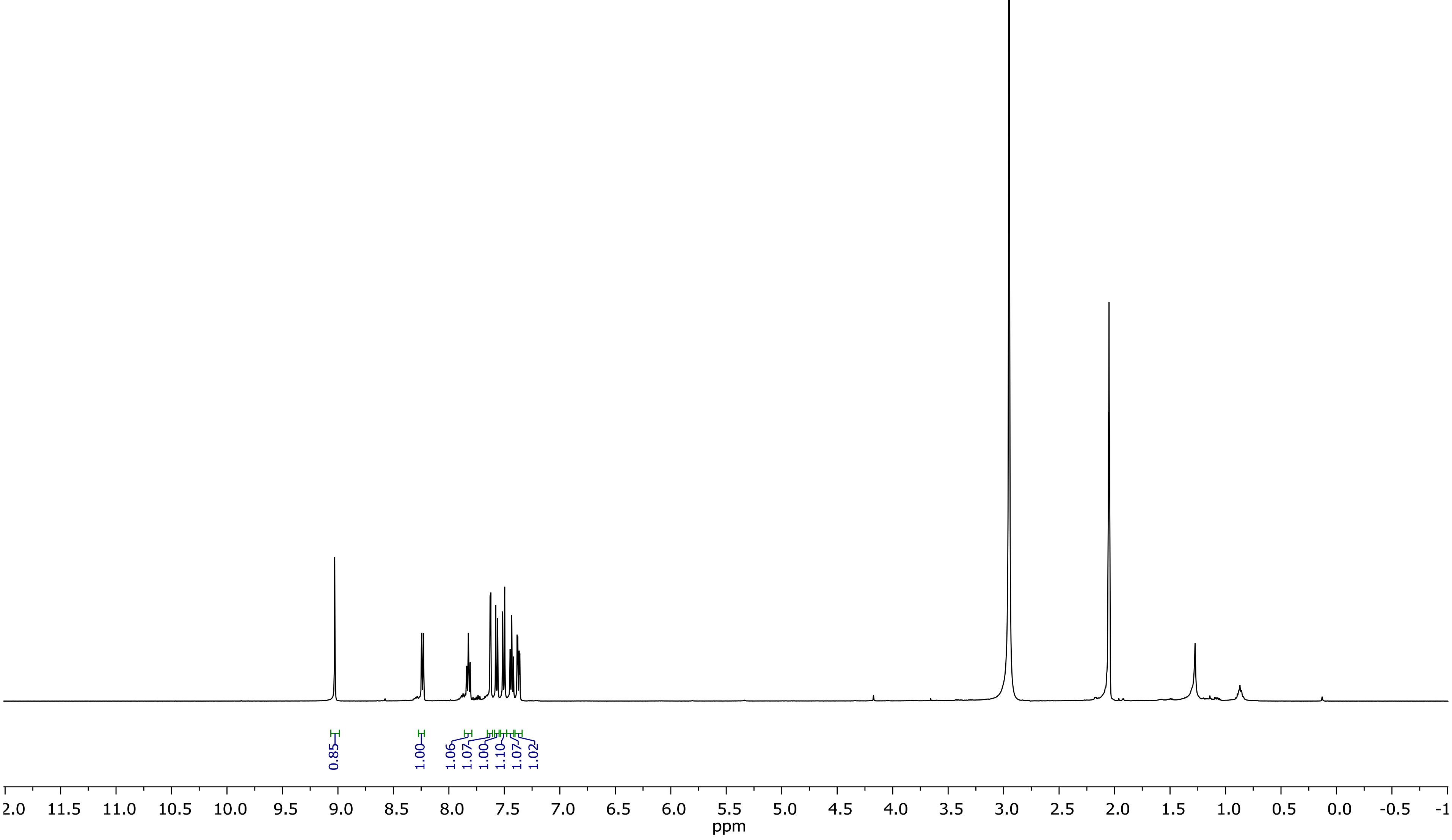

^{13}C NMR of hydroxyl xanthone (9)

$CO(CD_3)_2$, 25 °C

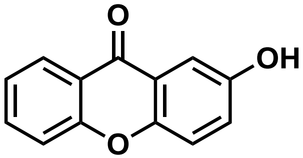

9

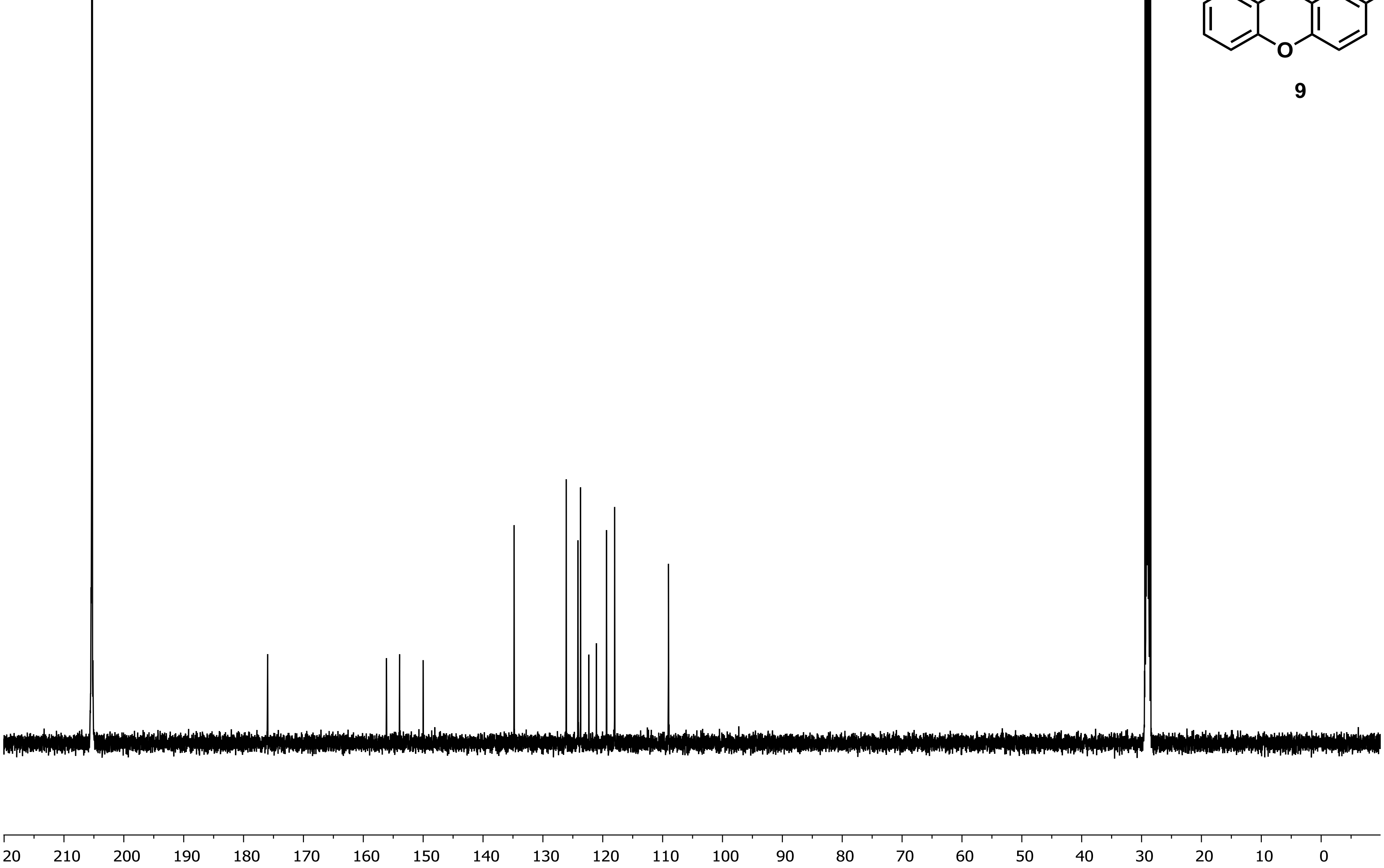

^{1}H NMR of (*R*)-4-(4-hydroxybenzyl)-3-propionyloxazolidin-2-one (10)

CD_3CN, 25 °C

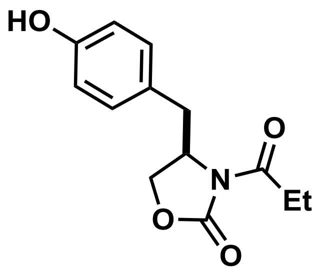

10

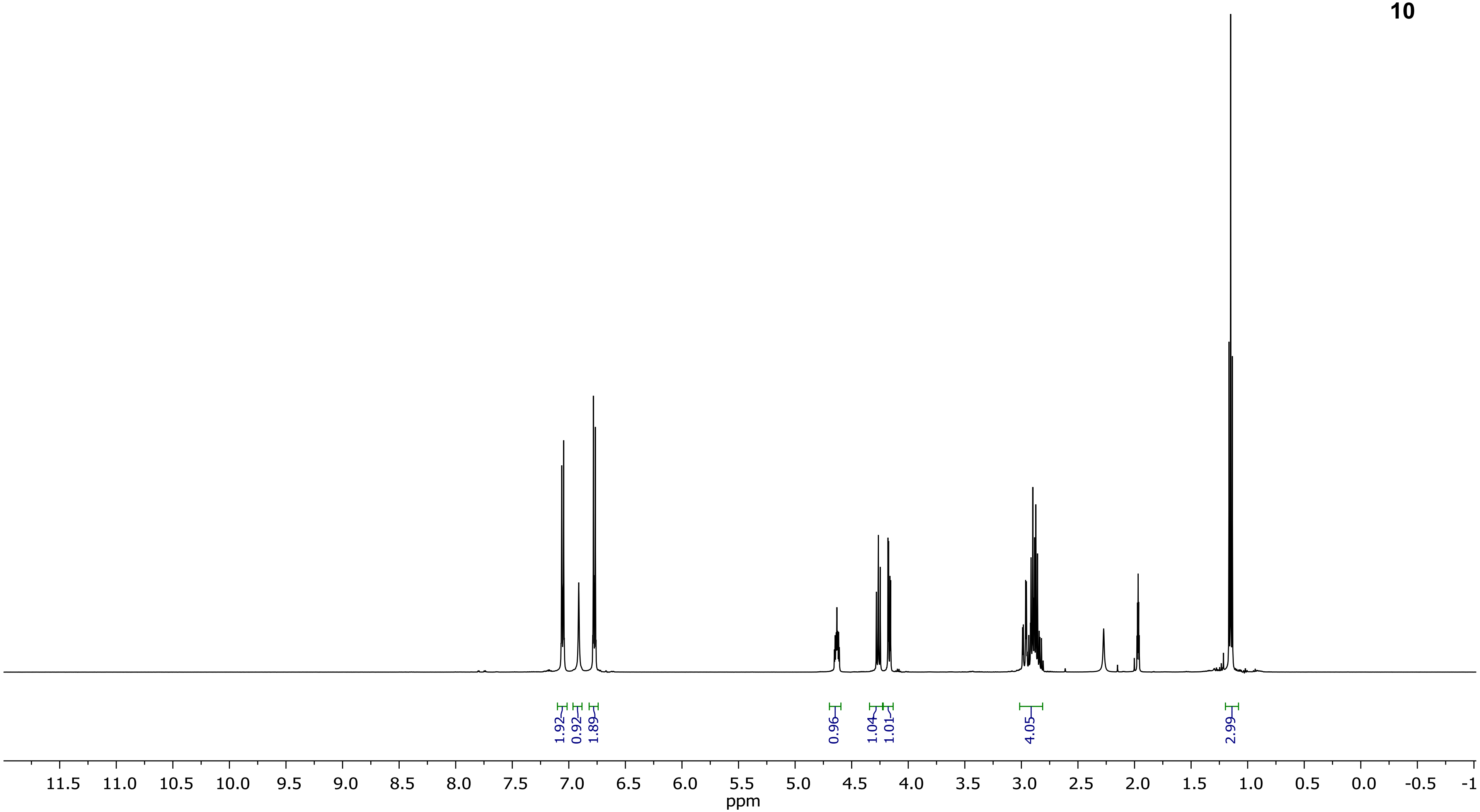

^{13}C NMR of (*R*)-4-(4-hydroxybenzyl)-3-propionyloxazolidin-2-one (10)

CD_3CN, 25 °C

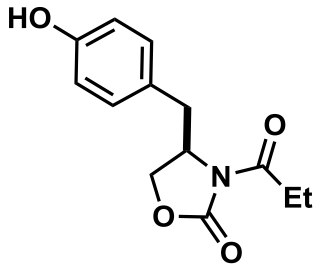

10

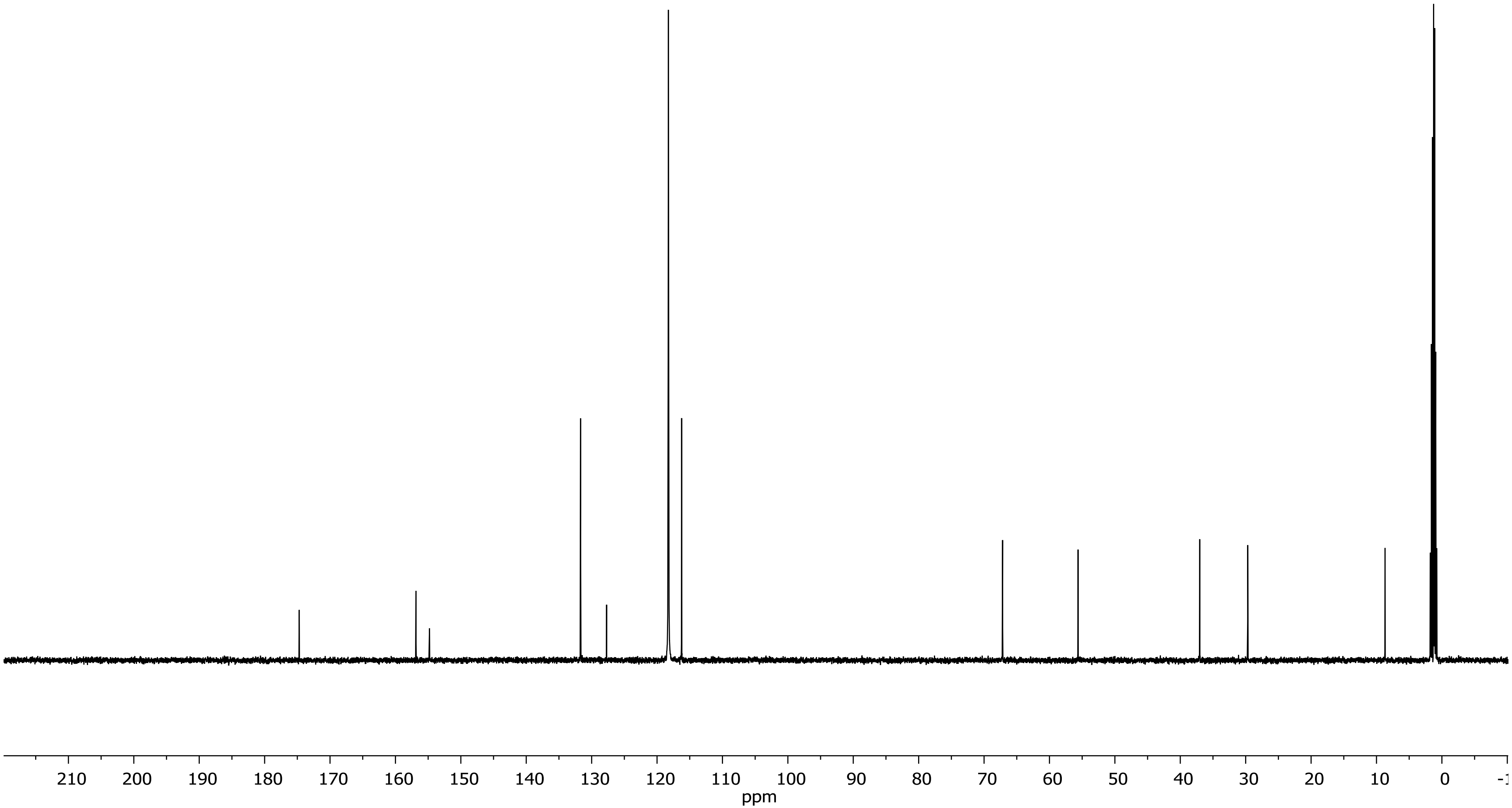

^{1}H NMR of hydroxy-boscalid (11)

CD_3CN, 25 °C

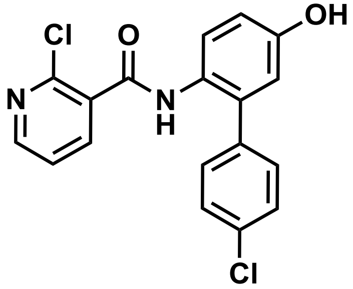

11

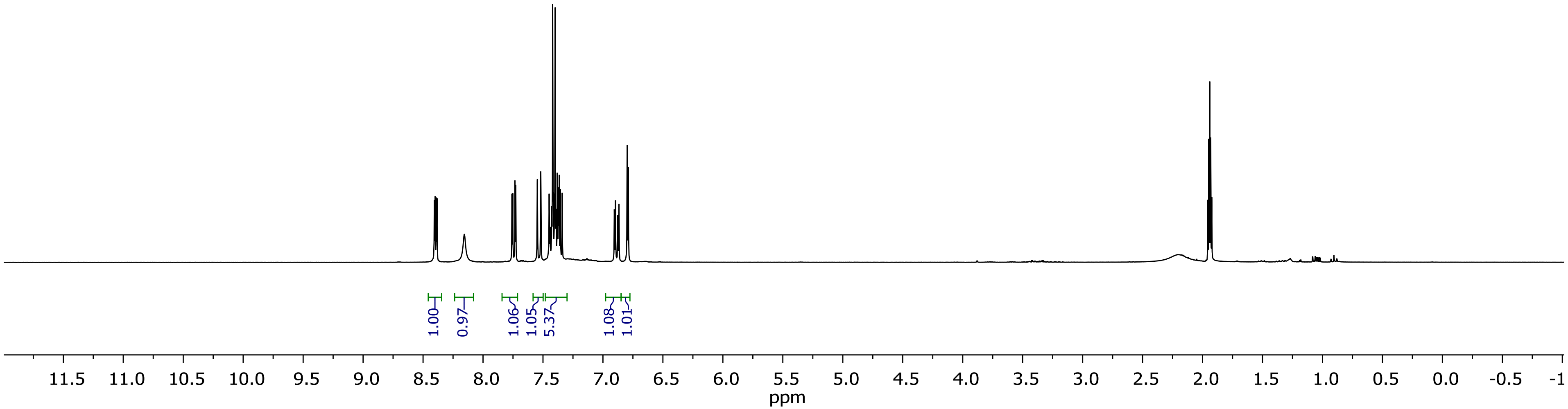

^{13}C NMR of hydroxy-boscalid (11)

CD_3CN, 25 °C

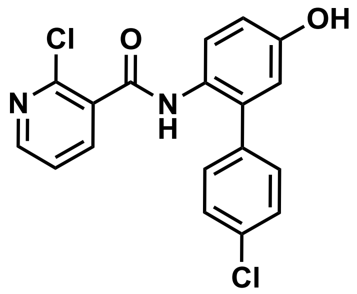

11

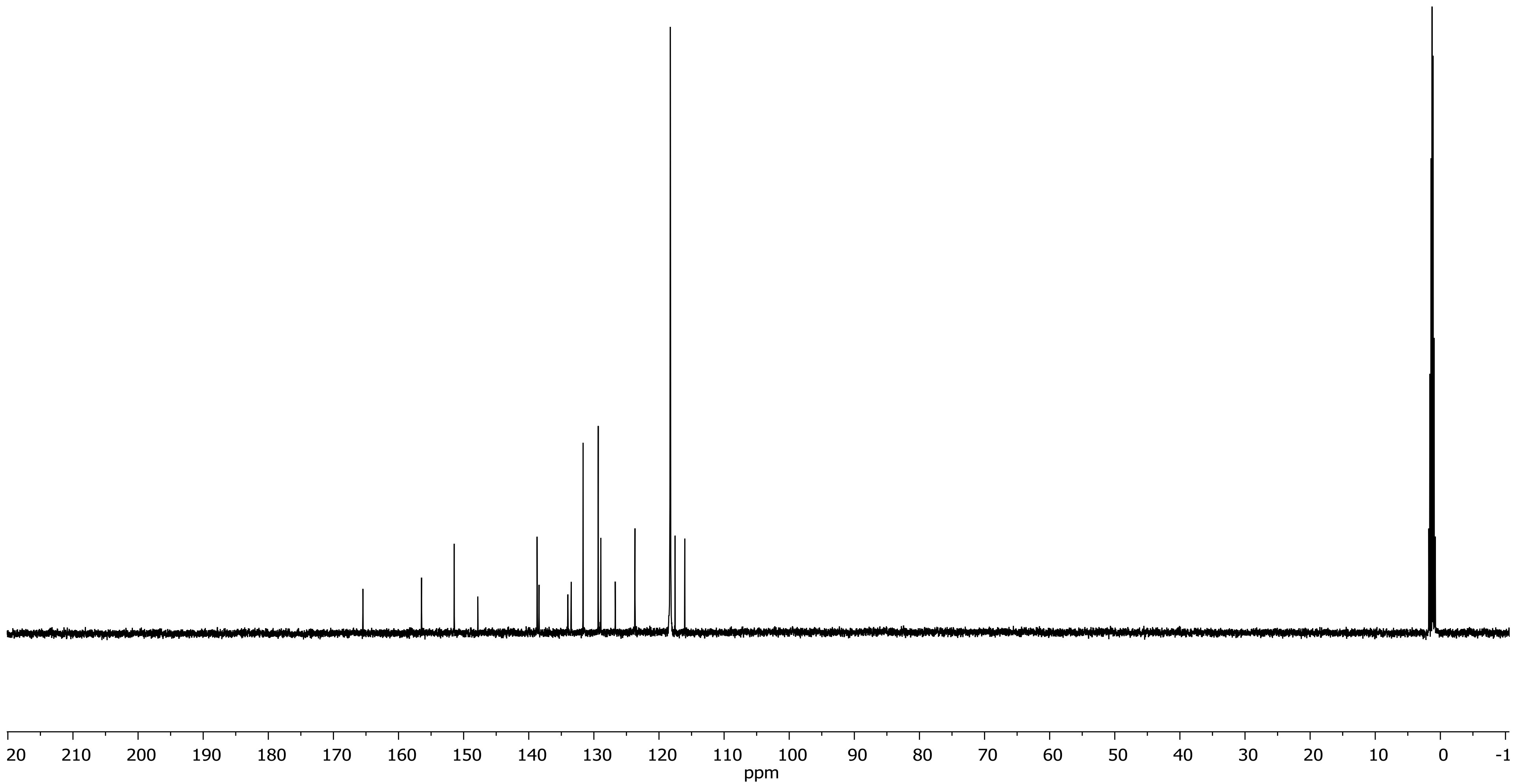

^{1}H NMR of methyl 2,4-dimethylbenzoate tetrafluorothianthrenium salt (12-TFT)

CD_3CN, 25 °C

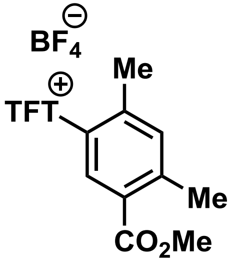

12-TFT

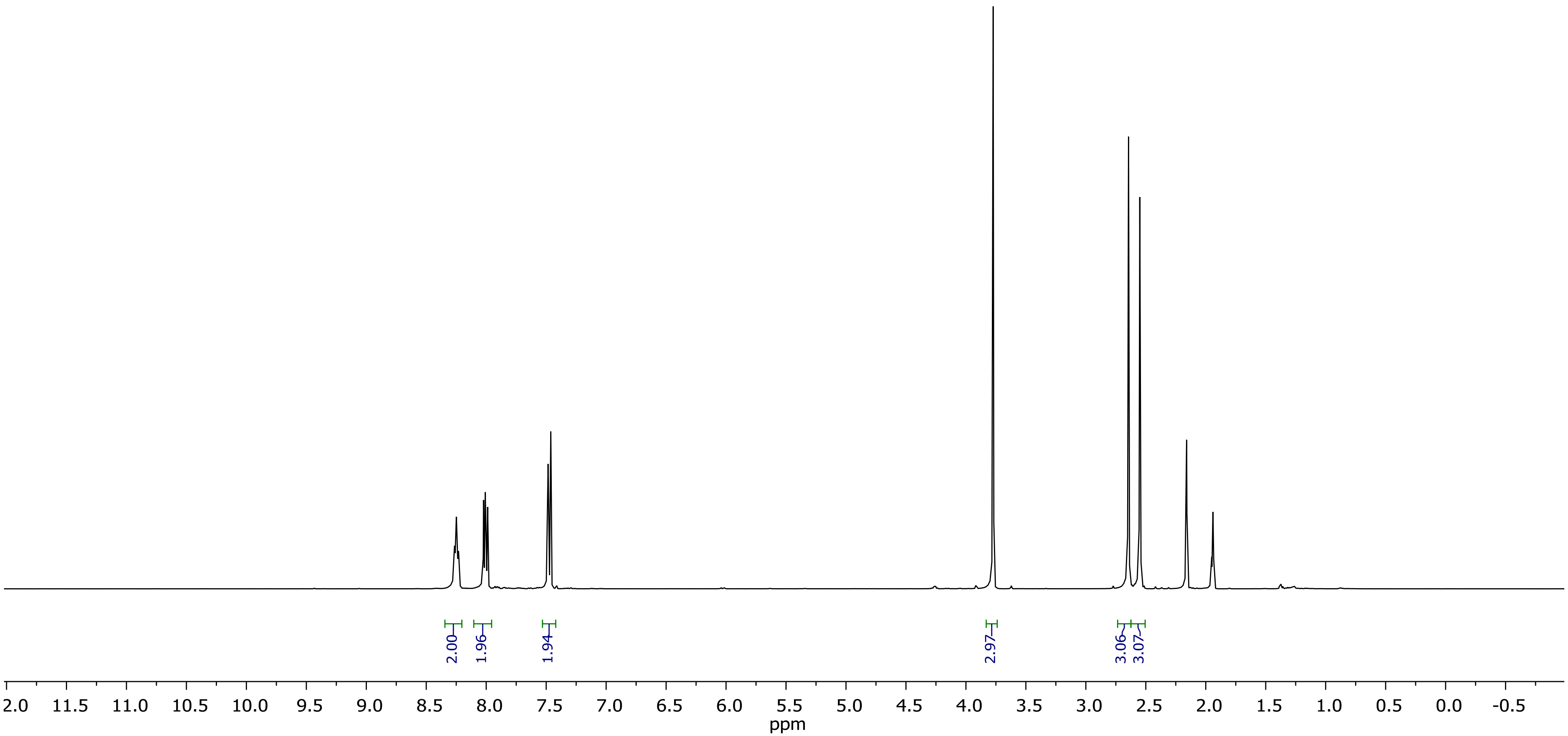

^{13}C NMR of methyl 2,4-dimethylbenzoate tetrafluorothianthrenium salt (12-TFT)

$CDCl_3$, 25 °C

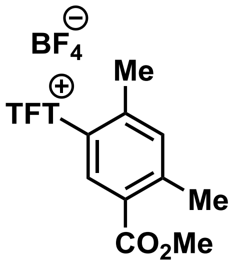

12-TFT

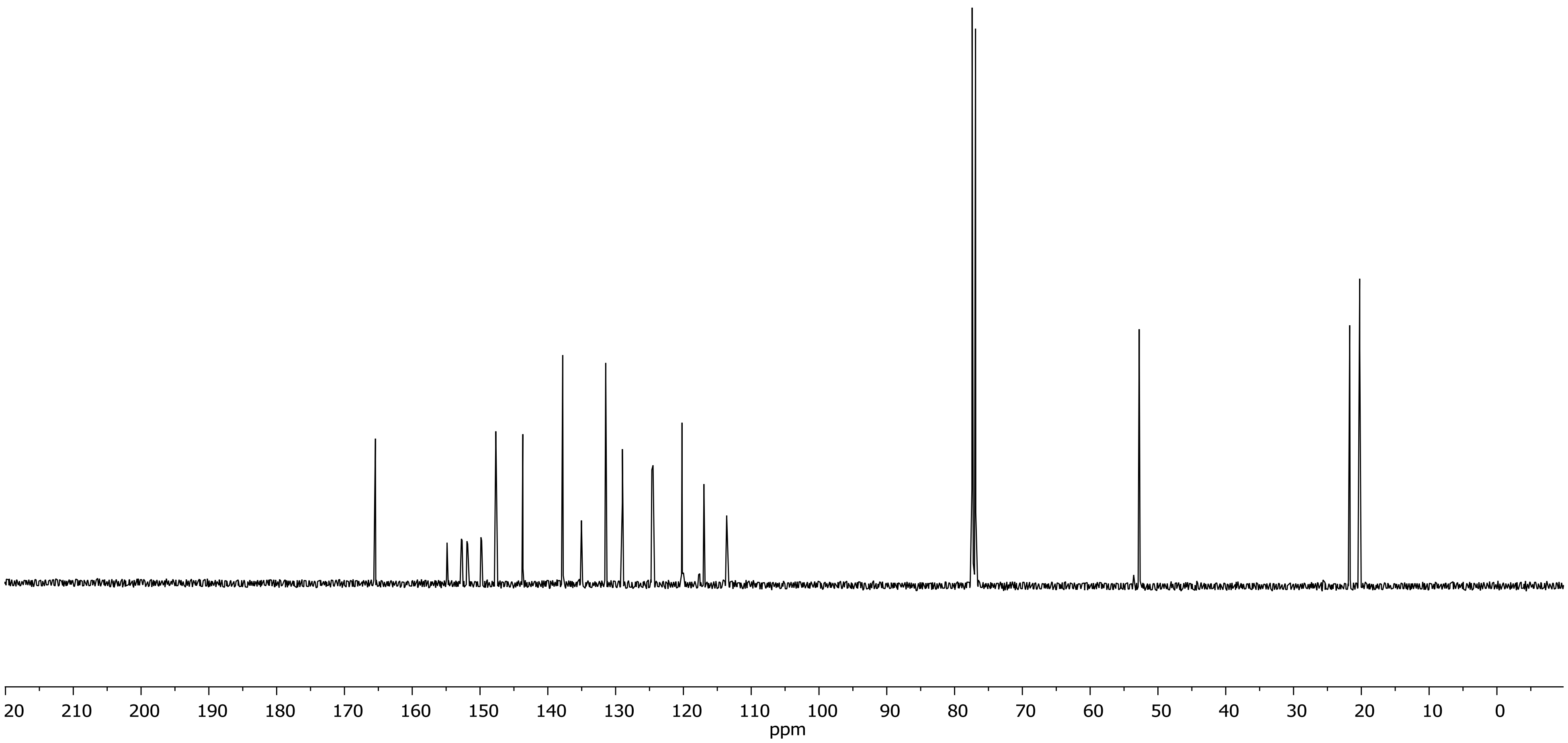

^{19}F NMR of methyl 2,4-dimethylbenzoate tetrafluorothianthrenium salt (12-TFT)

CD_3CN, 25 °C

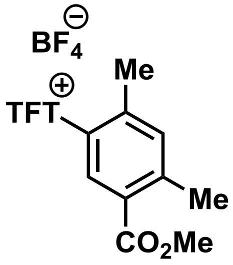

12-TFT

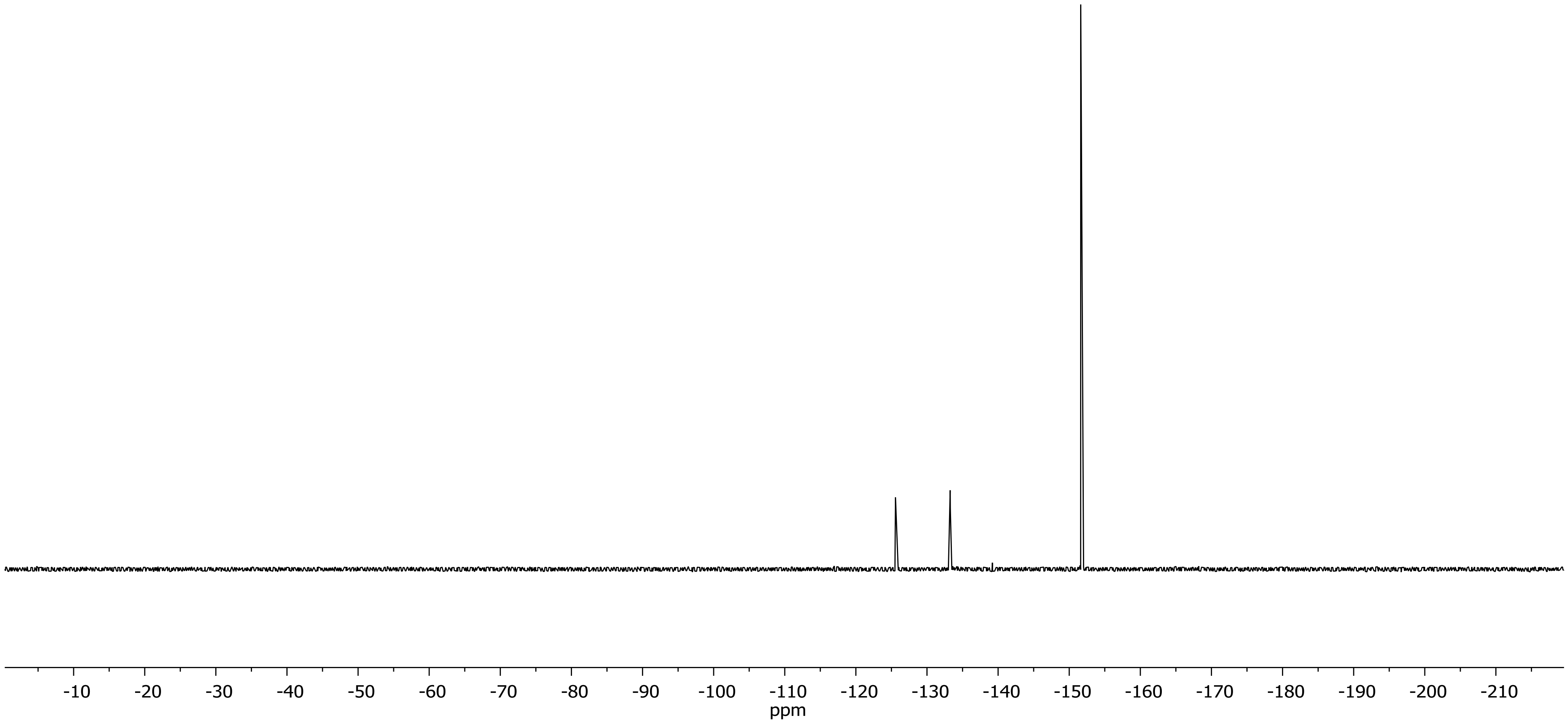

^{1}H NMR of methyl 5-hydroxy-2,4-dimethylbenzoate (12)

$CDCl_3$, 25 °C

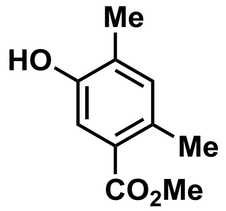

12

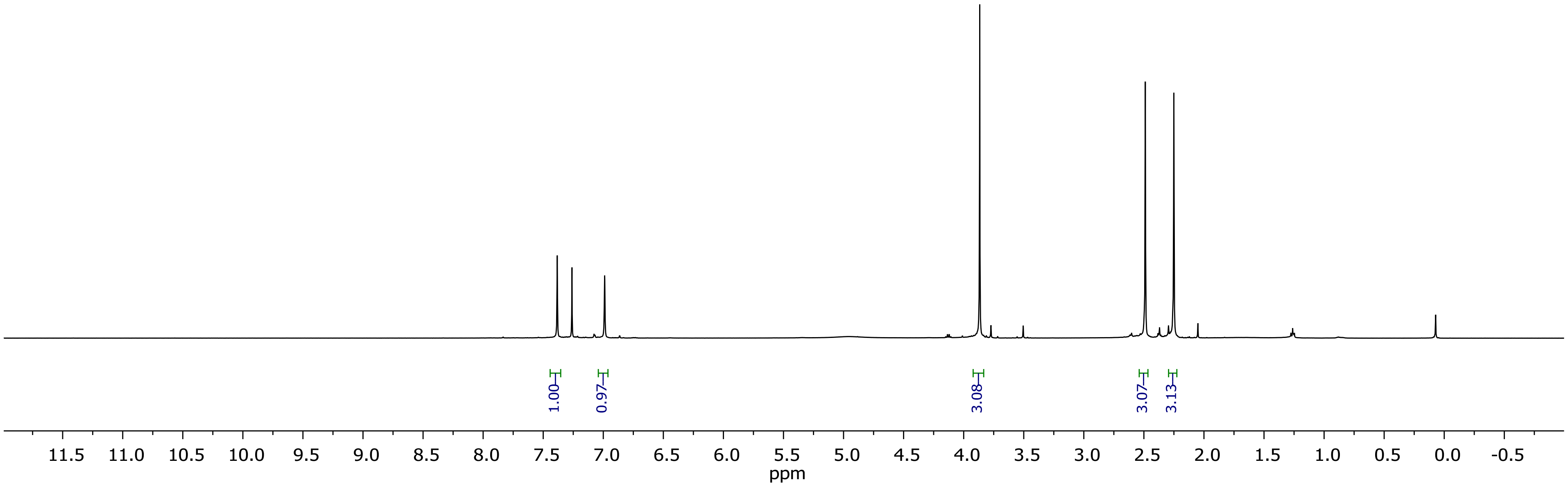

^{13}C NMR of methyl 5-hydroxy-2,4-dimethylbenzoate (12)

CD_3CN, 25 °C

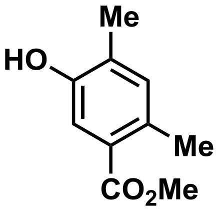

12

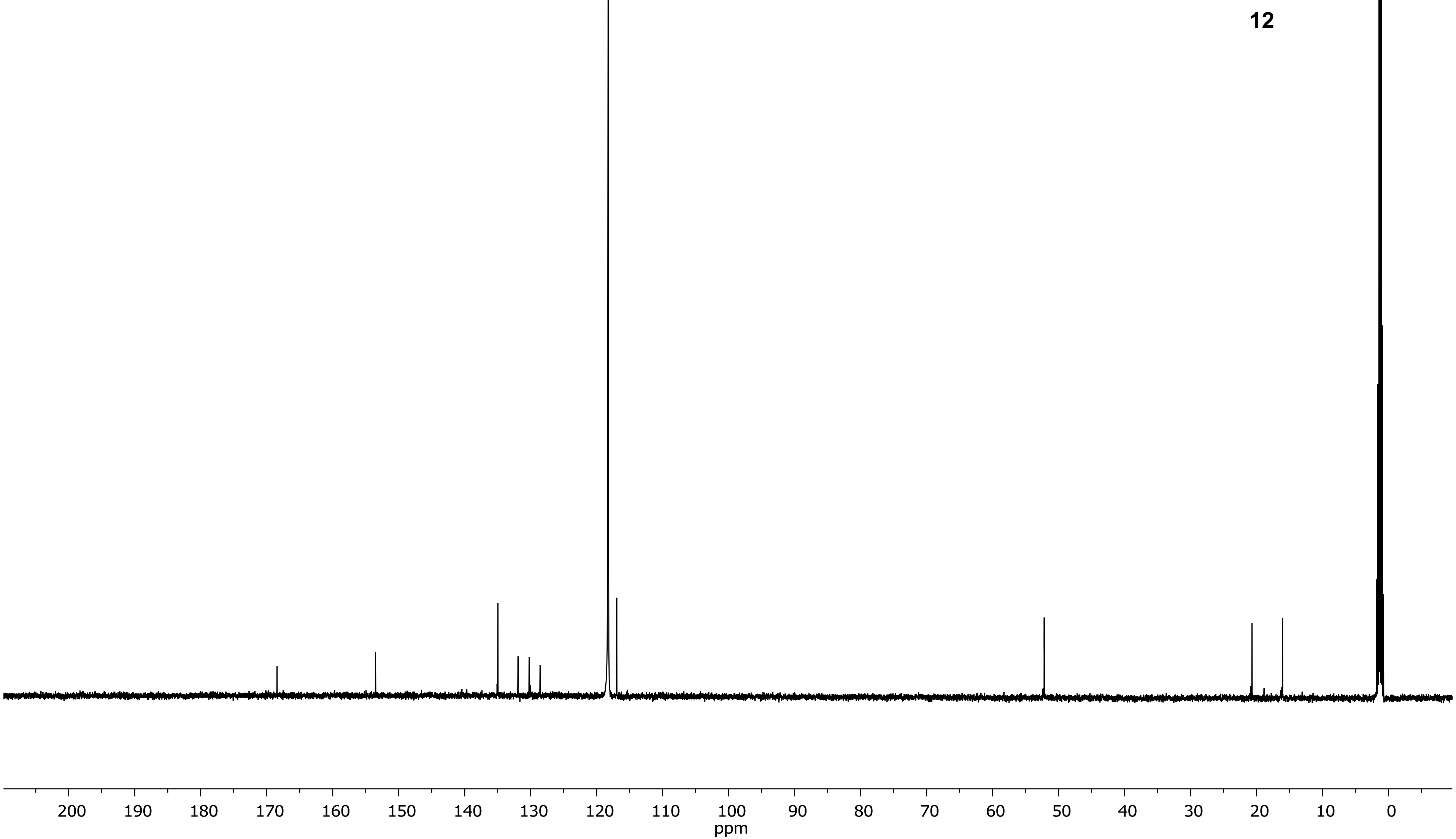

^{1}H NMR of 2-methoxy-5-methylbenzonitrile thianthrenium salt (13-TT)

CD_3CN, 25 °C

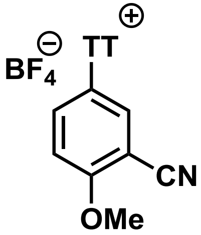

13-TT

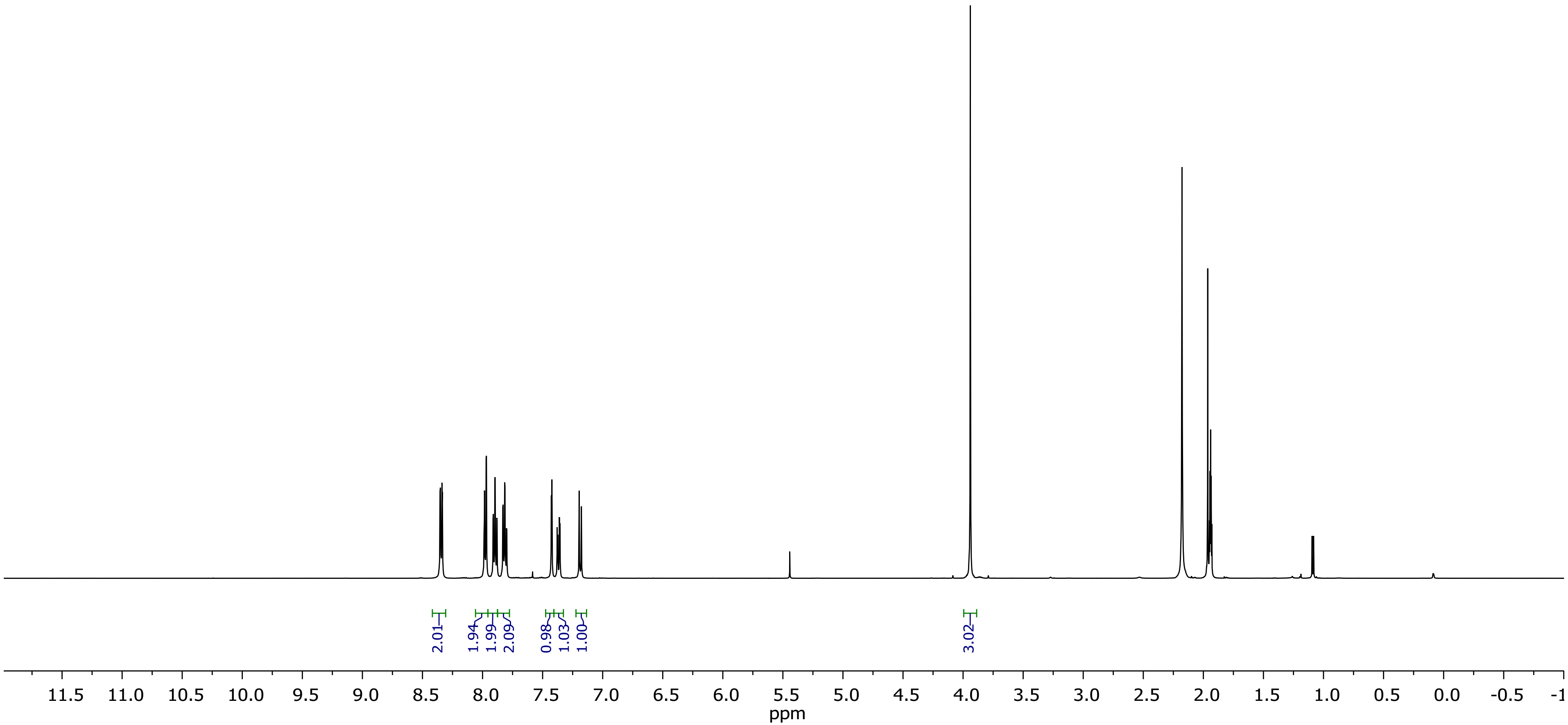

^{13}C NMR of 2-methoxy-5-methylbenzonitrile thianthrenium salt (13-TT)

CD_3CN, 25 °C

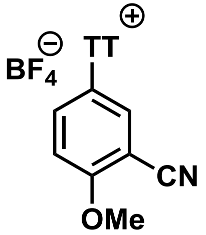

13-TT

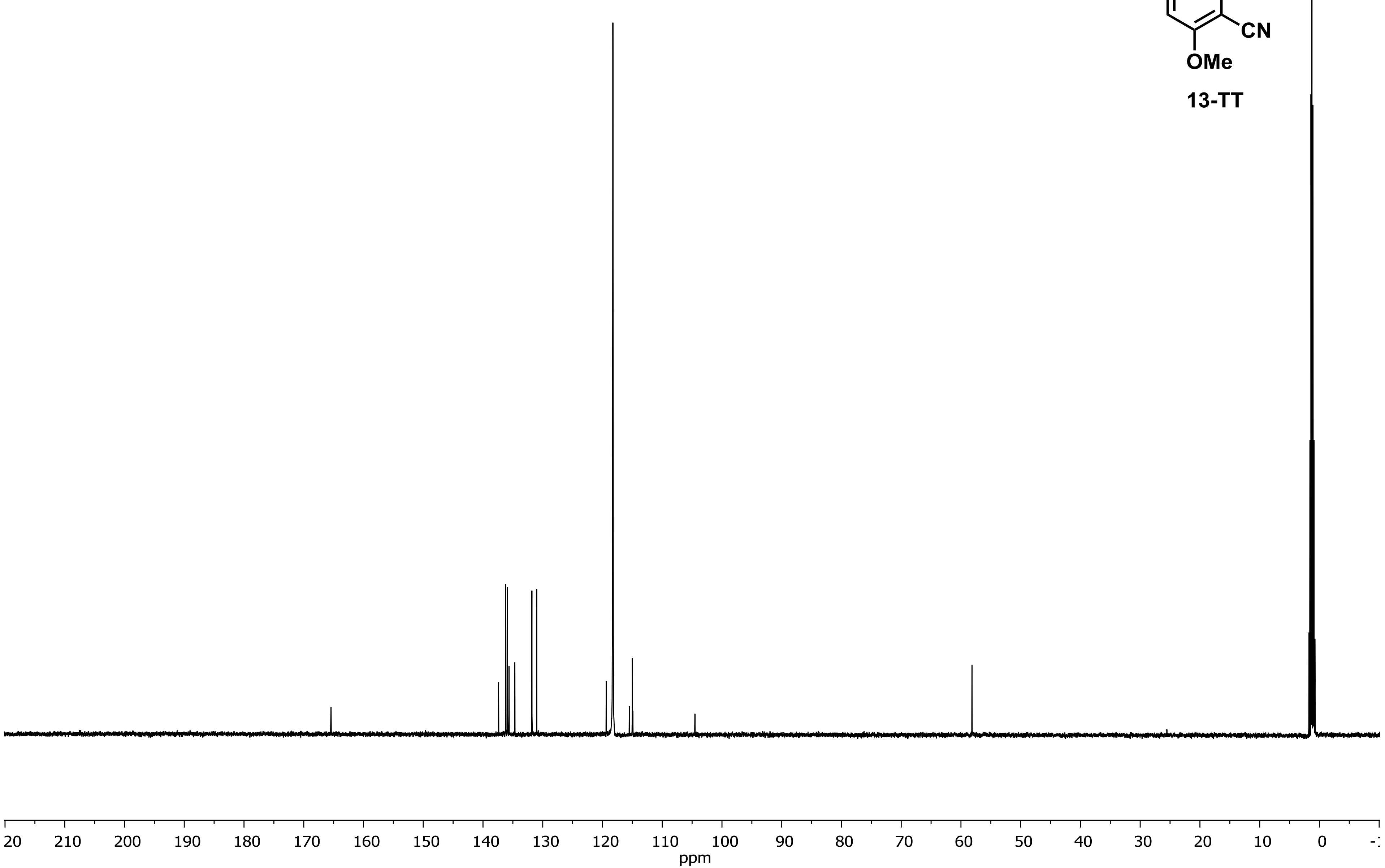

^{19}F NMR of 2-methoxy-5-methylbenzonitrile thianthrenium salt (13-TT)

CD_3CN, 25 °C

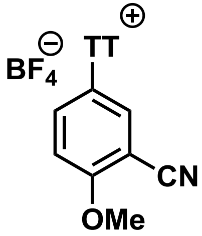

13-TT

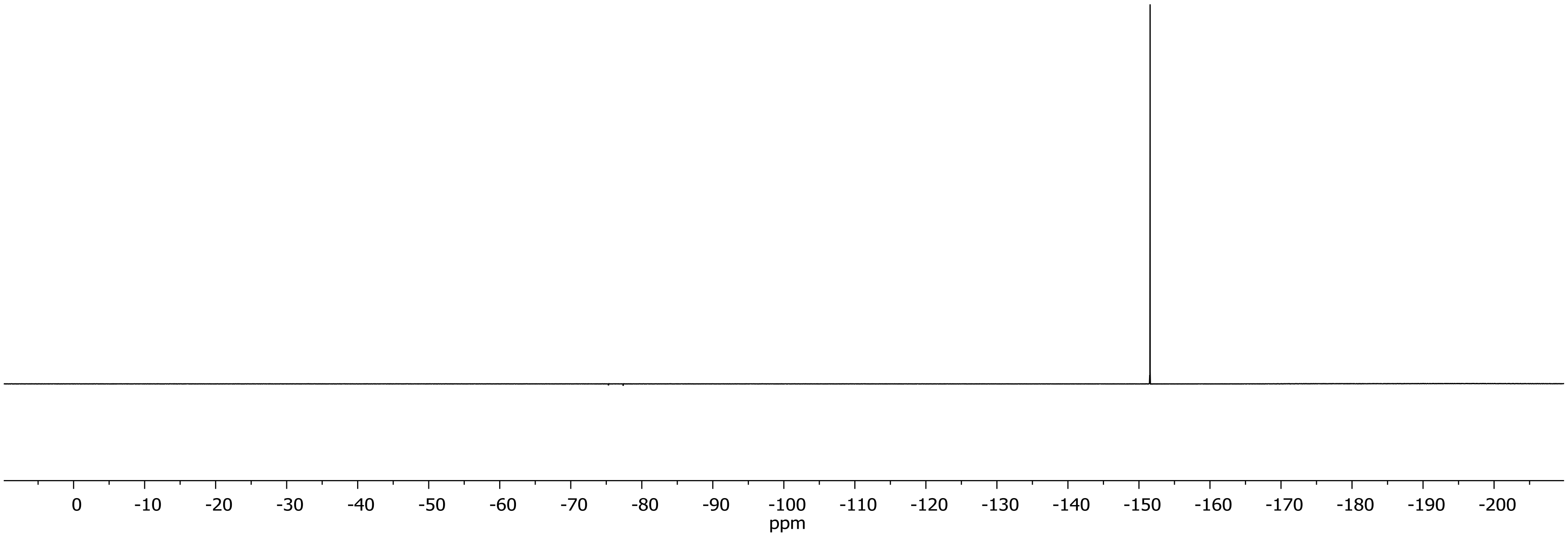

^{1}H NMR of 5-hydroxy-2-methoxybenzonitrile (13)

$CDCl_3$, 25 °C

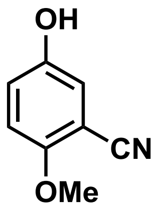

13

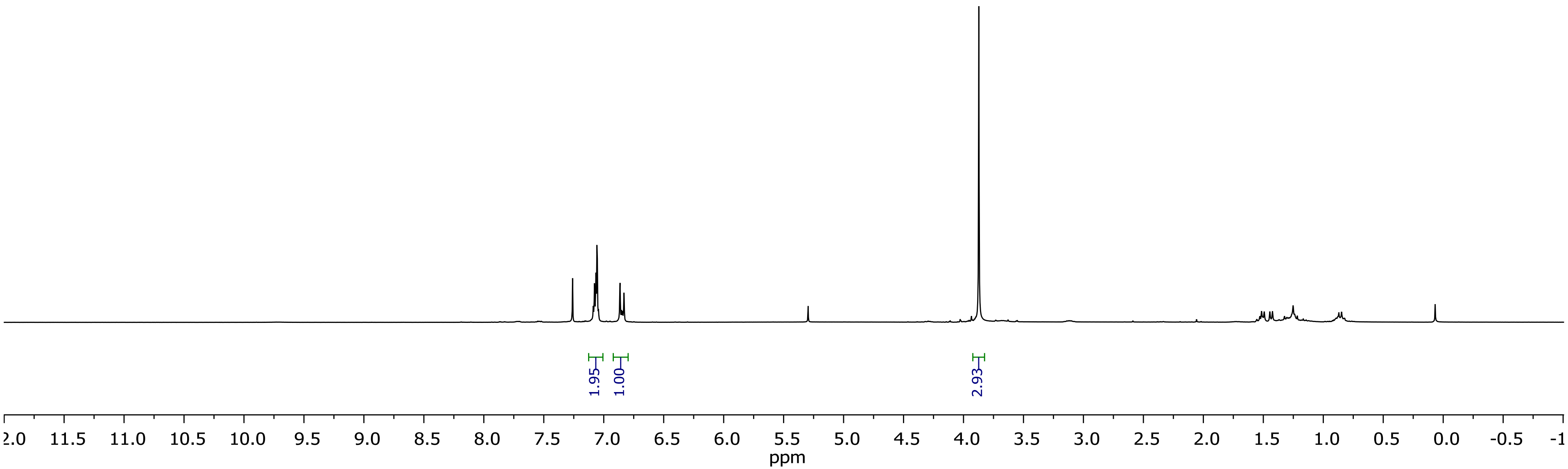

^{13}C NMR of 5-hydroxy-2-methoxybenzonitrile (13)

$CDCl_3$, 25 °C

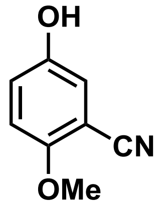

13

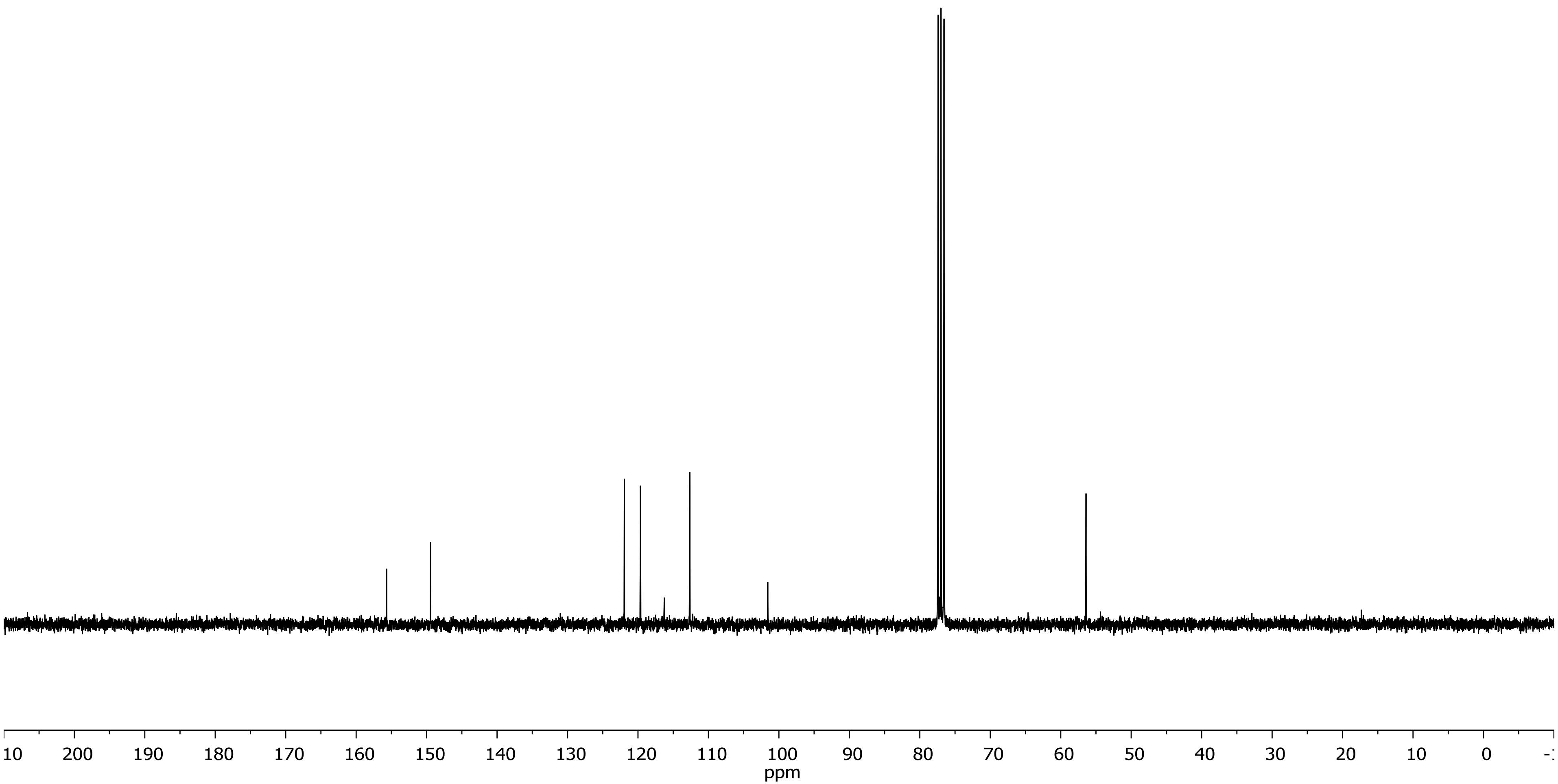

^{1}H NMR of 3-chloropropyl benzene tetrafluorothianthrenium salt (14-TFT)

CD_3CN, 25 °C

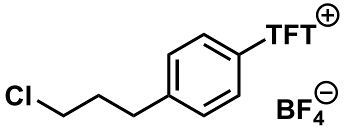

14-TFT

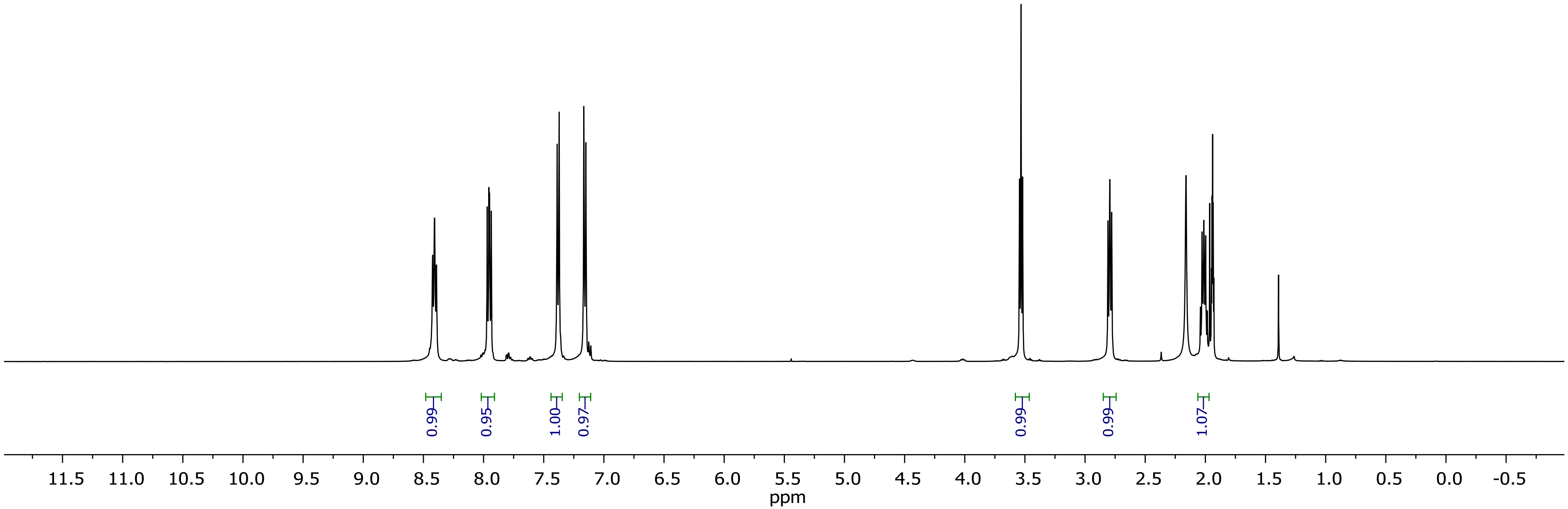

^{13}C NMR of 3-chloropropyl benzene tetrafluorothianthrenium salt (14-TFT)

CD_3CN, 25 °C

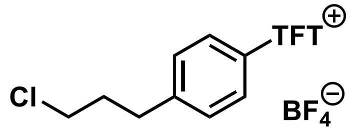

14-TFT

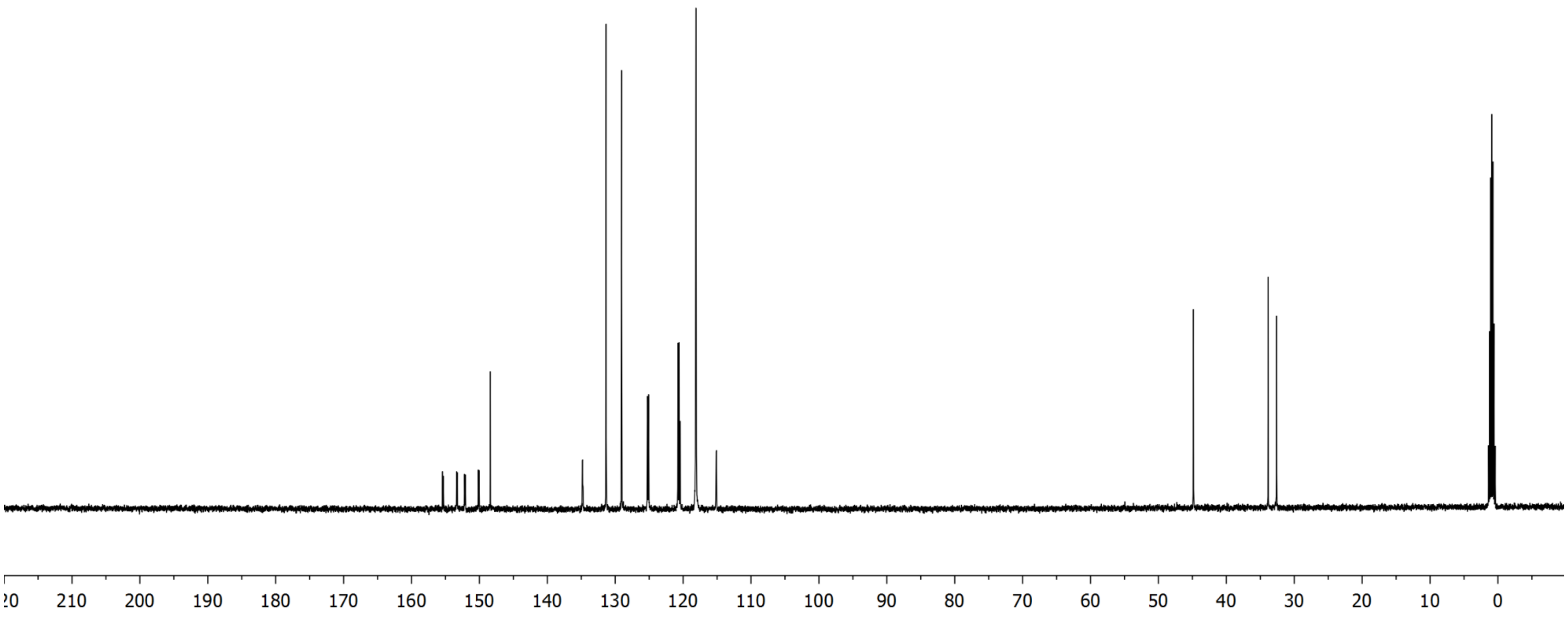

^{19}F NMR of 3-chloropropyl benzene tetrafluorothianthrenium salt (14-TFT)

CD_3CN, 25 °C

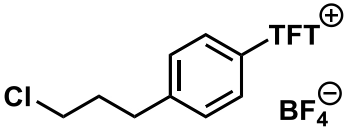

14-TFT

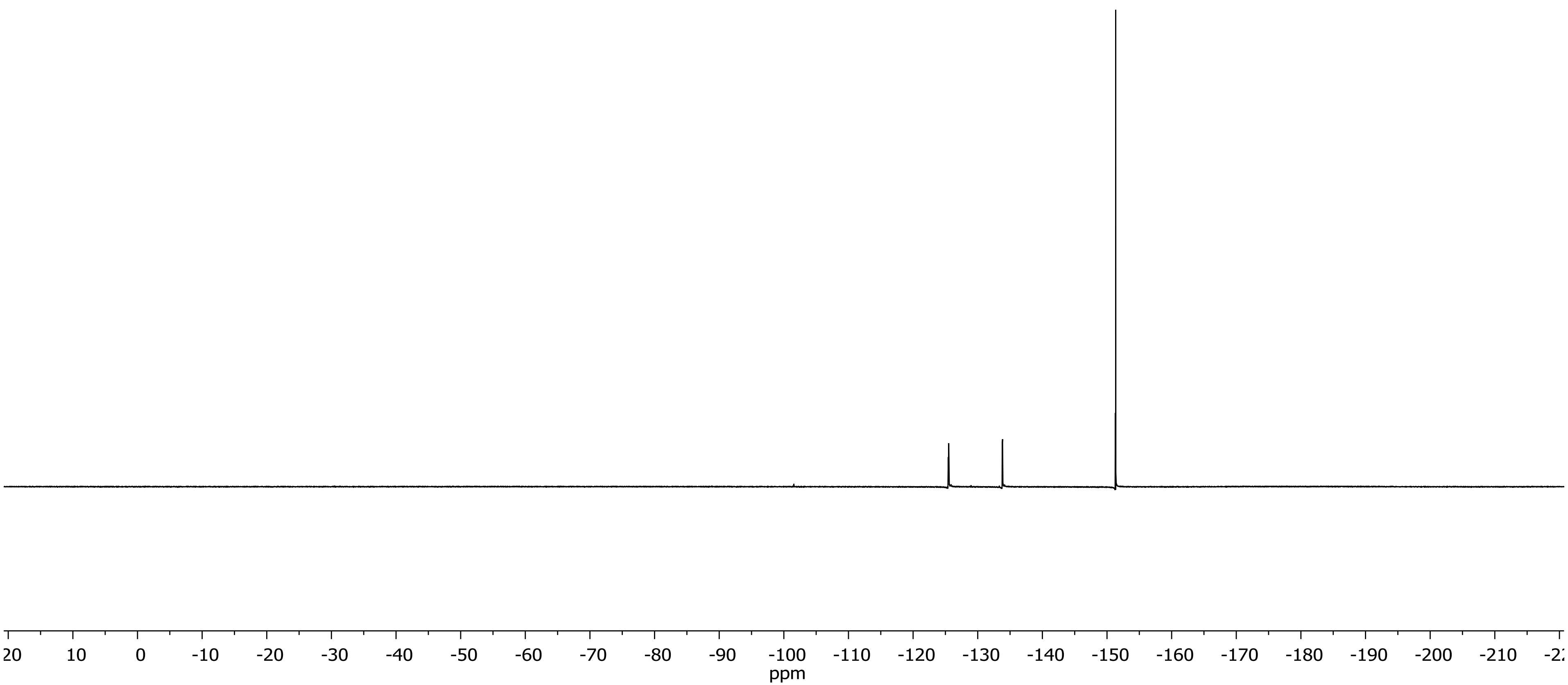

^{1}H NMR of 4-(3-chloropropyl)phenol (14)

CD_3CN, 25 °C

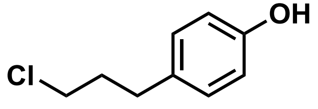

14

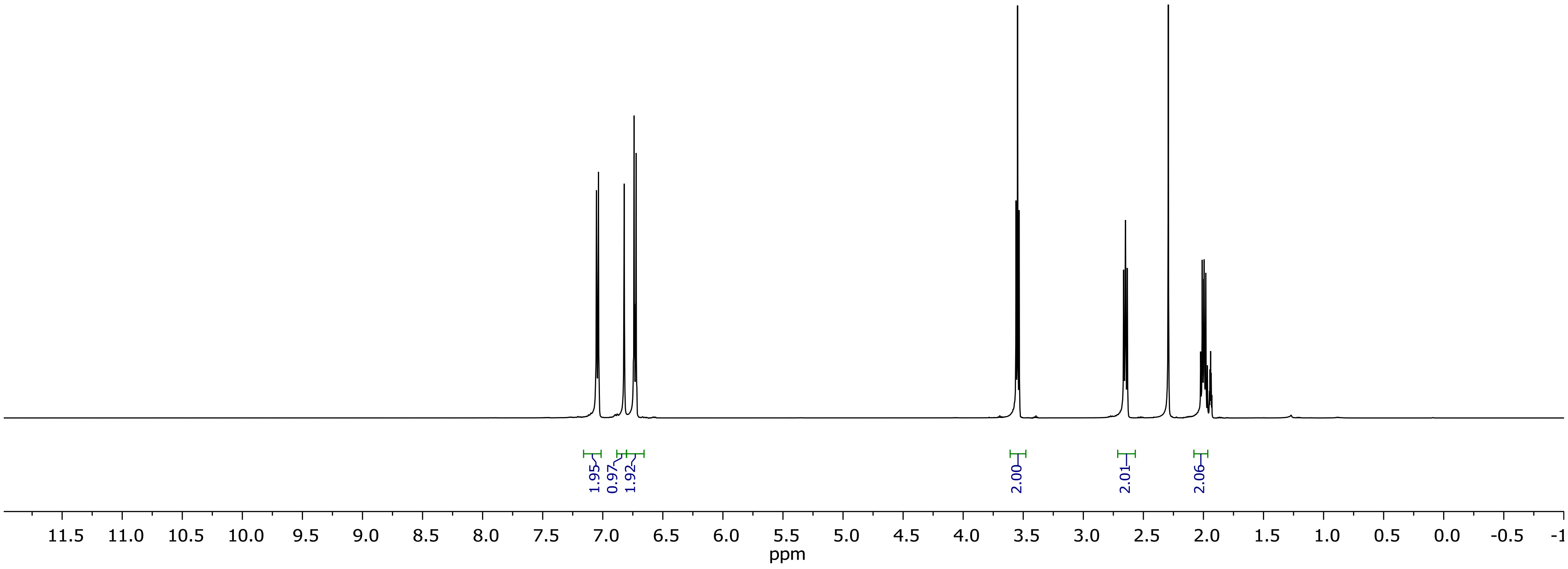

^{13}C NMR of 4-(3-chloropropyl)phenol (14)

CD_3CN, 25 °C

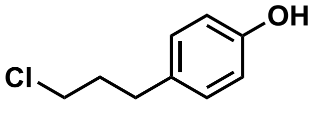

14

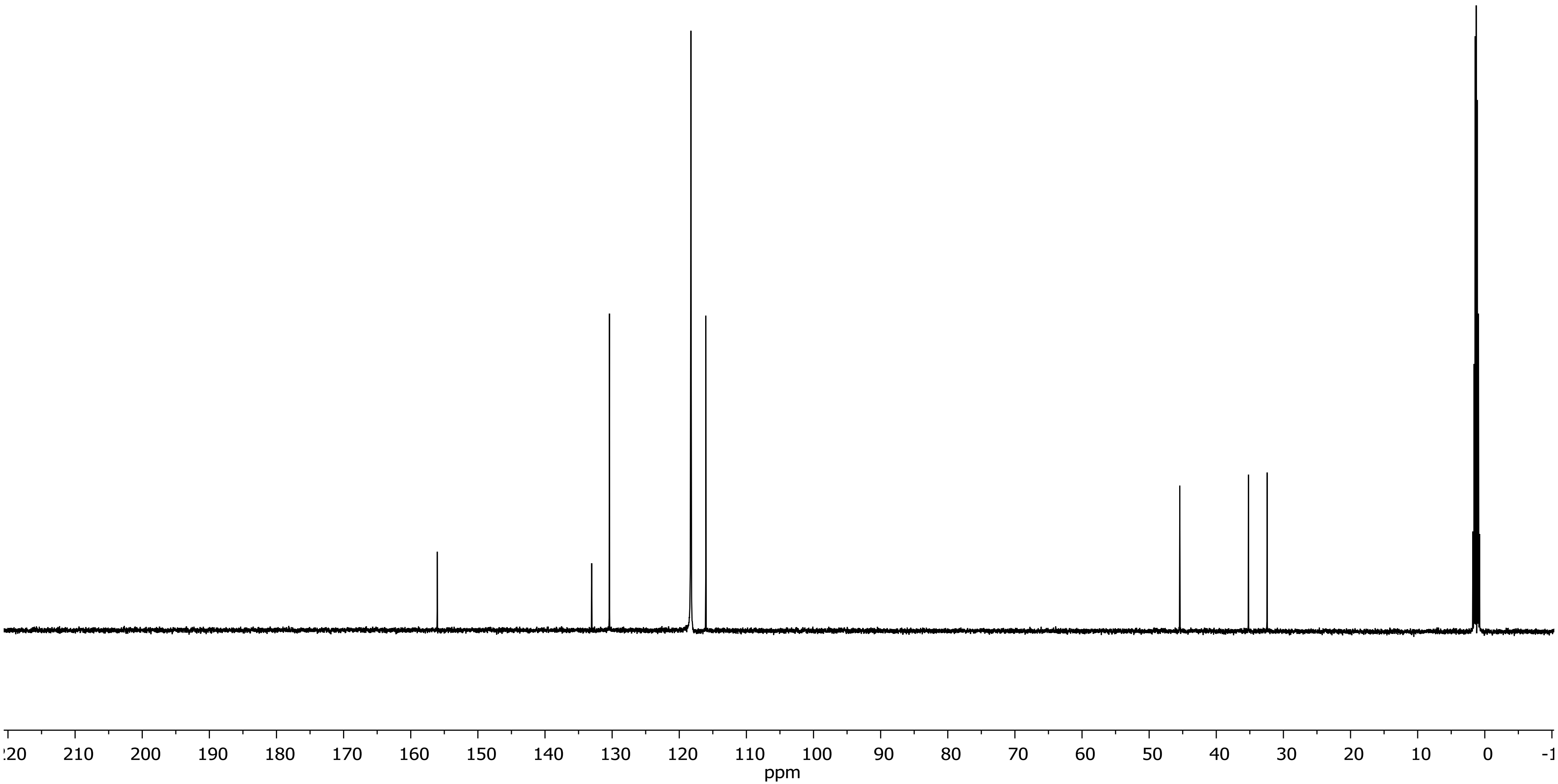

^{1}H NMR of 3-(3-hydroxy-1-phenylpropyl)phenol (15)

$CDCl_3$, 25 °C

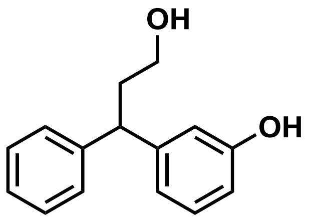

15

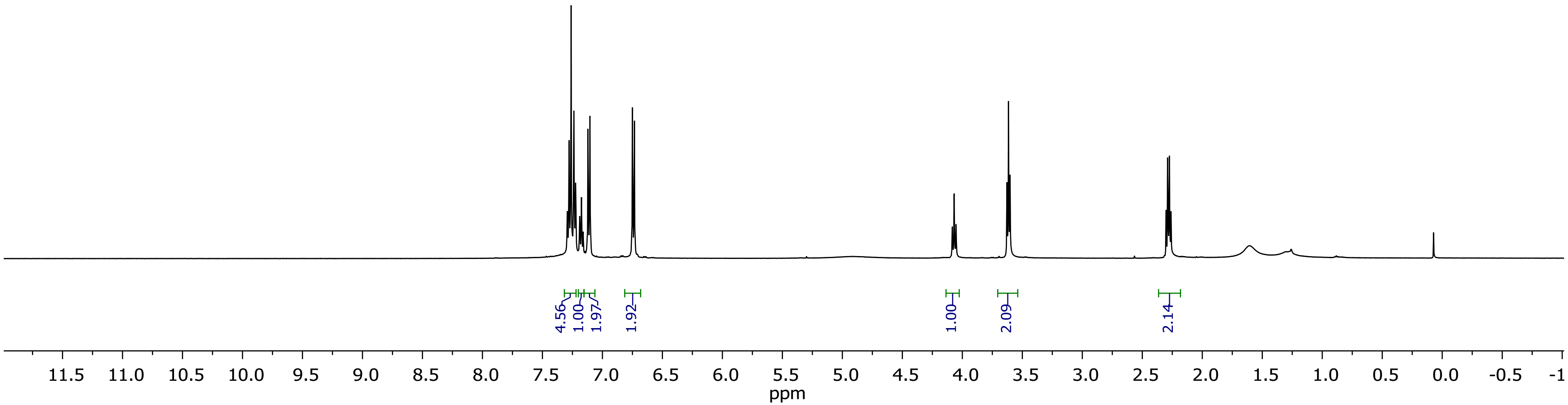

^{13}C NMR of 3-(3-hydroxy-1-phenylpropyl)phenol (15)

$CDCl_3$, 25 °C

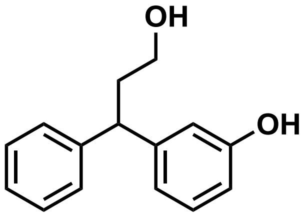

15

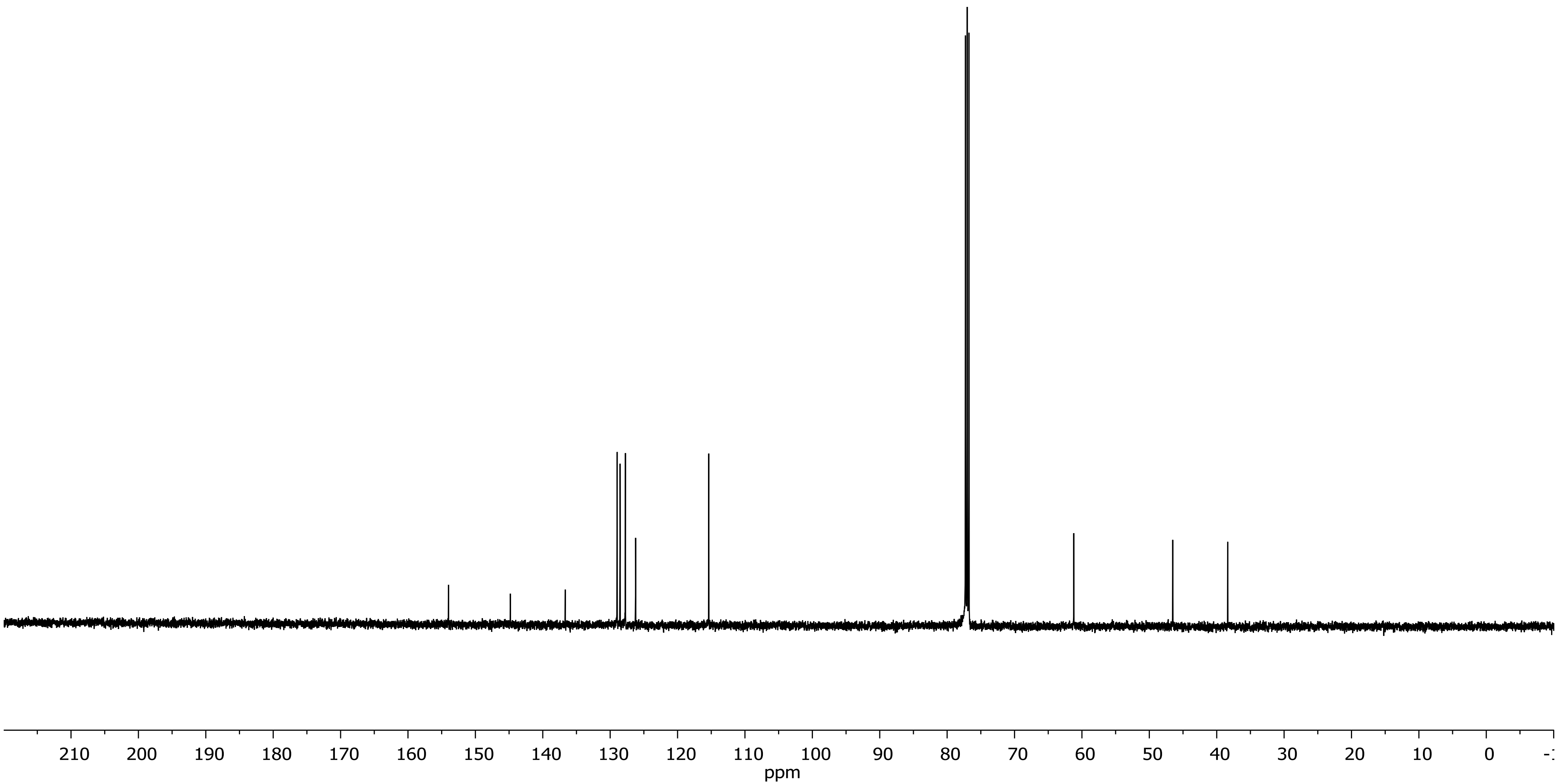

^{1}H NMR of hydroxy salicin pentaacetate (16)

$CDCl_3$, 25 °C

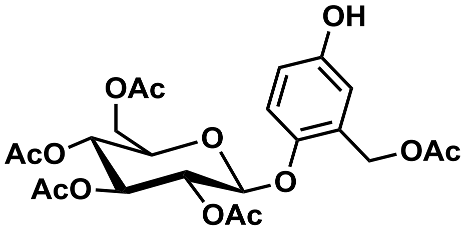

16

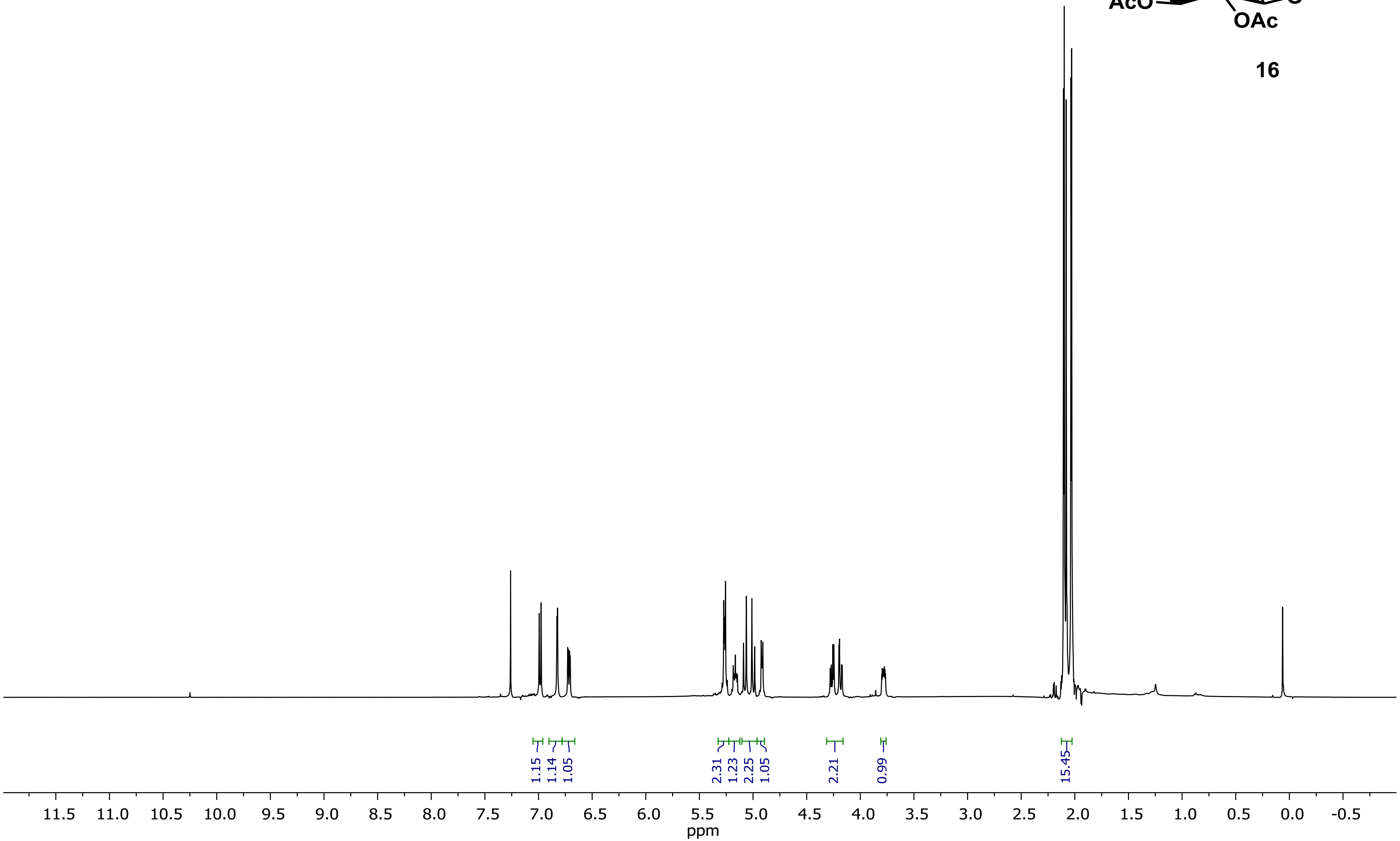

^{13}H NMR of hydroxy salicin pentaacetate (16)

$CDCl_3$, 25 °C

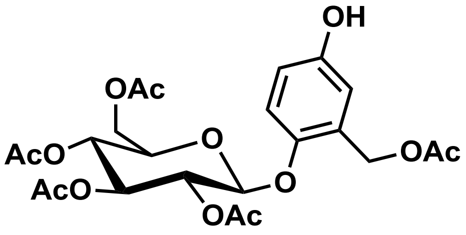

16

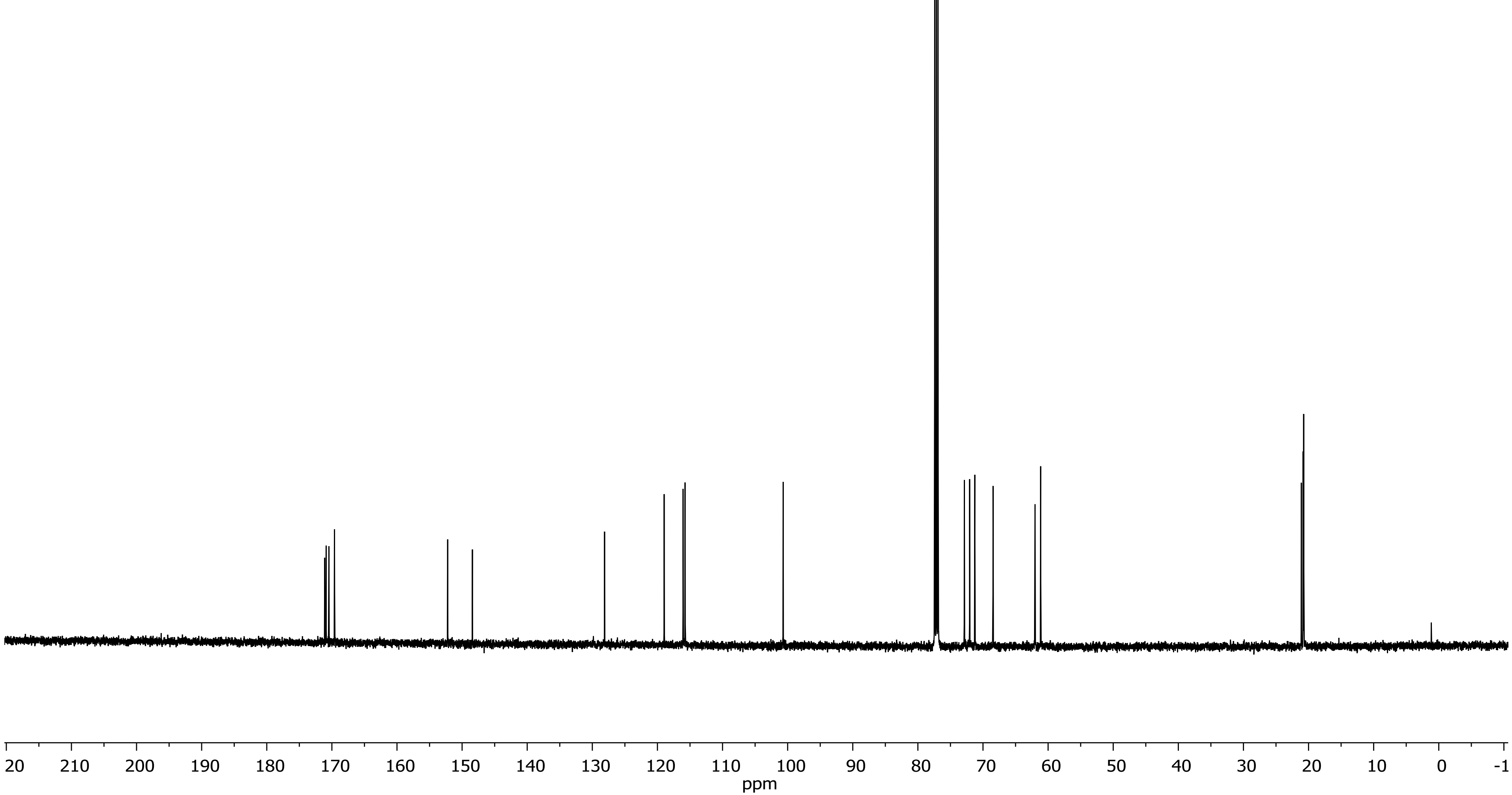

^{1}H NMR of methyl 1-phenylcyclopropane-1-carboxylate tetrafluorothianthrenium salt (17-TFT)

CD_3CN, 25 °C

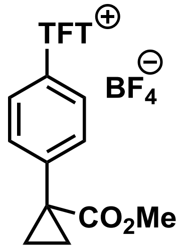

17-TFT

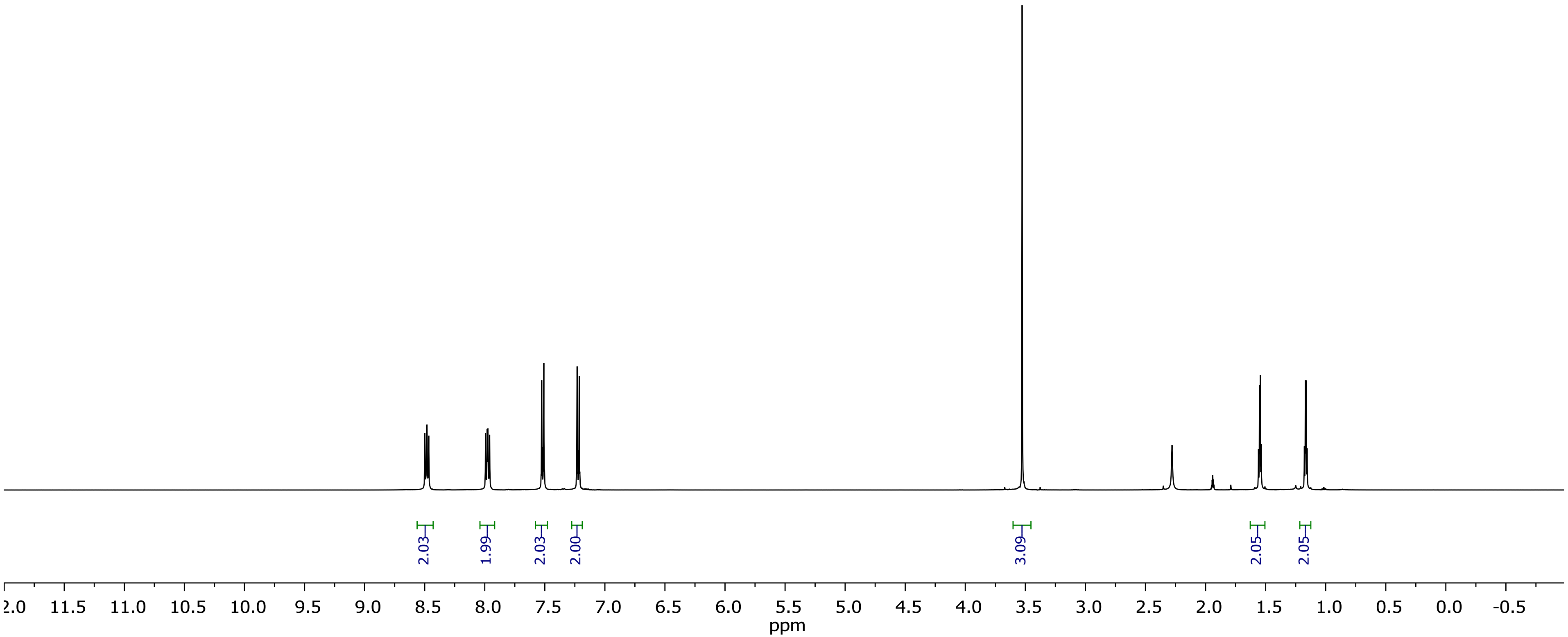

^{13}C NMR of methyl 1-phenylcyclopropane-1-carboxylate tetrafluorothianthrenium salt (17-TFT)

CD_3CN, 25 °C

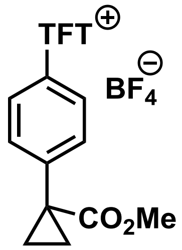

17-TFT

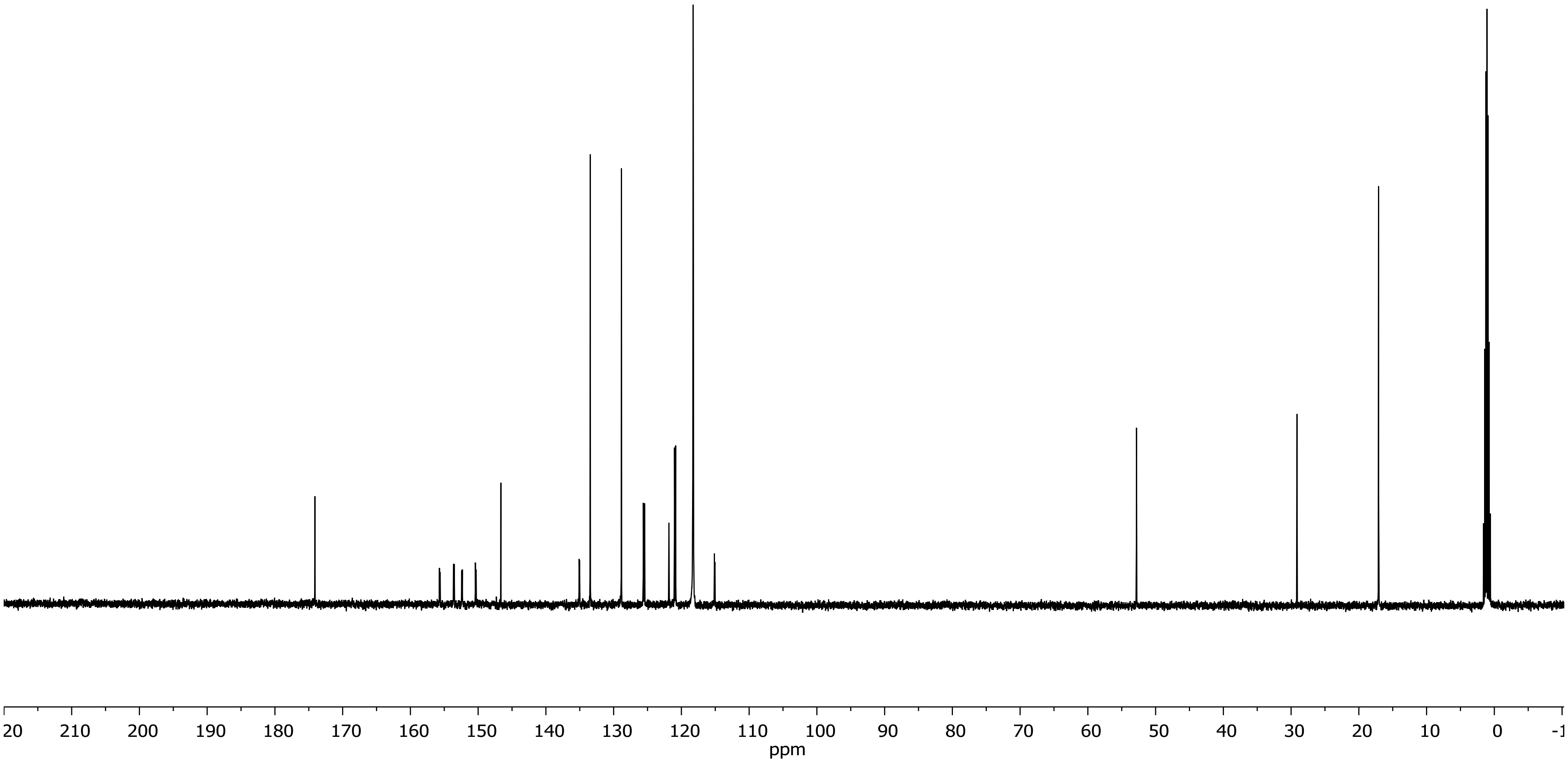

^{19}F NMR of methyl 1-phenylcyclopropane-1-carboxylate tetrafluorothianthrenium salt (17-TFT)

CD_3CN, 25 °C

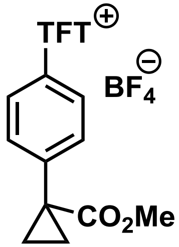

17-TFT

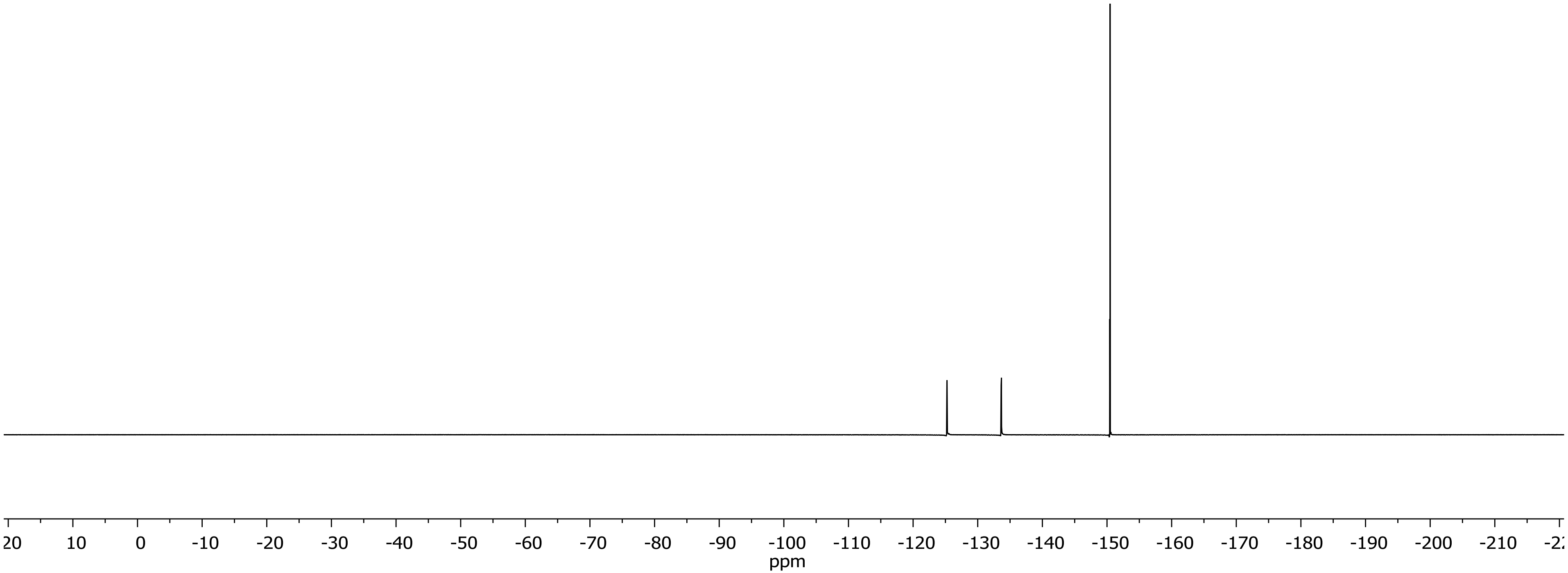

^{1}H NMR of methyl 1-(4-hydroxyphenyl)cyclopropane-1-carboxylate (17)

CD_3CN, 25 °C

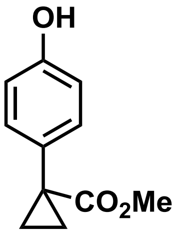

17

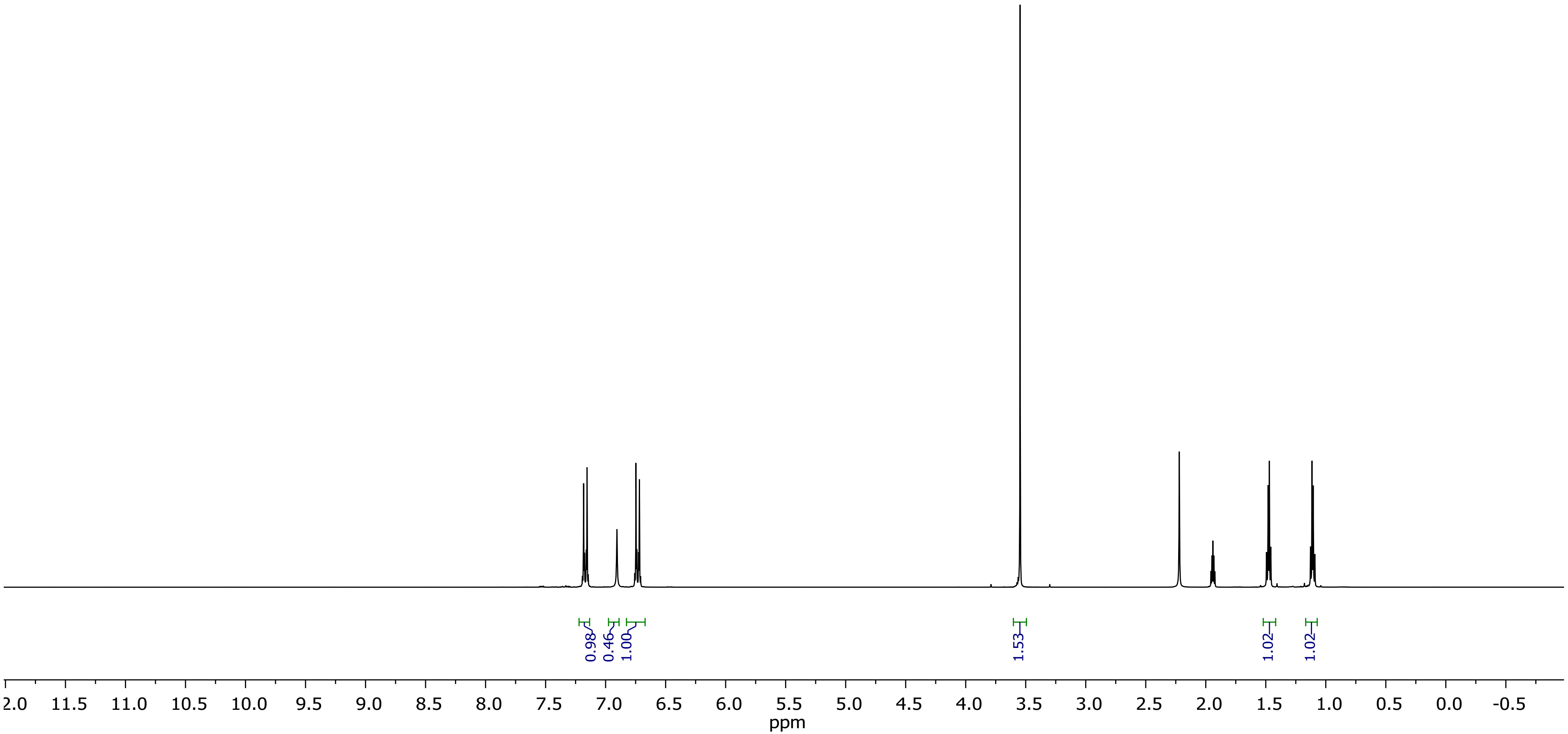

^{13}C NMR of methyl 1-(4-hydroxyphenyl)cyclopropane-1-carboxylate (17)

CD_3CN, 25 °C

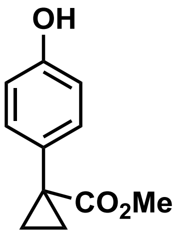

17

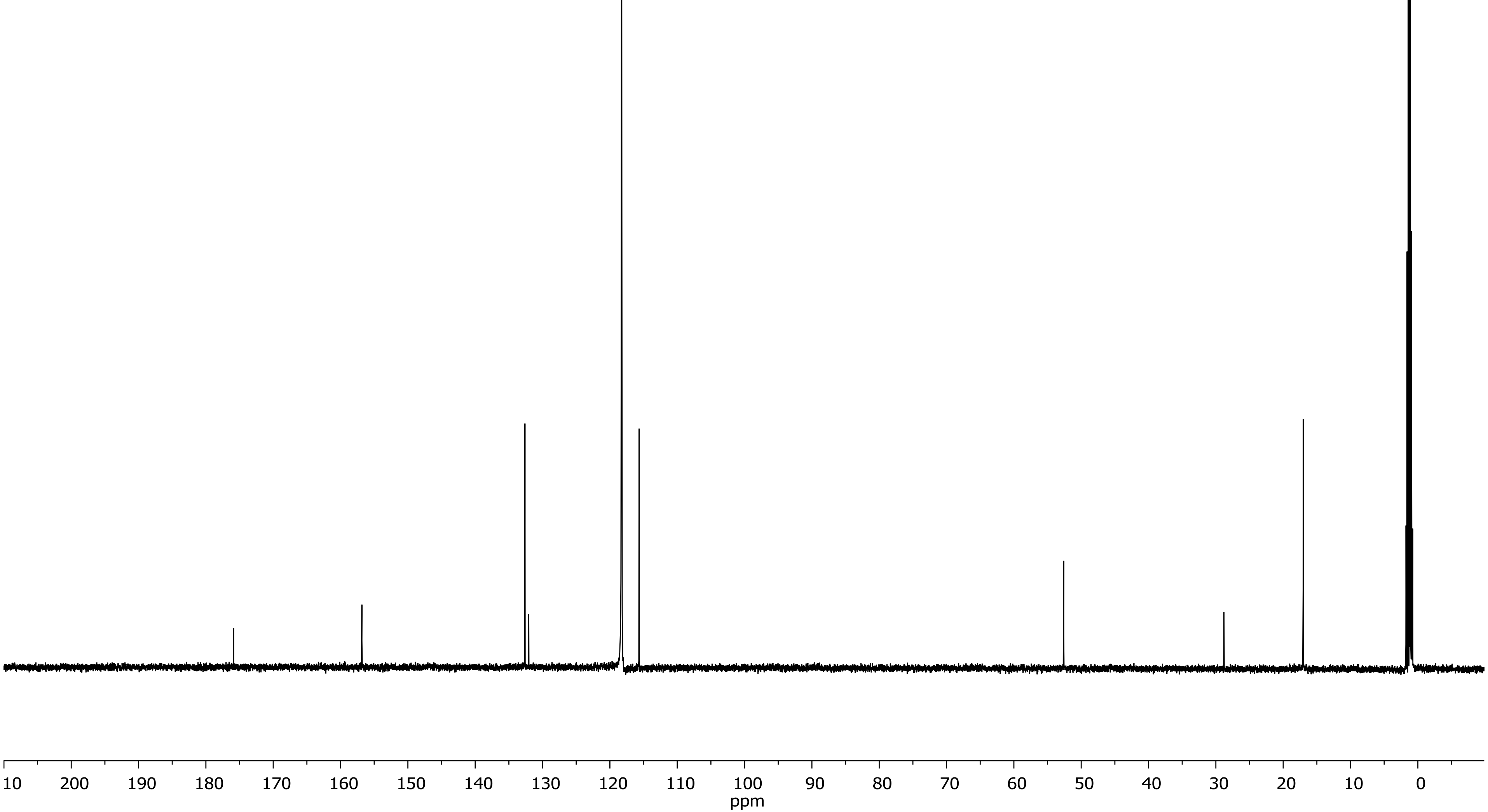

^{1}H NMR of 6-methyl-4-chromanone thianthrenium salt (18-TT)

$CDCl_3$, 25 °C

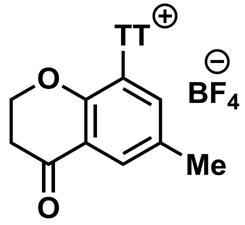

18-TT

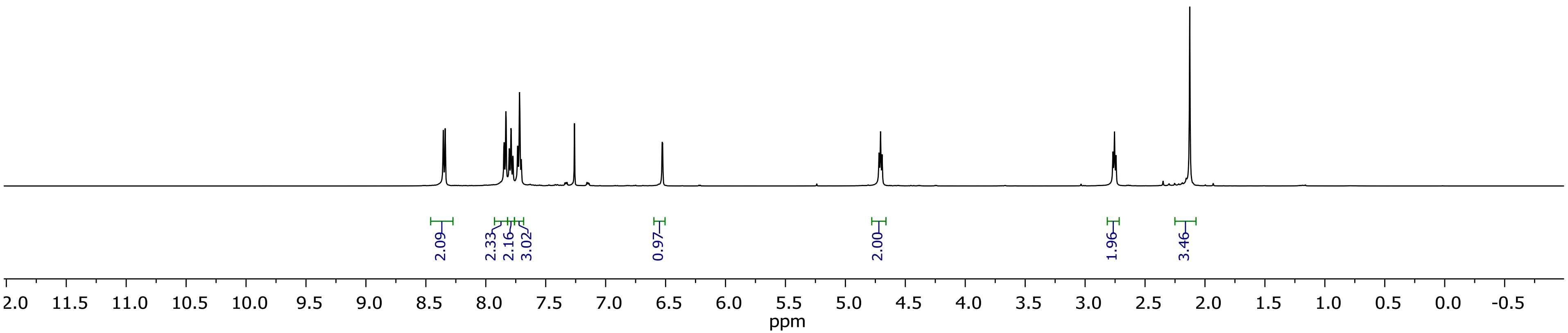

^{13}C NMR of 6-methyl-4-chromanone thianthrenium salt (18-TT)

$CDCl_3$, 25 °C

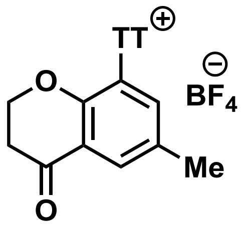

18-TT

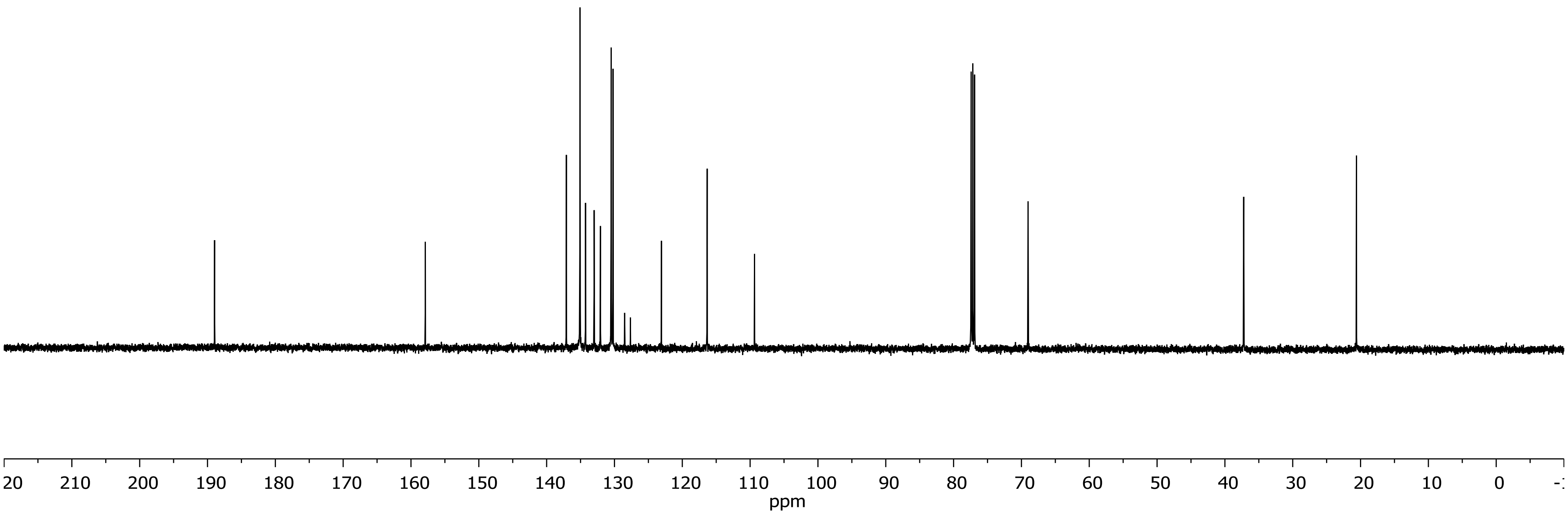

^{19}F NMR of 6-methyl-4-chromanone thianthrenium salt (18-TT)

$CDCl_3$, 25 °C

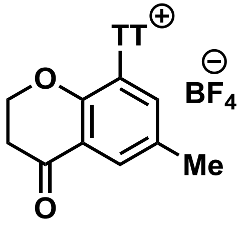

18-TT

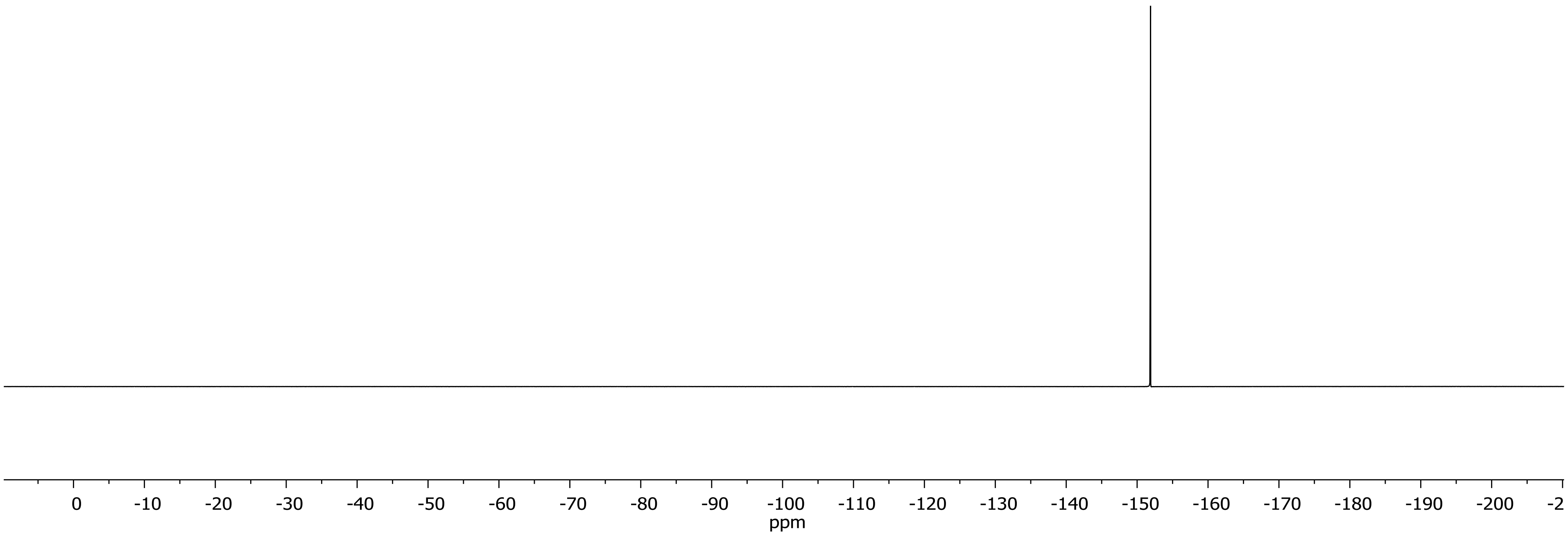

^{1}H NMR of 8-hydroxy-6-methylchroman-4-one (18)

CD_3CN, 25 °C

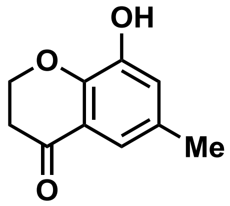

18

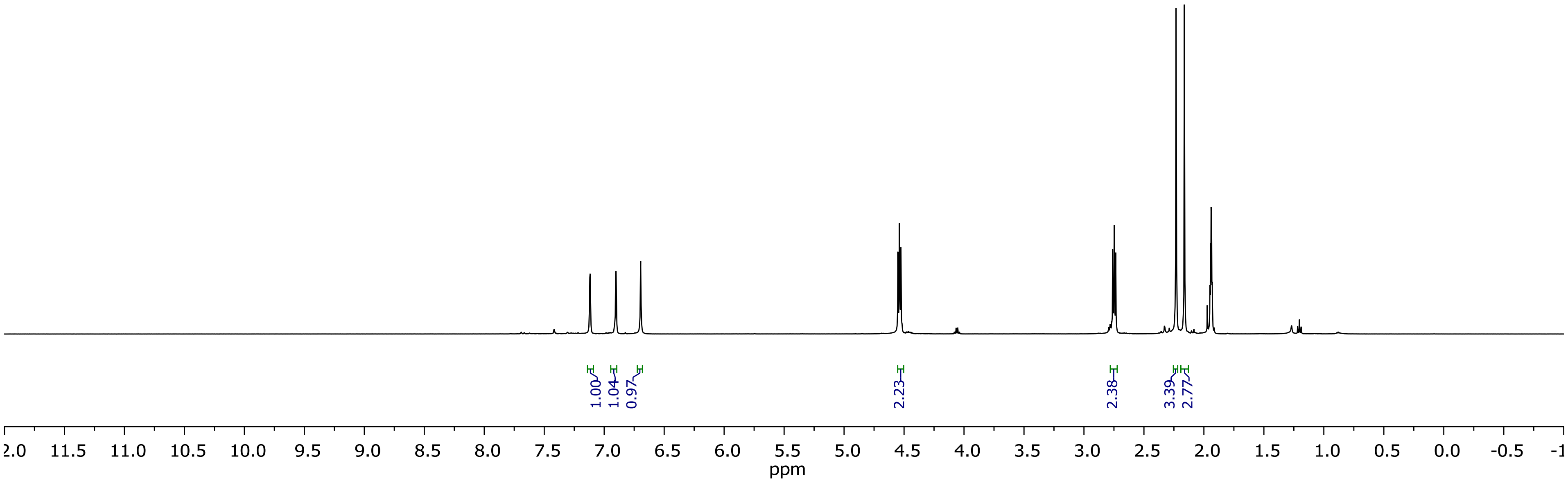

^{13}C NMR of 8-hydroxy-6-methylchroman-4-one (18)

CD_3CN, 25 °C

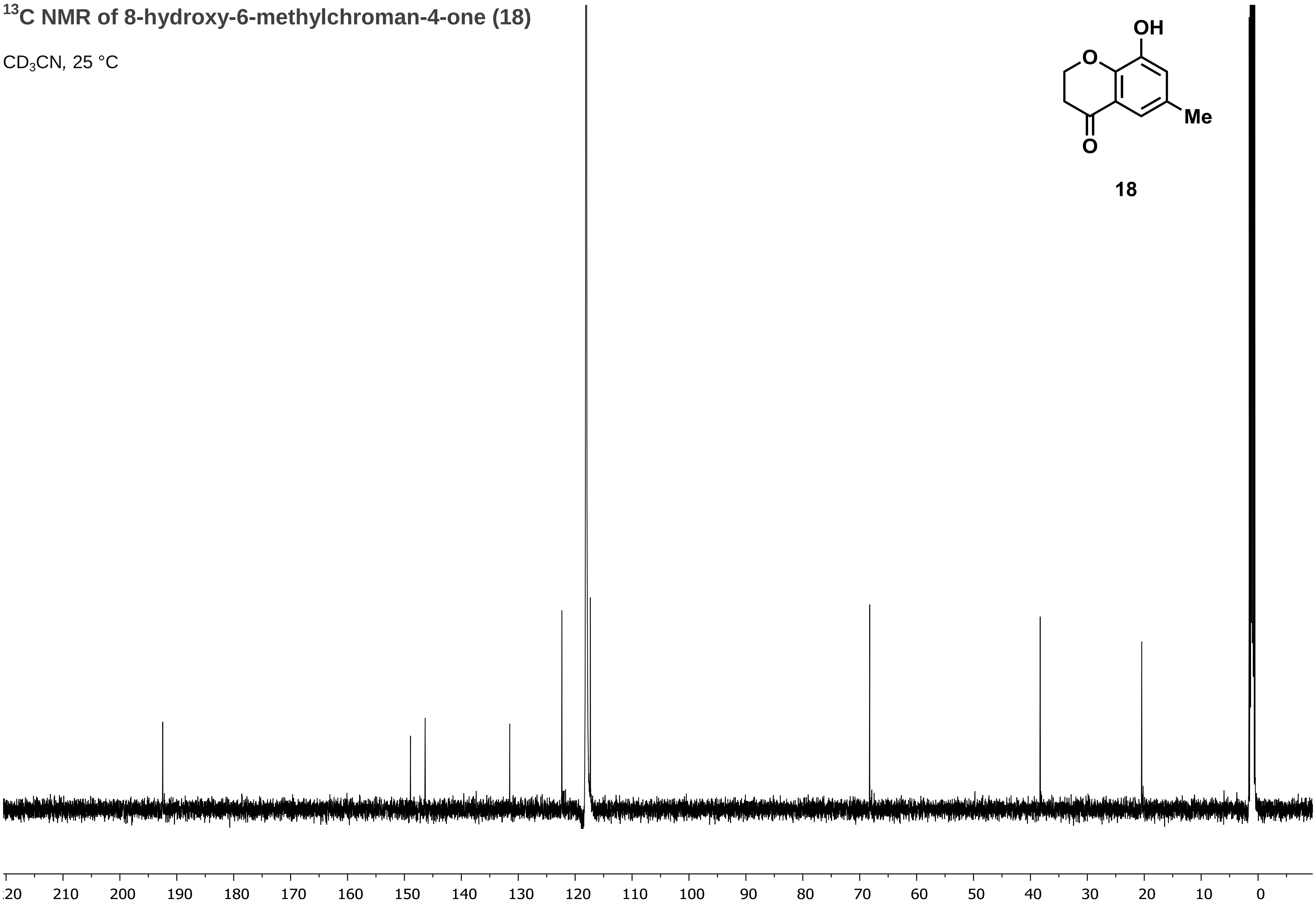

^{1}H NMR of benzyl benzoate tetrafluorothianthrenium salt (19-TFT)

CD_3CN, 25 °C

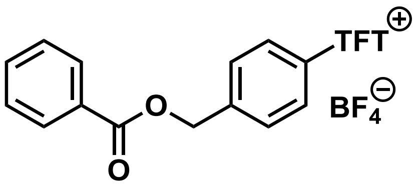

19-TFT

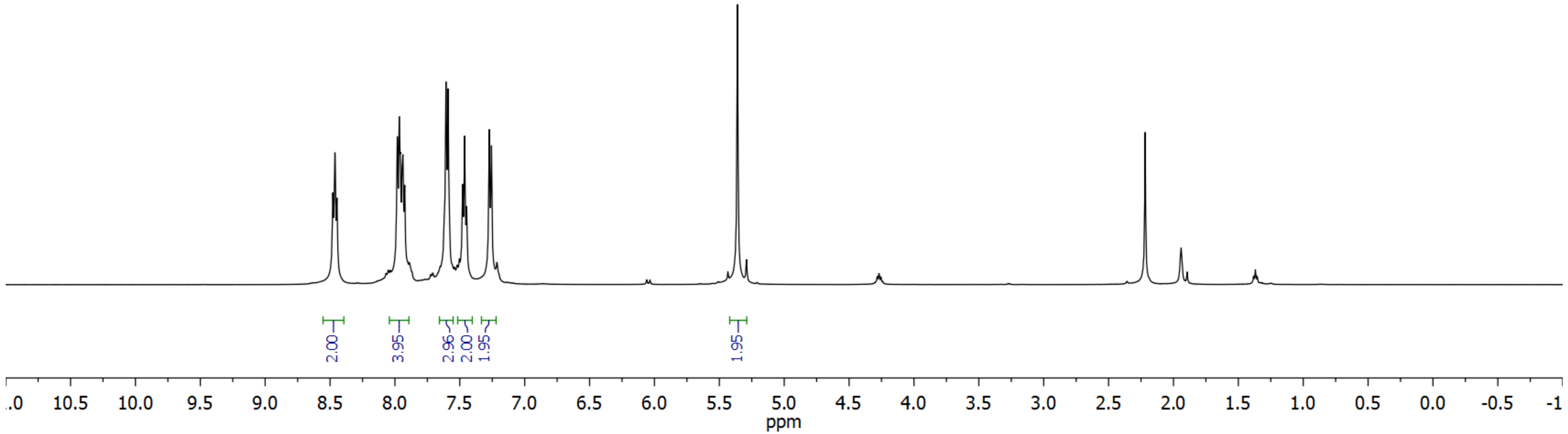

^{13}C NMR of benzyl benzoate tetrafluorothianthrenium salt (19-TFT)

CD_3CN, 25 °C

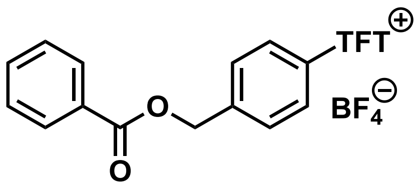

19-TFT

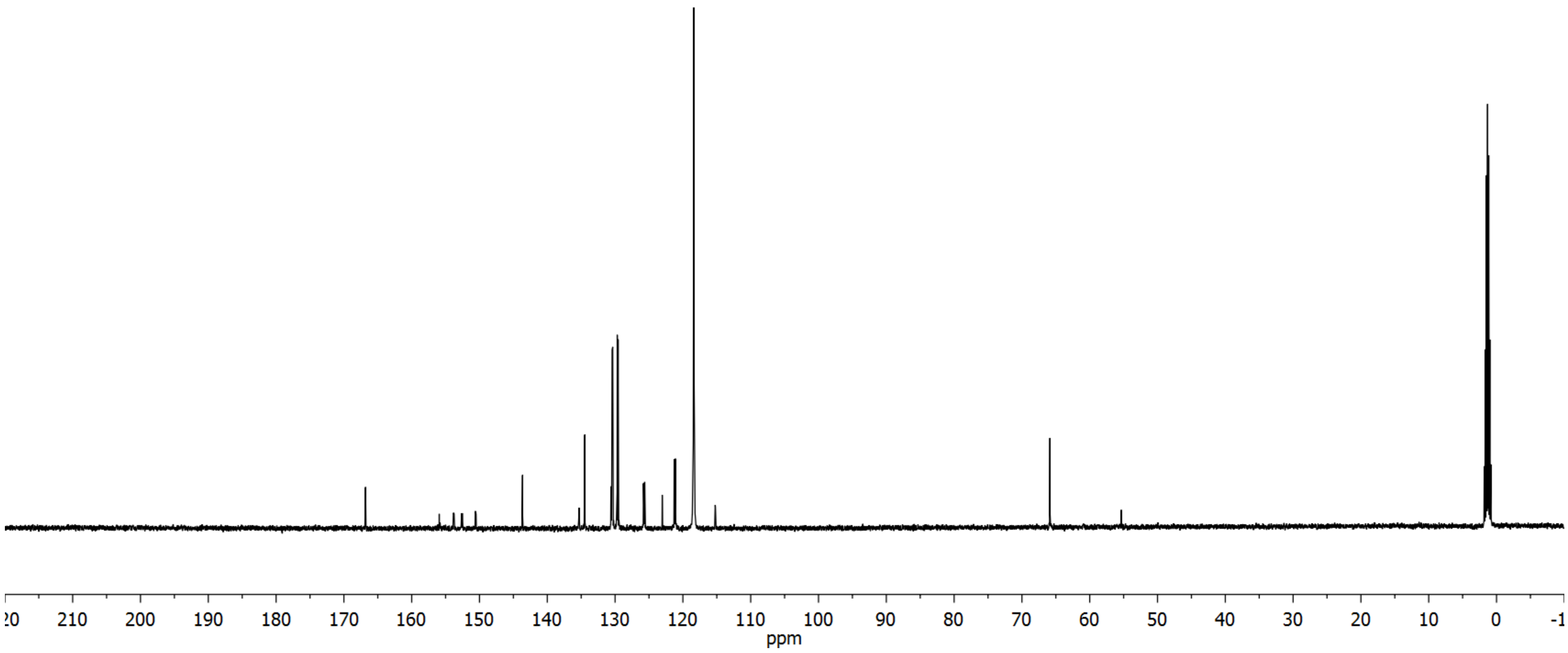

^{19}F NMR of benzyl benzoate tetrafluorothianthrenium salt (19-TFT)

CD_3CN, 25 °C

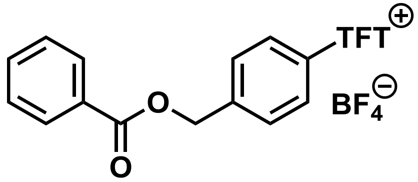

19-TFT

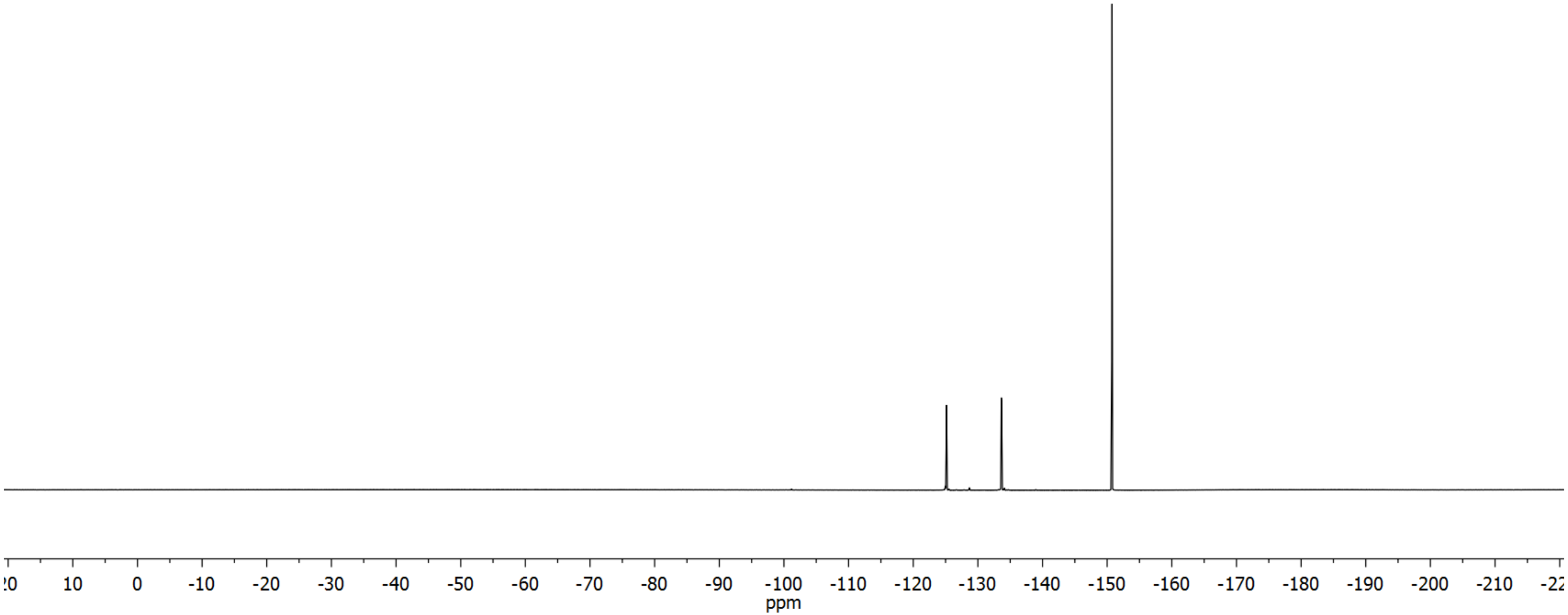

^{1}H NMR of 4-hydroxybenzyl benzoate (19)

CD_3CN, 25 °C

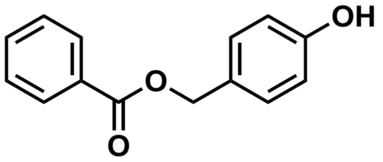

19

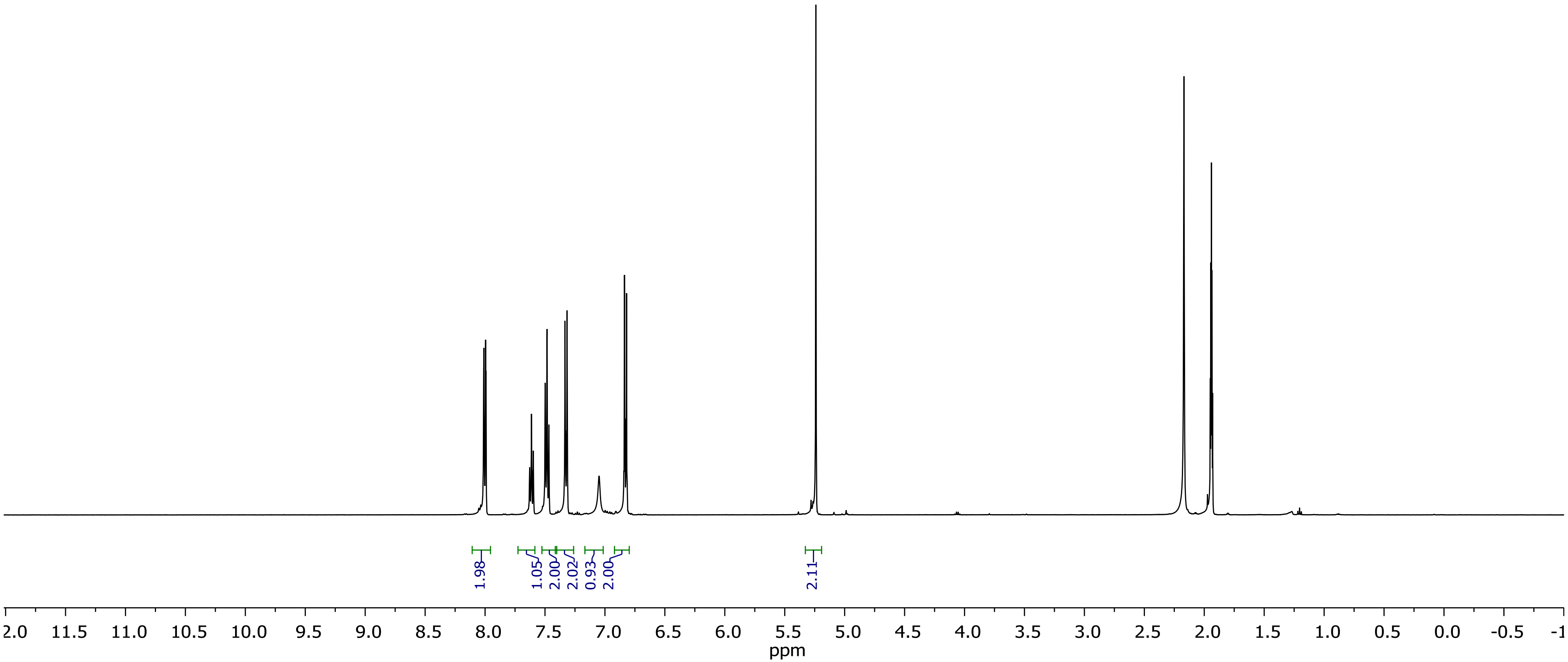

^{13}C NMR of 4-hydroxybenzyl benzoate (19)

CD_3CN, 25 °C

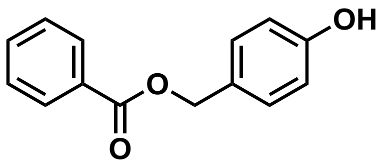

19

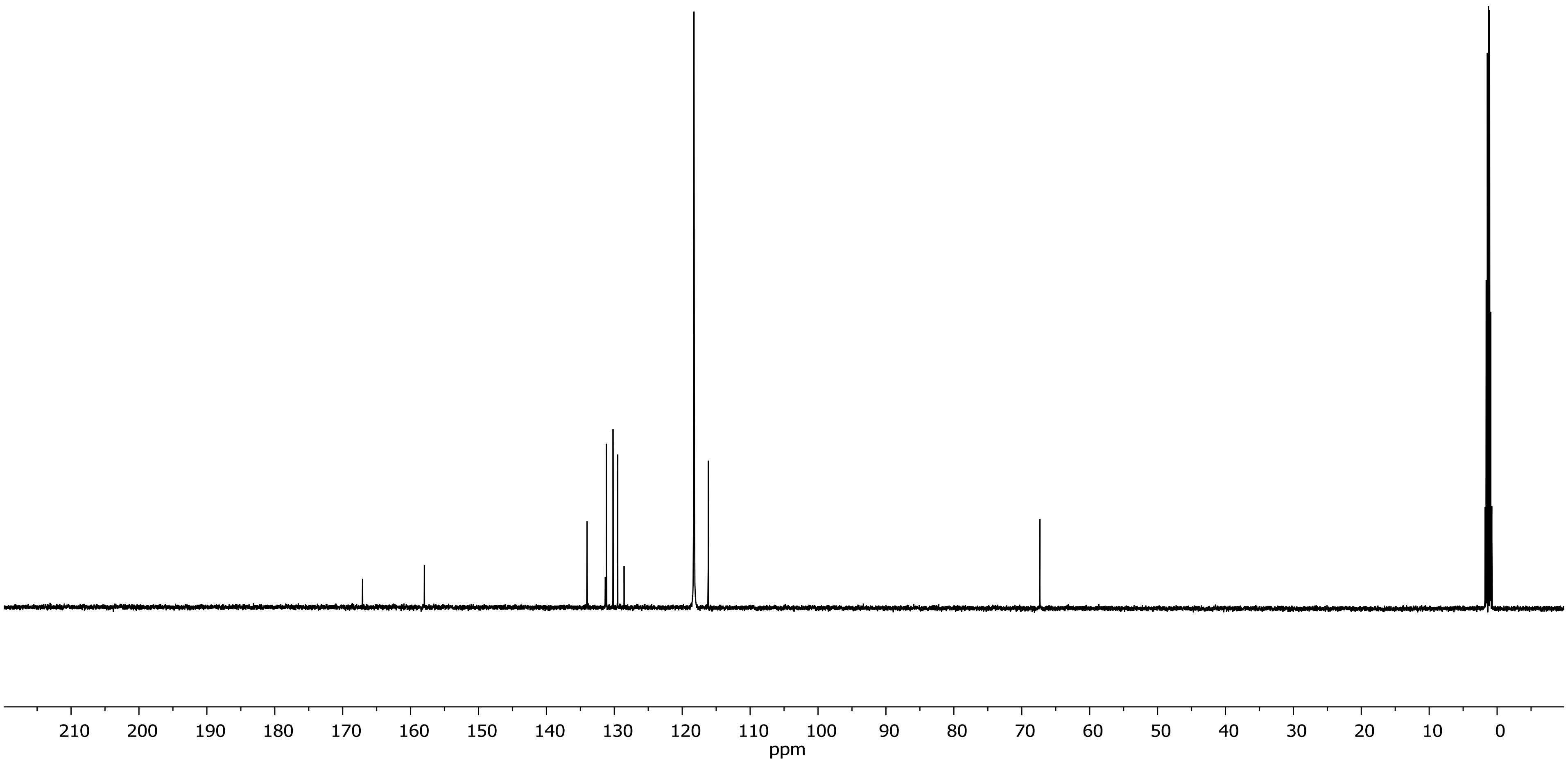

^{1}H NMR of N-benzyloxycarbonyl-4-*cis*-phenoxy-L-proline methyl ester tetrafluorothianthrenium salt (20-TFT)

CD_2Cl_2, 25 °C

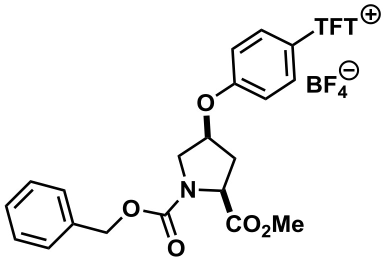

20-TFT

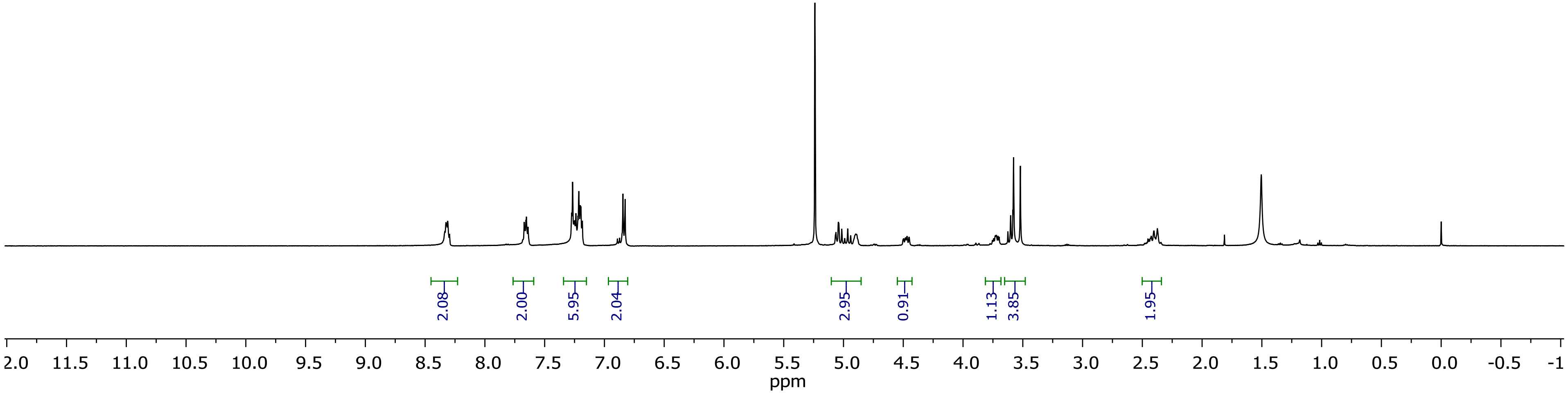

^{13}C NMR of N-benzyloxycarbonyl-4-*cis*-phenoxy-L-proline methyl ester tetrafluorothianthrenium salt (20-TFT)

CD_2Cl_2, 25 °C

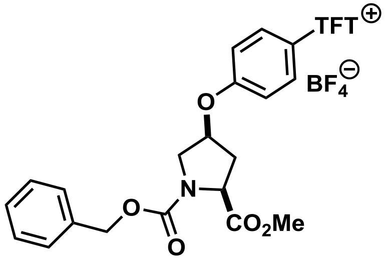

20-TFT

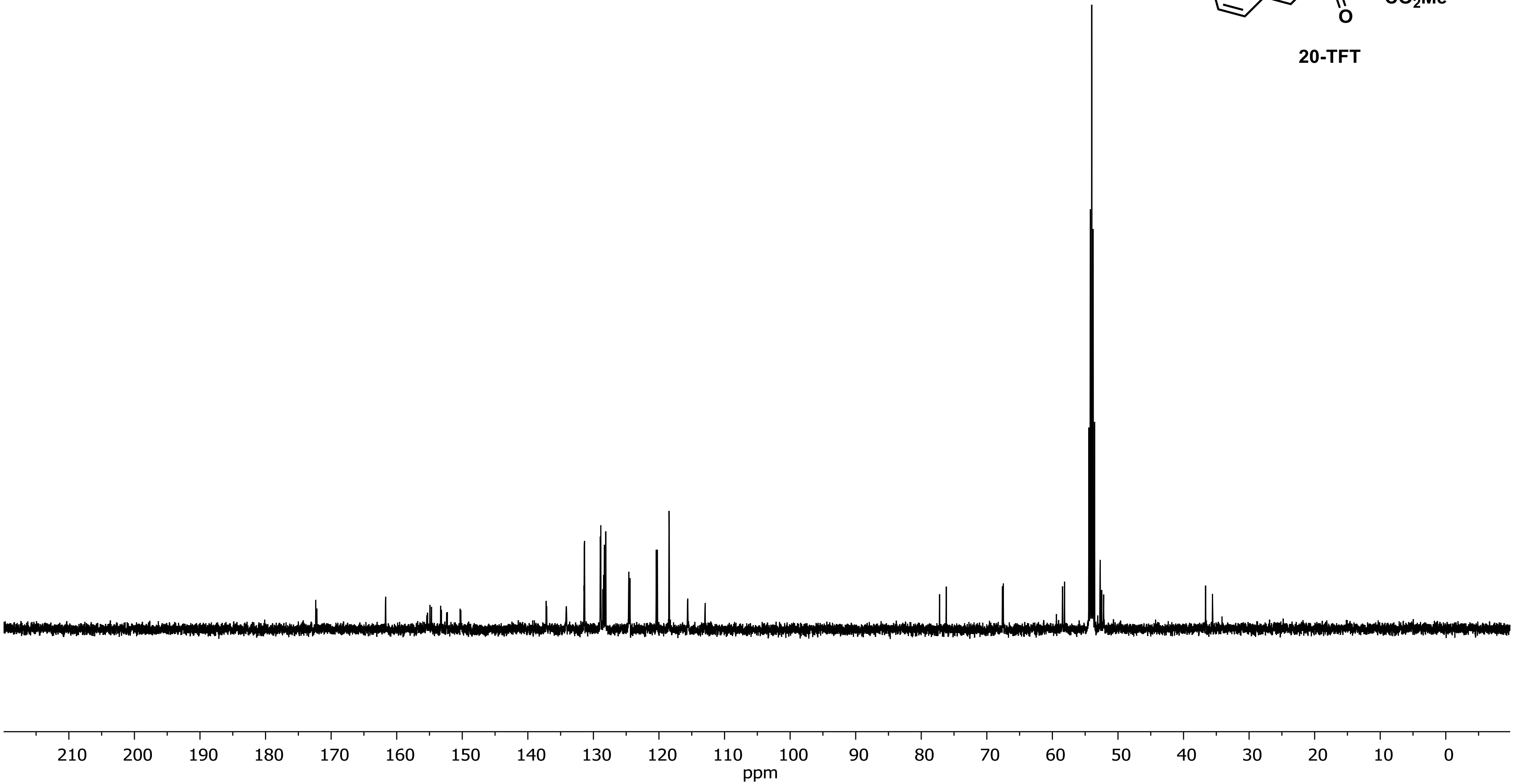

^{19}F NMR of N-benzyloxycarbonyl-4-*cis*-phenoxy-L-proline methyl ester tetrafluorothianthrenium salt (20-TFT)

CD_2Cl_2, 25 °C

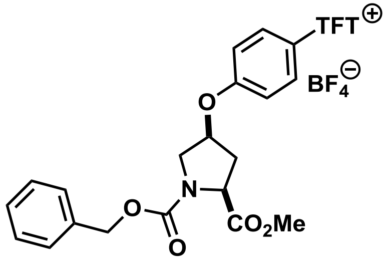

20-TFT

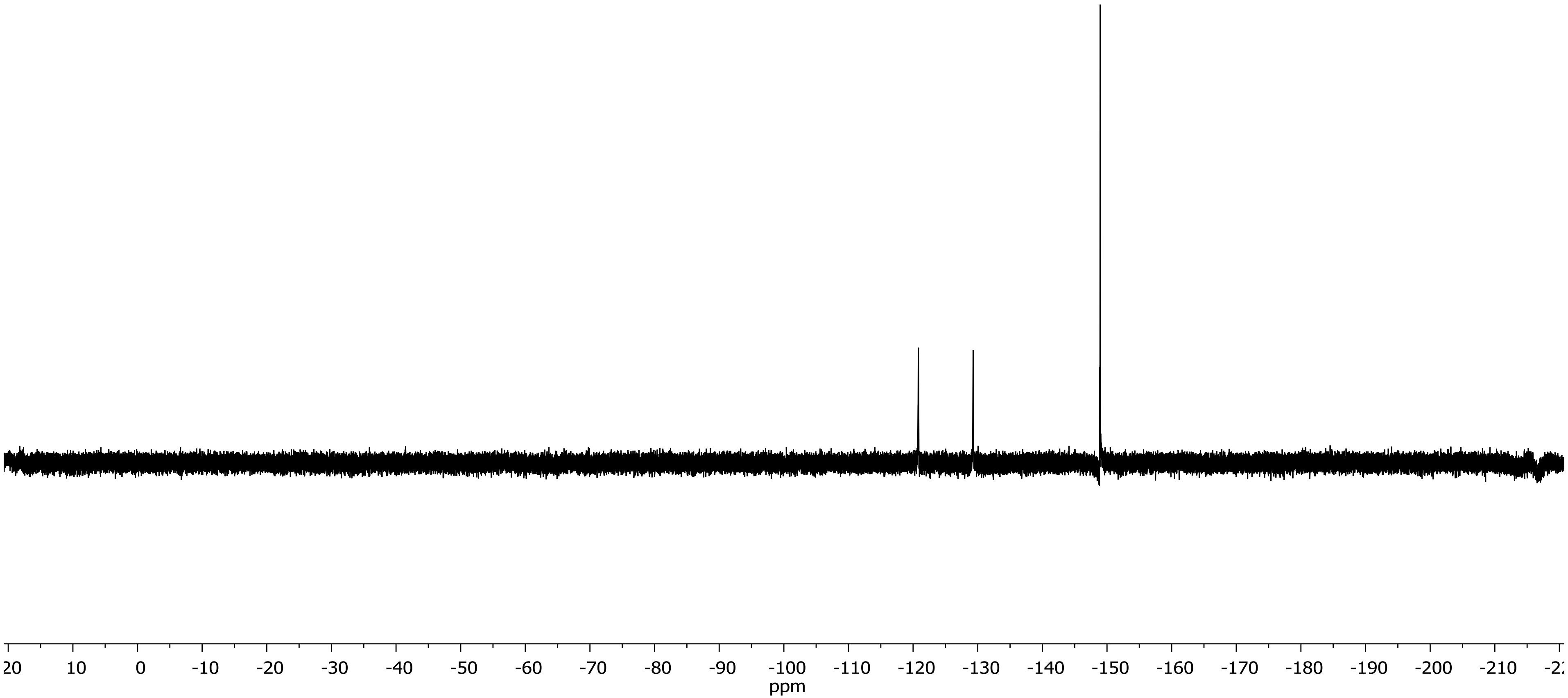

^{1}H NMR of hydroxy-N-benzyloxycarbonyl-4-*cis*-phenoxy-L-proline methyl ester (20)

CD_3CN, 25 °C

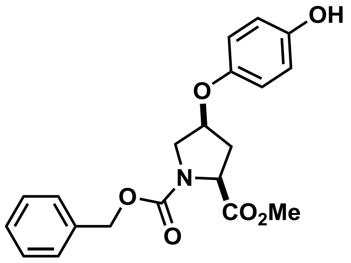

20

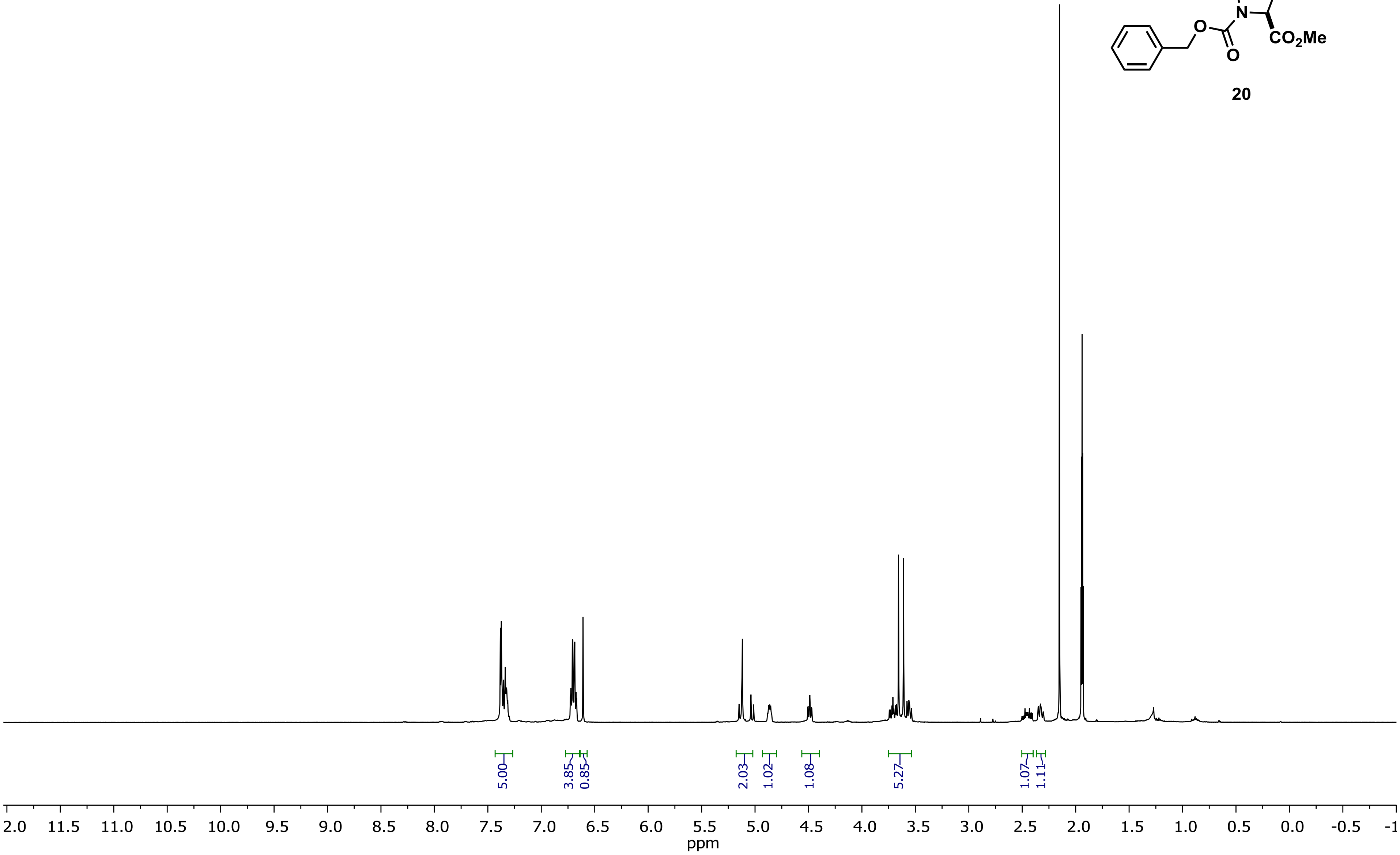

^{13}C NMR of hydroxy-N-benzyloxycarbonyl-4-*cis*-phenoxy-L-proline methyl ester (20)

CD_3CN, 25 °C

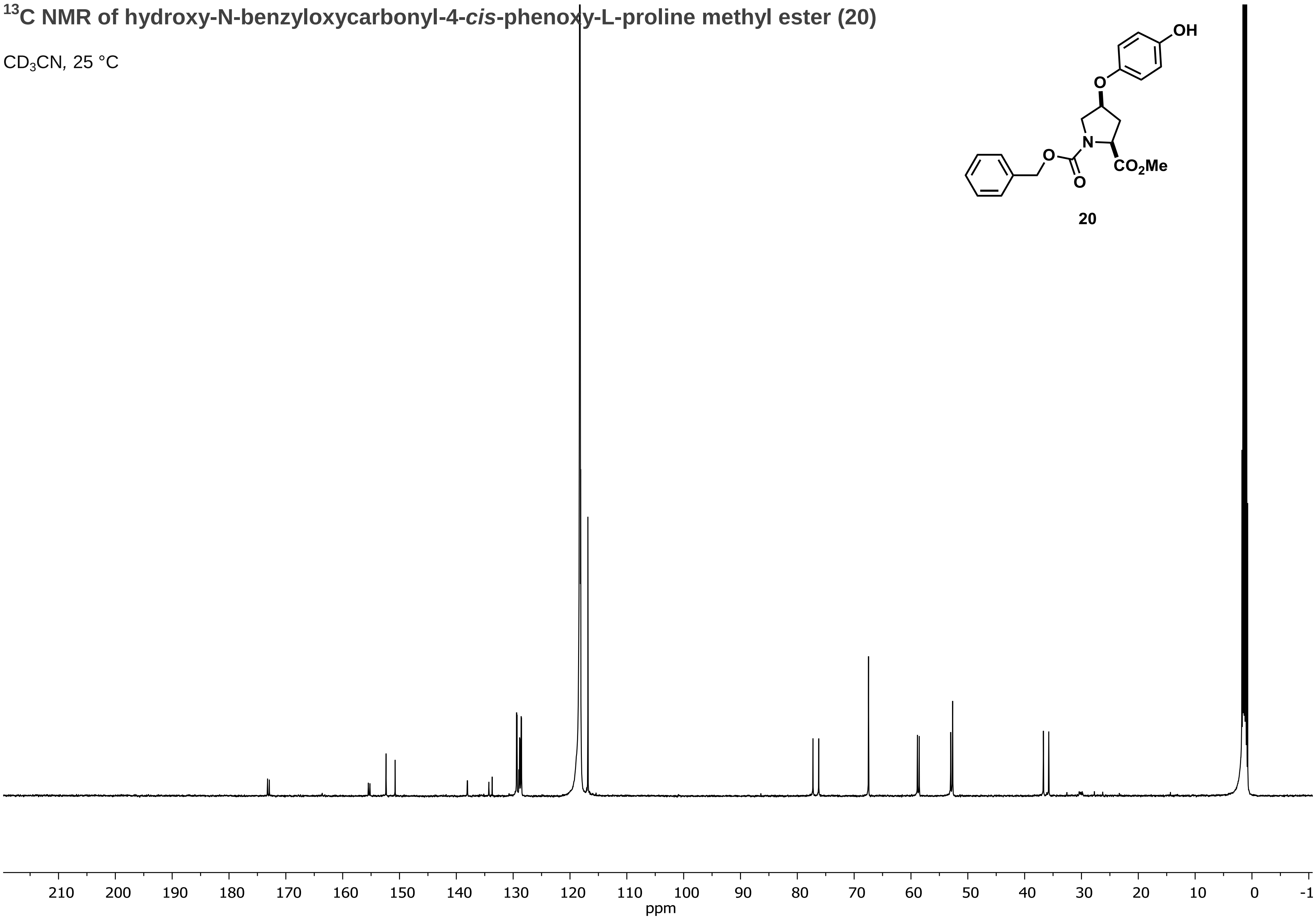

^{1}H-^{13}C HSQC NMR of hydroxy-N-benzyloxycarbonyl-4-*cis*-phenoxy-L-proline methyl ester (20)

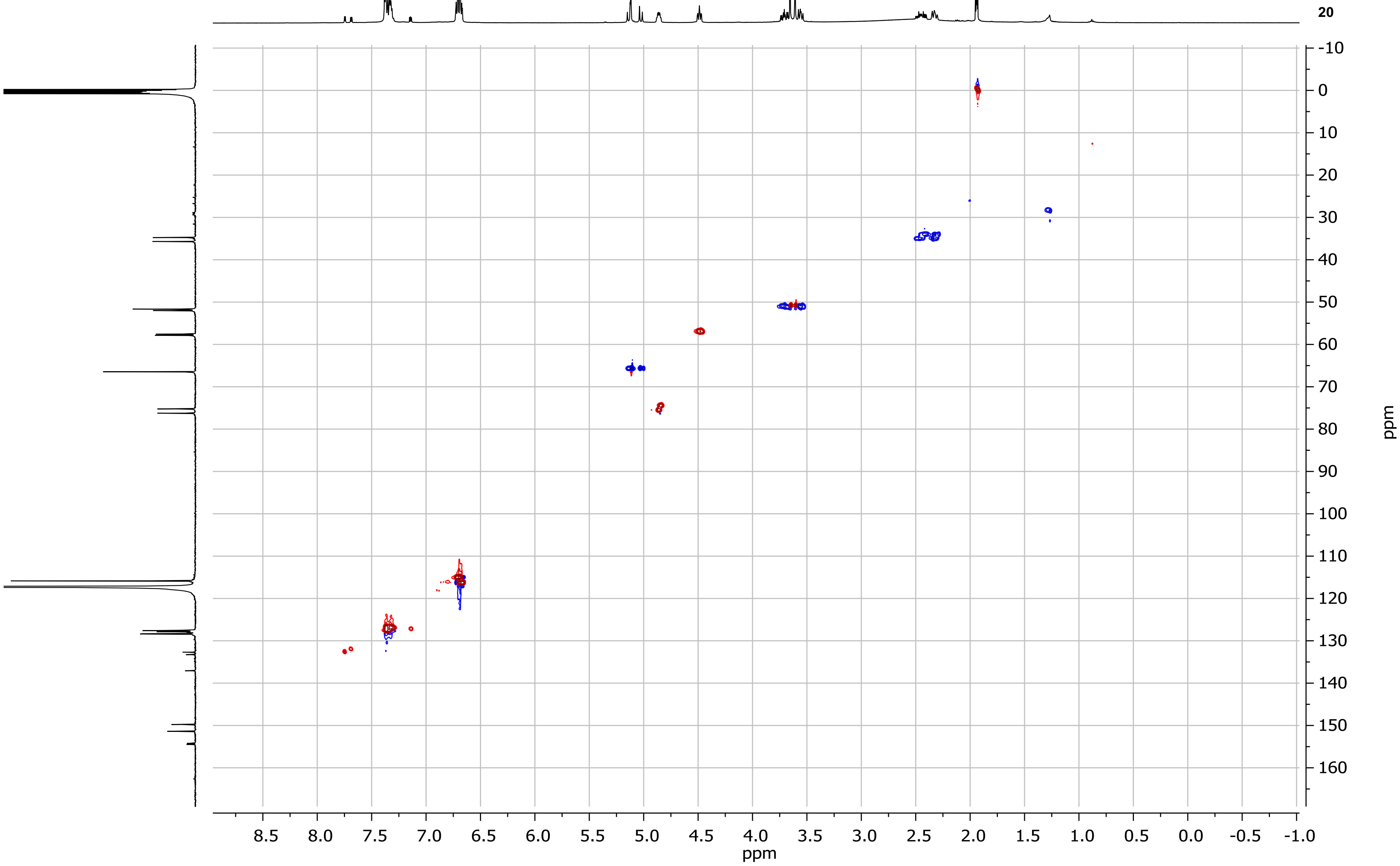

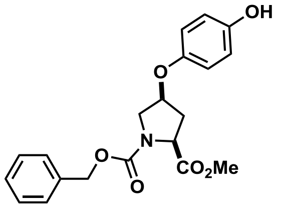

20

NOESY NMR of hydroxy-N-benzyloxycarbonyl-4-*cis*-phenoxy-L-proline methyl ester (20)

CD_3CN, 25 °C

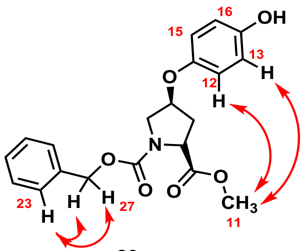

20

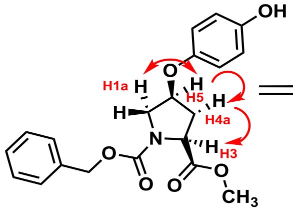

20

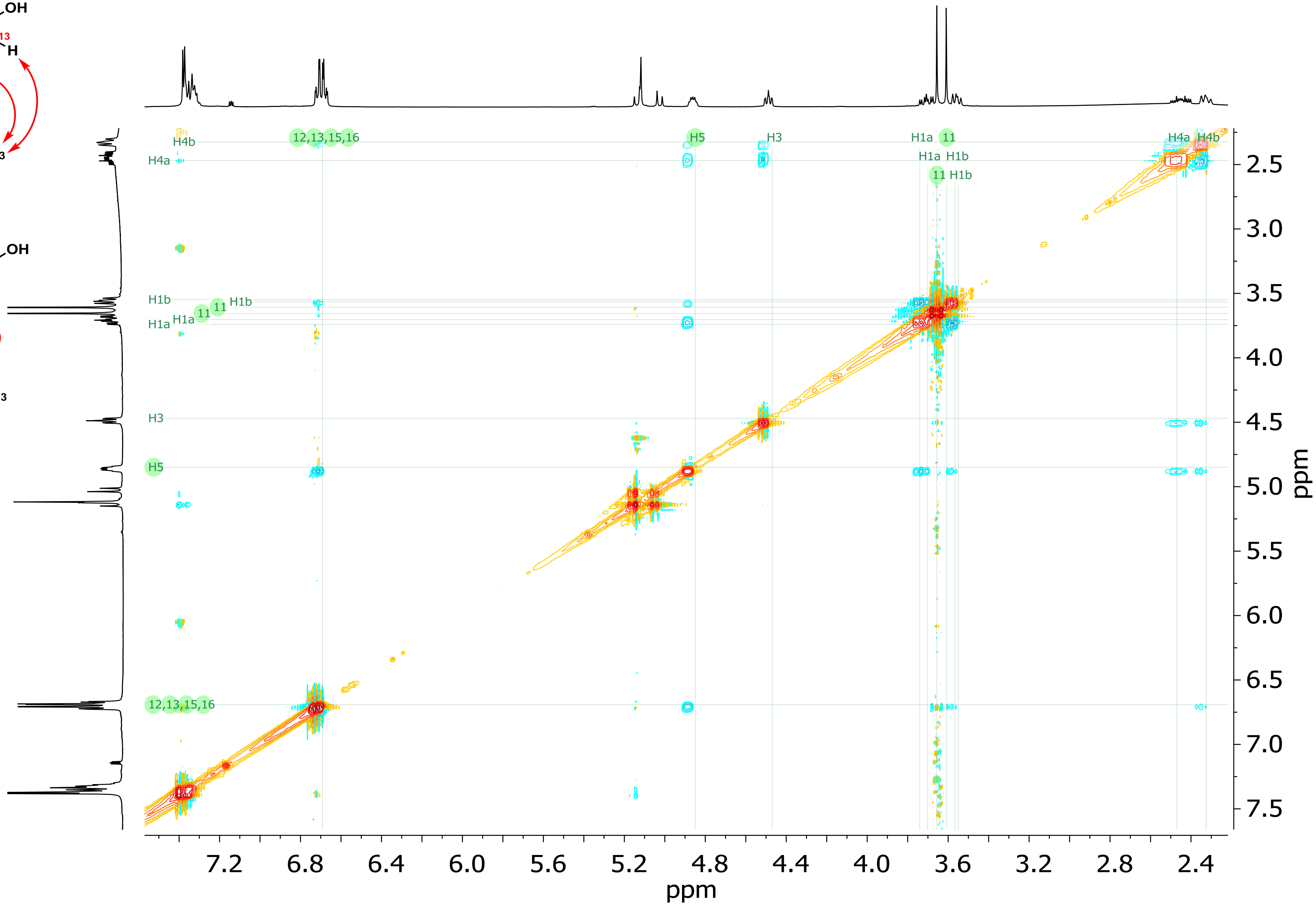

^{1}H NMR of hydroxy-etofenprox (21)

CDCl$_3$, 25 °C

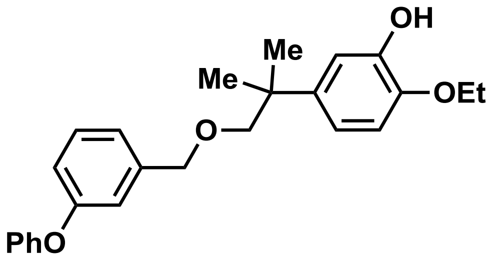

21

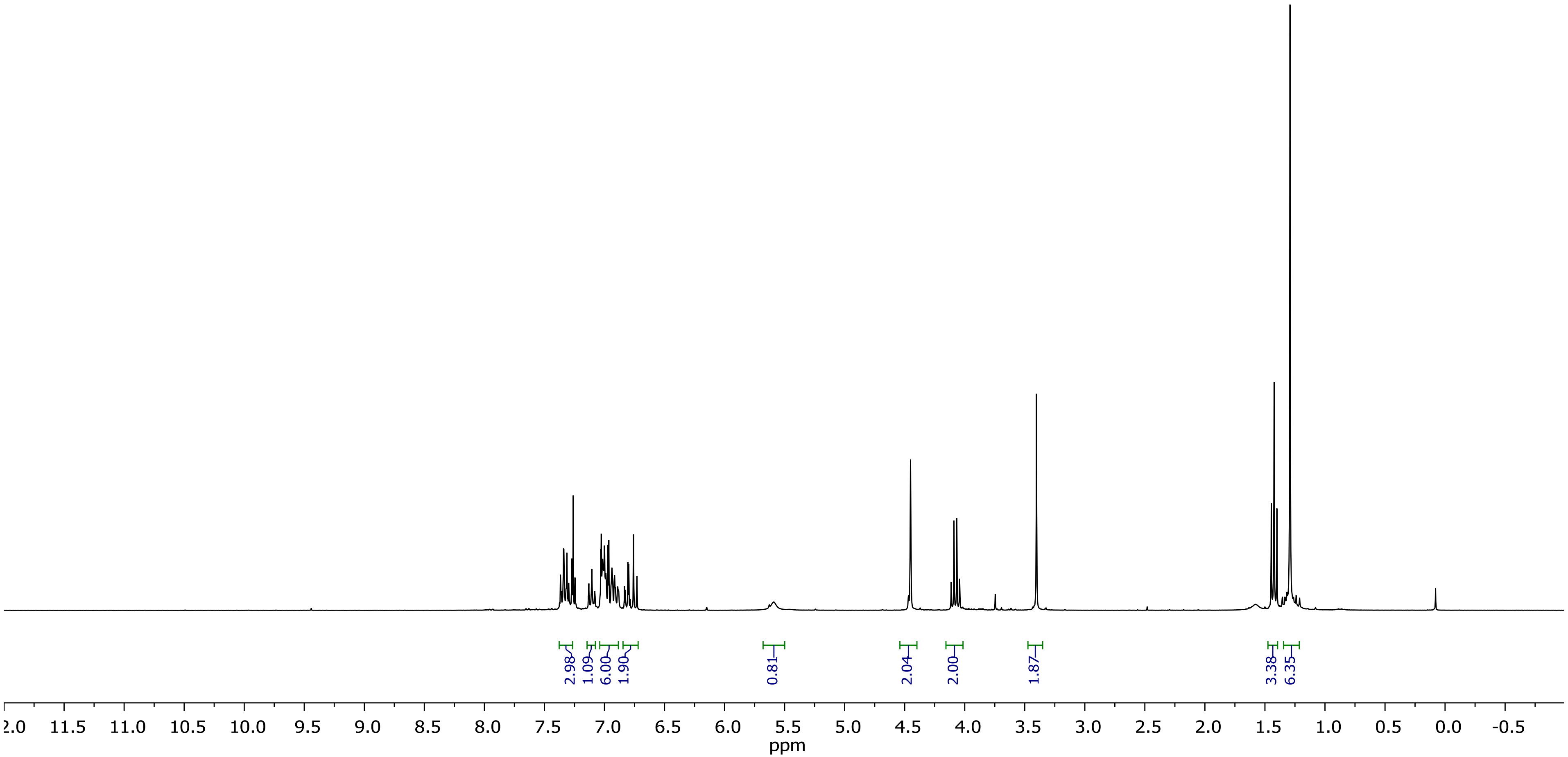

^{13}C NMR of hydroxy-etofenprox (21)

$CDCl_3$, 25 °C

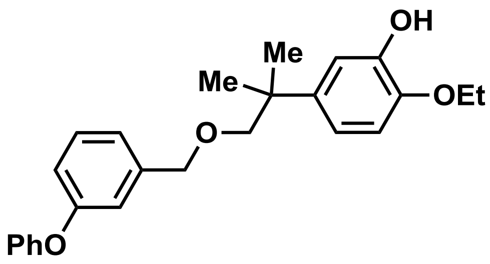

21

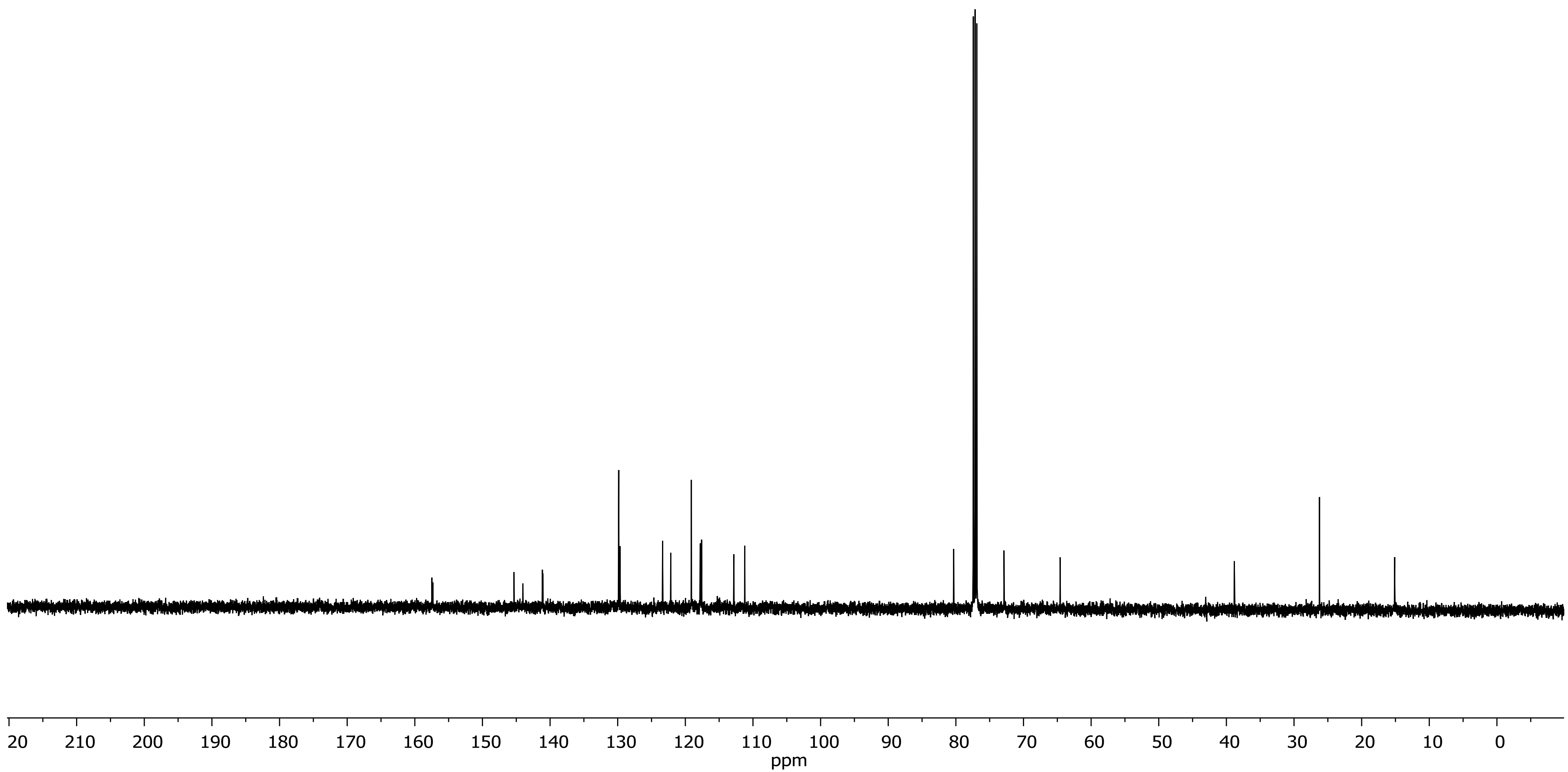

^{1}H NMR of biphenyl tetrafluorothianthrenium salt (22-TFT)

CD_3CN, 25 °C

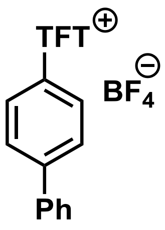

22-TFT

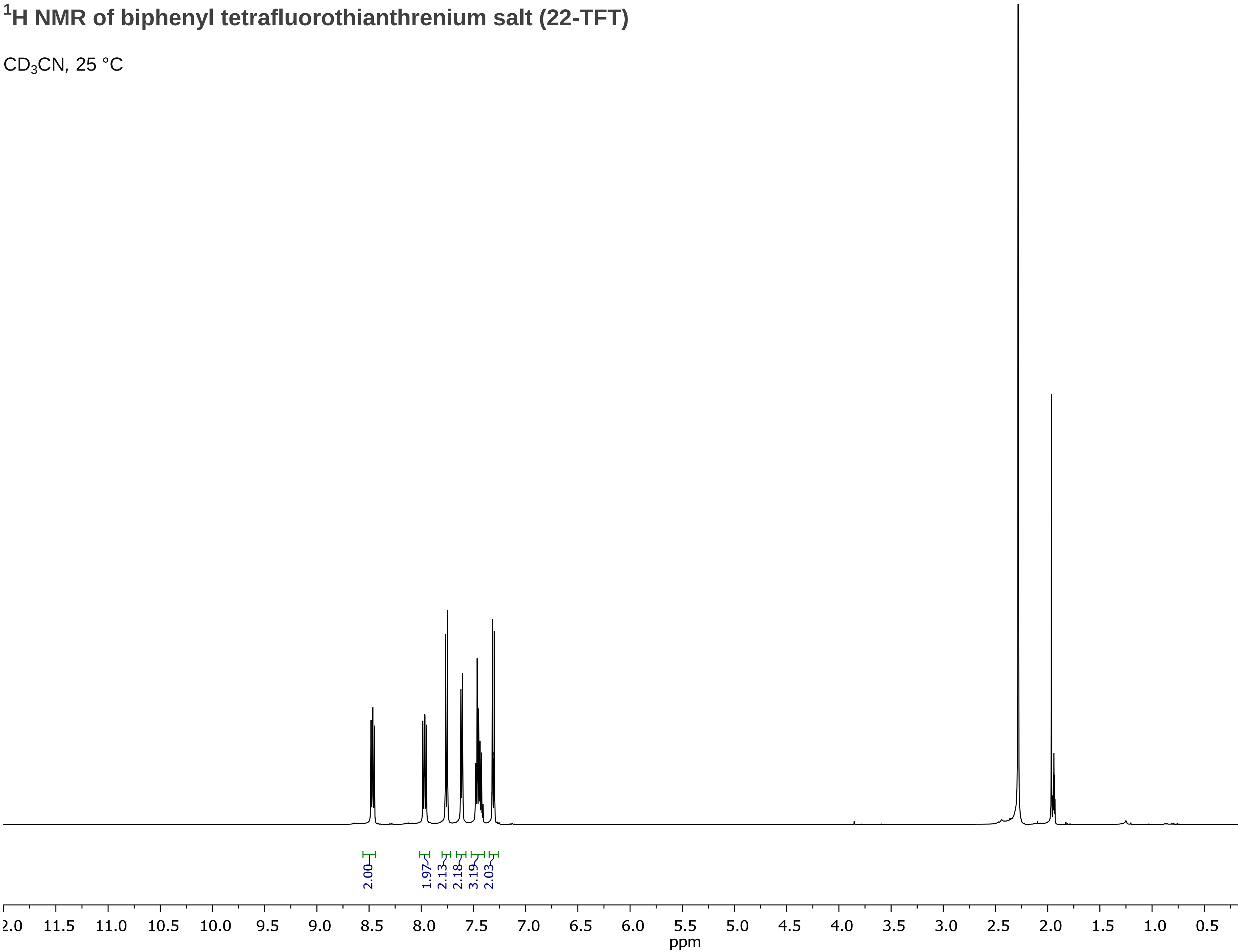

^{13}C NMR of biphenyl tetrafluorothianthrenium salt (22-TFT)

CD_3CN, 25 °C

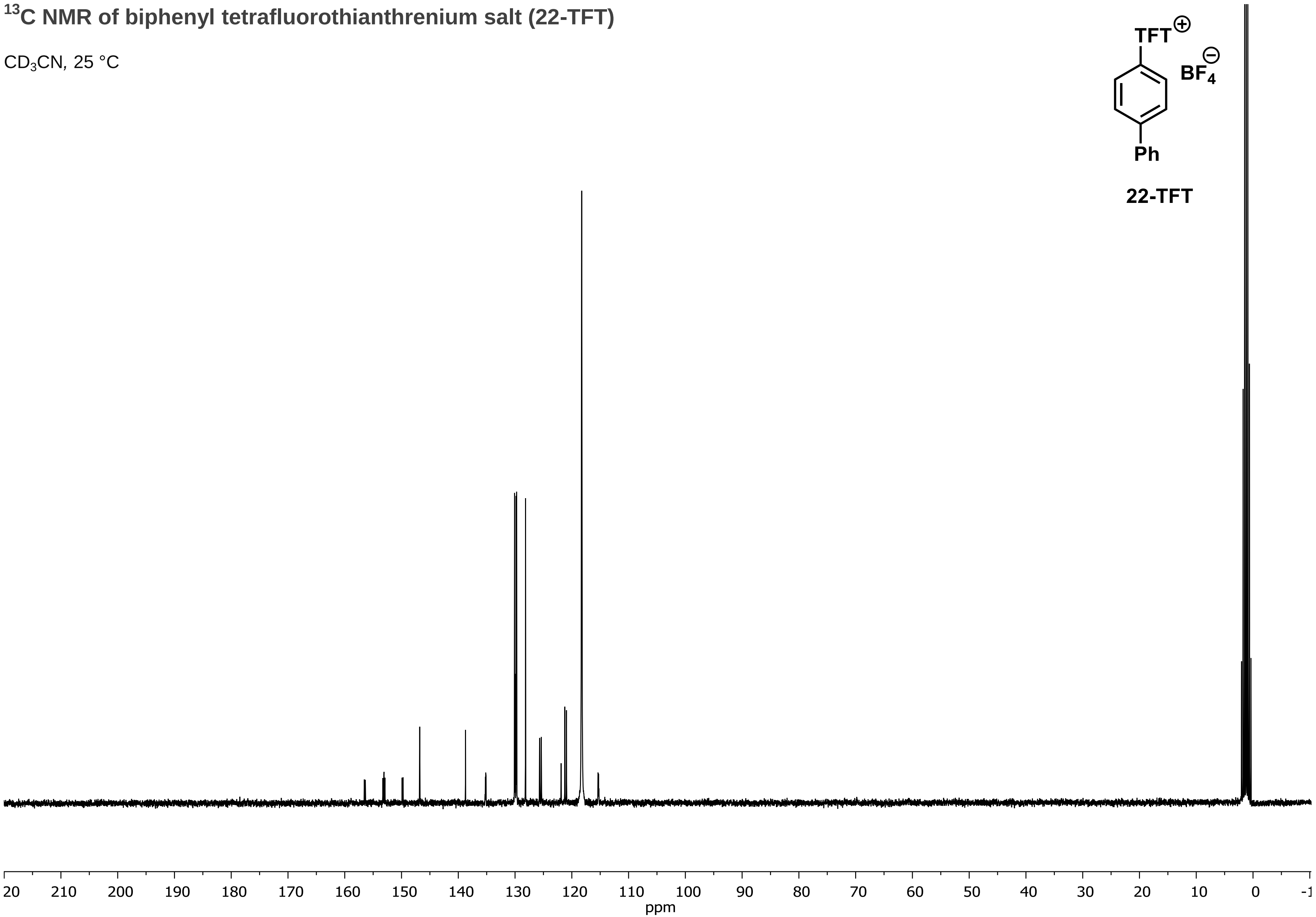

^{19}F NMR of biphenyl tetrafluorothianthrenium salt (22-TFT)

CD_3CN, 25 °C

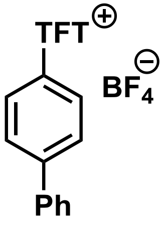

22-TFT

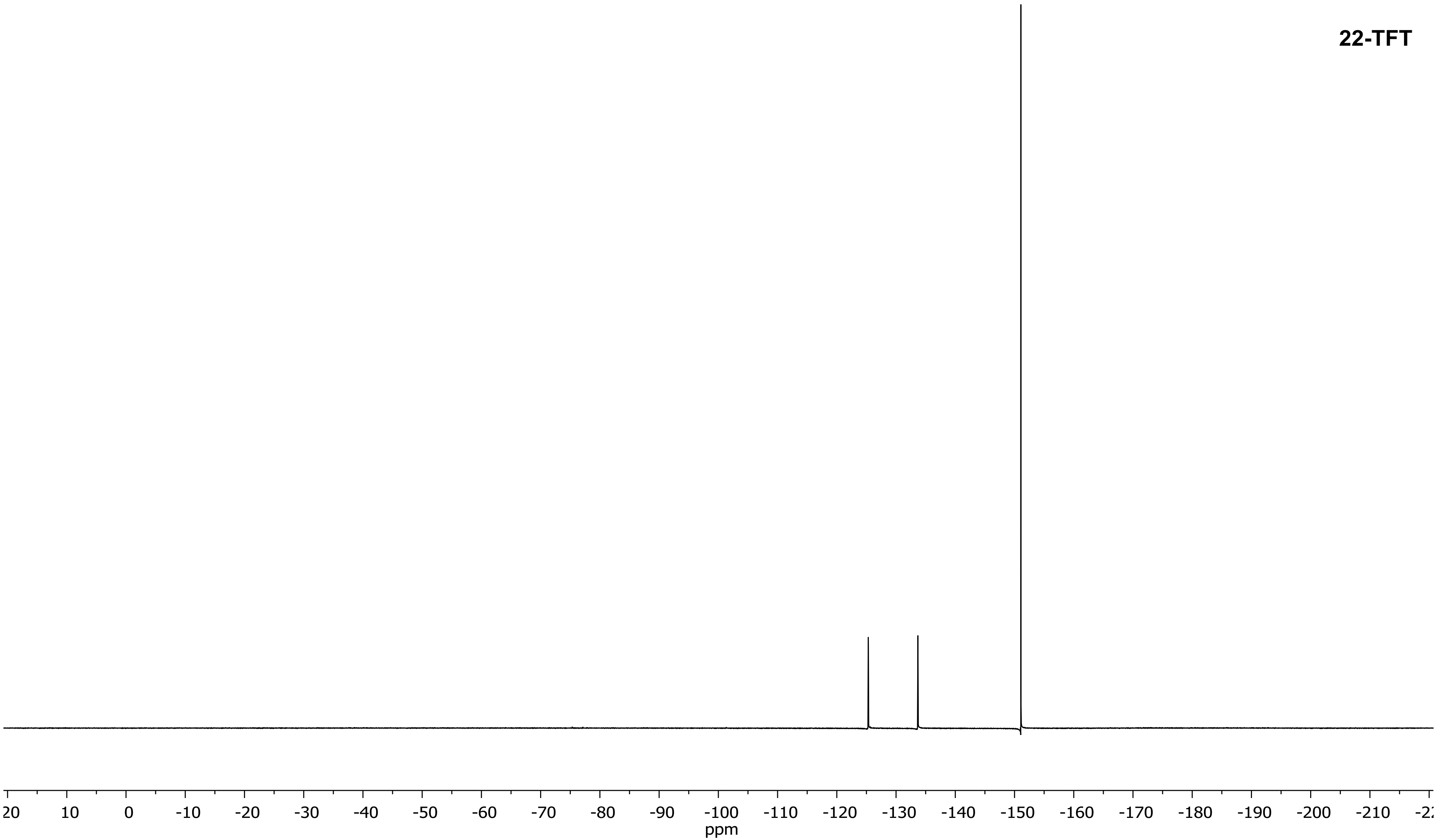

^{1}H NMR of biphenyl thianthrenium salt (22-TT)

DMSO-d_6, 25 °C

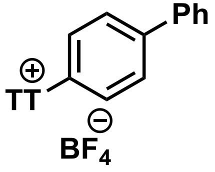

22-TT

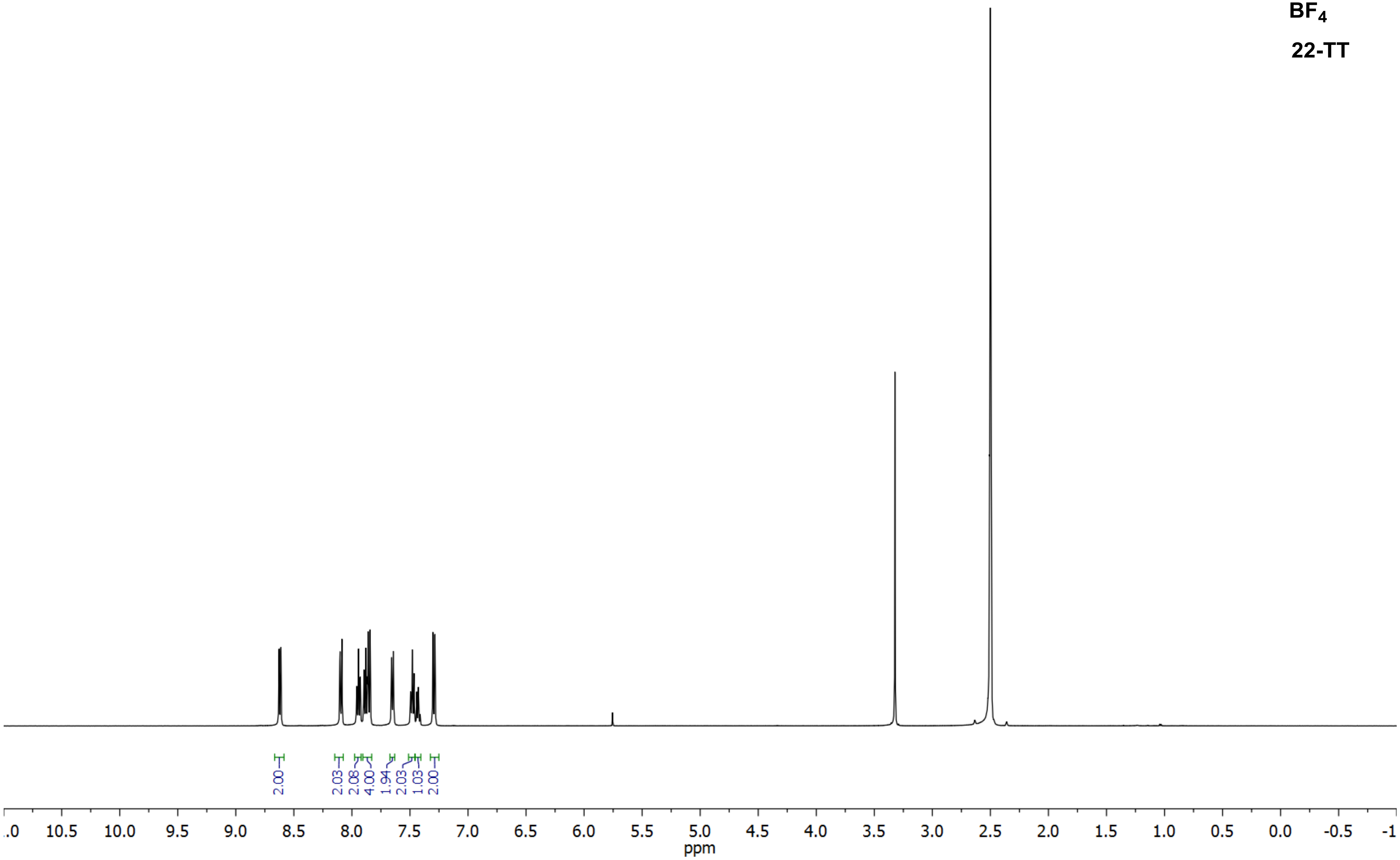

^{13}C NMR of biphenyl thianthrenium salt (22-TT)

CD_3CN, 25 °C

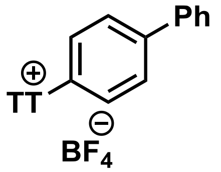

22-TT

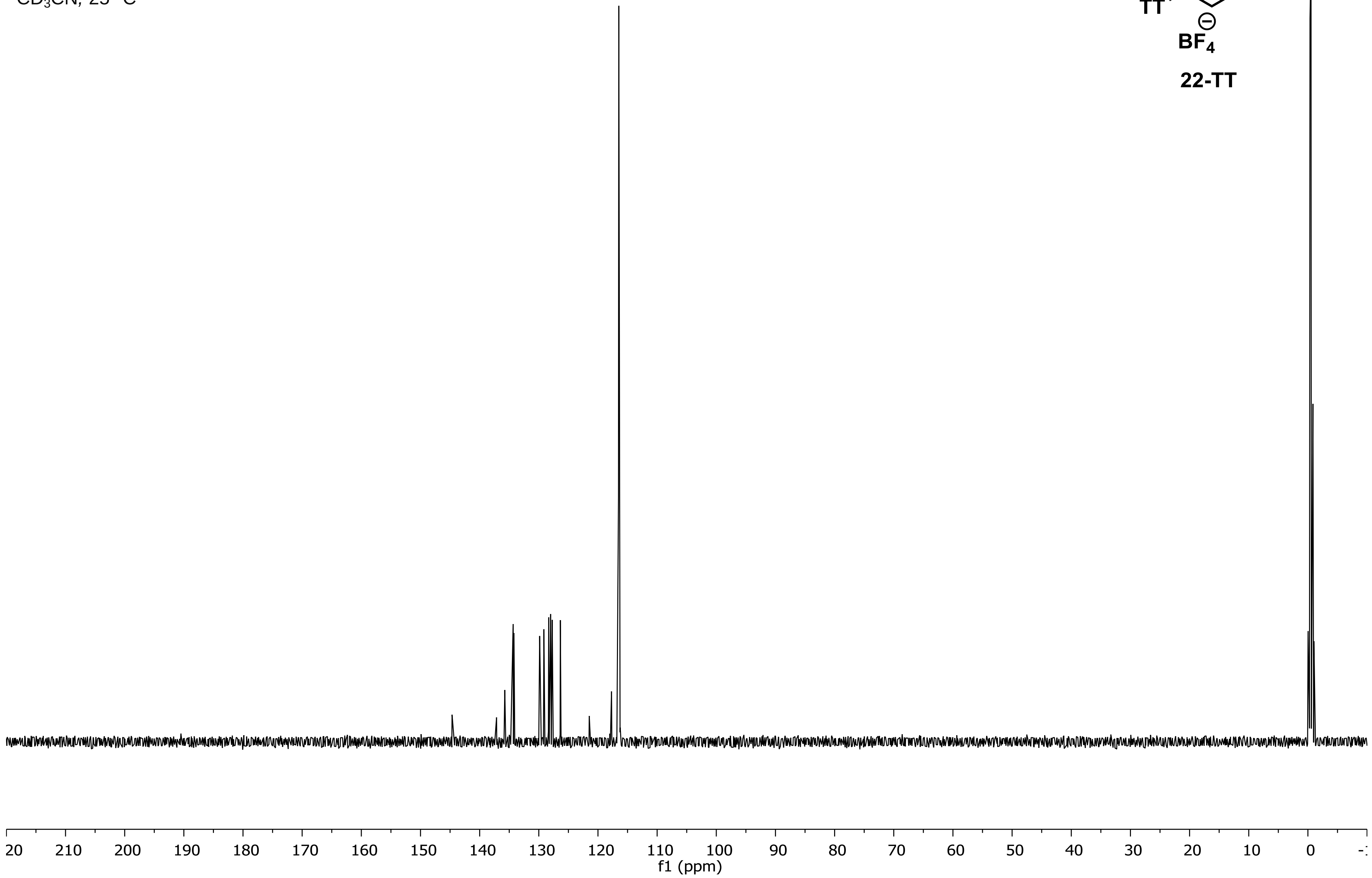

^{19}F NMR of biphenyl thianthrenium salt (22-TT)

CD_3CN, 25 °C

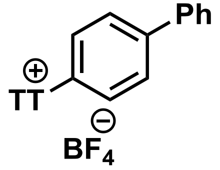

22-TT

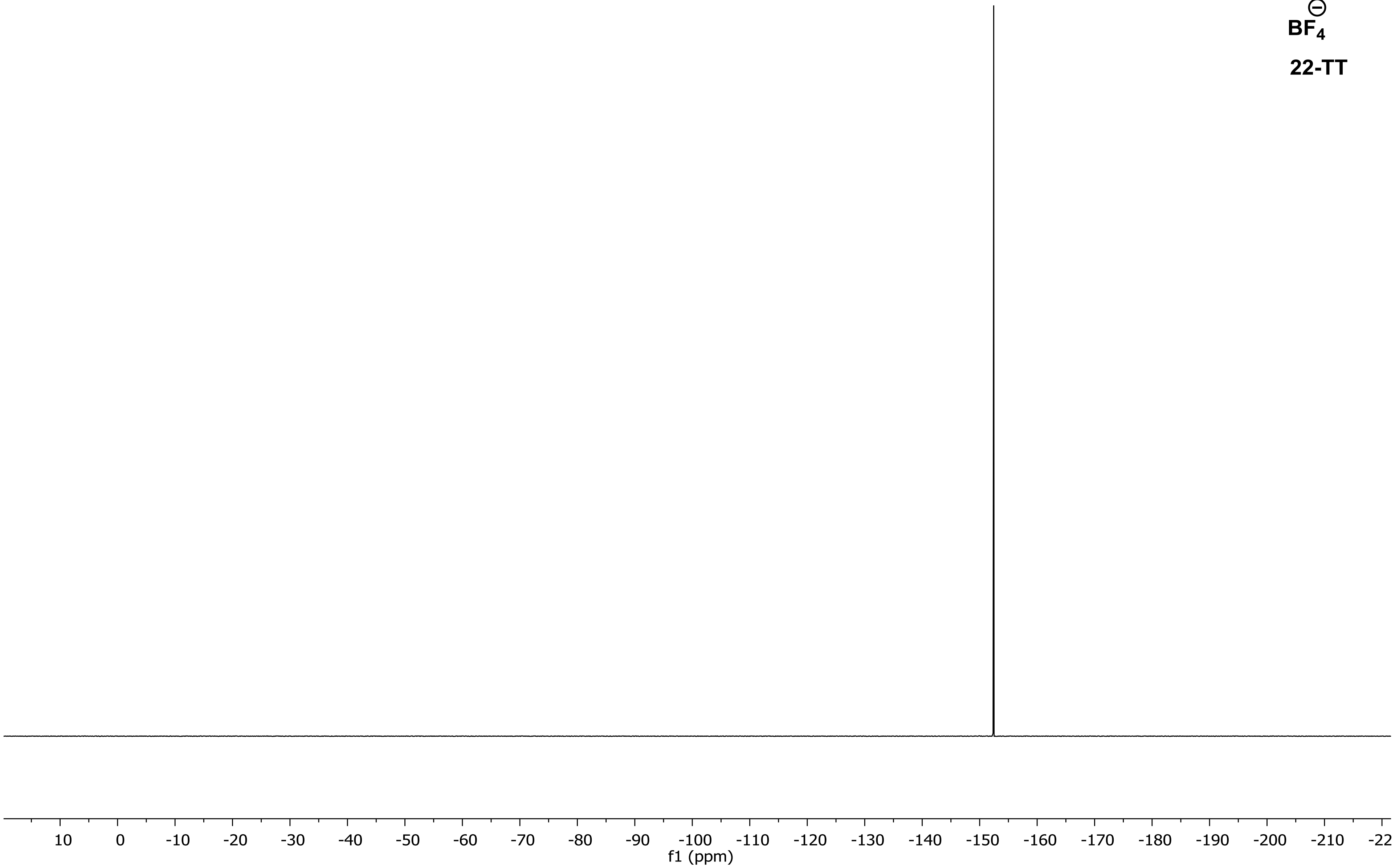

^{1}H NMR of 4-phenylphenol (22)

CD_3CN, 25 °C

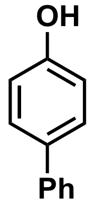

22

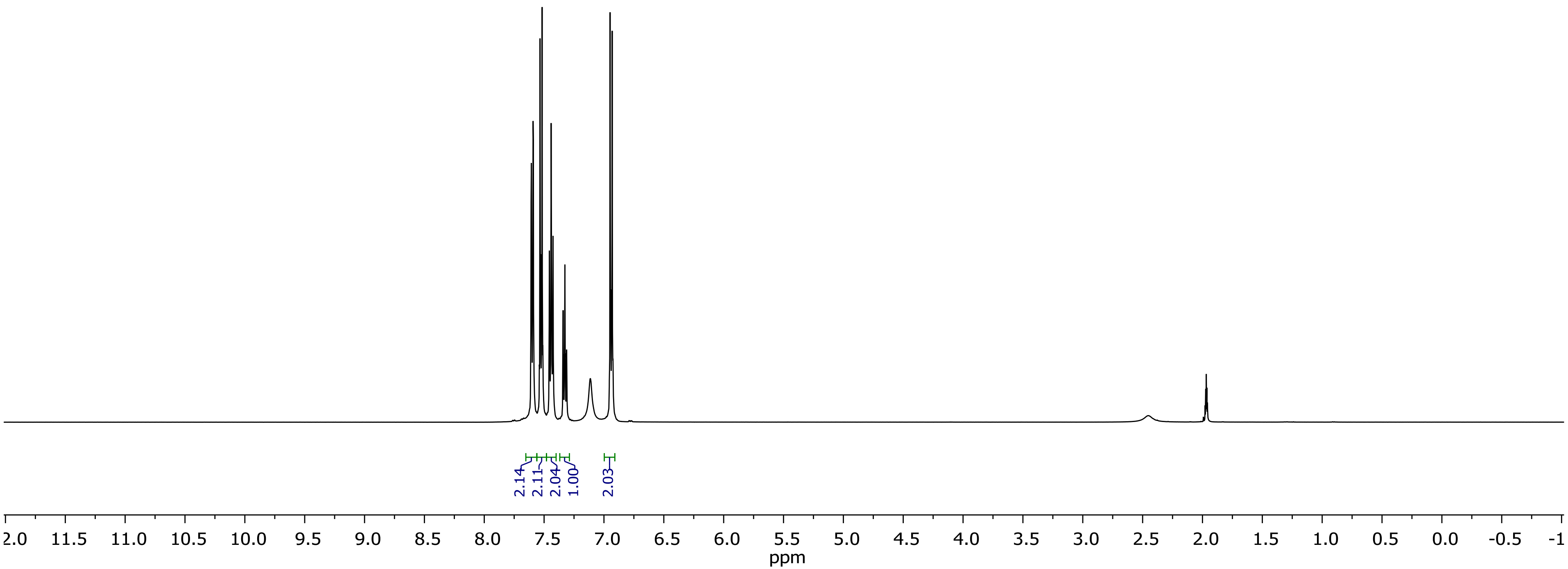

^{13}C NMR of 4-phenylphenol (22)

CD_3CN, 25 °C

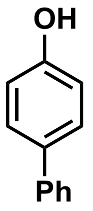

22

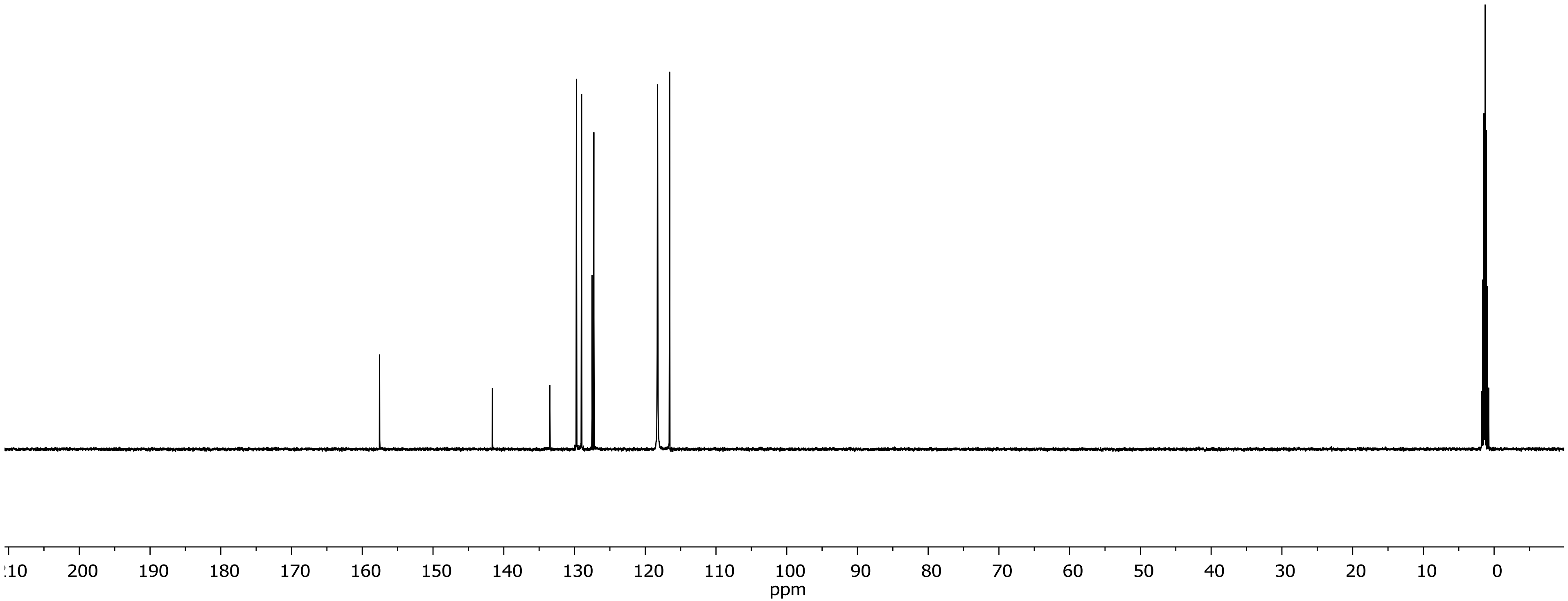

^{1}H NMR of (3r,5r,7r)-1-(*p*-tolyl)adamantane tetrafluorothianthrenium salt (23-TFT)

CD_3CN, 25 °C

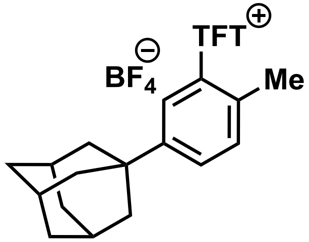

23-TFT

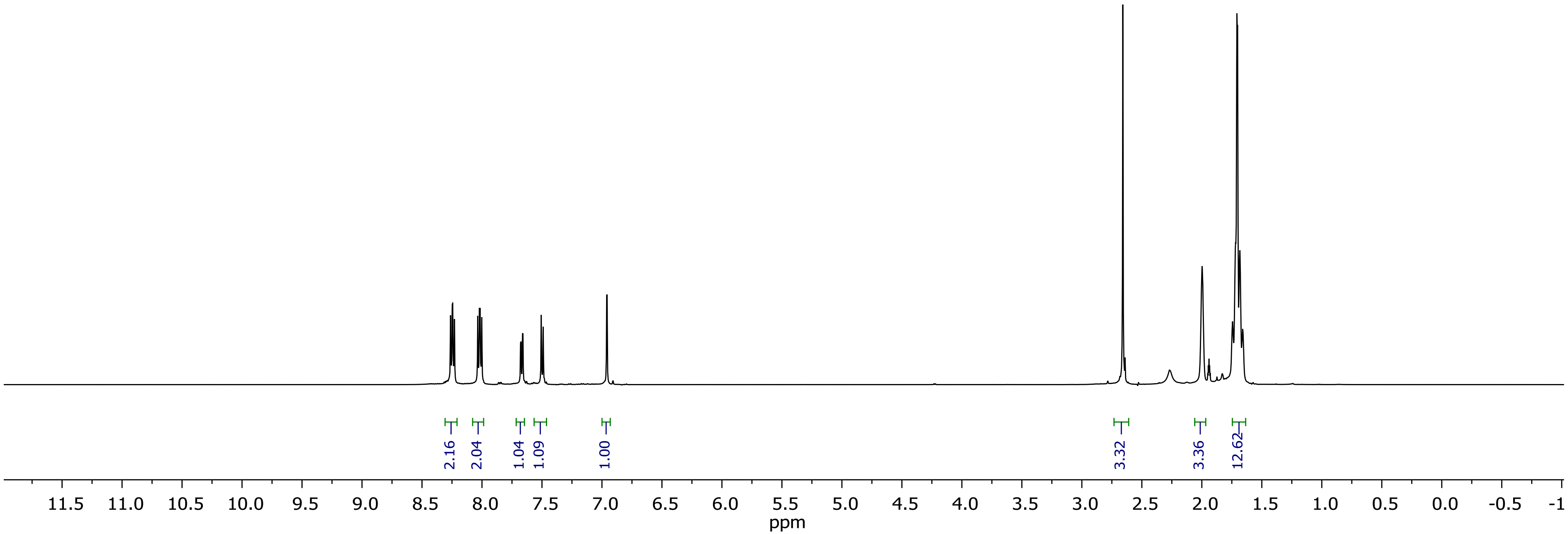

^{13}C NMR of (3r,5r,7r)-1-(*p*-tolyl)adamantane tetrafluorothianthrenium salt (23-TFT)

CD_3CN, 25 °C

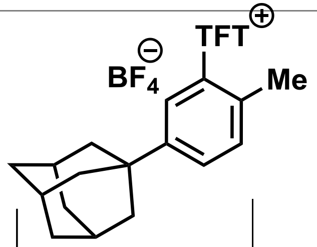

23-TFT

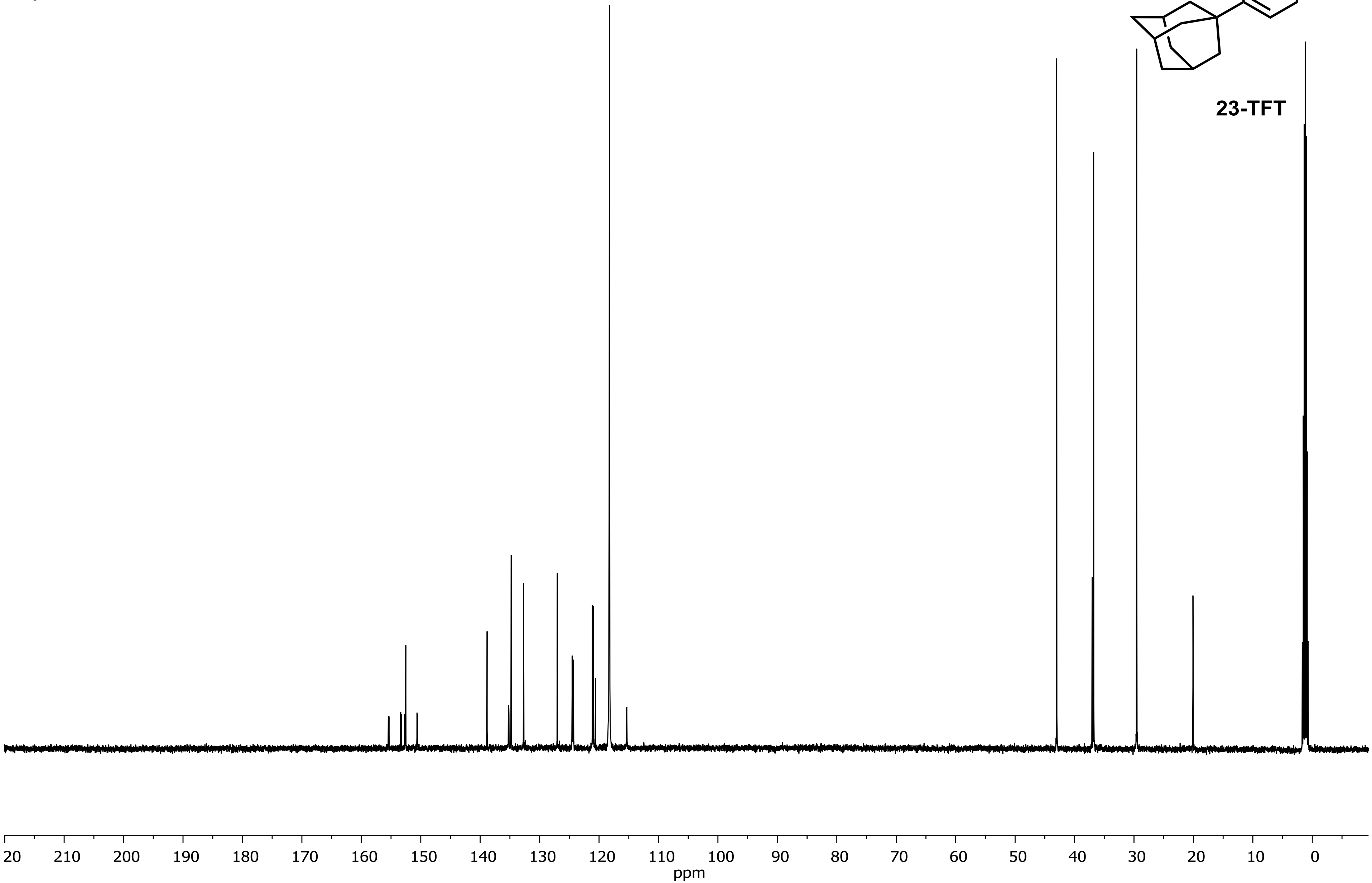

^{19}F NMR of (3r,5r,7r)-1-(*p*-tolyl)adamantane tetrafluorothianthrenium salt (23-TFT)

CD_3CN, 25 °C

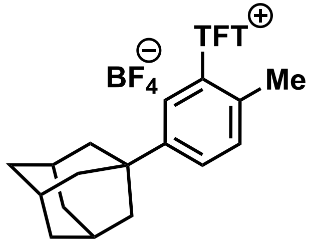

23-TFT

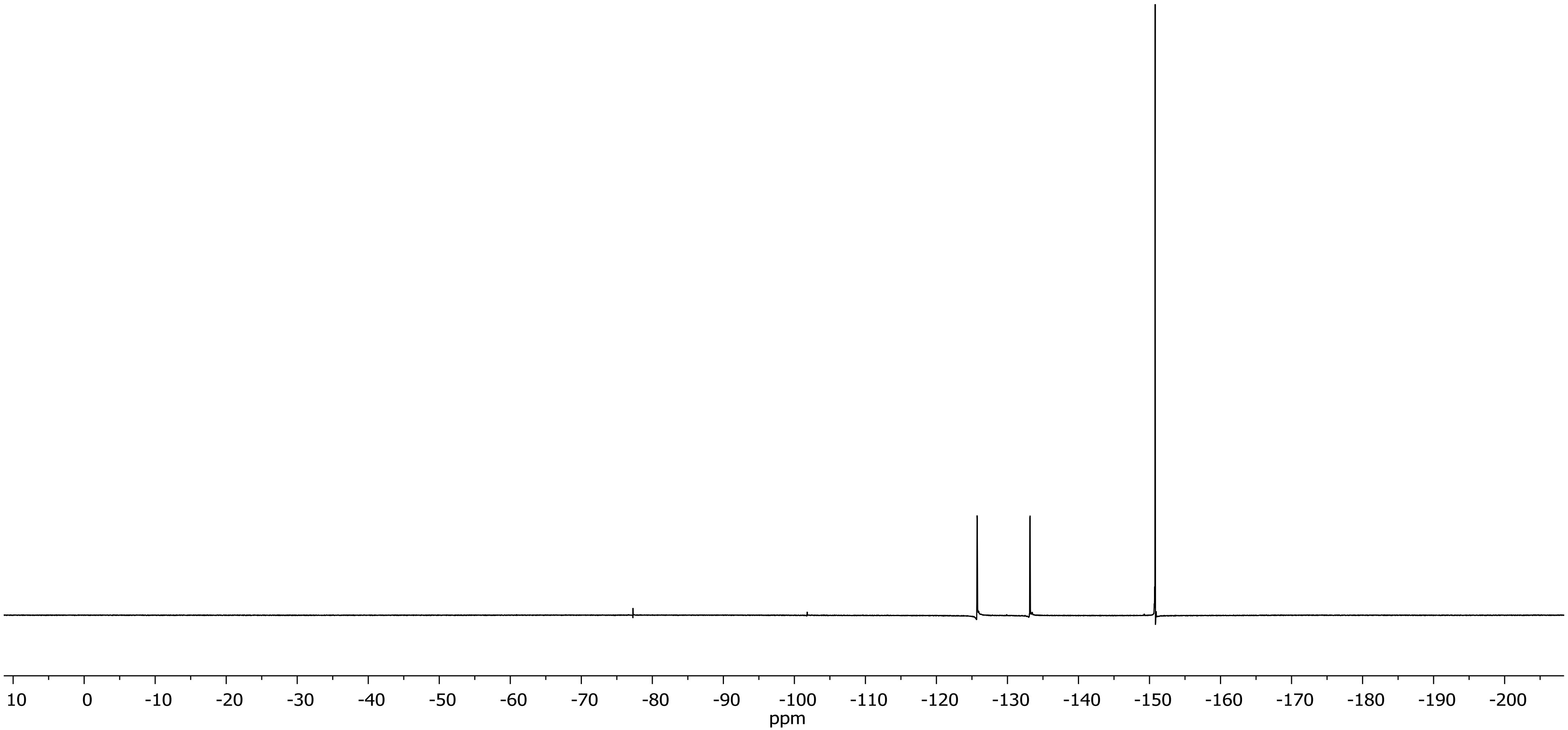

^{1}H NMR of 5-((3r,5r,7r)-adamantan-1-yl)-2-methylphenol (23)

CD_3CN, 25 °C

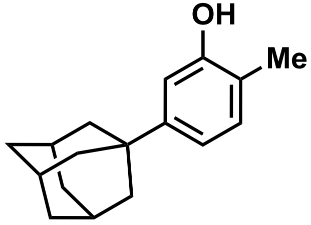

23

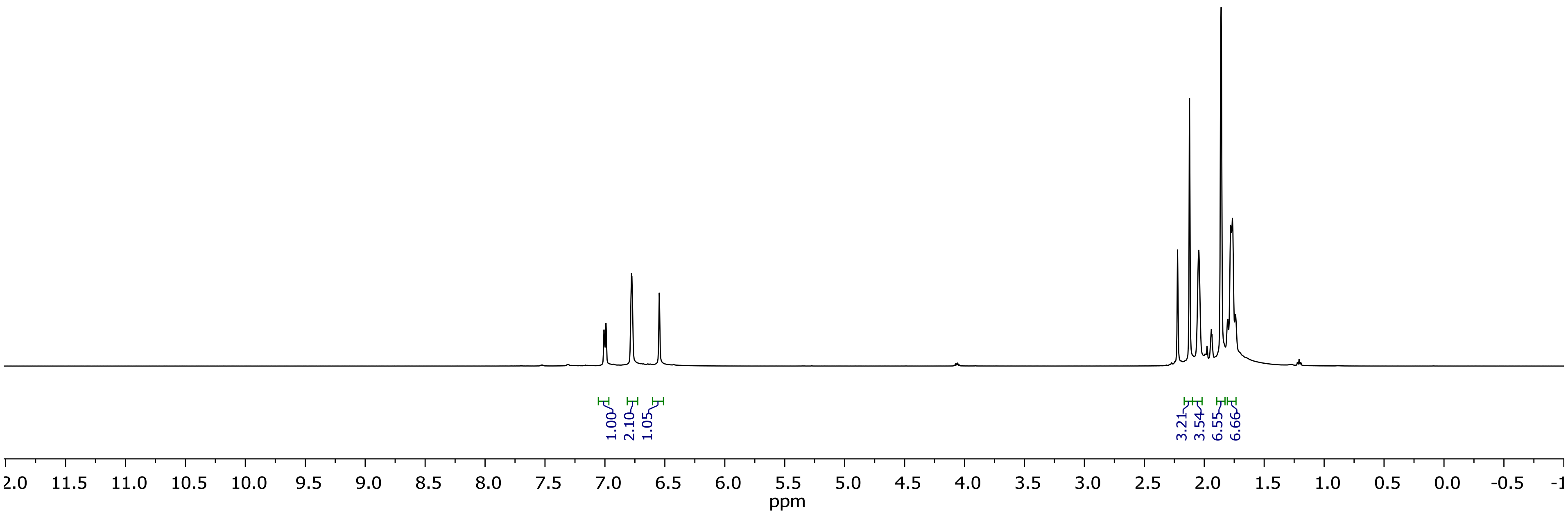

^{13}C NMR of 5-((3r,5r,7r)-adamantan-1-yl)-2-methylphenol (23)

CD_3CN, 25 °C

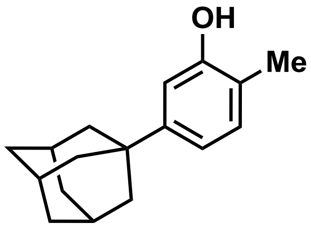

23

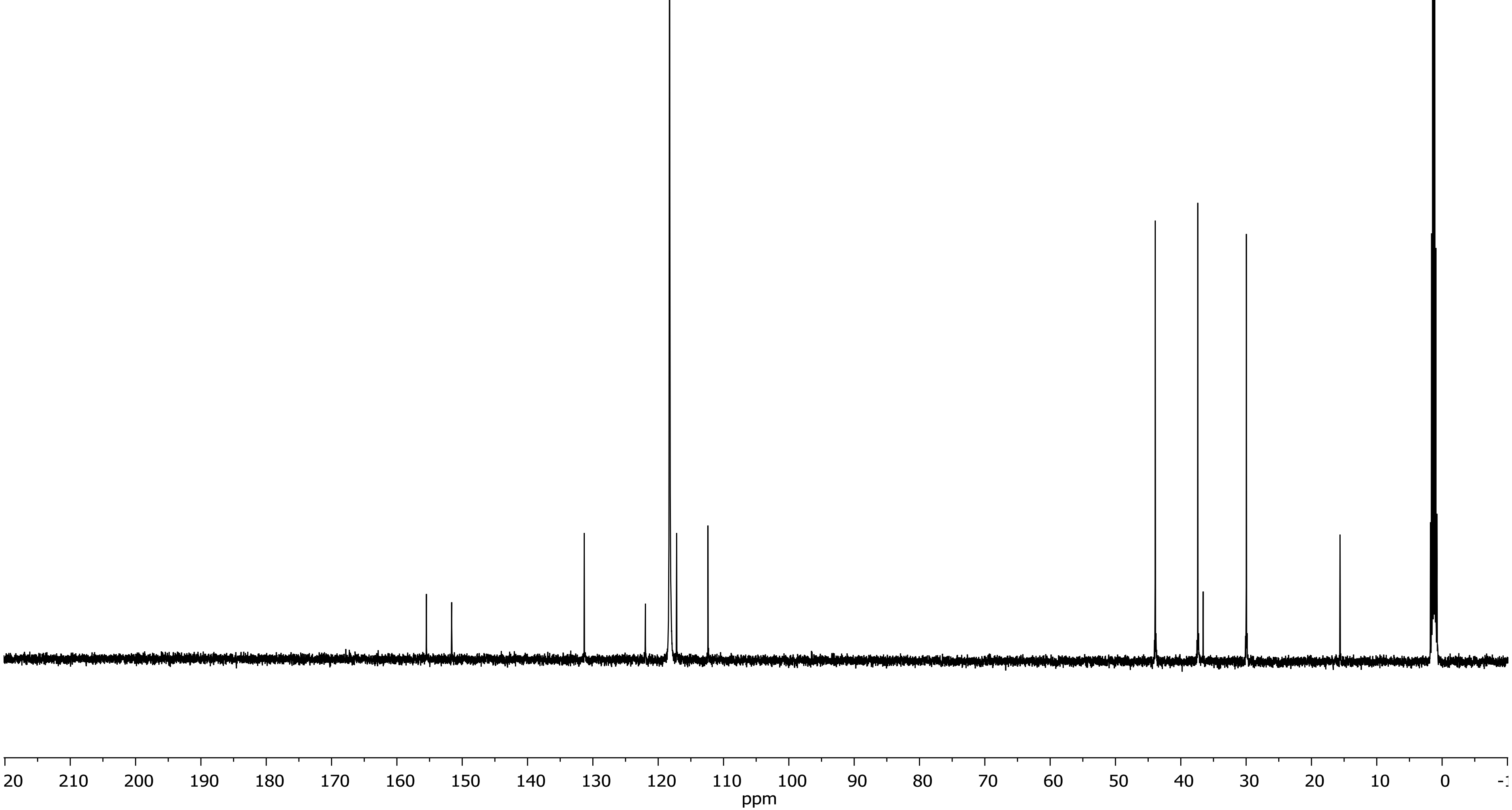

^{1}H NMR of 4'-hydroxy-[1,1'-biphenyl]-4-yl trifluoromethanesulfonate (24)

$CDCl_3$, 25 °C

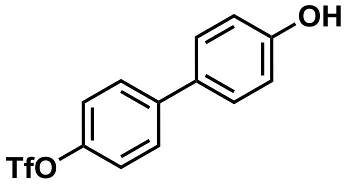

24

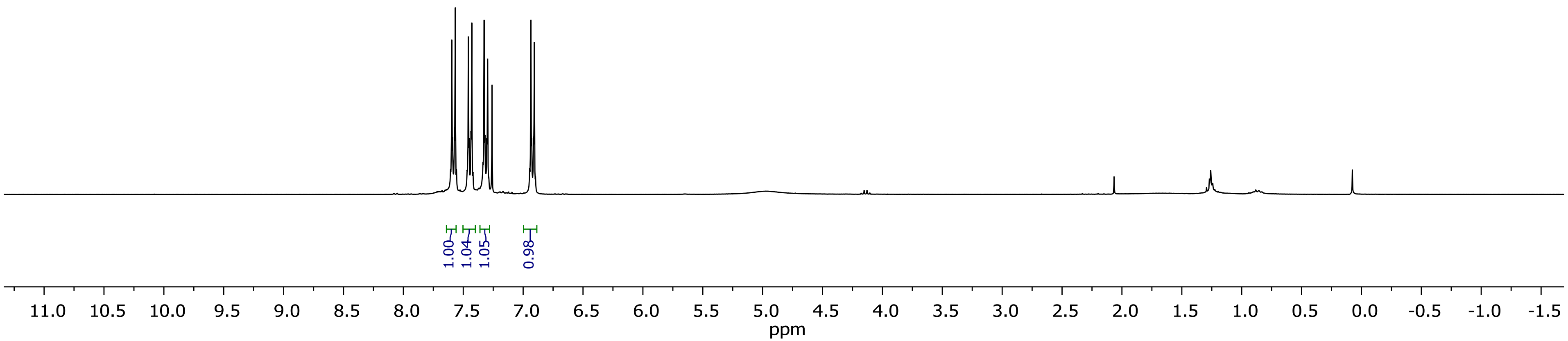

^{13}C NMR of 4'-hydroxy-[1,1'-biphenyl]-4-yl trifluoromethanesulfonate (24)

CDCl$_3$, 25 °C

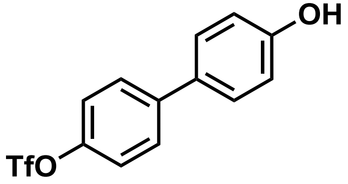

24

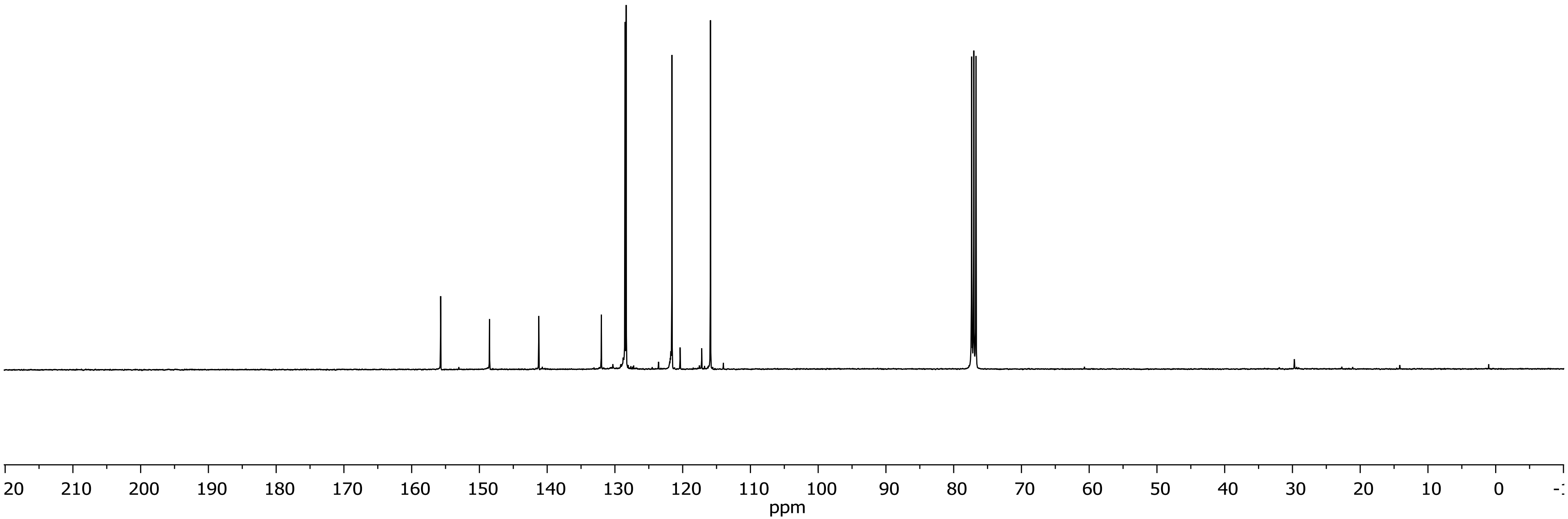

^{19}F NMR of 4'-hydroxy-[1,1'-biphenyl]-4-yl trifluoromethanesulfonate (24)

$CDCl_3$, 25 °C

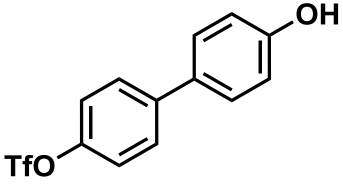

24

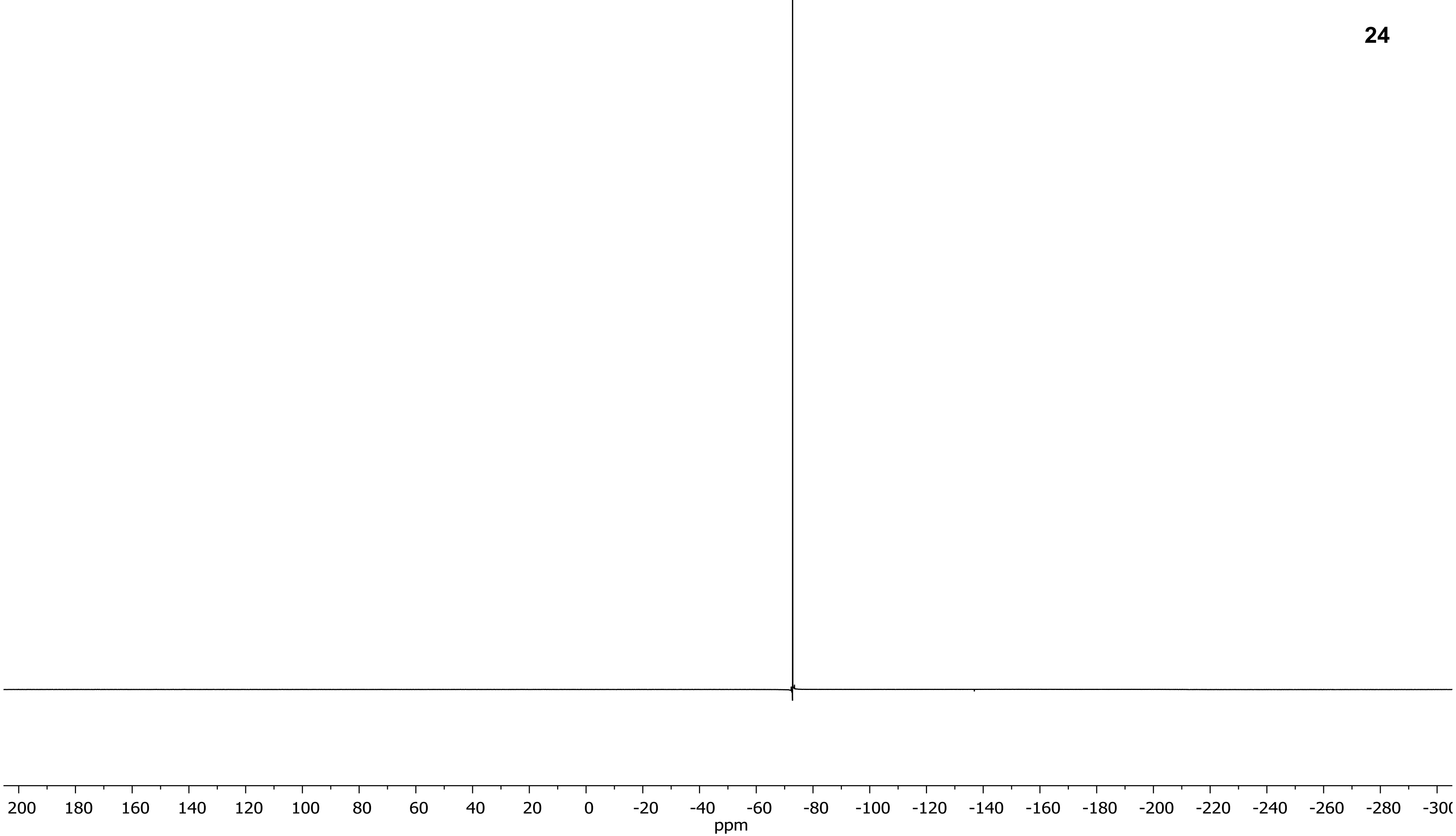

^{1}H NMR of diclofenac amide thianthrenium salt (25-TT)

CD_3CN, 25 °C

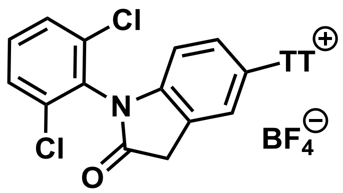

25-TT

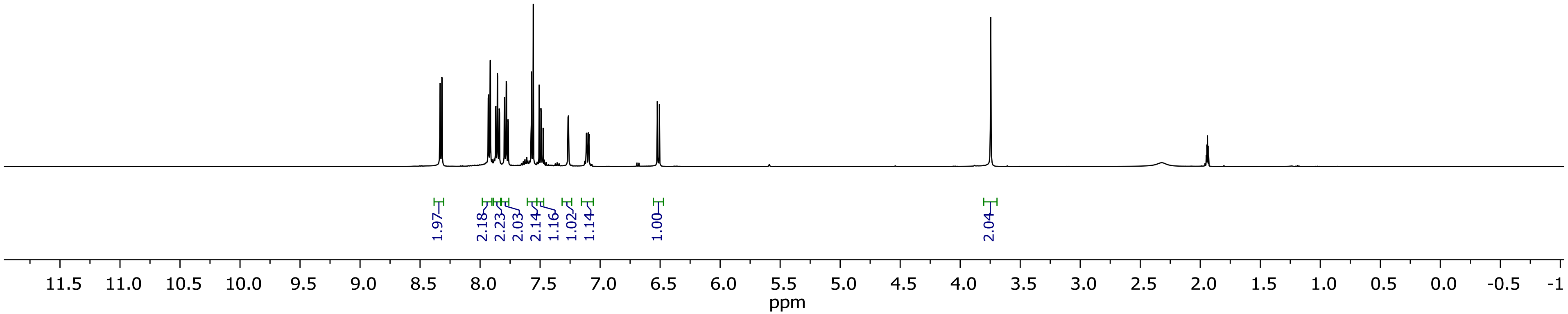

^{13}C NMR of diclofenac amide thianthrenium salt (25-TT)

CD_3CN, 25 °C

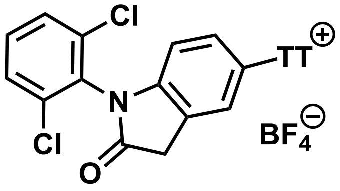

25-TT

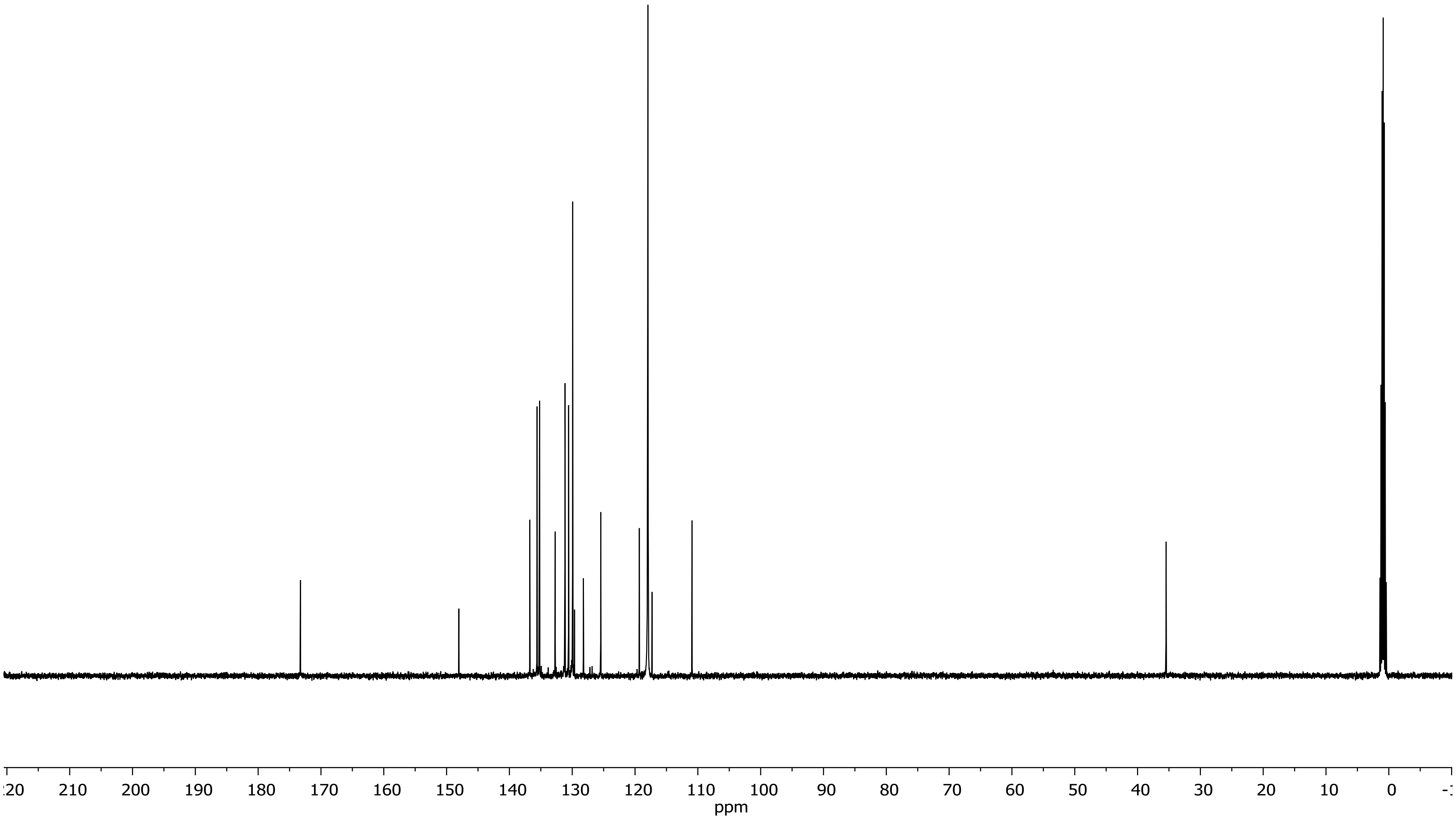

^{19}F NMR of diclofenac amide thianthrenium salt (25-TT)

CD_3CN, 25 °C

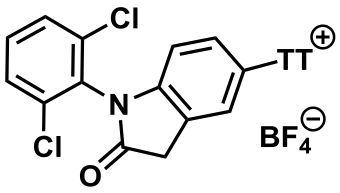

25-TT

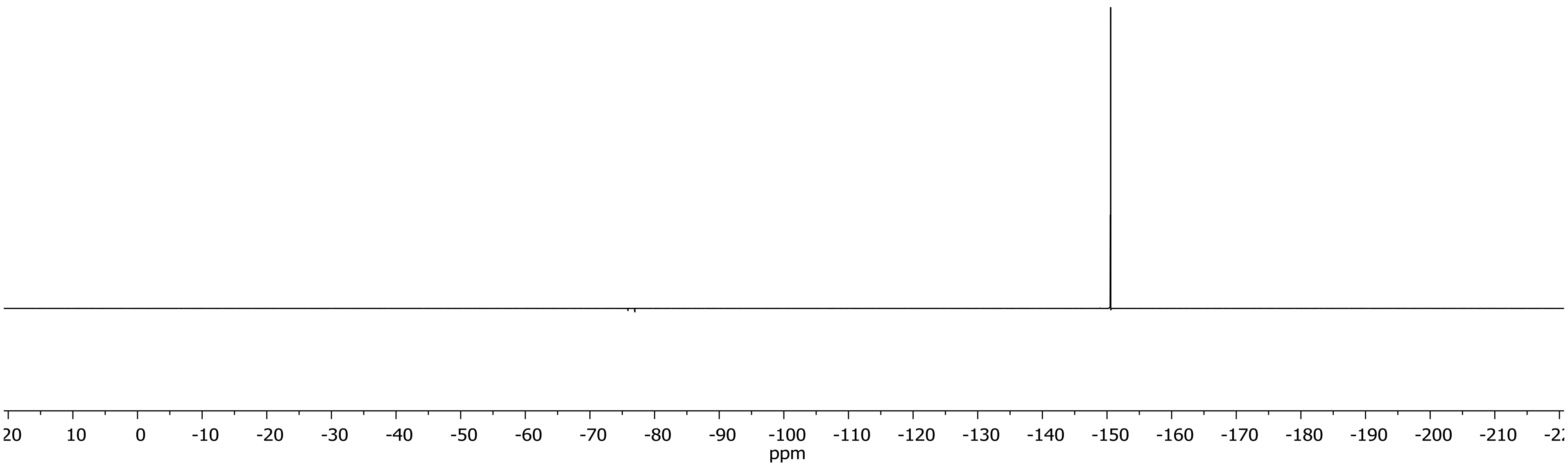

^{1}H NMR of hydroxy diclofenac amide (25)

CD_3CN, 25 °C

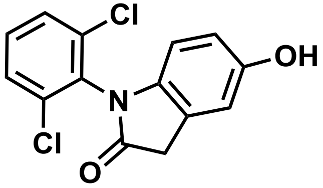

25

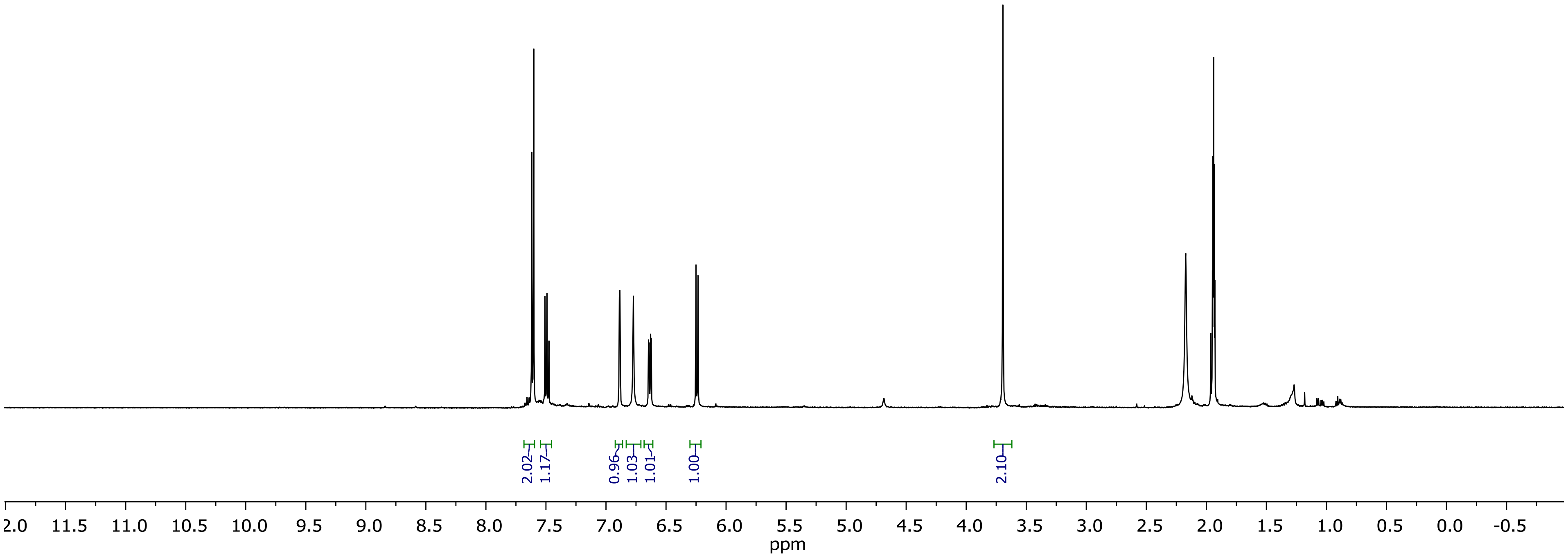

^{13}C NMR of hydroxy diclofenac amide (25)

CD_3CN, 25 °C

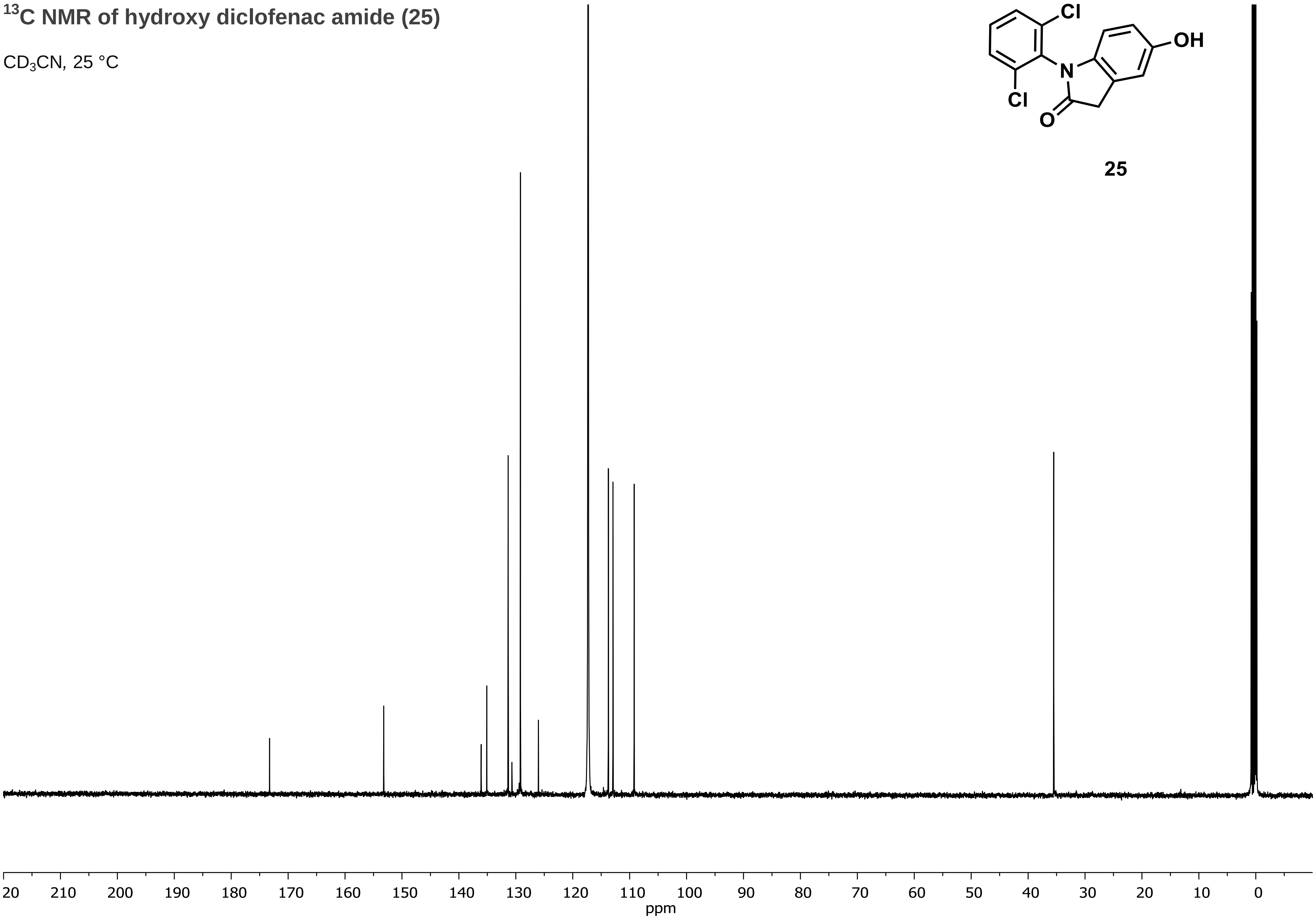

^{1}H NMR of hydroxy-pyriproxyfen (26)

CD_3CN, 25 °C

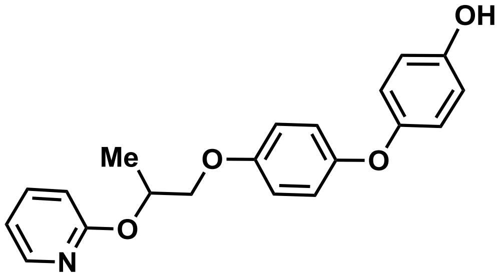

26

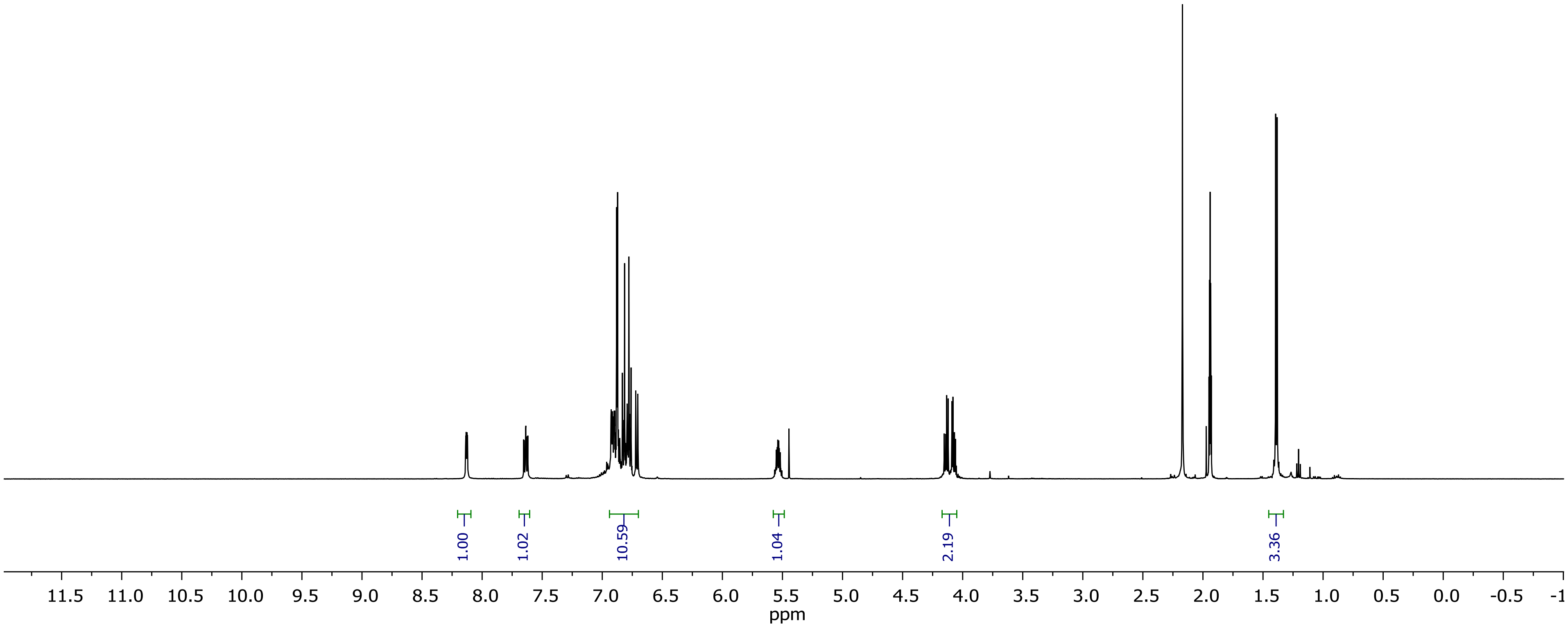

^{13}C NMR of hydroxy-pyriproxyfen (26)

$CDCl_3$, 25 °C

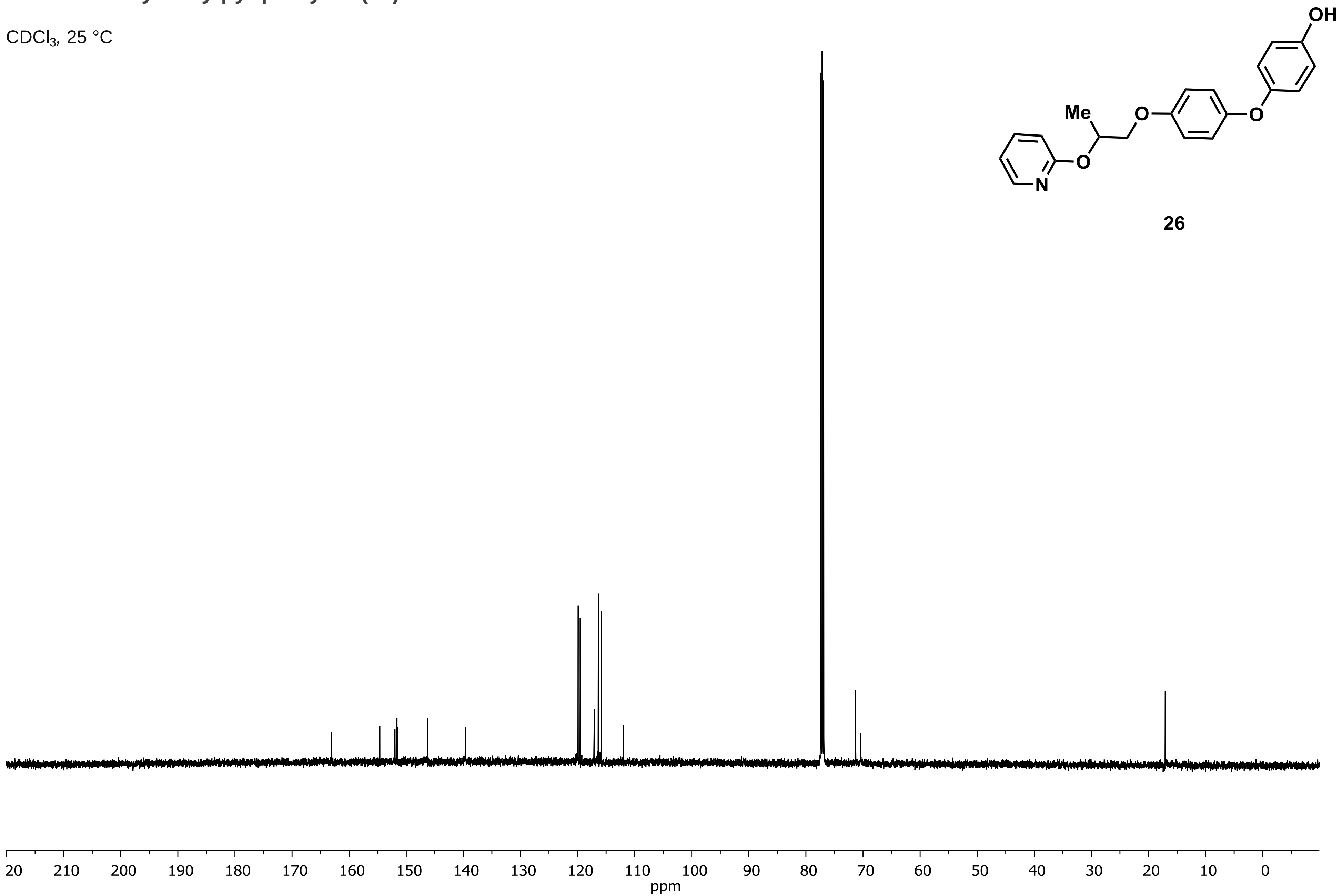

26

^{1}H NMR of 2-fluoro-6-phenoxybenzonitrile tetrafluorothianthrenium salt (27-TFT)

CD_3CN, 25 °C

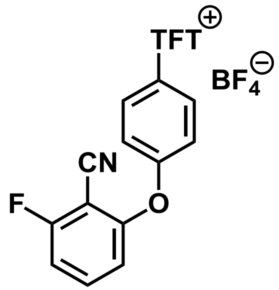

27-TFT

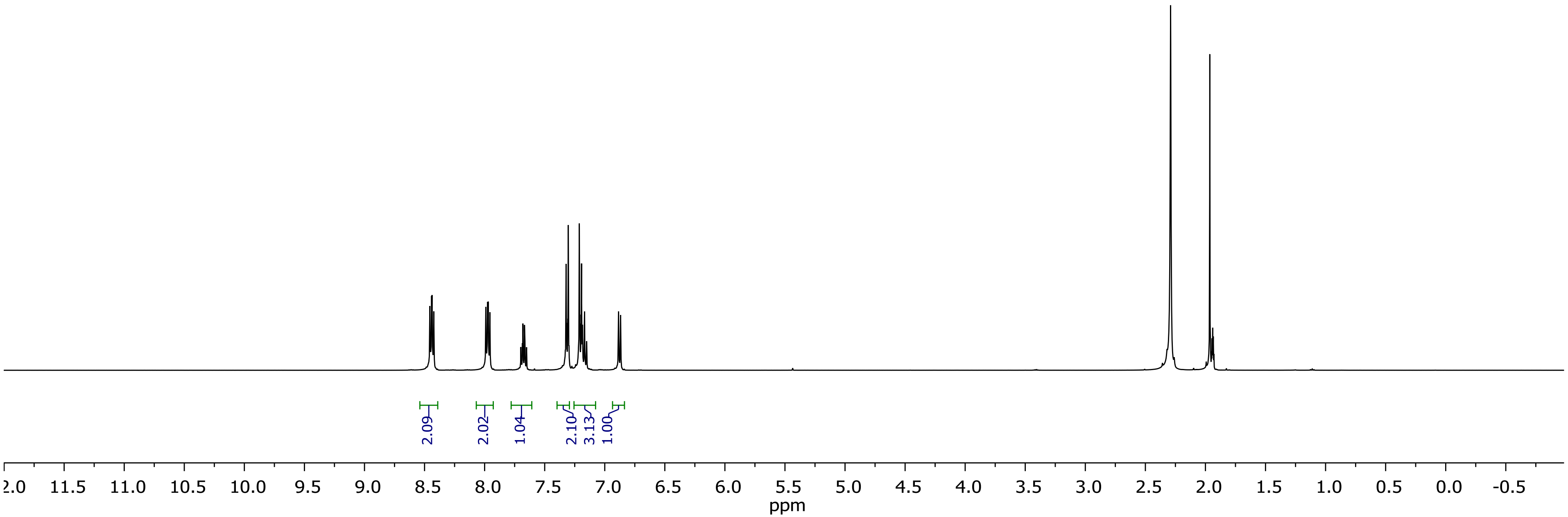

^{13}C NMR of 2-fluoro-6-phenoxybenzonitrile tetrafluorothianthrenium salt (27-TFT)

CD_3CN, 25 °C

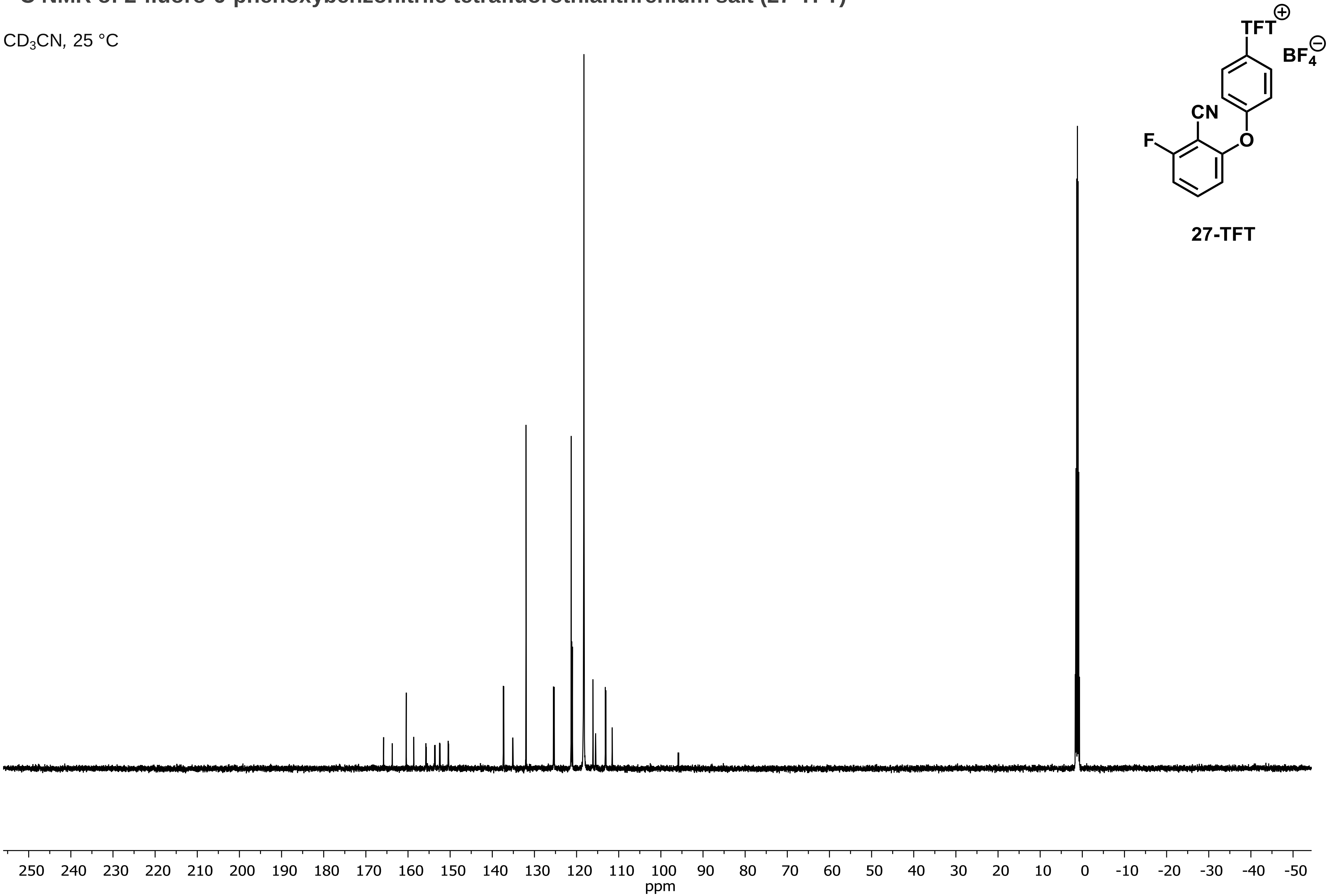

^{19}F NMR of 2-fluoro-6-phenoxybenzonitrile tetrafluorothianthrenium salt (27-TFT)

CD_3CN, 25 °C

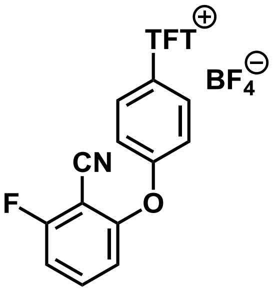

27-TFT

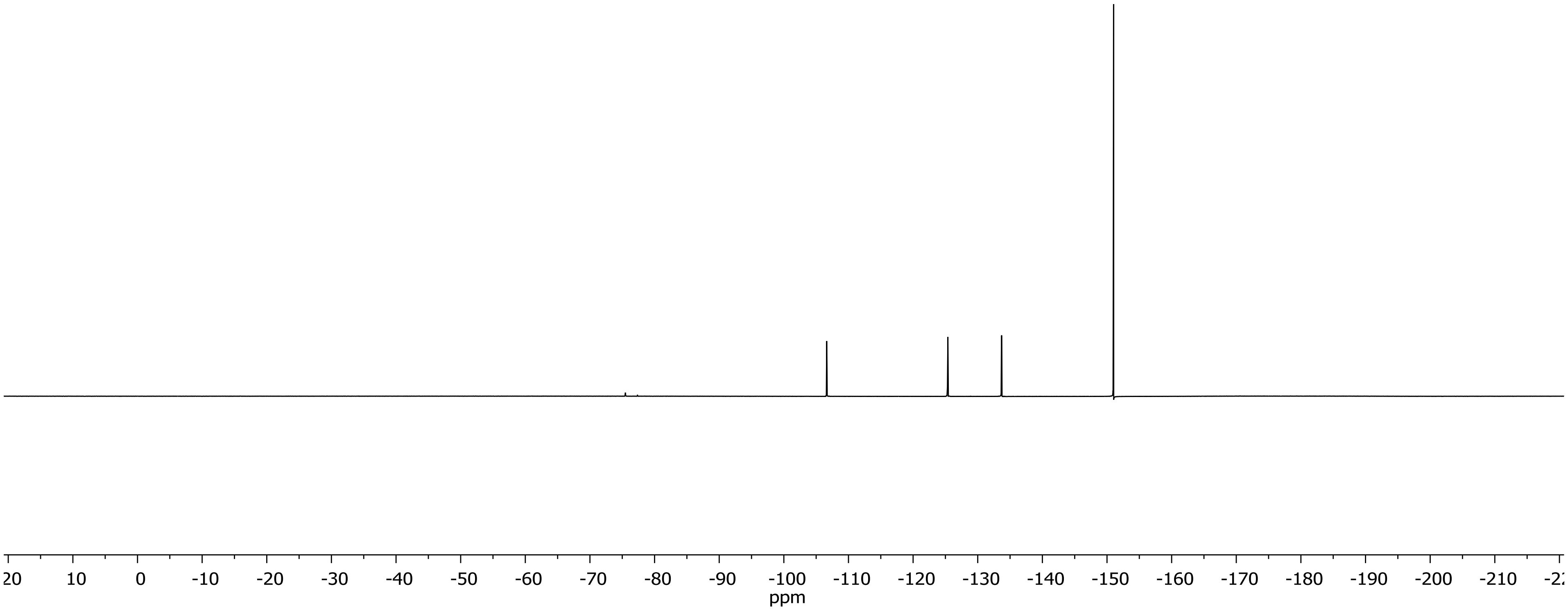

^{1}H NMR of 2-fluoro-6-(4-methoxyphenoxy)benzonitrile (27)

CD_2Cl_2, 25 °C

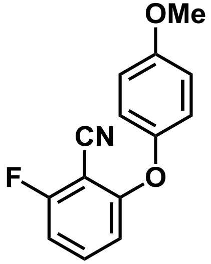

27

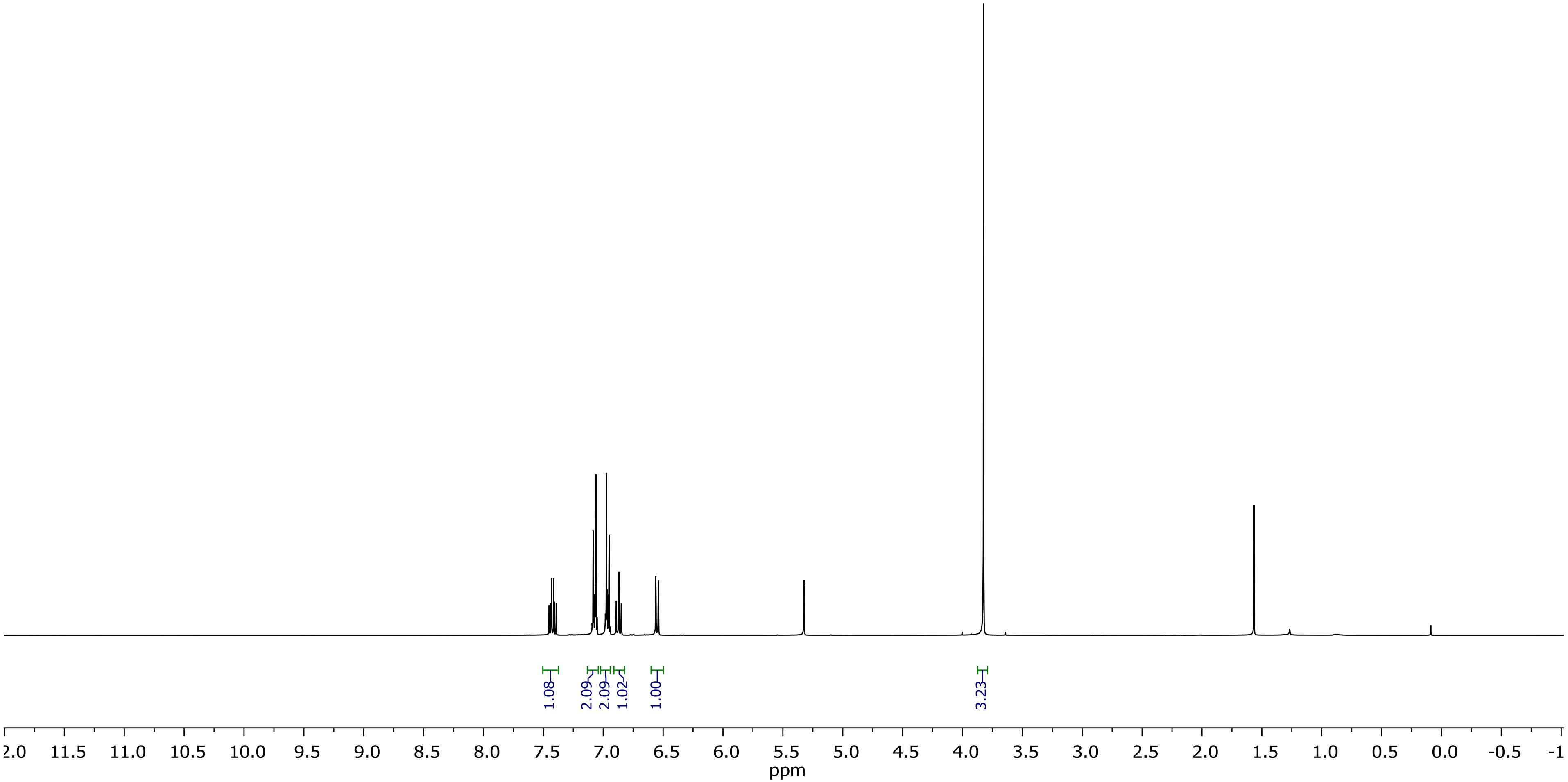

^{13}C NMR of 2-fluoro-6-(4-methoxyphenoxy)benzonitrile (27)

CD_2Cl_2, 25 °C

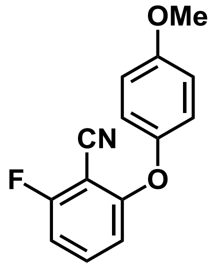

27

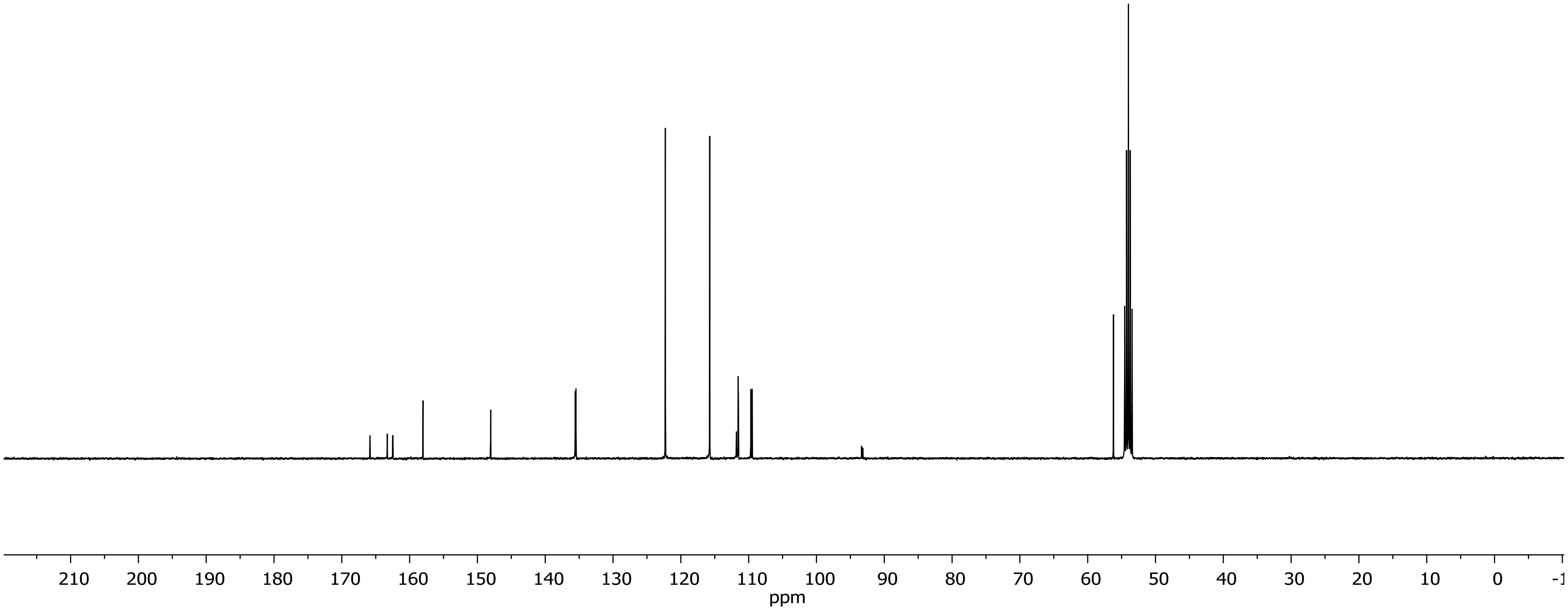

^{19}F NMR of 2-fluoro-6-(4-methoxyphenoxy)benzonitrile (27)

CD_2Cl_2, 25 °C

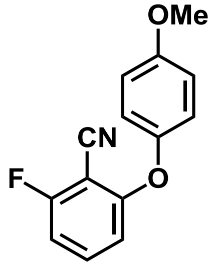

27

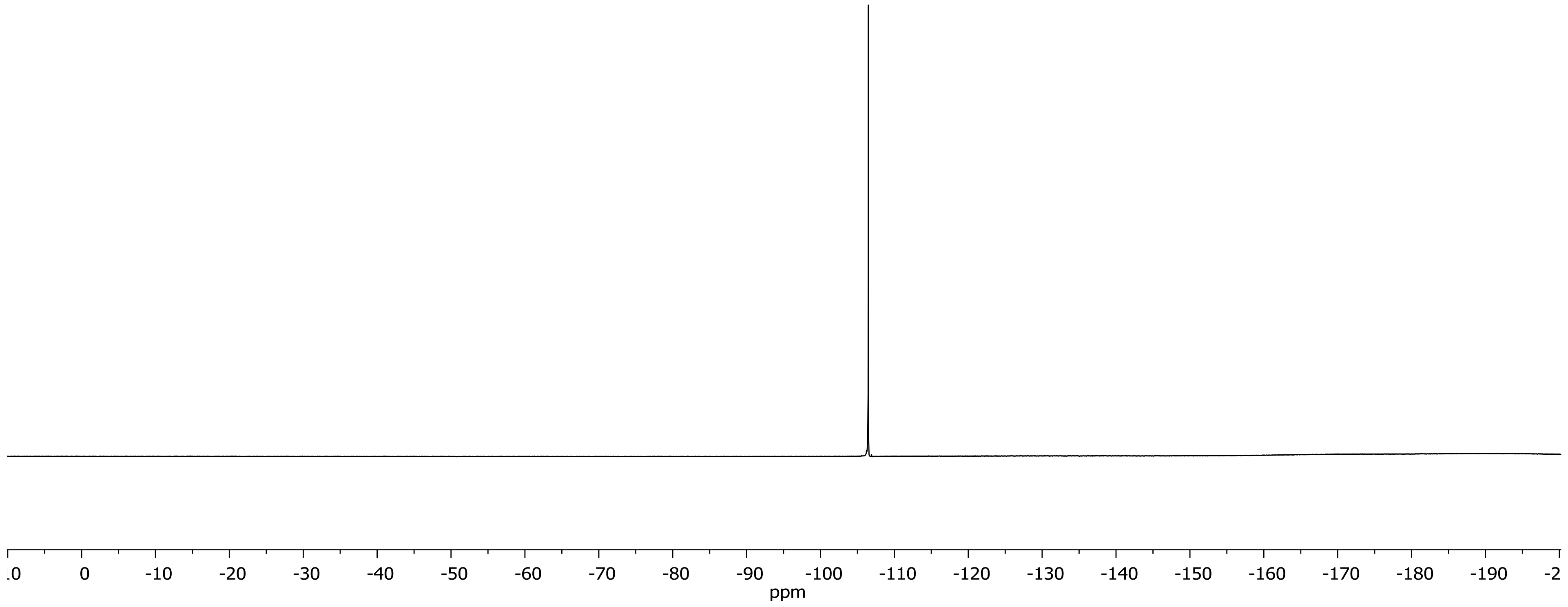

^{1}H NMR of 1-ethyl-4-phenoxybenzene (28)

$CDCl_3$, 25 °C

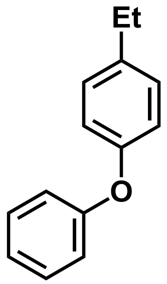

28

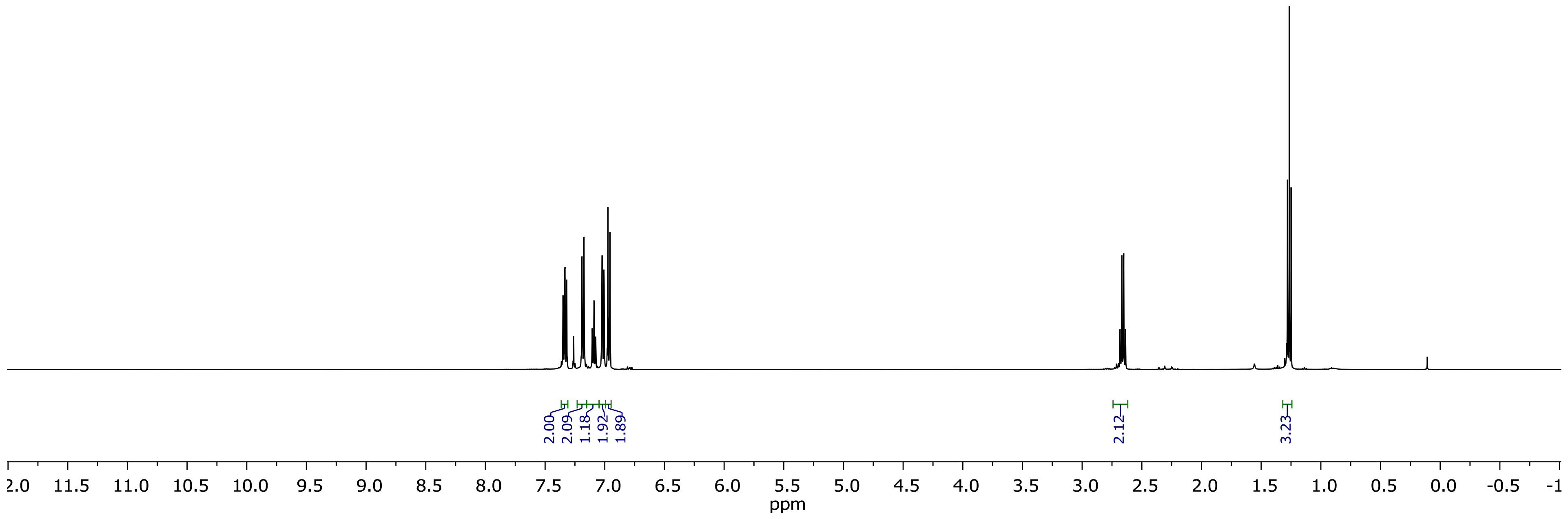

^{13}C NMR of 1-ethyl-4-phenoxybenzene (28)

CD_3CN, 25 °C

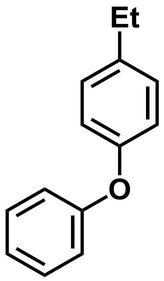

28

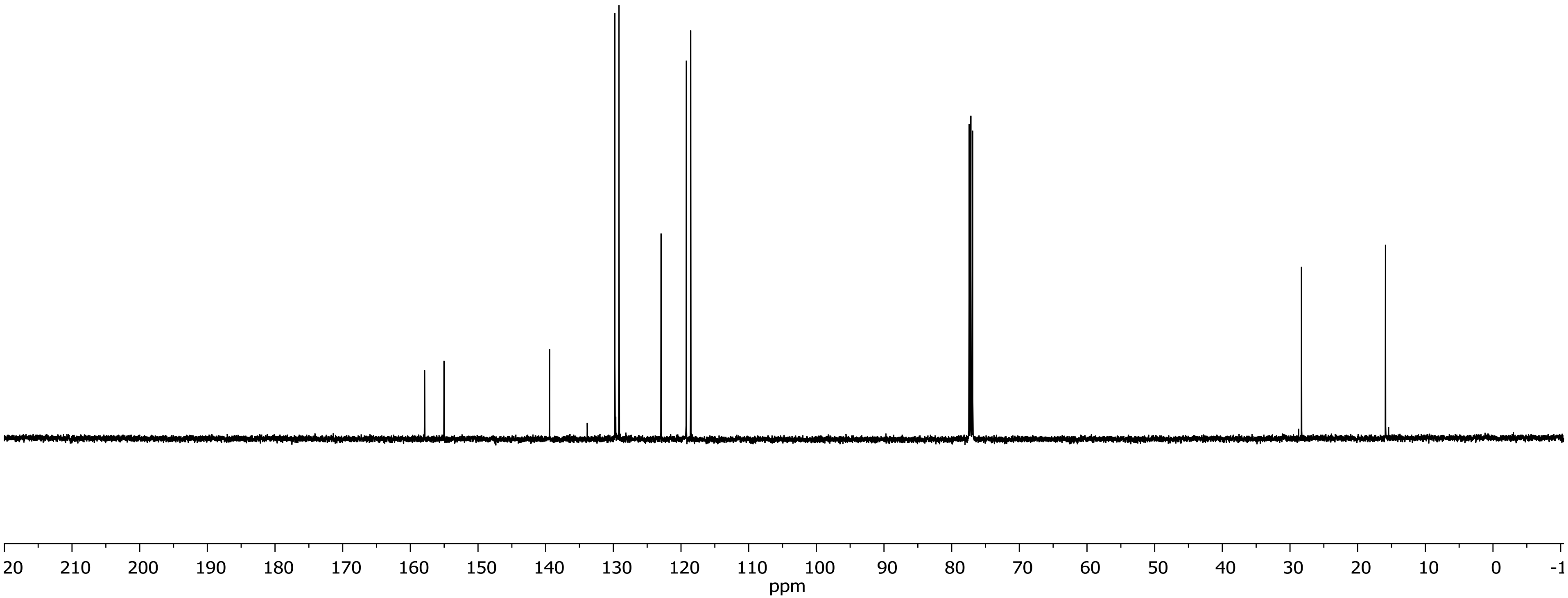

^{1}H NMR of *tert*-butyl 3-(4-ethylphenoxy)azetidine-1-carboxylate (29)

CD_3CN, 25 °C

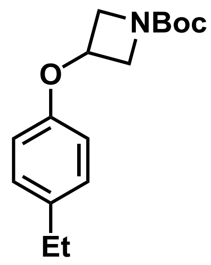

29

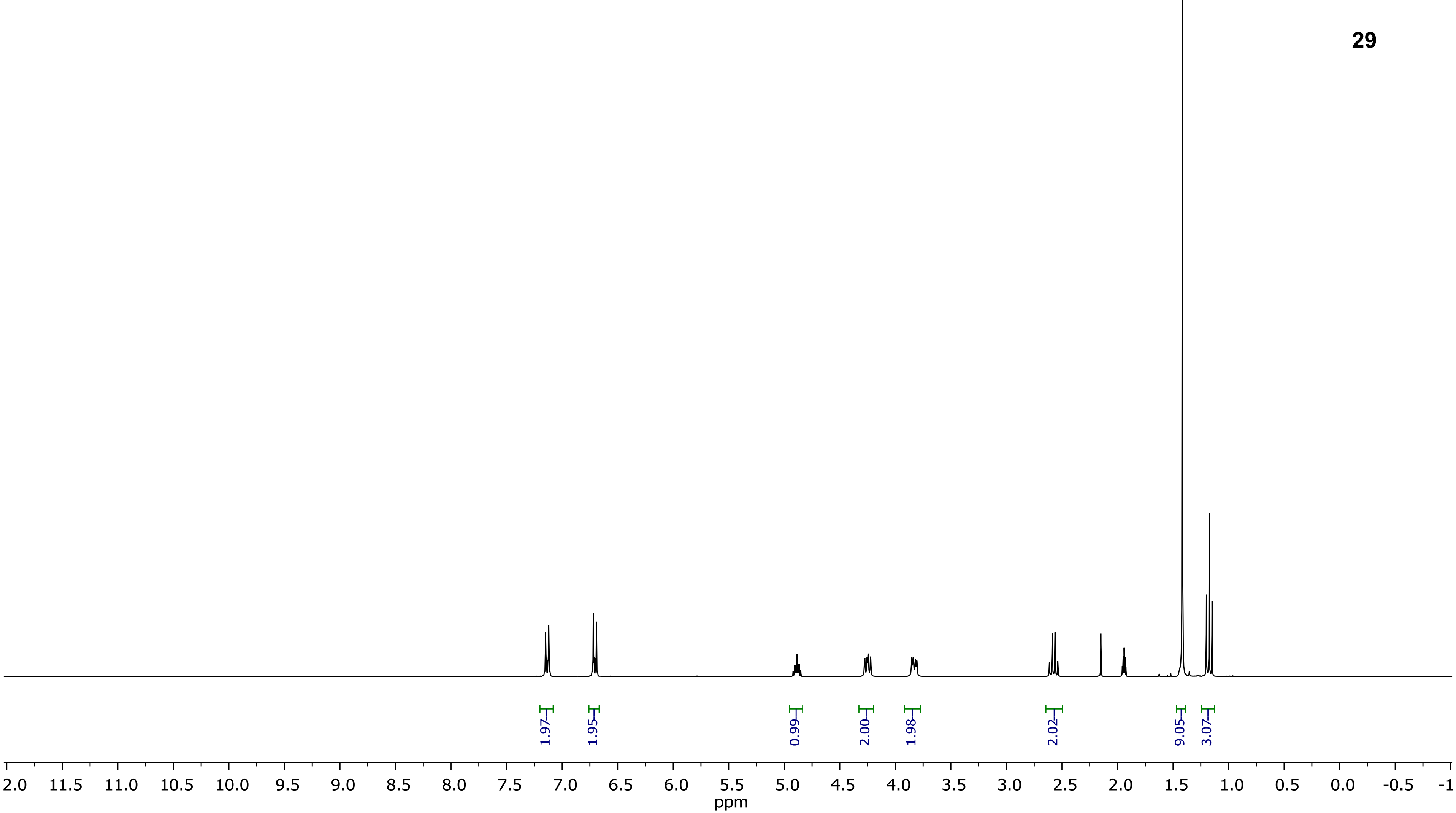

^{13}C NMR of *tert*-butyl 3-(4-ethylphenoxy)azetidine-1-carboxylate (29)

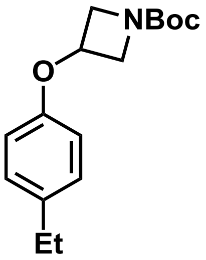

29

CD_3CN, 25 °C

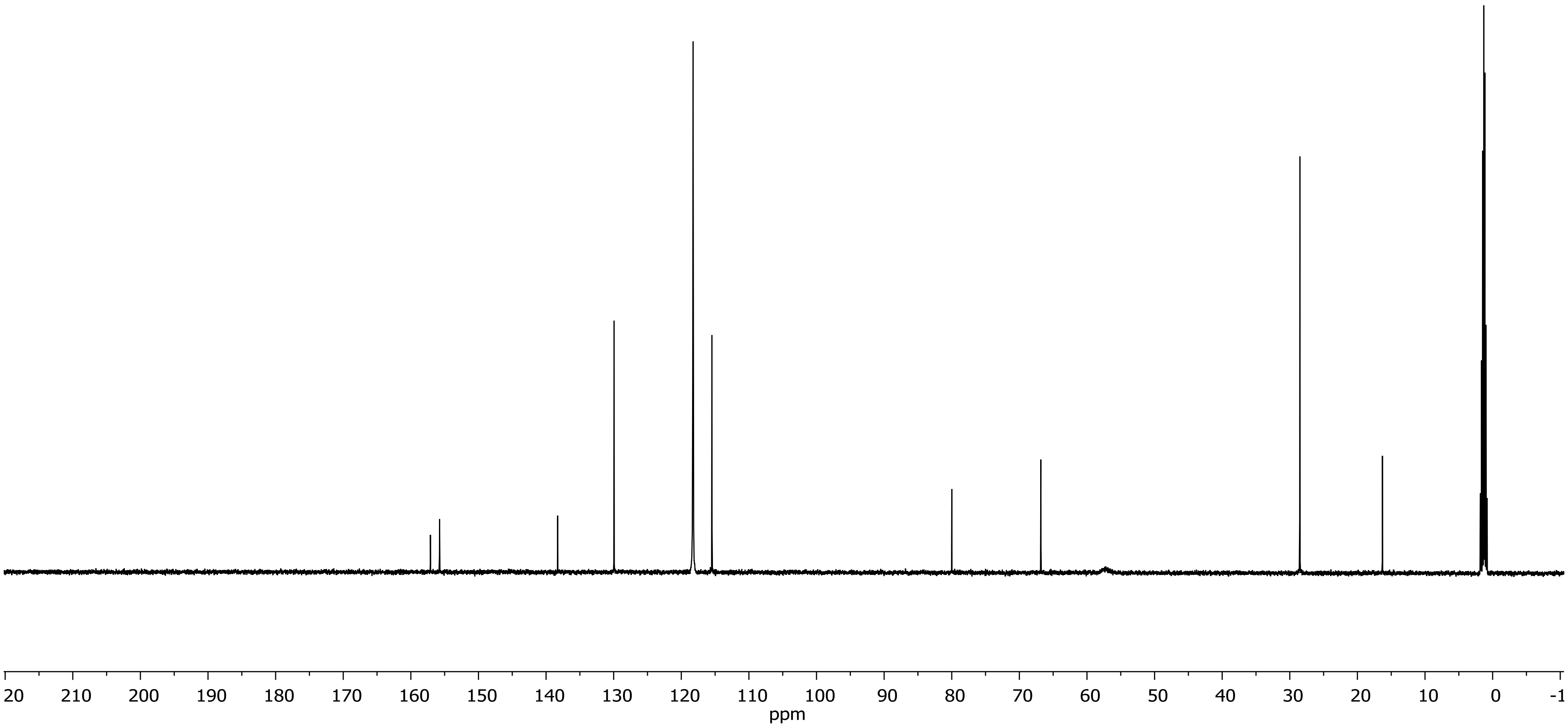

^{1}H NMR of 4-ethylphenyl thiophene-2-carboxylate (29-B)

$CDCl_3$, 25 °C

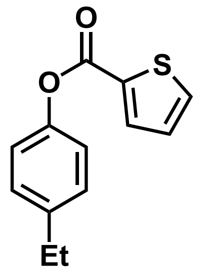

29-B

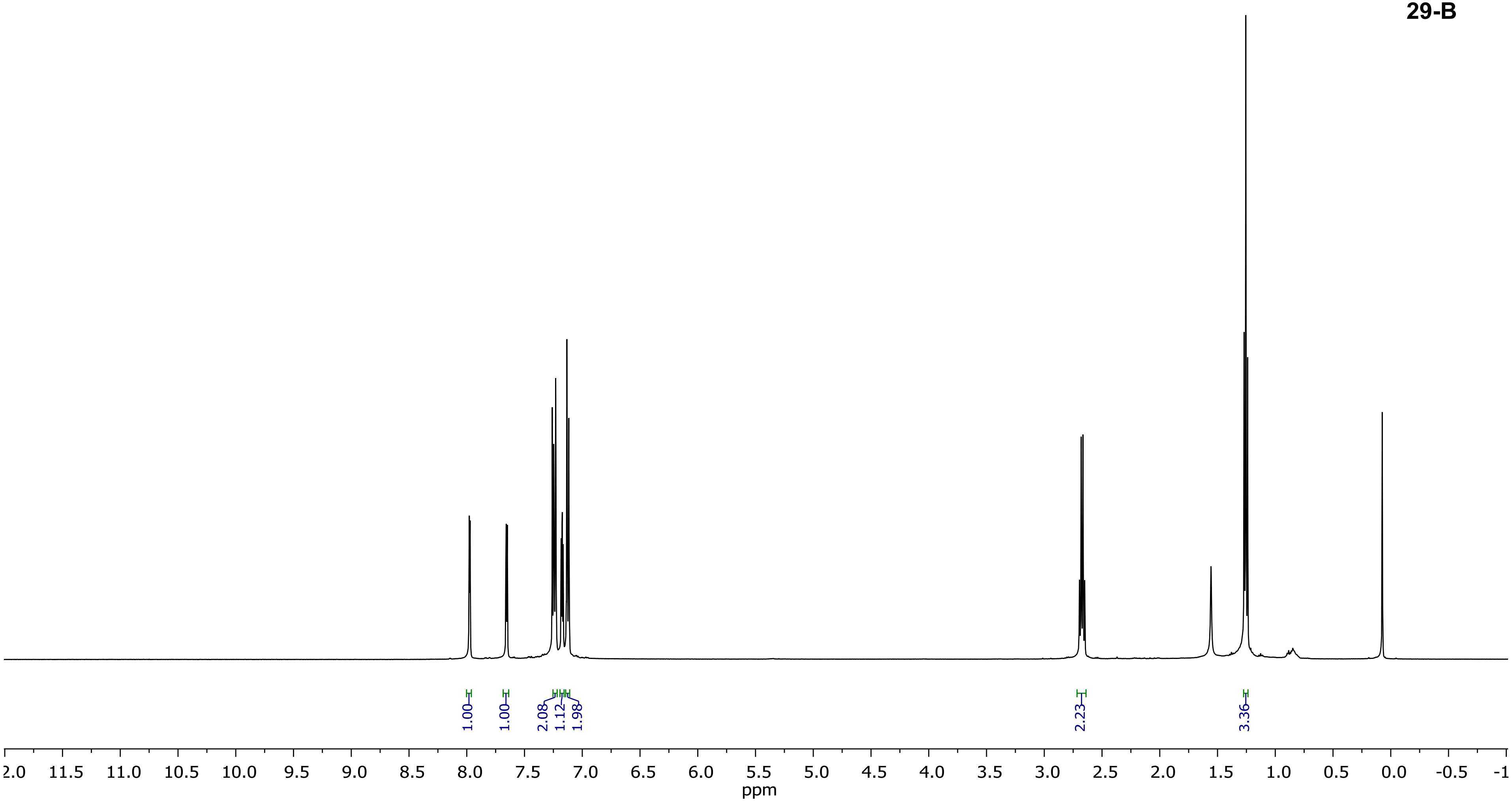

^{13}C NMR of 4-ethylphenyl thiophene-2-carboxylate (29-B)

$CDCl_3$, 25 °C

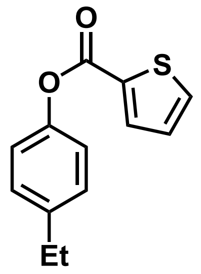

29-B

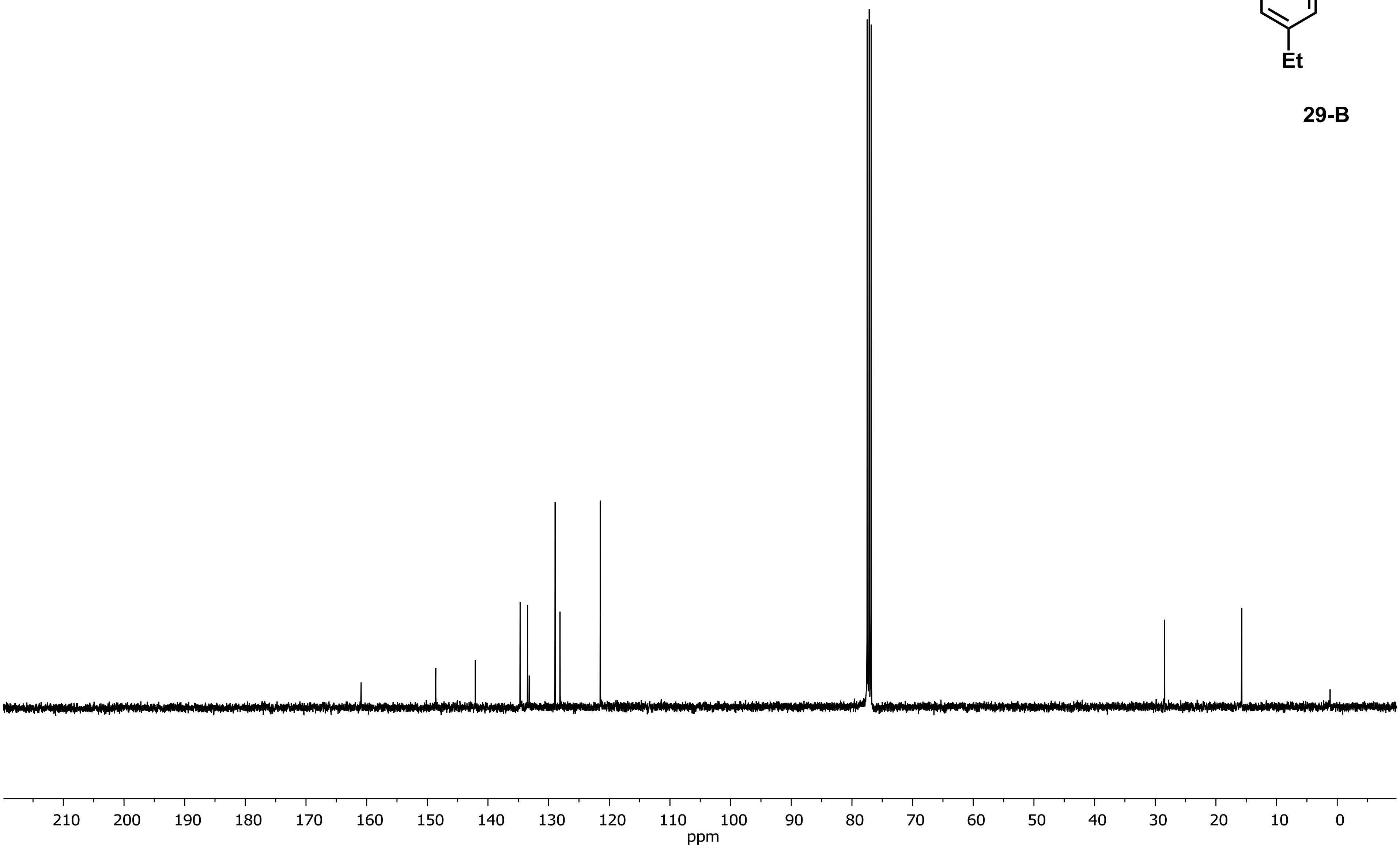

^{1}H NMR of 4,4'-oxybis(ethylbenzene) (29-C)

$CDCl_3$, 25 °C

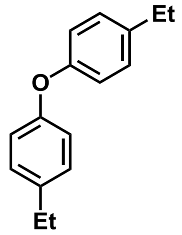

29-C

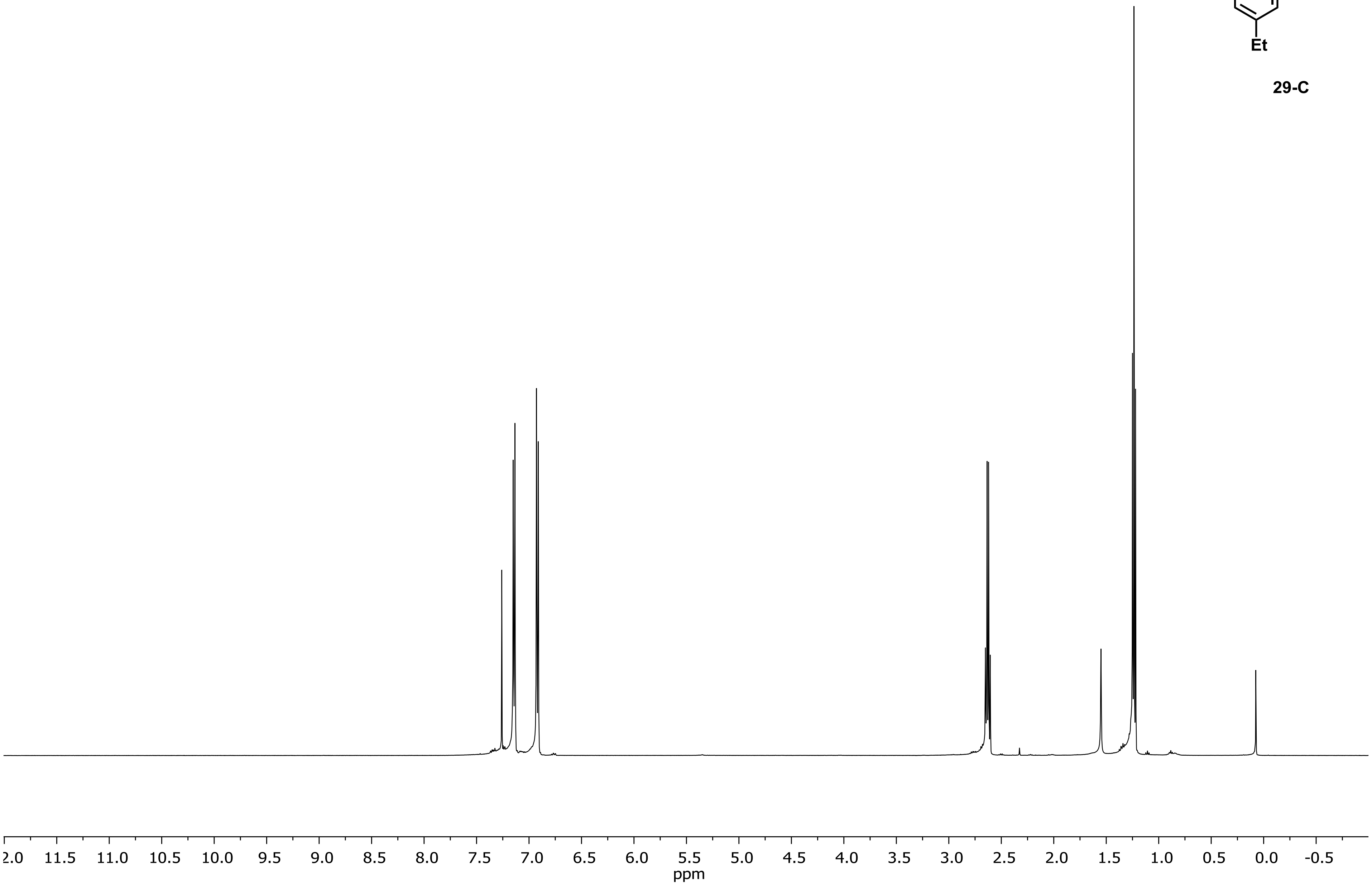

^{13}C NMR of 4,4'-oxybis(ethylbenzene) (29-C)

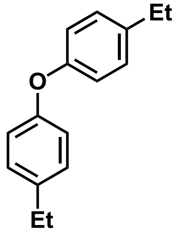

29-C

$CDCl_3$, 25 °C

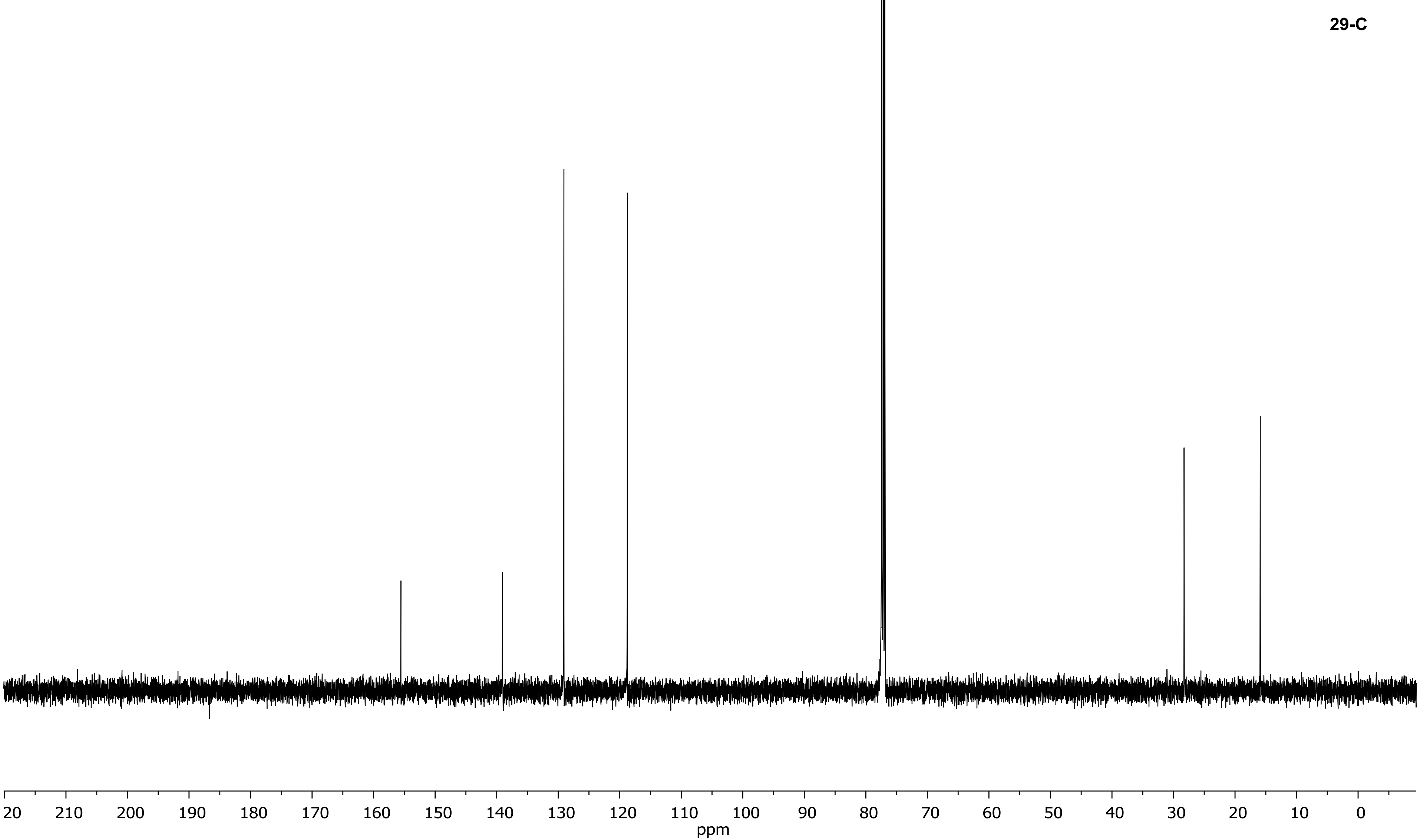

^{1}H NMR of *trans*-2-(4-ethylphenoxy)cyclohexan-1-ol (30)

CD_3CN, 25 °C

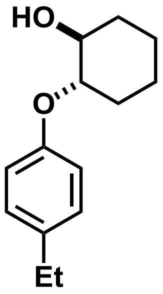

(±)30

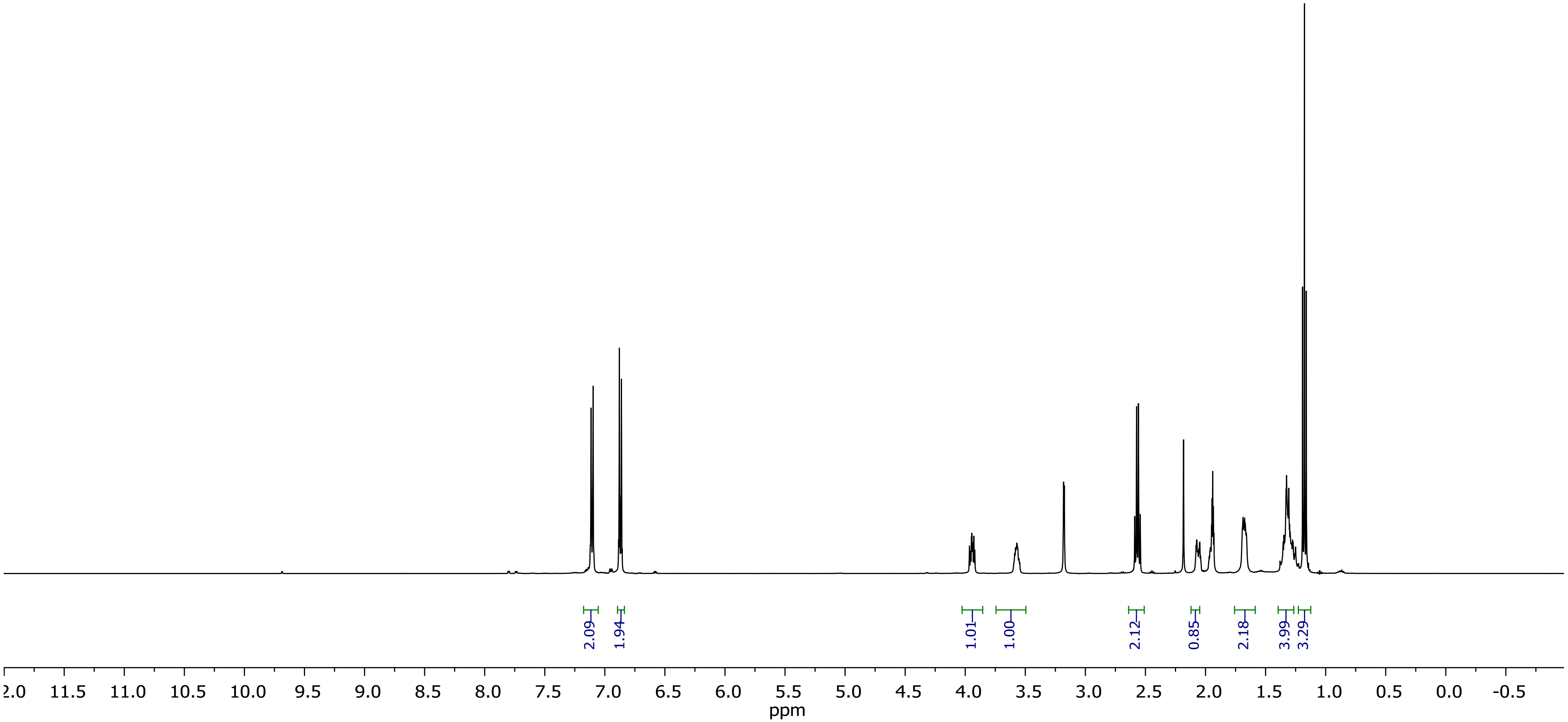

^{13}C NMR of *trans*-2-(4-ethylphenoxy)cyclohexan-1-ol (30)

CD_3CN, 25 °C

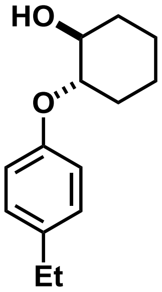

(±)30

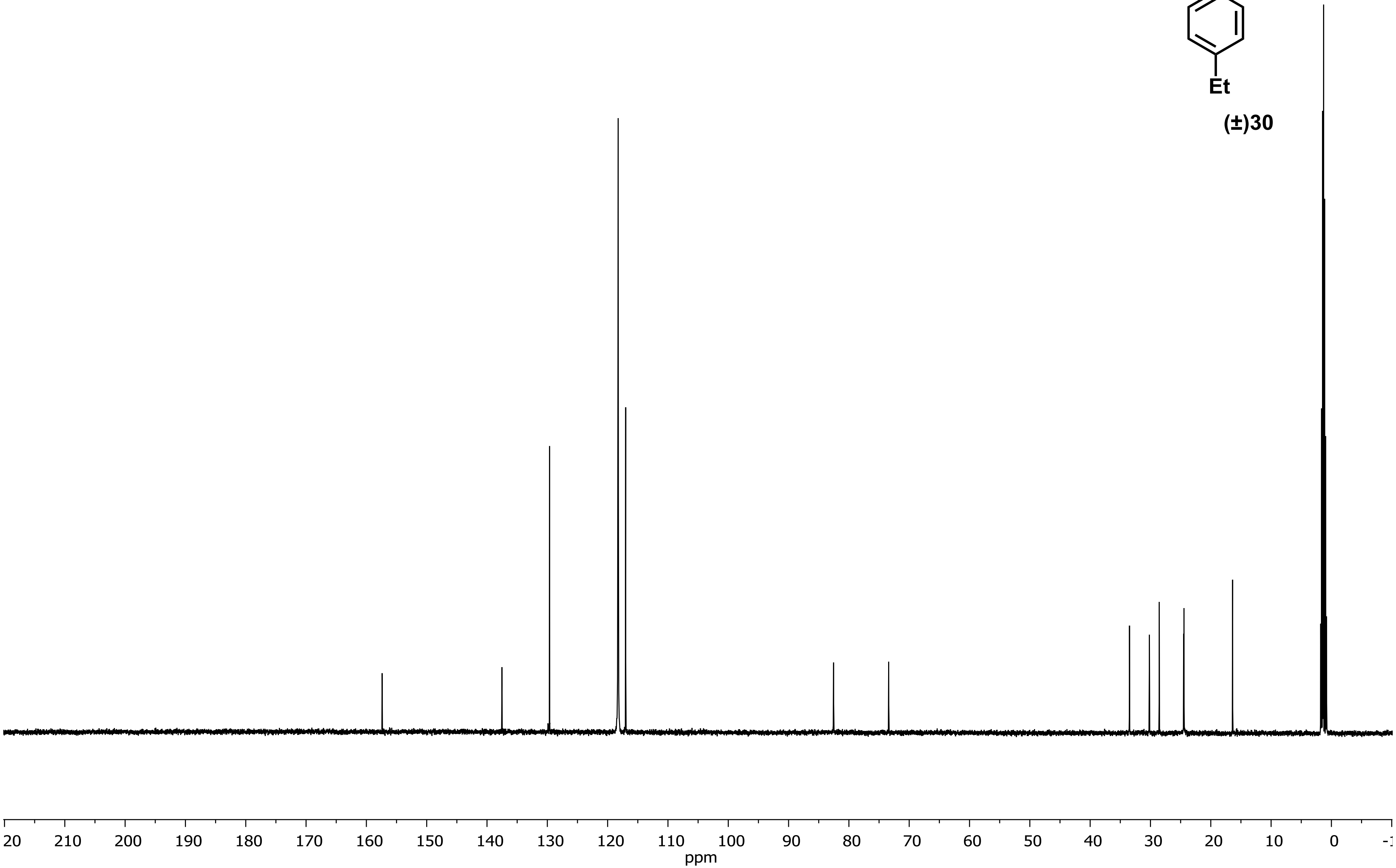

^{1}H NMR of 2-fluoro-6-(4-(methoxy-d$_3$)phenoxy)benzonitrile (31)

$CDCl_3$, 25 °C

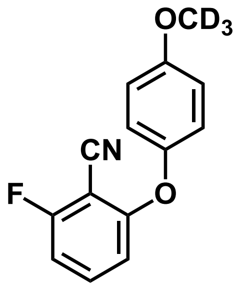

31

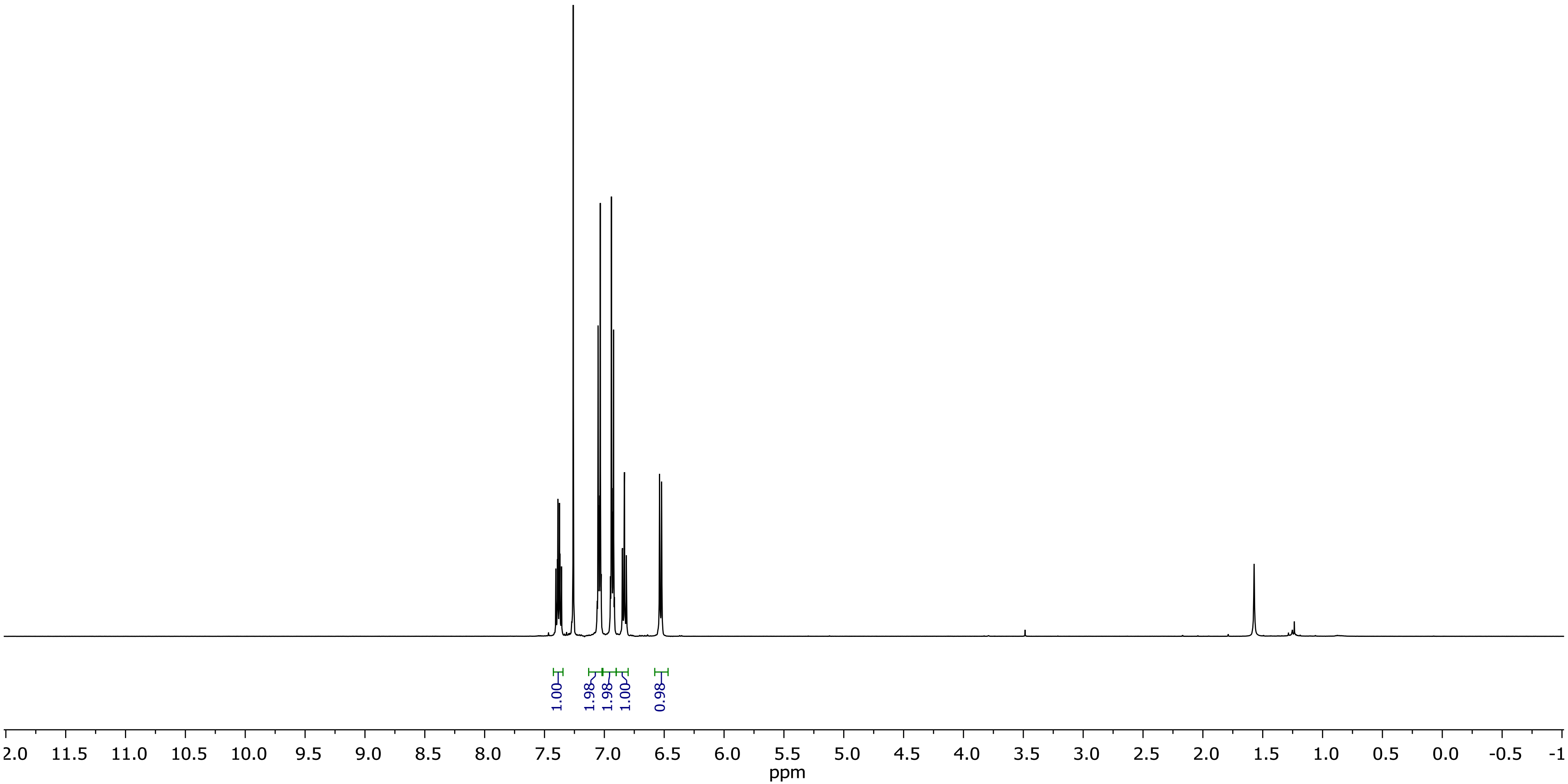

^{13}C NMR of 2-fluoro-6-(4-(methoxy-d$_3$)phenoxy)benzonitrile (31)

$CDCl_3$, 25 °C

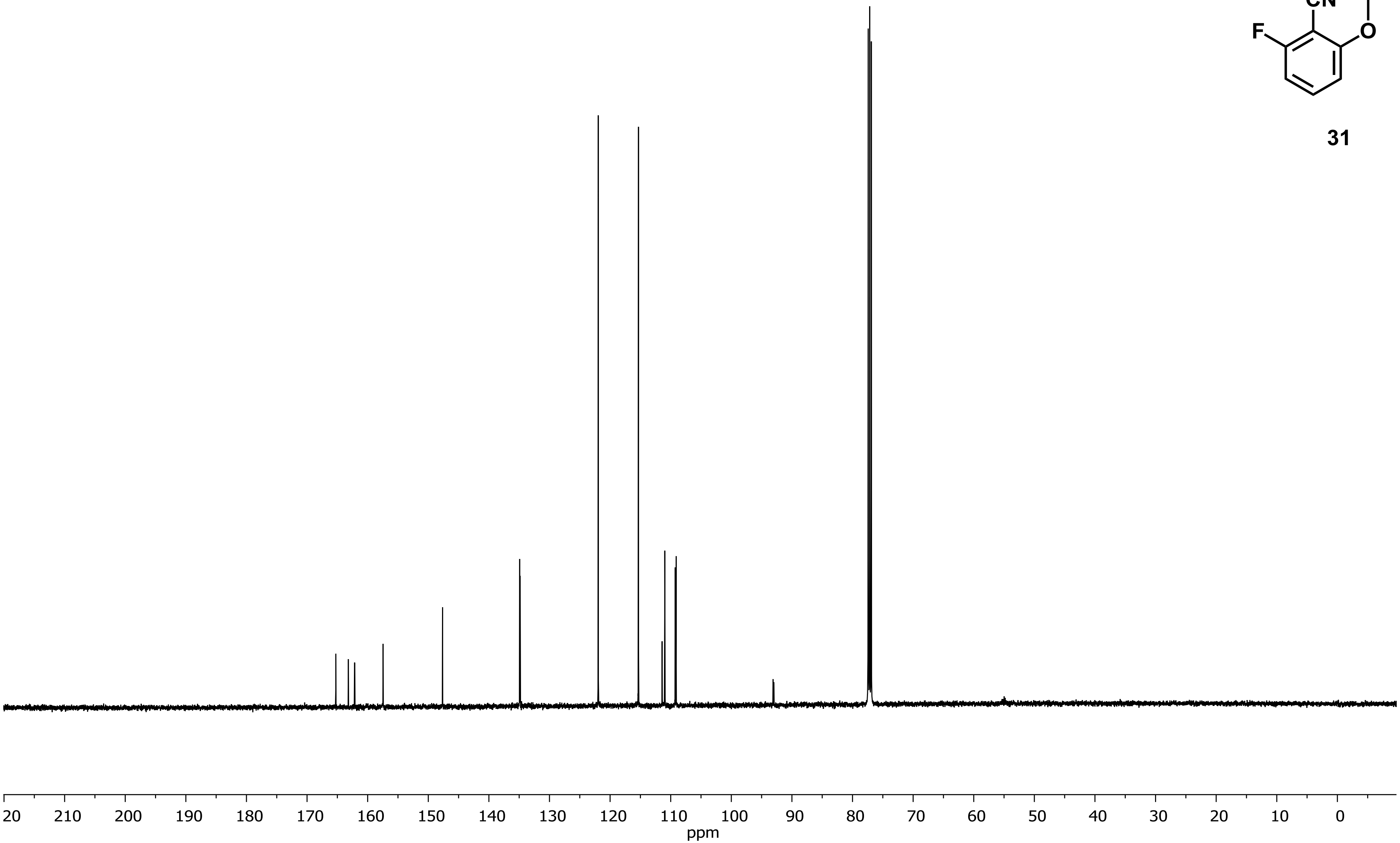

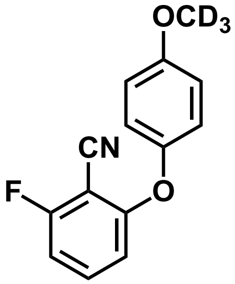

31

^{19}F NMR of 2-fluoro-6-(4-(methoxy-d$_3$)phenoxy)benzonitrile (31)

$CDCl_3$, 25 °C

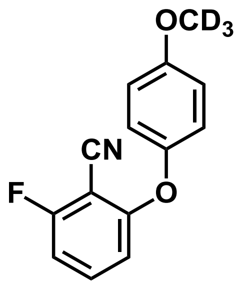

31

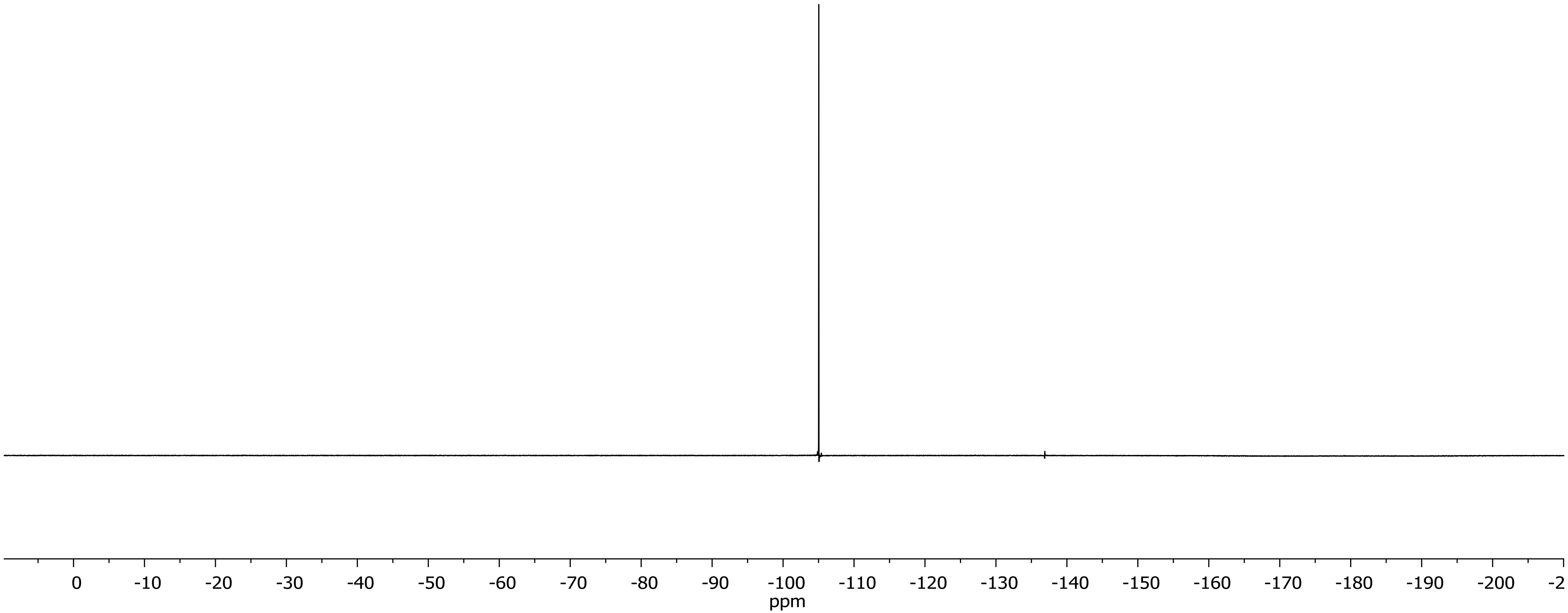

^{1}H NMR of 1-cyclobutoxy-4-ethylbenzene (32)

$CDCl_3$, 25 °C

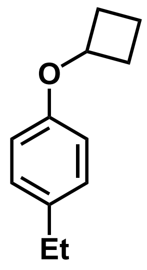

32

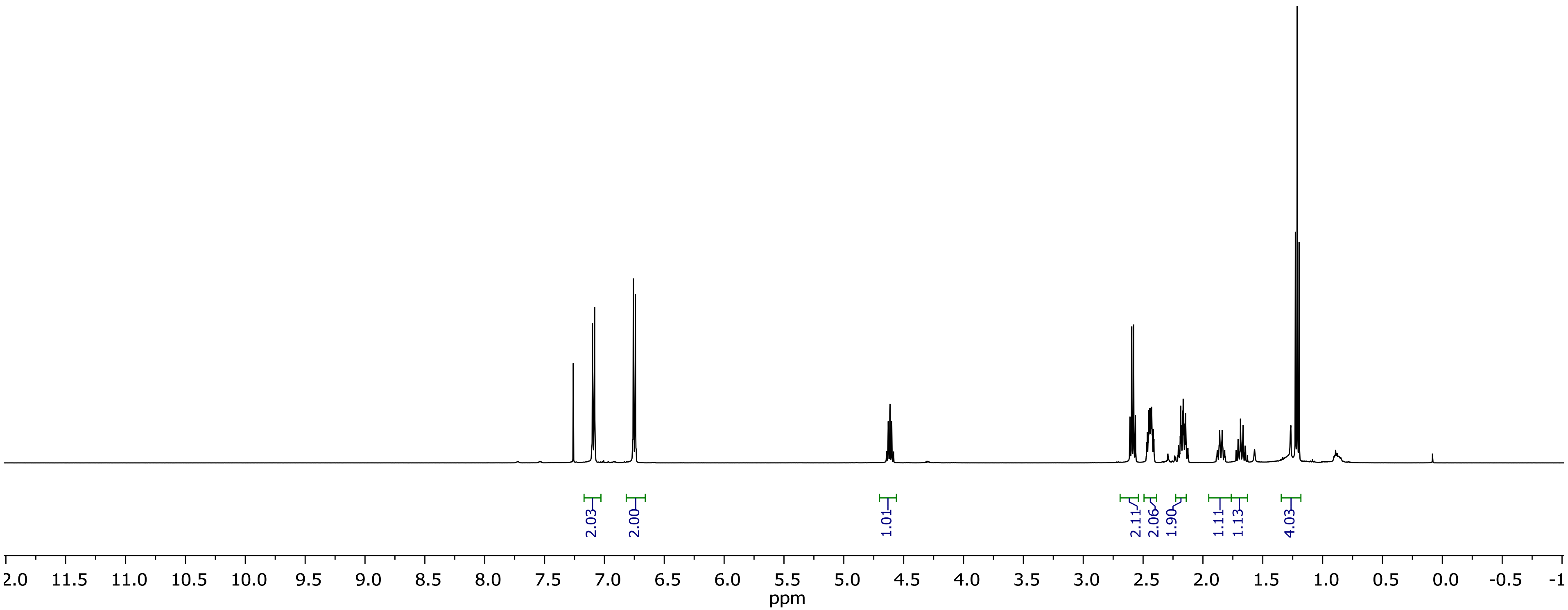

^{13}C NMR of 1-cyclobutoxy-4-ethylbenzene (32)

$CDCl_3$, 25 °C

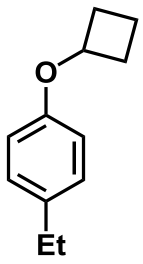

32

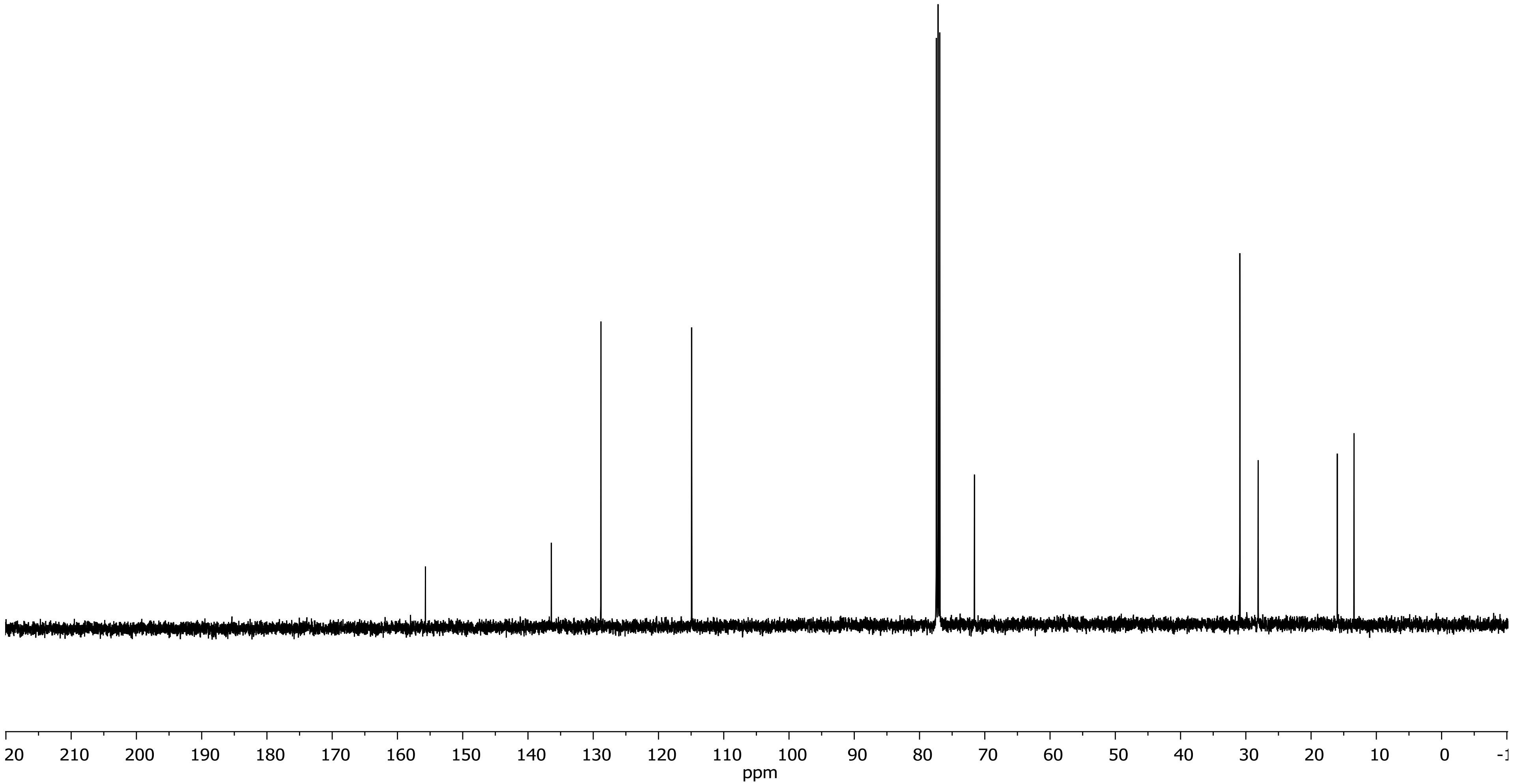

^{1}H NMR of 3-(4-ethylphenoxy)oxetane (33)

$CDCl_3$, 25 °C

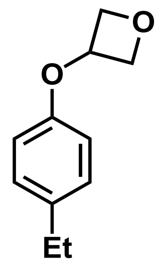

33

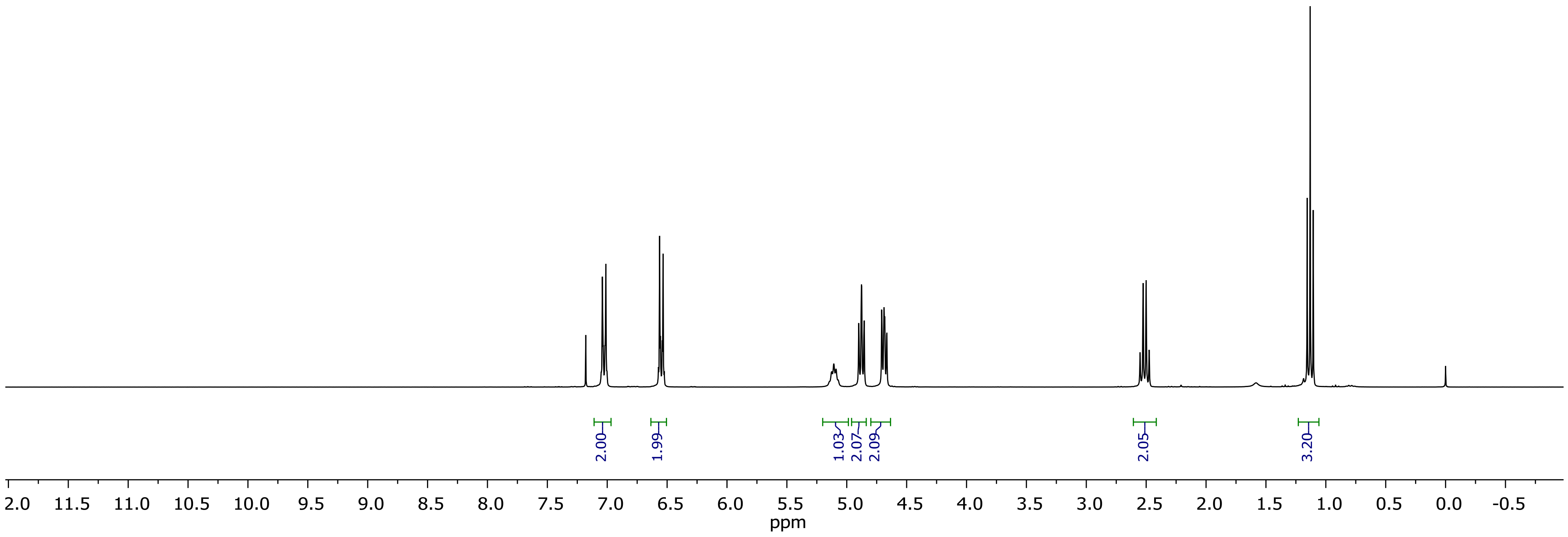

^{13}C NMR of 3-(4-ethylphenoxy)oxetane (33)

$CDCl_3$, 25 °C

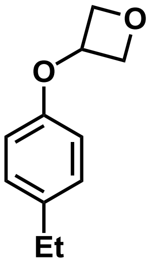

33

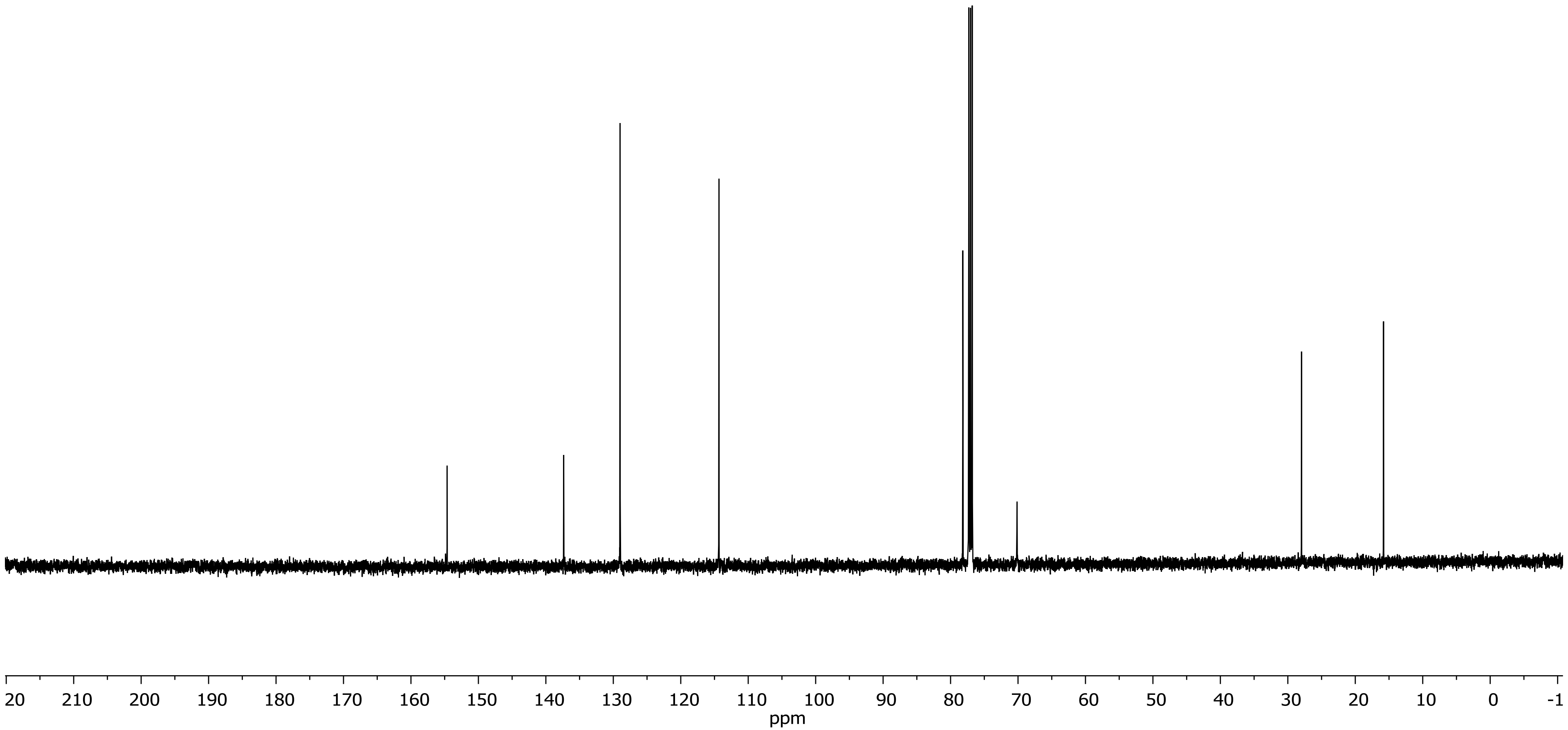

^{1}H NMR of 2-(2,2,2-trifluoroethoxy)-9H-xanthen-9-one (34)

$CDCl_3$, 25 °C

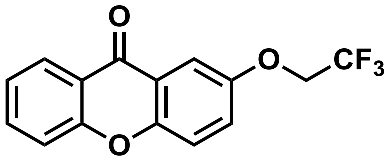

34

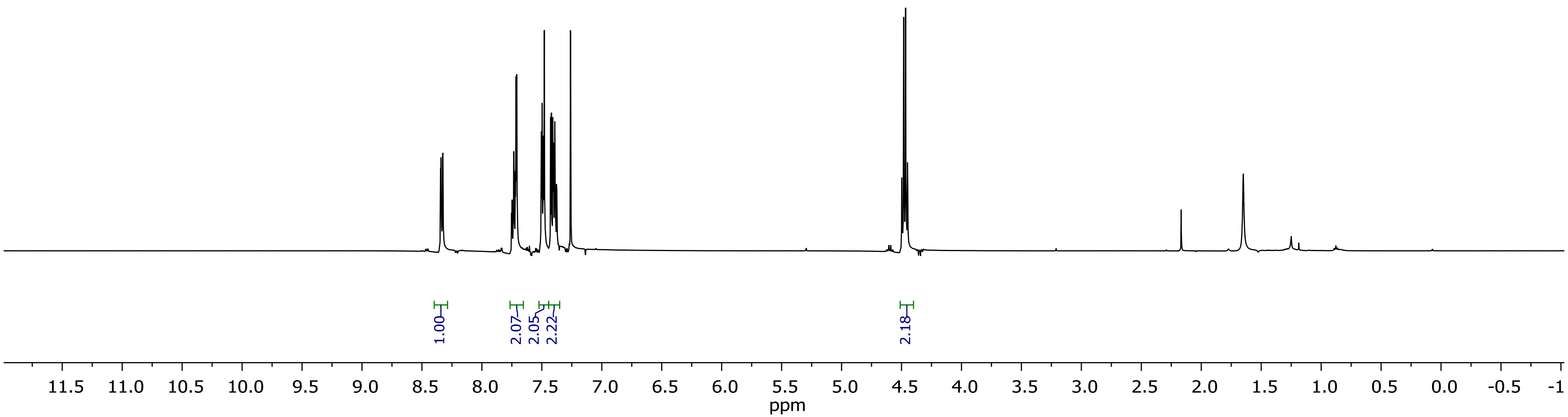

^{13}C NMR of 2-(2,2,2-trifluoroethoxy)-9H-xanthen-9-one (34)

$CDCl_3$, 25 °C

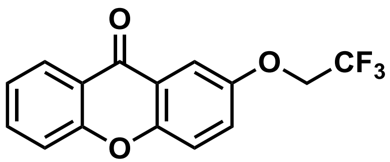

34

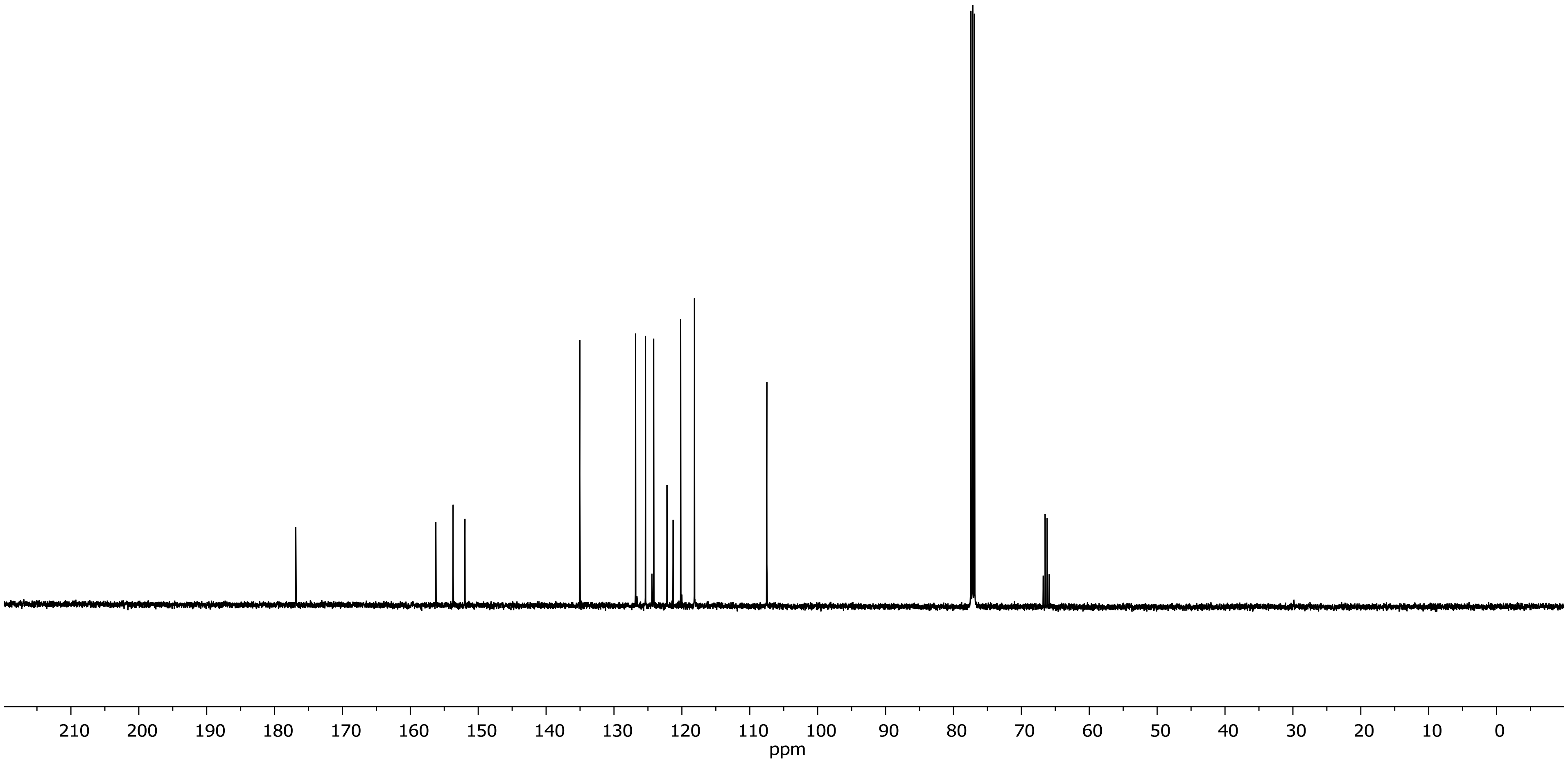

^{19}F NMR of 2-(2,2,2-trifluoroethoxy)-9H-xanthen-9-one (34)

$CDCl_3$, 25 °C

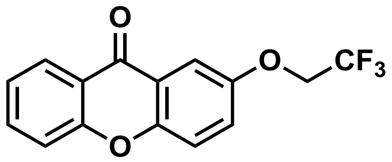

34

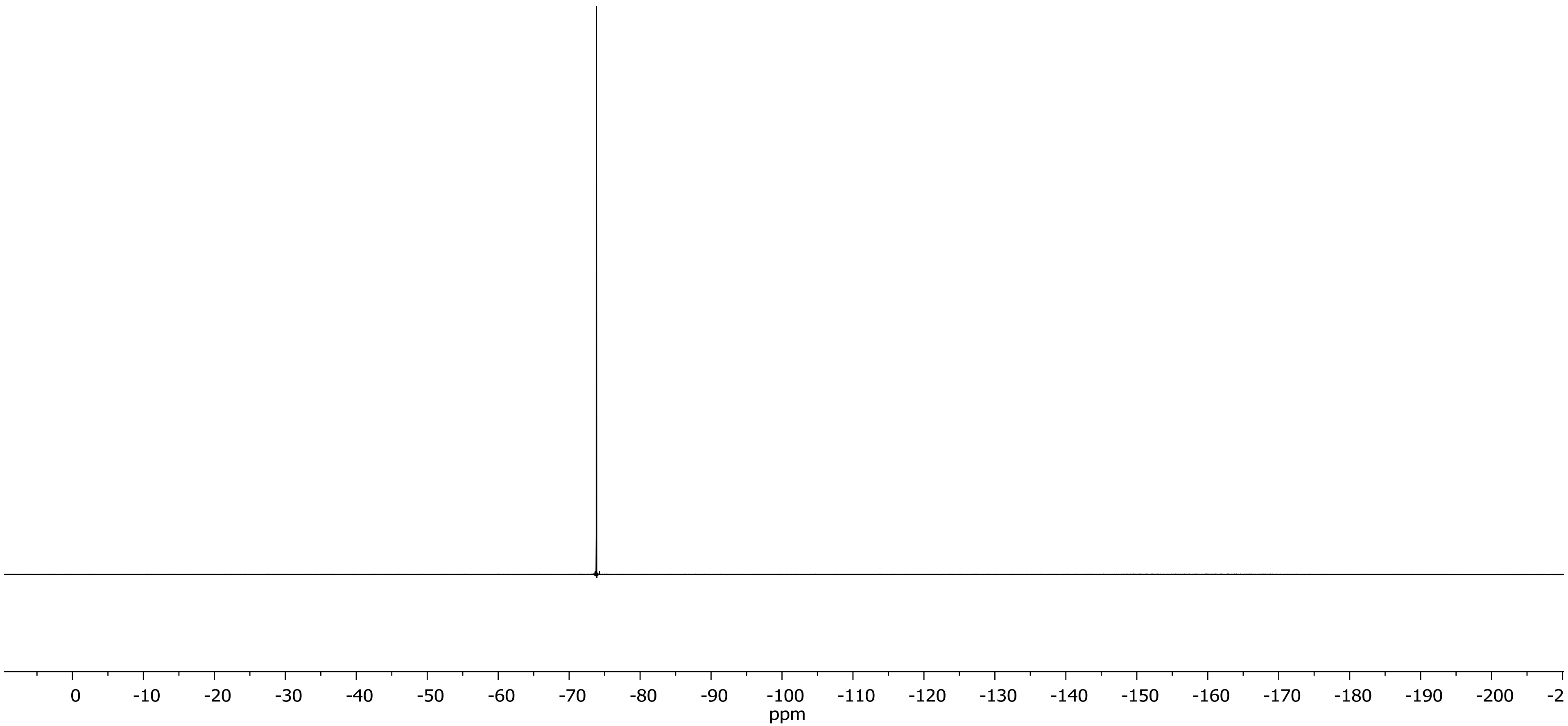

^{1}H NMR of methoxy-PEG(4) ethylbenzene (35)

CDCl$_3$, 25 °C

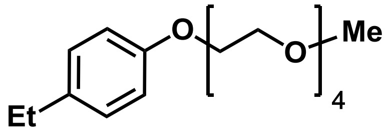

35

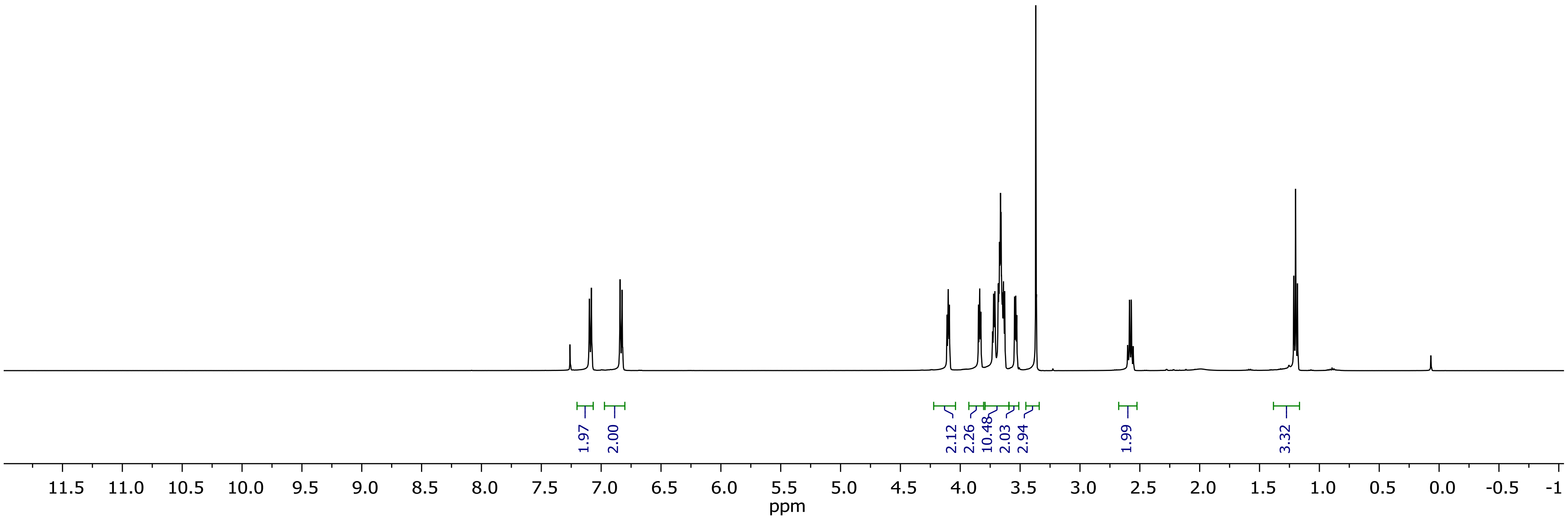

^{13}C NMR of methoxy-PEG(4) ethylbenzene (35)

$CDCl_3$, 25 °C

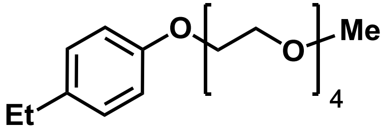

35

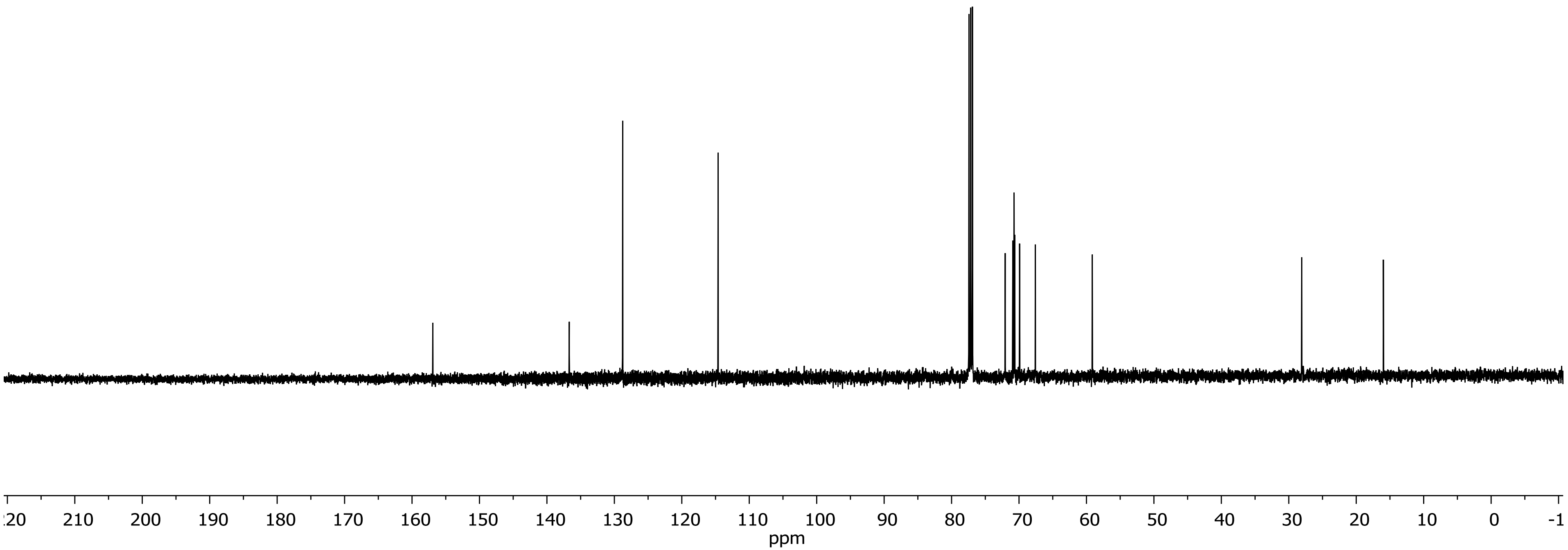

^{1}H NMR of 2-(4-ethoxyphenoxy)-6-fluorobenzonitrile (36)

CDCl$_3$, 25 °C

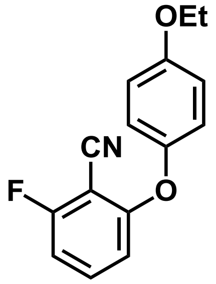

36

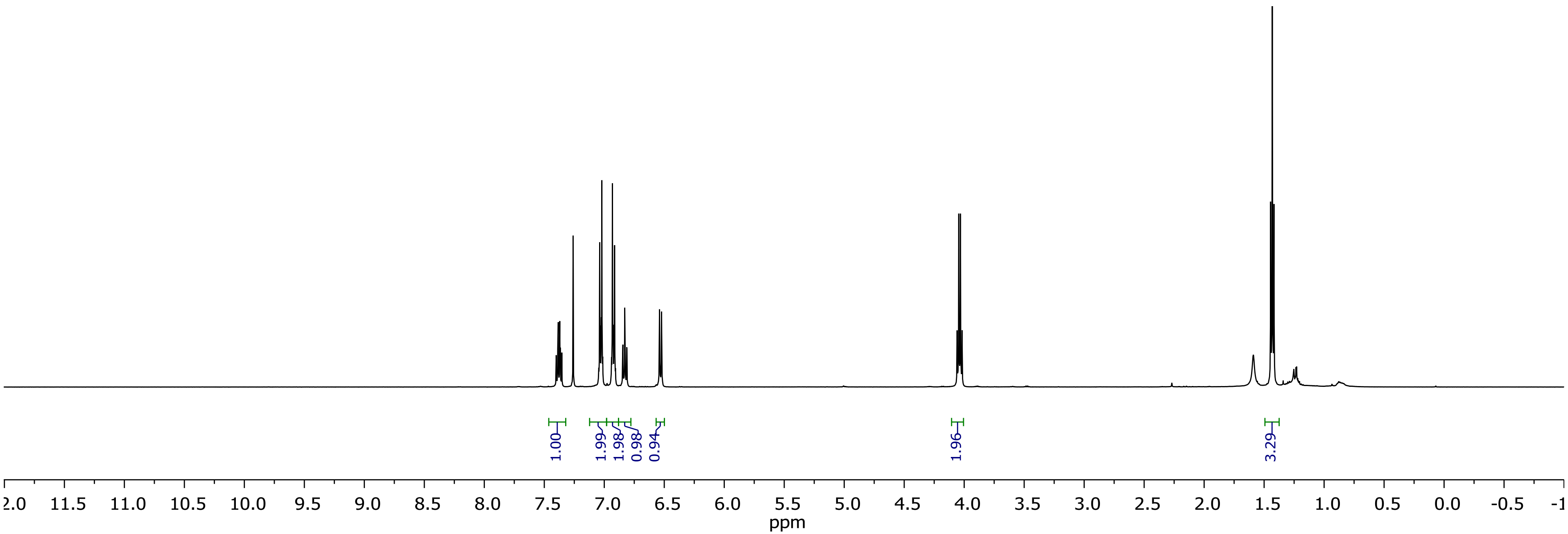

^{13}C NMR of 2-(4-ethoxyphenoxy)-6-fluorobenzonitrile (36)

CDCl$_3$, 25 °C

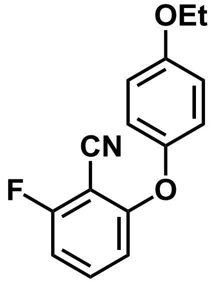

36

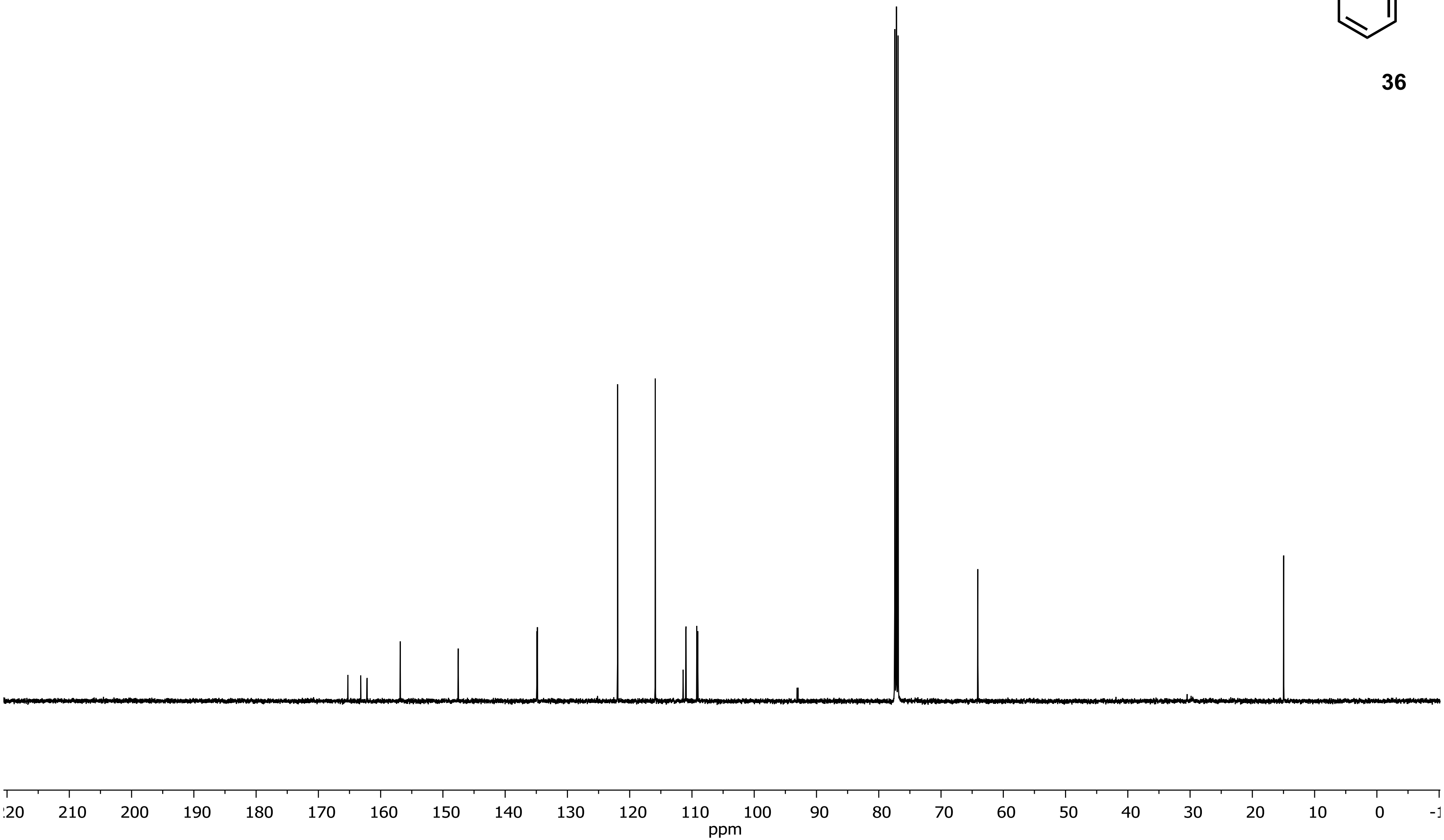

^{19}F NMR of 2-(4-ethoxyphenoxy)-6-fluorobenzonitrile (36)

$CDCl_3$, 25 °C

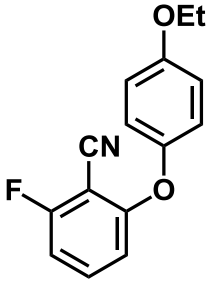

36

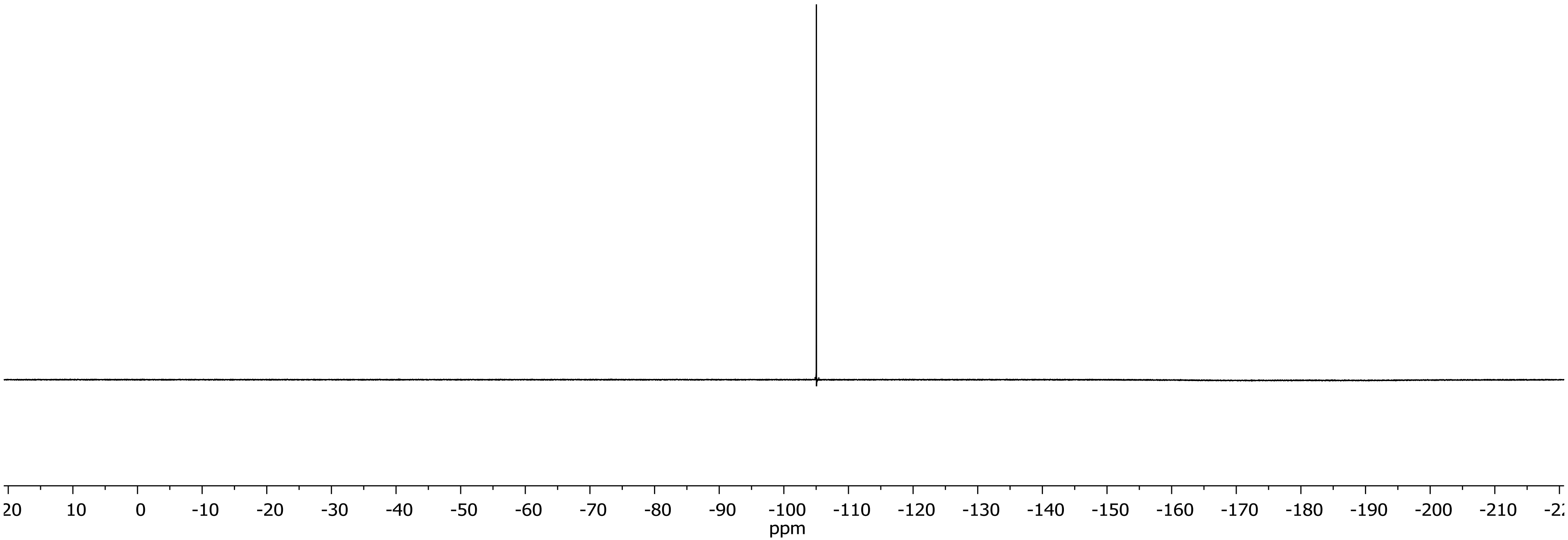

^{1}H NMR of *erythro*-3-(4-ethylphenoxy)butan-2-ol (37a)

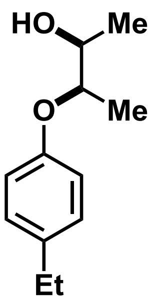

(±)37a

$CDCl_3$, 25 °C

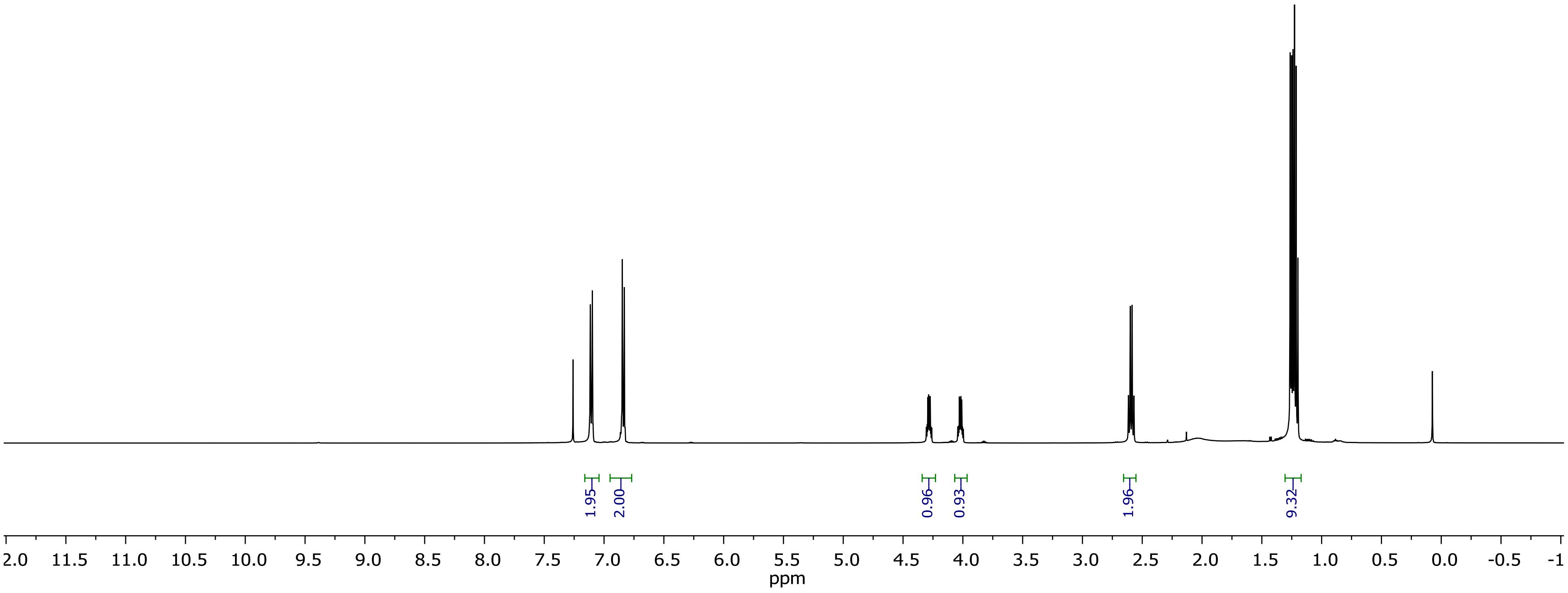

^{13}C NMR of *erythro*-3-(4-ethylphenoxy)butan-2-ol (37a)

$CDCl_3$, 25 °C

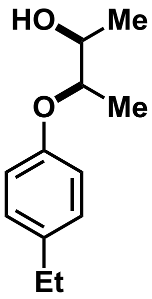

(±)37a

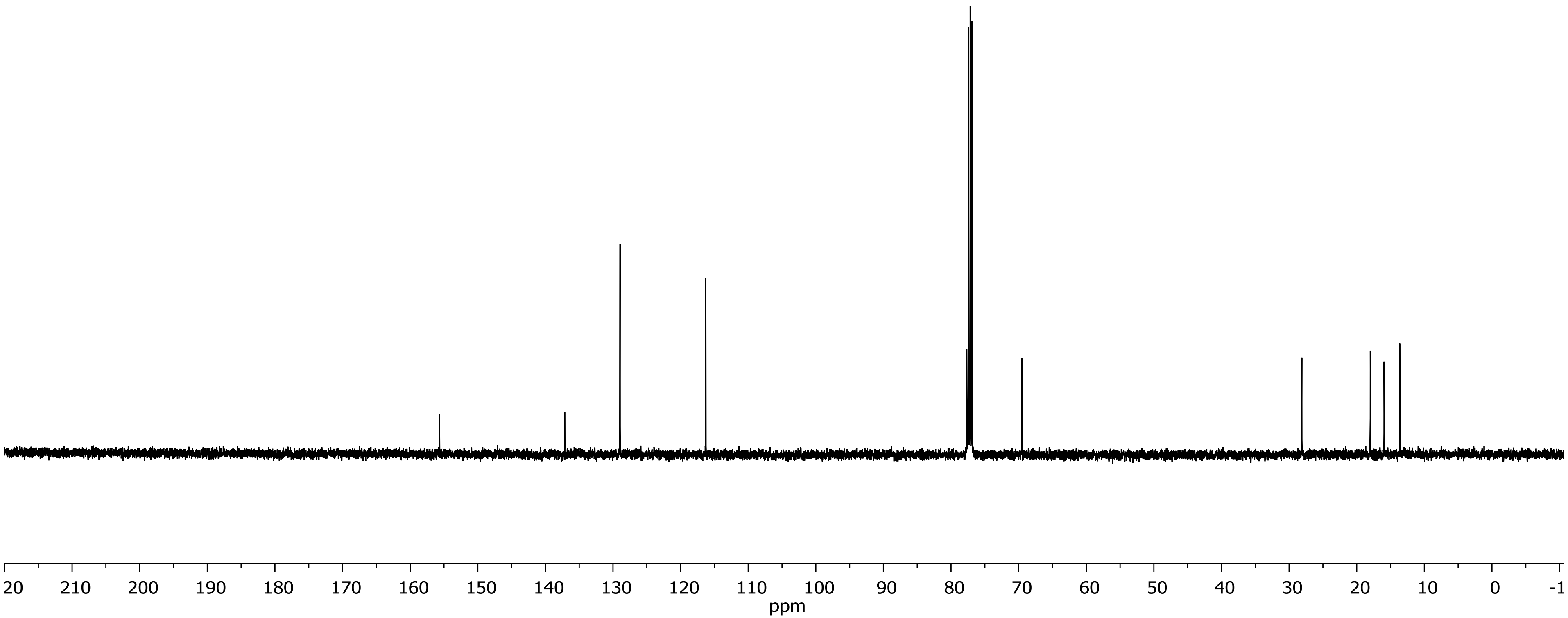

^{1}H NMR of *threo*-3-(4-ethylphenoxy)butan-2-ol (37b)

$CDCl_3$, 25 °C

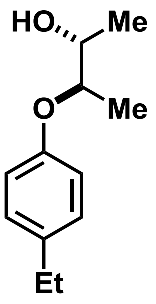

(±)37b

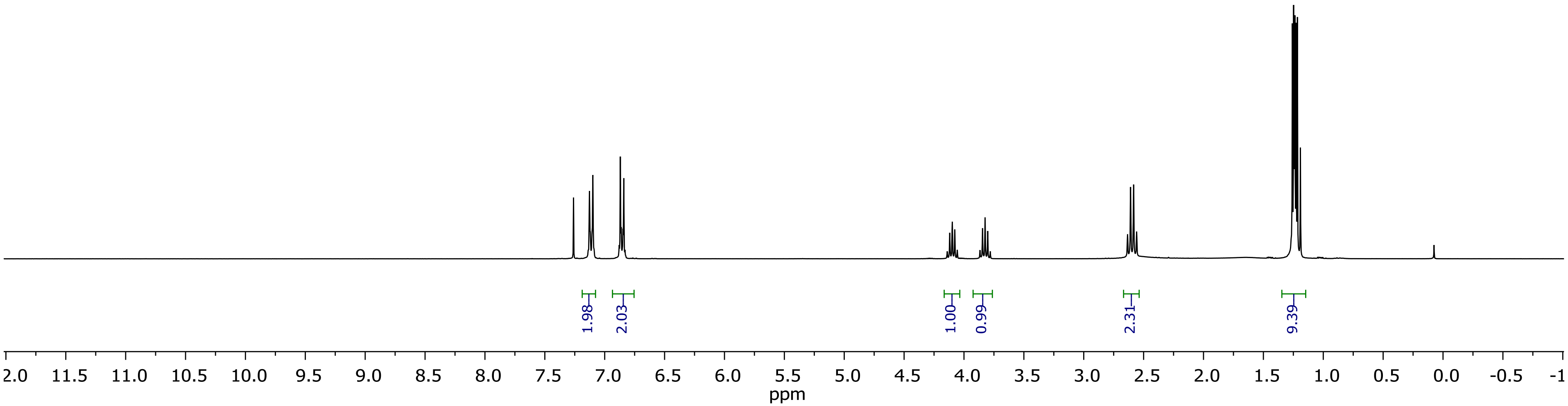

^{13}C NMR of *threo*-3-(4-ethylphenoxy)butan-2-ol (37b)

$CDCl_3$, 25 °C

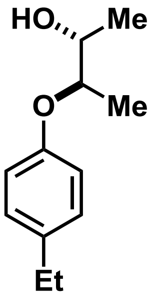

(±)37b

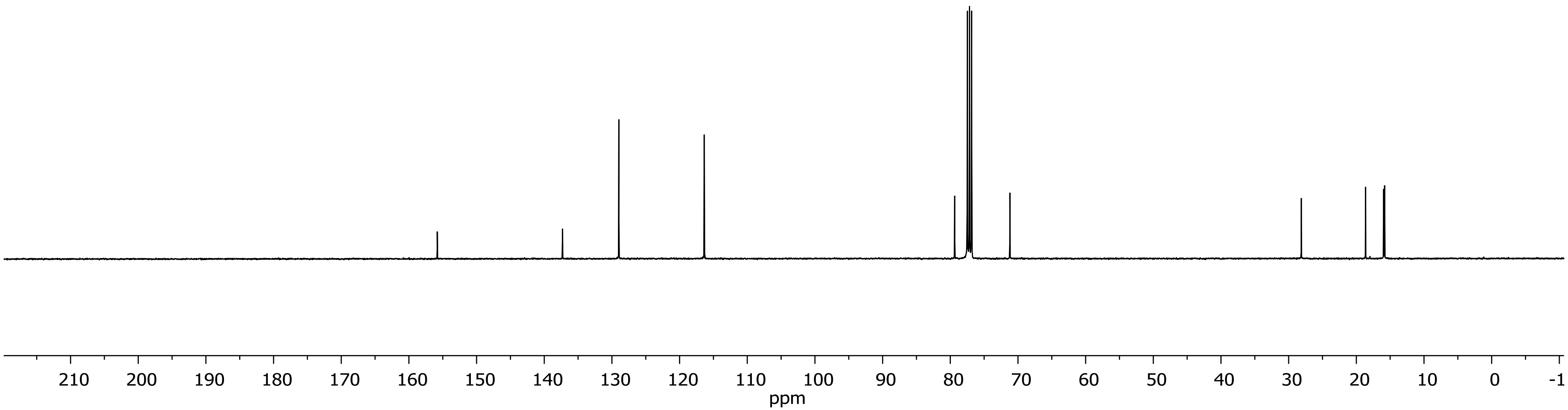

^{1}H NMR of 1-(*tert*-butyl) 2-methyl (2*S*,4*R*)-4-(4-ethylphenoxy)pyrrolidine-1,2-dicarboxylate (38)

CD_3CN_3, 25 °C

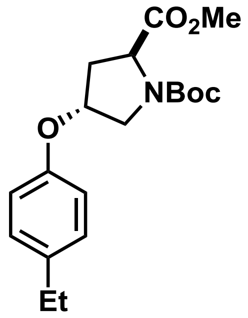

38

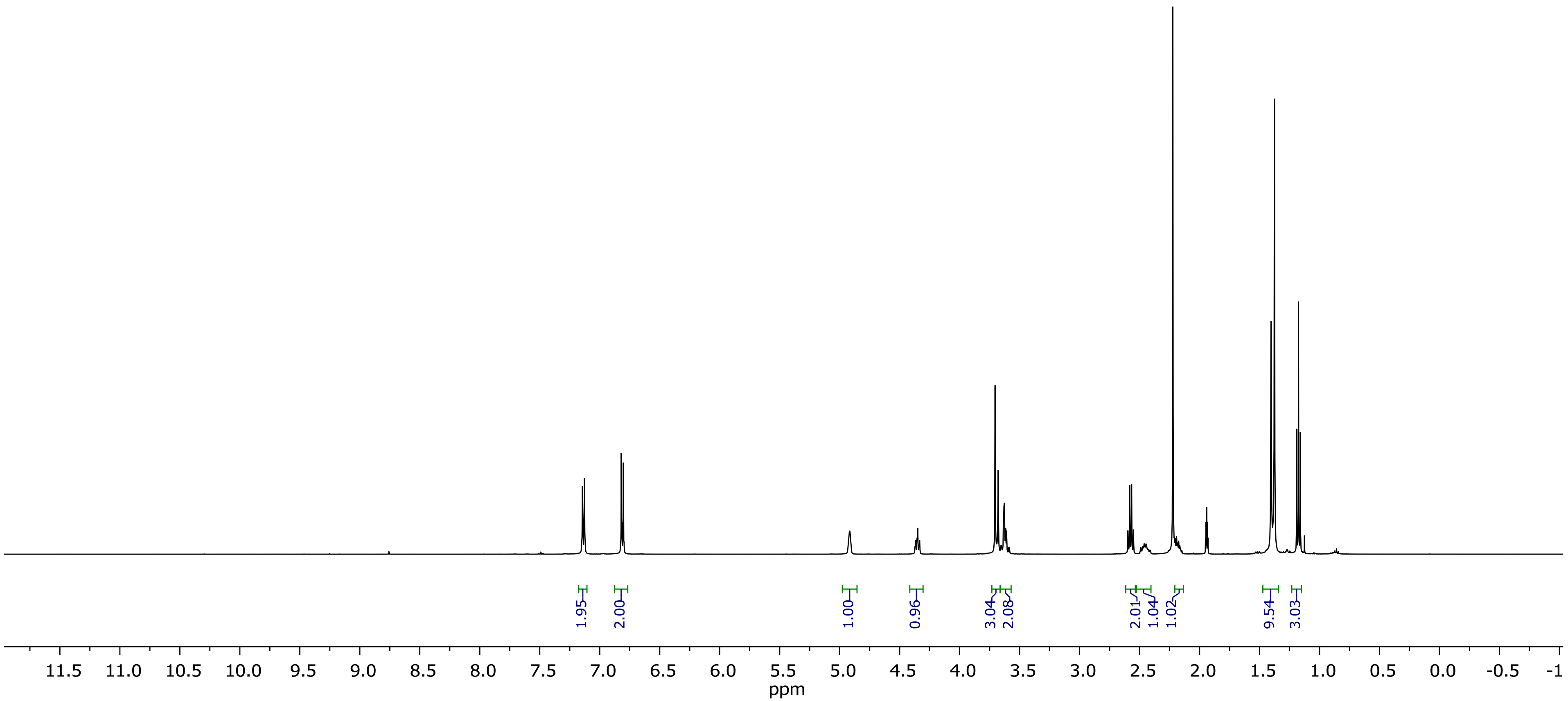

^{13}C NMR of 1-(*tert*-butyl) 2-methyl (2*S*,4*R*)-4-(4-ethylphenoxy)pyrrolidine-1,2-dicarboxylate (38)

CD_3CN, 25 °C

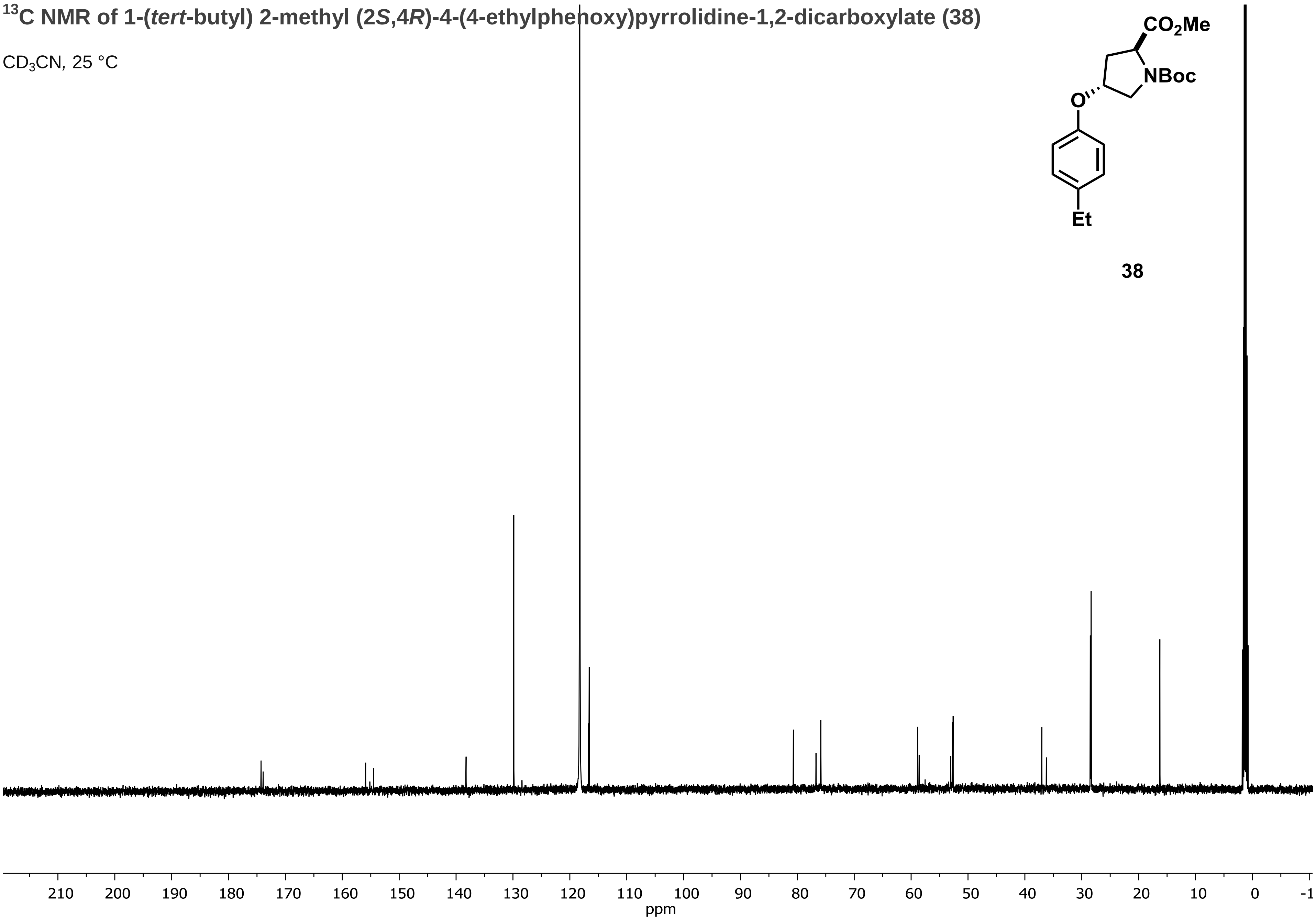

^{1}H NMR of (3a*R*,5*R*,6*S*,6a*R*)-5-((*R*)-2,2-dimethyl-1,3-dioxolan-4-yl)-6-(4-ethylphenoxy)-2,2-dimethyltetrahydro-furo[2,3-d][1,3]dioxole (39)

$CDCl_3$, 25 °C

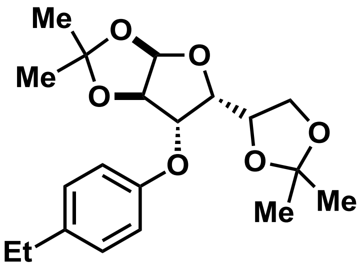

39

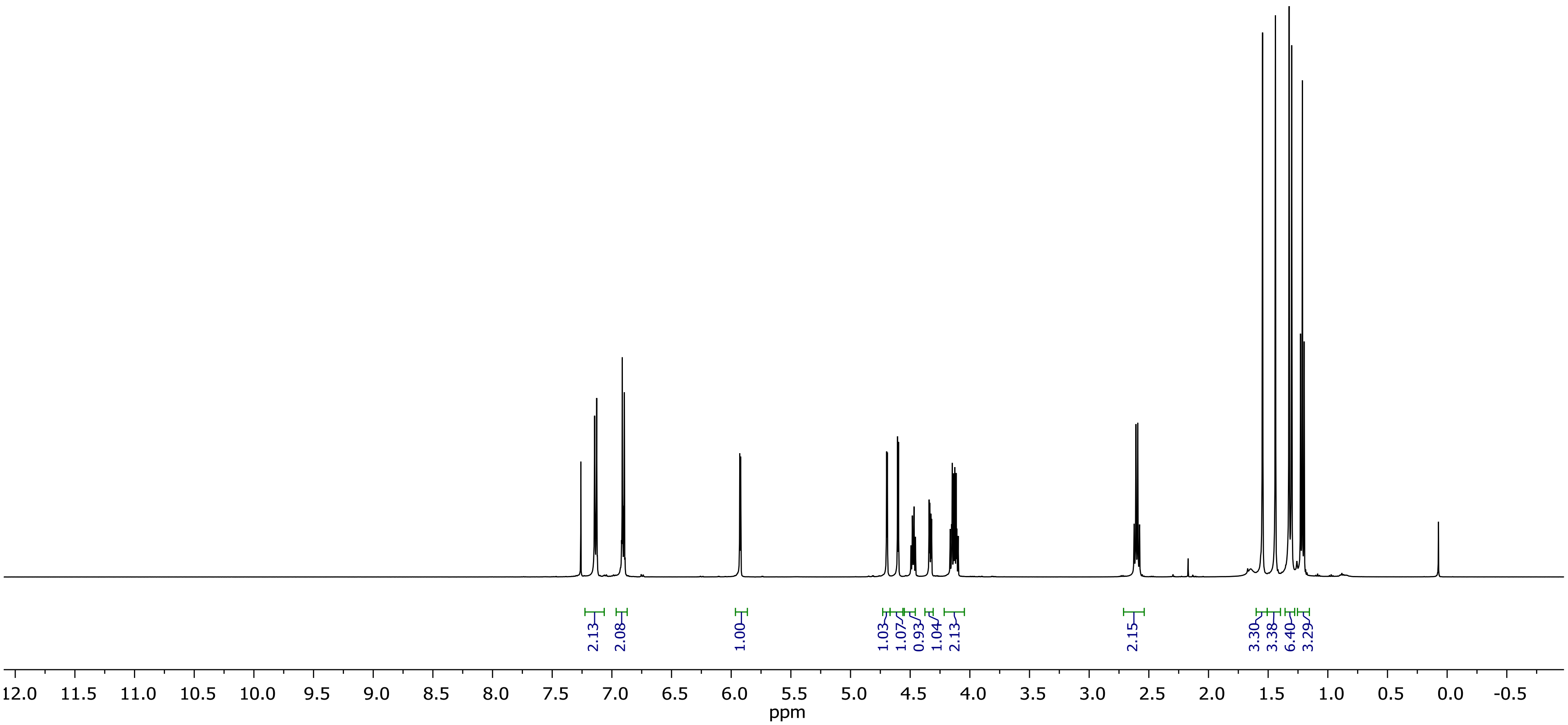

^{13}C NMR of (3a*R*,5*R*,6*S*,6a*R*)-5-((*R*)-2,2-dimethyl-1,3-dioxolan-4-yl)-6-(4-ethylphenoxy)-2,2-dimethyltetrahydro-furo[2,3-d][1,3]dioxole (39)

$CDCl_3$, 25 °C

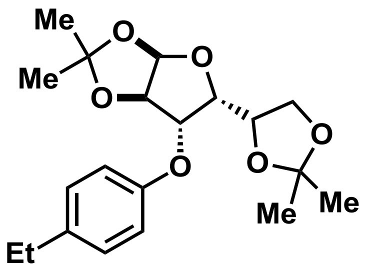

39

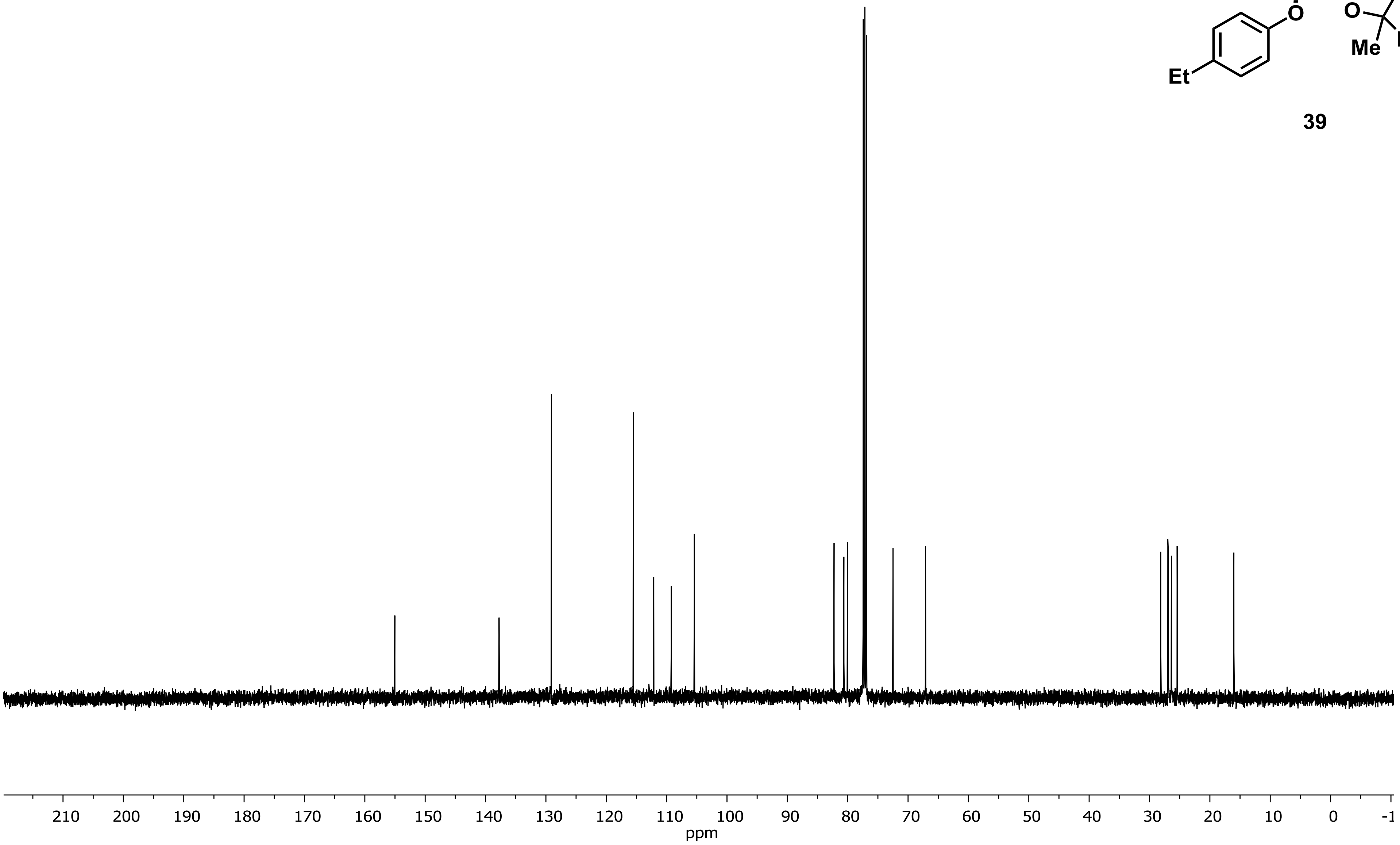

^{1}H NMR of 4-methyl-*N*-phenylbenzenesulfonamide tetrafluorothianthrenium salt (40-TFT)

CD_3CN, 25 °C

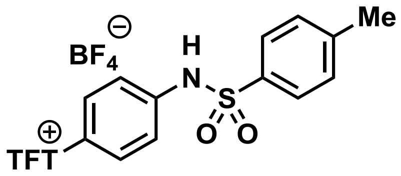

40-TFT

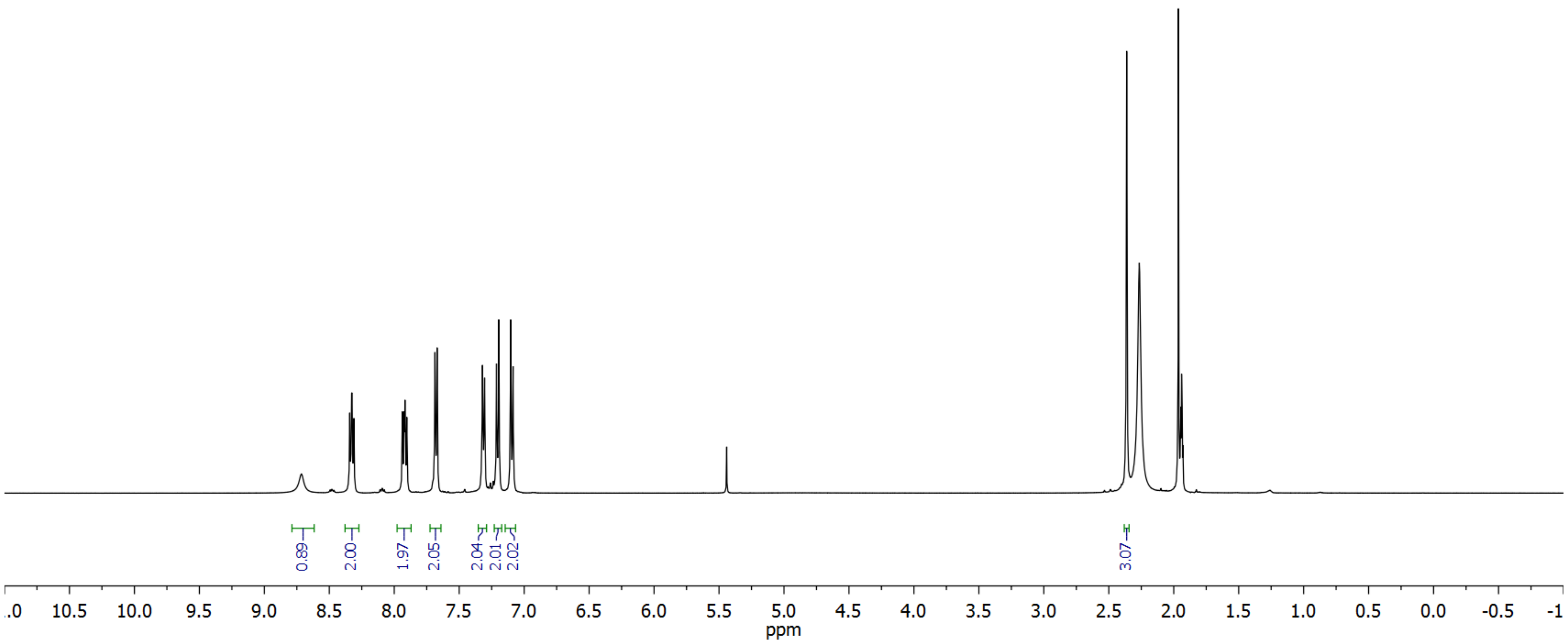

^{13}C NMR of 4-methyl-*N*-phenylbenzenesulfonamide tetrafluorothianthrenium salt (40-TFT)

CD_3CN, 25 °C

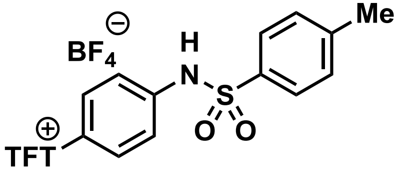

40-TFT

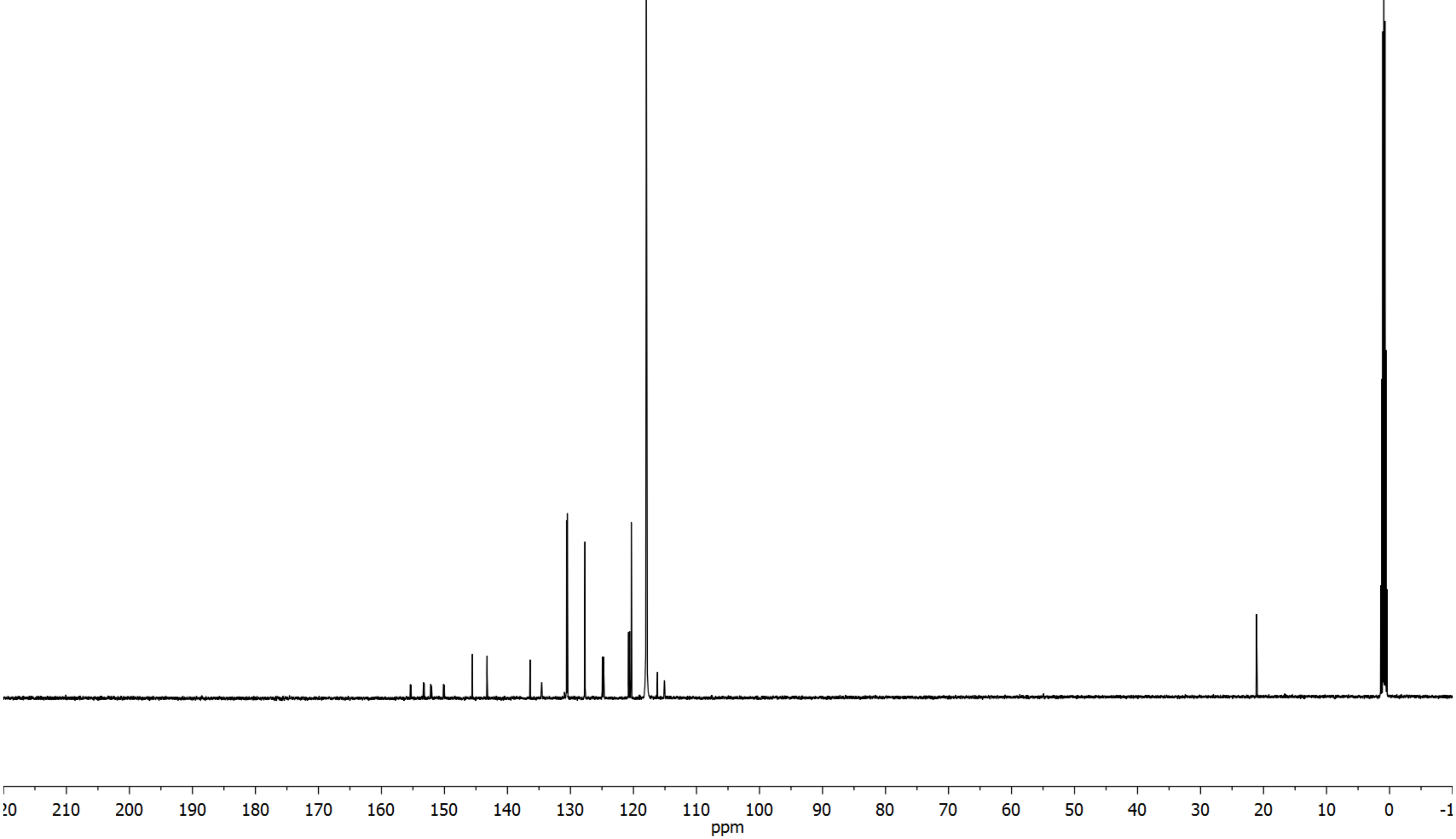

^{19}F NMR of 4-methyl-*N*-phenylbenzenesulfonamide tetrafluorothianthrenium salt (40-TFT)

CD_3CN, 25 °C

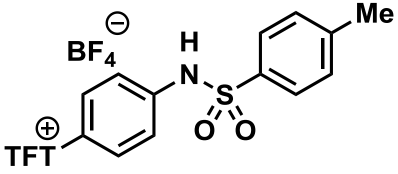

40-TFT

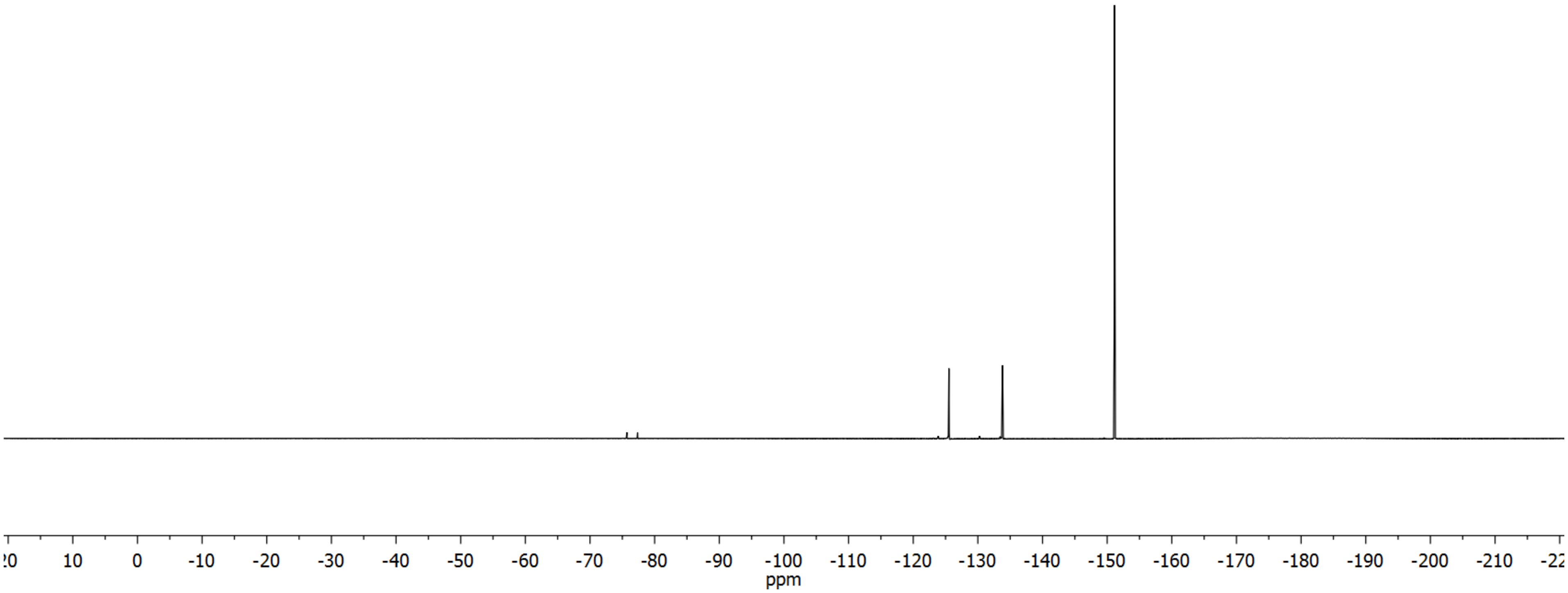

^{1}H NMR of N-(4-methoxyphenyl)-4-methylbenzenesulfonamide (40)

CD_2Cl_2, 25 °C

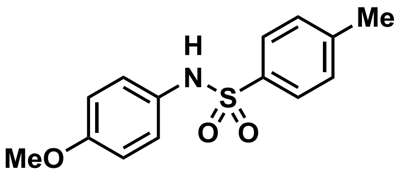

40

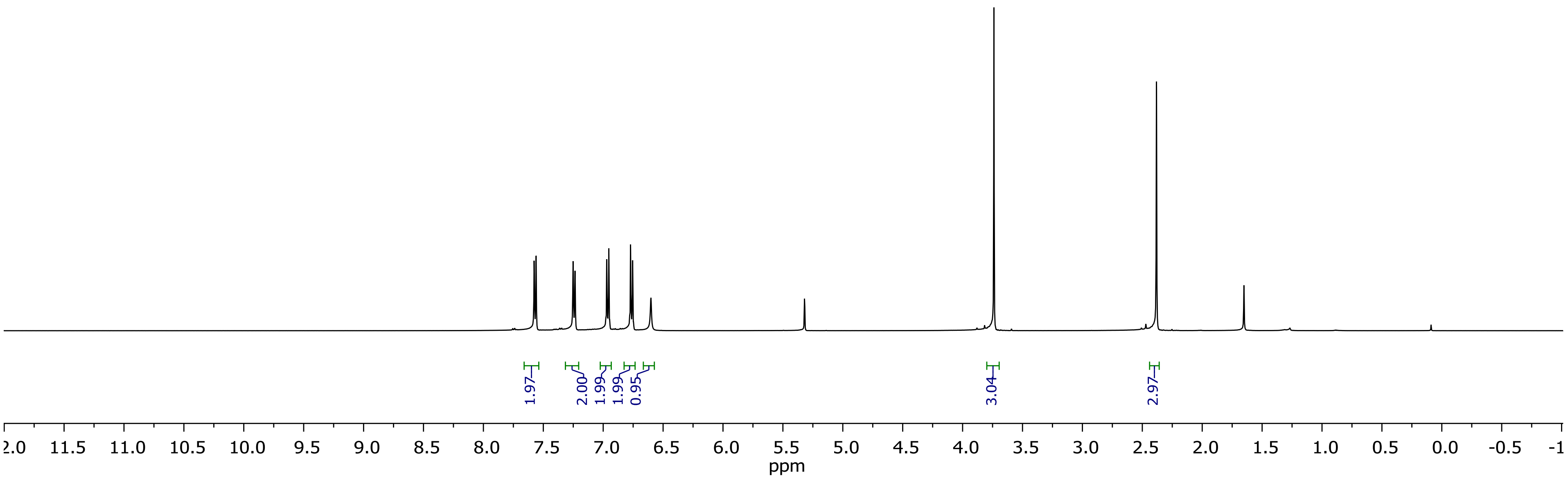

^{13}C NMR of N-(4-methoxyphenyl)-4-methylbenzenesulfonamide (40)

CD_2Cl_2, 25 °C

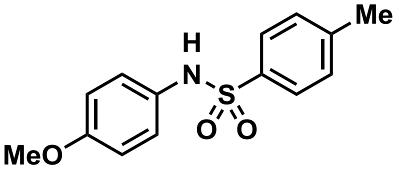

40

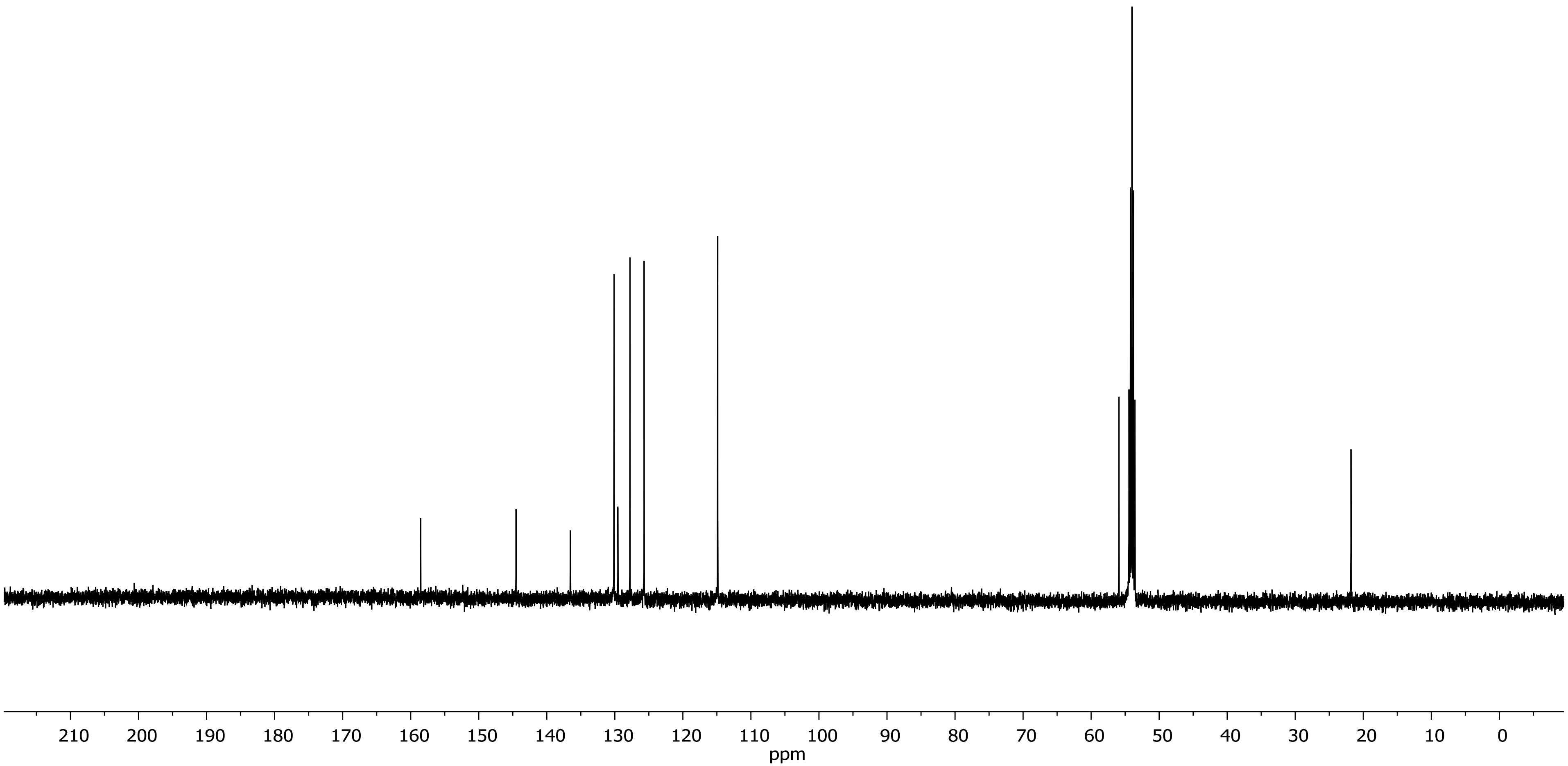

^{1}H NMR of *tert*-butyl 3-(4-((benzoyloxy)methyl)phenoxy)azetidine-1-carboxylate (41)

CD_3CN, 25 °C

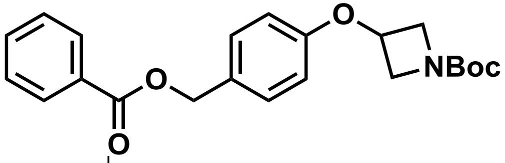

41

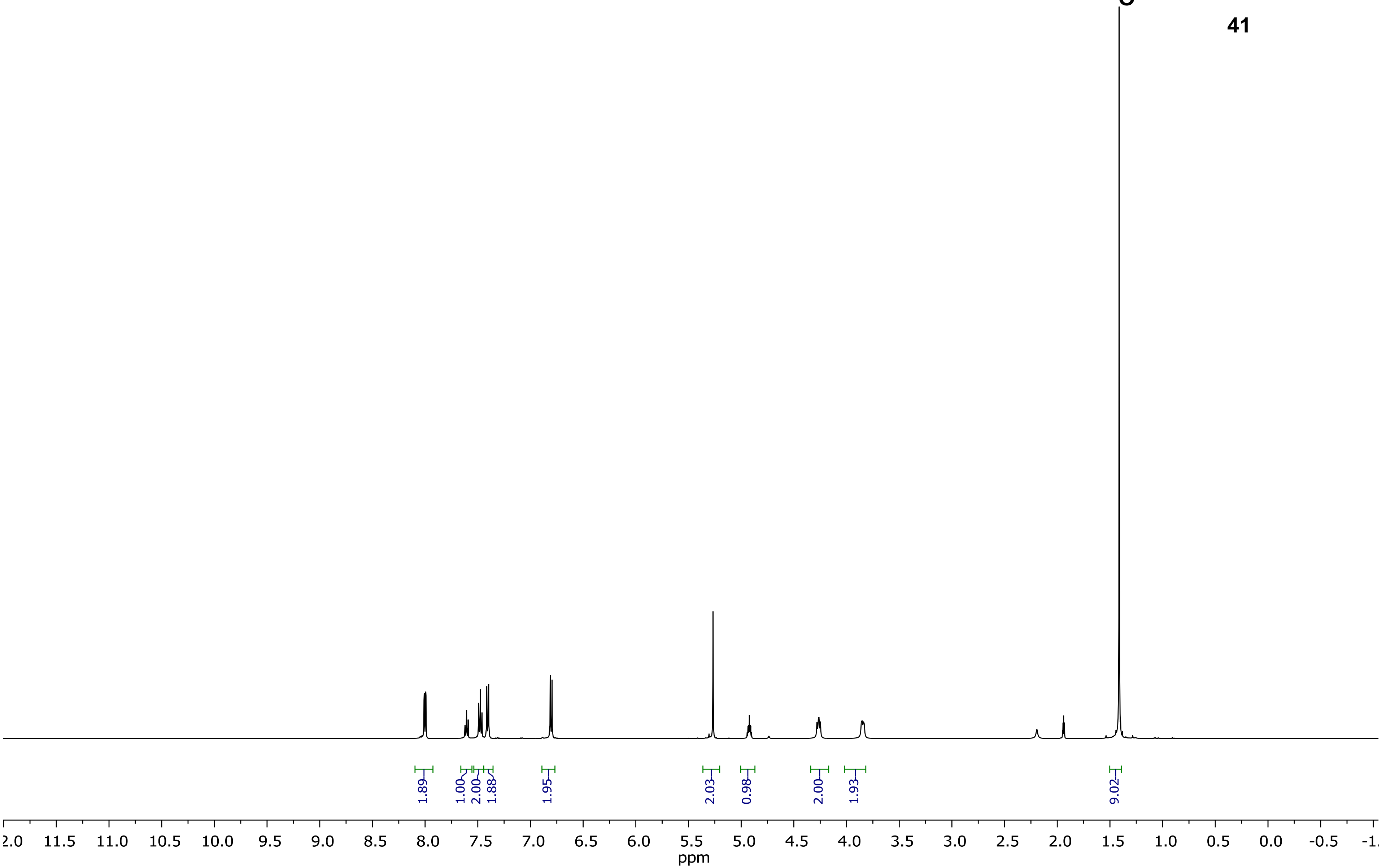

^{13}C NMR of *tert*-butyl 3-(4-((benzoyloxy)methyl)phenoxy)azetidine-1-carboxylate (41)

CD_3CN, 25 °C

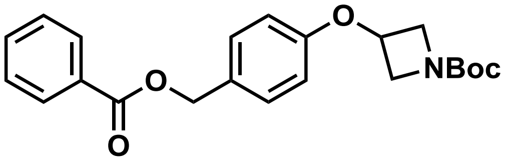

41

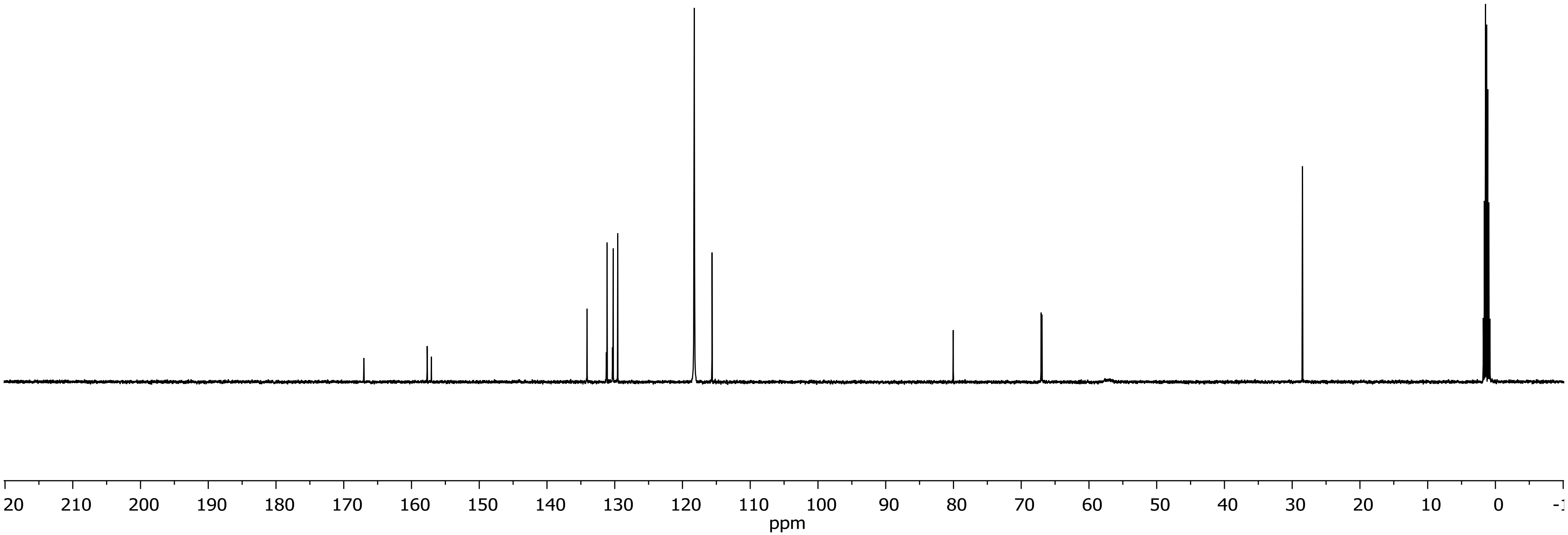

^{1}H NMR of *tert*-butyl (*R*)-3-(4-((2-oxo-3-propionyloxazolidin-4-yl)methyl)phenoxy)azetidine-1-carboxylate (42)

CD_3CN, 25 °C

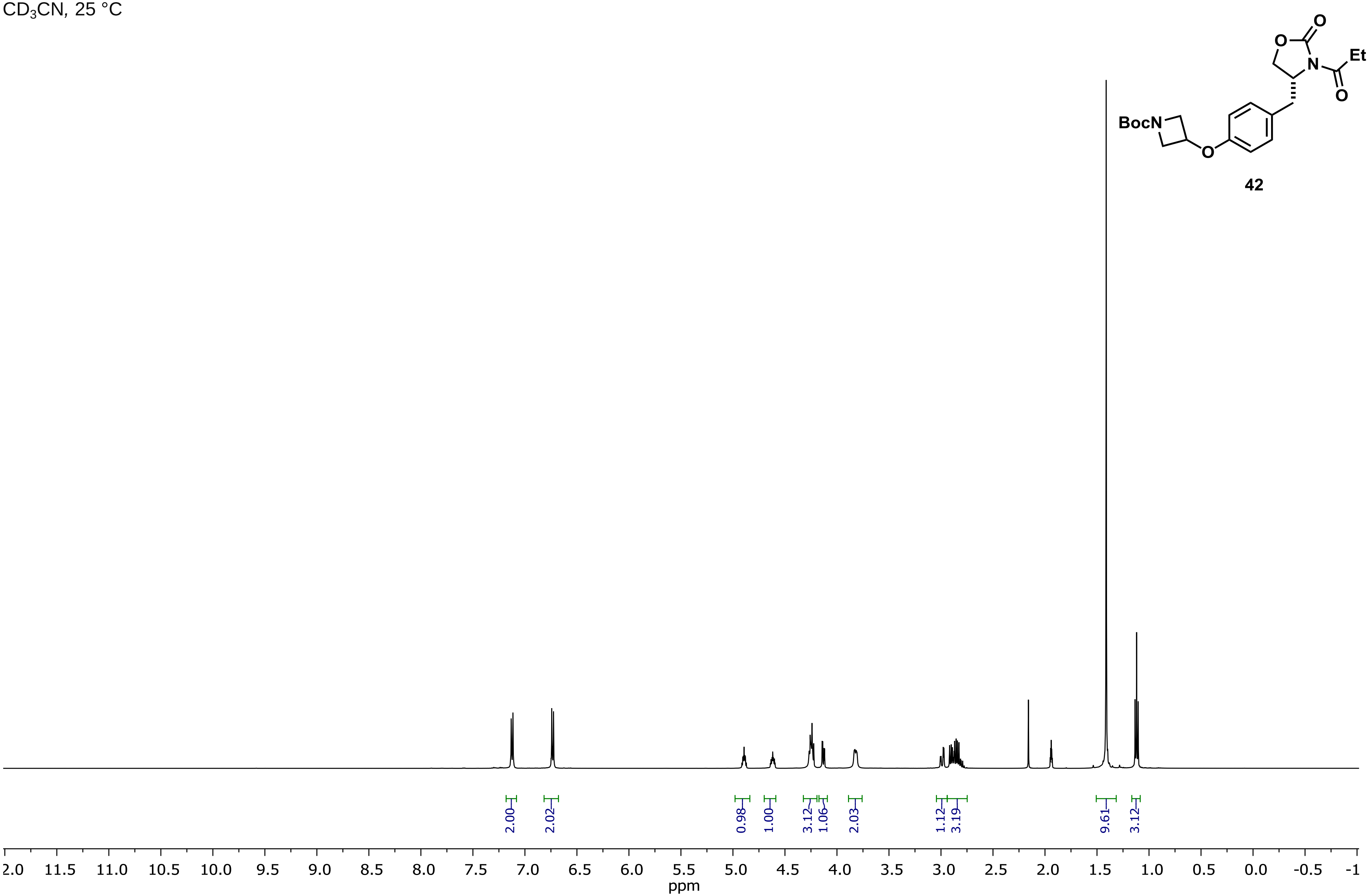

^{13}C NMR of *tert*-butyl (*R*)-3-(4-((2-oxo-3-propionyloxazolidin-4-yl)methyl)phenoxy)azetidine-1-carboxylate (42)

CD_3CN, 25 °C

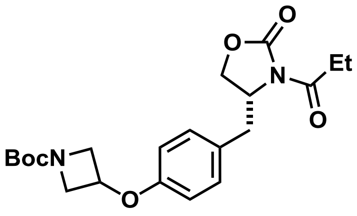

42

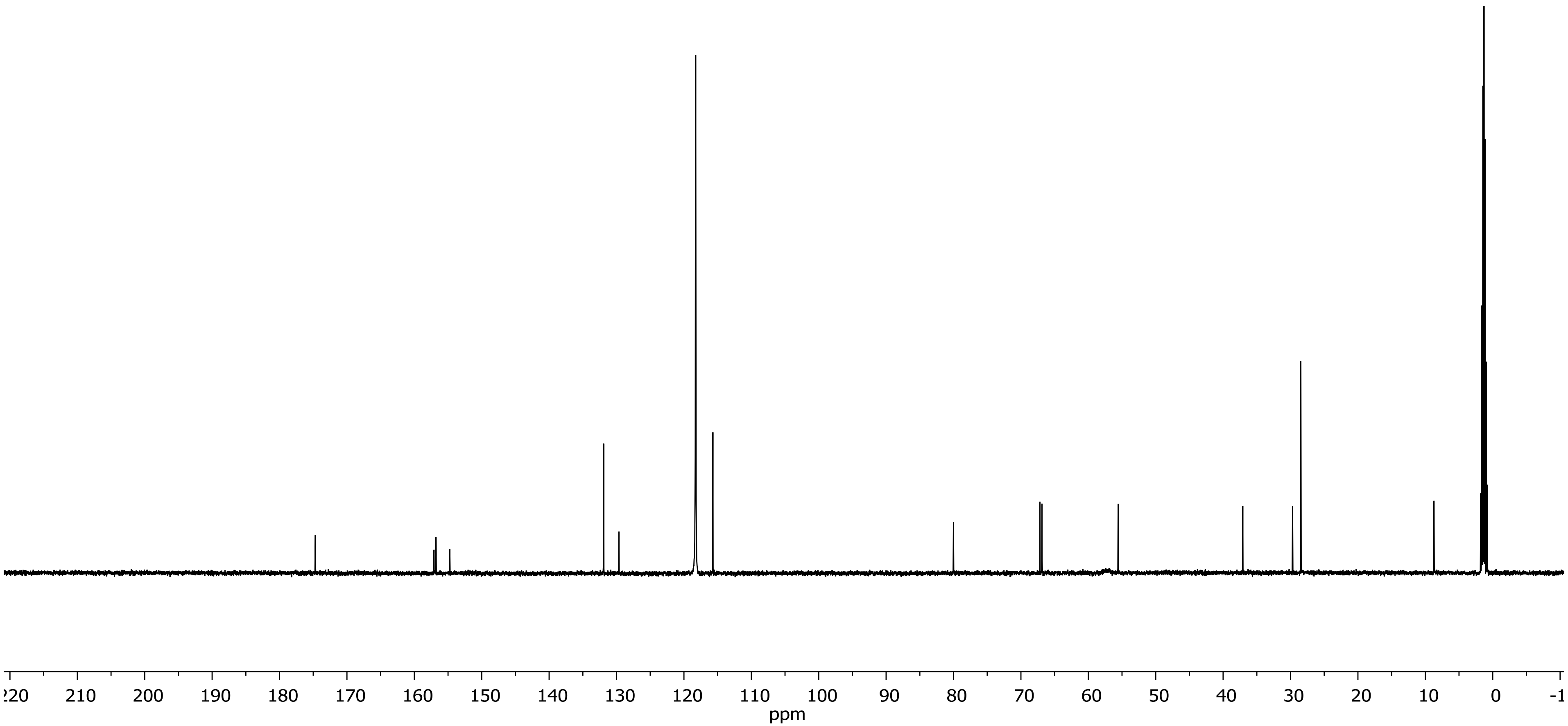

^{1}H NMR of 4-methoxy-flurbiprofen methyl ester (43)

$CDCl_3$, 25 °C

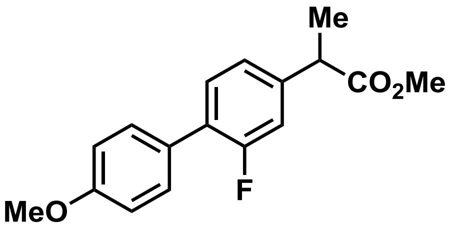

43

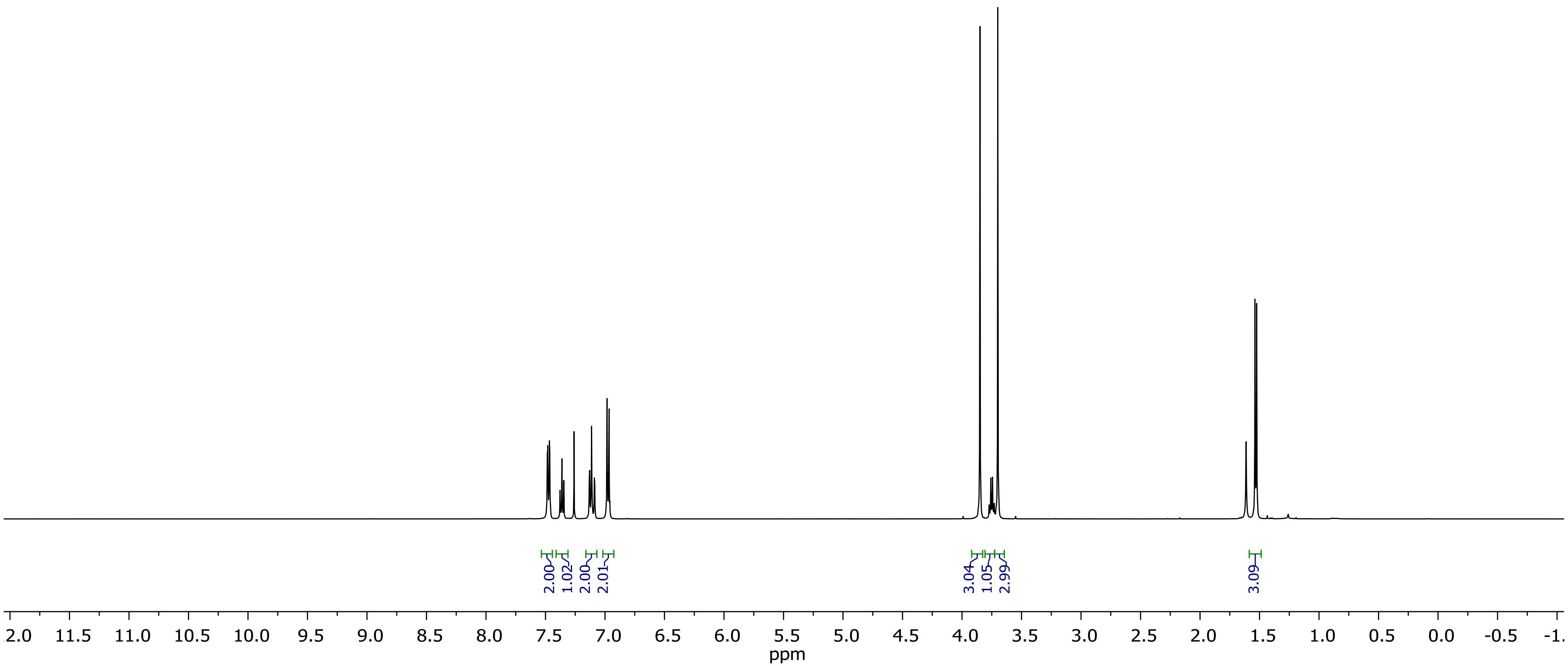

^{13}C NMR of 4-methoxy-flurbiprofen methyl ester (43)

$CDCl_3$, 25 °C

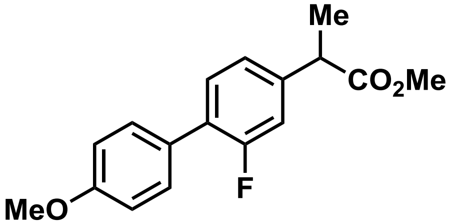

43

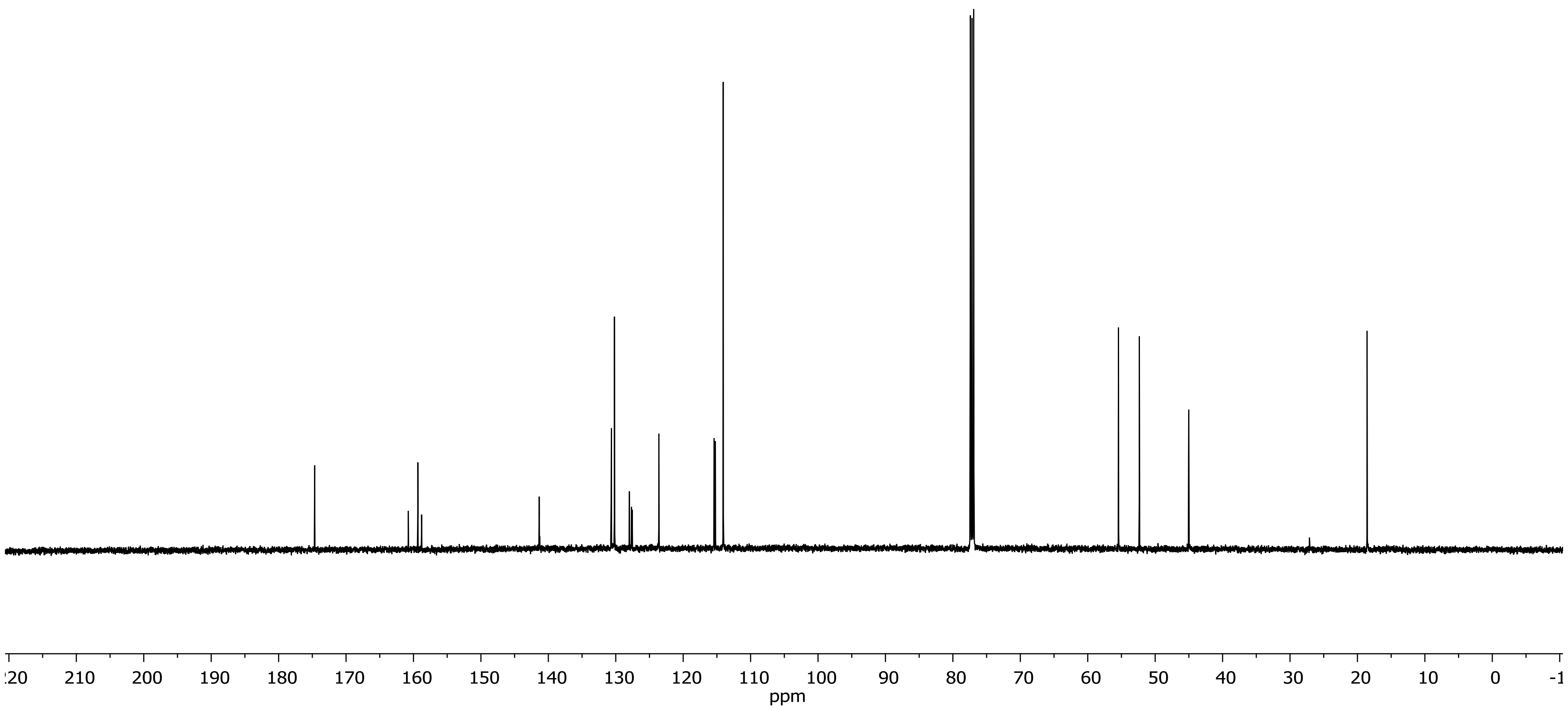

^{19}F NMR of 4-methoxy-flurbiprofen methyl ester (43)

$CDCl_3$, 25 °C

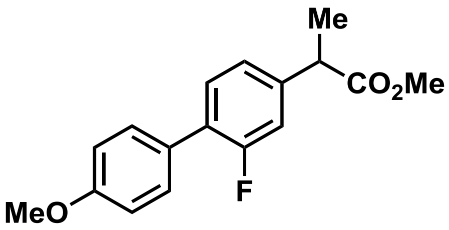

43

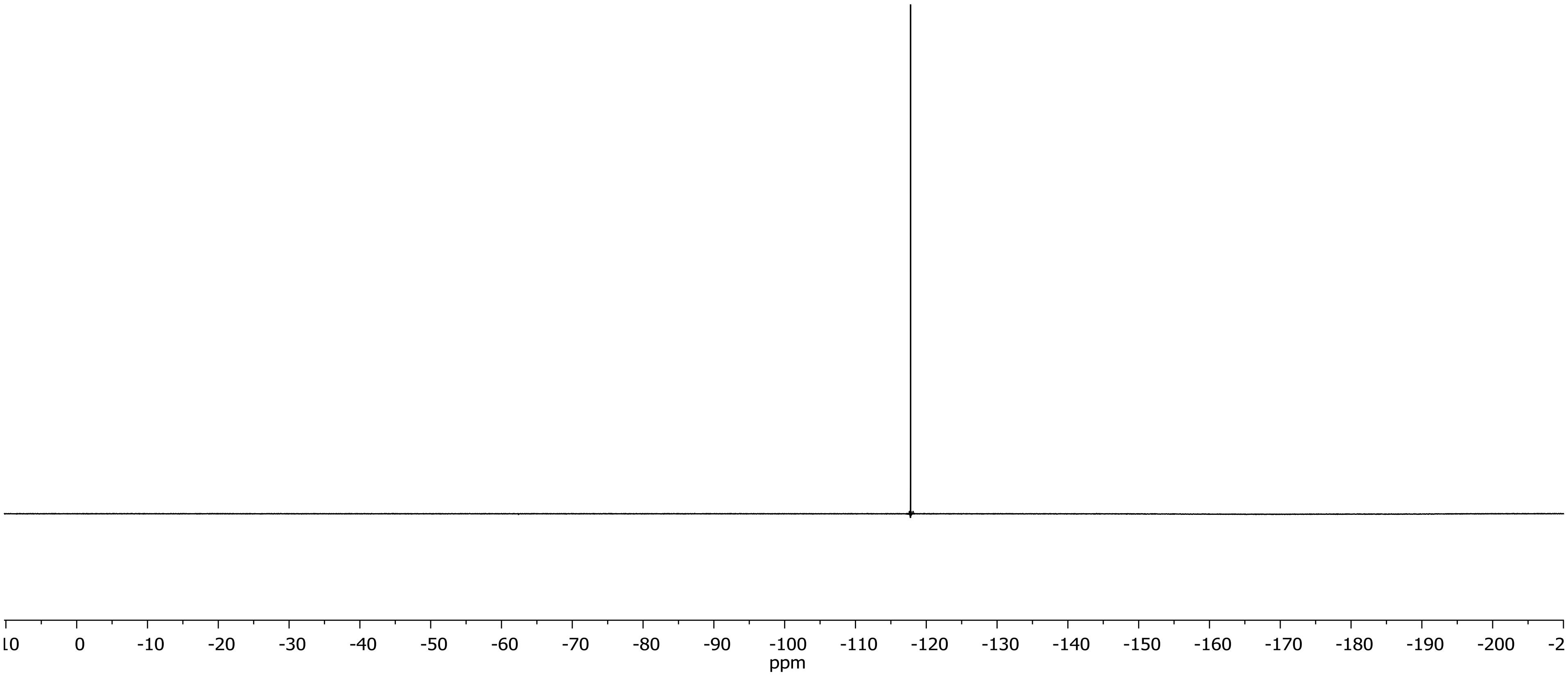

^{1}H NMR of *tert*-butyl 3-(3-formyl-4-methoxyphenoxy)azetidine-1-carboxylate (44)

$CDCl_3$, 25 °C

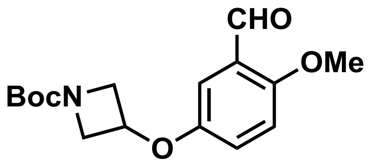

44

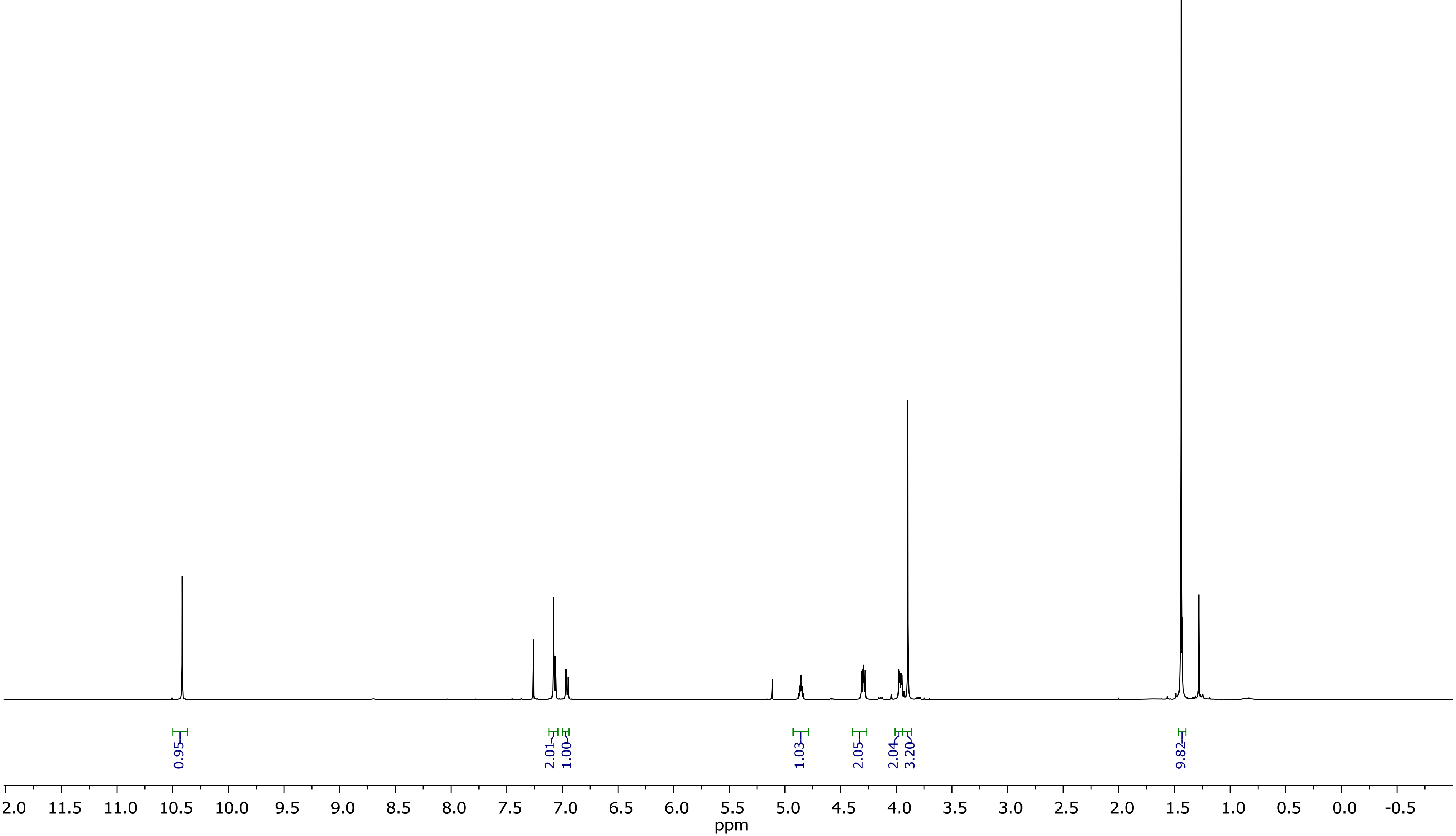

^{13}C NMR of *tert*-butyl 3-(3-formyl-4-methoxyphenoxy)azetidine-1-carboxylate (44)

$CDCl_3$, 25 °C

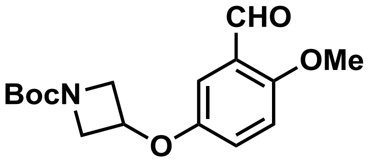

44

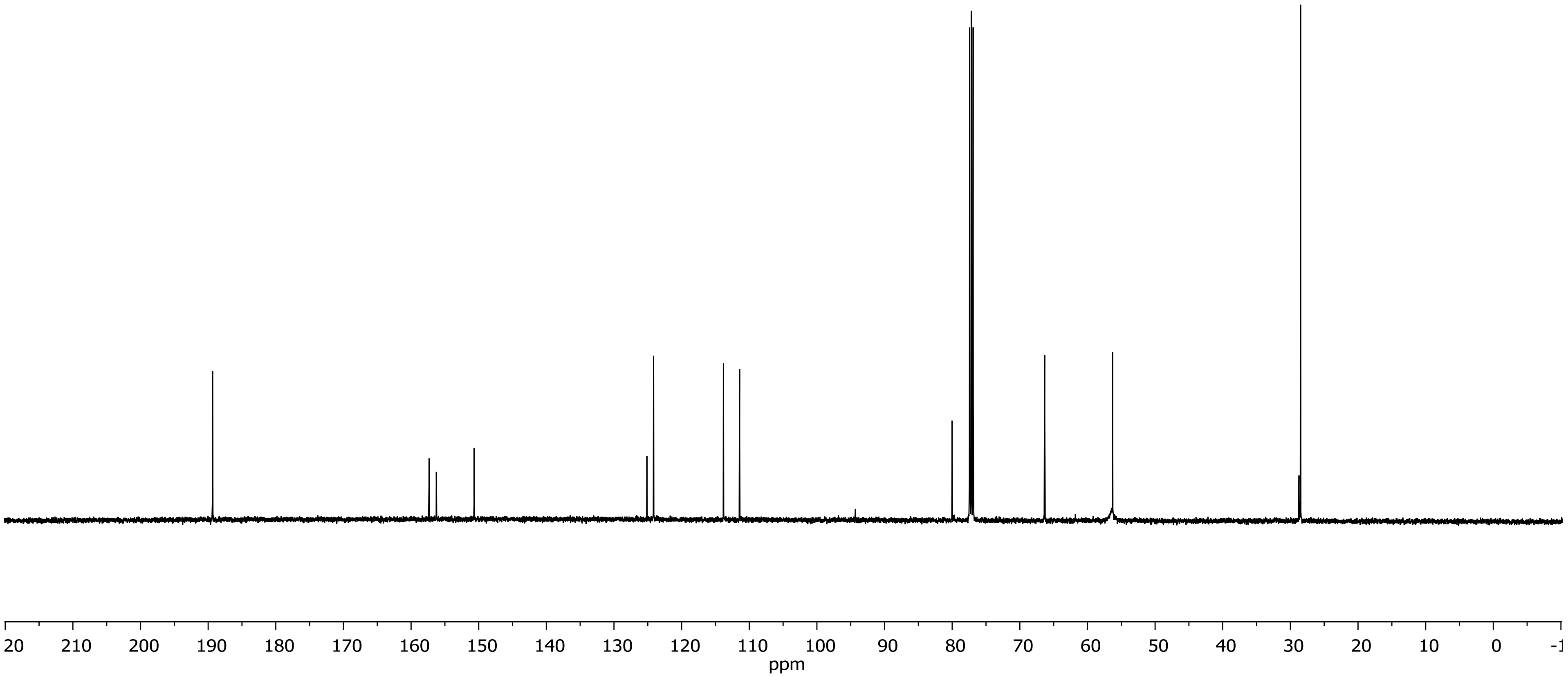

^{1}H NMR of *tert*-butyl 3-(5-((3r,5r,7r)-adamantan-1-yl)-2-methylphenoxy)azetidine-1-carboxylate (45)

$CDCl_3$, 25 °C

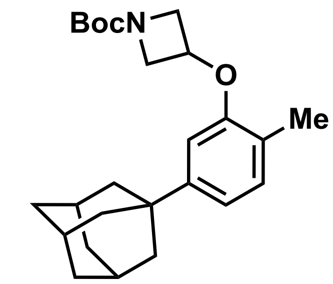

45

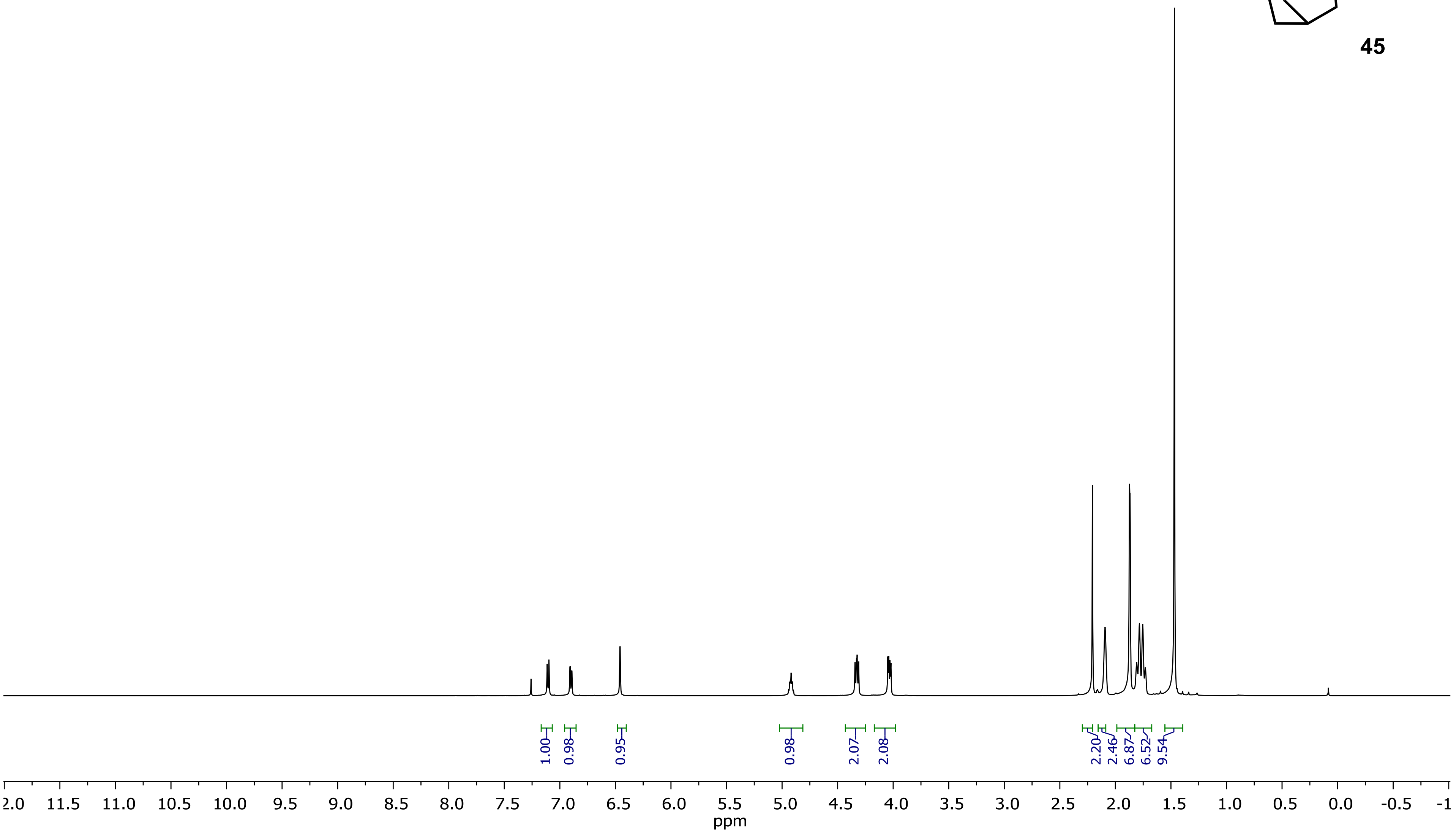

^{13}C NMR of *tert*-butyl 3-(5-((3r,5r,7r)-adamantan-1-yl)-2-methylphenoxy)azetidine-1-carboxylate (45)

$CDCl_3$, 25 °C

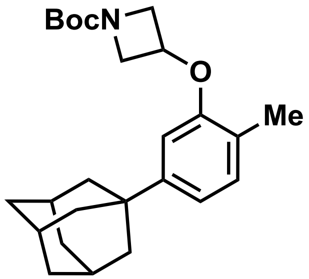

45

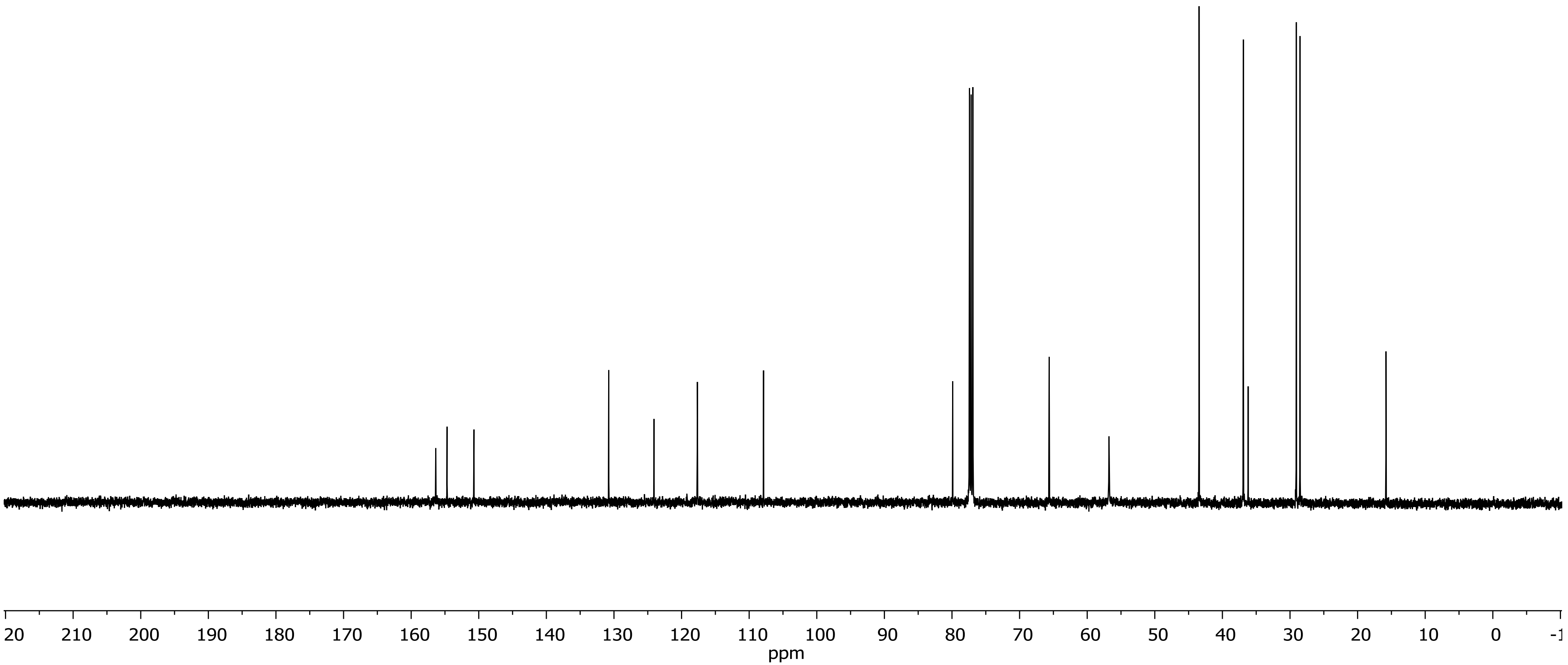

^{1}H NMR of *tert*-butyl 3-(4-(3-chloropropyl)phenoxy)azetidine-1-carboxylate (46)

CD_3CN, 25 °C

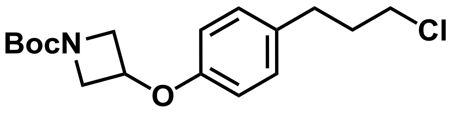

46

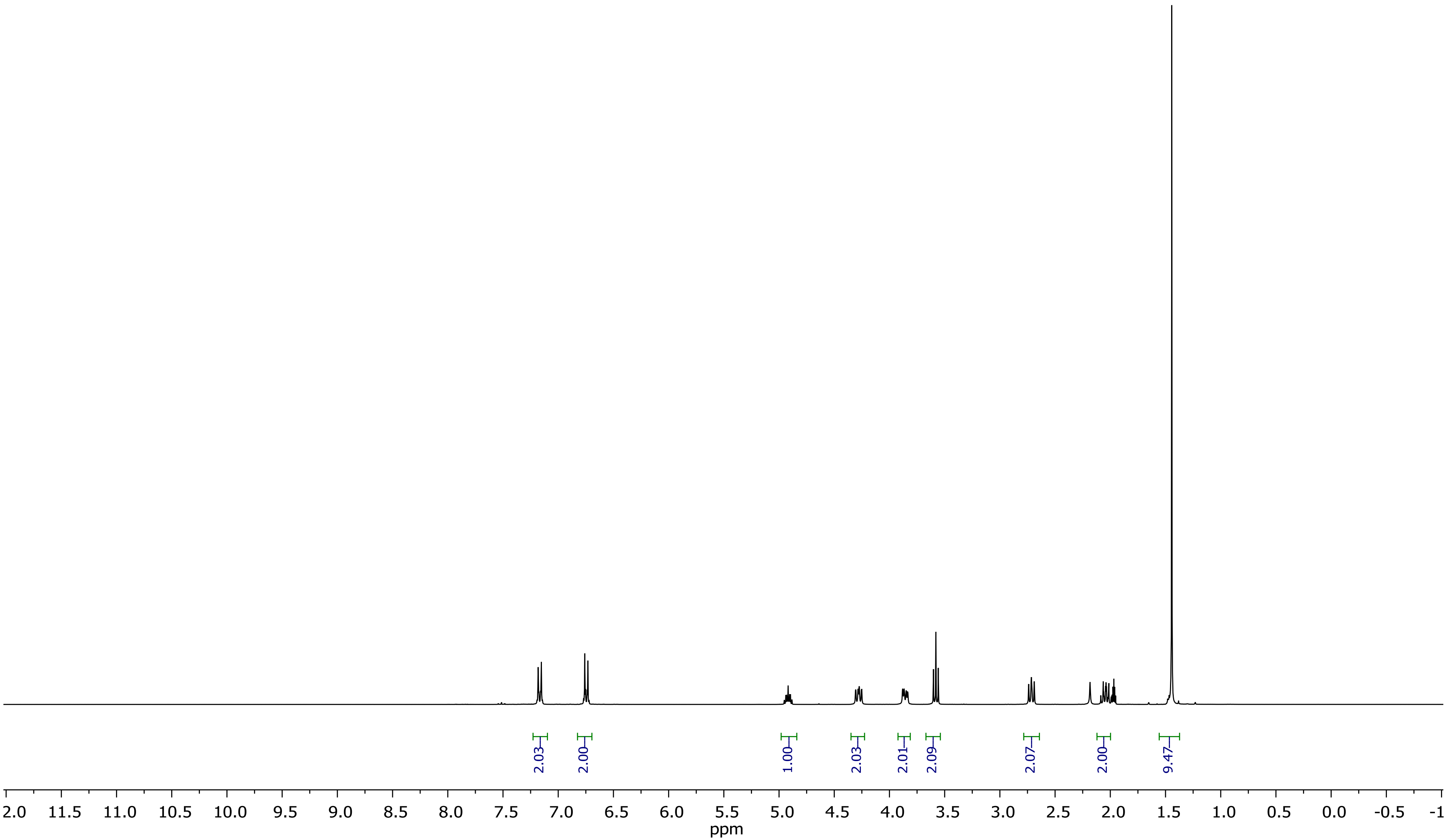

^{13}C NMR of *tert*-butyl 3-(4-(3-chloropropyl)phenoxy)azetidine-1-carboxylate (46)

CD_3CN, 25 °C

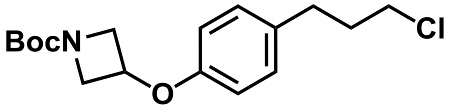

46

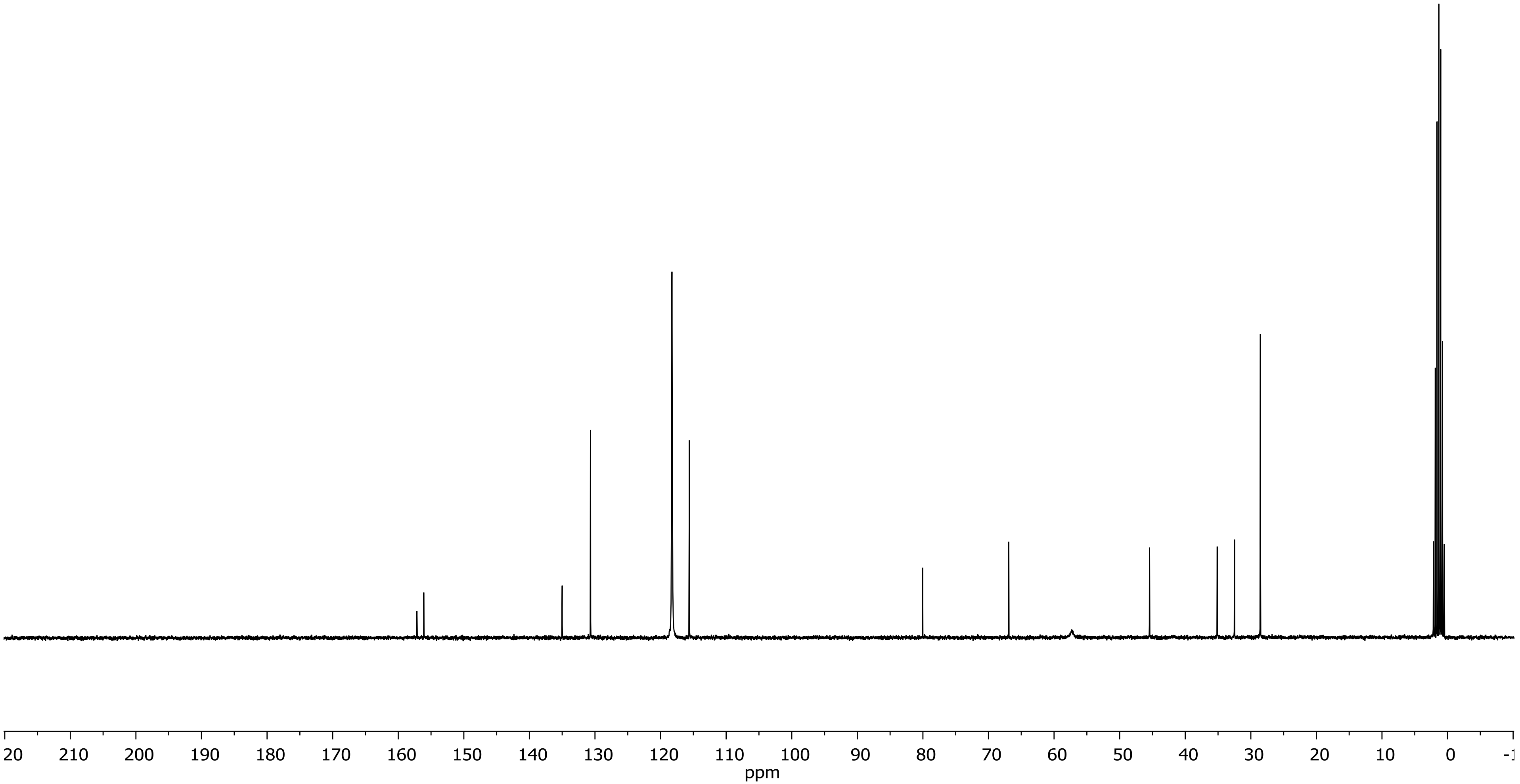

^{1}H NMR of 5-oxetan-3-yloxy boscalid (47)

$CDCl_3$, 25 °C

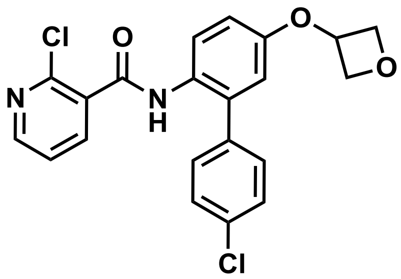

47

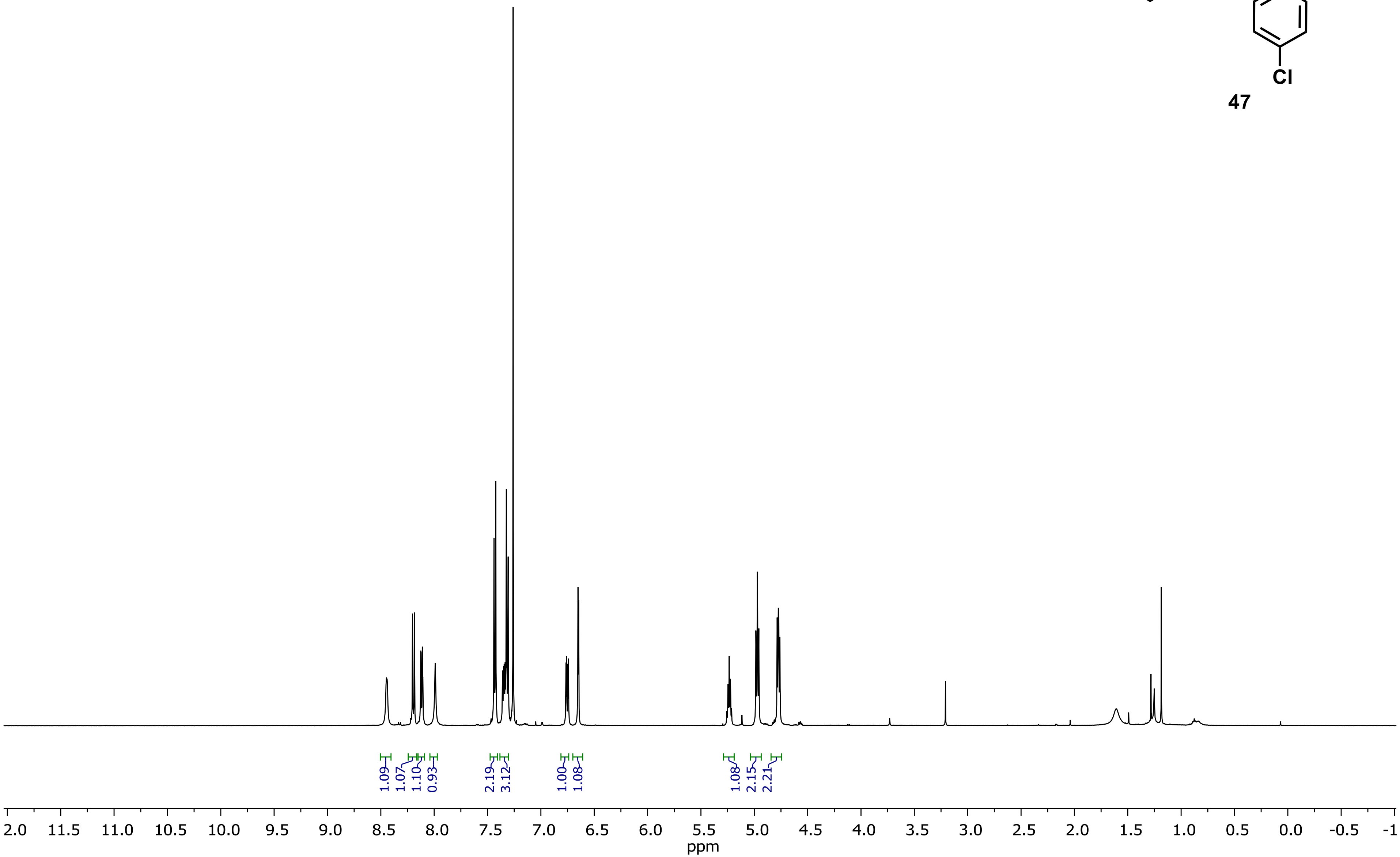

^{13}C NMR of 5-oxetan-3-yloxy boscalid (47)

$CDCl_3$, 25 °C

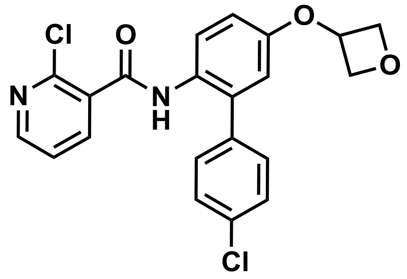

47

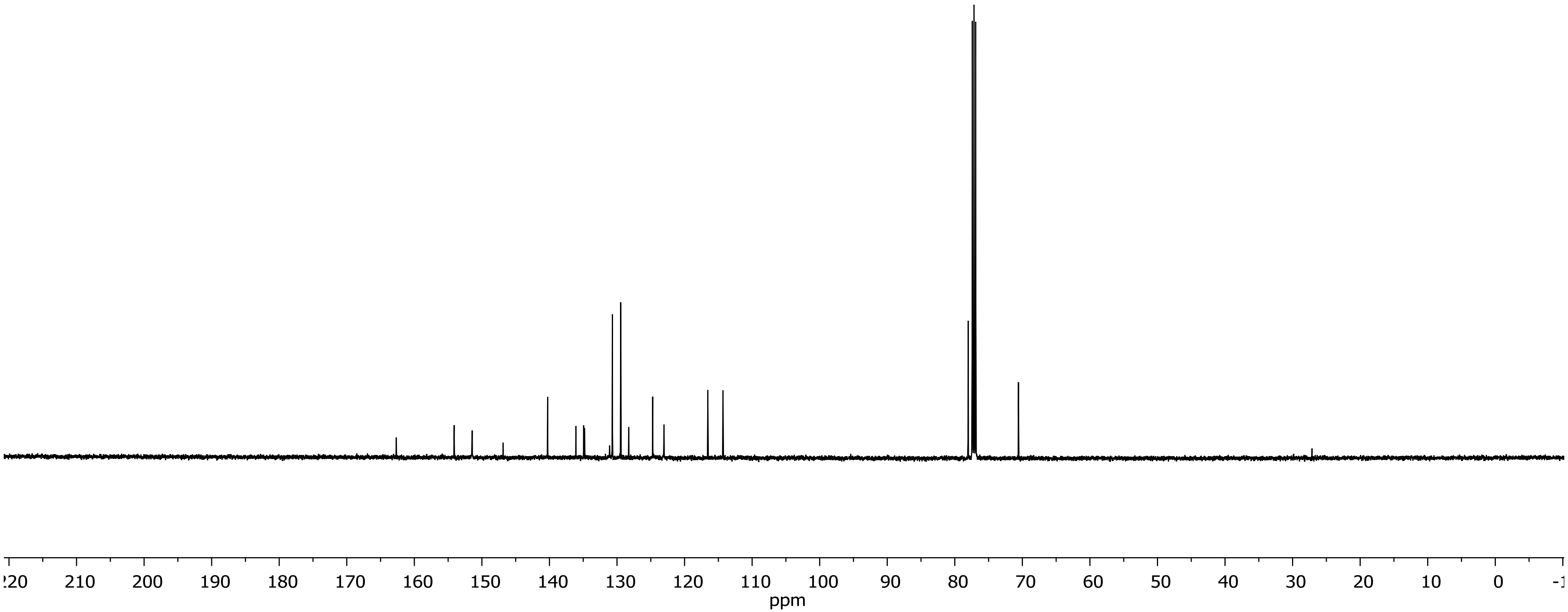

^{1}H NMR of 4-(cyclohexylthio)-1,1'-biphenyl (48)

$CDCl_3$, 25 °C

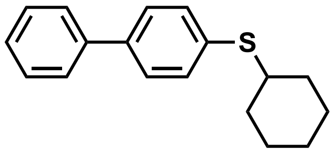

48

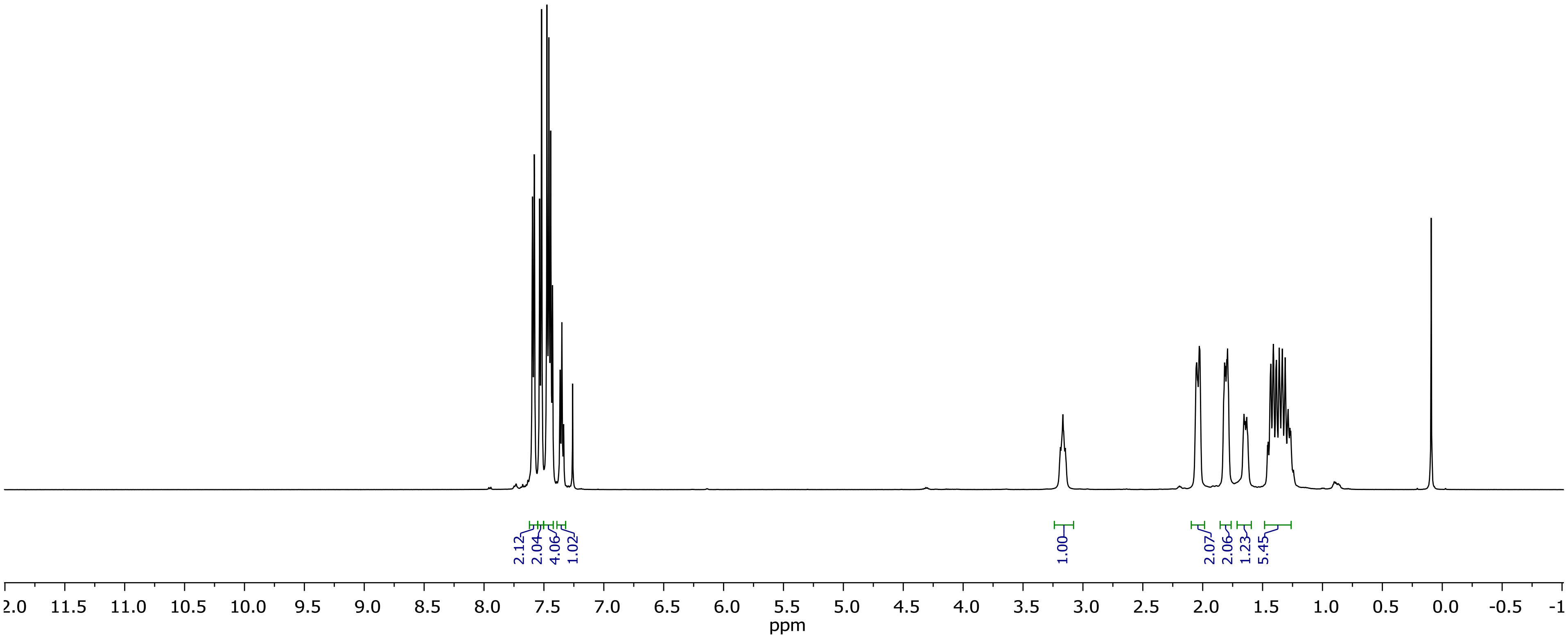

^{13}C NMR of 4-(cyclohexylthio)-1,1'-biphenyl (48)

$CDCl_3$, 25 °C

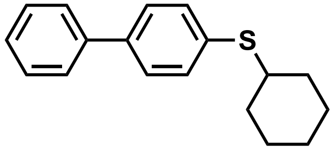

48

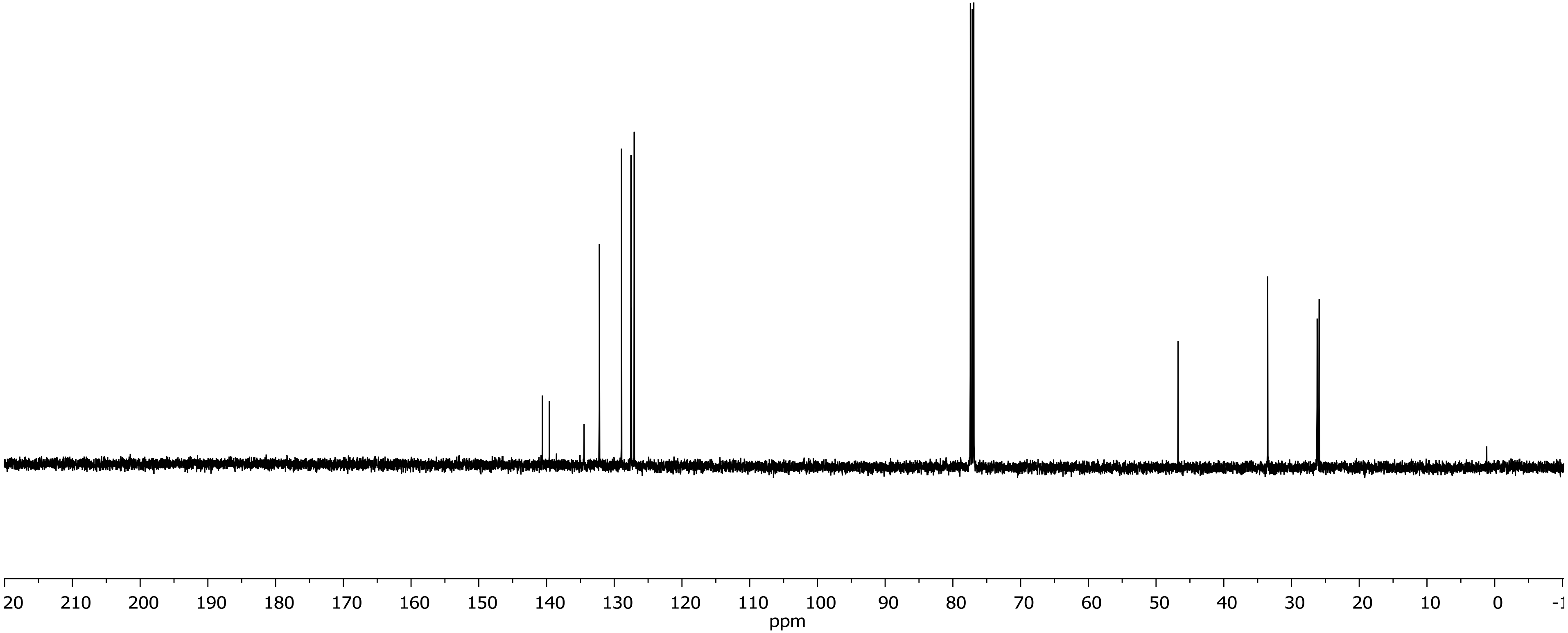

^{1}H NMR of 4-fluoro-1,1'-biphenyl (49-TT)

CD_3CN, 25 °C

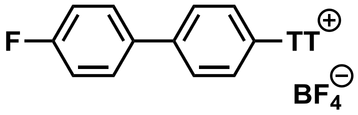

49-TT

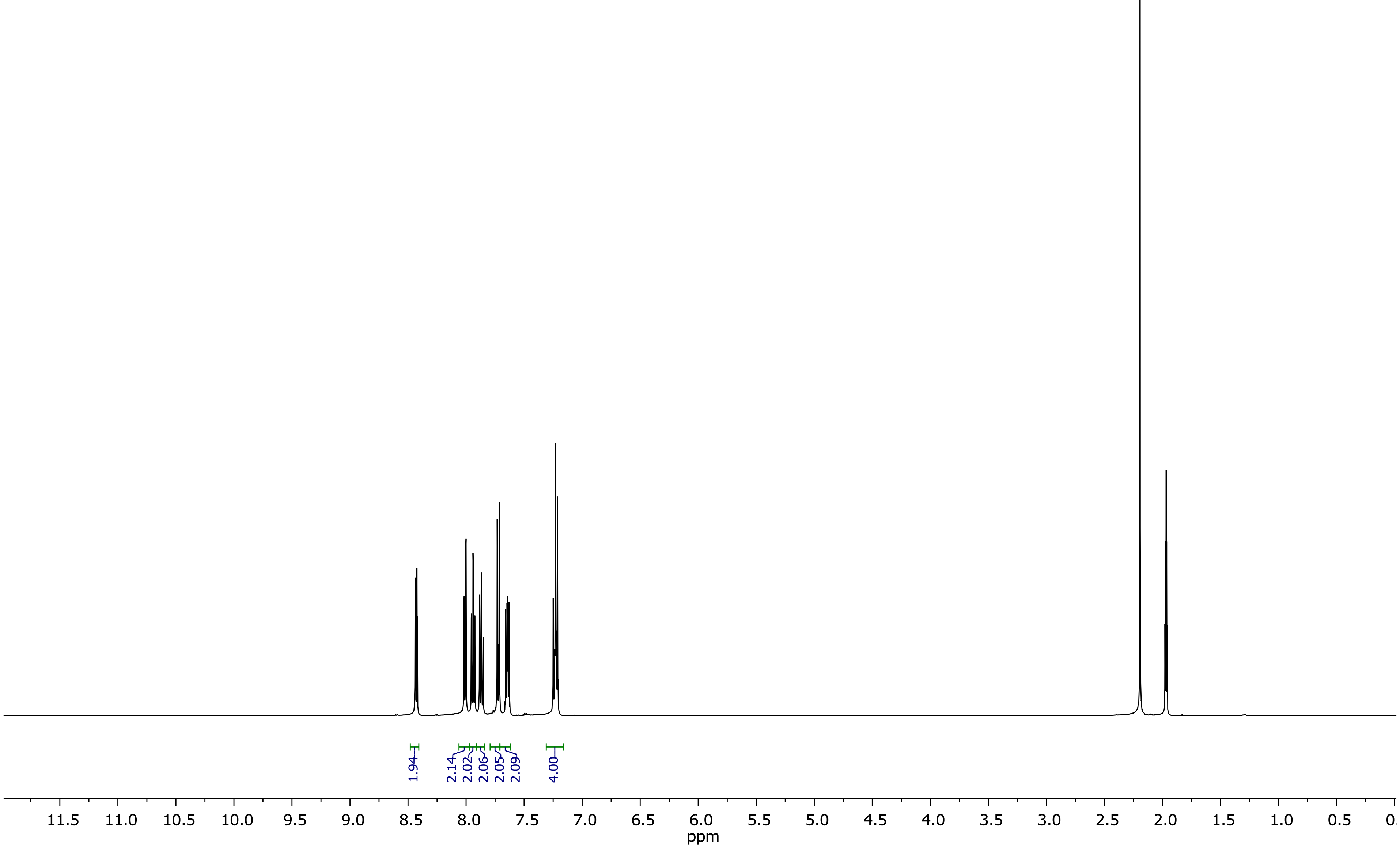

^{13}C NMR of 4-fluoro-1,1'-biphenyl (49-TT)

CD_3CN, 25 °C

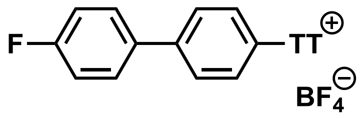

49-TT

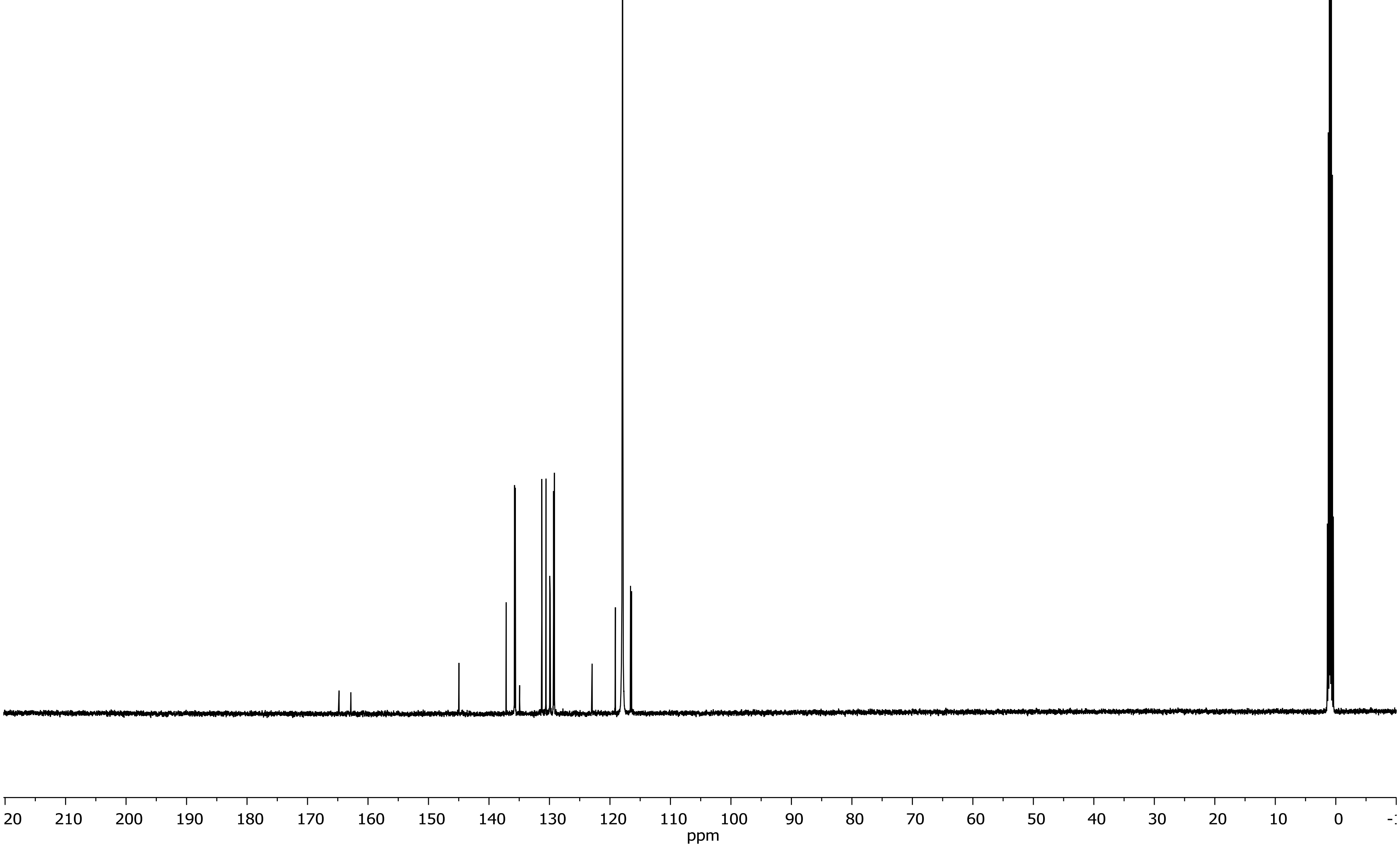

^{19}F NMR of 4-fluoro-1,1'-biphenyl (49-TT)

CD_3CN, 25 °C

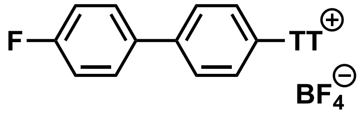

49-TT

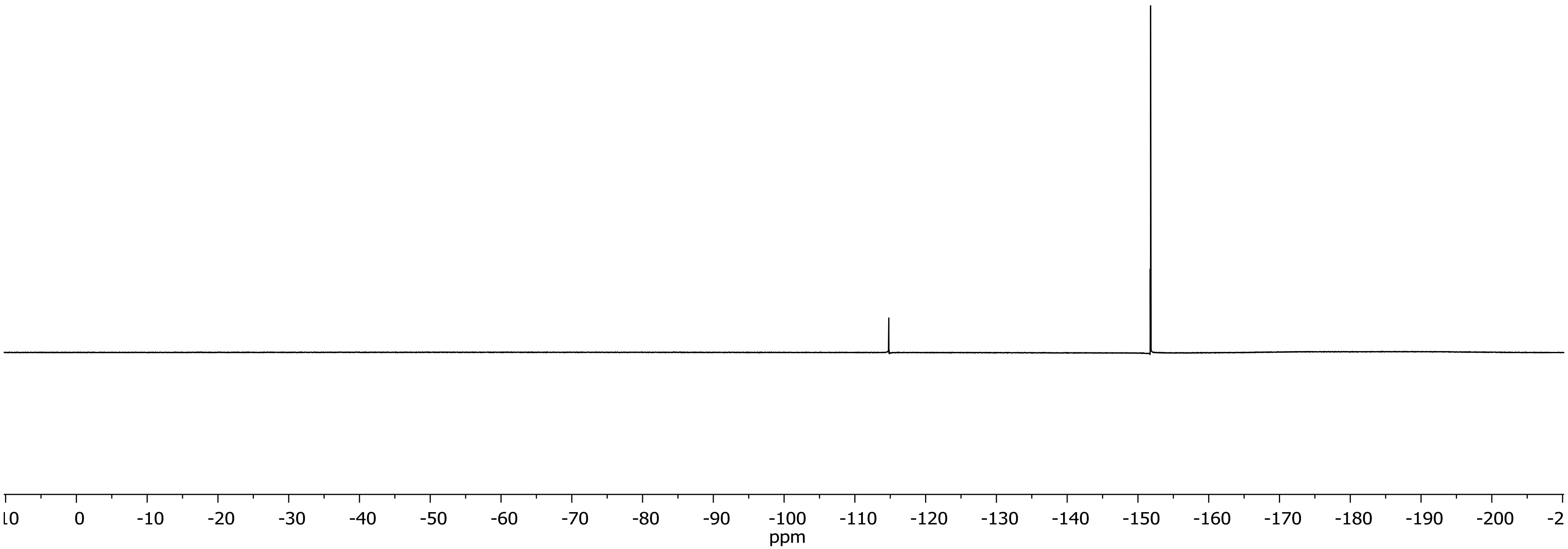

^{1}H NMR of cyclohexyl(4'-fluoro-[1,1'-biphenyl]-4-yl)sulfane (49)

$CDCl_3$, 25 °C

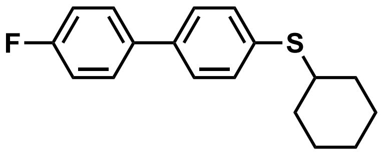

49

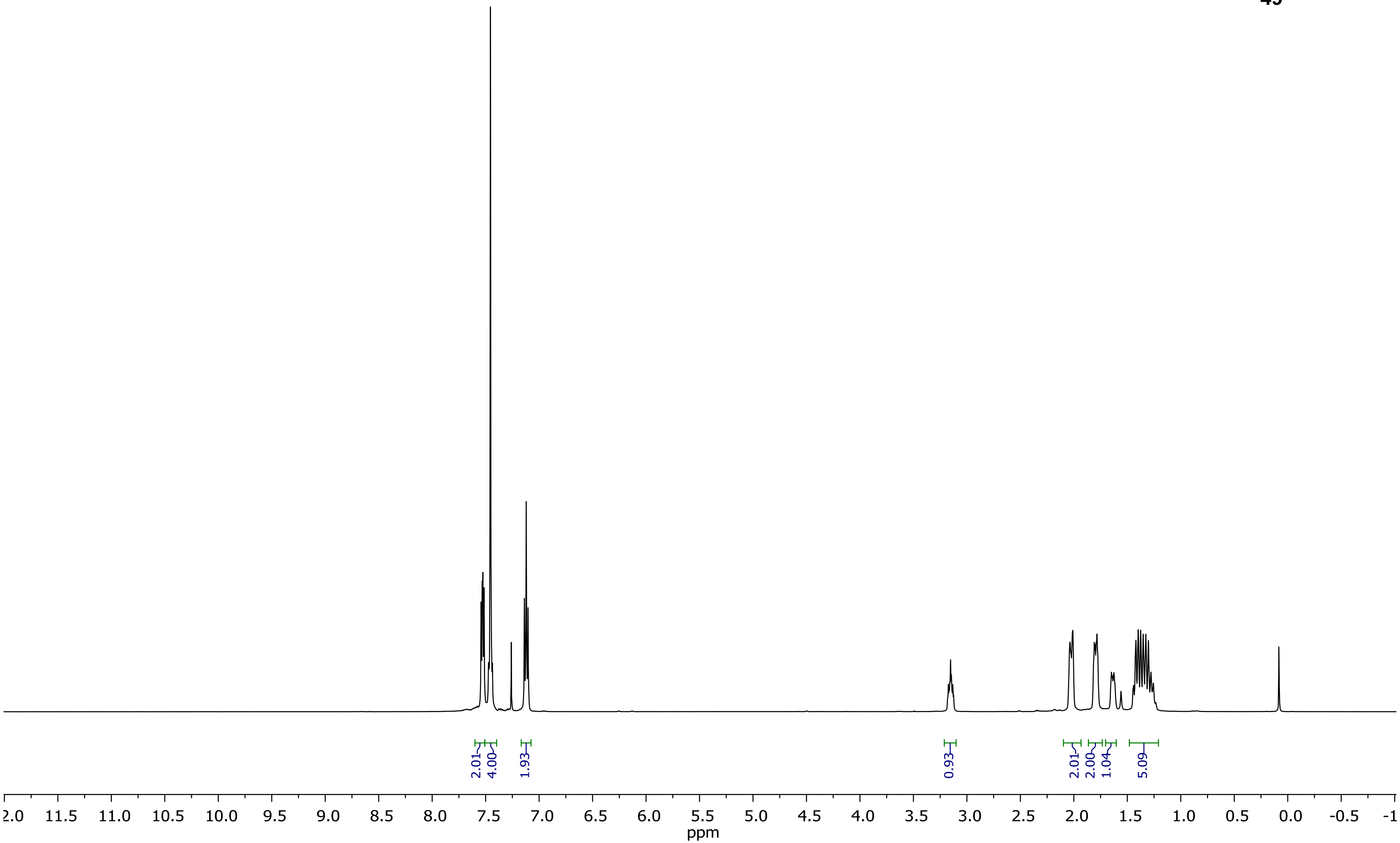

^{13}C NMR of cyclohexyl(4'-fluoro-[1,1'-biphenyl]-4-yl)sulfane (49)

$CDCl_3$, 25 °C

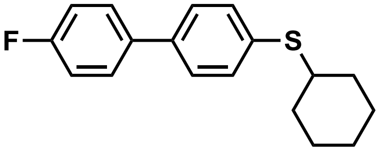

49

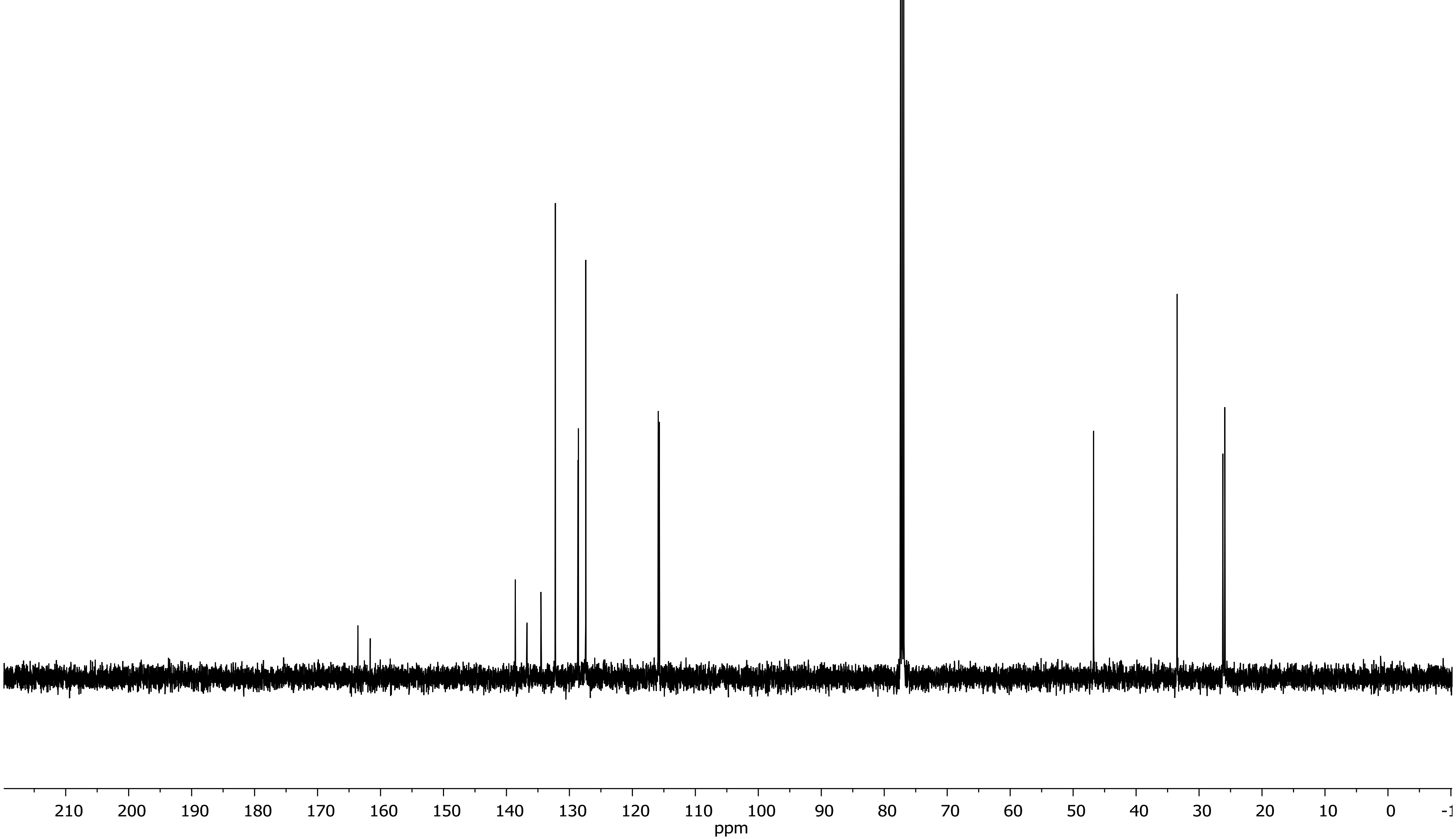

^{19}F NMR of cyclohexyl(4'-fluoro-[1,1'-biphenyl]-4-yl)sulfane (49)

CDCl$_3$, 25 °C

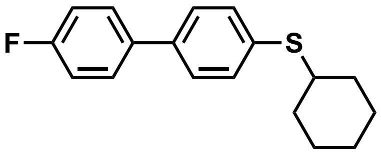

49

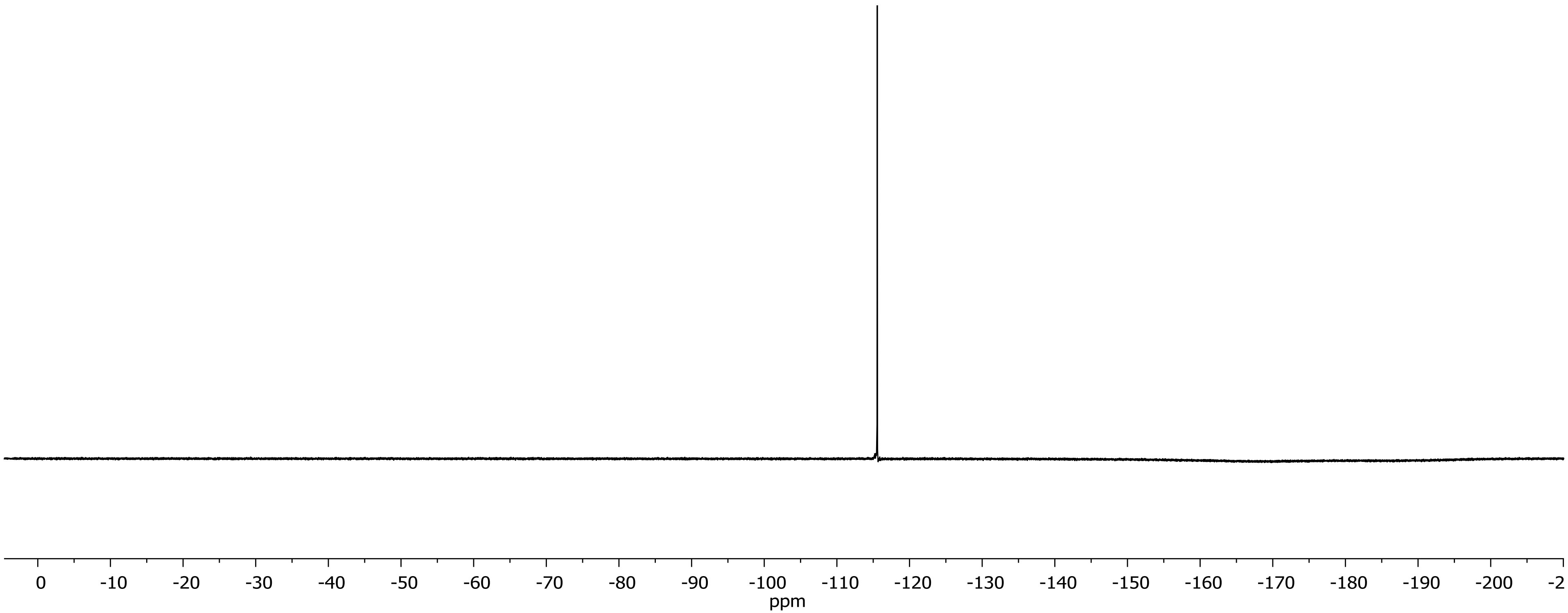

^{1}H NMR of 2-methoxy-5-(phenylthio)benzonitrile (50)

CD_3CN, 25 °C

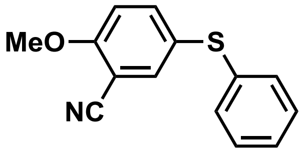

50

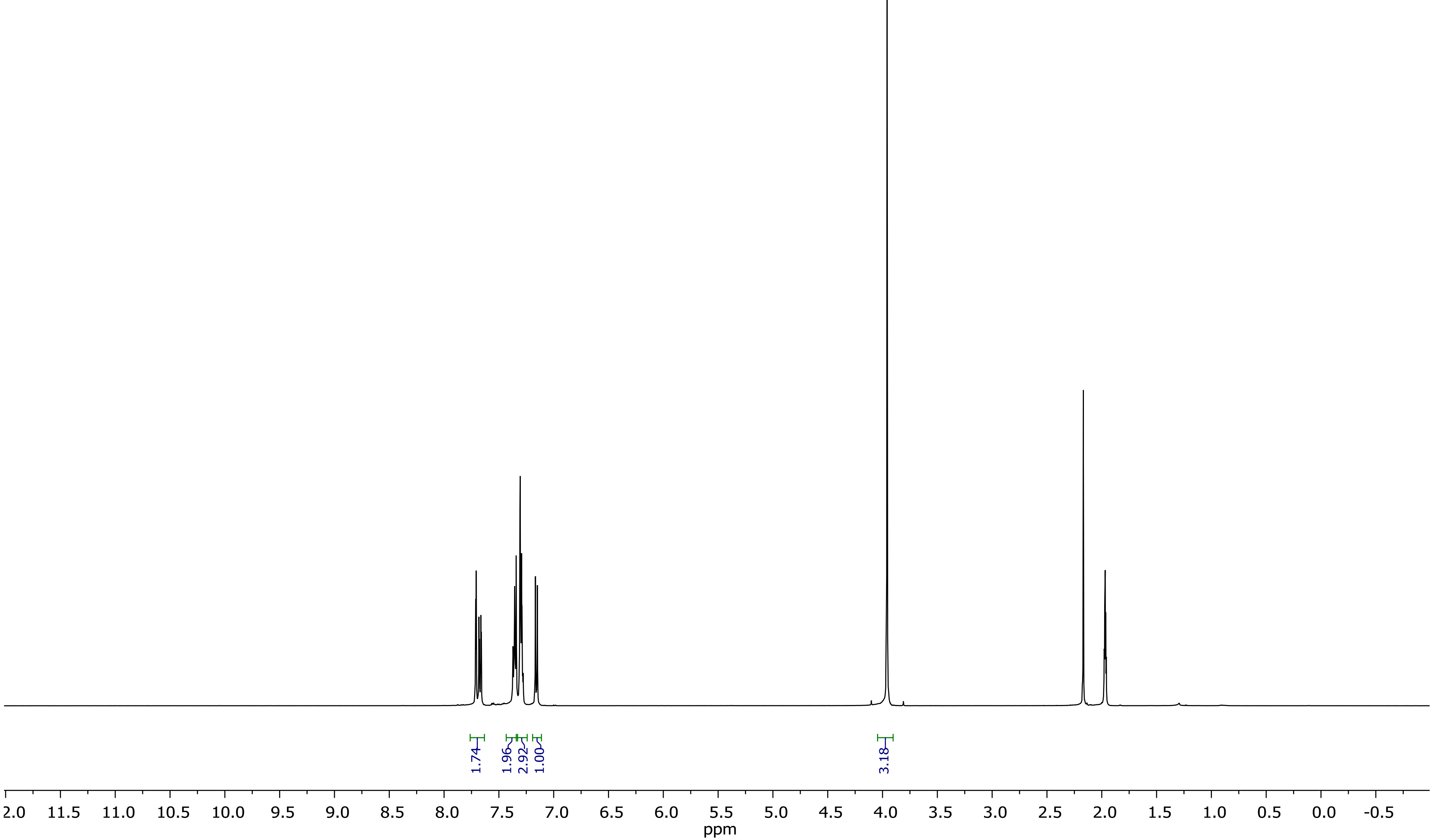

^{13}C NMR of 2-methoxy-5-(phenylthio)benzonitrile (50)

CD_3CN, 25 °C

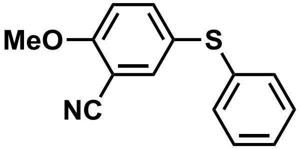

50

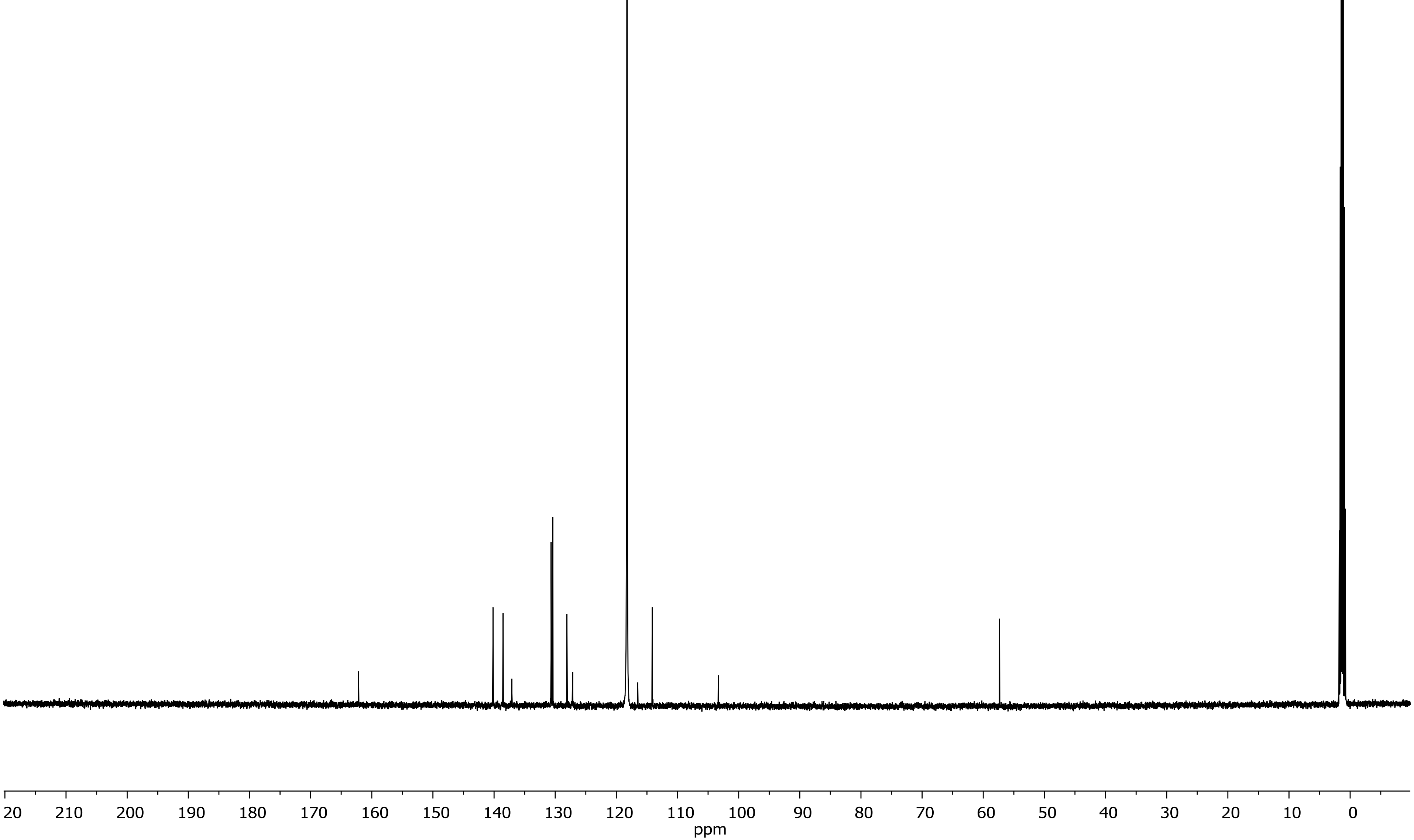

^{1}H NMR of phenylthio-etofenprox (51)

CD_3CN, 25 °C

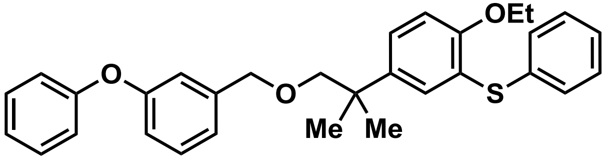

51

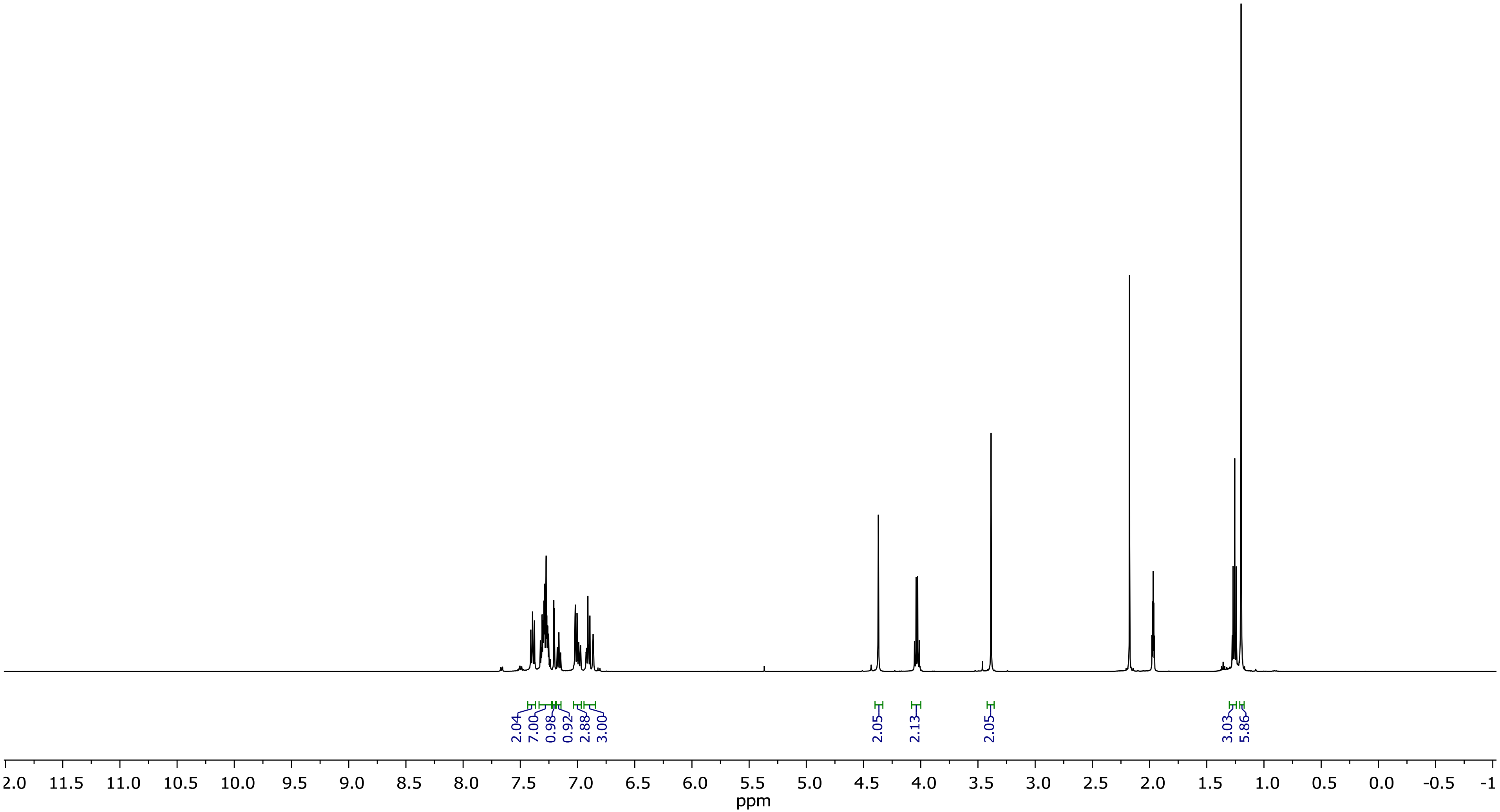

^{13}C NMR of phenylthio-etofenprox (51)

CD_3CN, 25 °C

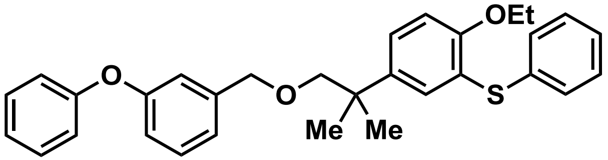

51

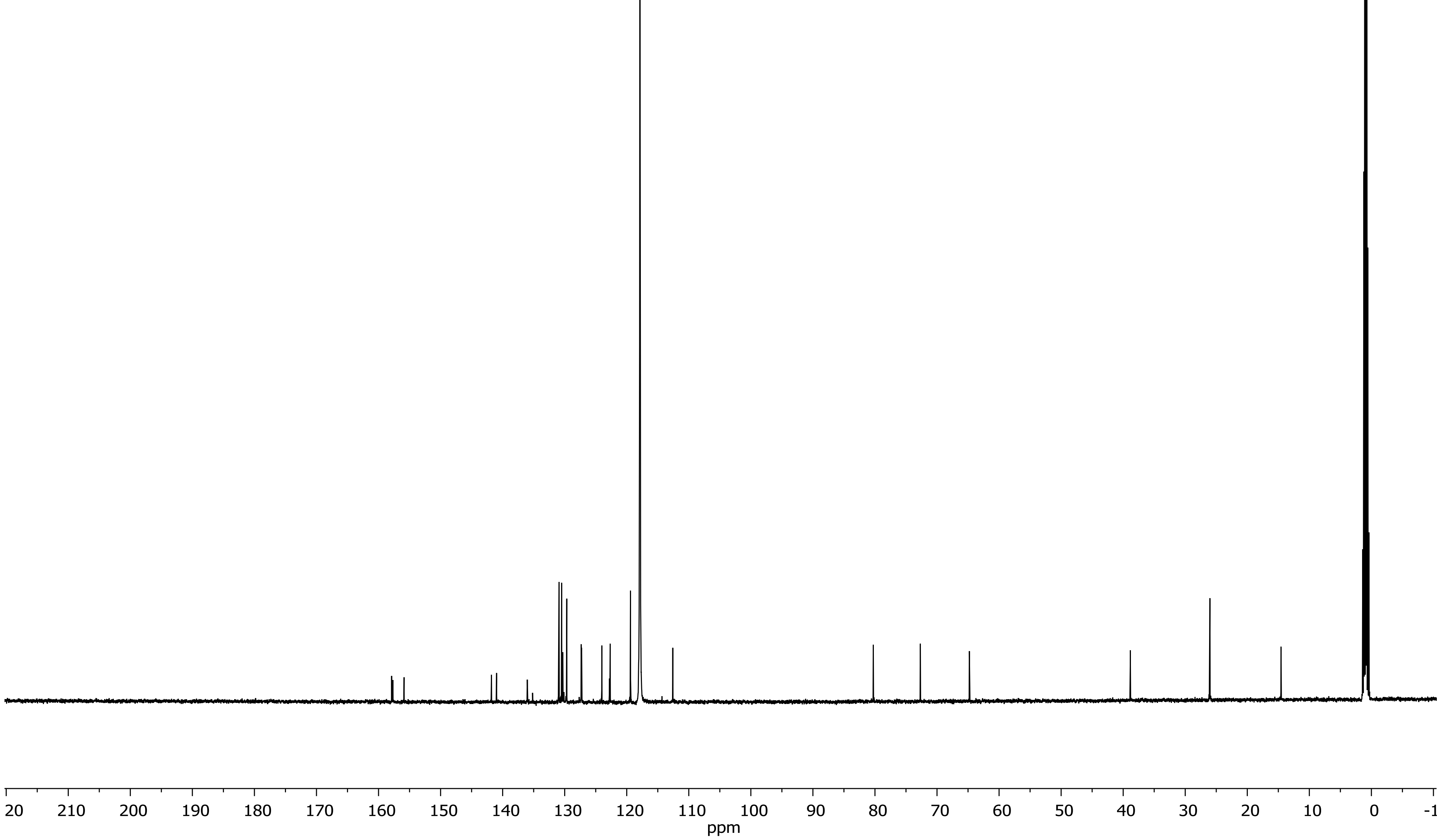

^{1}H NMR of ArTEMPO product (52)

CD_3CN, 25 °C

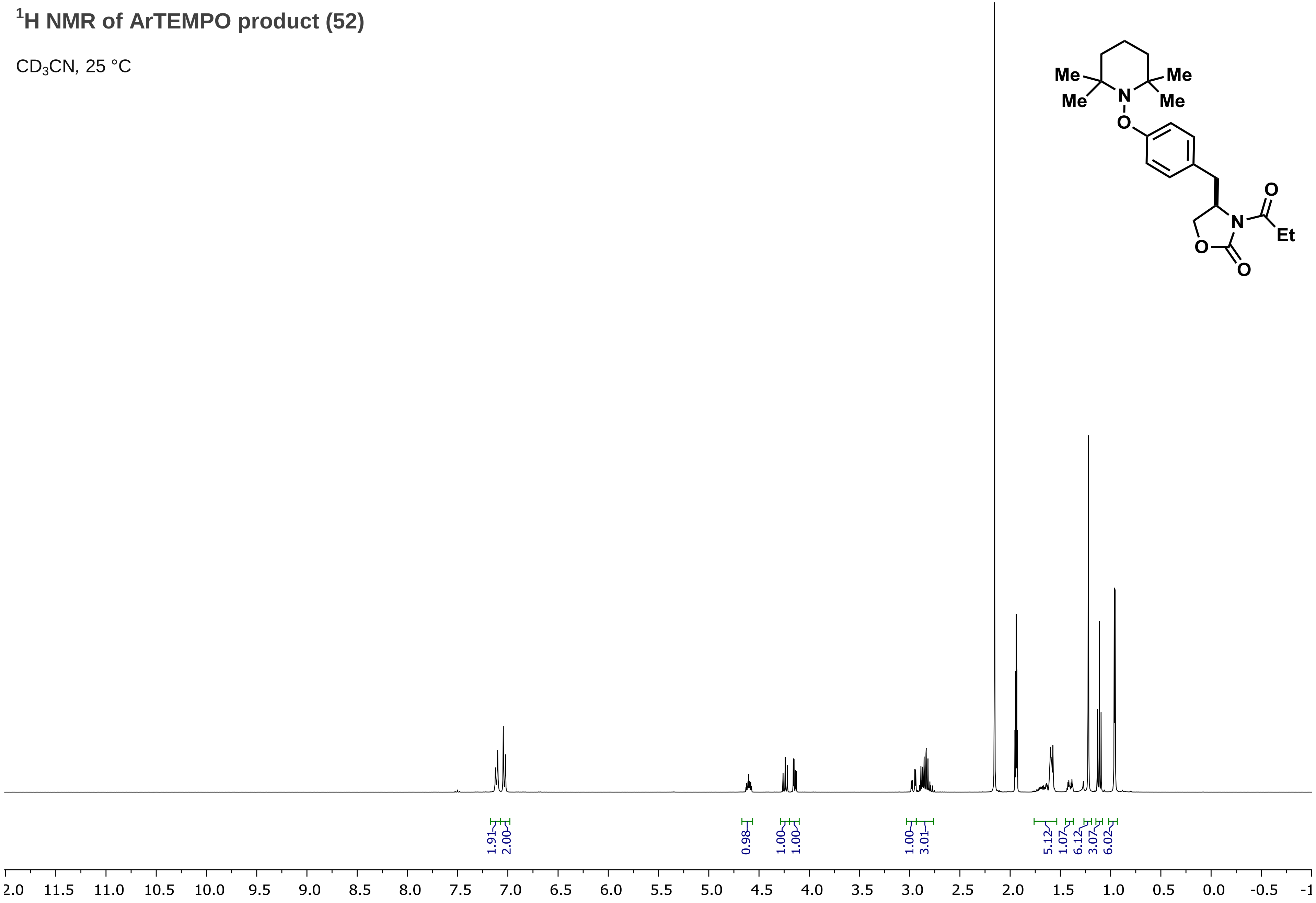

^{13}C NMR of ArTEMPO product (52)

CD_3CN, 25 °C

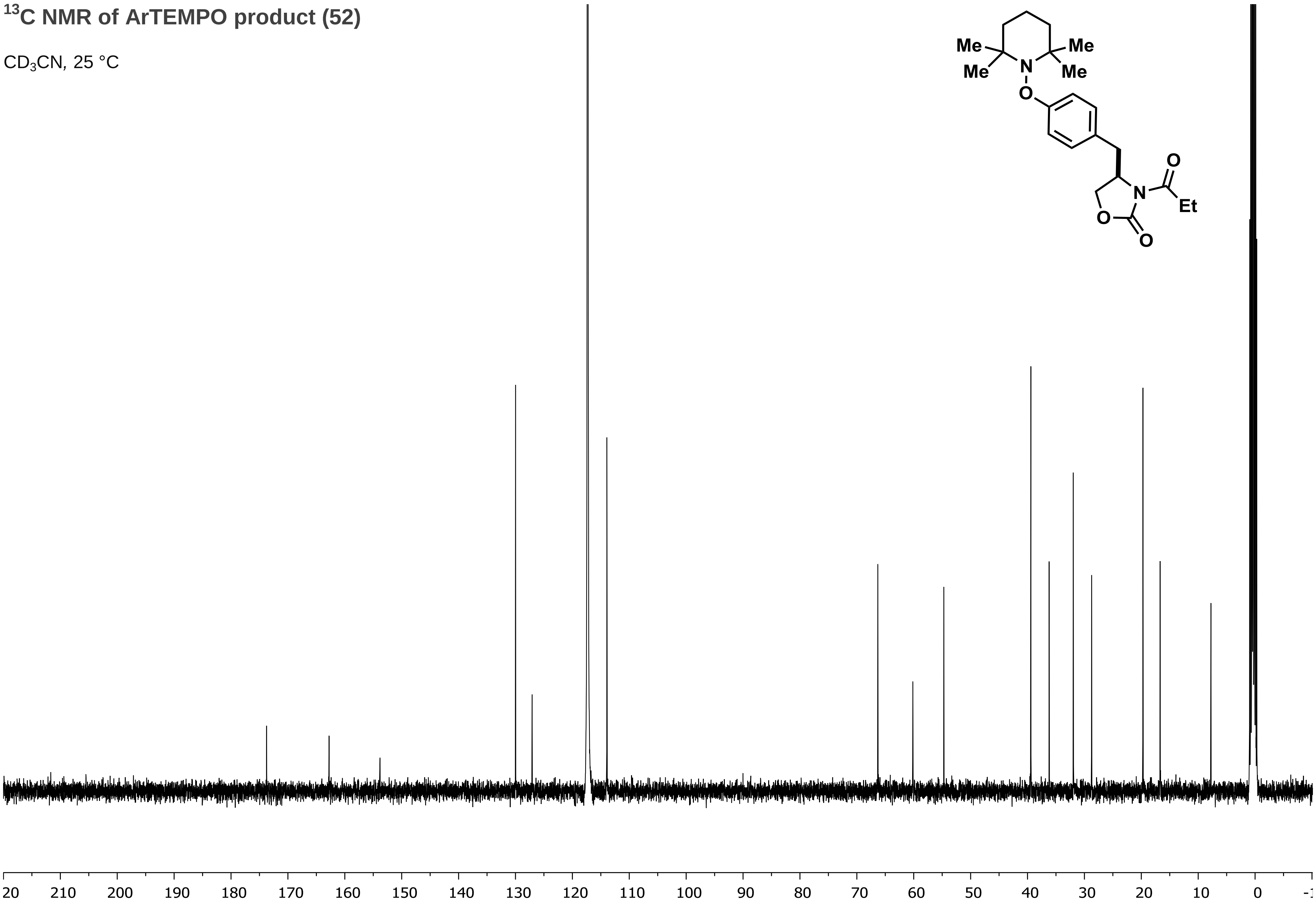

REFERENCES

1. F. Berger, M. B. Plutschack, J. Riegger, W. Yu, S. Speicher, M. Ho, N. Frank, T. Ritter, *Nature* **2019**, *567*, 223–228.
2. M. S. Lowry, J. I. Goldsmith, J. D. Slinker, R. Rohl, R. A. Pascal, G. G. Malliaras, S. Bernhard, *Chem. Mater.* ***2005*****,** 17, 5712–5719.
